# Liquid Crystals Comprising π-Electronic Ions from Porphyrin–Au^III^ Complexes

**DOI:** 10.1016/j.isci.2019.03.027

**Published:** 2019-04-01

**Authors:** Yohei Haketa, Yuya Bando, Yoshifumi Sasano, Hiroki Tanaka, Nobuhiro Yasuda, Ichiro Hisaki, Hiromitsu Maeda

**Affiliations:** 1 Department of Applied Chemistry, College of Life Sciences, Ritsumeikan University, Kusatsu 525–8577, Japan; 2 Research and Utilization Division, Japan Synchrotron Radiation Research Institute, Sayo 679–5198, Japan; 3 Green Nanotechnology Research Center, Research Institute for Electronic Science, Hokkaido University, Sapporo 001–0020, Japan

**Keywords:** Chemistry, Supramolecular Chemistry, Electronic Materials

## Abstract

Porphyrin–Au^III^ complexes were found to act as π-electronic cations, which can combine with various counteranions, including π-electronic anions. Single-crystal X-ray analyses revealed the formation of assemblies with contributions of charge-by-charge and charge-segregated assemblies, depending on the geometry and electronic state of the counteranions. Porphyrin–Au^III^ complexes possessing aliphatic alkyl chains formed dimension-controlled ion-pairing assemblies as thermotropic liquid crystals, whose ionic components were highly organized by π–π stacking and electrostatic interactions.

## Highlights

• Ion pairs based on porphyrin–Au^III^ complexes as π-electronic cations were prepared
• Porphyrin–Au^III^ complexes formed ion-pairing assemblies in combination with anions
• Aliphatic substituents in the cations provided liquid crystalline mesophases

## Graphical Abstract

**Figure:**
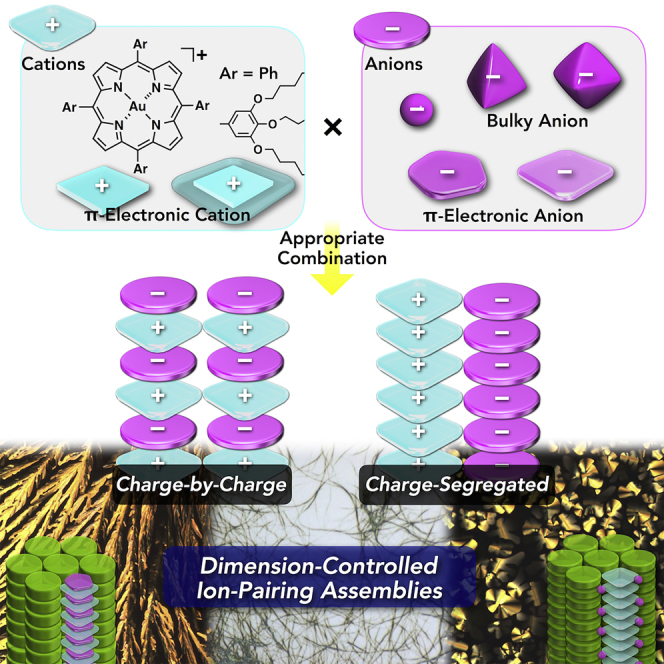

## Introduction

**Figure 1:**
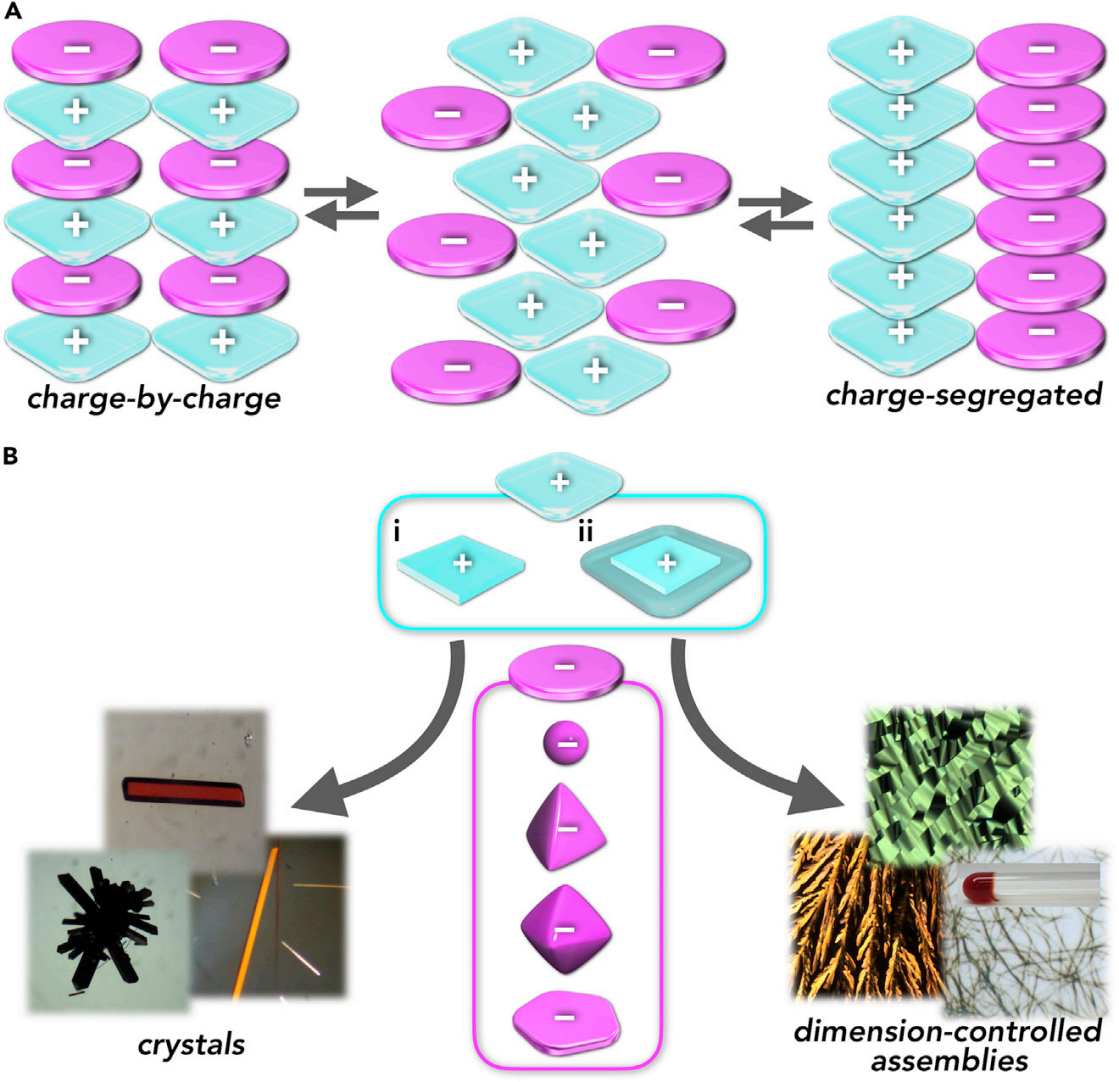
Conceptual Diagram of Ion-Pairing Arrangement and States (A and B) (A) Stacking assemblies of oppositely and identically charged π-electronic ions: charge-by-charge and charge-segregated assembling modes and their contributing assemblies and (B) schematic representation of the formation of ion-pairing assemblies (crystals and dimension-controlled assemblies) depending on the combination of appropriately modified cations ([i] non-aliphatic and [ii] aliphatic π-electronic cations) and anions. The assembling strategy in this study focuses on genuine π-electronic ions, whose core units have a charge, rather than the species bearing ionic moieties at the side chains.

An ordered arrangement of π-electronic species is crucial for the fabrication of functional organic materials such as organic electronic devices including field-effect transistors, light-emitting diodes, and photovoltaic cells (Würthner, 2005, Nakanishi, 2011, Koch, 2015). In contrast to the materials comprising single-species components, noncovalent interactions, such as hydrogen bonding, metal coordination, and donor-acceptor interactions, are required for the organization of complementary pairs of π-electronic molecules in materials (Kato et al., 2006). The obtained materials would exhibit diverse properties and functionalities according to the particular combinations of constituent building units. Synergetic uses of electrostatic interactions and other noncovalent interactions, including π–π stacking, is very important for the alignment of π-electronic charged species (cations and anions) and the formation of dimension-controlled assemblies including fiber and sheet solid materials, supramolecular gels, and liquid crystals (Faul, 2014, Goossens et al., 2016, Haketa and Maeda, 2017, Haketa and Maeda, 2018). An advantage of using electrostatic interactions is the formation of various ion-pairing materials by combining constituent π-electronic ions: for example, 10 kinds of cations and 10 kinds of anions are mixed to ideally provide 100 kinds of ion pairs. Furthermore, assembling modes can be modulated by constituent ions as well as by the environment, thus exhibiting particular properties according to the arrangement of charged building units even in an ion pair. Fundamentally, the stacking of oppositely and identically charged π-electronic ions results in charge-by-charge and charge-segregated assembling modes, respectively, as well as their contributing assemblies (Figure 1A) (Haketa and Maeda, 2017, Haketa and Maeda, 2018). Controlling these two characteristic assembling modes is an important issue for the fabrication of electronic materials. In addition, the combinations of cations and anions, not restricted to π-electronic ions, do not always afford ion pairs, whose production and state are significantly influenced by the geometries and electronic states of the constituent ions (Figure 1B). Thus the design and synthesis of charged species, along with their appropriate choice, are crucial for the examination of ion-pairing materials. Over the past few years, the formation of assemblies with charge-by-charge and charge-segregated contributions has been achieved based on π-electronic anions (Diels, 1942, Webster, 1965, Kuhn and Rewicki, 1967, Sakai et al., 2013) combined with appropriate cations in single crystals (Bruce et al., 1984, Bruce et al., 1986, Radhakrishnan et al., 1986, Watson et al., 1989, Jayanty and Radhakrishnan, 1999, Richardson and Reed, 2004, Less et al., 2010, Bando et al., 2016). For example, pentacyanocyclopentadienide (PCCp^–^) (Webster, 1965), a stable six π-electron aromatic anionic species with a planar geometry, afforded charge-by-charge assemblies with π-electronic cations in single crystals (Bruce et al., 1986, Bando et al., 2016) and also formed charge-segregated assemblies with bulky tetraalkylammonium cations in single crystals and liquid crystal mesophases (Less et al., 2010, Bando et al., 2016). Furthermore, ion-pairing assemblies, such as supramolecular gels and liquid crystals, were formed with pseudo π-electronic anions as complexes of π-electronic host molecules (receptors) and guest anions (Haketa et al., 2010, Haketa et al., 2012, Dong et al., 2012, Dong et al., 2013a, Dong et al., 2013b). More importantly, the above-mentioned soft materials based on π-electronic ion-pairing assemblies exhibit fascinating electric conductivity due to the contribution of charge-segregated assemblies (Dong et al., 2012, Dong et al., 2013a, Dong et al., 2013b, Haketa et al., 2012, Bando et al., 2016). Although the recent development of ionic self-assembly (Faul, 2014) has revealed potential applications for various functional nanostructured materials, there have been no studies of dimension-controlled ion-pairing assemblies based on charge-by-charge and charge-segregated assemblies, comprising genuine π-electronic cations and anions.

As porphyrin acts as a dianionic tetradentate ligand for various metal ions, trivalent metal cations can afford positively charged porphyrin–metal complexes as π-electronic cationic species in the absence of axial coordination. An important strategy is the use of Au^III^ (d^8^), which provides planar porphyrin–Au^III^ complexes as stable π-electronic cations that require no axial ligands (Fleischer and Laszlo, 1969, Timkovich and Tulinsky, 1977, Shachter et al., 1987, Brun et al., 1991, Kilså et al., 2001, Andersson et al., 2002, Che et al., 2003, Eng et al., 2005, Wang et al., 2007, Fortage et al., 2008, Sun et al., 2010, Ou et al., 2011, Ou et al., 2013, He et al., 2014, Preiß et al., 2016, Preiß et al., 2017), resulting in the potential formation of ion pairs with various anions. Thus far, porphyrin–Au^III^ complexes have been utilized as anticancer agents (Che et al., 2003, Wang et al., 2007, Sun et al., 2010, He et al., 2014), electron-accepting units in donor-acceptor systems (Brun et al., 1991, Kilså et al., 2001, Andersson et al., 2002, Eng et al., 2005, Fortage et al., 2008), and also as precursors for porphyrin–Au^II^ complexes (Ou et al., 2011, Ou et al., 2013, Preiß et al., 2016, Preiß et al., 2017). Although there is some research on the molecular structures and assemblies of porphyrin–Au^III^ complex ion pairs in single crystals (Timkovich and Tulinsky, 1977, Shachter et al., 1987, Che et al., 2003, Sun et al., 2010) and in irregularly shaped nanoscale aggregates (So et al., 2008), surprisingly, their dimension-controlled assemblies as soft materials have not been reported. Peripheral modifications of porphyrins enable the modulation of the structures and properties of porphyrin–Au^III^ complexes (π-electronic cations), which significantly affect the assembling states. In the past few decades, apart from porphyrin complexes, several cationic Au^III^ complexes have been reported, including cyclometalated complexes (Adams et al., 1991, Zhang et al., 2012, Ogawa et al., 2013, Yam et al., 2015, Kumar and Nevado, 2017). Among them, cationic Au^III^ complexes that provide dimension-controlled assemblies are very few, presumably because of their low thermal stability, difficulty for peripheral modification, and unsuitable combination with counteranions (Adams et al., 1991, Zhang et al., 2012, Ogawa et al., 2013). Therefore, there is a great advantage in utilizing porphyrin–Au^III^ complexes as the cationic components of stacking assemblies of π-electronic ions, especially for ordered columnar assemblies via the collaboration of π–π stacking and electrostatic interactions. Herein, the ion-pairing assemblies of porphyrin–Au^III^ complexes with various counteranions are investigated. This article reports the first example of dimension-controlled assemblies based on charge-by-charge and charge-segregated modes mainly as liquid crystals consisting of π-electronic systems, with both cationic and anionic building units.

## Results and Discussion

### Preparation of Ion Pairs

**Figure 2:**
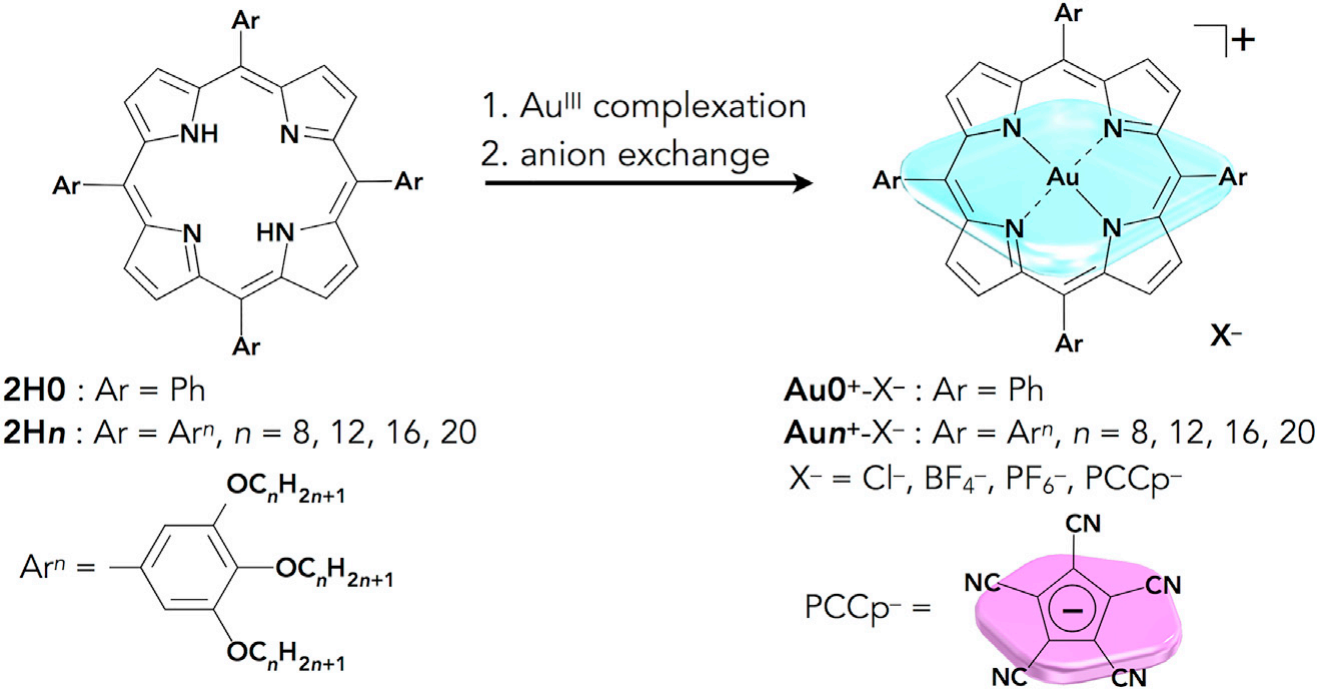
Synthesis of Porphyrin–Au^III^-Based Ion Pairs *Meso*-tetraarylporphyrin Au^III^ complexes: **Au0**^+^-X^–^ (X^–^ = Cl^–^, BF_4_^–^, PF_6_^–^, and PCCp^–^) and **Au*n***^+^-X^–^ (*n* = 8, 12, 16, and 20; X^–^ = Cl^–^, BF_4_^–^, PF_6_^–^, and PCCp^–^).

**Figure 3:**
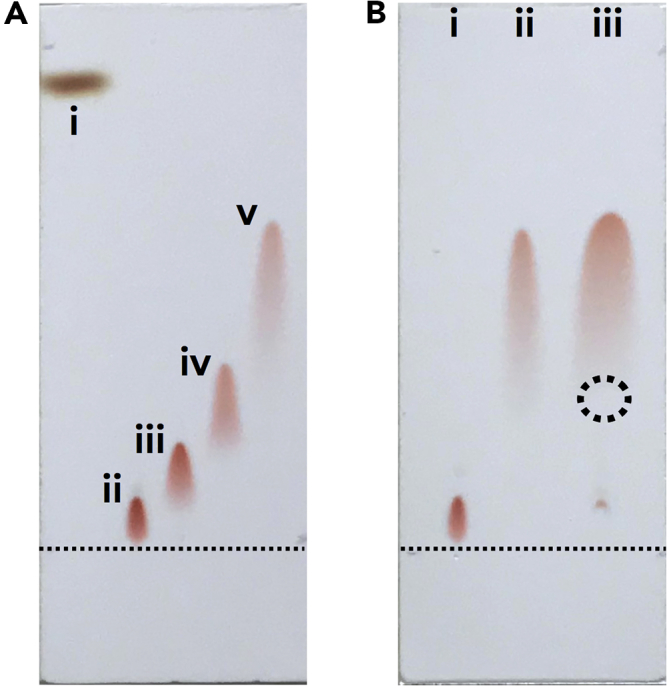
Thin-Layer Chromatography (TLC) Analysis for Ion Pairs TLC analysis for (A) (i) **2H0** as a reference, (ii) **Au0**^+^-Cl^–^, (iii) **Au0**^+^-BF_4_^–^, (iv) **Au0**^+^-PF_6_^–^, and (v) **Au0**^+^-PCCp^–^ and (B) (i) **Au0**^+^-Cl^–^, (ii) **Au0**^+^-PCCp^–^, and (iii) mixture of **Au0**^+^-Cl^–^ and TBAPCCp (2 equiv. to the samples in [i,ii] for each) using 5% MeOH/CH_2_Cl_2_ as an eluent. Dotted circles in (B) indicate the spot of TBAPCCp, which was observed under UV_254_ light. The detailed experimental procedures are described in the captions of Figures S2 and S3.

Porphyrin–Au^III^ complex cations paired with desired anions were prepared by Au^III^ complexation of porphyrins by treatment with KAuCl_4_ and a subsequent anion exchange with the obtained Cl^–^ ion pair of the porphyrin–Au^III^ complexes (Figure 2) (Che et al., 2003). For example, the Cl^–^ ion pair of a *meso*-tetraphenylporphyrin Au^III^ complex (**Au0**^+^-Cl^–^), prepared from **2H0**, was treated with 3 equiv. of Ag^+^ ion pairs of BF_4_^–^ and PF_6_^–^, followed by the removal of AgCl, silica gel column chromatography, and recrystallization with CH_2_Cl_2_/*n*-hexane, affording purified ion pairs **Au0**^+^-BF_4_^–^ and **Au0**^+^-PF_6_^–^ as red solids with yields of 70% and 55%, respectively. Treating **Au0**^+^-Cl^–^ with a Na^+^ ion pair of PCCp^–^ (Sakai et al., 2013) also afforded an ion pair **Au0**^+^-PCCp^–^, with an 82% yield, after the purification procedures. In this study, these counteranions were selected due to their characteristic geometries and electronic structures that can control assembling modes. It is noteworthy that the high stability of the ion pairs enables their purification through normal-phase silica gel columns and recrystallization under ambient conditions (see the detailed procedures in the Supplemental Information (Figures S1–S40)). Interestingly, the obtained ion pairs showed characteristic polarities according to coexisting counteranions (Cl^–^ > BF_4_^–^ > PF_6_^–^ > PCCp^–^) on silica gel chromatography (Figures 3A and S2). The characterization of the ion pairs, especially the determination of the 1:1 molar ratio of cationic **Au0**^+^ and corresponding anions, was conducted via elemental analysis. ^19^F nuclear magnetic resonance (NMR) was also measured to determine molar ratios for the ion pairs with BF_4_^–^ and PF_6_^–^. The ion pairs **Au0**^+^-X^–^ (X^–^ = Cl^–^, BF_4_^–^, PF_6_^–^, and PCCp^–^) showed similar ultraviolet-visible (UV-vis) absorption spectra in CH_2_Cl_2_ derived from **Au0**^+^ as a monomeric state with the maxima (λ_max_) of a Soret band at 409 nm (ɛ = ∼4 × 10^5^ M^−1^ cm^−1^) and a Q band at 521 nm (ɛ = ∼2 × 10^4^ M^−1^ cm^−1^) (Figure S40). The molecular orbitals (MO) of **Au0**^+^-PCCp^–^, investigated by density functional theory at B3LYP/6-31+G(d,p) with def2TZVP for Au, showed separately localized electron spin densities for **Au0**^+^ and PCCp^–^ (Figure S61) (Frisch et al., 2013). The theoretical studies support the fact that oppositely charged π-electronic ions exist as electronically independent species.

The polarities of the ion pairs depend on how delocalized negative charges the constituent anions have. Using different polarities, ion pairs based on porphyrin–Au^III^ cations can be separated by silica gel chromatography when two different ion pairs are mixed in one solution (Figure 3B). The ion pairs **Au0**^+^-Cl^–^ and **Au0**^+^-PCCp^–^ showed *R*_*f*_ values of 0.06 and 0.52, respectively, with 5% MeOH/CH_2_Cl_2_ as an eluent (i, ii). Thin-layer chromatography analysis of the 1:1 mixture of **Au0**^+^-Cl^–^ and PCCp^–^ as a tetrabutylammonium ion pair (TBAPCCp) in CH_2_Cl_2_ showed a distinct spot for **Au0**^+^-PCCp^–^, which was formed by ion exchange in a mixed CH_2_Cl_2_ solution, and small amounts of **Au0**^+^-Cl^–^ and TBAPCCp ([iii], Figure S3). The size of the spot of **Au0**^+^-PCCp^–^ in (iii) is approximately twice of that in (ii). This result clearly showed the preferential formation of **Au0**^+^-PCCp^–^ along with undetectable TBACl during the ion-exchange process.

### Solid-State Structures of Ion Pairs

**Figure 4:**
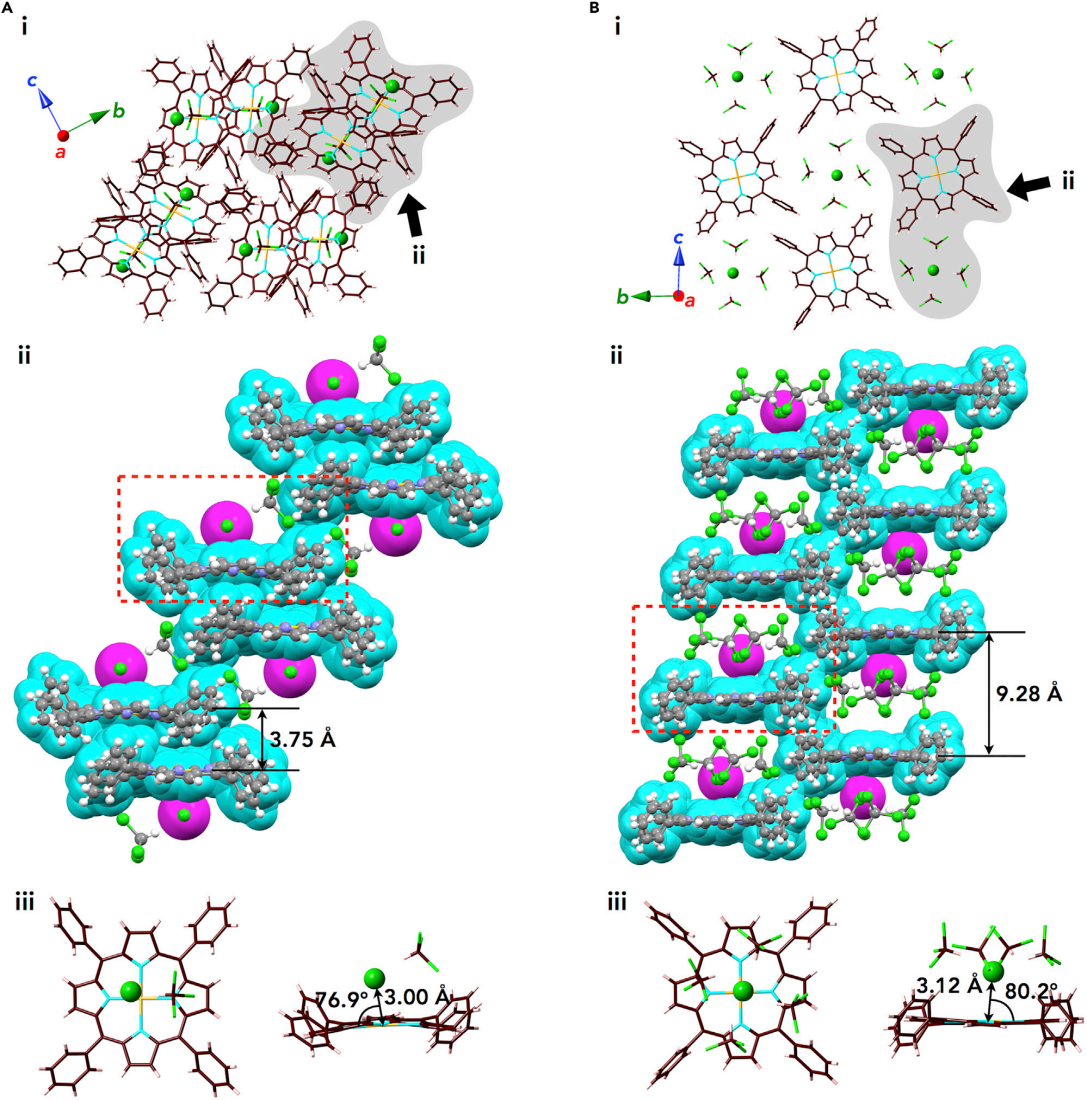
Single-Crystal X-Ray Structures of Au0^+^-Cl^–^ (A and B) (A) Type A and (B) type B: (i) representative packing modes as top views of one layer, (ii) space-filling packing models as side views from the arrows shown in (i), and (iii) top and side views of enlarged ion pairs, as indicated by red-dashed areas in (ii). Atom color code in (i) and (iii): brown, pink, light blue, green, and light orange refer to carbon, hydrogen, nitrogen, chlorine, and gold, respectively. Color code in (ii): cyan and magenta represent cations and anions, respectively.

The exact structures and ion-pairing assemblies of **Au0**^+^-Cl^–^ in the solid state were elucidated by X-ray analysis, as an examination of a previous study (Timkovich and Tulinsky, 1977), wherein single crystals were prepared by vapor diffusion of CHCl_3_/*n*-hexane (Figures S41 and S42, crystallographic details of all crystals are summarized in Table S1). Two crystal pseudo-polymorphs (type A and B) were obtained from the same crystallization conditions. In both packing types, columnar structures based on a charge-by-charge assembly were observed for **Au0**^+^ and Cl^–^ associated with co-crystallized CHCl_3_ molecules (Figure 4). In the type A polymorph, a two-by-two charge-by-charge structure was formed, based on repeating arrangement of a pair of **Au0**^+^-Cl^–^ and the stacking of two **Au0**^+^ planes with a distance of 3.75 Å (Figures 4A [ii] and S48). On the other hand, the type B polymorph showed a one-by-one charge-by-charge columnar structure of **Au0**^+^ and Cl^–^ associated with four CHCl_3_ molecules with a distance of 9.28 Å between two **Au0**^+^ planes (Figures 4B [ii] and S49). It is noteworthy that the proximal Au⋅⋅⋅Cl^–^ distances for type A and B polymorphs are 3.00 and 3.12 Å, respectively, which are comparable with the sum of the ionic radii of Au^3+^ and Cl^–^ (3.18 Å), suggesting the formation of contact ion pair (Figures 4A and 4B [iii]). Furthermore, the lines passing through both Au and Cl have angles of 76.9° and 80.2° for the type A and B structures, respectively, not 90°, to the mean planes of **Au0**^+^ (core 25 atoms including Au). Therefore, Cl^–^ is not likely coordinated to the core Au^III^, but proximally located around **Au0**^+^ as a π-electronic cation by rather electrostatic interactions.

**Figure 5:**
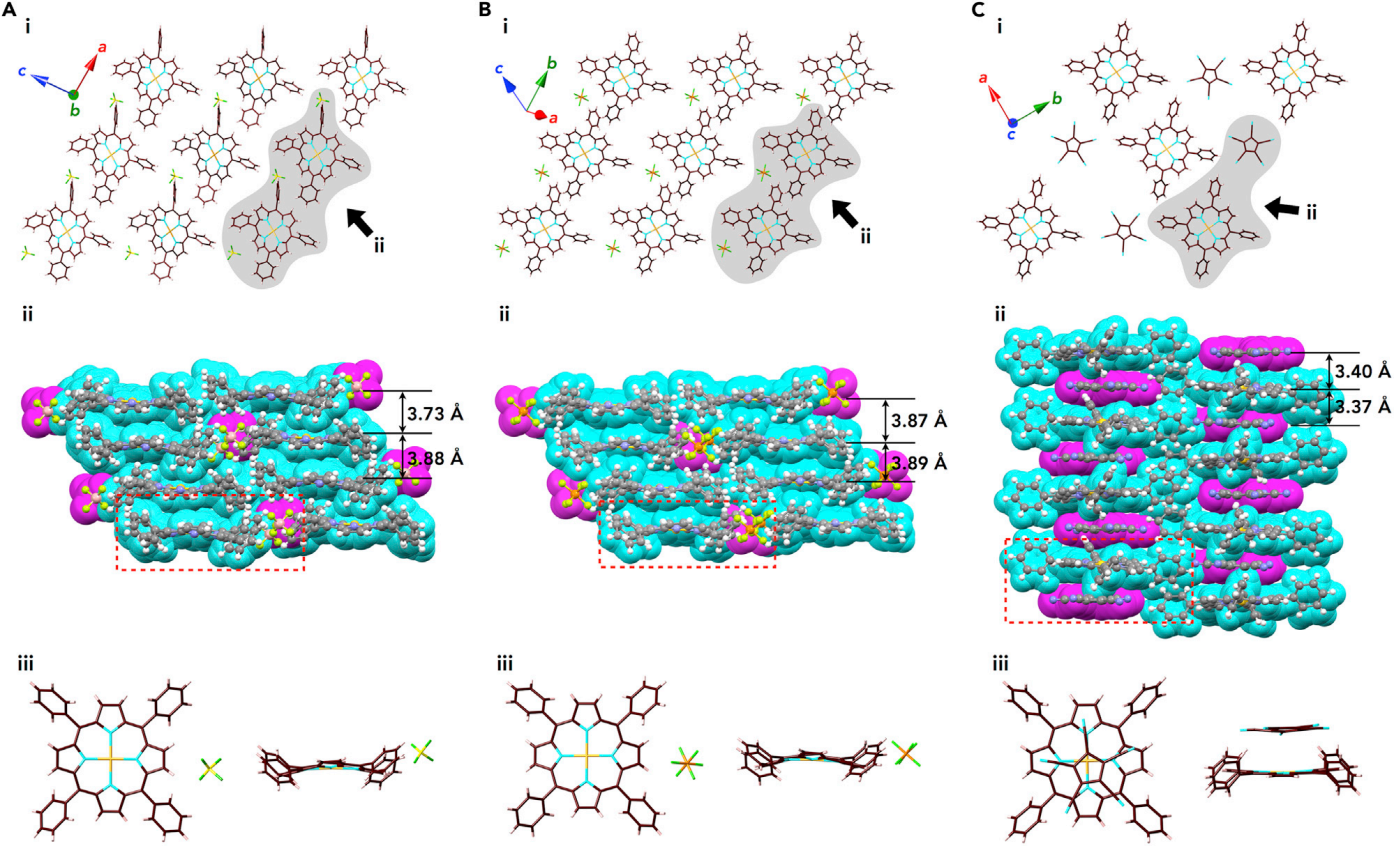
Single-Crystal X-Ray Structures of Au0^+^-X^–^ (X^–^ = BF_4_^–^, PF_6_^–^, and PCCp^–^) (A–C) (A) **Au0**^+^-BF_4_^–^, (B) **Au0**^+^-PF_6_^–^, and (C) **Au0**^+^-PCCp^–^: (i) representative packing modes as top views of one layer, (ii) space-filling packing models as side views from arrows in (i), and (iii) top and side views of enlarged ion pairs, as indicated by red-dashed areas in (ii). Solvent molecules are omitted for clarity. Atom color code in (i) and (iii): brown, pink, yellow, light blue, light green, orange, and light orange refer to carbon, hydrogen, boron, nitrogen, fluorine, phosphorus, and gold, respectively. Color code in (ii): cyan and magenta represent cations and anions, respectively.

The solid-state structures of the ion pairs **Au0**^+^-BF_4_^–^ and **Au0**^+^-PF_6_^–^ were also elucidated by X-ray analysis of single crystals prepared by vapor diffusion of CH_2_Cl_2_/*n*-hexane and (CH_2_Cl)_2_/*n*-hexane, respectively (Figures S43 and S44). A columnar structure comprising stacked **Au0**^+^ was observed in the crystal of **Au0**^+^-BF_4_^–^ with stacking distances of 3.73 and 3.88 Å between the porphyrin mean planes (core 25 atoms) and Au⋅⋅⋅Au distances of 5.05 and 5.48 Å (Figures 5A and S50). The counter BF_4_^–^ anion was located beside the **Au0**^+^ stacking columns, forming a charge-segregated assembly. Similar to **Au0**^+^-BF_4_^–^, **Au0**^+^-PF_6_^–^ formed a charge-segregated assembly with separately stacked **Au0**^+^, with distances of 3.87 and 3.89 Å, and PF_6_^–^ (Figures 5B and S51).

In contrast to the charge-segregated assemblies of **Au0**^+^-BF_4_^–^ and **Au0**^+^-PF_6_^–^, a charge-by-charge assembly was observed in the single crystal of **Au0**^+^-PCCp^–^, which was prepared by vapor diffusion of (CH_2_Cl)_2_/CH_3_CN (Figures 5C, S45, and S52). A columnar structure comprising alternately stacked **Au0**^+^ and PCCp^–^ was observed with stacking distances of 3.37 and 3.40 Å between the porphyrin mean planes (core 25 atoms) and PCCp^–^. The charge-by-charge column is fairly stabilized by π–π stacking, and electrostatic interactions worked for oppositely charged species. The Au⋅⋅⋅Au distance of 6.77 Å was almost equal to the sum of the stacking distances for **Au0**^+^ and PCCp^–^, suggesting an almost completely perpendicular stacking. The two farthest cyano nitrogens in a single component of PCCp^–^ have a distance of 7.20 Å, which is suitable for stacking with the porphyrin core planes, thus facilitating the alternate stacking process. The electrostatic potentials (ESP) of **Au0**^+^-PCCp^–^, calculated at B3LYP/6-31+G(d,p) with def2TZVP for Au based on the crystal structure, revealed absolutely small negative charges in **Au0**^+^ due to stacking with PCCp^–^ compared with **Au0**^+^-Cl^–^, where a more greater electron density was observed in **Au0**^+^ at the site proximal to Cl^–^ (Figures S55–S58) (Frisch et al., 2013).

### Dimension-Controlled Ion-Pairing Assemblies: Supramolecular Gels

**Figure 6:**
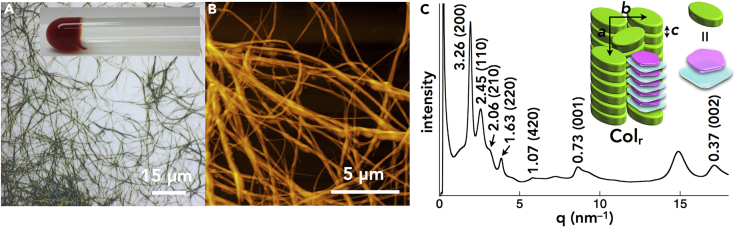
Characterization of the Xerogel of Au16^+^-PCCp^–^ (A–C) (A) Optical microscopic image (inset: photograph of the gel), (B) atomic force microscopic image, and (C) XRD pattern (inset: packing model) at RT of the xerogel of **Au16**^+^-PCCp^–^ prepared from *n*-octane gel (10 mg/mL).

To induce dimension-controlled ion-pairing assemblies in soft materials, aliphatic alkoxy chains were introduced at the *meso*-aryl moieties of porphyrin–Au^III^ cations. The Cl^–^ ion pairs **Au*n***^+^-Cl^–^ (*n* = 8, 12, 16, and 20), derived from the corresponding aliphatic porphyrins **2H*n*** (Maruyama et al., 2010, Nowak-Król et al., 2010) (Figure 2), were further anion exchanged and purified with silica gel followed by recrystallization with CH_2_Cl_2_/MeOH, resulting in ion pairs **Au*n***^+^-X^–^ (*n* = 8, 12, 16, and 20; X^–^ = BF_4_^–^, PF_6_^–^, and PCCp^–^) as air-stable red solids. Notably, this preparation process is easily adapted to the preparation of gram-scale ion pairs. Interestingly, the ion pairs formed anion-dependent nanostructured aggregates. For example, **Au16**^+^-PCCp^–^ formed a supramolecular gel in octane (10 mg/mL), comprising uniform fibers with diameters of ca. 0.5 μm and lengths of >50 μm, as revealed by optical and atomic force microscopic observations (Figures 6A, 6B, S64, and S65). **Au16**^+^-BF_4_^–^ also formed aggregates as a precipitate in octane (10 mg/mL) consisting of entangled fibers with diameters of 1–3 μm and lengths of >100 μm. Other aliphatic ion pairs **Au16**^+^-X^–^ (X^–^ = Cl^–^ and PF_6_^–^) showed no aggregation under these conditions. Synchrotron X-ray diffraction (XRD) analysis for the xerogel of **Au16**^+^-PCCp^–^ revealed the diffraction peaks of a rectangular columnar (Col_r_) structure with *a* = 4.90, *b* = 4.36, *c* = 0.73 nm, and *Z* = 2 (*ρ* = 0.83) based on a charge-by-charge assembly (Figures 6C, S121, and S122, and Table S20). Furthermore, the broader UV-vis absorption bands of **Au16**^+^-PCCp^–^ in octane (4 × 10^−6^ M) than those in CH_2_Cl_2_ suggested the formation of tightly bound ion pairs and resulting aggregates (Figure S66).

### Dimension-Controlled Ion-Pairing Assemblies: Thermotropic Liquid Crystals

**Figure 7:**
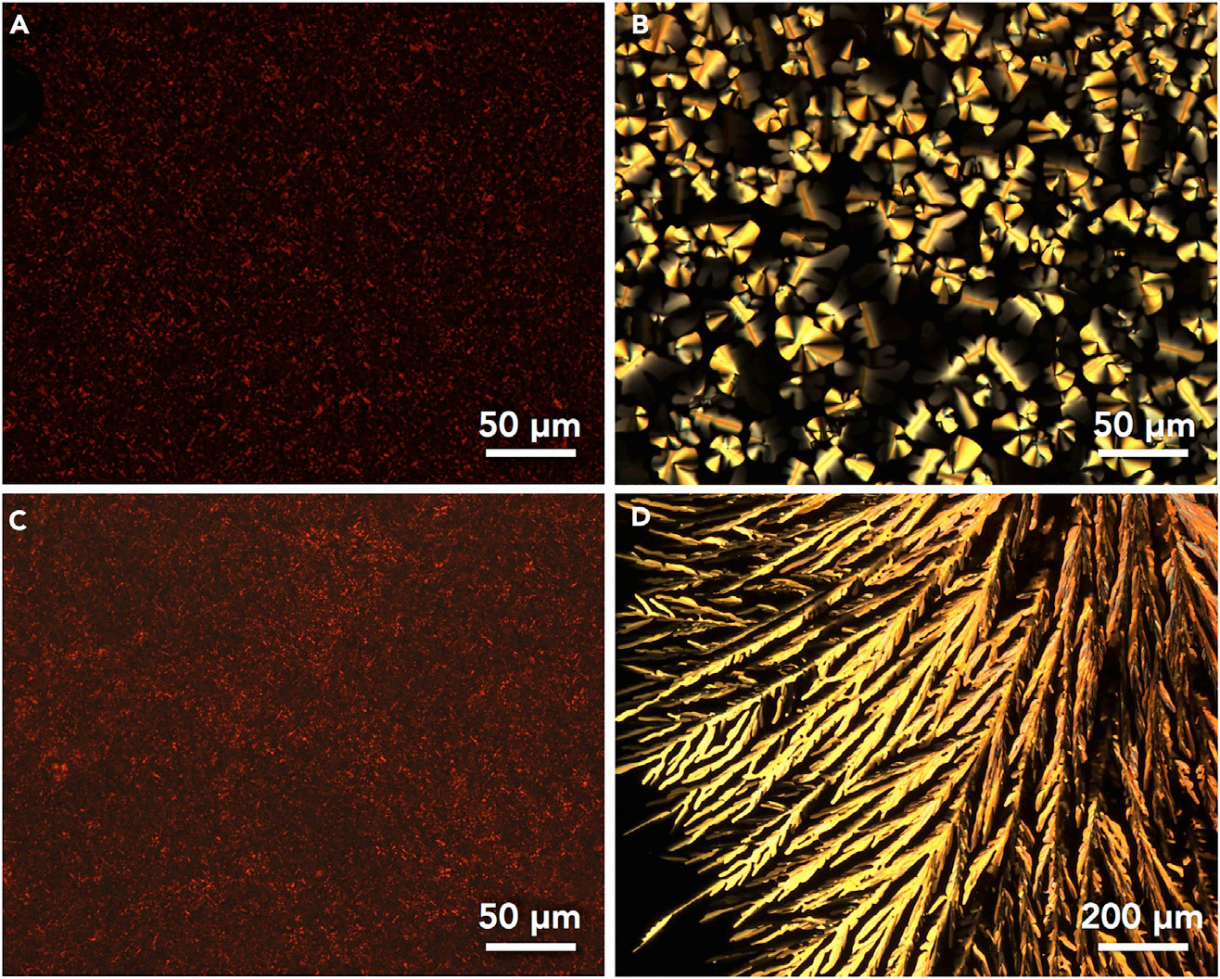
POM Observations (A–D) (A) **2H16**, (B) **Au16**^+^-Cl^–^, (C) **Au20**^+^-BF_4_^–^, and (D) **Au16**^+^-PCCp^–^ at 25°C, 100°C, 35°C, and 280°C, respectively, upon cooling.

Differential scanning calorimetry of the metal-free porphyrins **2H*n*** (*n* = 8, 12, 16, and 20) and ion pairs **Au*n***^+^-X^–^ (*n* = 8, 12, 16, and 20; X^–^ = Cl^–^, BF_4_^–^, PF_6_^–^, and PCCp^–^) revealed the formation of mesophases (Figures S67 and S68), whose transition temperatures are summarized in Table 1 (Table S2), as also observed in the corresponding polarized optical microscopic (POM) observations (Figure S69, also summarized in Figure S70). In the case of using precursory metal-free porphyrins as the reference species of the ion pairs, **2H8** exhibited no mesophases during thermal process, whereas **2H12** showed a transition to the lamellar state at −19°C upon cooling. On the other hand, **2H16** and **2H20** showed mesophase transitions, for example, at 26°C/17°C and 26°C/32°C for **2H16** upon cooling and heating, respectively, with the appearance of a fan-shaped POM texture (Figure 7A).

**Table 1:** Phase Transitions of Porphyrin–Au^III^-Based Ion Pairs^a^

Ion Pairs	Cooling^b^	Heating^b^
**2H*n***		

*n* = 8	Iso^c^	Iso^c^
*n* = 12	*lamellar* −19 Iso	*lamellar* −9 Iso
*n* = 16	*lamellar* 17^d^ lamellar 26 Iso	*lamellar* 26^d^ lamellar 32 Iso
*n* = 20	*lamellar* 43 lamellar 49 Iso	*lamellar* 53 Iso

**Au*n***^+^-Cl^–^		

*n* = 8	*Col*_*h*_ 122 Iso	*Col*_*h*_ 144^e^ Iso
*n* = 12	*Col*_*h*_ −22^d^ Col_h_ 120 Iso	*Col*_*h*_ −20^d^ Col_h_ 138^e^ Iso
*n* = 16	*Col*_*h*_ 37 Col_h_ 109^f^ Iso	*Col*_*h*_ 40 Col_h_ 111 Iso
*n* = 20	*Col*_*h*_ 53 Col_h_ 57 Col_h_ 61 Col_h_ 70 Iso	*Col*_*h*_ 60 Col_h_ 65 Col_h_ 73 Iso

**Au*n***^+^-BF_4_^–^		

*n* = 8	*Cr* 138 Iso	*Cr* 162 Iso
*n* = 12	amorphous −45^d^ Iso	amorphous −45^d^ Iso
*n* = 16	*Col*_*h*_ 19^d^^,^^g^ Iso	*Col*_*h*_ 20^d^ Col_h_ 61 Iso
*n* = 20	*Col*_*r*_ 48^d^ Col_h_ 63 Iso	*Col*_*r*_ 59^d^ Col_h_ 80 Iso

**Au*n***^+^-PF_6_^–^		

*n* = 8	*Cr* 148 Iso	*Cr* 161 Iso
*n* = 12	*Col*_*ob*_ −42^d^ Col_ob_ 75 Col_ob_ 105^e^ Iso	*Col*_*ob*_ −40^d^ Col_ob_ 113 Iso
*n* = 16	*lamellar* 10 Iso	*lamellar* 12^d^ Iso
*n* = 20	*lamellar* 39 Iso	*lamellar* 51 Iso

**Au*n***^+^-PCCp^–^		

*n* = 8	–h	–h
*n* = 12	–i	–i
*n* = 16	*Col*_*h*_ 36 Col_h_ 292 Iso	*Col*_*h*_ 43 Col_h_ 293 Iso
*n* = 20	*Col*_*r*_ 61 Col_h_ 260 Iso	*Col*_*r*_ 67 Col_h_ 262 Iso

a Iso presents isotropic liquid, and crystalline states are shown in italic.

b Transition temperatures (°C, the onset of the peak) from differential scanning calorimetry; first cooling and second heating scans (5°C min^−1^) and the examinations on first heating are excluded in the discussion.

c Evaluated from −100°C to 50°C.

d Peak top temperatures due to the broad differential scanning calorimetry peaks.

e Transition temperatures from POM.

f Transition temperatures from second cooling.

g Although there may be a transition at ∼0°C, the detailed examination on the possible mesophase was difficult.

h Decomposed at 383°C.

i Slightly transformed to other unidentified species after the transition to Iso at >300°C.

Compared with the porphyrins, the ion pairs **Au*n***^+^-Cl^–^ (*n* = 12 and 16) exhibited mesophases in wide temperature ranges; for example, **Au16**^+^-Cl^–^ showed transitions at 109°C/37°C and 40°C/111°C for cooling and heating, respectively, with the appearance of a focal conic POM texture (Figure 7B). **Au8**^+^-Cl^–^ showed no mesophase, whereas **Au20**^+^-Cl^–^ exhibited complicated mesophase transitions. On the other hand, **Au16**^+^-BF_4_^–^ showed a less clear mesophase between 20°C and 61°C upon heating, whereas **Au20**^+^-BF_4_^–^ showed thermal transitions at 63°C/48°C and 59°C/80°C upon cooling and heating, respectively (Figure 7C). Meanwhile, **Au*n***^+^-BF_4_^–^ (*n* = 8 and 12) exhibited no mesophases. **Au12**^+^-PF_6_^–^ exhibited mesophases with transition temperatures at 105°C/75°C/–42°C and −40°C/113°C upon cooling and heating, respectively, although the other PF_6_^–^ ion pairs showed no mesophases.

In sharp contrast to the less clear assembling behaviors of the nonplanar counteranions, the ion pairs **Au*n***^+^-PCCp^–^ (*n* = 16 and 20) exhibited clear mesophases. The mesophase temperatures for **Au*n***^+^-PCCp^–^ (*n* = 16 and 20) were observed at 292°C/36°C and 43°C/293°C and 260°C/61°C and 67°C/262°C upon cooling and heating, respectively, suggesting the existence of significantly wide-temperature-range mesophases compared with those for the other ion pairs investigated. The PCCp^–^ ion pairs exhibited dendritic POM textures, suggesting hexagonally arranged assemblies (Figure 7D). Interestingly, longer alkyl chains induced higher transition temperatures for the mesophases and lower clearing points, which can be explained by the compromise between the van der Waals interactions of aliphatic chains and the stacking assemblies of **Au*n***^+^ and PCCp^–^. In addition, the ion pair **Au8**^+^-PCCp^–^ showed no mesophase with a melting point at ca. 383°C, which corresponds to the decomposition of the ion pair, whereas **Au12**^+^-PCCp^–^ was slightly transformed to other unidentified species after the transition to isotropic liquid (Iso) at >300°C. The observations in **Au*n***^+^-PCCp^–^ (*n* = 8 and 12) also suggested that their stabilities as seen in the bulk states are maintained even at high temperatures.

### Structural Determinations of Ion-Pairing Liquid Crystals

**Figure 8:**
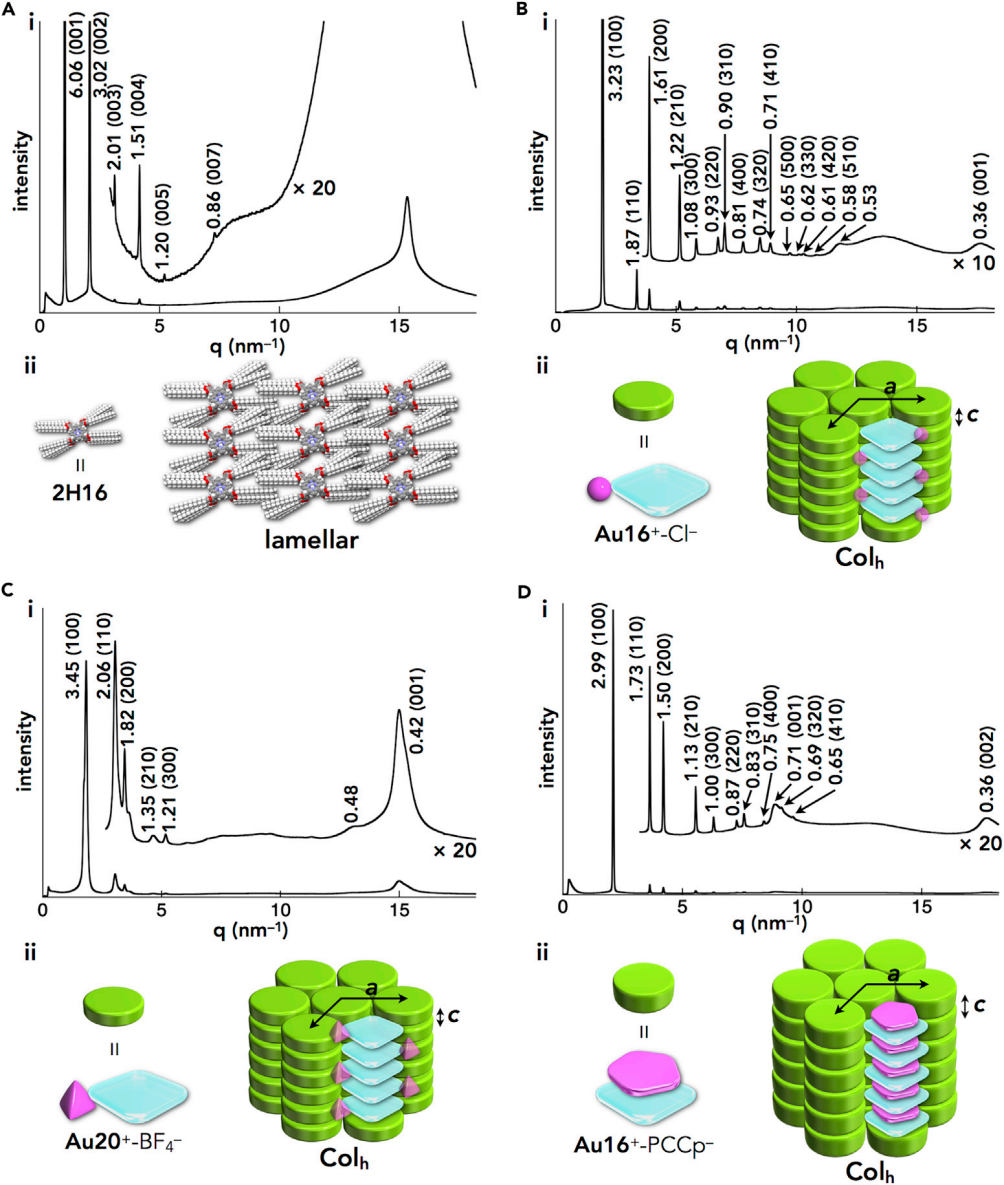
Synchrotron XRD Patterns and Packing Model Structures (A–D) (i) Synchrotron XRD patterns and (ii) packing model structures of (A) **2H16**, (B) **Au16**^+^-Cl^–^, (C) **Au20**^+^-BF_4_^–^, and (D) **Au16**^+^-PCCp^–^ in the mesophases at 22°C, 100°C, 52°C, and 280°C, respectively, upon cooling. Arrangement of the anions in the model structures of (B) and (C) is not exactly determined.

The packing structures of the ion-pairing assemblies in the mesophases were examined by synchrotron XRD analysis (Table 1, Figures S71–S120, and Tables S2–S19). For the metal-free porphyrins, in contrast to the liquid states of **2H8** and **2H12** with shorter alkyl chains (Maruyama et al., 2010, Nowak-Król et al., 2010), the assemblies of **2H16** and **2H20** exhibited lamellar structures with interdigitating alkyl chains (Figure 8A [i, ii]). In contrast, the porphyrin–Au^III^ ion pairs formed characteristic anion-dependent ordered assemblies. The results revealed that **Au*n***^+^-Cl^–^ (*n* = 12, 16, and 20) formed a hexagonal columnar (Col_h_) structure in the mesophase; for example, **Au16**^+^-Cl^–^ showed a Col_h_ structure at 100°C (cooling) with *a* = 3.73, *c* = 0.36 nm, and *Z* = 1 (*ρ* = 1.44) (Figure 8B [i, ii]). The peak at 0.36 nm (001) indicated the ordered π–π stacking distance of porphyrin–Au^III^ cations, which formed a charge-segregated columnar structure. Furthermore, the peak at 0.53 nm was ascribable to the ordered arrangement of counter Cl^–^ or peripheral aryl rings, although their exact positions could not be determined. The fairly high density of the mesophase for **Au16**^+^-Cl^–^ suggested an effective π–π stacking of porphyrin–Au^III^ cations as well as the tightly associated Cl^–^. The *a* values increased according to the alkyl chain lengths of the cations, as observed in **Au*n***^+^-Cl^–^ (*n* = 12 and 20) showing 3.37 and 4.14 nm at 100°C and 62°C (cooling), respectively. The lattice parameters of these Col_h_ structures were consistent with the AM1-optimized structures of the alkyl-substituted porphyrins (Figure S54) (Frisch et al., 2013).

The XRD analysis of **Au16**^+^-BF_4_^–^ at 45°C on heating revealed a Col_h_ structure, with peaks that were much broader than those of **Au16**^+^-Cl^–^, especially for the broad stacking height peak at 0.42 nm, suggesting the formation of a less ordered charge-segregated assembly. The formation of a charge-segregated assembly was also observed for the ion-pairing assembly of **Au20**^+^-BF_4_^–^, which formed a Col_h_ structure at 52°C (cooling) with *a* = 3.98, *c* = 0.42 nm, and *Z* = 1 (*ρ* = 1.30) (Figure 8C [i, ii]). These charge-segregated assembling modes were also observed in the crystal structure of **Au0**^+^-BF_4_^–^ (Figure 5A [ii]). The broad peak at 0.48 nm, which was observed in both **Au*n***^+^-BF_4_^–^ (*n* = 16 and 20), could be due to the arrangement of BF_4_^–^ or aryl rings. Meanwhile, **Au12**^+^-PF_6_^–^ formed an oblique columnar (Col_ob_) structure at 50°C (cooling) with *a* = 4.85, *b* = 4.11, *c* = 0.74 nm, *γ* = 99.3°, and *Z* = 2 (*ρ* = 0.73). In contrast to the charge-segregated ion-pairing assemblies of the Cl^–^ and BF_4_^–^ ion pairs, the broad XRD peak at 0.74 nm in the mesophase of **Au12**^+^-PF_6_^–^ was observed for the stacking of **Au12**^+^, indicating the contribution of a less ordered charge-by-charge assembly. Therefore, Cl^–^, which is a smaller anion (hard anion), is suitable for the formation of a charge-segregated assembly in bulk materials excluding solvents because it can interact with the pyrrole β-H of **Au12**^+^ through hydrogen bonding and thus can be localized around the columnar structures of **Au12**^+^. On the other hand, PF_6_^–^, which is a more bulky anion (soft anion), is less suitable for the formation of charge-segregated assemblies because bulky anions may interfere with the stacking of porphyrin–Au^III^ planes by preferentially interacting with the π-electronic porphyrin–Au^III^ cation (soft cation) plane. According to the hard and soft acids and bases theory, soft anions tend to interact with soft cations such as π-electronic cations. This tendency is also important for the formation of charge-by-charge assemblies with π-electronic anions.

Distinctive columnar assemblies were observed when a planar π-electronic anion was used as the counter species for the porphyrin–Au^III^ cations. In fact, mesophases of **Au*n***^+^-PCCp^–^ (*n* = 16 and 20) exhibited well-defined Col_h_ structures with perfect charge-by-charge assemblies. For example, in the mesophase at 280°C (cooling), **Au16**^+^-PCCp^–^ exhibited a Col_h_ structure with *a* = 3.46, *c* = 0.71 nm, and *Z* = 1 (*ρ* = 0.88) (Figure 8D [i, ii]). The value of 0.71 nm is almost twice that of a π–π stacking distance, indicating the distance between identical π-electronic charged species due to the formation of a charge-by-charge columnar assembly, as observed in the crystal structure of **Au0**^+^-PCCp^–^ (Figure 5C [ii]). The diffraction at 0.36 nm is a (002) peak derived from the stacking height of 0.71 nm as a (001) peak. A highly ordered arrangement of charge-by-charge assembly was also suggested by the observation of (003) and (004) peaks in wide-angle XRD (Figure S116). The speculated density of 0.88 for **Au16**^+^-PCCp^–^, which is smaller than that of **Au16**^+^-Cl^–^ (*ρ* = 1.44), reflects the alternate stacking of porphyrin–Au^III^ and PCCp^–^ at the center of the columns and the resulting less dense packing of the peripheral aliphatic chains. The mesophase of **Au20**^+^-PCCp^–^ also showed a similar Col_h_ structure (*a* = 3.75, *c* = 0.71 nm, and *Z* = 1 [*ρ* = 0.88] at 250°C [cooling]) as **Au16**^+^-PCCp^–^. The exceptionally wide-temperature-range Col_h_ mesophases, which were observed from approximately room temperature (RT) to 300°C, were maintained by the charge-by-charge stacking of genuine π-electronic ions through synergetic π–π and electrostatic interactions.

**Figure 9:**
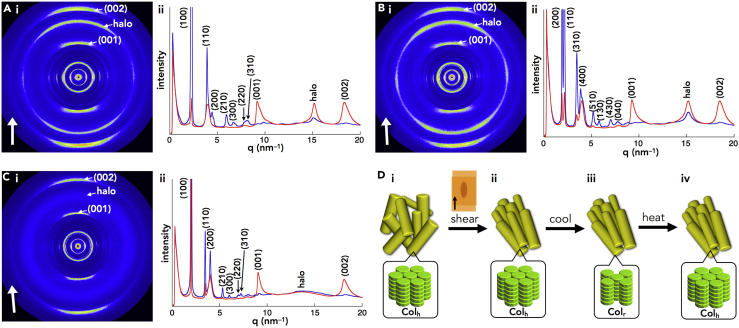
Assembling Behavior of Ion Pairs under Mechanically Sheared Conditions (A–C) (i) Two-dimensional XRD patterns (lower left white arrows indicate the sheared direction) and (ii) XRD patterns extracted from 2D XRD patterns for the meridional (90 ± 20°) (red line) and equatorial (0 ± 20°) (blue line) regions for mechanically sheared ion pairs: (A) **Au16**^+^-PCCp^–^, sheared at 250°C and measured at RT; (B) **Au20**^+^-PCCp^–^, sheared at 230°C and measured at RT; and (C) **Au20**^+^-PCCp^–^, sheared at 230°C, cooled to RT, and measured at 100°C. (D) Orientations of domains (yellow cylinders) (top) and the packing mode inside of the domains (bottom) in **Au20**^+^-PCCp^–^ for (i) the original mesophase (Col_h_), (ii) the sheared sample at 230°C (Col_h_), (iii) after cooling to RT (Col_r_), and (iv) after heating to 100°C (Col_r_).

The XRD analysis of mechanically sheared **Au16**^+^-PCCp^–^ at 250°C revealed that the diffractions in the wider-angle region, including that at 0.68 nm (001), were enhanced in the meridional (sheared) direction, affording an anisotropic XRD pattern at RT (Figure 9A and S124). The enhancement of the peak at 0.68 nm clearly indicates the orientation of charge-by-charge columns with an ordered intercolumnar arrangement along the sheared direction. The enhanced peak for the halo, at an angle of approximately ±20° to meridional (sheared) direction, can be ascribed to the charge-by-charge columnar assembly, which has an arrangement of laterally rotating porphyrin–Au^III^ complexes with a rotating angle of approximately ±20° (Pisula et al., 2007). A similar anisotropic arrangement was observed for sheared **Au20**^+^-PCCp^–^ in the Col_r_ phase (*a* = 6.57, *b* = 3.26, *c* = 0.69 nm, and *Z* = 2 [*ρ* = 1.03]) at RT (Figures 9B and S125). Surprisingly, the anisotropic orientation was maintained even in the (original) higher-temperature Col_h_ phase (Figures 9C and S126). In this case, the Col_h_ mesophase at 230°C (Figure 9D [i]) showed anisotropic orientation after shearing (Figure 9D [ii]). The ion pair exhibited the Col_r_ assembly at RT as a crystalline state (Figure 9D [iii], the state of Figure 9B). Furthermore, the anisotropic orientation was maintained at 100°C in the Col_h_ mesophase after the phase transition from Col_r_ (Figure 9D [iv], the state of Figure 9C). The retention of the domain orientations during thermal processes can be correlated with the contribution of a robust packing state due to the charge-by-charge assemblies (Figure S123) (Grigoriadis et al., 2010, Haase et al., 2011).

The solid-state UV-vis absorption spectra of **Au16**^+^-X^–^ (X^–^ = Cl^–^ and PCCp^–^) and **Au20**^+^-BF_4_^–^ at RT upon cooling showed a λ_max_ at 425 nm with characteristic small bands; the Cl^–^ and BF_4_^–^ ion pairs showed similar peaks at 535/576 (shoulder) and 539/577 nm, respectively, whereas the PCCp^–^ ion pair showed a blue-shifted shoulder peak at 527 nm (Figure S63). The red-shifted shoulder peaks of the Cl^–^ and BF_4_^–^ ion pairs are derived from the stacking of porphyrin–Au^III^ cations in the charge-segregated assembly. The large peak of **Au20**^+^-BF_4_^–^ at 577 nm can be the result of a possible slipped stacking of the porphyrin core unit due to the less ordered arrangement. In contrast, the blue-shifted shoulder of **Au16**^+^-PCCp^–^ is more similar to the Q band of the solution-state monomeric ion pairs as observed at 521 nm. The blue-shifted absorption compared with those for the Cl^–^ and BF_4_^–^ ion pairs can be explained by the distinct alternate stacking of **Au16**^+^ and PCCp^–^, resulting in the monomer-state UV-vis absorption. These results showed that the ion pairs with Cl^–^ and BF_4_^–^ form preferentially charge-segregated assemblies, whereas planar PCCp^–^ forms a charge-by-charge assembly.

### Extended π-Electronic Ion Pair

**Figure 10:**
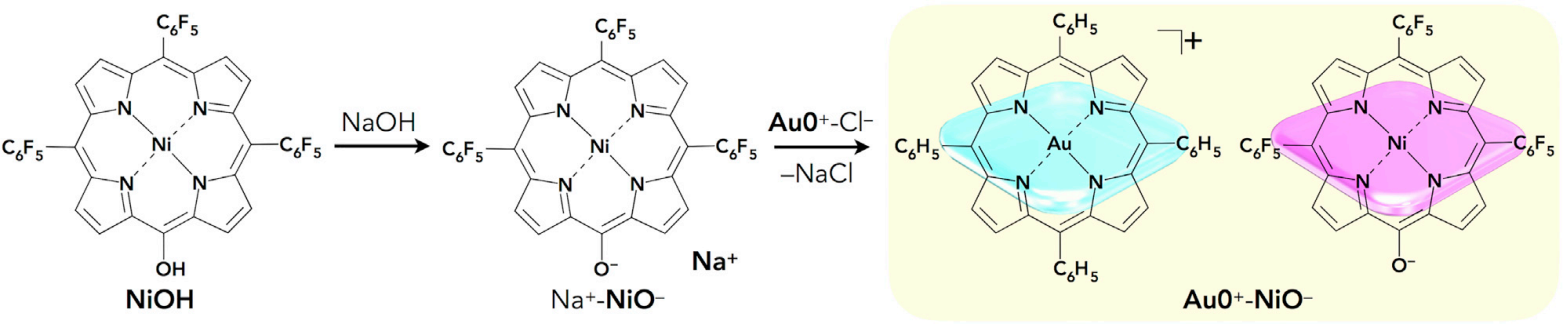
Preparation of Porphyrin Ion Pair Au0^+^-NiO^–^

The preparation protocol of ion pairs is also applicable to the formation of a variety of π-electronic ion pairs. Counteranions of π-electronic porphyrin–Au^III^ cations can be exchanged by the introduction of deprotonated species of extended π-electronic units with appropriate acid moieties such as hydroxy and carboxy units. The negative charge of the deprotonated species can be delocalized on the π-electronic unit for stabilization. Ni^II^ porphyrin **NiOH**, which has a hydroxy unit at one of the *meso* positions (Sasano et al., 2017, Stähler et al., 2017), was used as the precursor for the π-electronic anionic species **NiO**^–^ as the counteranion for the porphyrin–Au^III^ cation (Figure 10) (Stähler et al., 2017). The countercation of **NiO**^–^ was exchanged using the following stepwise preparation protocol: (1) **NiOH** in CH_2_Cl_2_ was treated with an excess amount of aqueous NaOH to yield Na^+^-**NiO**^–^ in a CH_2_Cl_2_ phase and (2) after washing with water to remove NaCl and subsequent recrystallization with EtOAc/*n*-hexane, 1 equiv. of **Au0**^+^-Cl^–^ was added to Na^+^-**NiO**^–^ to form **Au0**^+^-**NiO**^–^. The π-electronic ion pair **Au0**^+^-**NiO**^–^ was obtained as a brown solid with 48% yield. The characterization of the ion pair and the determination of the 1:1 molar ratio of **Au0**^+^ and **NiO**^–^ were conducted by ^1^H NMR and elemental analysis.

**Figure 11:**
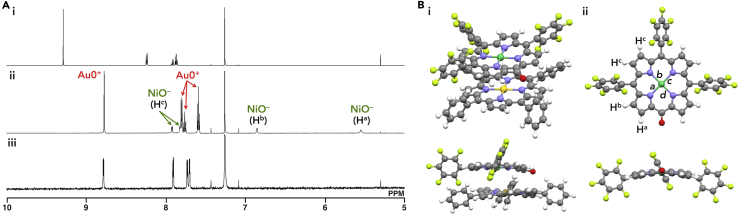
Solution-State Ion-Pairing Behavior of NiO^–^-Based Ion Pairs (A and B) (A) ^1^H NMR spectra of (i) **Au0**^+^-Cl^–^, (ii) **Au0**^+^-**NiO**^–^, and (iii) TBA^+^-**NiO**^–^ (Sasano et al., 2017) in CDCl_3_ (1.0 × 10^−3^ M) at 20°C and (B) (i) side perspective and side views of optimized structure of **Au0**^+^-**NiO**^–^ and (ii) top and side views of **NiO**^–^ in (i). The labels of hydrogens in (A) (ii) correspond to those in (B) (ii). Atom color code in (B): gray, white, blue, red, light green, green, and yellow refer to carbon, hydrogen, nitrogen, oxygen, fluorine, nickel, and gold, respectively.

The ion-pairing formation of **Au0**^+^ and **NiO**^–^ exhibited characteristic ^1^H NMR signal shifts based on the effects of (1) aromatic ring current (π-electrons) and (2) proximally located charges. The ion pair **Au0**^+^-**NiO**^–^ showed sharp ^1^H NMR signals at 8.78, 7.80, 7.76, and 7.59 ppm for **Au0**^+^ and 7.92, 7.82, 6.85, and 5.54 ppm for **NiO**^–^ in CDCl_3_ (1.0 × 10^−3^ M) at 20°C (Figure 11A [ii]); these values are shifted upfield compared with those of **Au0**^+^-Cl^–^ and TBA^+^-**NiO**^–^ (Sasano et al., 2017), with signals at 9.29, 8.23, 7.92, and 7.87 ppm and 8.78, 7.91, 7.73, and 7.70 ppm, respectively (Figure 11A [i and iii]). The upfield-shifted signals of **Au0**^+^-**NiO**^–^ suggested the interaction of these cations and anions in the solution state ([1] the effect of aromatic ring current (π-electrons)). On the other hand, the signals of **Au0**^+^-**NiO**^–^ were broadened and shifted downfield when the concentrations were lowered to 1.0 × 10^−5^ M due to the fast exchange between the ion pair and monomeric **Au0**^+^ and **NiO**^–^. In addition, the signals of **Au0**^+^ and **NiO**^–^ were shifted upfield and downfield, respectively, upon cooling from 50°C to −50°C in CDCl_3_ (1.0 × 10^−3^ M). The results are representative of the shielding effect of electron-rich anionic **NiO**^–^ on **Au0**^+^ and the deshielding effect of electron-poor cationic **Au0**^+^ on **NiO**^–^ in the tightly bound ion pair ([2] the effect of proximally located charges).

The geometry-optimized structure of **Au0**^+^-**NiO**^–^, at B3LYP-GD3BJ level with the 6-31G(d,p) basis set for C, H, N, O, F, and Ni and LanL2DZ for Au (calculated starting from the crystal structure as described below), showed a stacking structure of oppositely charged porphyrin π-planes (Figures 11B and S59) (Frisch et al., 2013). The Ni–N distances in the optimized structure of **Au0**^+^-**NiO**^–^ were 1.964 (*a*), 1.963 (*b*), 1.962 (*c*), and 1.968 (*d*) Å (Figure 11B [ii]), and the mean-plane deviation of the 25-atom plane was 0.17 Å. According to this optimized stacking structure of **Au0**^+^-**NiO**^–^, the ^1^H NMR signals of **NiO**^–^ at 7.92 and 7.82 ppm can be assigned to H^c^ and those at 6.85 and 5.54 ppm can be assigned to H^b^ and H^a^, respectively, due to the shielding effect of current ring of **Au0**^+^ (Figure 11A [ii]). These assignments were also supported by ^1^H–^1^H COSY (correlation spectroscopy) and ^1^H–^13^C HMBC (heteronuclear multiple bond coherence). ^1^H–^1^H COSY in CDCl_3_ showed the correlation between signals at 6.85 and 5.54 ppm, suggesting that the corresponding protons were located at the vicinal positions. ^1^H–^13^C HMBC in C_6_D_6_ (1.0 × 10^−2^ M at 20°C) showed the correlation between the ^1^H NMR signals of 8.00 and 7.91 ppm (comparable to the ^1^H NMR signals at 7.92 and 7.82 ppm in CDCl_3_) and the ^13^C NMR signal at 147.20 ppm, which can be assigned to the C_6_F_5_-attached 15-position carbon of **NiO**^–^. The Ni–N distances in **NiO**^–^ of the optimized TBA^+^-**NiO**^–^ structure at the B3LYP-GD3BJ/6-31G(d,p) level, calculated starting from the crystal structure (Sasano et al., 2017), were 1.942, 1.941, 1.941, and 1.943 Å and the mean-plane deviation of the 25-atom plane was 0.41 Å. The longer Ni–N distance and planar structure of **NiO**^–^ in **Au0**^+^-**NiO**^–^ compared with those in TBA^+^-**NiO**^–^ may be attributed to the contribution of the coordination of **Au0**^+^ as a π-ligand to **NiO**^–^. These characteristic behaviors of π-electronic ion pairs in a solution state can be derived from the favorable interactions between π-electronic cations and anions. The ESP, calculated at B3LYP-GD3BJ/6-31+G(d,p) level with LanL2DZ for Au based on the optimized structure, revealed the delocalized negative and positive charges in **NiO**^–^ and **Au0**^+^, respectively, effective for stacking (Figure S60). In addition, the UV-vis absorption spectrum in CH_2_Cl_2_ (4.6 × 10^−4^ M) of **Au0**^+^-**NiO**^–^, mainly existed as an ion pair, corresponding to the sum of the independent absorption bands of each π-electronic ion, suggesting that the electronic interaction between the π-electronic cations and anions is weak under these conditions; this is also supported by the independent electron spin densities for each π-electronic ion in the MO of the ion pair (Figure S62).

**Figure 12:**
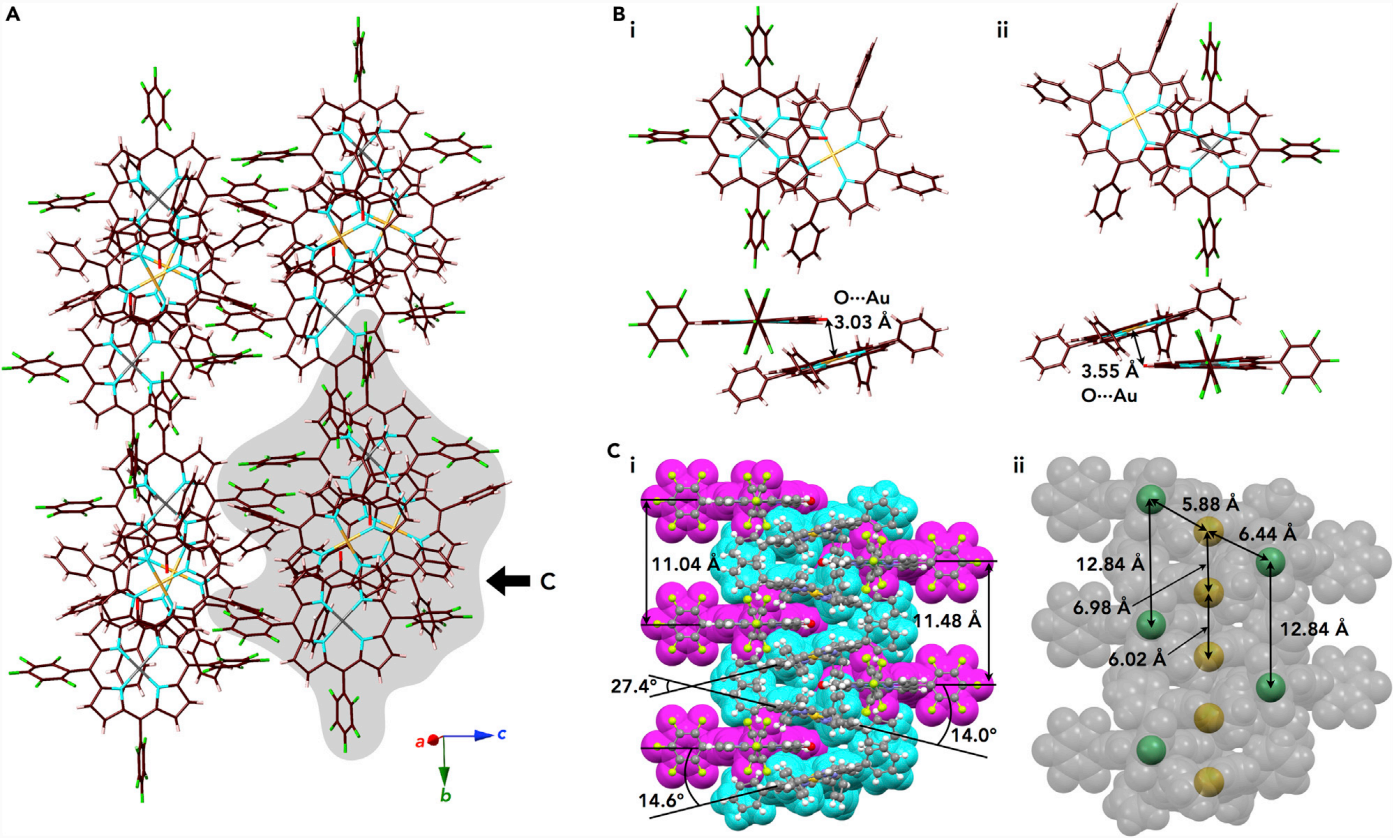
Single-Crystal X-Ray Structure of Au0^+^-NiO^–^ (A–C) (A) Representative packing mode as a top view, (B) top and side views of enlarged ion pairs with independent **NiO**^–^ (i,ii), and (C) (i) space-filling packing model as a side view from the direction indicated by the arrow in (A) and (ii) that highlighting the arrangement of metal ions. Solvent molecules are omitted for clarity. Atom color code in (A) and (B): brown, pink, light blue, light green, gray, and light orange refer to carbon, hydrogen, nitrogen, fluorine, nickel, and gold, respectively. Color code in (C) (i): cyan and magenta represent cations and anions, respectively. Color code in (C) (ii): green and yellow refer to nickel and gold, respectively.

The solid-state ion-pairing assembly of **Au0**^+^-**NiO**^–^ was revealed by the X-ray analysis of a single crystal prepared by vapor diffusion of EtOAc/*n*-octane (Figure 12A, S46, and S47). In the crystal, **Au0**^+^ and **NiO**^–^ were alternately stacked in a charge-by-charge columnar assembly with (C–)O⋅⋅⋅Au distances of 3.03 and 3.55 Å, and the dihedral angles between the mean planes of **Au0**^+^ and **NiO**^–^ (core 25 atoms) were 14.0° and 14.6° (Figures 12B, 12C [i], and S53). Considering the distances, the anionic oxygen of **NiO**^–^ had no coordination to the Au^III^ site with the proximal location by electrostatic interaction, as also supported by the O–Au–**Au0**^+^-plane angles of 71.5° and 55.9°. The parallel arrangement of **NiO**^–^ units showed distances of 11.04 and 11.48 Å, whereas **Au0**^+^ units are not parallel and have a dihedral angle of 27.4°. In addition, the different metal ions, Au^3+^ and Ni^2+^, were arranged in a zigzag fashion with Au⋅⋅⋅Ni distances of 5.88 and 6.44 Å and Au–Ni–Au angles of 61.7° and 65.6°. The distances for the identical metal ions Au^3+^ and Ni^2+^ in the columnar direction are 6.02/6.98 and 12.84 Å, respectively; the value of 12.84 Å is consistent with the lattice parameter *a*. The spatial arrangement of heterometals (Figure 12C [ii]) was achieved by the formation of ion-pairing assemblies comprising extended π-electronic ions such as appropriately designed positively and negatively charged porphyrin–metal complexes.

### Conclusions

Diverse ion pairs were prepared based on porphyrin–Au^III^ complexes as stable π-electronic cations. Porphyrin–Au^III^-based ion pairs formed charge-by-charge and charge-segregated assemblies in single crystals, according to the geometry of the anionic species. Porphyrin–Au^III^-based ion pairs substituted with aliphatic chains afforded anion-dependent mesophases. In particular, the ion pairs with a π-electronic anion clearly afforded charge-by-charge-based mesophases, which existed at exceptionally wide temperature ranges, in contrast to the assemblies with relatively small anions. Furthermore, the ion pair with a negatively charged porphyrin–metal complex has the potential to be used in ion-pairing strategies for functional electronic materials. Ion-pairing assemblies comprising genuine π-electronic ions are stabilized by synergetic π–π stacking and electrostatic interactions, showing that this methodology has a great advantage for the fabrication of a variety of nanostructured materials with fine-tuned electronic states. Further modifications at the peripheries of π-electronic systems such as porphyrins will enable the preparation of various fascinating π-electronic ion pairs and their associated functional ion-pairing assemblies and materials, exhibiting ferroelectric or electric conductive properties.

## Methods

All methods can be found in the accompanying Transparent Methods supplemental file.

### Limitation of the Study

In our study, a variety of π-electronic ion pairs comprising porphyrin–Au^III^ complexes and their assemblies were prepared as crystals, supramolecular gels, and liquid crystals. Ion-pairing assemblies based on π-electronic ions exhibited organized states with the contributions of charge-by-charge and charge-segregated assemblies, depending on the geometries and electronic states of the counteranions. However, at present, the complete control of the formation of charge-by-charge and charge-segregated assemblies have not been fully investigated particularly focusing on the appropriate combination of constituent ions. More systematic and detailed investigations on the relationships between the ion-pairing combination and their assembling modes are required for the further development of fascinating ferroelectric materials and electric conductive materials.

## Supplemental Information

Document S1. Transparent Methods, Figures S1–S126, and Tables S1–S20
