# Supplementary material for: Liquid Crystals Comprising π-Electronic Ions from Porphyrin–AuIII Complexes
Source: iScience. 2019 Apr 1;14:241–56. doi: 10.1016/j.isci.2019.03.027 (PMC6463747; doi:10.1016/j.isci.2019.03.027)
Supplement: Document S1. Transparent Methods, Figures S1–S126, and Tables S1–S20 [file mmc1.pdf]

ISCI, Volume *14*

Supplemental Information

Liquid Crystals Comprising π-Electronic Ions from Porphyrin–Au^{III} Complexes

Yohei Haketa, Yuya Bando, Yoshifumi Sasano, Hiroki Tanaka, Nobuhiro Yasuda, Ichiro Hisaki, and Hiromitsu Maeda

Table of Contents

1. Transparent Methods S2
1-1. Synthetic procedures and spectroscopic data S2
Figure S1 Synthesis and preparations of ion pairs comprising Au^{III} complexes. S2
Figure S2,3 TLC analysis. S13
Figure S4–39 ^{1}H, ^{13}C, and ^{19}F NMR spectra. S14
Figure S40 UV/vis absorption spectra. S49
1-2. X-ray crystallographic data S50
Figure S41–47 Ortep drawings of single-crystal X-ray structures. S52
Figure S48–53 Packing diagrams. S57
1-3. Theoretical studies S63
Figure S54–60 Optimized structures and electron density diagrams. S63
Figure S61,62 Molecular orbitals of stacking ion pairs. S67
Cartesian coordination of optimized structures. S70
1-4. Examination of organized structures S116
Figure S63 Solid-state UV/vis absorption spectra. S116
Figure S64 Photograph of gel. S116
Figure S65 OM and AFM of fibrous morphologies. S117
Figure S66 VT UV/vis absorption spectra of ion pairs. S117
Figure S67,68 DSC thermographs. S118
Figure S69 POM images. S120
Figure S70 Summary for phase transitions. S122
Figure S71–120 XRD and packing diagrams. S122
Figure S123 Summary for the XRD of sheared samples. S180
Figure S124–126 XRD of sheared samples. S181
2. Supplemental references S184

1. Transparent Methods

1-1. Synthetic procedures and spectroscopic data

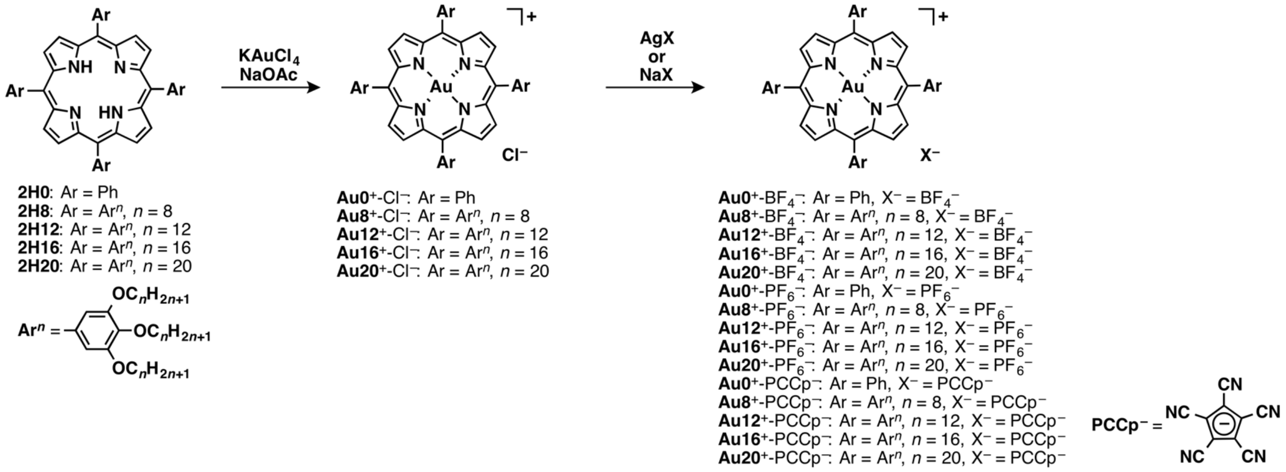

Figure S1 Synthesis and preparations of ion pairs comprising Au^{III} complexes of *meso*-aryl-substituted porphyrins with various anions, Related to Figure 2.

General Procedures. Starting materials were purchased from FUJIFILM Wako Pure Chemical Corp., Nacalai Tesque Inc., and Sigma-Aldrich Co. and used without further purification unless otherwise stated. **2H8** (Maruyama et al., 2010), **2H12** (Maruyama et al., 2010; Nowak-Król et al., 2010), and **Au0**$^+$-Cl$^-$ (Che et al., 2003) were prepared according to the published procedures. NMR spectra used in the characterization of products were recorded on a JEOL ECA-600 600 MHz spectrometer and a Bruker AVANCE III 600 MHz spectrometer, with the help of Dr. Ryohei Yamakado, Yamagata University, and Dr. Ryosuke Miyake, Ochanomizu University. ^{1}H and ^{13}C NMR spectra were referenced to solvent and ^{19}F NMR spectra were referenced to C_6F_6. UV-visible absorption spectra were recorded on a Hitachi U-3500 spectrometer. Matrix-assisted laser desorption ionization time-of-flight mass spectrometries (MALDI-TOF-MS) were recorded on a Shimadzu Axima-CFRplus with TCNQ matrix. Elemental analyses were performed on a Yanaco CHN Corders (MT-3, MT-5, and MT-6) and JSL JM-10 for carbon, hydrogen, and nitrogen and on Mitsubishi Chemical Analytech AQF-100 and Dionex ICS-1500 instruments for fluorine and chlorine, at the Laboratory for Organic Elemental Microanalysis, Kyoto University. TLC analyses were carried out on aluminum sheets coated with silica gel 60 (Merck 5554). Column chromatography was performed on Sumitomo alumina KCG-1525 and Wakogel C-300.

5,10,15,20-Tetrakis(3,4,5-trihexadecyloxyphenyl)porphyrin, 2H16. According to the literature procedures (Nowak-Król et al., 2010), a solution of pyrrole (260 mg, 3.88 mmol) and 3,4,5-trihexadecyloxybenzaldehyde (Nowak-Król et al., 2010) (2.50 g, 3.02 mmol) in CH_2Cl_2 (300 mL) was stirred under N_2 with shielding from light. Trifluoroacetic acid (TFA) (0.37 mL, 4.80 mmol) and $BF_3{\cdot}OEt_2$ (11.4 μL, 90.6 μmol) was added, and the solution was stirred for 2.5 h at r.t. 2,3-Dichloro-5,6-dicyano-1,4-benzoquinone (DDQ) (530 mg, 2.33 mmol) was added to the solution, and the resulting solution was stirred for an additional 2 h. After the reaction mixture was neutralized by triethylamine, concentrated under reduced pressure, and passed over an alumina column, the solvent was removed. The residue was then chromatographed over a silica gel column (Wakogel C-300, eluent: 50% CH_2Cl_2/*n*-hexane) and recrystallized from CH_2Cl_2/MeOH to give **2H16** (790 mg, 0.23 mmol, 30%) as a purple solid. R_f = 0.45 (50% CH_2Cl_2/*n*-hexane). ^{1}H NMR (600 MHz, $CDCl_3$, 20 °C): δ(ppm): 8.94 (s, 8H, β-H), 7.41 (s, 8H, Ar-H), 4.29 (t, J = 6.6 Hz, 8H, OCH_2), 4.07 (t, J = 6.6 Hz, 16H, OCH_2), 1.97 (quin, J = 7.2 Hz, 8H, $OCH_2\underline{CH_2}$), 1.86 (quin, J = 7.2 Hz, 16H, $OCH_2\underline{CH_2}$), 1.67 (quin, J = 7.8 Hz, 8H, $O(CH_2)_2\underline{CH_2}$), 1.51–1.21 (m, 304H, $O(CH_2)_2\underline{CH_2}$ + $O(CH_2)_3\underline{(CH_2)_{12}}$), 0.88 (t, J = 7.2 Hz, 12H, $O(CH_2)_{15}\underline{CH_3}$), 0.86 (t, J = 7.2 Hz, 24H, $O(CH_2)_{15}\underline{CH_3}$), –2.81 (s, 2H, NH). ^{13}C NMR (151 MHz, $CDCl_3$, 20 °C): δ(ppm) 151.36, 138.11, 137.25, 120.34, 114.44, 73.92, 69.52, 32.10, 32.06, 30.79, 30.05, 30.01, 29.94, 29.85, 29.80, 29.70, 29.64, 29.55, 29.50, 26.50, 26.33, 22.86, 22.83, 14.28, 14.25 (some of the signals for aryl units and hexadecyl chains were overlapped). UV/vis (CH_2Cl_2, λ_{max}[nm] (ε, 10^5 $M^{-1}cm^{-1}$)): 426 (5.2), 519 (2.3), 555 (1.1), 594 (0.70), 649 (0.55). MALDI-TOF-MS: *m/z* (% intensity): 3499.2 (100). Calcd for $C_{236}H_{414}N_4O_{12}$ ($[M]^+$): 3499.20.

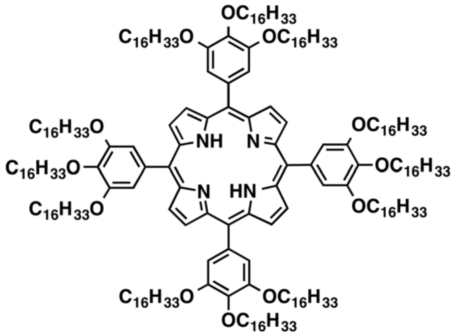

5,10,15,20-Tetrakis(3,4,5-triicosyloxypheny)porphyrin, 2H20. According to the literature procedures (Nowak-Król et al., 2010), a solution of pyrrole (270 mg, 4.02 mmol) and 3,4,5-triicosyloxybenzaldehyde (Nowak-Król et al., 2010) (3.15 g, 3.16 mmol) in CH_2Cl_2 (320 mL) was stirred under N_2 with shielding from light. TFA (0.39 mL, 5.0 mmol) and $BF_3 \cdot OEt_2$ (12.0 µL, 94.9 µmol) was added, and the solution was stirred for 2.5 h at r.t. DDQ (550 mg, 2.42 mmol) was added to the solution, and the resulting solution was stirred for an additional 2 h. After the reaction mixture was neutralized by triethylamine, concentrated under reduced pressure, and passed over an alumina column, the solvent was removed. The residue was then chromatographed over a silica gel column (Wakogel C-300, eluent: 50% $CHCl_3$/*n*-hexane) and recrystallized from CH_2Cl_2/MeOH to give **2H20** (820 mg, 0.20 mmol, 25%) as a purple solid. R_f = 0.73 (50% CH_2Cl_2/*n*-hexane). ^{1}H NMR (600 MHz, $CDCl_3$, 20 °C): δ(ppm): 8.94 (s, 8H, β-H), 7.41 (s, 8H, Ar-H), 4.29 (t, J = 6.6 Hz, 8H, OCH_2), 4.07 (t, J = 6.6 Hz, 16H, OCH_2), 1.97 (quin, J = 7.2 Hz, 8H, $OCH_2\underline{CH_2}$), 1.86 (quin, J = 7.2 Hz, 16H, $OCH_2\underline{CH_2}$), 1.67 (quin, J = 7.8 Hz, 8H, $O(CH_2)_2\underline{CH_2}$), 1.51–1.21 (m, 400H, $O(CH_2)_2\underline{CH_2}$ + $O(CH_2)_3\underline{(CH_2)_{16}}$), 0.88 (t, J = 7.2 Hz, 12H, $O(CH_2)_{19}\underline{CH_3}$), 0.87 (t, J = 7.2 Hz, 24H, $O(CH_2)_{19}\underline{CH_3}$), –2.81 (s, 2H, NH). ^{13}C NMR (151 MHz, $CDCl_3$, 20 °C): δ(ppm) 151.36, 138.09, 137.25, 120.33, 114.43, 73.91, 69.51, 32.09, 30.79, 30.05, 30.02, 29.98, 29.93, 29.88, 29.86, 29.81, 29.70, 29.64, 29.51, 26.50, 26.34, 22.84, 14.25 (some of the signals for aryl units and icosyl chains were overlapped). UV/vis (CH_2Cl_2, λ_{max}[nm] (ε, 10^5 $M^{-1}cm^{-1}$)): 426 (4.6), 519 (2.0), 556 (0.93), 593 (0.60), 648 (0.45). MALDI-TOF-MS: *m/z* (% intensity): 4172.9 (100). Calcd for $C_{284}H_{510}N_4O_{12}$ ($[M]^+$): 4172.95.

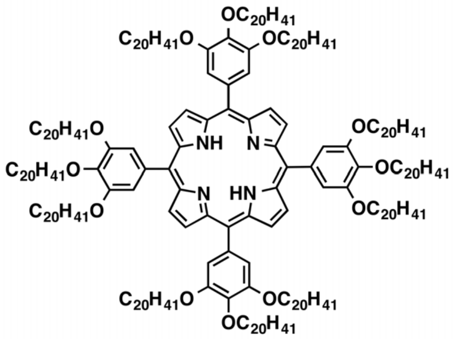

Au^{III} complex of 2H8 as a Cl$^–$ salt, Au8$^+$-Cl$^–$. A solution of $KAuCl_4$ (190 mg, 0.50 mmol) and NaOAc (206 mg, 2.52 mmol) in acetic acid (19 mL) were heated at 80 °C for 15 min. A solution of **2H8** (867 mg, 0.40 mmol) in $(CH_2Cl)_2$ (9.9 mL) was added dropwise. The mixture was heated under reflux for 2 h. Upon removal of solvent by vacuum, the residue was dissolved in CH_2Cl_2. The CH_2Cl_2 solution was washed with water and brine and evaporated to dryness. The residue was then chromatographed over ion-exchanged resin (Amberlite IRA402BL, eluent: CH_2Cl_2) and a silica gel column (Wakogel C-300, eluent: 5% MeOH/CH_2Cl_2) and recrystallized from CH_2Cl_2/MeOH to give **Au8**$^+$-Cl$^–$ (100 mg, 0.042 mmol, 10.5%) as a red solid. R_f = 0.27 (5% MeOH/CH_2Cl_2). ^{1}H NMR (600 MHz, $CDCl_3$, 20 °C): δ(ppm): 9.34 (s, 8H, β-H), 7.44 (s, 8H, Ar-H), 4.31 (t, J = 6.6 Hz, 8H, OCH_2), 4.07 (t, J = 6.6 Hz, 16H, OCH_2), 1.97 (quin, J = 7.8 Hz, 8H, $OCH_2\underline{CH_2}$), 1.86 (quin, J = 7.2 Hz, 16H, $OCH_2\underline{CH_2}$), 1.67 (quin, J = 7.8 Hz, 8H, $O(CH_2)_2\underline{CH_2}$), 1.51–1.21 (m, 112H, $O(CH_2)_2\underline{CH_2}$ + $O(CH_2)_3\underline{(CH_2)_4}$), 0.90 (t, J = 7.2 Hz, 12H, $O(CH_2)_7\underline{CH_3}$), 0.84 (t, J = 7.2 Hz, 24H, $O(CH_2)_7\underline{CH_3}$). ^{13}C NMR (151 MHz, $CDCl_3$, 20 °C): δ(ppm) 152.07, 139.04, 137.15, 133.66, 132.22, 123.72, 114.05, 74.00, 69.72, 32.13, 31.92, 30.73, 29.81, 29.63, 29.58, 29.52, 29.39, 26.42, 26.26, 22.91, 22.78, 14.31, 14.21 (some of the signals for octyl chains were overlapped). UV/vis (CH_2Cl_2, λ_{max}[nm] (ε, 10^5 $M^{-1}cm^{-1}$)): 429 (1.3), 530

(0.25). MALDI-TOF-MS: *m/z* (% intensity): 2347.5 (100). Calcd for $C_{140}H_{220}AuN_4O_{12}$ ($[M - Cl]^+$): 2347.64. Elemental analysis: C 69.20, H 9.39, Cl 1.45, N 2.05. Calcd (%) for $C_{140}H_{220}AuClN_4O_{12}\cdot 2H_2O$: C 69.49, H 9.33, Au 8.14, Cl 1.47, N 2.32, O 9.26.

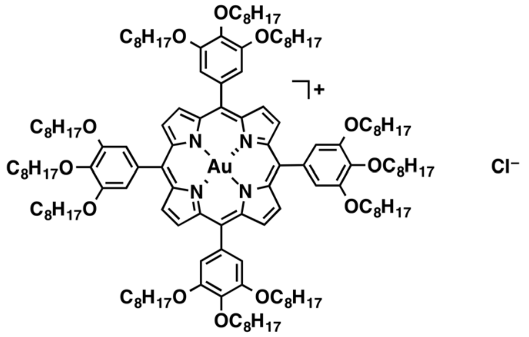

Au^{III} complex of 2H12 as a Cl$^-$ salt, Au12$^+$-Cl$^-$. A solution of $KAuCl_4$ (150 mg, 0.41 mmol) and NaOAc (170 mg, 2.04 mmol) in acetic acid (16 mL) were heated at 80 °C for 15 min. A solution of **2H12** (920 mg, 0.33 mmol) in $(CH_2Cl)_2$ (16 mL) was added dropwise. The mixture was heated under reflux for 2 h. Upon removal of solvent by vacuum, the residue was dissolved in CH_2Cl_2. The CH_2Cl_2 solution was washed with water and brine and evaporated to dryness. The residue was then chromatographed over a silica gel column (Wakogel C-300, eluent: 5% $MeOH/CH_2Cl_2$) and recrystallized from $CH_2Cl_2/MeOH$ to give **Au12**$^+$-Cl$^-$ (320 mg, 0.10 mmol, 32%) as a red solid. R_f = 0.16 (5% $MeOH/CH_2Cl_2$). ^1H NMR (600 MHz, $CDCl_3$, 20 °C): δ(ppm): 9.33 (s, 8H, β-H), 7.43 (s, 8H, Ar-H), 4.31 (t, *J* = 6.6 Hz, 8H, OCH_2), 4.07 (t, *J* = 6.6 Hz, 16H, OCH_2), 1.97 (quin, *J* = 7.8 Hz, 8H, $OCH_2\underline{CH_2}$), 1.86 (quin, *J* = 7.8 Hz, 16H, $OCH_2\underline{CH_2}$), 1.67 (quin, *J* = 7.8 Hz, 8H, $O(CH_2)_2\underline{CH_2}$), 1.51–1.22 (m, 208H, $O(CH_2)_2\underline{CH_2}$ + $O(CH_2)_3\underline{(CH_2)_8}$), 0.90 (t, *J* = 7.2 Hz, 12H, $O(CH_2)_{11}\underline{CH_3}$), 0.84 (t, *J* = 7.2 Hz, 24H, $O(CH_2)_{11}\underline{CH_3}$). ^{13}C NMR (151 MHz, $CDCl_3$, 20 °C): δ(ppm) 152.13, 139.11, 137.21, 133.74, 132.25, 123.76, 114.11, 74.05, 69.78, 32.16, 32.08, 30.80, 30.06, 29.94, 29.87, 29.81, 29.64, 29.62, 29.52, 26.50, 26.35, 22.91, 22.84, 14.32, 14.27 (some of the signals for dodecyl chains were overlapped). UV/vis (CH_2Cl_2, λ_{max}[nm] (ε, 10^5 $M^{-1}cm^{-1}$)): 429 (1.2), 530 (0.22). MALDI-TOF-MS: *m/z* (% intensity): 3021.4 (100). Calcd for $C_{188}H_{316}AuN_4O_{12}$ ($[M - Cl]^+$): 3021.40. Elemental analysis: C 73.85, H 10.66, Cl 1.15, N 1.76. Calcd (%) for $C_{188}H_{316}AuClN_4O_{12}$: C 73.86, H 10.42, Au 6.44, Cl 1.16, N 1.83, O 6.28.

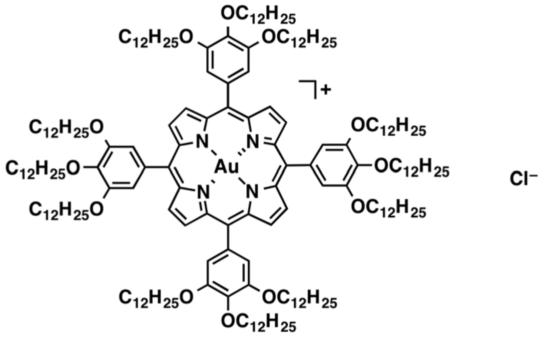

Au^{III} complex of 2H16 as a Cl$^-$ salt, Au16$^+$-Cl$^-$. A solution of $KAuCl_4$ (54.0 mg, 0.14 mmol) and NaOAc (58.6 mg, 0.71 mmol) in acetic acid (5.6 mL) were heated at 80 °C for 15 min. A solution of **2H16** (400 mg, 0.11 mmol) in $(CH_2Cl)_2$ (5.6 mL) was added dropwise. The mixture was heated under reflux for 2 h. Upon removal of solvent by vacuum, the residue was dissolved in CH_2Cl_2. The CH_2Cl_2 solution was washed with water and brine and evaporated to dryness. The residue was then chromatographed over a silica gel column (Wakogel C-300, eluent: 5% $MeOH/CH_2Cl_2$) and recrystallized from $CH_2Cl_2/MeOH$ to give **Au16**$^+$-Cl$^-$ (120 mg, 0.031 mmol, 27%) as a red solid. R_f = 0.18 (5% $MeOH/CH_2Cl_2$). ^1H NMR (600 MHz, $CDCl_3$, 20 °C): δ(ppm): 9.33 (s, 8H, β-H), 7.43 (s, 8H, Ar-H), 4.30 (t, *J* = 6.6 Hz, 8H, OCH_2), 4.07 (t, *J* = 6.6 Hz, 16H, OCH_2), 1.97 (quin, *J* = 6.6 Hz, 8H, $OCH_2\underline{CH_2}$), 1.86 (quin, *J* = 7.2 Hz, 16H, $OCH_2\underline{CH_2}$), 1.67 (quin, *J* = 7.8 Hz, 8H, $O(CH_2)_2\underline{CH_2}$), 1.51–1.21 (m, 304H, $O(CH_2)_3\underline{(CH_2)_{12}}$), 0.88 (t, *J* = 6.6 Hz, 12H, $O(CH_2)_{15}\underline{CH_3}$), 0.86 (t, *J* = 7.2 Hz, 24H, $O(CH_2)_{15}\underline{CH_3}$). ^{13}C NMR (151 MHz, $CDCl_3$, 20 °C): δ (ppm) 152.10, 139.10, 137.18, 133.74, 132.21, 114.12, 74.02, 69.78, 32.10, 32.06, 30.79, 30.04, 30.01, 29.97, 29.95, 29.81, 29.86, 29.80, 29.64, 29.55, 29.50, 26.48, 26.33, 22.86, 22.83, 14.28, 14.25 (some of the signals for hexadecyl chains were overlapped). UV/vis

(CH_2Cl_2, λ_{max}[nm] (ε, 10^5 $M^{-1}cm^{-1}$)): 429 (1.2), 530 (0.22). MALDI-TOF-MS: *m/z* (% intensity): 3694.3 (100). Calcd for $C_{236}H_{412}AuN_4O_{12}$ ([M – Cl]$^+$): 3694.15. Elemental analysis: C 75.73, H 11.37, Cl 0.96, N 1.41. Calcd (%) for $C_{236}H_{412}AuClN_4O_{12}$: C 75.99, H 11.13, Au 5.28, Cl 0.95, N 1.50, O 5.15.

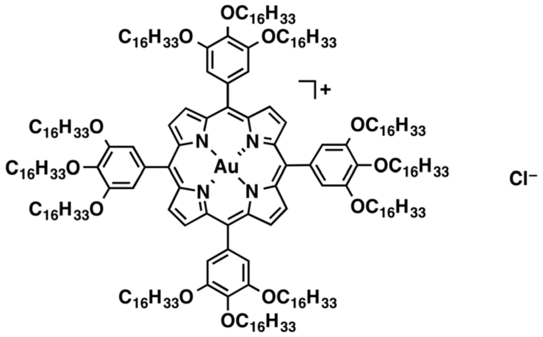

Au^{III} complex of 2H20 as a Cl$^-$ salt, Au20$^+$-Cl$^-$. A solution of $KAuCl_4$ (56.7 mg, 0.15 mmol) and NaOAc (61.4 mg, 0.75 mmol) in acetic acid (5.8 mL) were heated at 80 °C for 15 min. A solution of **2H20** (500 mg, 0.12 mmol) in $(CH_2Cl)_2$ (6 mL) was added dropwise. The mixture was heated under reflux for 2 h. Upon removal of solvent by vacuum, the residue was dissolved in CH_2Cl_2. The CH_2Cl_2 solution was washed with water and brine and evaporated to dryness. The residue was then chromatographed over ion-exchanged resin (Amberlite IRA402BL, eluent: CH_2Cl_2) and a silica gel column (Wakogel C-300, eluent: 5% MeOH/CH_2Cl_2) and recrystallized from CH_2Cl_2/MeOH to give **Au20**$^+$-Cl$^-$ (150 mg, 0.034 mmol, 29%) as a red solid. R_f = 0.21 (5% MeOH/CH_2Cl_2). ^{1}H NMR (600 MHz, $CDCl_3$, 20 °C): δ(ppm): 9.33 (s, 8H, β-H), 7.43 (s, 8H, Ar-H), 4.30 (t, *J* = 6.6 Hz, 8H, OCH_2), 4.07 (t, *J* = 6.6 Hz, 16H, OCH_2), 1.97 (quin, *J* = 7.8 Hz, 8H, $OCH_2\underline{CH_2}$), 1.86 (quin, *J* = 7.8 Hz, 16H, $OCH_2\underline{CH_2}$), 1.67 (quin, *J* = 7.8 Hz, 8H, $O(CH_2)_2\underline{CH_2}$), 1.51–1.21 (m, 400H, $O(CH_2)_2\underline{CH_2}$ + $O(CH_2)_3\underline{(CH_2)_{16}}$), 0.88 (t, *J* = 6.6 Hz, 12H, $O(CH_2)_{19}\underline{CH_3}$), 0.86 (t, *J* = 7.2 Hz, 24H, $O(CH_2)_{19}\underline{CH_3}$). ^{13}C NMR (151 MHz, $CDCl_3$, 20 °C): δ(ppm) 152.06, 139.04, 137.16, 133.73, 132.18, 123.67, 114.06, 73.99, 69.73, 32.07, 32.05, 30.76, 30.03, 30.00, 29.97, 29.95, 29.93, 29.91, 29.88, 29.84, 29.81, 29.79, 29.62, 29.52, 29.50, 26.46, 26.32, 22.83, 22.81, 14.24 (some of the signals for icosyl chains were overlapped). UV/vis (CH_2Cl_2, λ_{max}[nm] (ε, 10^5 $M^{-1}cm^{-1}$)): 429 (0.85), 530 (0.16). MALDI-TOF-MS: *m/z* (% intensity): 4367.8 (100). Calcd for $C_{284}H_{508}AuN_4O_{12}$ ([M – Cl]$^+$): 4367.90. Elemental analysis: C 76.69, H 11.86, Cl 0.79, N 1.13. Calcd (%) for $C_{284}H_{508}AuClN_4O_{12}\cdot1.5H_2O$: C 76.99, H 11.63, Au 4.45, Cl 0.80, N 1.26, O 4.87.

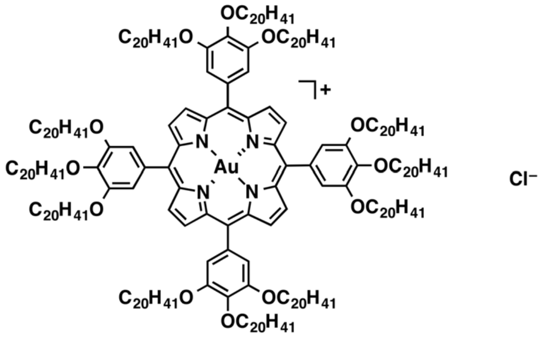

General preparation protocol for anion exchanges from Cl$^-$ to other anions. A solution of Ag$^+$ or Na$^+$ salt of anions (3 equiv) in CH_3CN was added to a solution of Cl$^-$ salts of porphyrin–Au^{III} complexes in CH_3CN or CH_3CN/CH_2Cl_2 and stirred for a few minutes. The resulting precipitates were collected and washed with CH_3CN and water. After confirming that no precipitate formed, the products were filtered and the solvent was evaporated. The residue was then chromatographed over a silica gel column and recrystallized from suitable solvents afforded ion pairs as solid materials. The obtained ion pairs were characterized by ^{1}H, ^{13}C, and ^{19}F NMR and elemental analysis. The details for each ion pair are described as below.

Au^{III} complex of 2H0 as a BF_4^- salt, Au0$^+$-BF_4^-. $AgBF_4$ was used for anion exchange. After the workup, the residue was purified by chromatography over a silica gel column (Wakogel C-300, eluent: 5% MeOH/CH_2Cl_2) and recrystallized from CH_2Cl_2/*n*-hexane to give **Au0**$^+$-BF_4^- (22.3 mg, 0.025 mmol, 70%) as a red solid. R_f = 0.29 (5% MeOH/CH_2Cl_2). ^{1}H NMR (600 MHz, $CDCl_3$, 20 °C): δ(ppm): 9.28 (s, 8H, β-H), 8.26–

8.25 (m, 8H, Ph), 7.92–7.84 (m, 12H, Ph). ^{13}C NMR (151 MHz, $CDCl_3$, 20 °C): δ(ppm) 138.81, 137.14, 134.39, 132.39, 129.57, 127.85, 123.75. ^{19}F NMR (564 MHz, $CDCl_3$, 20 °C): δ(ppm) –159.21 (s, $^{10}BF_4^-$), –159.26 (s, $^{11}BF_4^-$). UV/vis (CH_2Cl_2, λ_{max}[nm] (ε, 10^5 $M^{-1}cm^{-1}$)): 409 (3.9), 521 (1.8). Elemental analysis: C 57.24, H 3.22, N 6.04. Calcd (%) for $C_{44}H_{28}AuBF_4N_4{\cdot}0.4CH_2Cl_2$: C 57.31, H 3.12, Au 21.17, B 1.16, Cl 3.05, F 8.17, N 6.02. This compound was further characterized by single-crystal X-ray diffraction analysis.

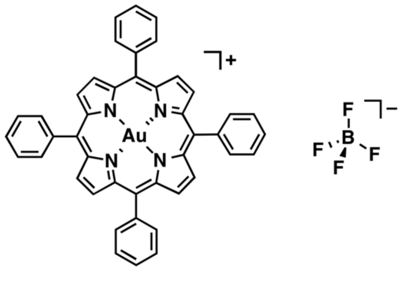

Au^{III} complex of 2H8 as a BF_4^- salt, Au8$^+$-BF_4^-. $AgBF_4$ was used for anion exchange. After the workup, the residue was purified by chromatography over a silica gel column (Wakogel C-300, eluent: 5% MeOH/CH_2Cl_2) and recrystallized from CH_2Cl_2/MeOH to give **Au8**$^+$-BF_4^- (68.0 mg, 0.028 mmol, 67%) as a red solid. R_f = 0.23 (5% MeOH/CH_2Cl_2). ^{1}H NMR (600 MHz, $CDCl_3$, 20 °C): δ(ppm): 9.33 (s, 8H, β-H), 7.44 (s, 8H, Ar-H), 4.31 (t, J = 6.6 Hz, 8H, OCH_2), 4.08 (t, J = 6.6 Hz, 16H, OCH_2), 1.97 (quin, J = 7.8 Hz, 8H, $OCH_2\underline{CH_2}$), 1.86 (quin, J = 7.2 Hz, 16H, $OCH_2\underline{CH_2}$), 1.67 (quin, J = 7.8 Hz, 8H, $O(CH_2)_2\underline{CH_2}$), 1.49–1.22 (m, 112H, $O(CH_2)_2\underline{CH_2}$ + $O(CH_2)_3\underline{(CH_2)_4}$), 0.94 (t, J = 7.2 Hz, 12H, $O(CH_2)_7\underline{CH_3}$), 0.84 (t, J = 7.2 Hz, 24H, $O(CH_2)_7\underline{CH_3}$). ^{13}C NMR (151 MHz, $CDCl_3$, 20 °C): δ(ppm) 152.04, 139.00, 137.19, 133.80, 132.13, 123.59, 114.07, 73.98, 69.69, 32.14, 31.94, 30.73, 29.82, 29.64, 29.60, 29.54, 29.41, 26.43, 26.27, 22.92, 22.79, 14.31, 14.21 (some of the signals for octyl chains were overlapped). ^{19}F NMR (564 MHz, $CDCl_3$, 20 °C): δ(ppm) –159.65 (s, $^{10}BF_4^-$), –159.70 (s, $^{11}BF_4^-$). UV/vis (CH_2Cl_2, λ_{max}[nm] (ε, 10^5 $M^{-1}cm^{-1}$)): 429 (1.2), 529 (0.22). Elemental analysis: C 68.92, H 9.29, N 2.28. Calcd (%) for $C_{140}H_{220}AuBF_4N_4O_{12}$: C 69.05, H 9.11, Au 8.09, B 0.44, F 3.12, N 2.30, O 7.89.

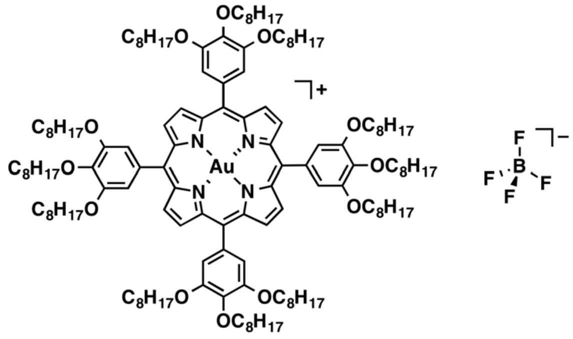

Au^{III} complex of 2H12 as a BF_4^- salt, Au12$^+$-BF_4^-. $AgBF_4$ was used for anion exchange. After the workup, the residue was purified by chromatography over a silica gel column (Wakogel C-300, eluent: 5% MeOH/CH_2Cl_2) and recrystallized from CH_2Cl_2/MeOH to give **Au12**$^+$-BF_4^- (37.9 mg, 0.012 mmol, 75%) as a red solid. R_f = 0.32 (5% MeOH/CH_2Cl_2). ^{1}H NMR (600 MHz, $CDCl_3$, 20 °C): δ(ppm): 9.32 (s, 8H, β-H), 7.44 (s, 8H, Ar-H), 4.30 (t, J = 6.6 Hz, 8H, OCH_2), 4.08 (t, J = 6.6 Hz, 16H, OCH_2), 1.97 (quin, J = 7.2 Hz, 8H, $OCH_2\underline{CH_2}$), 1.86 (quin, J = 6.6 Hz, 16H, $OCH_2\underline{CH_2}$), 1.67 (quin, J = 7.2 Hz, 8H, $O(CH_2)_2\underline{CH_2}$), 1.51–1.21 (m, 208H, $O(CH_2)_2\underline{CH_2}$ + $O(CH_2)_3\underline{(CH_2)_8}$), 0.90 (t, J = 6.6 Hz, 12H, $O(CH_2)_{11}\underline{CH_3}$), 0.84 (t, J = 7.2 Hz, 24H, $O(CH_2)_{11}\underline{CH_3}$). ^{13}C NMR (151 MHz, $CDCl_3$, 20 °C): δ(ppm) 152.06, 139.03, 137.21, 133.82, 132.14, 123.60, 114.09, 74.00, 69.73, 32.13, 32.04, 30.77, 30.03, 29.98, 29.90, 29.83, 29.77, 29.64, 29.62, 29.59, 29.49, 26.47, 26.32, 22.88, 22.81, 14.29, 14.24 (some of the signals for dodecyl chains were overlapped). ^{19}F NMR (564 MHz, $CDCl_3$, 20 °C): δ(ppm) –159.56 (s, $^{10}BF_4^-$), –159.62 (s, $^{11}BF_4^-$). UV/vis (CH_2Cl_2, λ_{max}[nm] (ε, 10^5 $M^{-1}cm^{-1}$)): 428 (1.4), 529 (0.27). Elemental analysis: C 72.72, H 10.48, N 1.86. Calcd (%) for $C_{188}H_{316}AuBF_4N_4O_{12}$: C 72.64, H 10.25, Au 6.34, B 0.35, F 2.44, N 1.80, O 6.18.

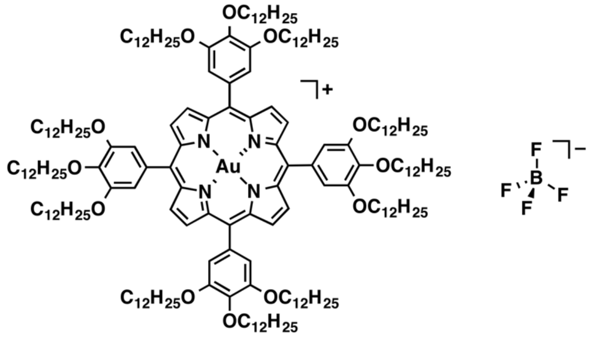

Au^III complex of 2H16 as a BF_4^- salt, Au16⁺-BF_4^-. $AgBF_4$ was used for anion exchange. After the workup, the residue was purified by chromatography over a silica gel column (Wakogel C-300, eluent: 5% $MeOH/CH_2Cl_2$) and recrystallized from CH_2Cl_2/MeOH to give **Au16**$^+$-BF_4^- (43.0 mg, 0.012 mmol, 87%) as a red solid. R_f = 0.33 (5% $MeOH/CH_2Cl_2$). ^1H NMR (600 MHz, $CDCl_3$, 20 °C): δ(ppm): 9.32 (s, 8H, β-H), 7.44 (s, 8H, Ar-H), 4.30 (t, *J* = 6.6 Hz, 8H, OCH_2), 4.08 (t, *J* = 6.6 Hz, 16H, OCH_2), 1.97 (quin, *J* = 7.2 Hz, 8H, $OCH_2\underline{CH_2}$), 1.86 (quin, *J* = 6.6 Hz, 16H, $OCH_2\underline{CH_2}$), 1.67 (quin, *J* = 7.2 Hz, 8H, $O(CH_2)_2\underline{CH_2}$), 1.51–1.21 (m, 304H, $O(CH_2)_2\underline{CH_2}$ + $O(CH_2)_3\underline{(CH_2)_{12}}$), 0.88 (t, *J* = 7.2 Hz, 12H, $O(CH_2)_{15}\underline{CH_3}$), 0.86 (t, *J* = 7.2 Hz, 24H, $O(CH_2)_{15}\underline{CH_3}$). ^{13}C NMR (151 MHz, $CDCl_3$, 20 °C): δ(ppm) 152.06, 139.03, 137.21, 133.83, 132.13, 123.60, 114.10, 74.00, 69.73, 32.10, 32.06, 30.78, 30.05, 30.01, 29.96, 29.91, 29.86, 29.80, 29.65, 29.55, 29.50, 26.48, 26.33, 22.86, 22.83, 14.28, 14.25 (some of the signals for hexadecyl chains were overlapped). ^{19}F NMR (564 MHz, $CDCl_3$, 20 °C): δ(ppm) –159.55 (s, $^{10}BF_4^-$), –159.61 (s, $^{11}BF_4^-$). UV/vis (CH_2Cl_2, λ_{max}[nm] (ε, 10^5 $M^{-1}cm^{-1}$)): 429 (1.1), 529 (0.20). Elemental analysis: C 74.96, H 11.16, N 1.41. Calcd (%) for $C_{236}H_{412}AuBF_4N_4O_{12}$: C 74.96, H 10.98, Au 5.21, B 0.29, F 2.01, N 1.48, O 5.08.

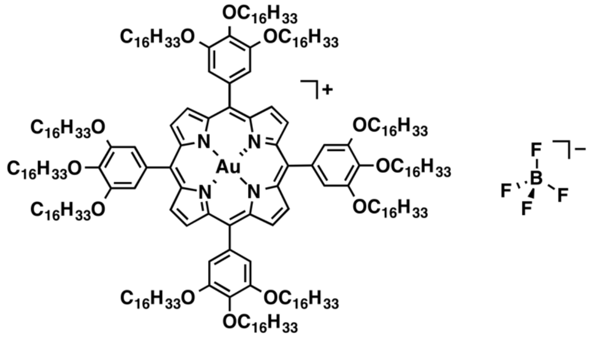

Au^III complex of 2H20 as a BF_4^- salt, Au20⁺-BF_4^-. $AgBF_4$ was used for anion exchange. After the workup, the residue was purified by chromatography over a silica gel column (Wakogel C-300, eluent: 5% $MeOH/CH_2Cl_2$) and recrystallized from CH_2Cl_2/MeOH to give **Au20**$^+$-BF_4^- (24.5 mg, 0.0055 mmol, 61%) as a red solid. R_f = 0.30 (5% $MeOH/CH_2Cl_2$). ^1H NMR (600 MHz, $CDCl_3$, 20 °C): δ(ppm): 9.33 (s, 8H, β-H), 7.44 (s, 8H, Ar-H), 4.30 (t, *J* = 6.6 Hz, 8H, OCH_2), 4.08 (t, *J* = 6.0 Hz, 16H, OCH_2), 1.97 (quin, *J* = 7.2 Hz, 8H, $OCH_2\underline{CH_2}$), 1.85 (quin, *J* = 7.2 Hz, 16H, $OCH_2\underline{CH_2}$), 1.67 (quin, *J* = 7.2 Hz, 8H, $O(CH_2)_2\underline{CH_2}$), 1.51–1.21 (m, 400H, $O(CH_2)_2\underline{CH_2}$ + $O(CH_2)_3\underline{(CH_2)_{16}}$), 0.87 (t, *J* = 7.2 Hz, 12H, $O(CH_2)_{19}\underline{CH_3}$), 0.86 (t, *J* = 7.2 Hz, 24H, $O(CH_2)_{19}\underline{CH_3}$). ^{13}C NMR (151 MHz, $CDCl_3$, 20 °C): δ(ppm) 152.04, 138.99, 137.20, 133.82, 132.11, 123.59, 114.06, 73.98, 69.71, 32.08, 30.79, 30.06, 30.03, 29.98, 29.93, 29.87, 29.82, 29.66, 29.52, 26.48, 26.34, 22.85, 14.27 (some of the signals for icosyl chains were overlapped). ^{19}F NMR (564 MHz, $CDCl_3$, 20 °C): δ(ppm) –159.58 (s, $^{10}BF_4^-$), –159.63 (s, $^{11}BF_4^-$). UV/vis (CH_2Cl_2, λ_{max}[nm] (ε, 10^5 $M^{-1}cm^{-1}$)): 429 (1.3), 530 (0.25). Elemental analysis: C 76.78, H 11.30, N 1.31. Calcd (%) for $C_{284}H_{508}AuBF_4N_4O_{12}$: C 76.57, H 11.49, Au 4.42, B 0.24, F 1.71, N 1.26, O 4.31.

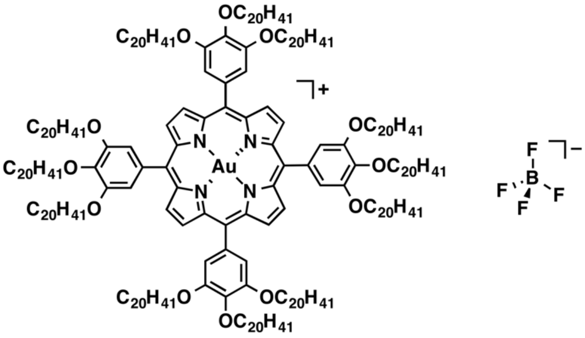

Au^{III} complex of 2H0 as a PF_6^- salt, Au0$^+$-PF_6^-. $AgPF_6$ was used for anion exchange. After the workup, the residue was purified by chromatography over a silica gel column (Wakogel C-300, eluent: 5% MeOH/CH_2Cl_2) and recrystallized from CH_2Cl_2/*n*-hexane to give **Au0**$^+$-PF_6^- (17.4 mg, 0.020 mmol, 55%) as a red solid. R_f = 0.42 (5% MeOH/CH_2Cl_2). ^{1}H NMR (600 MHz, $CDCl_3$, 20 °C): δ(ppm): 9.27 (s, 8H, β-H), 8.26–8.24 (m, 8H, Ph), 7.92–7.84 (m, 12H, Ph). ^{13}C NMR (151 MHz, $CDCl_3$, 20 °C): δ(ppm) 138.70, 137.10, 134.33, 132.43, 129.58, 127.87, 123.72. ^{19}F NMR (564 MHz, $CDCl_3$, 20 °C): δ(ppm) –79.17 (d, J = 712 Hz, 6F, PF_6^-). UV/vis (CH_2Cl_2, λ_{max}[nm] (ε, 10^5 $M^{-1}cm^{-1}$)): 409 (4.0), 521 (0.19). Elemental analysis: C 54.61, H 2.83, F 11.72, N 5.79. Calcd (%) for $C_{44}H_{28}AuF_6N_4P\cdot 0.2CH_2Cl_2$: C 54.64, H 2.95, Au 20.27, Cl 1.46, F 11.73, N 5.77, P 3.19. This compound was further characterized by single-crystal X-ray diffraction analysis.

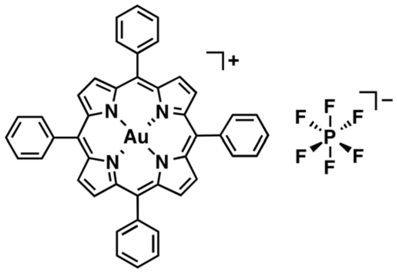

Au^{III} complex of 2H8 as a PF_6^- salt, Au8$^+$-PF_6^-. $AgPF_6$ was used for anion exchange. After the workup, the residue was purified by chromatography over a silica gel column (Wakogel C-300, eluent: 5% MeOH/CH_2Cl_2) and recrystallized from CH_2Cl_2/MeOH to give **Au8**$^+$-PF_6^- (81.0 mg, 0.033 mmol, 78%) as a red solid. R_f = 0.33 (5% MeOH/CH_2Cl_2). ^{1}H NMR (600 MHz, $CDCl_3$, 20 °C): δ(ppm): 9.33 (s, 8H, β-H), 7.44 (s, 8H, Ar-H), 4.31 (t, J = 6.6 Hz, 8H, OCH_2), 4.09 (t, J = 6.6 Hz, 16H, OCH_2), 1.97 (quin, J = 7.8 Hz, 8H, $OCH_2\underline{CH_2}$), 1.86 (quin, J = 7.8 Hz, 16H, $OCH_2\underline{CH_2}$), 1.67 (quin, J = 7.8 Hz, 8H, $O(CH_2)_2\underline{CH_2}$), 1.49–1.20 (m, 112H, $O(CH_2)_2\underline{CH_2}$ + $O(CH_2)_3\underline{(CH_2)_4}$), 0.94 (t, J = 7.2 Hz, 12H, $O(CH_2)_7\underline{CH_3}$), 0.84 (t, J = 7.2 Hz, 24H, $O(CH_2)_7\underline{CH_3}$). ^{13}C NMR (151 MHz, $CDCl_3$, 20 °C): δ(ppm) 152.05, 139.01, 137.21, 133.74, 132.18, 123.62, 114.05, 73.98, 69.69, 32.14, 31.94, 30.73, 29.82, 29.65, 29.60, 29.54, 29.41, 26.43, 26.27, 22.92, 22.79, 14.32, 14.22 (some of the signals for octyl chains were overlapped). ^{19}F NMR (564 MHz, $CDCl_3$, 20 °C): δ(ppm) –79.17 (d, J = 715 Hz, 6F, PF_6^-). UV/vis (CH_2Cl_2, λ_{max}[nm] (ε, 10^5 $M^{-1}cm^{-1}$)): 429 (1.1), 529 (0.22). Elemental analysis: C 67.52, H 9.11, F 4.53, N 2.15. Calcd (%) for $C_{140}H_{220}AuF_6N_4O_{12}P$: C 67.44, H 8.89, Au 7.90, F 4.57, N 2.25, O 7.70, P 1.24.

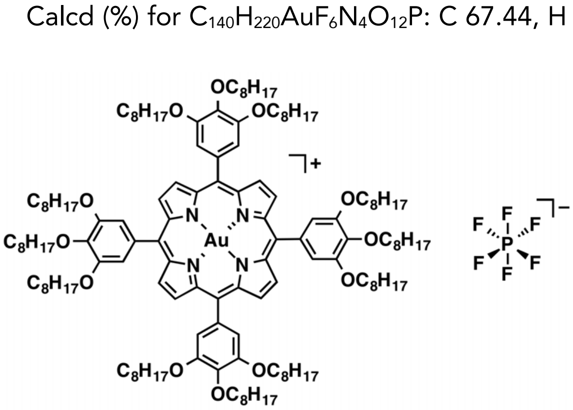

Au^{III} complex of 2H12 as a PF_6^- salt, Au12$^+$-PF_6^-. $AgPF_6$ was used for anion exchange. After the workup, the residue was purified by chromatography over a silica gel column (Wakogel C-300, eluent: 5%

$MeOH/CH_2Cl_2$) and recrystallized from CH_2Cl_2/MeOH to give **Au12**$^+$-PF_6^- (34.0 mg, 0.011 mmol, 66%) as a red solid. R_f = 0.49 (5% $MeOH/CH_2Cl_2$). ^1H NMR (600 MHz, $CDCl_3$, 20 °C): δ(ppm): 9.33 (s, 8H, β-H), 7.44 (s, 8H, Ar-H), 4.30 (t, J = 6.6 Hz, 8H, OCH_2), 4.09 (t, J = 6.6 Hz, 16H, OCH_2), 1.96 (quin, J = 7.8 Hz, 8H, $OCH_2\underline{CH_2}$), 1.86 (quin, J = 7.2 Hz, 16H, $OCH_2\underline{CH_2}$), 1.67 (quin, J = 7.8 Hz, 8H, $O(CH_2)_2\underline{CH_2}$), 1.52–1.21 (m, 208H, $O(CH_2)_2\underline{CH_2}$ + $O(CH_2)_3\underline{(CH_2)_8}$), 0.90 (t, J = 6.6 Hz, 12H, $O(CH_2)_{11}\underline{CH_3}$), 0.84 (t, J = 7.2 Hz, 24H, $O(CH_2)_{11}\underline{CH_3}$). ^{13}C NMR (151 MHz, $CDCl_3$, 20 °C): δ(ppm) 152.06, 139.01, 137.21, 133.75, 132.18, 123.62, 114.04, 73.99, 69.71, 32.13, 32.05, 30.77, 30.04, 29.99, 29.92, 29.85, 29.79, 29.63, 29.60, 29.50, 26.47, 26.31, 22.89, 22.82, 14.30, 14.25 (some of the signals for dodecyl chains were overlapped). ^{19}F NMR (564 MHz, $CDCl_3$, 20 °C): δ(ppm) –79.17 (d, J = 712 Hz, 6F, PF_6^-). UV/vis (CH_2Cl_2, λ_{max}[nm] (ε, 10^5 $M^{-1}cm^{-1}$)): 429 (1.3), 530 (0.24). Elemental analysis: C 71.48, H 10.23, F 3.54, N 1.72. Calcd (%) for $C_{188}H_{316}AuF_6N_4O_{12}P$: C 71.31, H 10.06, Au 6.22, F 3.60, N 1.77, O 6.06, P 0.98.

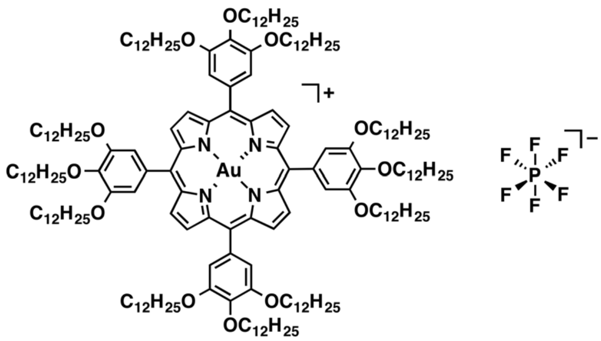

Au^{III} complex of 2H16 as a PF_6^- salt, Au16$^+$-PF_6^-. $AgPF_6$ was used for anion exchange. After the workup, the residue was purified by chromatography over a silica gel column (Wakogel C-300, eluent: 5% $MeOH/CH_2Cl_2$) and recrystallized from CH_2Cl_2/MeOH to give **Au16**$^+$-PF_6^- (42.4 mg, 0.011 mmol, 82%) as a red solid. R_f = 0.49 (5% $MeOH/CH_2Cl_2$). ^1H NMR (600 MHz, $CDCl_3$, 20 °C): δ(ppm): 9.33 (s, 8H, β-H), 7.44 (s, 8H, Ar-H), 4.30 (t, J = 6.6 Hz, 8H, OCH_2), 4.09 (t, J = 6.6 Hz, 16H, OCH_2), 1.97 (quin, J = 7.2 Hz, 8H, $OCH_2\underline{CH_2}$), 1.86 (quin, J = 6.6 Hz, 16H, $OCH_2\underline{CH_2}$), 1.67 (quin, J = 7.8 Hz, 8H, $O(CH_2)_2\underline{CH_2}$), 1.53–1.21 (m, 304H, $O(CH_2)_2\underline{CH_2}$ + $O(CH_2)_3\underline{(CH_2)_{12}}$), 0.88 (t, J = 7.2 Hz, 12H, $O(CH_2)_{15}\underline{CH_3}$), 0.86 (t, J = 7.2 Hz, 24H, $O(CH_2)_{15}\underline{CH_3}$). ^{13}C NMR (151 MHz, $CDCl_3$, 20 °C): δ(ppm) 152.05, 139.00, 137.21, 133.75, 132.18, 123.62, 114.03, 73.98, 69.70, 32.10, 32.07, 30.78, 30.05, 30.01, 29.98, 29.95, 29.93, 29.85, 29.80, 29.65, 29.55, 29.50, 26.48, 26.32, 22.86, 22.83, 14.28, 14.26 (some of the signals for hexadecyl chains were overlapped). ^{19}F NMR (564 MHz, $CDCl_3$, 20 °C): δ(ppm) –79.20 (d, J = 714 Hz, 6F, PF_6^-). UV/vis (CH_2Cl_2, λ_{max}[nm] (ε, 10^5 $M^{-1}cm^{-1}$)): 429 (1.3), 530 (0.24). Elemental analysis: C 73.71, H 10.60, F 3.10, N 1.41. Calcd (%) for $C_{236}H_{412}AuF_6N_4O_{12}P$: C 73.82, H 10.82, Au 5.13, F 2.97, N 1.46, O 5.00, P 0.81.

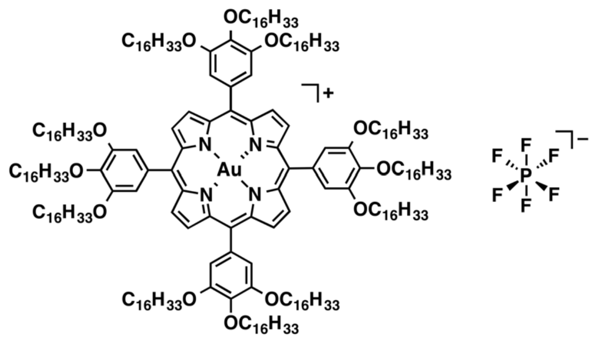

Au^{III} complex of 2H20 as a PF_6^- salt, Au20$^+$-PF_6^-. $AgPF_6$ was used for anion exchange. After the workup, the residue was purified by chromatography over a silica gel column (Wakogel C-300, eluent: 5% $MeOH/CH_2Cl_2$) and recrystallized from CH_2Cl_2/MeOH to give **Au20**$^+$-PF_6^- (27.9 mg, 0.0062 mmol, 68%) as a red solid. R_f = 0.44 (5% $MeOH/CH_2Cl_2$). ^1H NMR (600 MHz, $CDCl_3$, 20 °C): δ(ppm): 9.33 (s, 8H, β-H), 7.44 (s, 8H, Ar-H), 4.30 (t, J = 6.6 Hz, 8H, OCH_2), 4.08 (t, J = 6.6 Hz, 16H, OCH_2), 1.97 (quin, J = 7.2 Hz, 8H, $OCH_2\underline{CH_2}$), 1.86 (quin, J = 7.2 Hz, 16H, $OCH_2\underline{CH_2}$), 1.67 (quin, J = 7.2 Hz, 8H, $O(CH_2)_2\underline{CH_2}$), 1.51–1.21 (m, 400H, $O(CH_2)_2\underline{CH_2}$ + $O(CH_2)_3\underline{(CH_2)_{16}}$), 0.87 (t, J = 7.2 Hz, 12H, $O(CH_2)_{19}\underline{CH_3}$), 0.86 (t, J = 7.8 Hz, 24H, $O(CH_2)_{19}\underline{CH_3}$). ^{13}C NMR (151 MHz, $CDCl_3$, 20 °C): δ(ppm) 152.06, 139.02, 137.21, 133.75, 132.18, 123.62,

114.04, 73.99, 69.71, 32.08, 30.79, 30.06, 30.03, 30.00, 29.98, 29.94, 29.87, 29.82, 29.66, 29.52, 26.49, 26.33, 22.85, 14.27 (some of the signals for icosyl chains were overlapped). ^{19}F NMR (564 MHz, $CDCl_3$, 20 °C): δ (ppm) –79.00 (d, J = 713 Hz, 6F, PF_6^-). UV/vis (CH_2Cl_2, λ_{max}[nm] (ε, 10^5 $M^{-1}cm^{-1}$)): 429 (0.90), 530 (0.17). Elemental analysis: C 75.64, H 11.56, F 2.43, N 1.20. Calcd (%) for $C_{284}H_{512}AuF_6N_4O_{12}P$: C 75.58, H 11.35, Au 4.36, F 2.53, N 1.24, O 4.25, P 0.69.

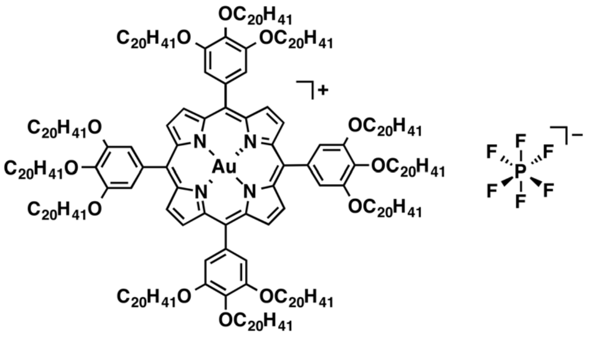

Au^{III} complex of 2H0 as a PCCp$^-$ salt, Au0$^+$-PCCp$^-$. Sodium pentacyanocyclopentadienide (NaPCCp) (Sakai et al., 2013) was used for anion exchange. After the workup, the residue was purified by chromatography over a silica gel column (Wakogel C-300, eluent: 5% MeOH/CH_2Cl_2) and recrystallized from CH_2Cl_2/n-hexane to give **Au0**$^+$-PCCp$^-$ (29.2 mg, 0.029 mmol, 82%) as a red solid. R_f = 0.52 (5% MeOH/CH_2Cl_2). ^{1}H NMR (600 MHz, $CDCl_3$, 20 °C): δ (ppm): 9.38 (s, 8H, β-H), 8.38–8.36 (m, 8H, Ph), 7.93–7.87 (m, 12H, Ph). ^{13}C NMR (151 MHz, $CDCl_3$, 20 °C): δ (ppm) 138.66, 137.23, 134.76, 132.71, 129.65, 127.90, 124.15, 110.62, 99.16. UV/vis (CH_2Cl_2, λ_{max}[nm] (ε, 10^5 $M^{-1}cm^{-1}$)): 409 (3.9), 521 (0.18). Elemental analysis: C 64.66, H 3.05, N 12.58. Calcd (%) for $C_{54}H_{28}AuN_9$: C 64.87, H 2.82, Au 19.70, N 12.61. This compound was further characterized by single-crystal X-ray diffraction analysis.

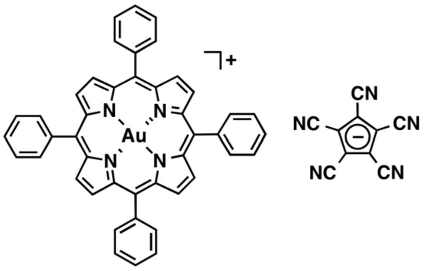

Au^{III} complex of 2H8 as a PCCp$^-$ salt, Au8$^+$-PCCp$^-$. NaPCCp (Sakai et al., 2013) was used for anion exchange. After the workup, the residue was purified by chromatography over a silica gel column (Wakogel C-300, eluent: 5% MeOH/CH_2Cl_2) and recrystallized from CH_2Cl_2/MeOH to give **Au8**$^+$-PCCp$^-$ (55 mg, 0.022 mmol, 51%) as a red solid. R_f = 0.51 (5% MeOH/CH_2Cl_2). ^{1}H NMR (600 MHz, $CDCl_3$, 20 °C): δ (ppm): 9.46 (s, 8H, β-H), 7.63 (s, 8H, Ar-H), 4.32 (t, J = 6.6 Hz, 8H, OCH_2), 4.11 (t, J = 6.6 Hz, 16H, OCH_2), 1.98 (quin, J = 7.8 Hz, 8H, $OCH_2\underline{CH_2}$), 1.86 (quin, J = 7.8 Hz, 16H, $OCH_2\underline{CH_2}$), 1.67 (quin, J = 7.8 Hz, 8H, $O(CH_2)_2\underline{CH_2}$), 1.49–1.20 (m, 112H, $O(CH_2)_2\underline{CH_2}$ + $O(CH_2)_3\underline{(CH_2)_4}$), 0.94 (t, J = 7.2 Hz, 12H, $O(CH_2)_7\underline{CH_3}$), 0.83 (t, J = 7.2 Hz, 24H, $O(CH_2)_7\underline{CH_3}$). ^{13}C NMR (151 MHz, $CDCl_3$, 20 °C): δ (ppm) 152.08, 139.04, 137.21, 133.52, 132.53, 124.07, 114.50, 110.16, 98.44, 73.98, 69.72, 32.16, 31.93, 30.74, 29.83, 29.65, 29.62, 29.54, 29.41, 26.43, 26.28, 22.93 22.78, 14.32, 14.21. UV/vis (CH_2Cl_2, λ_{max}[nm] (ε, 10^5 $M^{-1}cm^{-1}$)): 428 (1.2), 530 (0.23). Elemental analysis: C 70.88, H 8.55, N 4.81. Calcd (%) for $C_{150}H_{220}AuN_9O_{12}$: C 70.98, H 8.74, Au 7.76, N 4.97, O 7.56.

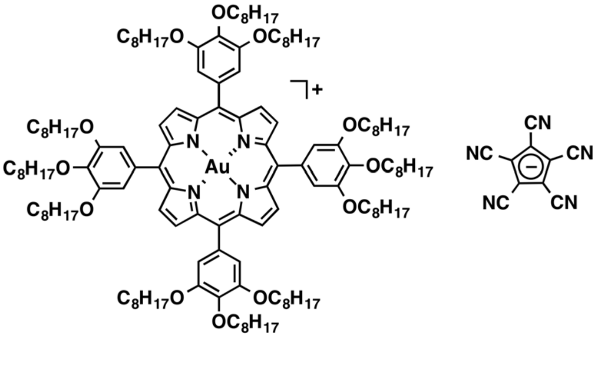

Au^{III} complex of 2H12 as a PCCp$^-$ salt, Au12$^+$-PCCp$^-$. NaPCCp (Sakai et al., 2013) was used for anion exchange. After the workup, the residue was purified by chromatography over a silica gel column (Wakogel C-300, eluent: 5% MeOH/CH_2Cl_2) and recrystallized from CH_2Cl_2/MeOH to give **Au12**$^+$-PCCp$^-$ (75.3 mg, 0.023 mmol, 72%) as a red solid. R_f = 0.76 (5% MeOH/CH_2Cl_2). ^{1}H NMR (600 MHz, $CDCl_3$, 20 °C): δ (ppm): 9.46 (s, 8H, β-H), 7.62 (s, 8H, Ar-H), 4.31 (t, J = 6.6 Hz, 8H, OCH_2), 4.10 (t, J = 6.6 Hz, 16H, OCH_2), 1.98 (quin, J = 7.2 Hz, 8H, $OCH_2\underline{CH_2}$), 1.86 (quin, J = 7.2 Hz, 16H, $OCH_2\underline{CH_2}$), 1.67 (quin, J = 7.8 Hz, 8H, $O(CH_2)_2\underline{CH_2}$), 1.53–1.20 (m, 208H, $O(CH_2)_2\underline{CH_2}$ + $O(CH_2)_3\underline{(CH_2)_8}$), 0.90 (t, J = 7.2 Hz, 12H, $O(CH_2)_{11}\underline{CH_3}$), 0.83 (t, J = 7.2 Hz, 24H, $O(CH_2)_{11}\underline{CH_3}$). ^{13}C NMR (151 MHz, $CDCl_3$, 20 °C): δ (ppm) 152.08, 139.04, 137.22, 133.52, 132.54, 124.08, 114.49, 110.14, 98.43, 73.99, 69.73, 32.13, 32.03, 30.77, 30.03, 29.98, 29.91, 29.82, 29.79, 29.75, 29.64, 29.59, 29.48, 29.47, 26.47, 26.32, 22.80, 14.29, 14.23 (some of the signals for dodecyl chains were overlapped). UV/vis (CH_2Cl_2, λ_{max}[nm] (ε, 10^5 $M^{-1}cm^{-1}$)): 429 (1.2), 530 (0.23). Elemental analysis: C 73.91, H 9.92, N 3.93. Calcd (%) for $C_{198}H_{316}AuN_9O_{12}$: C 74.05, H 9.92, Au 6.13, N 3.93, O: 5.98.

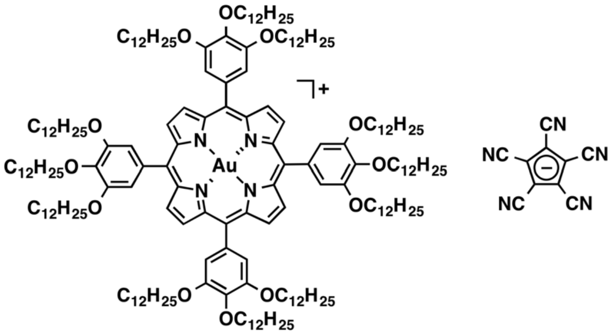

Au^{III} complex of 2H16 as a PCCp$^-$ salt, Au16$^+$-PCCp$^-$. NaPCCp (Sakai et al., 2013) was used for anion exchange. After the workup, the residue was purified by chromatography over a silica gel column (Wakogel C-300, eluent: 5% MeOH/CH_2Cl_2) and recrystallized from CH_2Cl_2/MeOH to give **Au16**$^+$-PCCp$^-$ (70.0 mg, 0.018 mmol, 67%) as a red solid. R_f = 0.80 (5% MeOH/CH_2Cl_2). ^{1}H NMR (600 MHz, $CDCl_3$, 20 °C): δ (ppm): 9.46 (s, 8H, β-H), 7.62 (s, 8H, Ar-H), 4.31 (t, J = 6.6 Hz, 8H, OCH_2), 4.10 (t, J = 6.6 Hz, 16H, OCH_2), 1.98 (quin, J = 7.2 Hz, 8H, $OCH_2\underline{CH_2}$), 1.86 (quin, J = 7.2 Hz, 16H, $OCH_2\underline{CH_2}$), 1.67 (quin, J = 7.2 Hz, 8H, $O(CH_2)_2\underline{CH_2}$), 1.51–1.20 (m, 304H, $O(CH_2)_2\underline{CH_2}$ + $O(CH_2)_3\underline{(CH_2)_{12}}$), 0.88 (t, J = 7.2 Hz, 12H, $O(CH_2)_{15}\underline{CH_3}$), 0.85 (t, J = 7.2 Hz, 24H, $O(CH_2)_{15}\underline{CH_3}$). ^{13}C NMR (151 MHz, $CDCl_3$, 20 °C): δ (ppm) 152.11, 139.07, 137.23, 133.53, 132.55, 124.10, 114.51, 110.17, 98.46, 73.99, 69.75, 32.11, 32.06, 30.79, 30.05, 30.02, 29.98, 29.96, 29.94, 29.85, 29.80, 29.64, 29.55, 29.50, 26.49, 26.34, 22.86, 22.83, 14.28, 14.25 (some of the signals for hexadecyl chains were overlapped). UV/vis (CH_2Cl_2, λ_{max}[nm] (ε, 10^5 $M^{-1}cm^{-1}$)): 429 (1.9), 530 (0.34). Elemental analysis: C 76.09, H 10.81, N 3.27. Calcd (%) for $C_{246}H_{412}AuN_9O_{12}$: C 76.05, H 10.69, Au 5.07, N 3.24, O: 4.94.

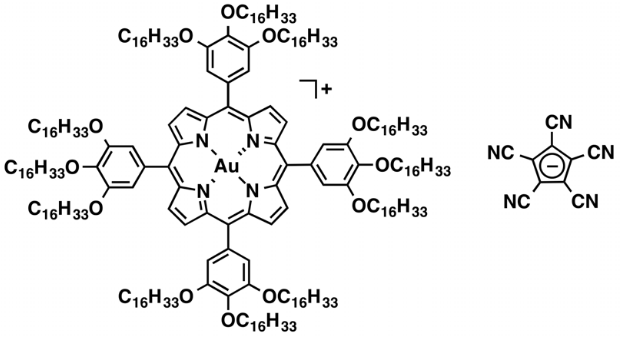

Au^{III} complex of 2H20 as a PCCp$^-$ salt, Au20$^+$-PCCp$^-$. NaPCCp (Sakai et al., 2013) was used for anion exchange. After the workup, the residue was purified by chromatography over a silica gel column (Wakogel C-300, eluent: 5% MeOH/CH_2Cl_2) and recrystallized from CH_2Cl_2/MeOH to give **Au20**$^+$-PCCp$^-$ (51.2 mg, 0.011 mmol, 82%) as a red solid. R_f = 0.71 (5% MeOH/CH_2Cl_2). ^{1}H NMR (600 MHz, $CDCl_3$, 20 °C): δ (ppm): 9.46 (s, 8H, β-H), 7.62 (s, 8H, Ar-H), 4.31 (t, J = 6.6 Hz, 8H, OCH_2), 4.10 (t, J = 6.6 Hz, 16H, OCH_2), 1.97 (quin, J = 7.2 Hz, 8H, $OCH_2\underline{CH_2}$), 1.86 (quin, J = 7.2 Hz, 16H, $OCH_2\underline{CH_2}$), 1.67 (quin, J = 7.8 Hz, 8H, $O(CH)_2\underline{CH_2}$), 1.52–1.20 (m, 400H, $O(CH_2)_2\underline{CH_2}$ + $O(CH_2)_3\underline{(CH_2)_{16}}$), 0.87 (t, J = 7.2 Hz, 12H, $O(CH_2)_{19}\underline{CH_3}$), 0.86 (t, J = 6.6 Hz,

24H, $O(CH_2)_{19}\underline{CH_3}$). ^{13}C NMR (151 MHz, $CDCl_3$, 20 °C): δ (ppm) 152.11, 139.07, 137.22, 133.53, 132.54, 124.10, 114.51. 110.16, 98.45, 73.99, 69.74, 32.10, 32.07, 30.79, 30.06, 30.03, 29.98, 29.94, 29.89, 29.86, 29.81, 29.65, 29.54, 29.51, 26.49, 26.35, 22.84, 14.27 (some of the signals for icosyl chains were overlapped). UV/vis (CH_2Cl_2, λ_{max}[nm] (ε, 10^5 $M^{-1}cm^{-1}$)): 429 (1.2), 530 (0.22). Elemental analysis: C 77.40, H 11.42, N 2.67. Calcd (%) for $C_{294}H_{508}AuN_9O_{12}$: C 77.47, H 11.23, Au 4.23, N 2.77, O 4.21.

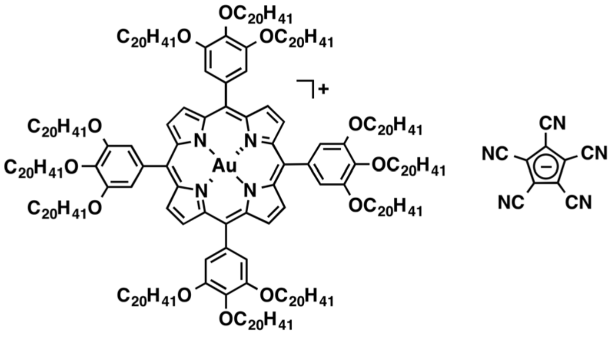

Au^{III} complex of 2H0 as a NiO$^-$ salt, Au0$^+$-NiO$^-$. Ni^{II} complex of 5-hydroxy-10,15,20-tris(pentafluorophenyl)porphyrin (Sasano et al., 2017; Stähler et al., 2017) (**NiOH**) (22.9 mg, 0.026 mmol) in CH_2Cl_2 was treated with an excess amount of aqueous NaOH to yield Na^+-**NiO**$^-$ in a CH_2Cl_2 phase. Then, **Au0**$^+$-Cl^- (23.1 mg, 0.0260 mmol) was added to Na^+-**NiO**$^-$ to form **Au0**$^+$-**NiO**$^-$ after washing with water to remove NaCl. The CH_2Cl_2 solution was filtered and evaporated to dryness. The residue was then recrystallized from EtOAc/*n*-hexane to give **Au0**$^+$-**NiO**$^-$ (21.3 mg, 0.0126 mmol, 48%) as a brown solid. ^{1}H NMR (600 MHz, $CDCl_3$, 20 °C; Figure S35b for the assignment): δ (ppm): 8.78 (s, 8H, β-H (**Au0**$^+$)), 7.92 (d, *J* = 4.2 Hz, 2H, β-H (**NiO**$^-$)), 7.82 (d, *J* = 5.4 Hz, 2H, β-H (**NiO**$^-$)), 7.80 (d, *J* = 8.4 Hz, 8H, Ph (**Au0**$^+$)), 7.76 (t, *J* = 7.8 Hz, 4H, Ph (**Au0**$^+$)), 7.59 (t, *J* = 8.4 Hz, 8H, Ph (**Au0**$^+$)), 6.85 (d, *J* = 3.6 Hz, 2H, β-H (**NiO**$^-$)), 5.54 (br, 2H, β-H (**NiO**$^-$)). ^{13}C NMR (151 MHz, $CDCl_3$, 20 °C): δ (ppm) 146.67 (d, $J_{13C-19F}$ = 251 Hz), 146.46, 146.08 (d, $J_{13C-19F}$ = 249 Hz), 141.94, 141.53 (d, $J_{13C-19F}$ = 244 Hz), 138.92, 138.77, 137.53 (d, $J_{13C-19F}$ = 233 Hz), 136.96, 135.59, 134.64, 131.85, 131.37, 129.32, 127.59, 123.28, 122.32, 122.04, 116.05, 103.27, 93.01 (some signals were overlapped). ^{19}F NMR (564 MHz, $CDCl_3$, 20 °C): δ (ppm) –139.84 (d, *J* = 25.9 Hz, 2F, C_6F_5), –140.72 (d, *J* = 25.9 Hz, 4F, C_6F_5), –157.75 (m, 1F, C_6F_5), –157.87 (m, 2F, C_6F_5), –165.60 (m, 4F, C_6F_5), –165.93 (m, 2F, C_6F_5). UV/vis (CH_2Cl_2, λ_{max}[nm] (ε, 10^5 $M^{-1}cm^{-1}$)): 410 (4.8), 435 (1.3), 524 (0.24), 674 (0.21). Elemental analysis: C 57.87, H 2.75, F 15.99, N 6.38. Calcd (%) for $C_{82}H_{36}AuF_{15}N_8NiO\cdot0.4C_4H_8O_2\cdot0.4C_6H_{14}\cdot1.5H_2O$: C 57.82, H 2.70, Au 11.02, F 15.95, N 6.27, Ni 3.29, O: 2.96.

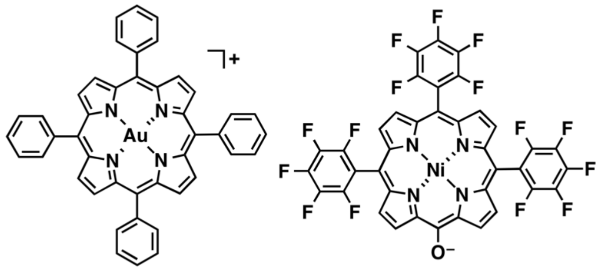

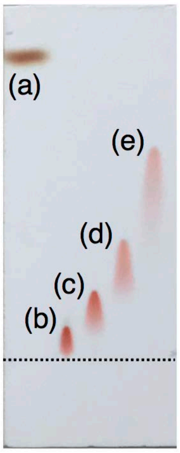

Figure S2 TLC analysis of ion pairs, Related to Figure 3.
TLC analysis for (a) **2H0**, (b) **Au0**$^+$-Cl$^-$, (c) **Au0**$^+$-BF_4^-, (d) **Au0**$^+$-PF_6^-, and (e) **Au0**$^+$-PCCp$^-$ using 5% MeOH/CH_2Cl_2 as an eluent.

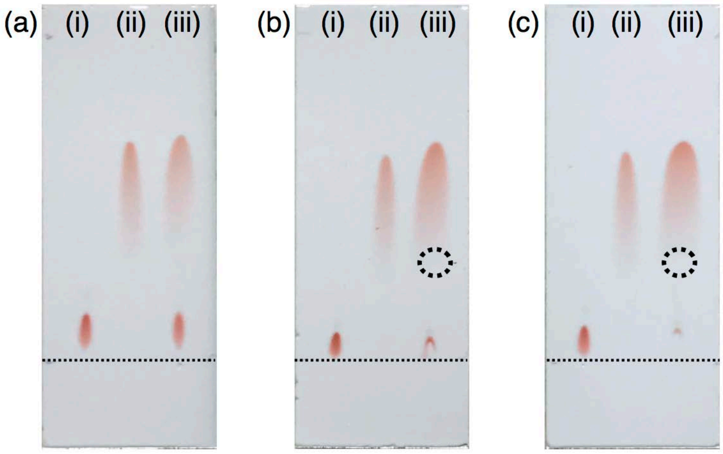

Figure S3 TLC analysis for ion pairs with mixing conditions, Related to Figure 3.
TLC analysis for (a)(i) **Au0**$^+$-Cl$^-$, (ii) **Au0**$^+$-PCCp$^-$, and (iii) the mixture of **Au0**$^+$-Cl$^-$ and **Au0**$^+$-PCCp$^-$, (b)(i) **Au0**$^+$-Cl$^-$, (ii) **Au0**$^+$-PCCp$^-$, and (iii) the mixture of **Au0**$^+$-PCCp$^-$ and tetrabutylammonium chloride (TBACl), (c)(i) **Au0**$^+$-Cl$^-$, (ii) **Au0**$^+$-PCCp$^-$, and (iii) the mixture of **Au0**$^+$-Cl$^-$ and TBAPCCp using 5% MeOH/CH_2Cl_2 as an eluent. Dotted circles in (b) and (c) indicate the spots of TBAPCCp, which were observed under UV_{254} light. As for the preparation process, stock CH_2Cl_2 solutions (1×10^{-3} M) of **Au0**$^+$-Cl$^-$, **Au0**$^+$-PCCp$^-$, TBACl, and TBAPCCp were prepared. The 1:1 mixed ion pair solutions for (iii) in (a–c) were prepared by mixing two ion pair stock solutions by sonication for 30 sec before spotting onto the TLC. On each TLC starting line, **Au0**$^+$-Cl$^-$ (2 μL), **Au0**$^+$-PCCp$^-$ (2 μL), the mixed solution of **Au0**$^+$-Cl$^-$ (2 μL) and **Au0**$^+$-PCCp$^-$ (2 μL), the mixed solution of **Au0**$^+$-PCCp$^-$ (4 μL) and TBACl (4 μL), and the mixed solution of **Au0**$^+$-Cl$^-$ (4 μL) and TBAPCCp (4 μL) were spotted. In (a)(iii), although two ion pairs **Au0**$^+$-Cl$^-$ and **Au0**$^+$-PCCp$^-$ were mixed in the starting spot, they were completely separated on the TLC. In (b)(iii), the TLC analysis of the mixture of **Au0**$^+$-PCCp$^-$ and TBACl showed a distinct spot of **Au0**$^+$-PCCp$^-$ and small amounts of **Au0**$^+$-Cl$^-$ and TBAPCCp, suggesting the unfavorable ion exchange between **Au0**$^+$-PCCp$^-$ and TBACl. Furthermore, in (c)(iii), the TLC analysis of the mixture of **Au0**$^+$-Cl$^-$ and TBAPCCp also showed a distinct spot of **Au0**$^+$-PCCp$^-$, which was formed by the ion exchange, and small amounts of **Au0**$^+$-Cl$^-$ and TBAPCCp. The sizes of the spots of **Au0**$^+$-PCCp$^-$ in (b)(iii) and (c)(iii) are approximately twice of those in (ii) as **Au0**$^+$-PCCp$^-$. These results clearly suggested the stability and the preferential formation of **Au0**$^+$-PCCp$^-$ along with undetectable TBACl during the ion-exchange process.

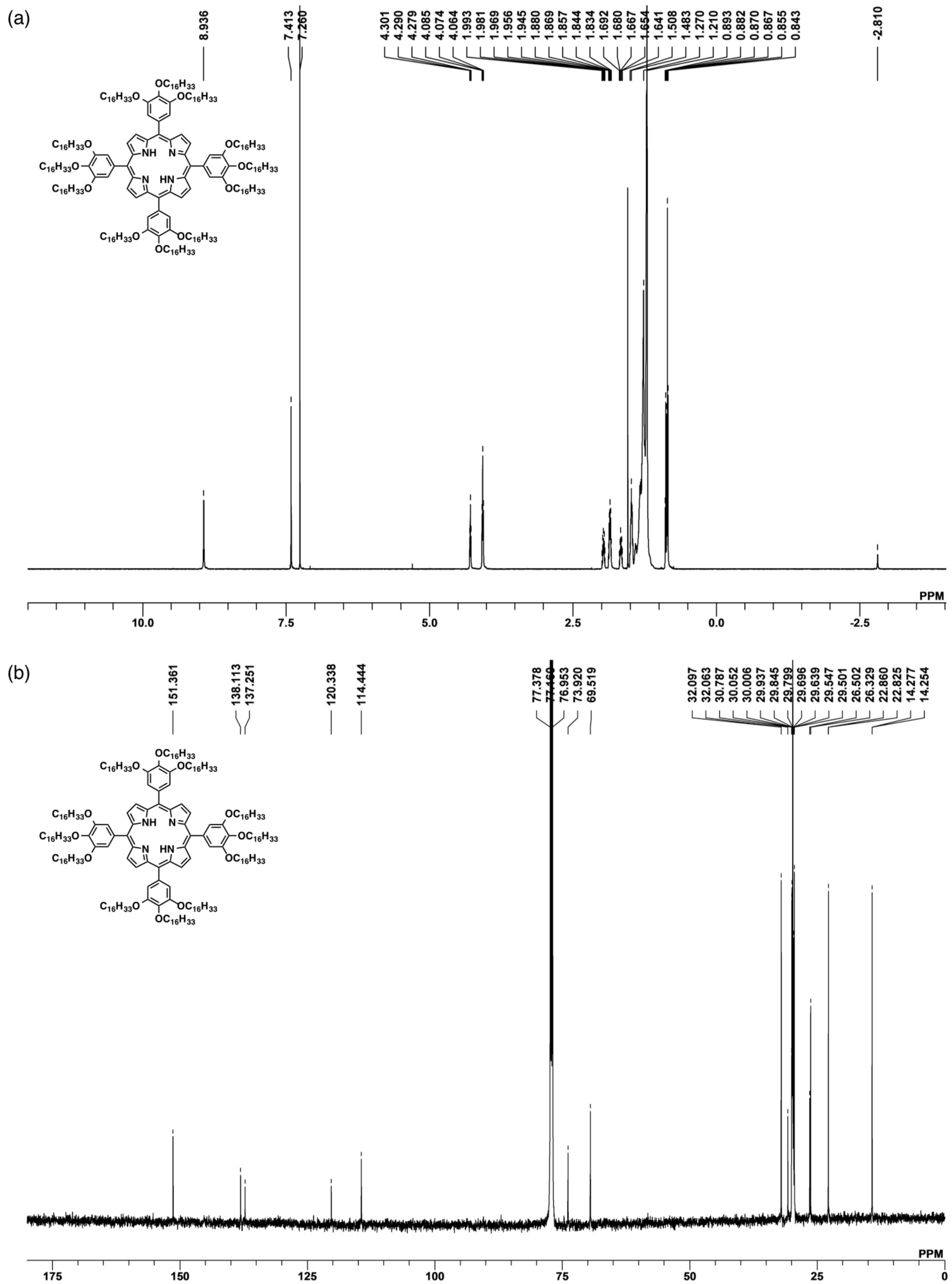

Figure S4 NMR spectra of 2H16, Related to Figure 2.
(a) ^{1}H NMR and (b) ^{13}C NMR spectra of **2H16** in $CDCl_3$ at 20 °C.

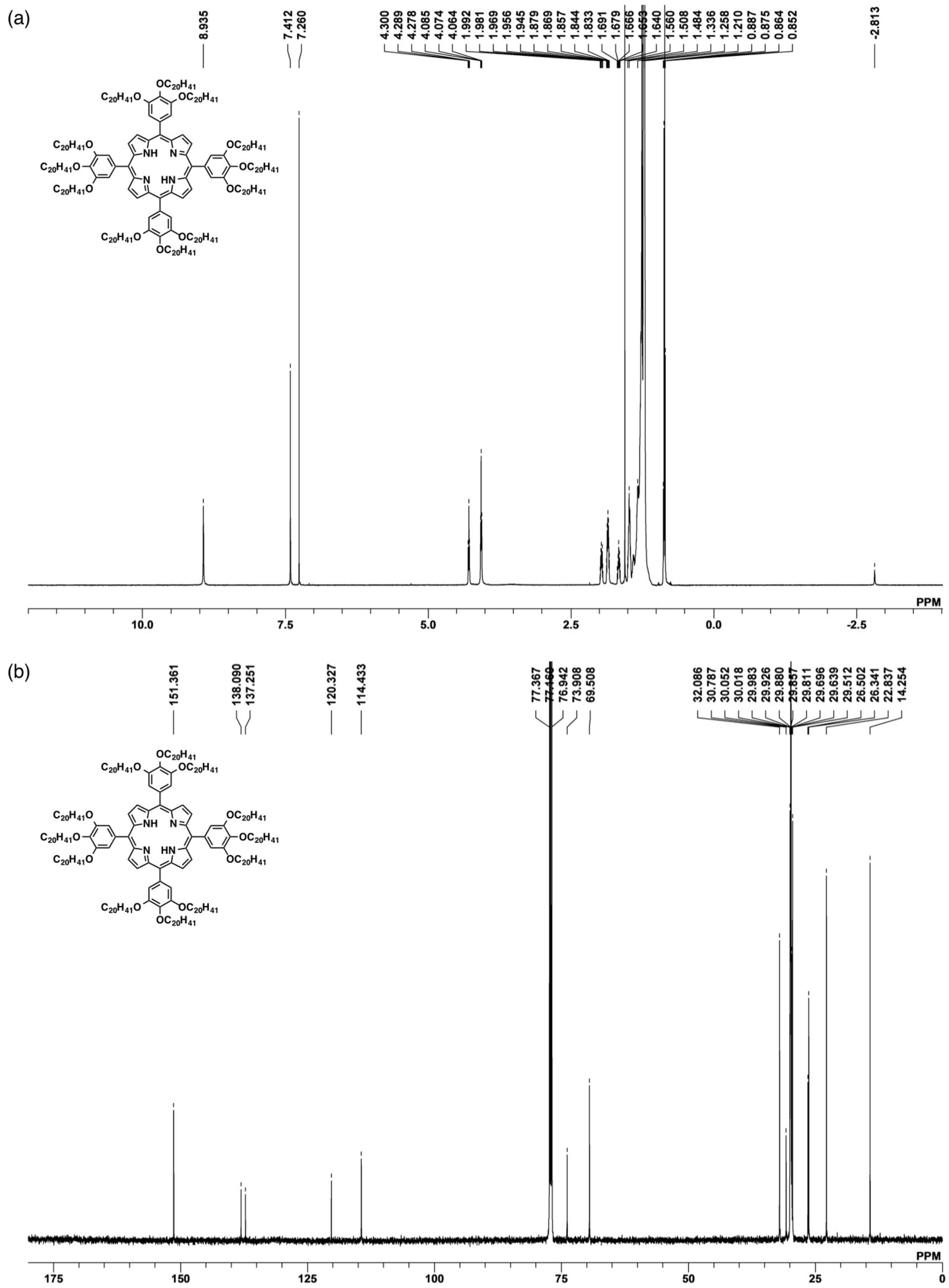

Figure S5 NMR spectra of 2H20, Related to Figure 2.
(a) ^{1}H NMR and (b) ^{13}C NMR spectra of **2H20** in $CDCl_3$ at 20 °C.

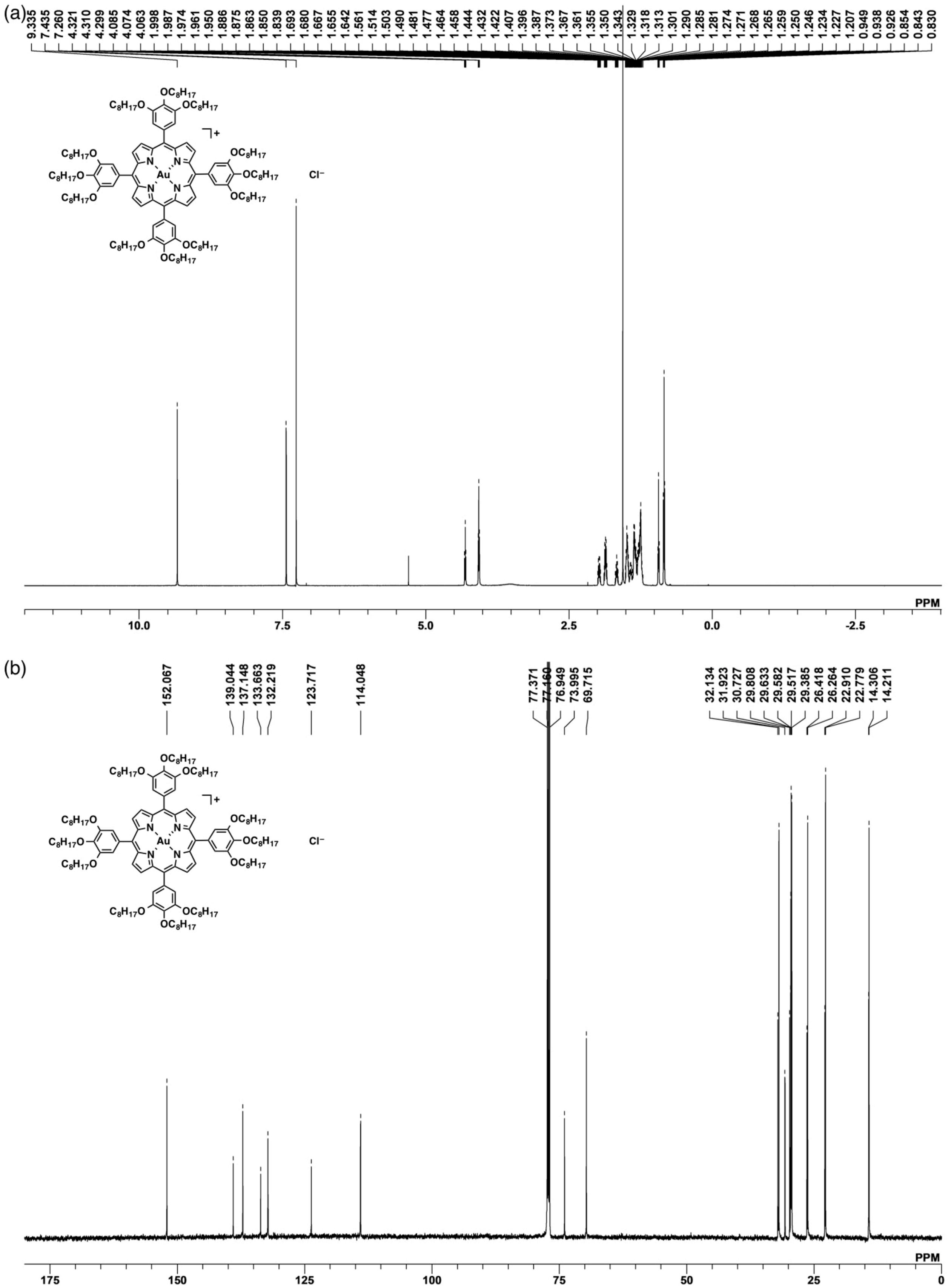

Figure S6 NMR spectra of Au8⁺-Cl⁻, Related to Figure 2.
(a) ^{1}H NMR and (b) ^{13}C NMR spectra of **Au8**⁺-Cl⁻ in $CDCl_3$ at 20 °C.

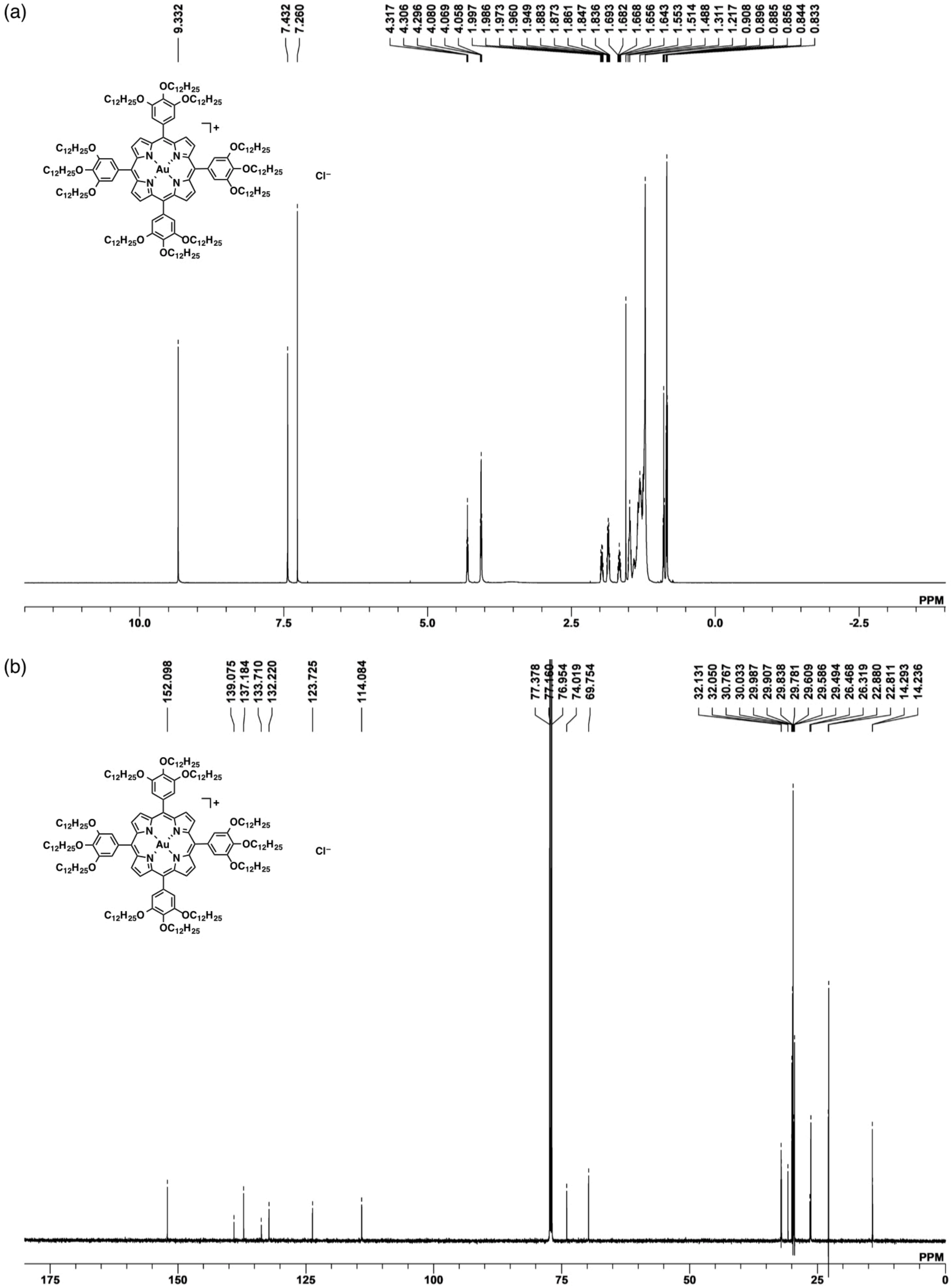

Figure S7 NMR spectra of Au12⁺-Cl⁻, Related to Figure 2.

(a) ^{1}H NMR and (b) ^{13}C NMR spectra of **Au12**⁺-Cl⁻ in $CDCl_3$ at 20 °C.

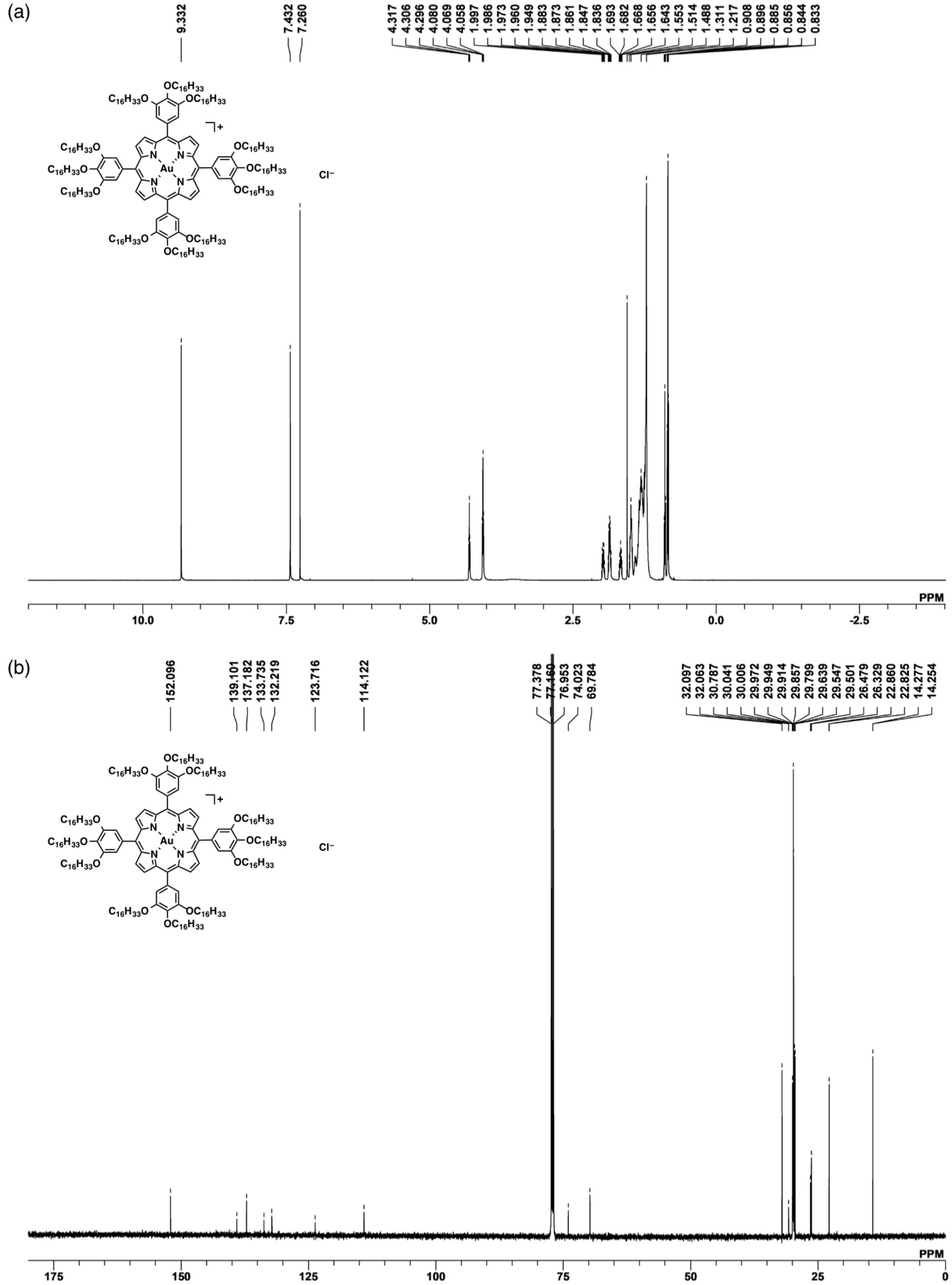

Figure S8 NMR spectra of Au16⁺-Cl⁻, Related to Figure 2.
(a) ^{1}H NMR and (b) ^{13}C NMR spectra of **Au16**⁺-Cl⁻ in $CDCl_3$ at 20 °C.

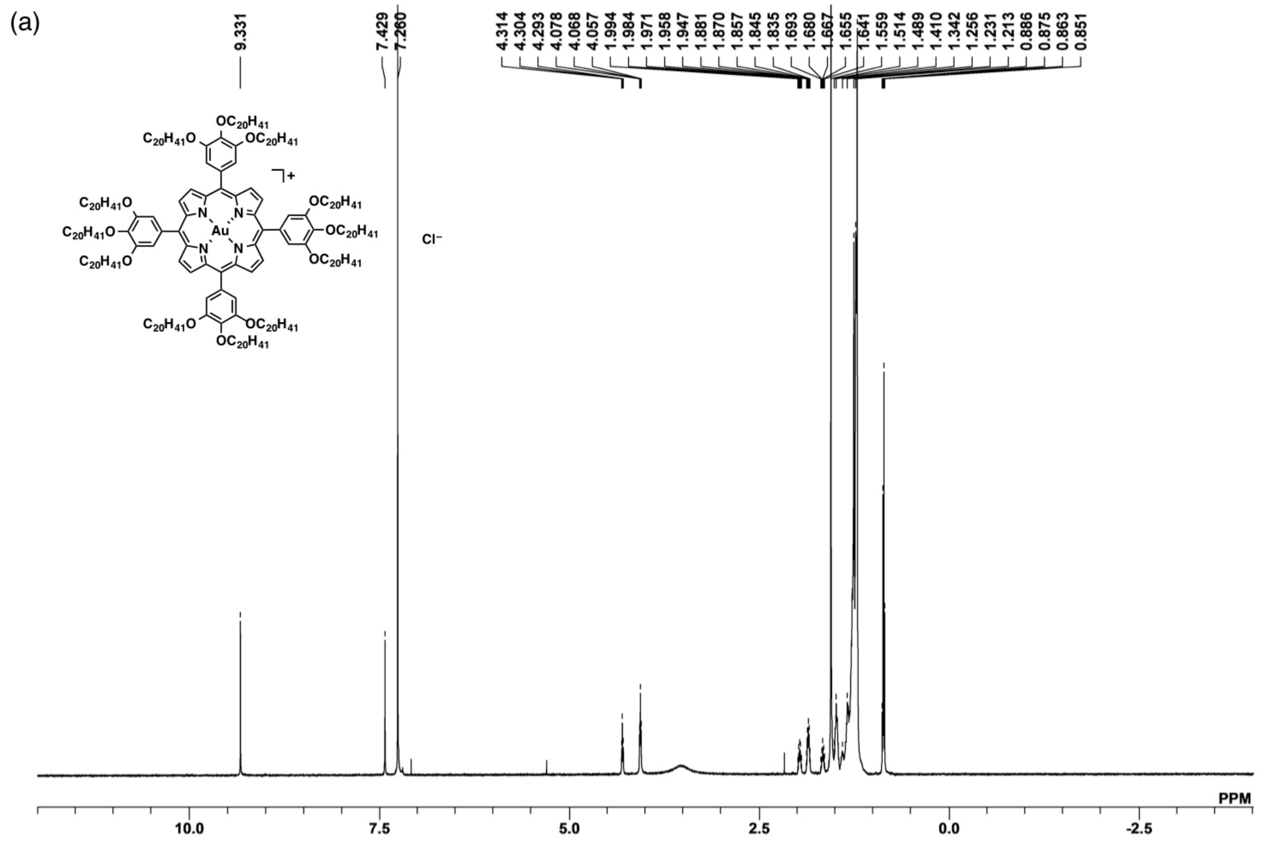

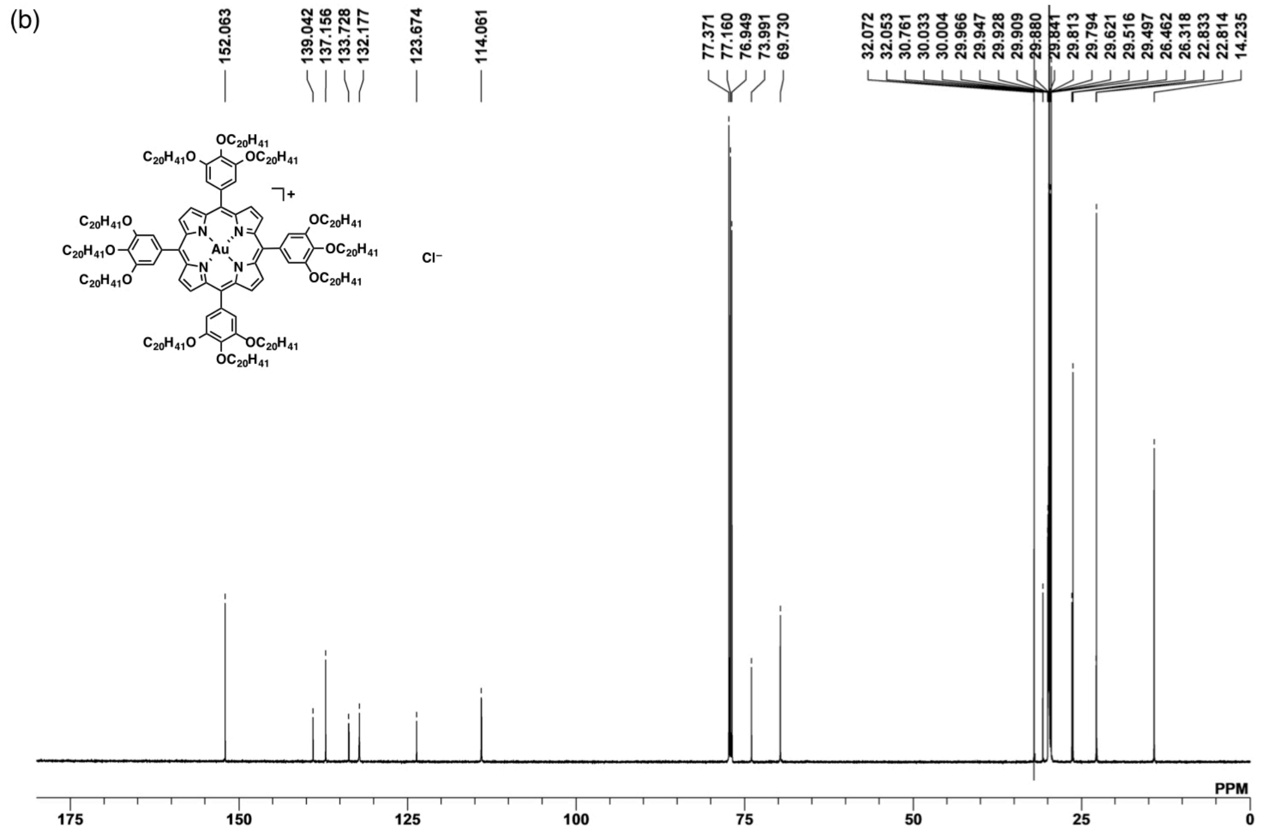

Figure S9 NMR spectra of Au20⁺-Cl⁻, Related to Figure 2.

(a) ^{1}H NMR and (b) ^{13}C NMR spectra of **Au20**⁺-Cl⁻ in $CDCl_3$ at 20 °C.

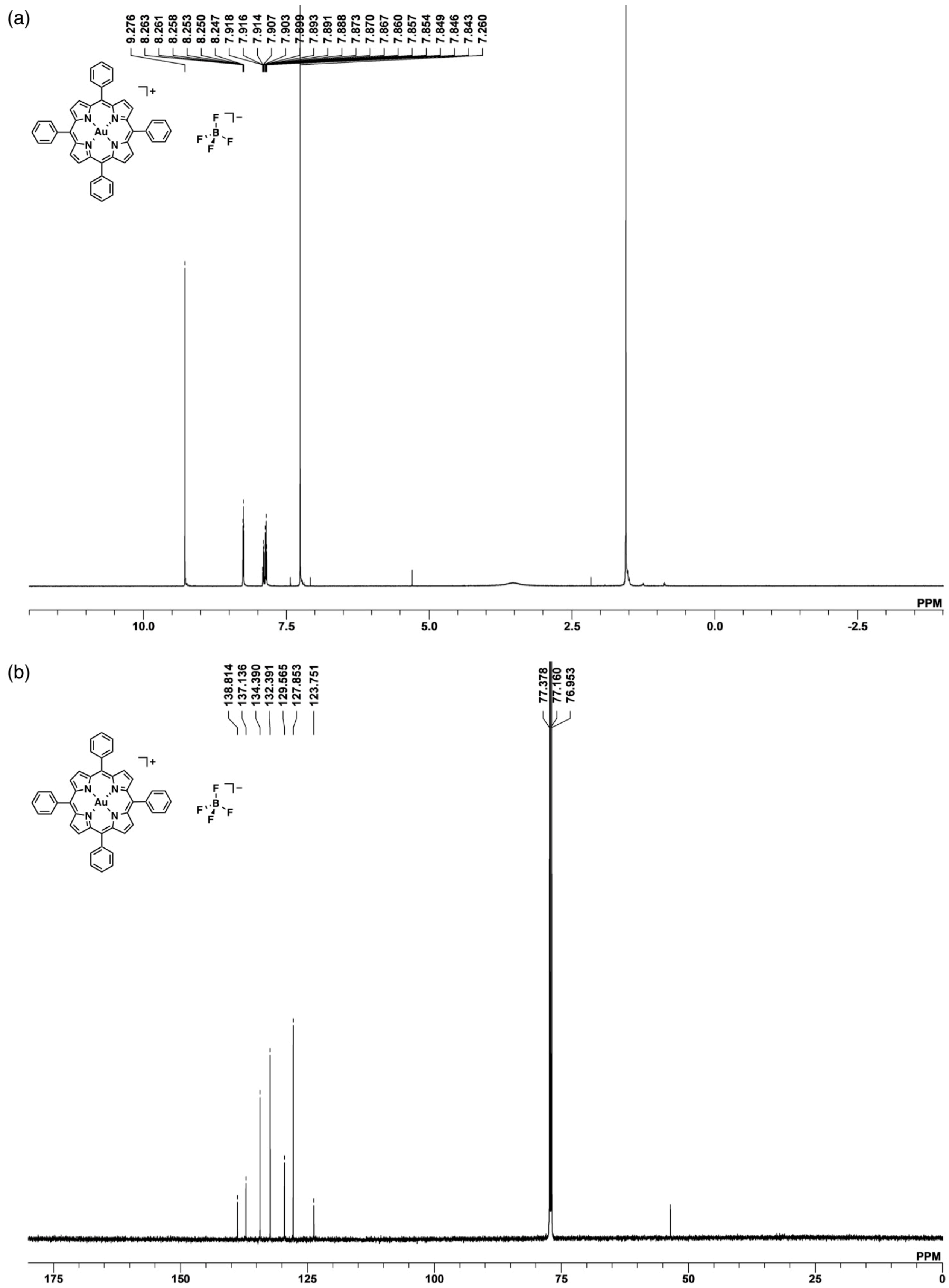

Figure S10 NMR spectra of Au0^{+}-BF_4^{-}, Related to Figure 2.

(a) ^{1}H NMR and (b) ^{13}C NMR of **Au0**$^{+}$-BF_4^{-} in $CDCl_3$ at 20 °C.

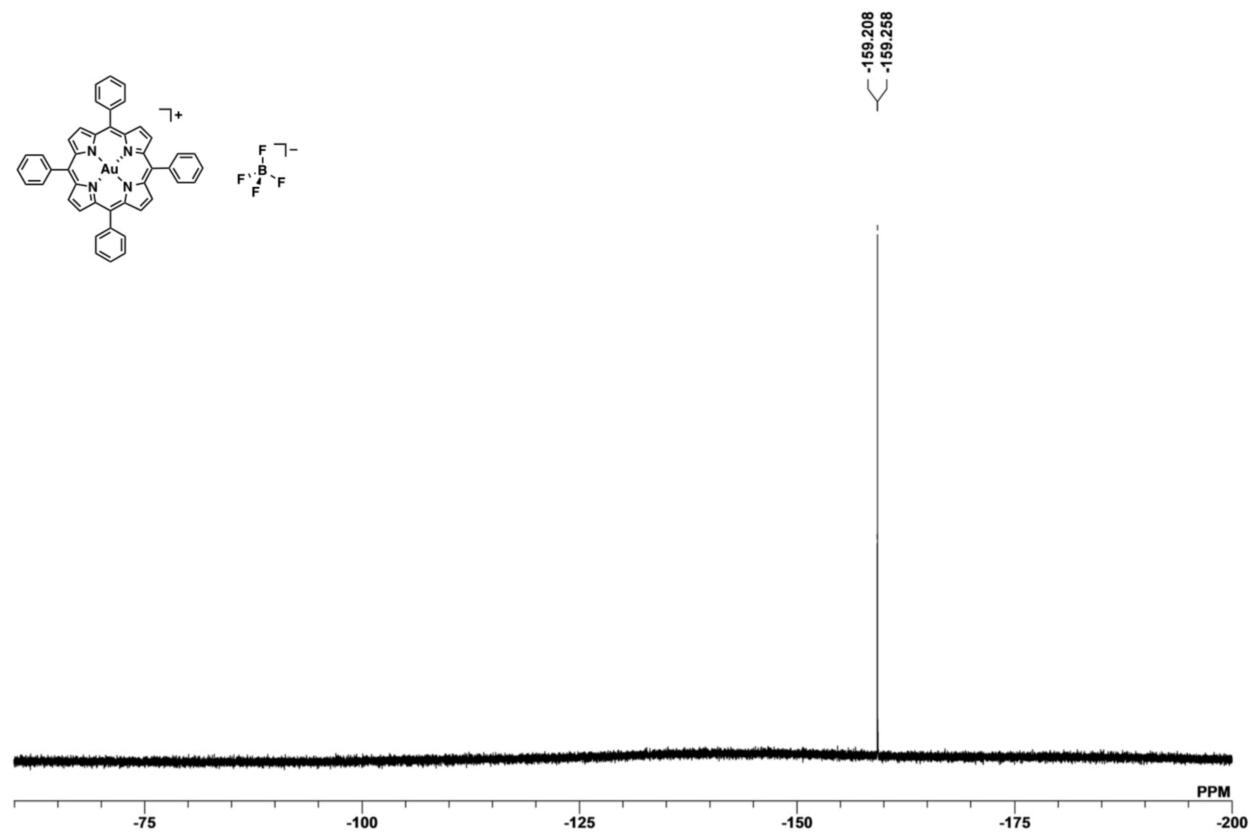

Figure S11 NMR spectrum of Au0^{+}-BF_4^{-}, Related to Figure 2.
^{19}F NMR spectrum of **Au0**$^{+}$-BF_4^{-} in $CDCl_3$ at 20 °C.

(a)

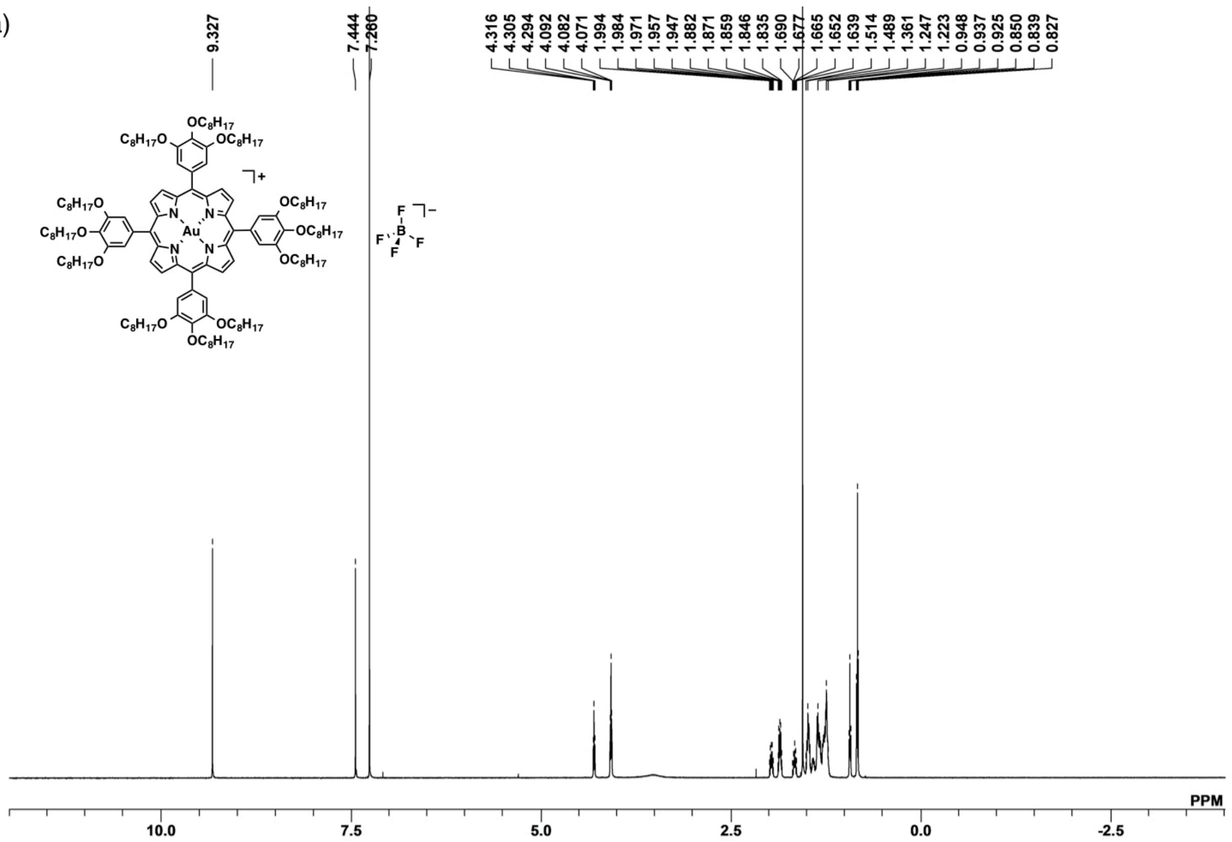

(b)

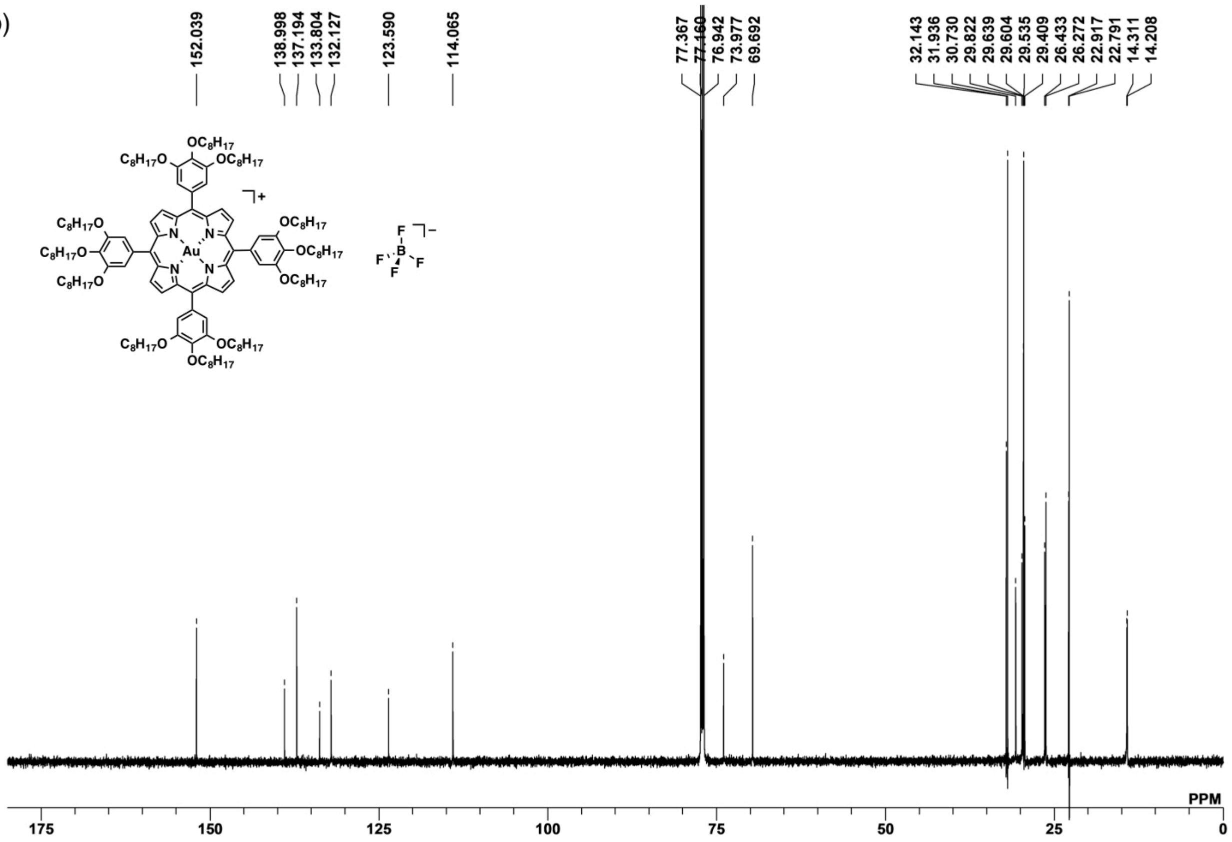

Figure S12 NMR spectra of Au8^{+}-BF$_4^{-}$, related to Figure 2.

(a) ^{1}H NMR and (b) ^{13}C NMR spectra of **Au8**$^{+}$-BF$_4^{-}$ in CDCl$_3$ at 20 °C.

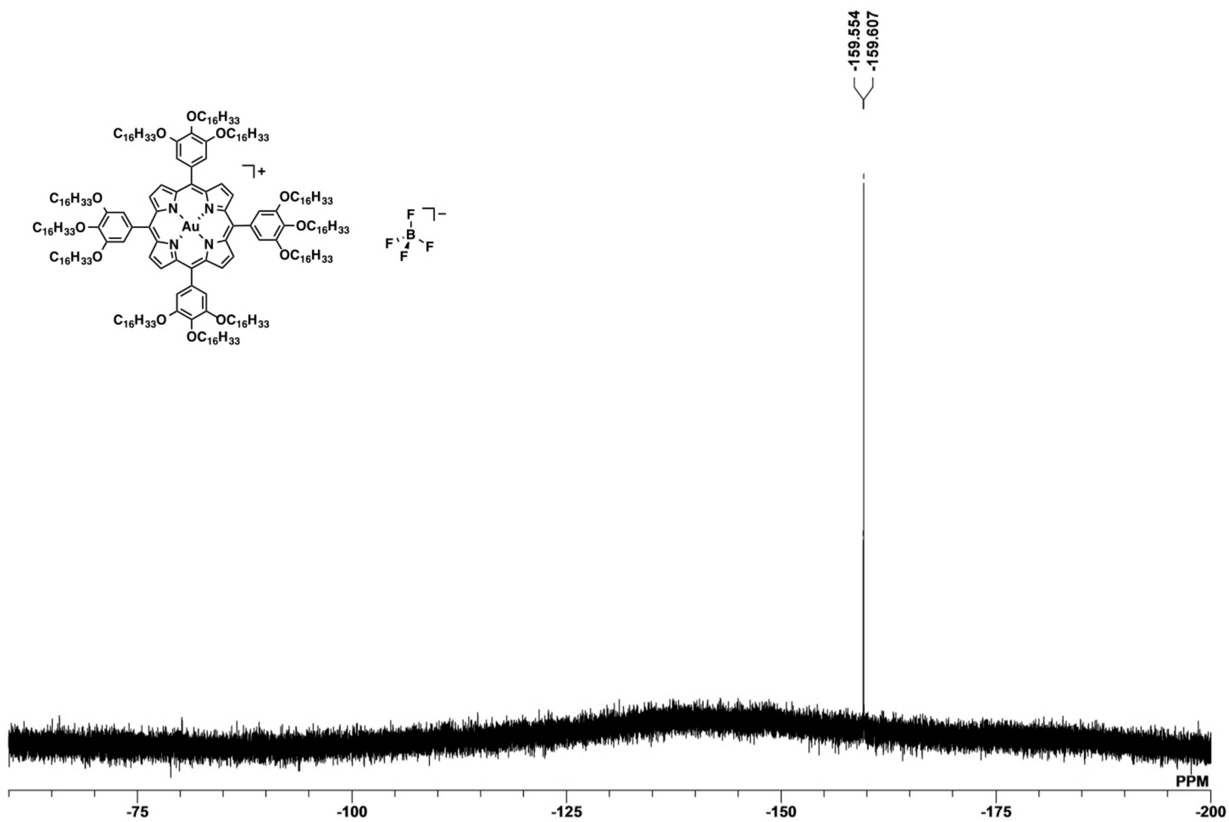

Figure S13 NMR spectrum of Au8^{+}-BF$_4^{-}$, Related to Figure 2.
^{19}F NMR spectrum of **Au8**$^{+}$-BF$_4^{-}$ in CDCl$_3$ at 20 °C.

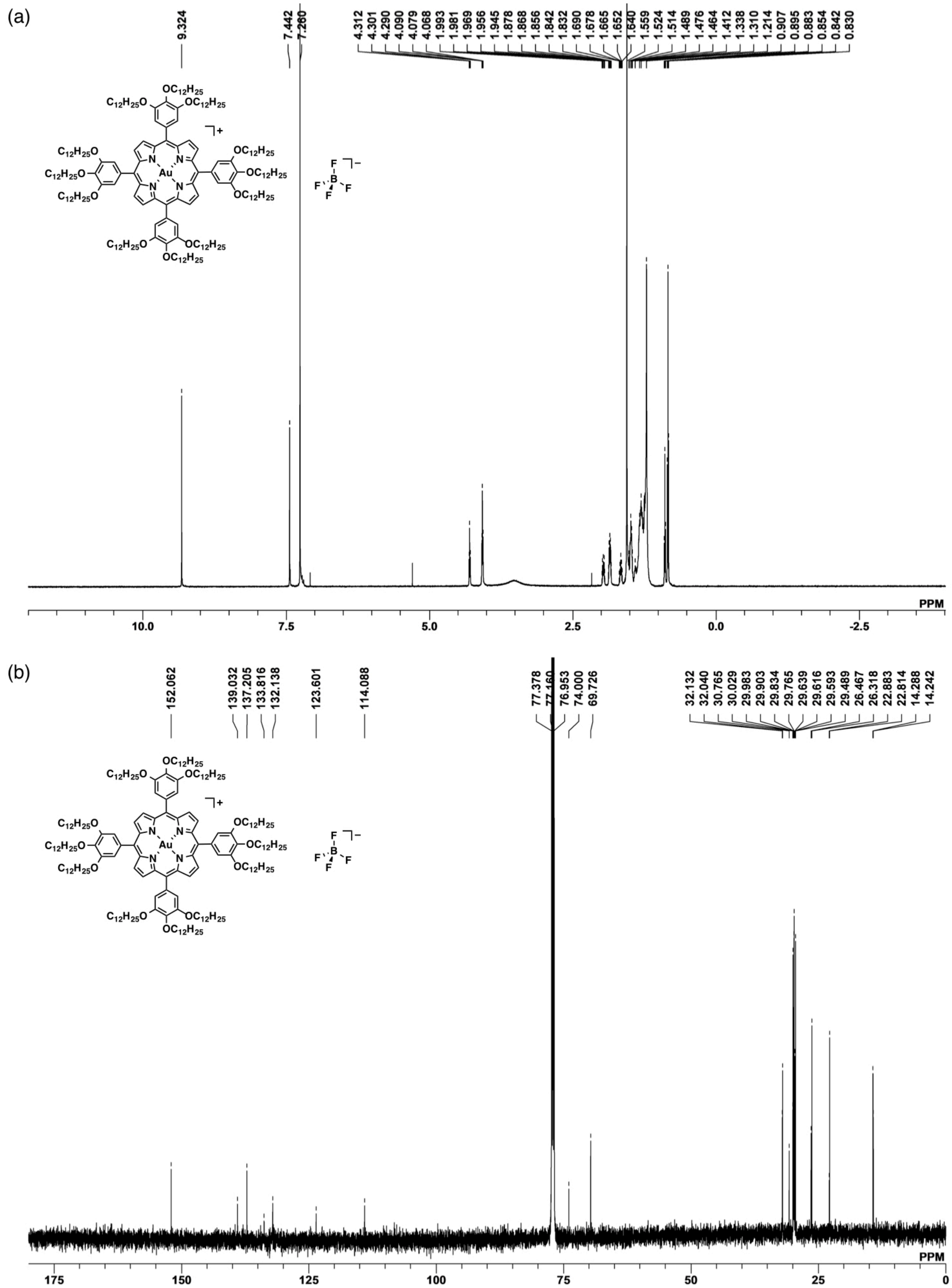

Figure S14 NMR spectra of Au12$^+$-BF$_4^-$, Related to Figure 2.
(a) ^{1}H NMR and (b) ^{13}C NMR spectra of **Au12**$^+$-BF$_4^-$ in CDCl$_3$ at 20 °C.

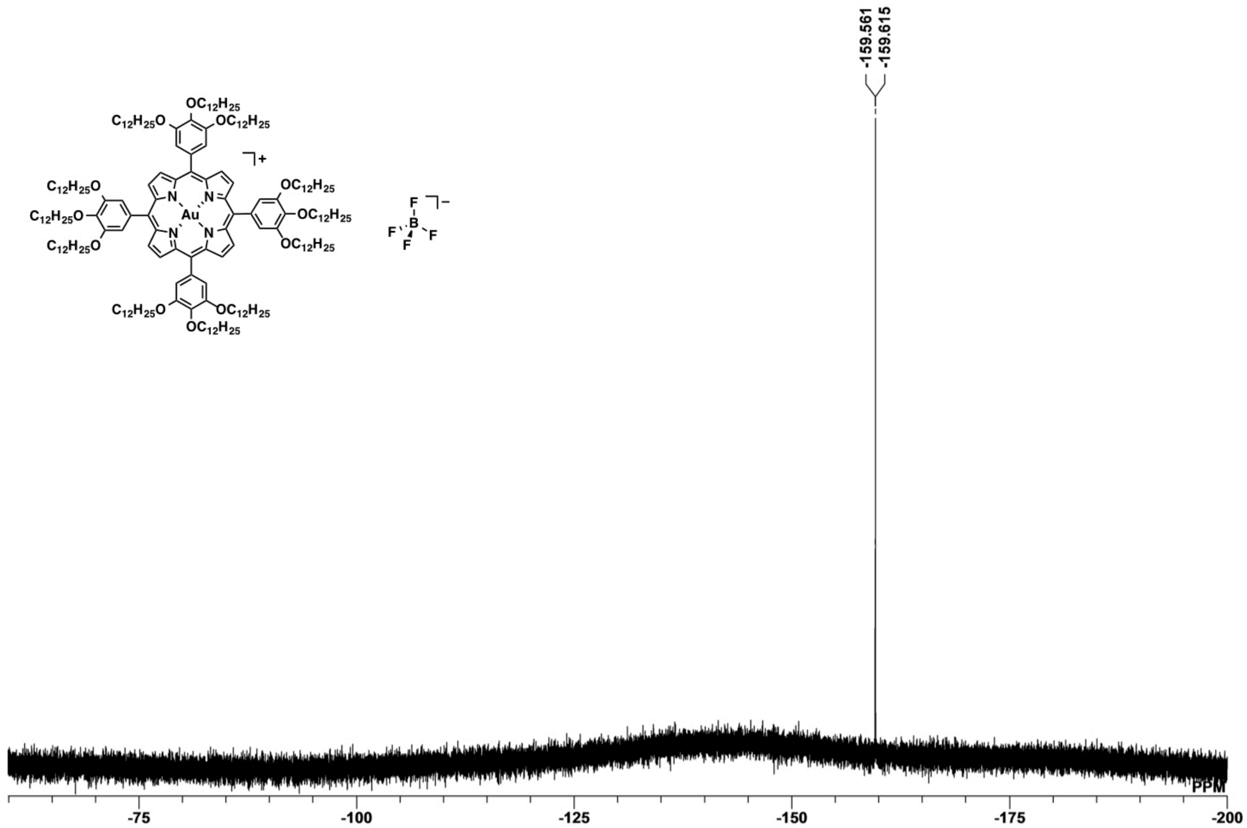

Figure S15 NMR spectrum of Au12^{+}-BF_4^{-}, Related to Figure 2.
^{19}F NMR spectrum of **Au12**$^{+}$-BF_4^{-} in $CDCl_3$ at 20 °C.

(a)

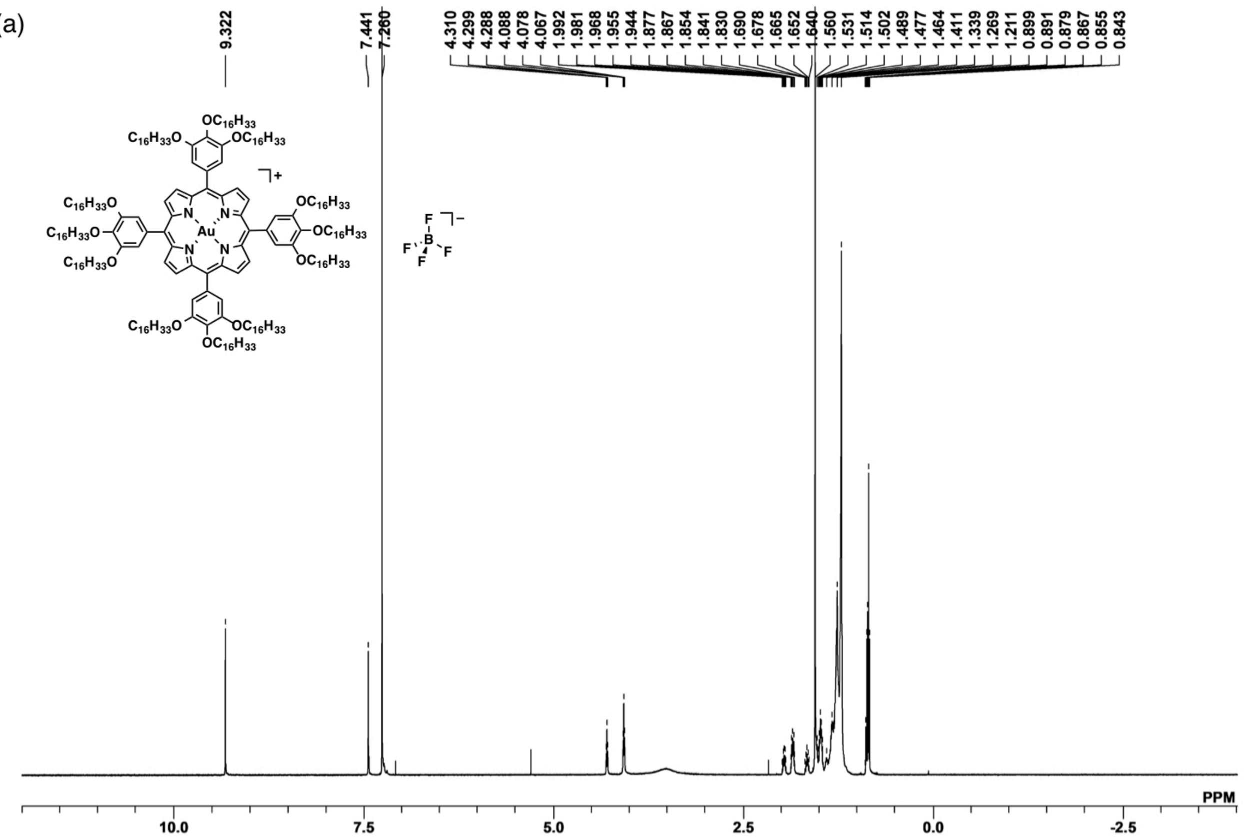

(b)

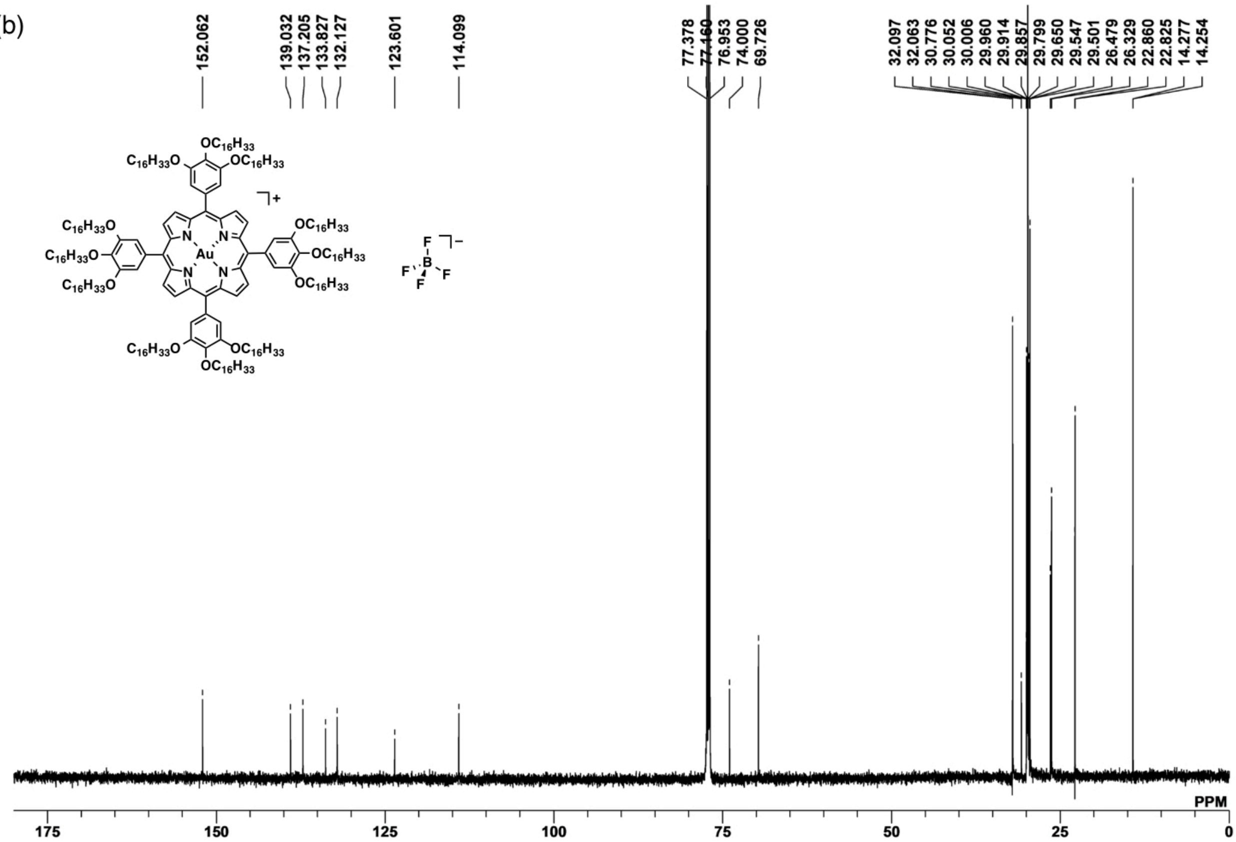

Figure S16 NMR spectra of Au16⁺-BF₄⁻, Related to Figure 2.

(a) ^{1}H NMR and (b) ^{13}C NMR spectra of **Au16**$^{+}$-BF_4^{-} in $CDCl_3$ at 20 °C.

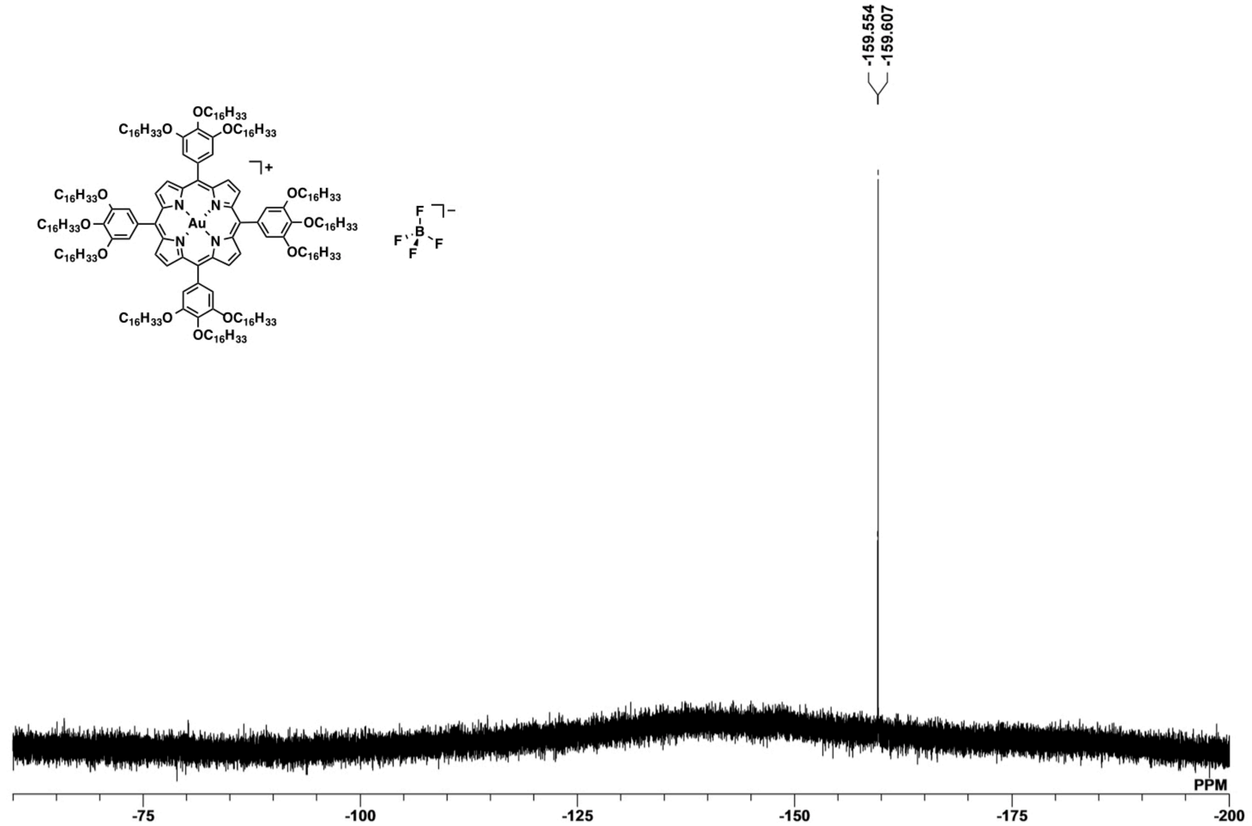

Figure S17 NMR spectrum of Au16^{+}-BF_4^{-}, Related to Figure 2.

^{19}F NMR spectrum of **Au16**$^{+}$-BF_4^{-} in $CDCl_3$ at 20 °C.

(a)

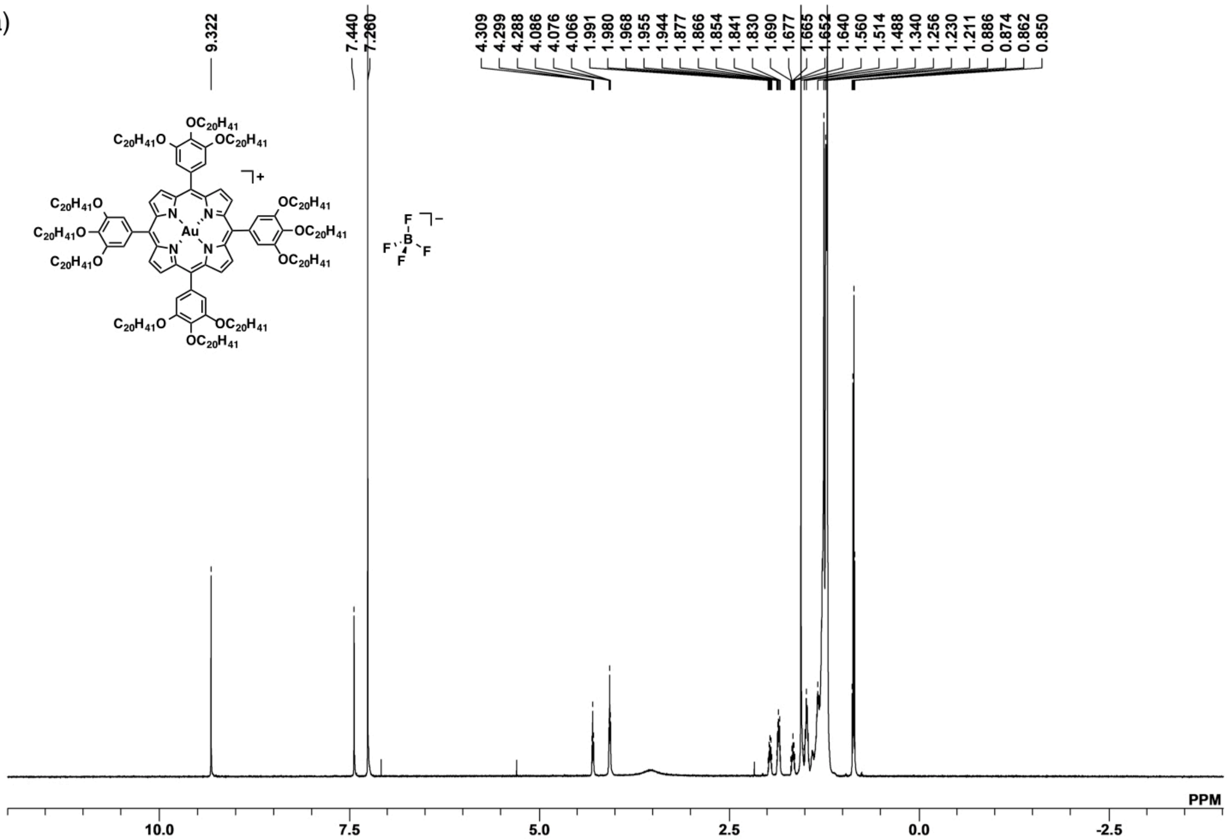

(b)

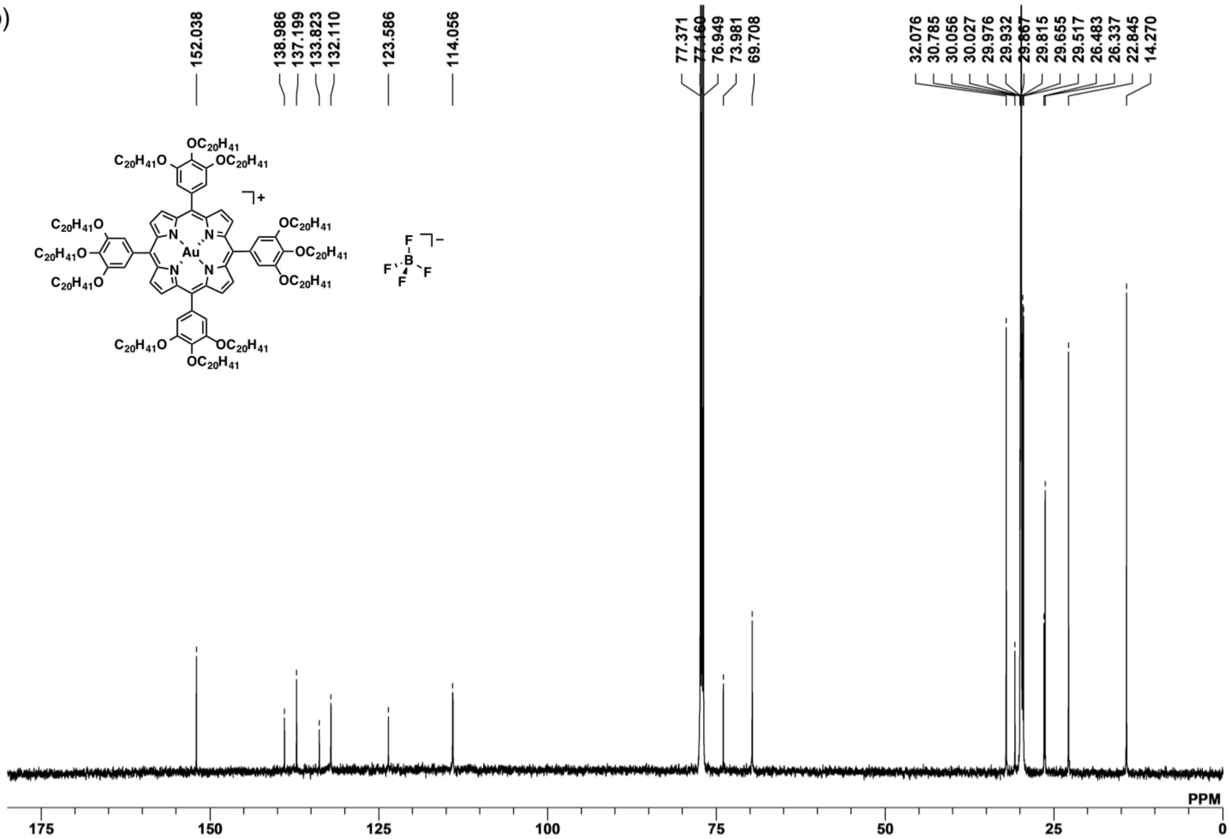

Figure S18 NMR spectra of Au20^{+}-BF$_4^{-}$, Related to Figure 2.

(a) ^{1}H NMR and (b) ^{13}C NMR spectra of **Au20**$^{+}$-BF$_4^{-}$ in CDCl$_3$ at 20 °C.

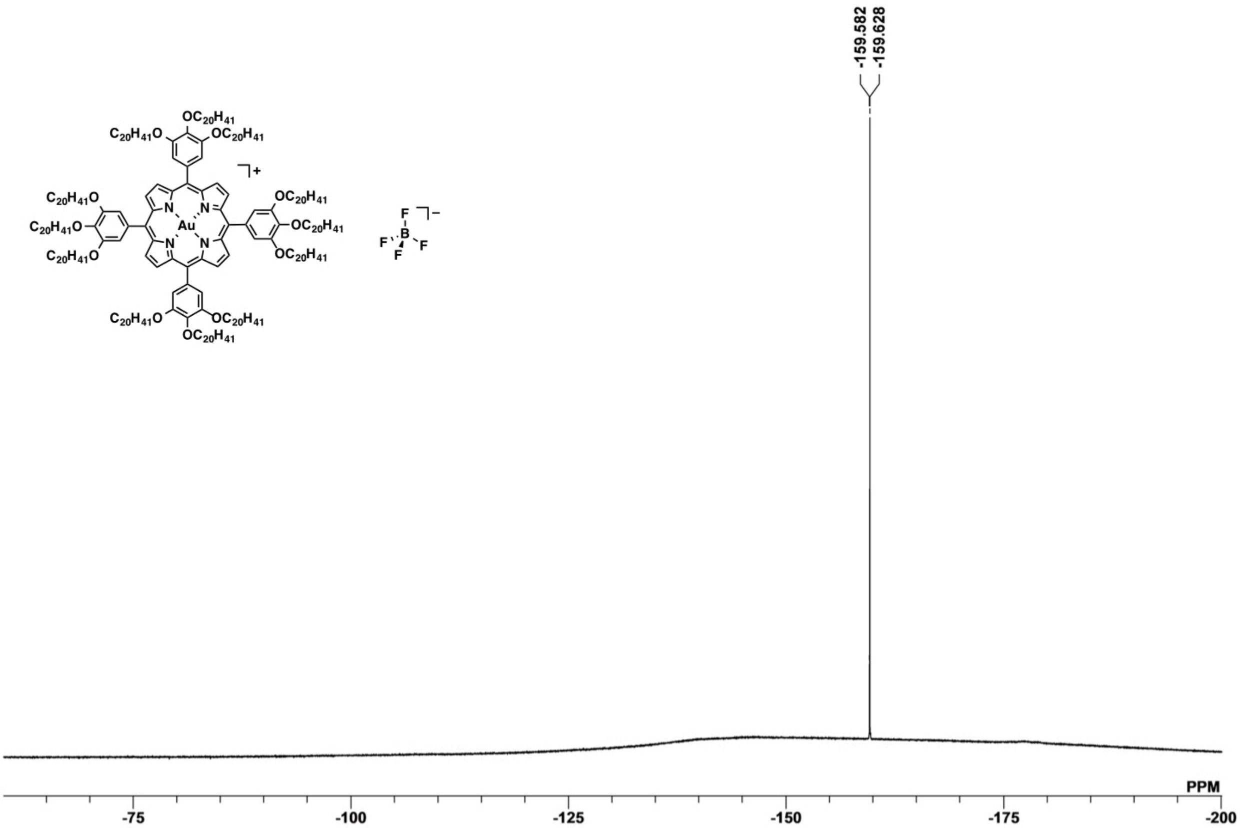

Figure S19 NMR spectrum of Au20^{+}-BF$_4^{-}$, Related to Figure 2.

^{19}F NMR spectrum of **Au20**$^{+}$-BF$_4^{-}$ in CDCl$_3$ at 20 °C.

(a)

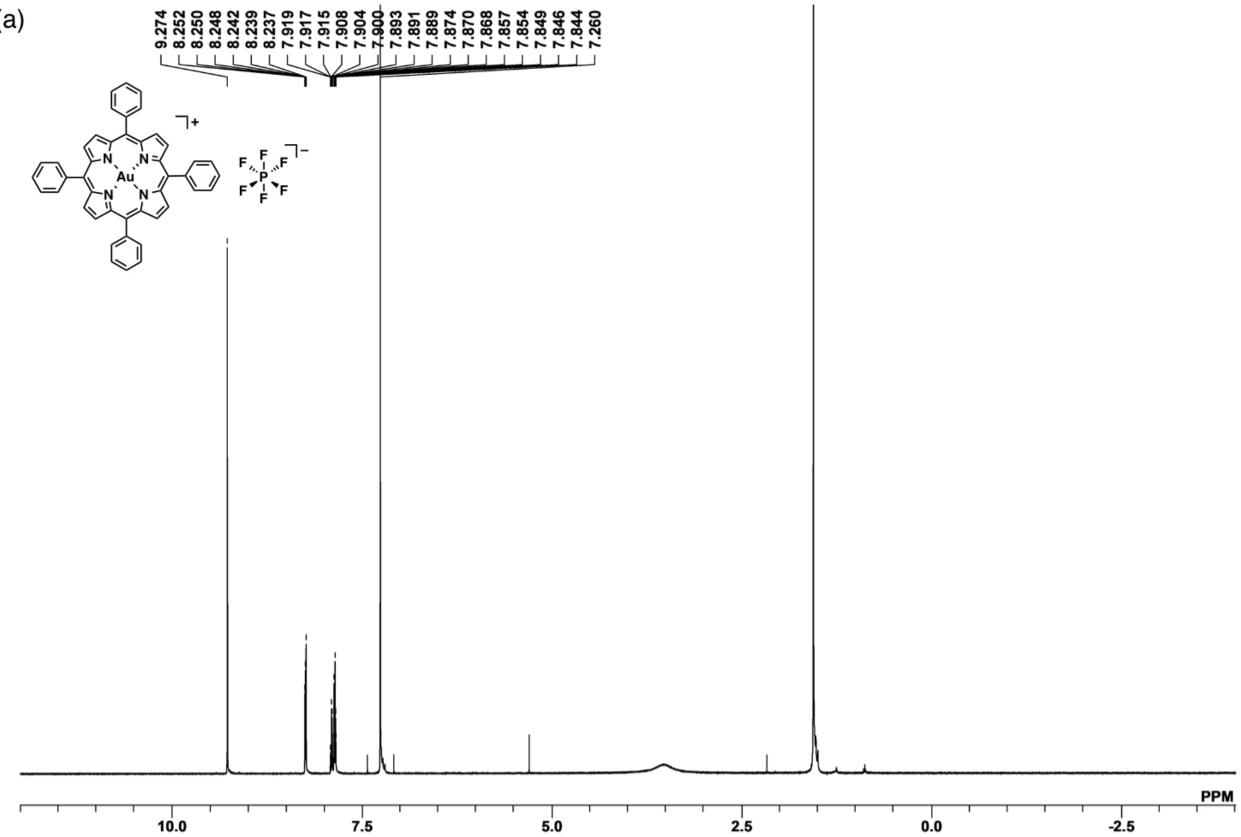

(b)

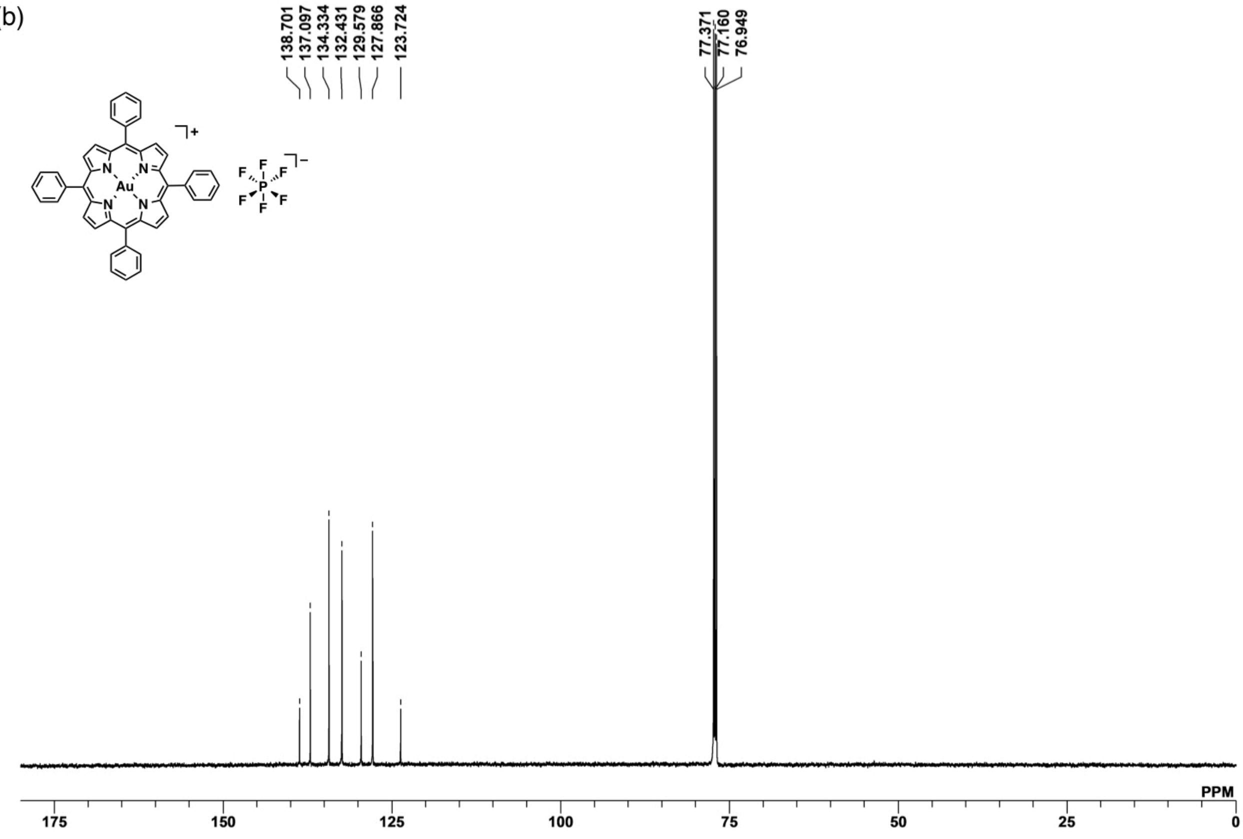

Figure S20 NMR spectra of Au0$^+$-PF$_6^-$, Related to Figure 2.
(a) ^{1}H NMR and (b) ^{13}C NMR spectra of **Au0**$^+$-PF$_6^-$ in CDCl$_3$ at 20 °C.

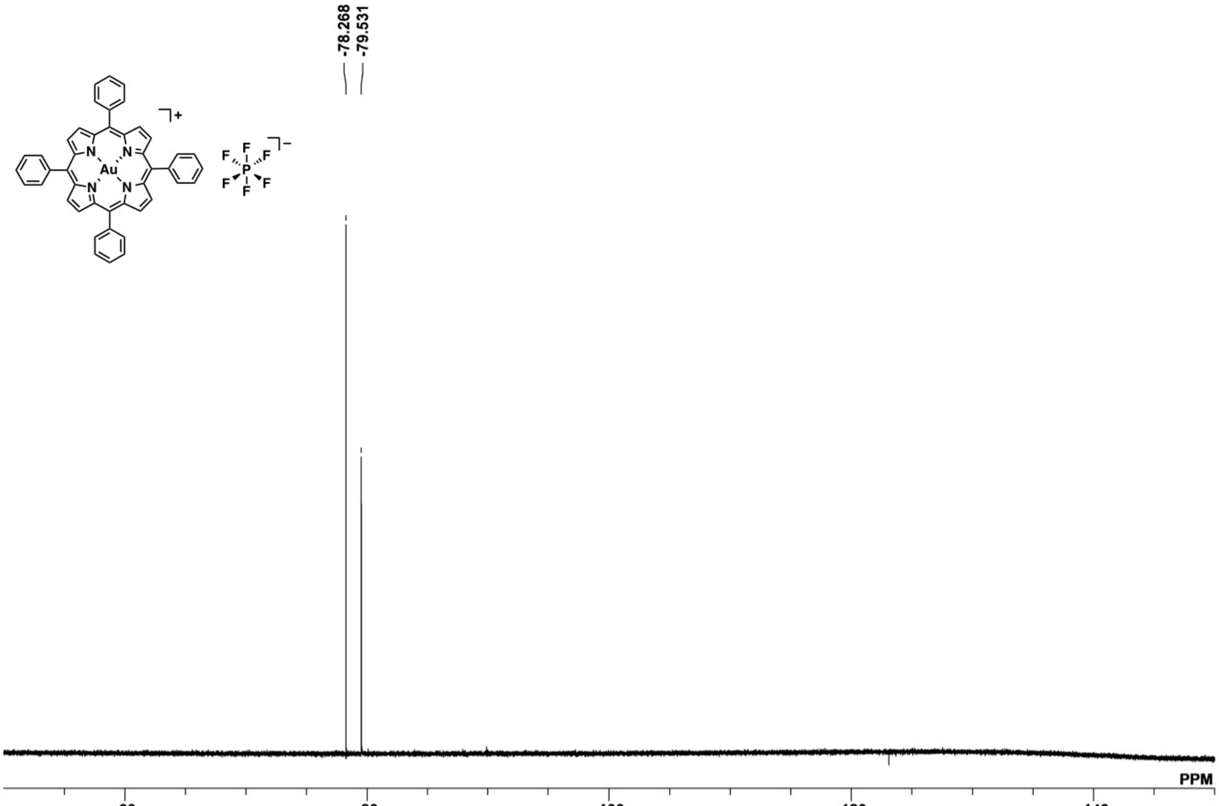

Figure S21 NMR spectrum of Au0^{+}-PF$_6^{-}$, Related to Figure 2.
^{19}F NMR spectrum of **Au0**$^{+}$-PF$_6^{-}$ in $CDCl_3$ at 20 °C.

(a)

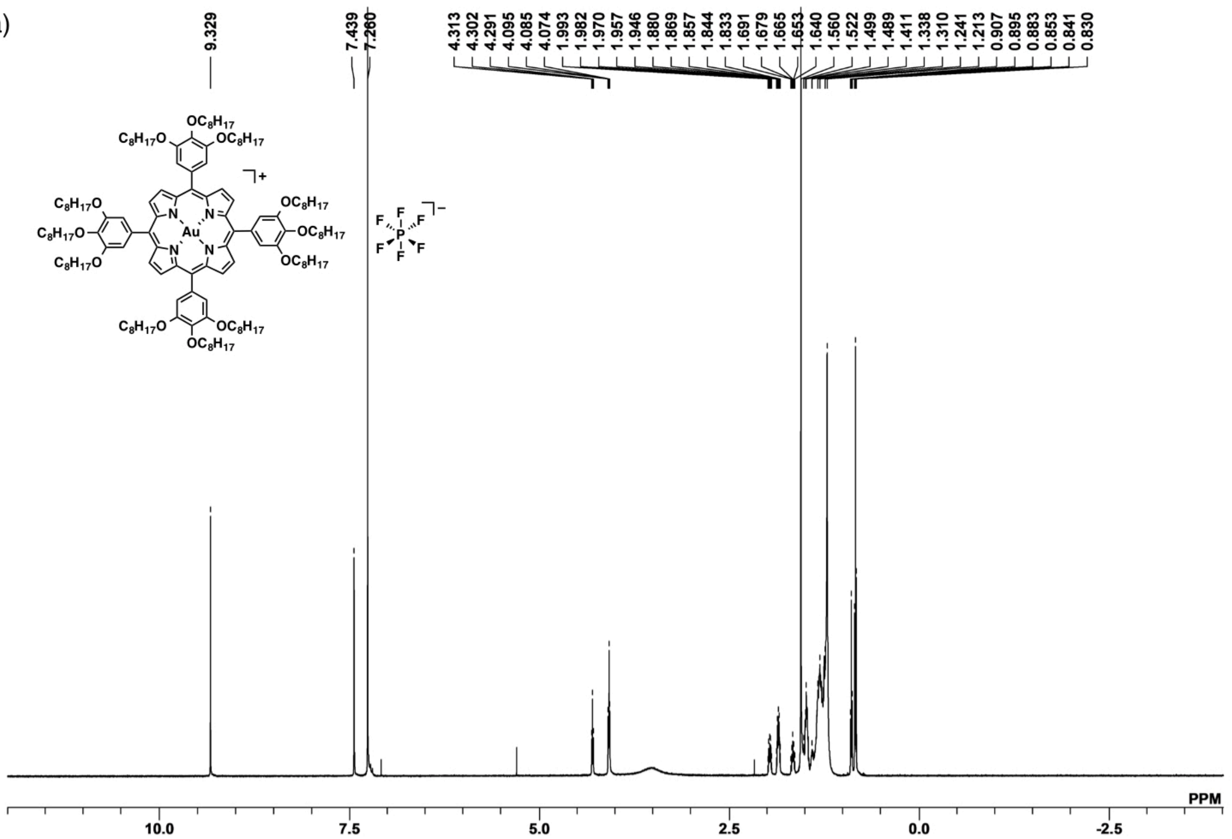

(b)

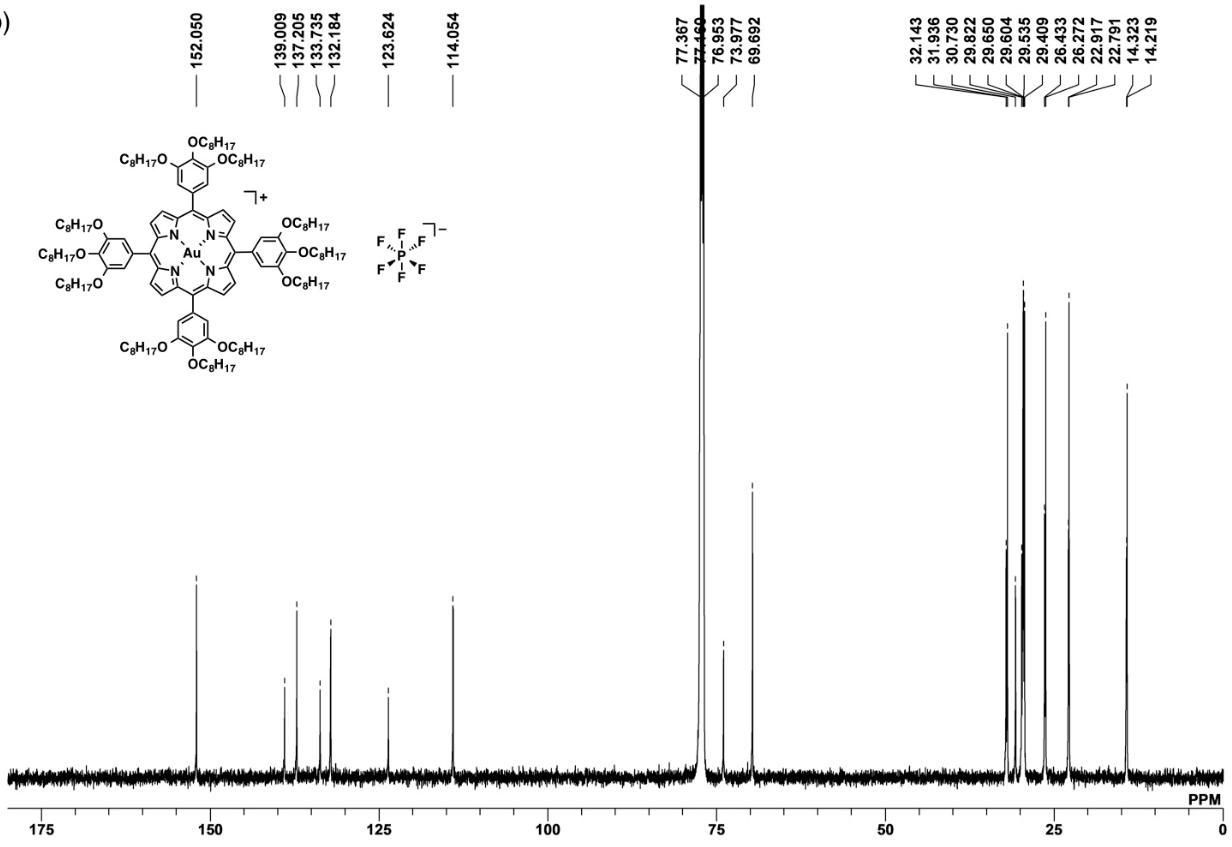

Figure S22 NMR spectra of Au8^{+}-PF_6^{-}, Related to Figure 2.
(a) ^{1}H NMR and (b) ^{13}C NMR spectra of **Au8**$^{+}$-PF_6^{-} in $CDCl_3$ at 20 °C.

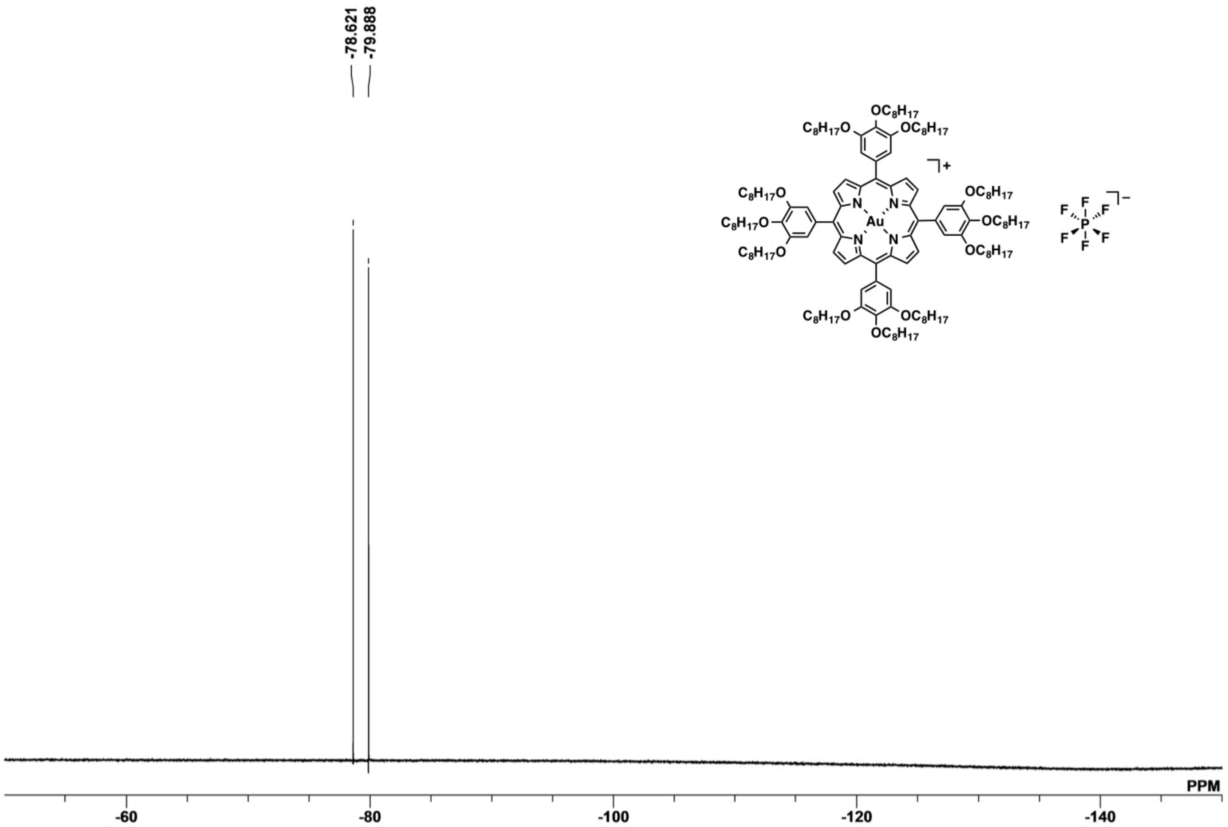

Figure S23 NMR spectrum of Au8^{+}-PF$_6^{-}$, Related to Figure 2.
^{19}F NMR spectrum of **Au8**$^{+}$-PF$_6^{-}$ in $CDCl_3$ at 20 °C.

(a)

(b)

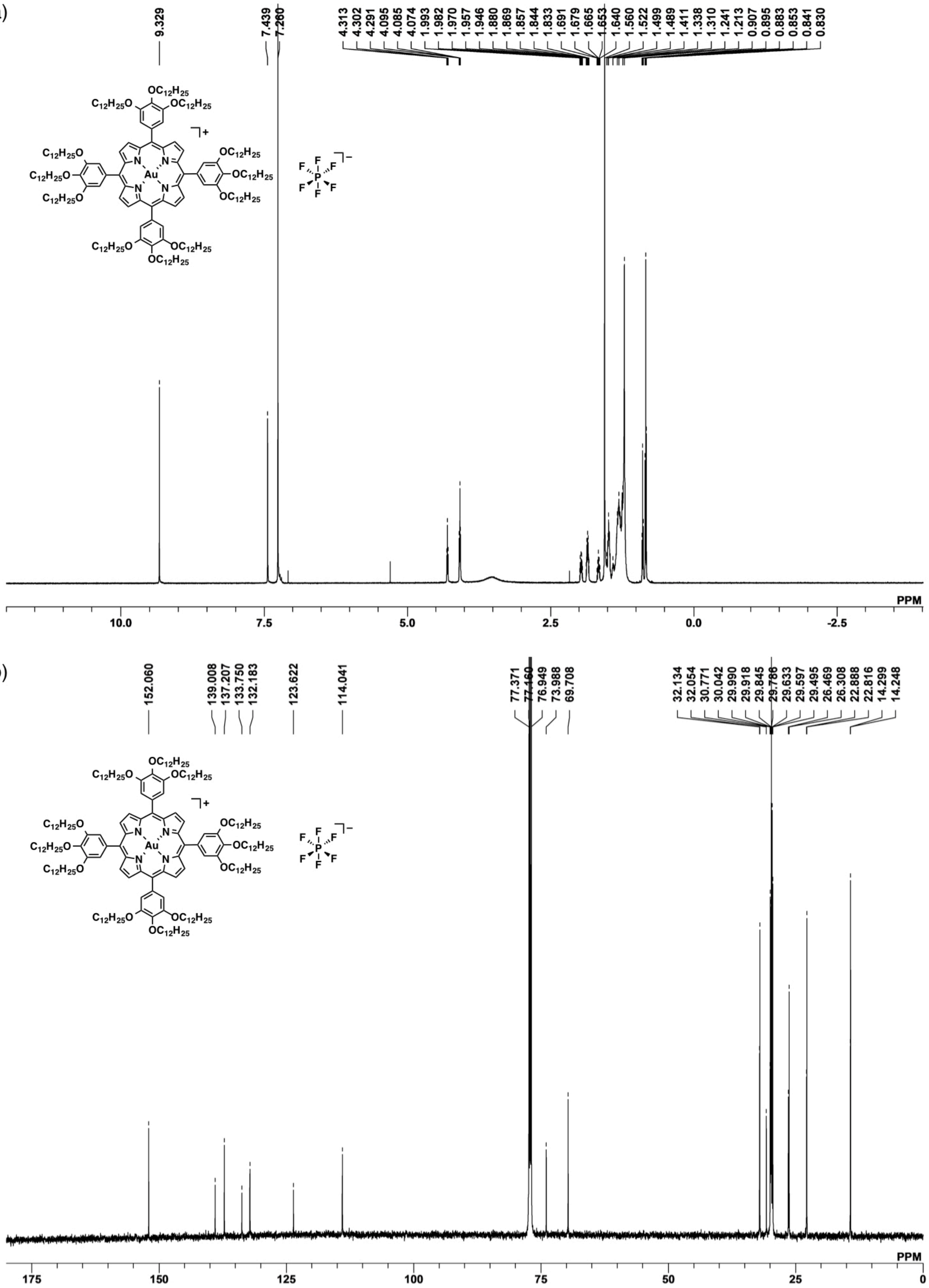

Figure S24 NMR spectra of Au12^{+}-PF$_6^{-}$, Related to Figure 2.
(a) ^{1}H NMR and (b) ^{13}C NMR spectra of **Au12**$^{+}$-PF$_6^{-}$ in CDCl$_3$ at 20 °C.

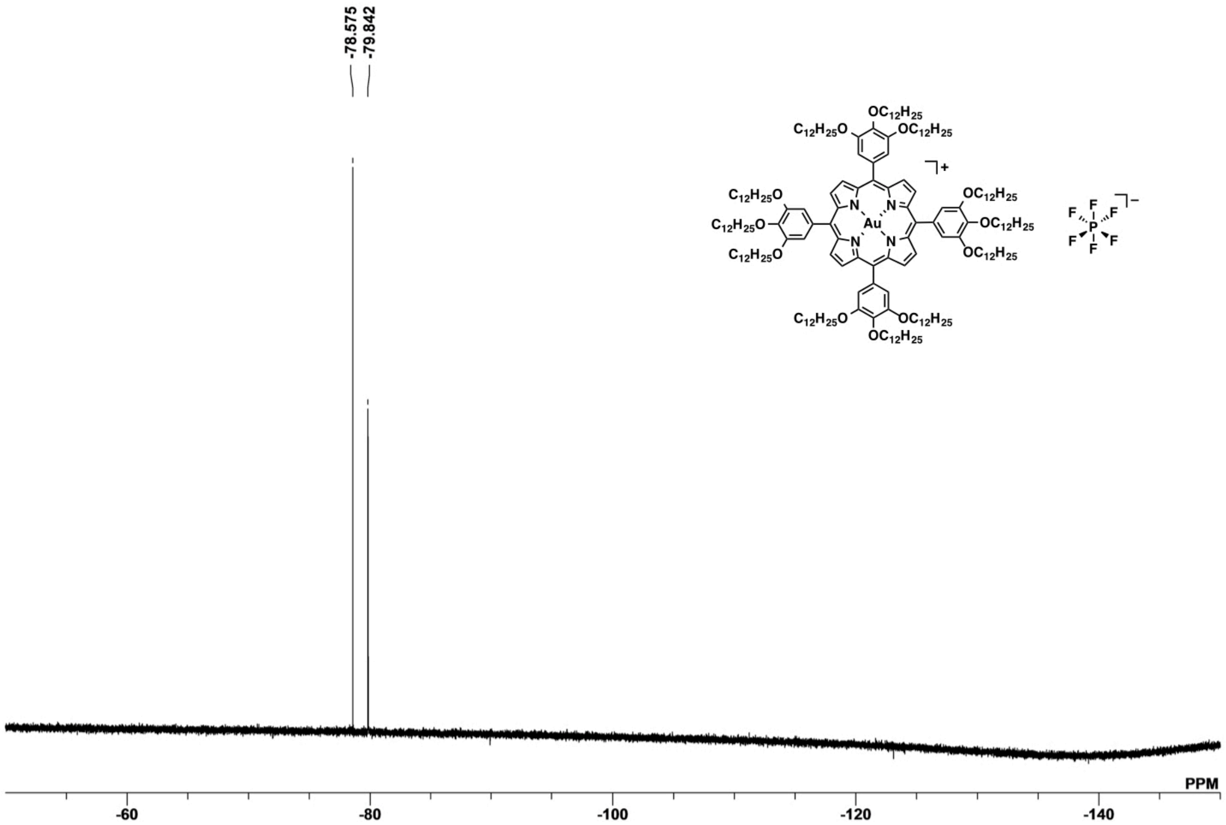

Figure S25 NMR spectrum of Au12^{+}-PF$_6^{-}$, Related to Figure 2.
^{19}F NMR spectrum of **Au12**$^{+}$-PF$_6^{-}$ in $CDCl_3$ at 20 °C.

(a)

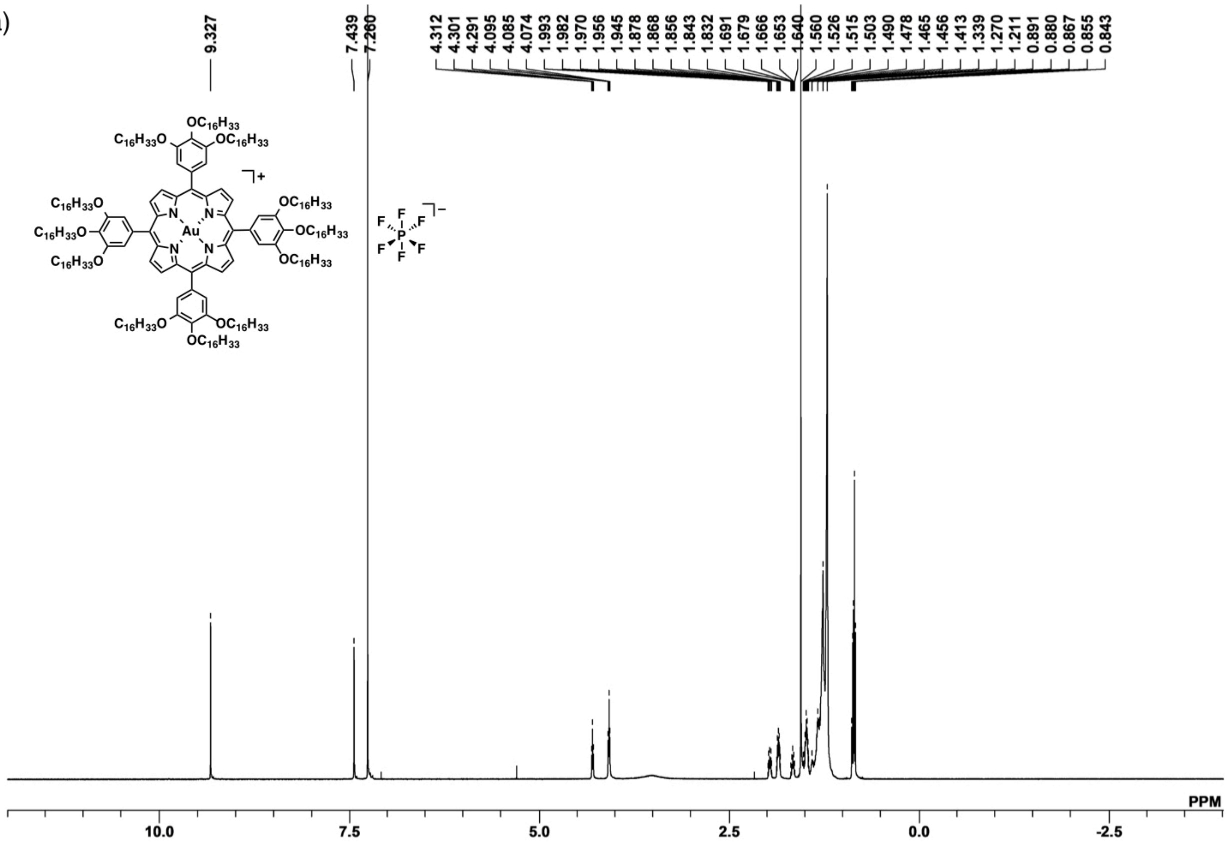

(b)

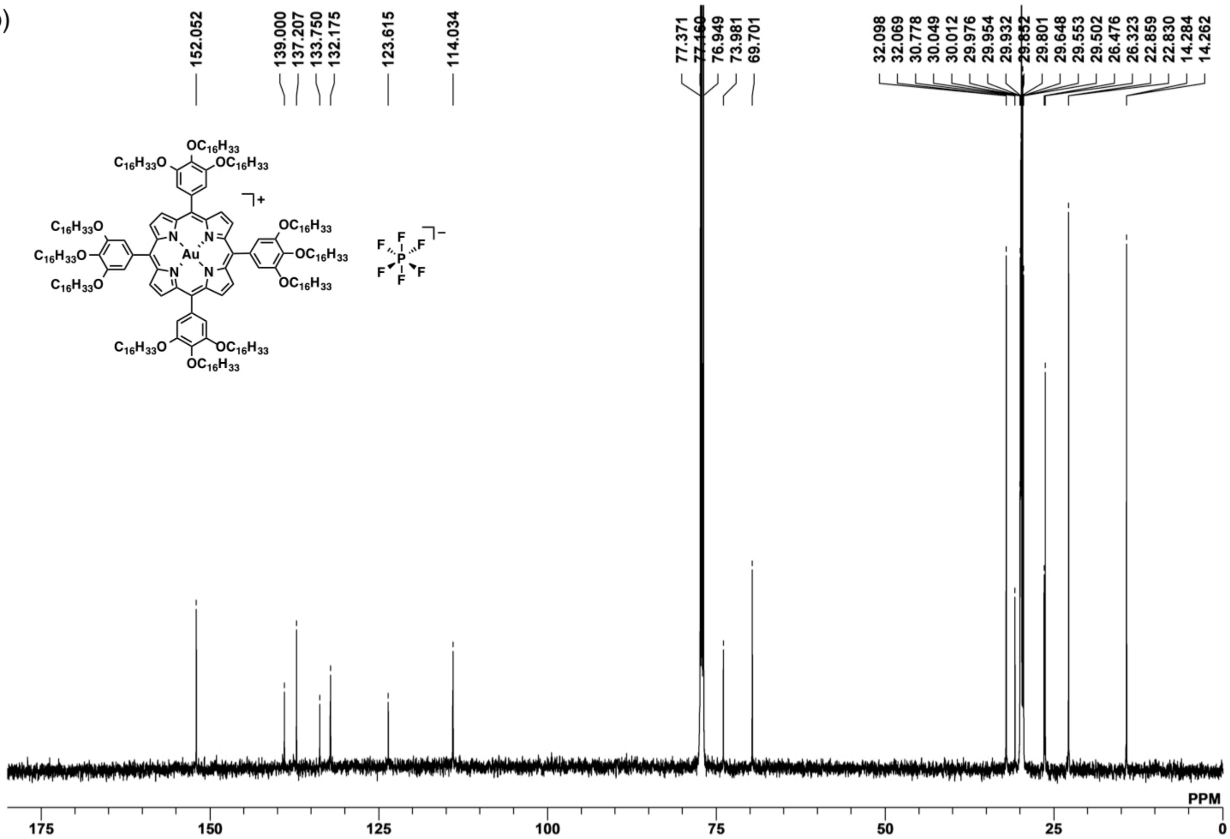

Figure S26 NMR spectra of Au16^{+}-PF$_6^{-}$, Related to Figure 2.
(a) ^{1}H NMR and (b) ^{13}C NMR spectra of **Au16**$^{+}$-PF$_6^{-}$ in CDCl$_3$ at 20 °C.

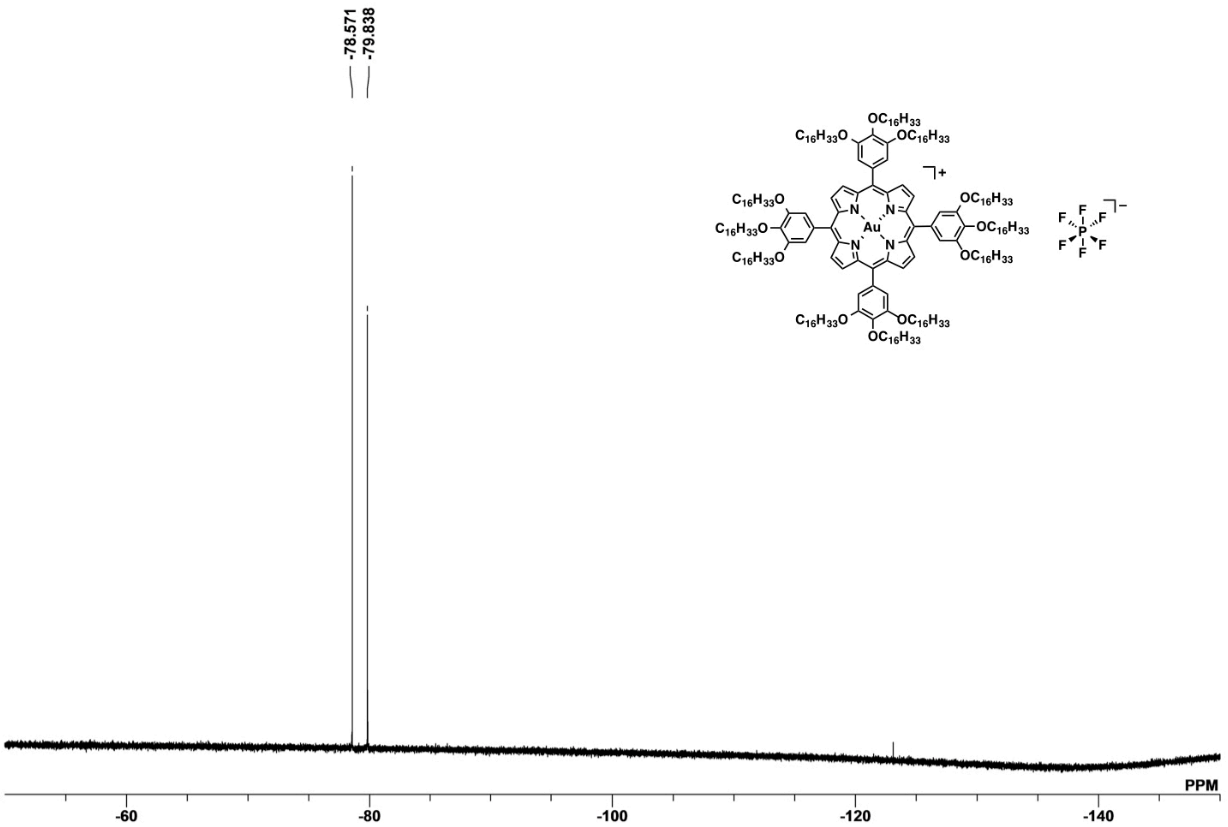

Figure S27 NMR spectrum of Au16$^+$-PF_6^-, Related to Figure 2.

^{19}F NMR spectrum of **Au16**$^+$-PF_6^- in $CDCl_3$ at 20 °C.

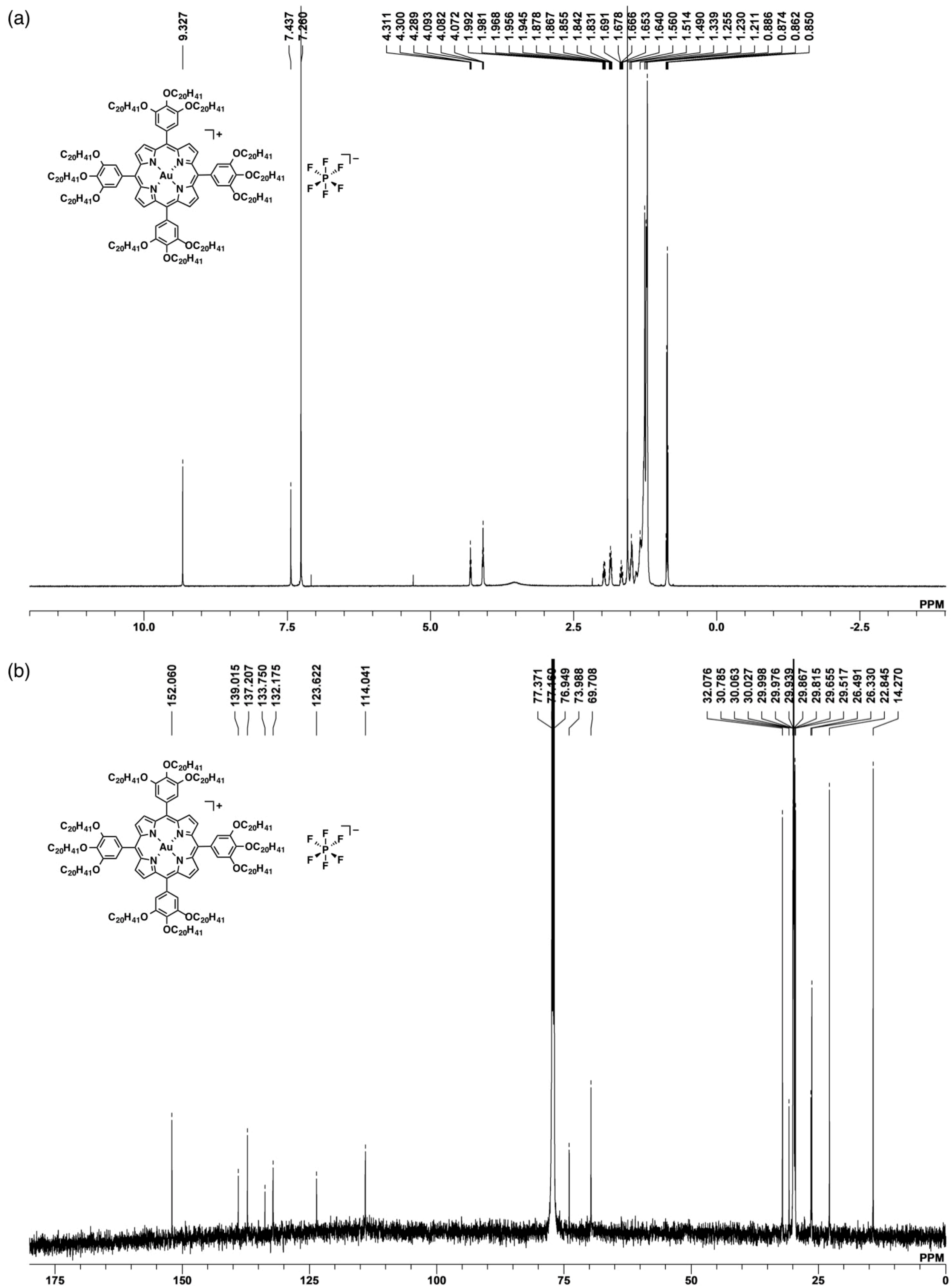

Figure S28 NMR spectra of Au20^{+}-PF$_6^{-}$, Related to Figure 2.
^{19}F NMR spectra of **Au20**$^{+}$-PF$_6^{-}$ in CDCl$_3$ at 20 °C.

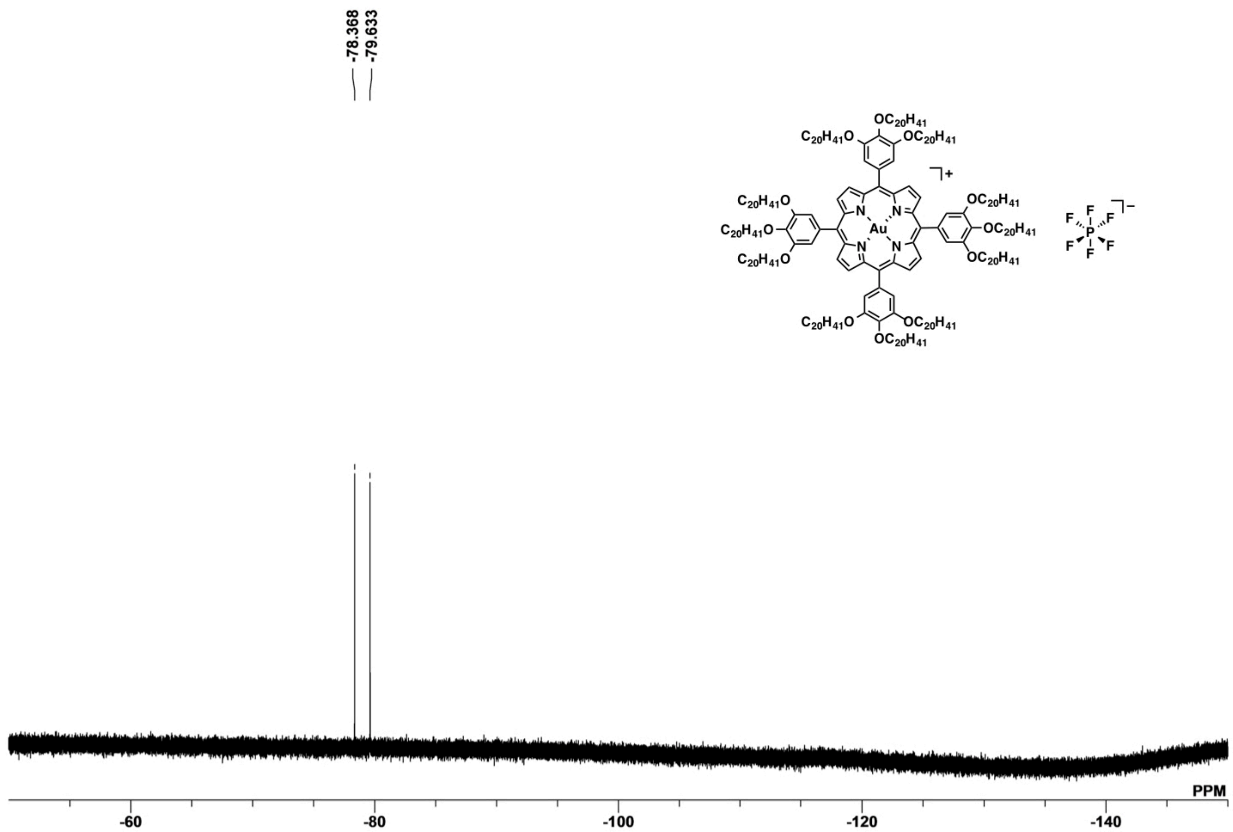

Figure S29 NMR spectrum of Au20^{+}-PF_6^{-}, Related to Figure 2.

^{19}F NMR spectrum of **Au20**$^{+}$-PF_6^{-} in $CDCl_3$ at 20 °C.

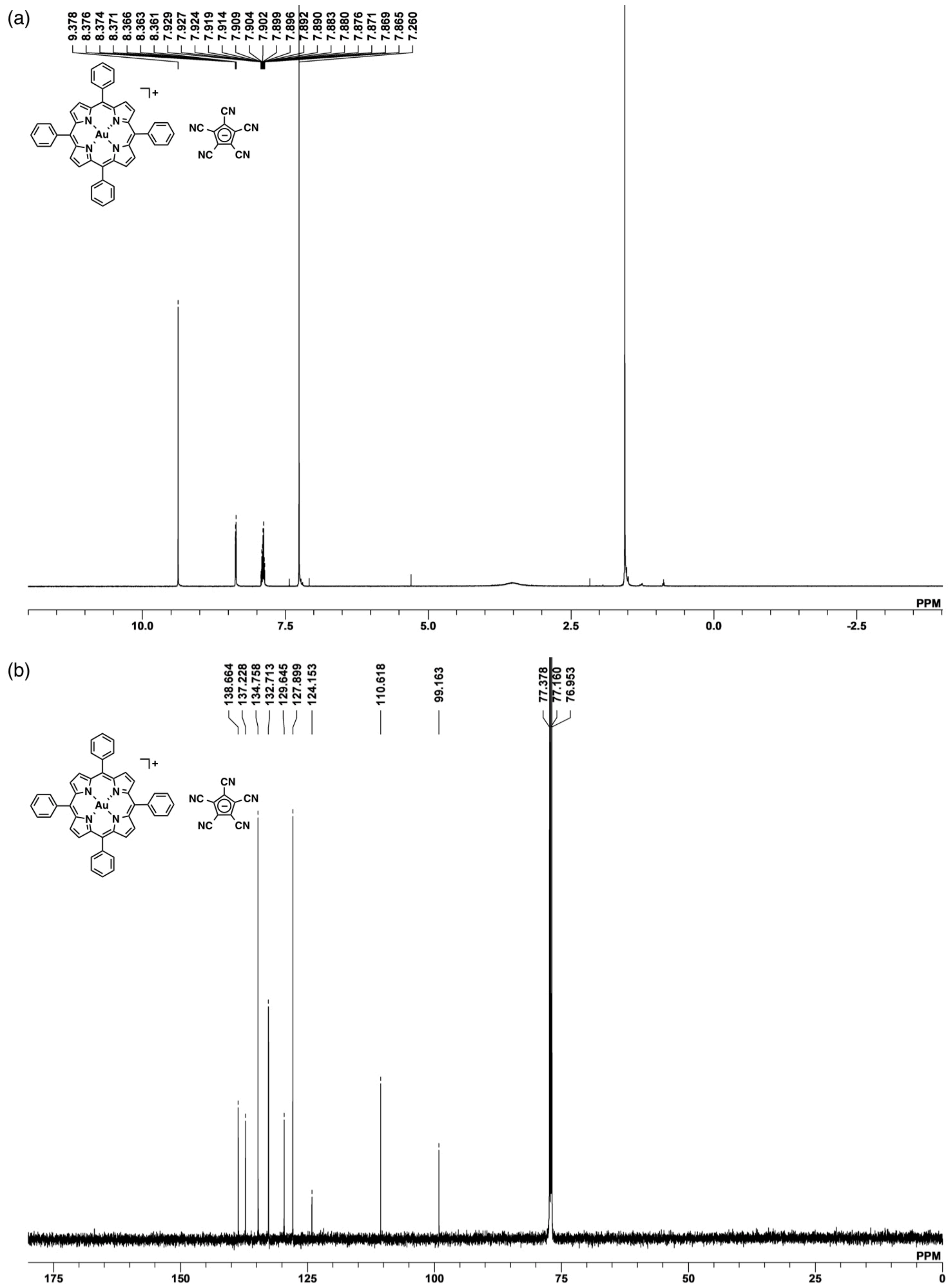

Figure S30 NMR spectra of Au0$^+$-PCCp$^-$, Related to Figure 2.
(a) ^{1}H NMR and (b) ^{13}C NMR spectra of **Au0**$^+$-PCCp$^-$ in $CDCl_3$ at 20 °C.

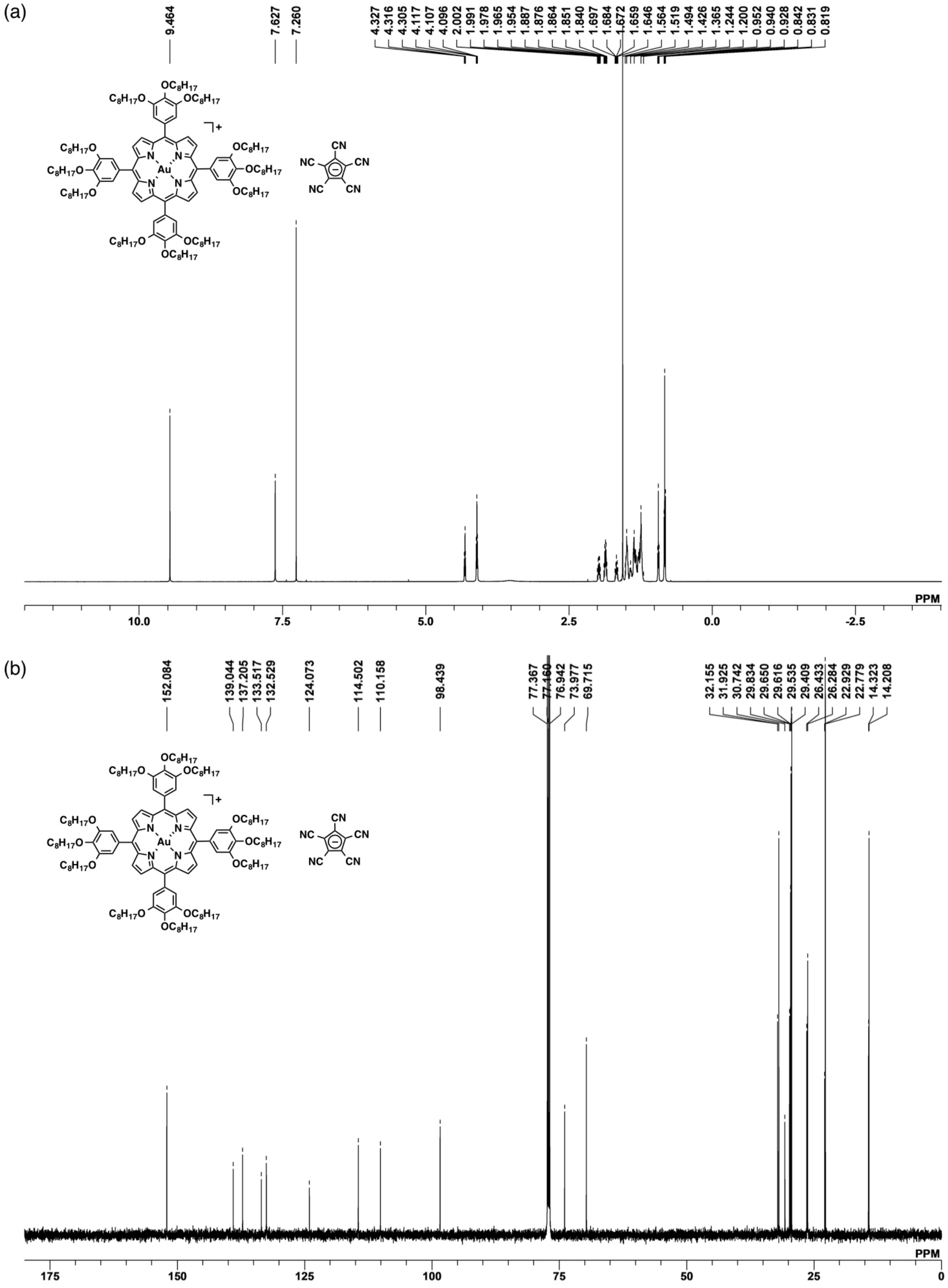

Figure S31 NMR spectra of Au8$^+$-PCCp$^-$, Related to Figure 2.

(a) ^{1}H NMR and (b) ^{13}C NMR spectra of **Au8**$^+$-PCCp$^-$ in $CDCl_3$ at 20 °C.

(a)

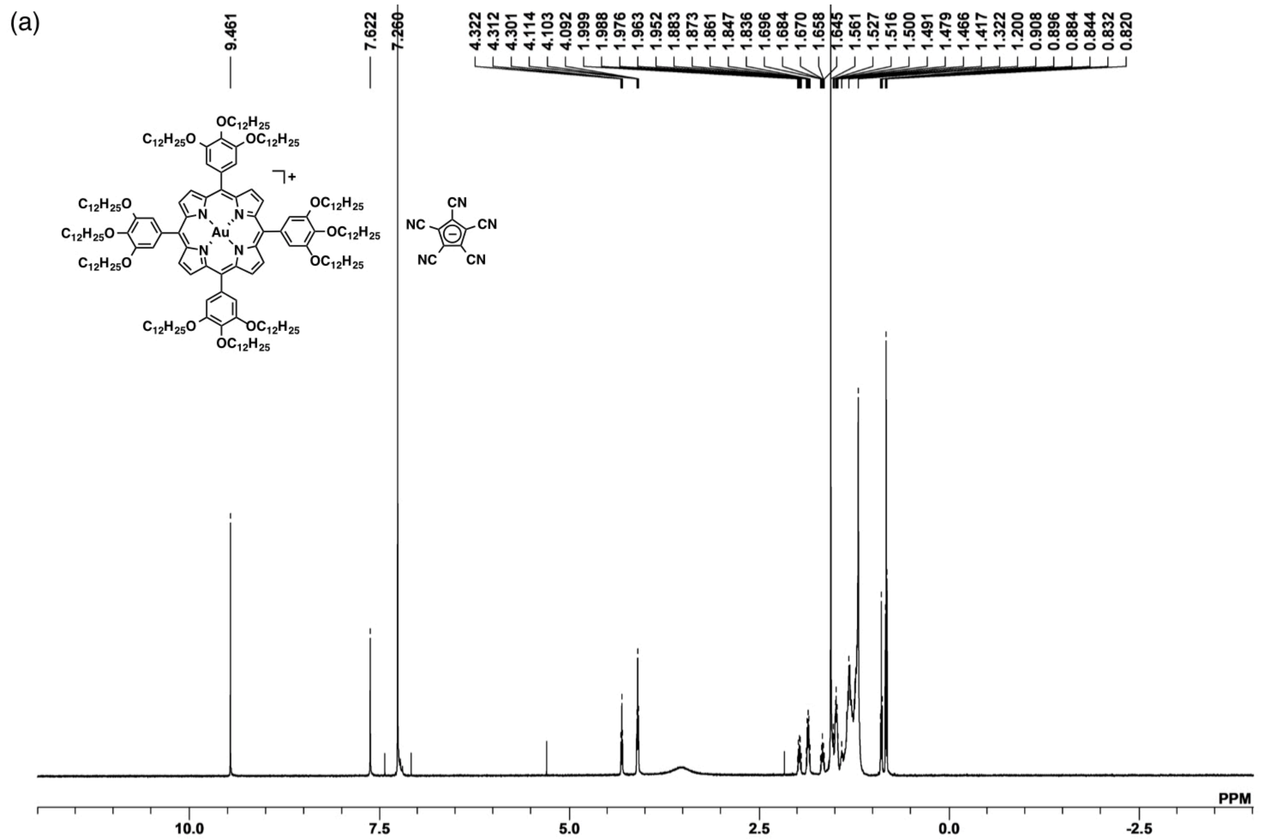

(b)

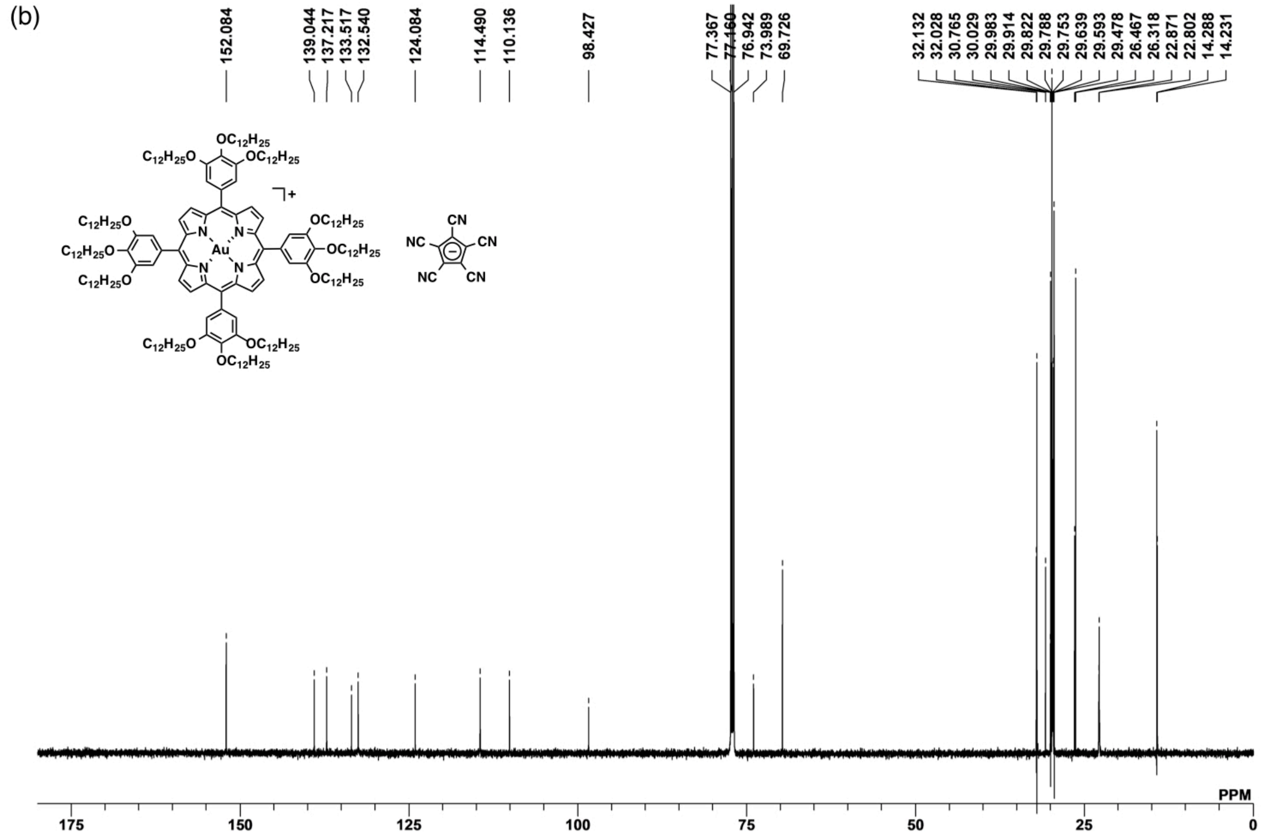

Figure S32 NMR spectra of Au12^{+}-PCCp^{-}, Related to Figure 2.
(a) ^{1}H NMR and (b) ^{13}C NMR spectra of **Au12**$^{+}$-PCCp^{-} in $CDCl_3$ at 20 °C.

(a)

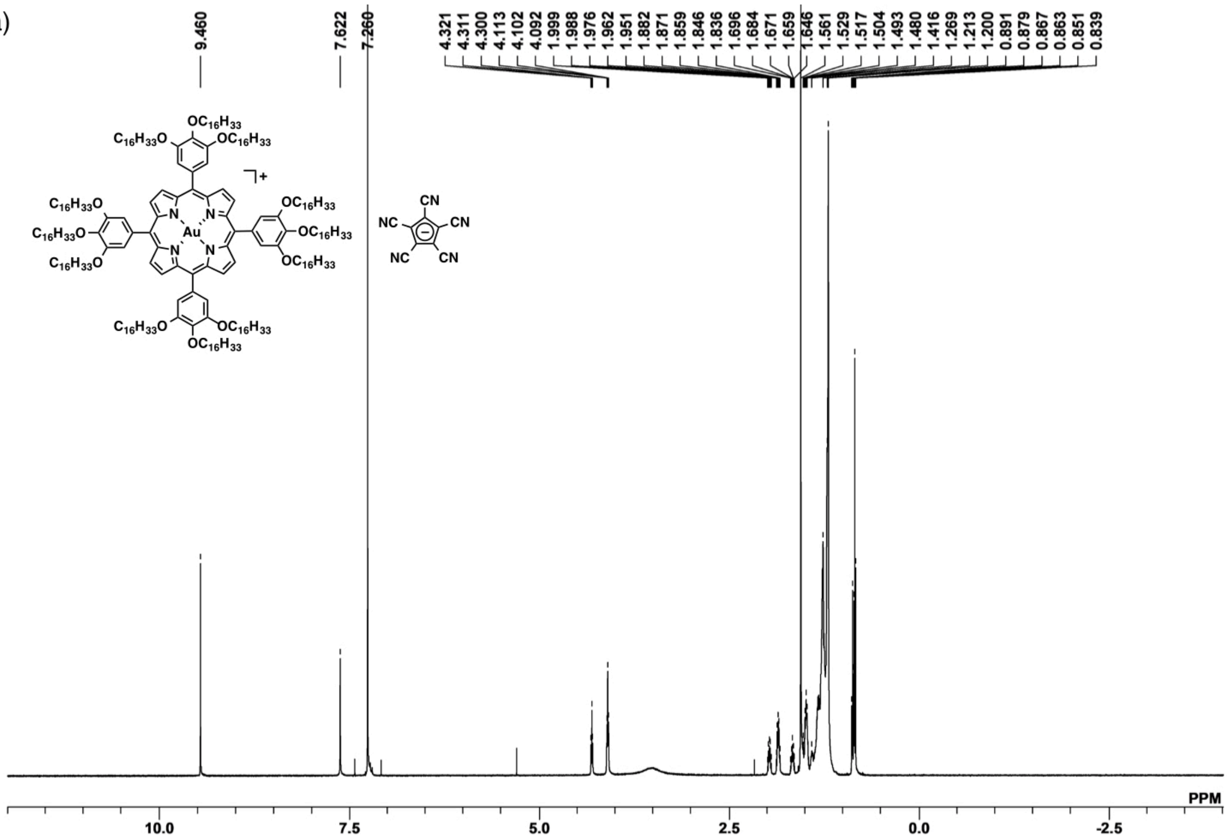

(b)

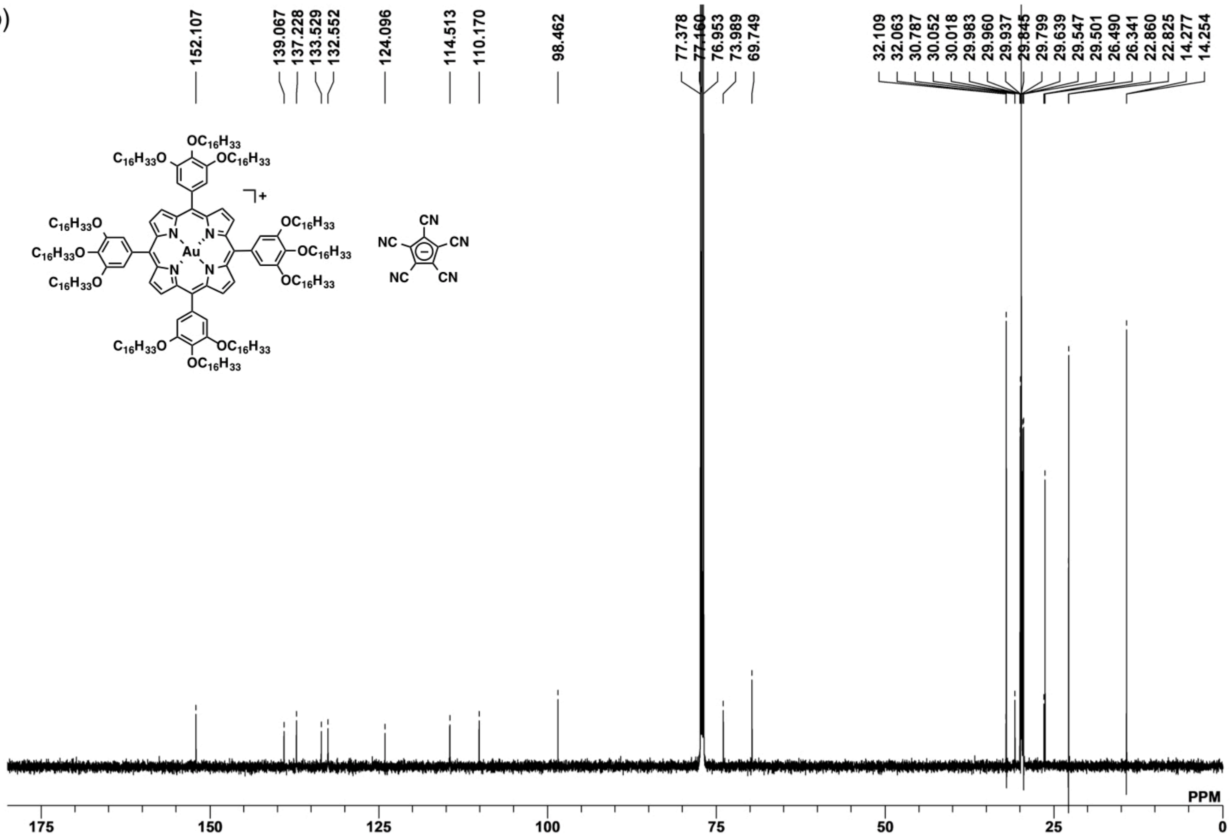

Figure S33 NMR spectra of Au16^{+}-PCCp^{-}, Related to Figure 2.
(a) ^{1}H NMR and (b) ^{13}C NMR spectra of **Au16**$^{+}$-PCCp^{-} in $CDCl_3$ at 20 °C.

(a)

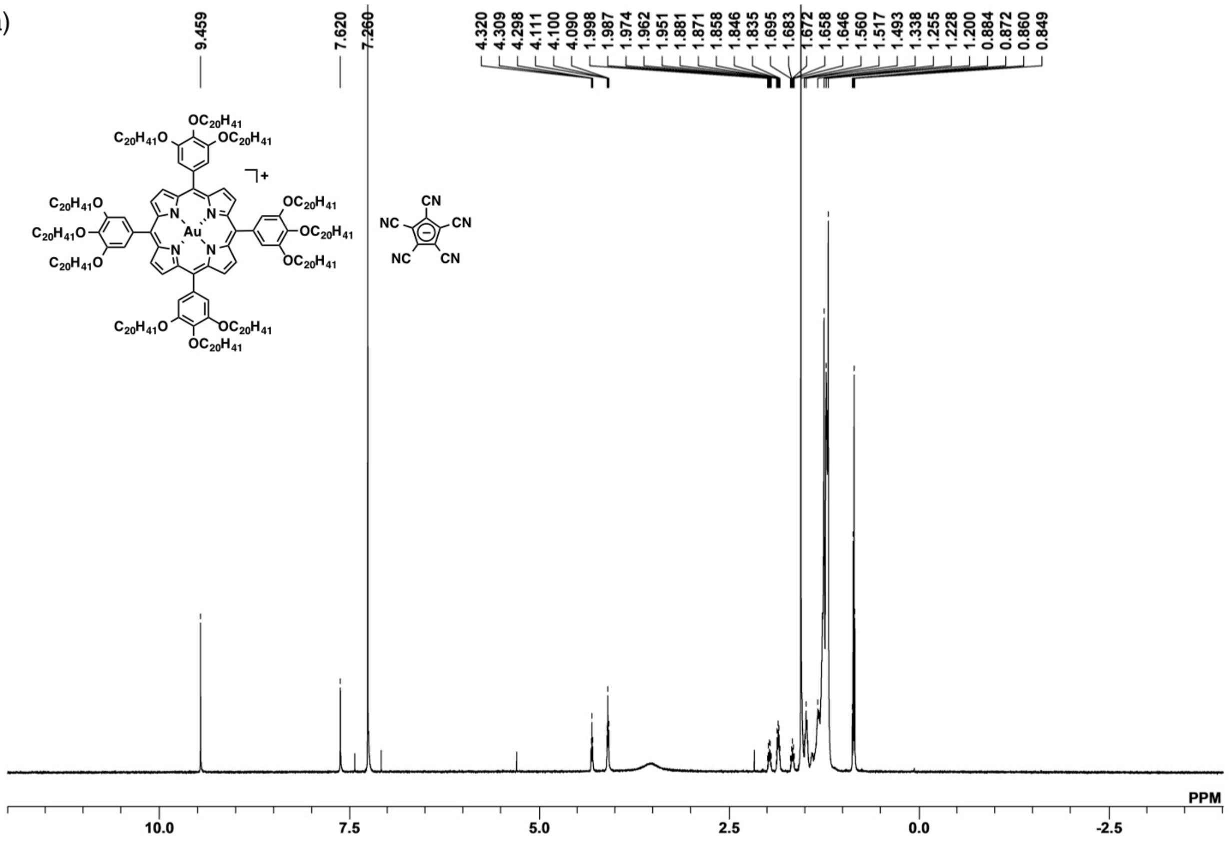

(b)

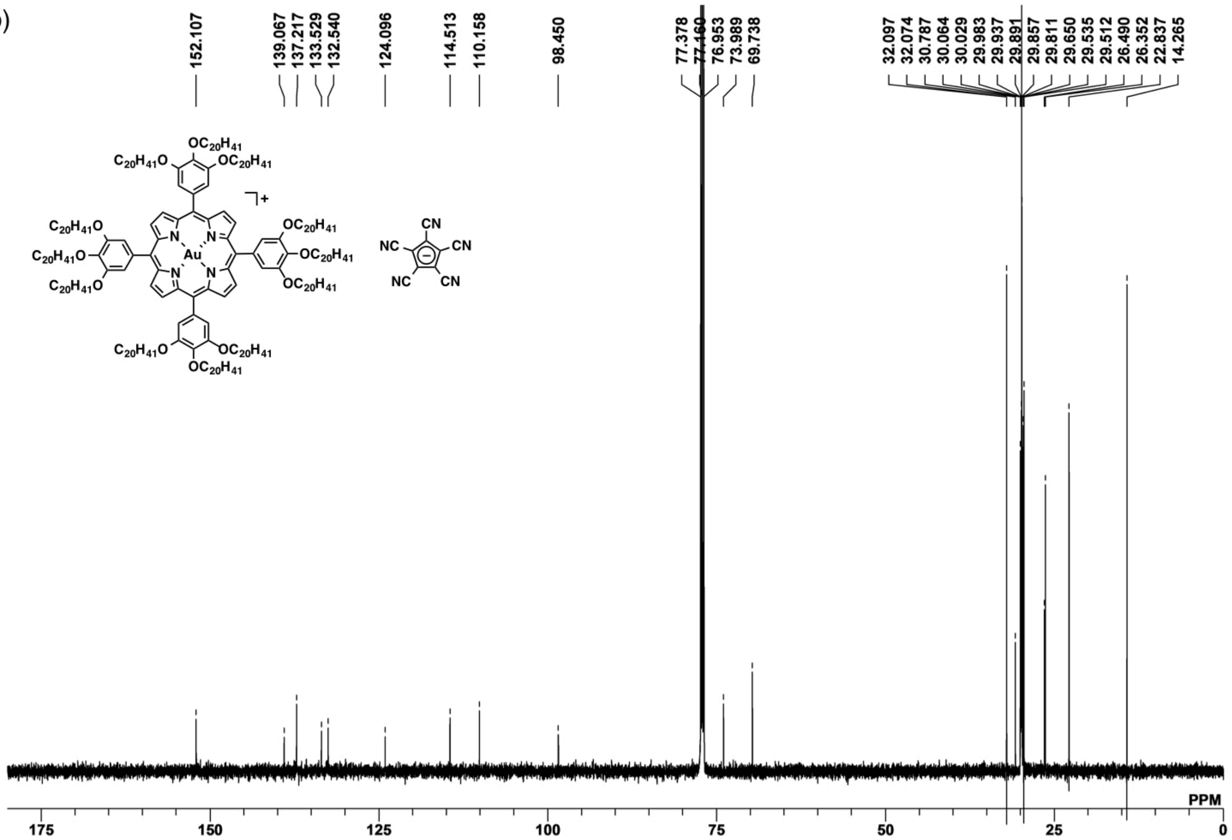

Figure S34 NMR spectra of Au20⁺-PCCp⁻, Related to Figure 2.
(a) ^{1}H NMR and (b) ^{13}C NMR spectra of **Au20**$^{+}$-PCCp^{-} in $CDCl_3$ at 20 °C.

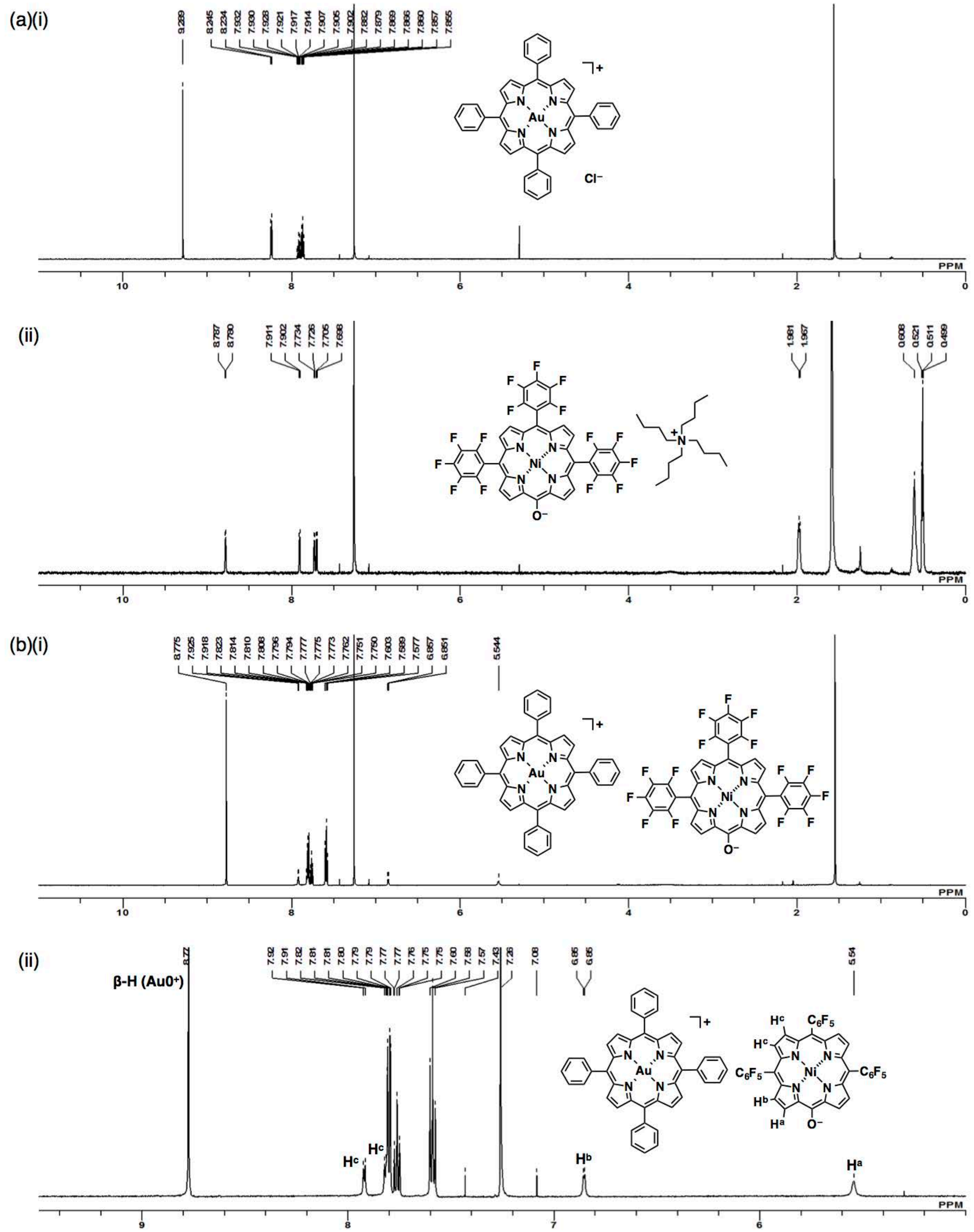

Figure S35 NMR spectra of Au0^{+}-Cl^{-}, TBA^{+}-NiO^{-}, and Au0^{+}-NiO^{-}, Related to Figure 10.
(a) ^{1}H NMR spectra of (i) **Au0**$^{+}$-Cl^{-} and (ii) TBA^{+}-**NiO**$^{-}$ (Sasano et al., 2017), (b) ^{1}H NMR spectra of **Au0**$^{+}$-**NiO**$^{-}$ ((i) 0–11 ppm and (ii) 5–9.5 ppm) in $CDCl_3$ (1.0 × 10^{-3} M) at 20 °C. Upfield shifts of the signals of **Au0**$^{+}$-**NiO**$^{-}$ compared to those of **Au0**$^{+}$-Cl^{-} and TBA^{+}-**NiO**$^{-}$ suggested the interaction between the π-electronic cation and anion.

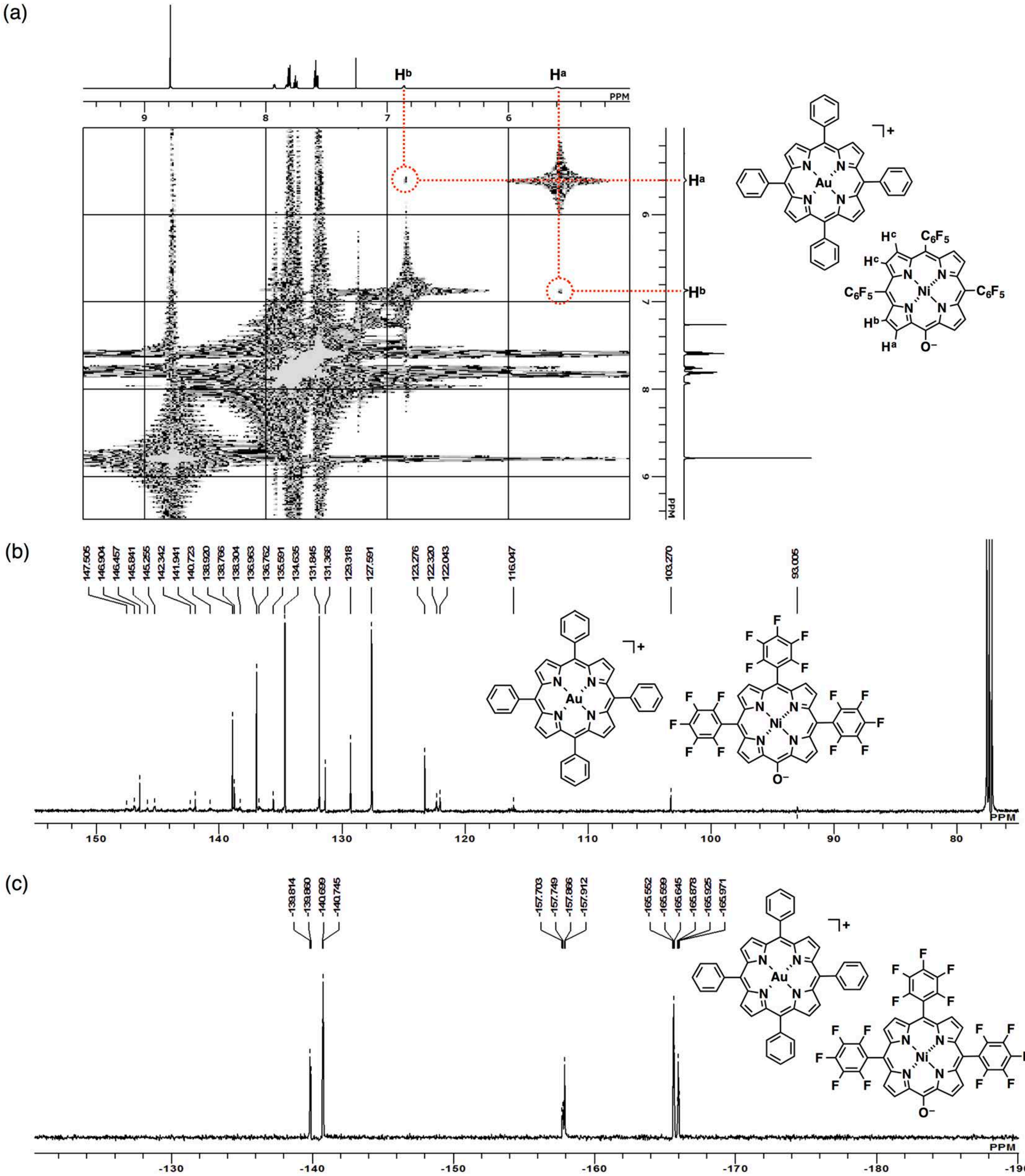

Figure S36 ^{1}H–^{1}H COSY, ^{13}C NMR, ^{19}F NMR spectra of Au0$^+$-NiO$^-$, Related to Figure 10.
(a) ^{1}H–^{1}H COSY spectrum of **Au0$^+$-NiO$^-$** in $CDCl_3$ at 20 °C, (b) ^{13}C NMR, and (c) ^{19}F NMR spectra of **Au0$^+$-NiO$^-$** in $CDCl_3$ at 20 °C. Assignments for H^a, H^b, and H^c were supported by ^{1}H–^{1}H COSY and ^{1}H–^{13}C HMBC spectra (Figure S37), as well as by the optimized stacking structure based on DFT calculations (Figure S59).

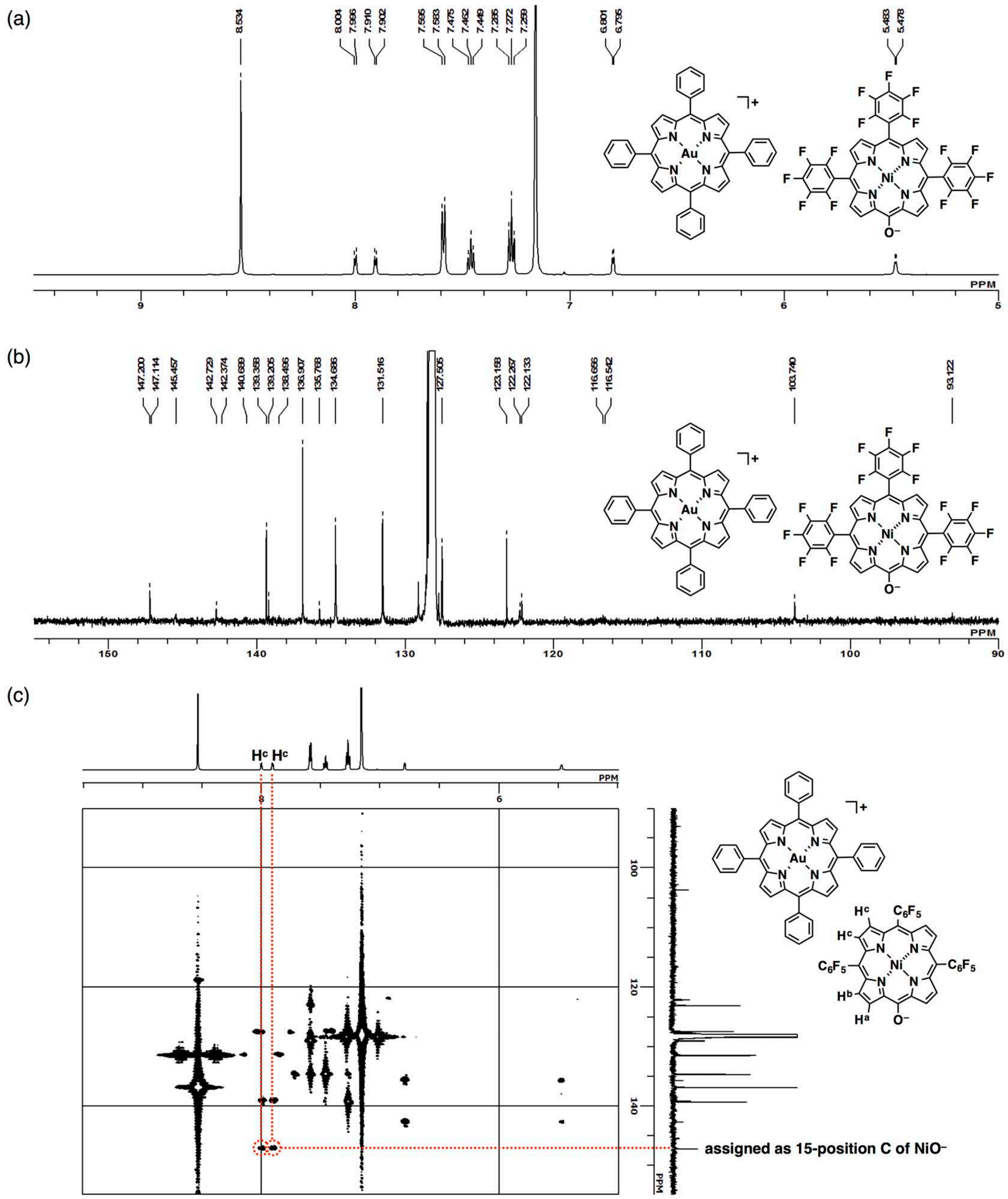

Figure S37 ^{1}H NMR, ^{13}C NMR, and ^{1}H–^{13}C HMBC spectra of Au0⁺-NiO⁻, Related to Figure 10.
(a) ^{1}H NMR, (b) ^{13}C NMR, and (c) ^{1}H–^{13}C HMBC spectra of **Au0⁺**-**NiO⁻** in C_6D_6 (1.0×10^{-2} M), used for clearly separated signals, at 20 °C. Assignments for H^a, H^b, and H^c were supported by ^{1}H–^{1}H COSY (Figure S36) and ^{1}H–^{13}C HMBC spectra, as well as by the optimized stacking structure based on DFT calculations (Figure S59).

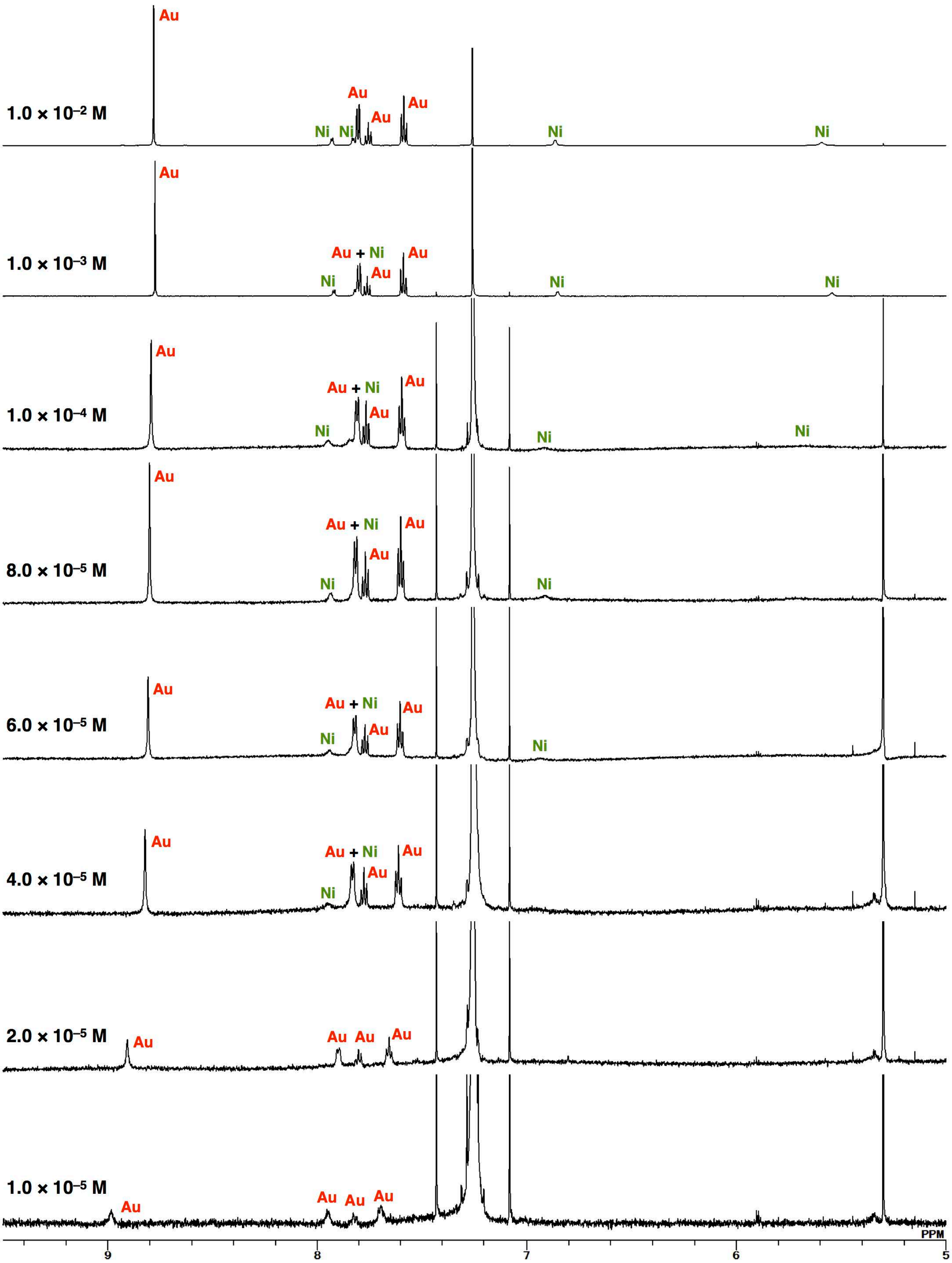

Figure S38 ^{1}H NMR spectra of Au0^{+}-NiO^{-} at various concentrations, Related to Figure 11.

^{1}H NMR spectra of **Au0^{+}-NiO^{-}** at various concentrations in $CDCl_3$ at 20 °C. The labels Au and Ni refer to the signals of **Au0^{+}** and **NiO^{-}**, respectively. The signals of β-H in **Au0^{+}-NiO^{-}** were shifted downfield with broadening when the concentrations were lowered to 1.0×10^{-5} M in $CDCl_3$ at 20 °C, exhibiting the fast exchange between the ion pair and monomeric **Au0^{+}** and **NiO^{-}**. In addition, the downfield shifts of **NiO^{-}** at 1.0×10^{-2} M may be attributed to the partial formation of oligomeric assemblies.

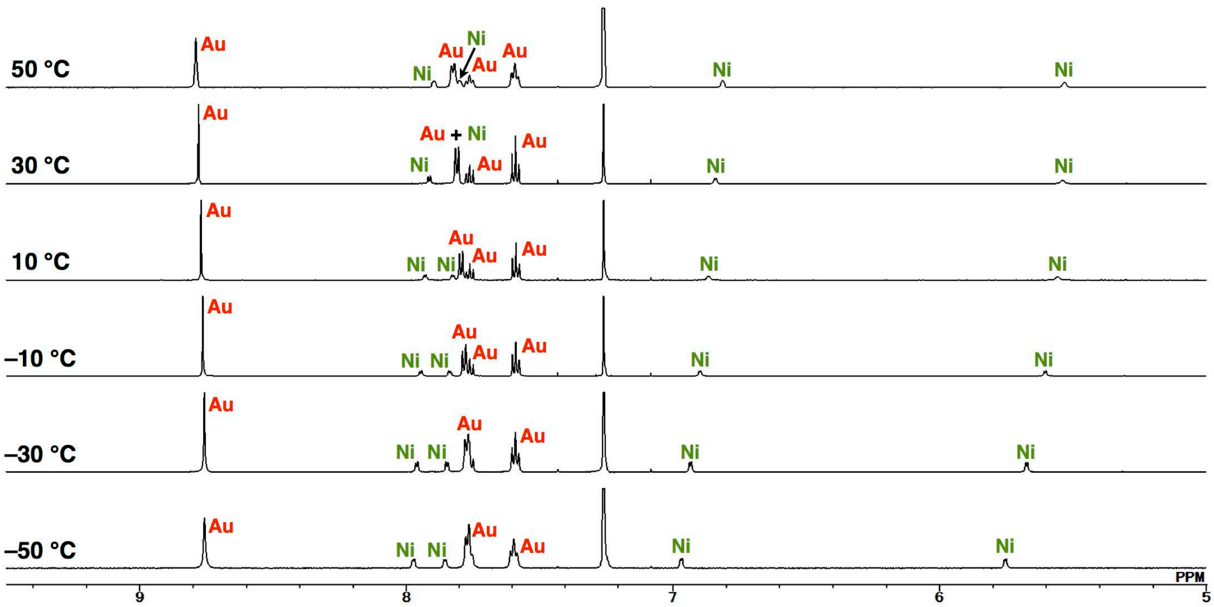

Figure S39 VT-^{1}H NMR spectra of Au0$^+$-NiO$^−$, Related to Figure 11.

VT-^{1}H NMR spectra of **Au0**$^+$-**NiO**$^−$ from 50 °C to –50 °C in $CDCl_3$ (1.0×10^{-3} M). The labels Au and Ni refer to the signals of **Au0**$^+$ and **NiO**$^−$, respectively. The signals of **Au0**$^+$ were shifted upfield and those of **NiO**$^−$ were shifted downfield upon cooling. The results suggested the shielding effect of electron-rich anionic **NiO**$^−$ on **Au0**$^+$ and the deshielding effect of electron-poor cationic **Au0**$^+$ on **NiO**$^−$ in tightly paired states.

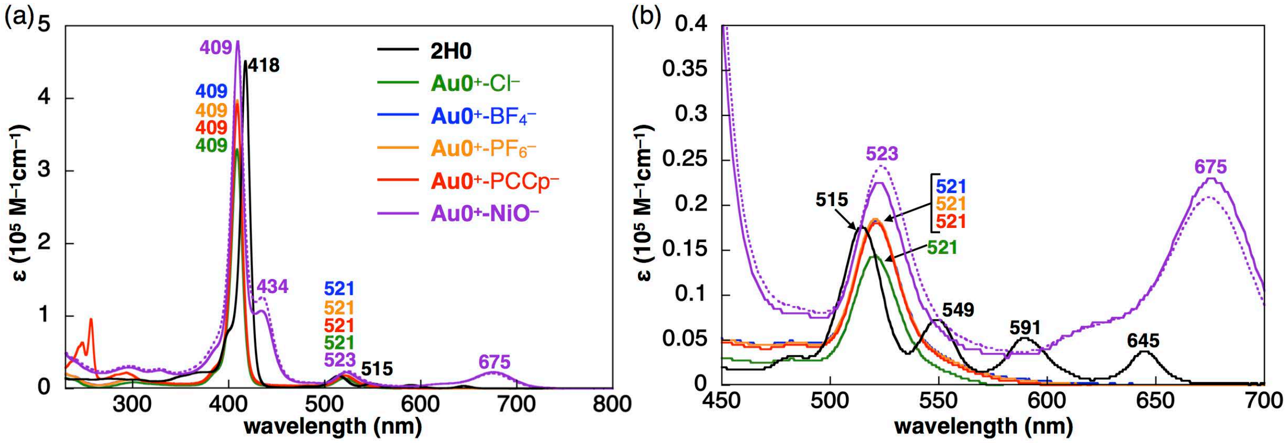

Figure S40 UV/vis absorption spectra of ion pairs, Related to Figure 2 and10.

(a) UV/vis absorption spectra with (b) enlarged version for **2H0** (black), **Au0**$^+$-$Cl^−$ (green), **Au0**$^+$-$BF_4^−$ (blue), **Au0**$^+$-$PF_6^−$ (orange), **Au0**$^+$-PCCp$^−$ (red), and **Au0**$^+$-**NiO**$^−$ (purple) in CH_2Cl_2 (4×10^{-6} M (solid lines) for **2H0**, **Au0**$^+$-$Cl^−$, **Au0**$^+$-$BF_4^−$, **Au0**$^+$-$PF_6^−$, and **Au0**$^+$-PCCp$^−$, and 2×10^{-6} M (solid line) and 4.6×10^{-4} M (dotted line) for **Au0**$^+$-**NiO**$^−$). Au^{III} complexes have similar Soret and Q bands, and thus the spectrum of **Au0**$^+$-$BF_4^−$ (blue) is almost completely overlapped with those of **Au0**$^+$-$PF_6^−$ (orange) and **Au0**$^+$-PCCp$^−$ (red). The ion pairs are soluble in CH_2Cl_2 as monomeric states in these concentrations. In (b), slightly enhanced absorption bands at around 434 and 523 nm were observed for **Au0**$^+$-**NiO**$^−$ at 4.6×10^{-4} M compared to those at 2×10^{-6} M, suggesting the formation of a more amount of the ion pair, whose structure was supported by the DFT study (Figure S59), as observed in the ^{1}H NMR (Figure S35–38). Even in a higher concentration, **Au0**$^+$-**NiO**$^−$ showed the sum of the independent absorption bands derived from each π-electronic ion (Sasano et al., 2017), suggesting that the electronic interaction between π-electronic cation and anion is weak in this condition, as also supported by the independent electron spin densities in the MO for each π-electronic ion (Figure S62).

1-2. X-ray crystallographic data

Single-crystal X-ray analysis. Crystallographic data for ion pairs are summarized in Table S1. A single crystal of **Au0**$^+$-Cl$^-$ showed two crystal pseudo polymorphs (type A and B). A single crystal of **Au0**$^+$-Cl$^-$ (type A) was obtained by vapor diffusion of *n*-hexane into a $CHCl_3$ solution of **Au0**$^+$-Cl$^-$. The data crystal was a red prism of approximate dimensions 0.050 mm × 0.020 mm × 0.020 mm. Data was collected at 90 K on a Rigaku Saturn 724 diffractometer with Si (111) monochromated synchrotron radiation (λ = 0.78255 Å) at BL40XU (SPring-8) (Yasuda et al., 2009; Yasuda et al., 2010). A single crystal of **Au0**$^+$-Cl$^-$ (type B) was obtained by vapor diffusion of *n*-hexane into a $CHCl_3$ solution of **Au0**$^+$-Cl$^-$. The data crystal was a red prism of approximate dimensions 0.22 mm × 0.15 mm × 0.05 mm. Data was collected at 93 K on a Rigaku XtaLAB P200 diffractometer with graphite monochromated Cu-Kα radiation (λ = 1.54187 Å). A single crystal of **Au0**$^+$-BF_4^- was obtained by vapor diffusion of *n*-hexane into a CH_2Cl_2 solution of **Au0**$^+$-BF_4^-. The data crystal was a red prism of approximate dimensions 0.120 mm × 0.002 mm × 0.002 mm. Data was collected at 90 K on a Rigaku Saturn 724 diffractometer with Si (111) monochromated synchrotron radiation (λ = 0.78203 Å) at BL40XU (SPring-8) (Yasuda et al., 2009; Yasuda et al., 2010). A single crystal of **Au0**$^+$-PF_6^- was obtained by vapor diffusion of *n*-hexane into a $(CH_2Cl)_2$ solution of **Au0**$^+$-PF_6^-. The data crystal was a red prism of approximate dimensions 0.020 mm × 0.010 mm × 0.010 mm. The data was collected at 100 K on a CCD diffractometer (Rayonix/MX225HE), with Si (111) monochromated synchrotron radiation (λ = 0.80000 Å) at BL38B1 (SPring-8). A single crystal of **Au0**$^+$-PCCp$^-$ was obtained by vapor diffusion of CH_3CN into a $(CH_2Cl)_2$ solution of **Au0**$^+$-PCCp$^-$. The data crystal was a red prism of approximate dimensions 0.080 mm × 0.040 mm × 0.040 mm. The data was collected at 93 K on a CCD diffractometer (Rayonix/MX225HE), with Si (111) monochromated synchrotron radiation (λ = 0.80000 Å) at BL38B1 (SPring-8). A single crystal of **Au0**$^+$-**NiO**$^-$ was obtained by vapor diffusion of *n*-hexane into an EtOAc solution of the 1:1 mixture of **NiO**$^-$ as a Na^+ salt, which was prepared by washing an CH_2Cl_2 solution of **NiOH** with NaOH aq., and **Au0**$^+$-Cl$^-$ upon washing with ion-exchanged water several times to remove NaCl. The data crystal was a red prism of approximate dimensions 0.010 mm × 0.002 mm × 0.002 mm. Data was collected at 90 K on a Rigaku Saturn 724 diffractometer with Si (111) monochromated synchrotron radiation (λ = 0.78229 Å) at BL40XU (SPring-8) (Yasuda et al., 2009; Yasuda et al., 2010). In each case, the structure was solved by dual-space method, and the non-hydrogen atoms were refined anisotropically. The calculations were performed using Yadokari-XG (Kabuto et al., 2009). CIF files (CCDC-1877986–1877991) can be obtained free of charge from the Cambridge Crystallographic Data Centre via www.ccdc.cam.ac.uk/data_request/cif.

Table S1 Crystallographic details, Related to Figure 4, 5, and 12.

	**Au0**$^+$-Cl$^-$ (type A)[a]	**Au0**$^+$-Cl$^-$ (type B)[a]	**Au0**$^+$-BF_4^-	**Au0**$^+$-PF_6^-	**Au0**$^+$-PCCp$^-$	**Au0**$^+$-**NiO**$^-$
formula	$C_{44}H_{28}AuN_4$·Cl·$CHCl_3$	$C_{44}H_{28}AuN_4$·Cl·4$CHCl_3$	$C_{44}H_{28}AuN_4$·BF_4·CH_2Cl_2	$C_{44}H_{28}AuN_4$·PF_6·$C_2H_4Cl_2$	$C_{44}H_{28}AuN_4$·$C_{10}N_5$·0.416$C_2H_4Cl_2$·C_2H_3N·0.5H_2O	$C_{44}H_{28}AuN_4$·$C_{38}H_8F_{15}N_4NiO$·0.70C_8H_{18}
fw	964.52	1322.59	981.40	1053.59	1091.04	1767.96
crystal size, mm	0.050 × 0.020 × 0.020	0.22 × 0.15 × 0.05	0.120 × 0.002 × 0.002	0.020 × 0.010 × 0.010	0.080 × 0.040 × 0.040	0.010 × 0.002 × 0.002
crystal system	monoclinic	triclinic	monoclinic	triclinic	orthorhombic	monoclinic
space group	*P*2$_1$/*n* (no. 14)	*P*-1 (no. 2)	*P*2$_1$/*n* (no. 14)	*P*-1 (no. 2)	*Fdd*2 (no. 43)	*P*2/*n* (no. 13)
a, Å	11.8931(3)	9.4537(17)	13.9228(5)	9.42100(10)	28.4997(8)	12.8429(6)
b, Å	22.2174(4)	12.527(3)	9.2425(4)	14.51380(10)	48.3324(14)	20.8194(8)
c, Å	14.4087(3)	23.480(4)	28.2662(10)	15.4764(2)	13.4721(3)	27.9708(12)
α, °	90	88.072(19)	90	65.8757(4)	90	90
β, °	103.2750(10)	78.581(15)	94.994(3)	87.6449(5)	90	101.923(3)
γ, °	90	69.026(18)	90	86.7701(6)	90	90
V, Å^3	3705.53(14)	2542.9(9)	3623.5(2)	1927.89(4)	18557.3(9)	7317.5(5)
ρ_{calcd}, gcm^{-3}	1.729	1.727	1.799	1.815	1.562	1.605
Z	4	2	4	2	16	4
T, K	90(2)	93(2)	100(2)	100(2)	93(2)	100(2)
μ, mm^{-1}	5.464[b]	12.037[c]	5.413[b]	5.464[b]	4.390[b]	2.991[b]
no. of reflns	20050	29975	19364	11796	25279	40182
no. of unique reflns	6615	8546	6472	5978	4093	13043
variables	497	622	507	541	618	927
λ, Å	0.78255[b]	1.54187[c]	0.78201[b]	0.80000[b]	0.80000[b]	0.78229[b]
R_1 ($I > 2\sigma(I)$)	0.0245	0.0346	0.0377	0.0394	0.0563	0.0790
wR_2 ($I > 2\sigma(I)$)	0.0538	0.0877	0.0868	0.1061	0.1526	0.1885
GOF	1.043	1.076	1.084	1.041	1.026	0.994

[a] In 1977, Tulinsky, et al. reported the crystal structure of **Au0**$^+$-Cl$^-$ with 0.7 equiv of $CHCl_3$ (Timkovich and Tulinsky, 1977), whose structure was different from our cases (**Au0**$^+$-Cl$^-$ (type A and B)) although the crystallization conditions are similar. [b] The values under the synchrotron radiation. [c] The values under the Cu-Kα radiation.

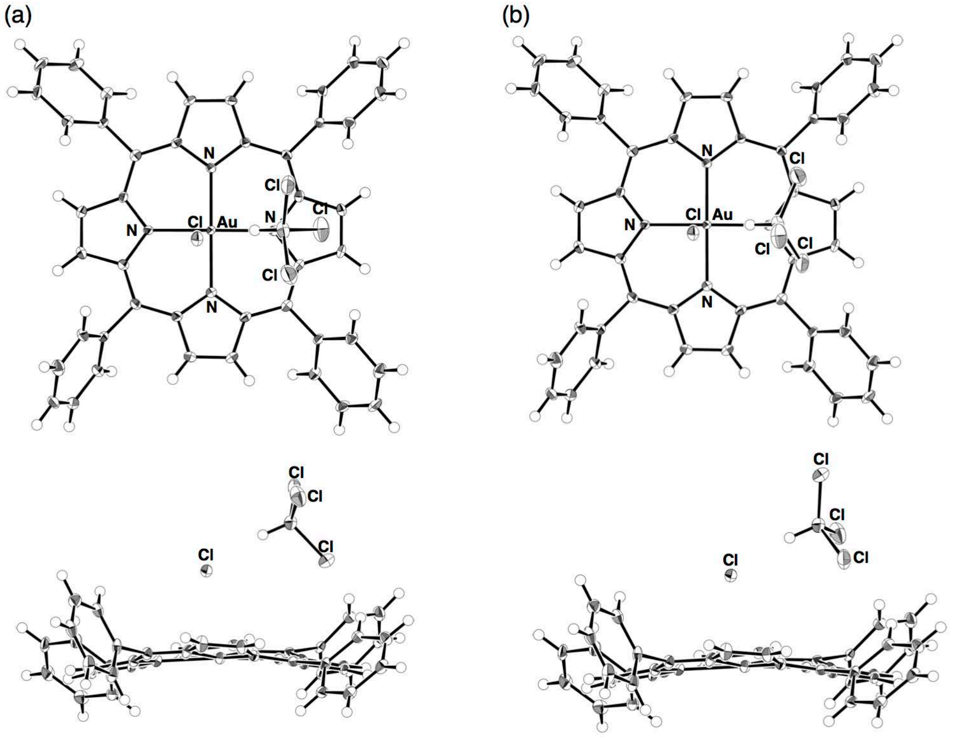

Figure S41 Single-crystal X-ray structure of Au0⁺-Cl⁻, Related to Figure 4.
Single-crystal X-ray structure of **Au0**$^{+}$-Cl^{-} (type A) as Ortep drawings (top and side views) with two disordered $CHCl_3$ (a,b) in the ratio of 0.96:0.04 for top and bottom structures, wherein thermal ellipsoids are scaled to the 50% probability level.

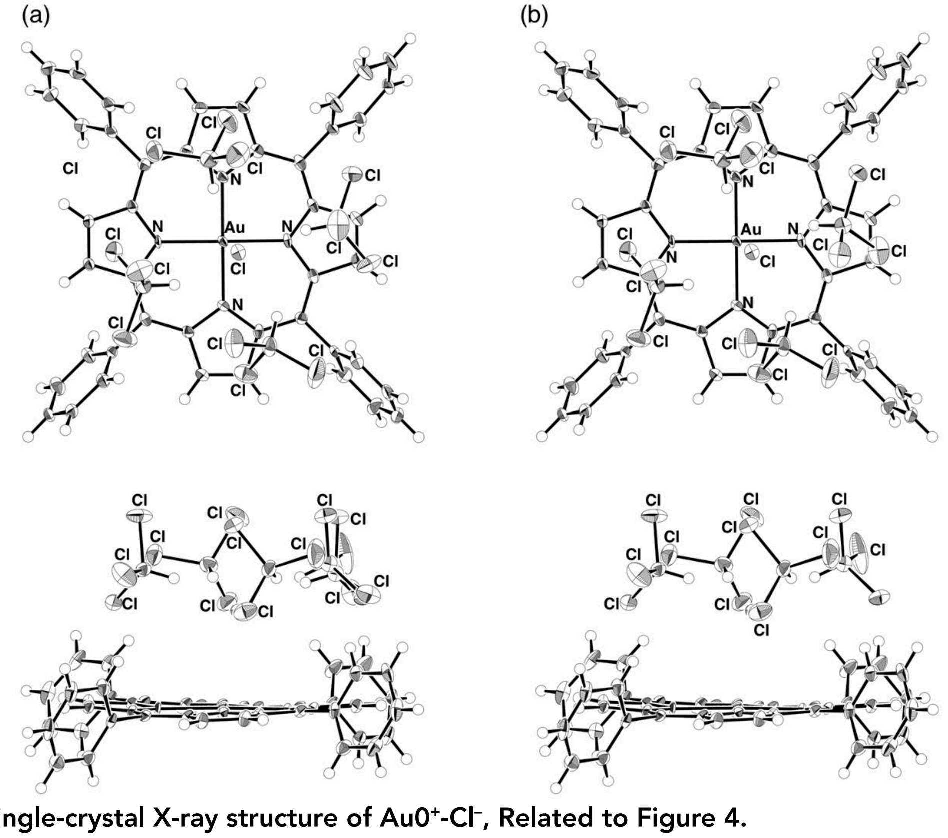

Figure S42 Single-crystal X-ray structure of Au0⁺-Cl⁻, Related to Figure 4.
Single-crystal X-ray structure of **Au0**$^{+}$-Cl^{-} (type B) as Ortep drawings (top and side views) with two disordered $CHCl_3$ (a,b) in the ratio of 0.54:0.46 for top and bottom structures, wherein thermal ellipsoids are scaled to the 50% probability level.

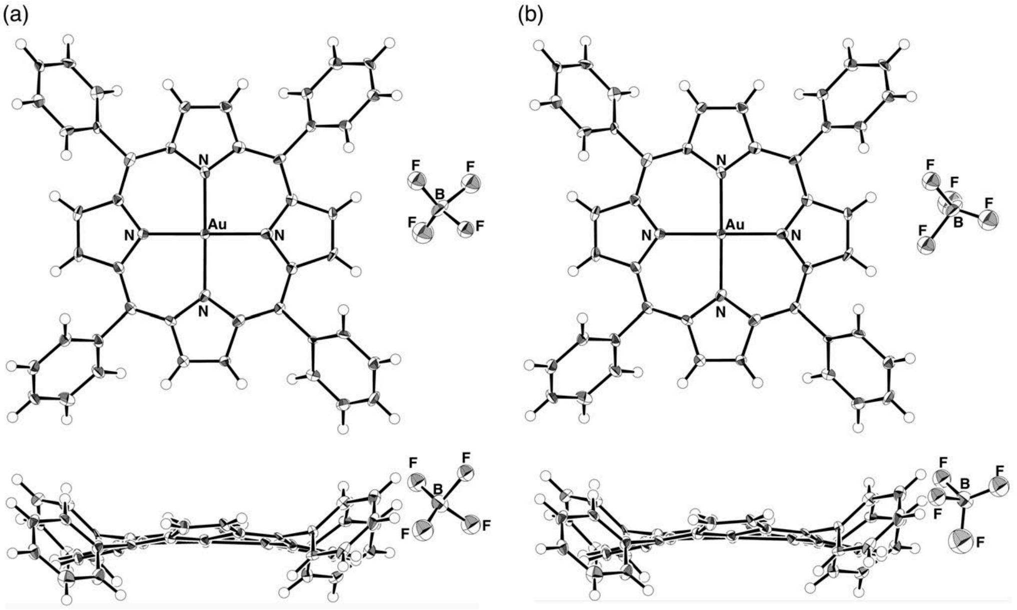

Figure S43 Single-crystal X-ray structure of Au0$^+$-BF_4^-, Related to Figure 5.
Single-crystal X-ray structure of **Au0**$^+$-BF_4^- as Ortep drawings (top and side views) with two disordered BF_4^- (a,b) in the ratio of 0.53:0.47 for top and bottom structures, wherein thermal ellipsoids are scaled to the 50% probability level.

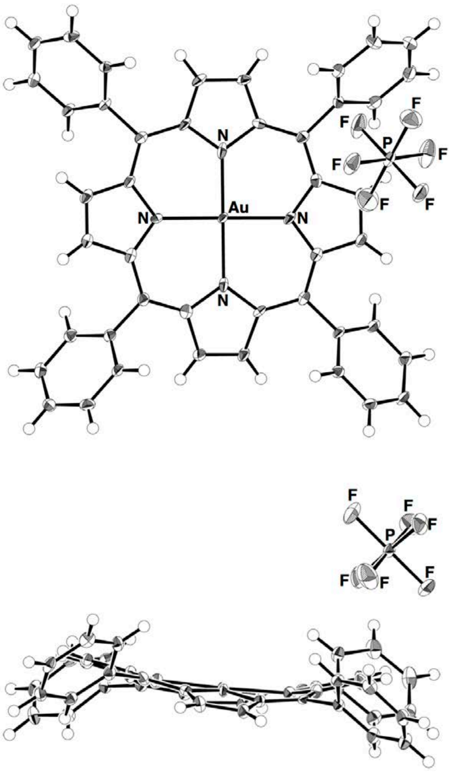

Figure S44 Single-crystal X-ray structure of Au0$^+$-PF_6^-, Related to Figure 5.
Single-crystal X-ray structure of **Au0**$^+$-PF_6^- as Ortep drawings (top and side views) for top and bottom structures, wherein thermal ellipsoids are scaled to the 50% probability level.

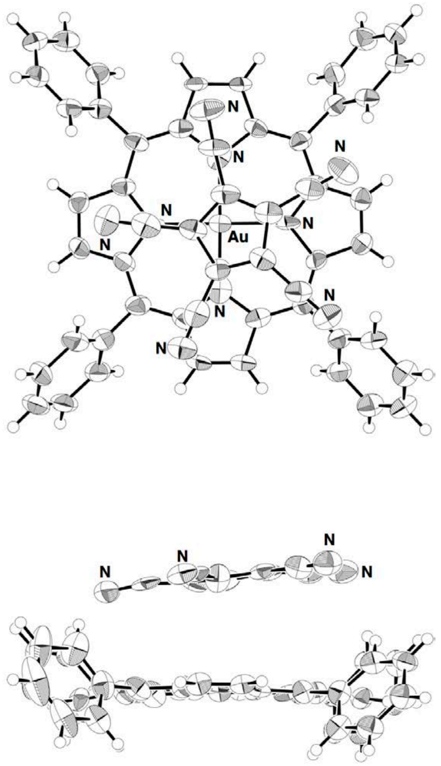

Figure S45 Single-crystal X-ray structure of Au0$^+$-PCCp$^-$, Related to Figure 5.
Single-crystal X-ray structure of **Au0**$^+$-PCCp$^-$ as Ortep drawings (top and side views) for top and bottom structures, wherein thermal ellipsoids are scaled to the 50% probability level.

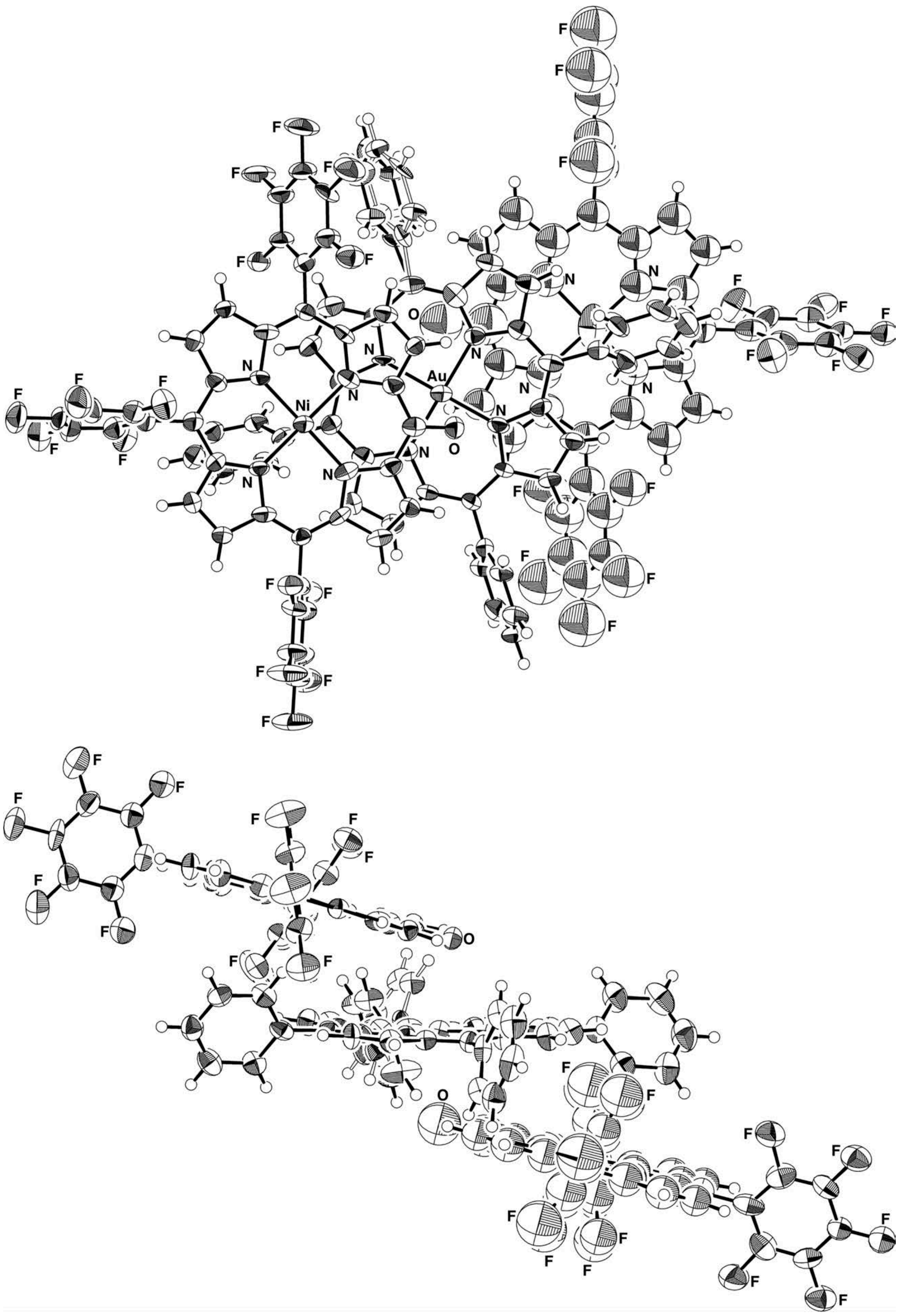

Figure S46 Single-crystal X-ray structure of Au0^{+}-NiO^{-}, Related to Figure 12.
Single-crystal X-ray structure of **Au0^{+}**-**NiO^{-}** as Ortep drawings (top and side views) for top and side views containing an **Au0^{+}** unit and two **NiO^{-}** units. Thermal ellipsoids are scaled to the 50% probability level. One **Au0^{+}** and two independent half anionic species **NiO^{-}**, in which structures were expanded for clarity, exist in the crystal. A phenyl ring of **Au0^{+}** has a disordered structure in the ratio of 52 (black bond) : 48 (white bond).

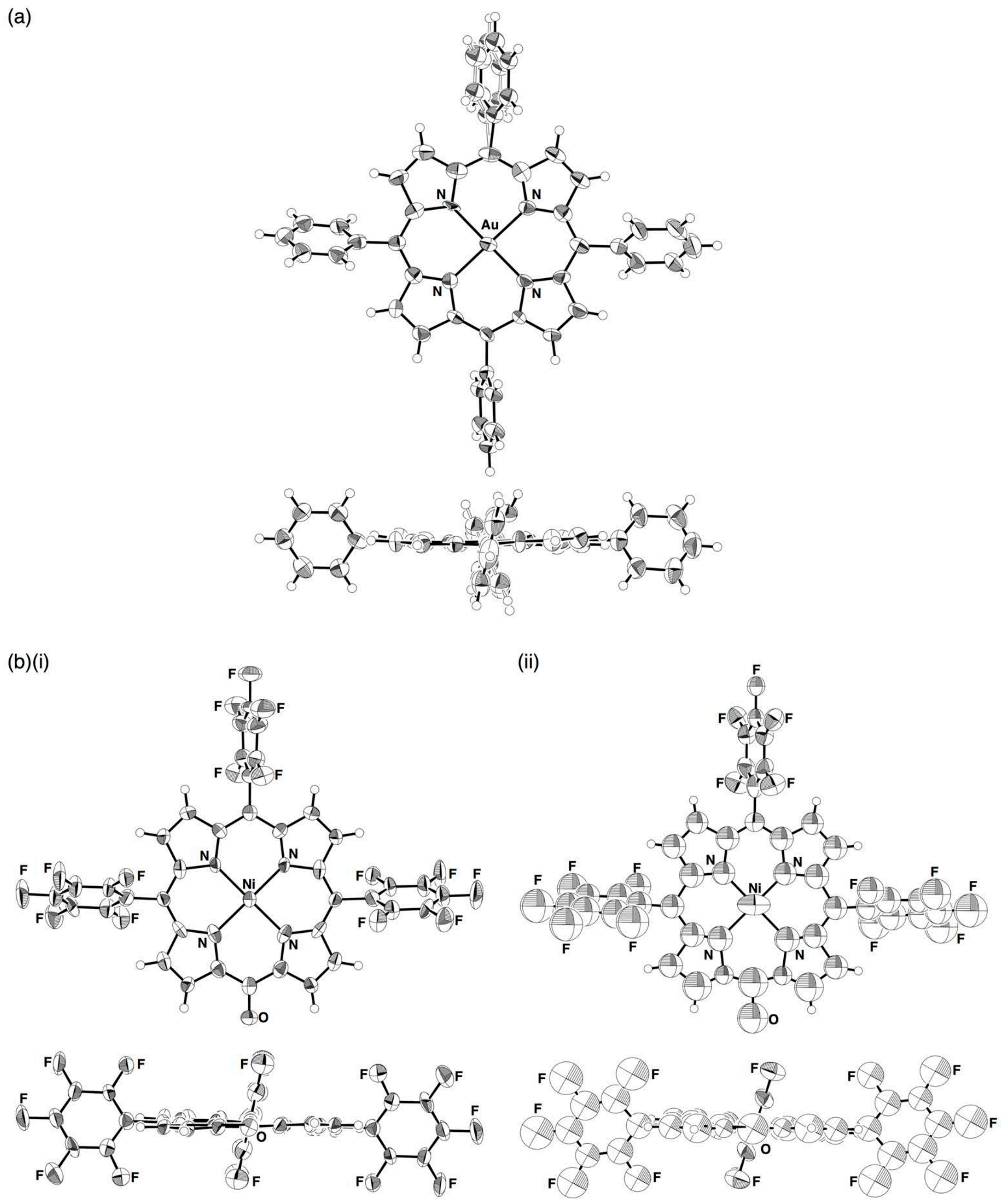

Figure S47 Single-crystal X-ray structure of Au0^{+}-NiO^{-}, Related to Figure 12.
Single-crystal X-ray structure of **Au0^{+}-NiO^{-}** as Ortep drawings (top and side views) for (a) **Au0^{+}** and (b)(i,ii) two independent units of **NiO^{-}** (top and side views). Thermal ellipsoids are scaled to the 50% probability level. One **Au0^{+}** and two independent half anionic species **NiO^{-}**, in which structures were expanded for clarity, exist in the crystal. A phenyl ring of **Au0^{+}** has a disordered structure in the ratio of 52 (black bond) : 48 (white bond). Partial atoms in one of the **NiO^{-}** units ((b)(ii)) were set as isotropic displacement parameters.

(a)

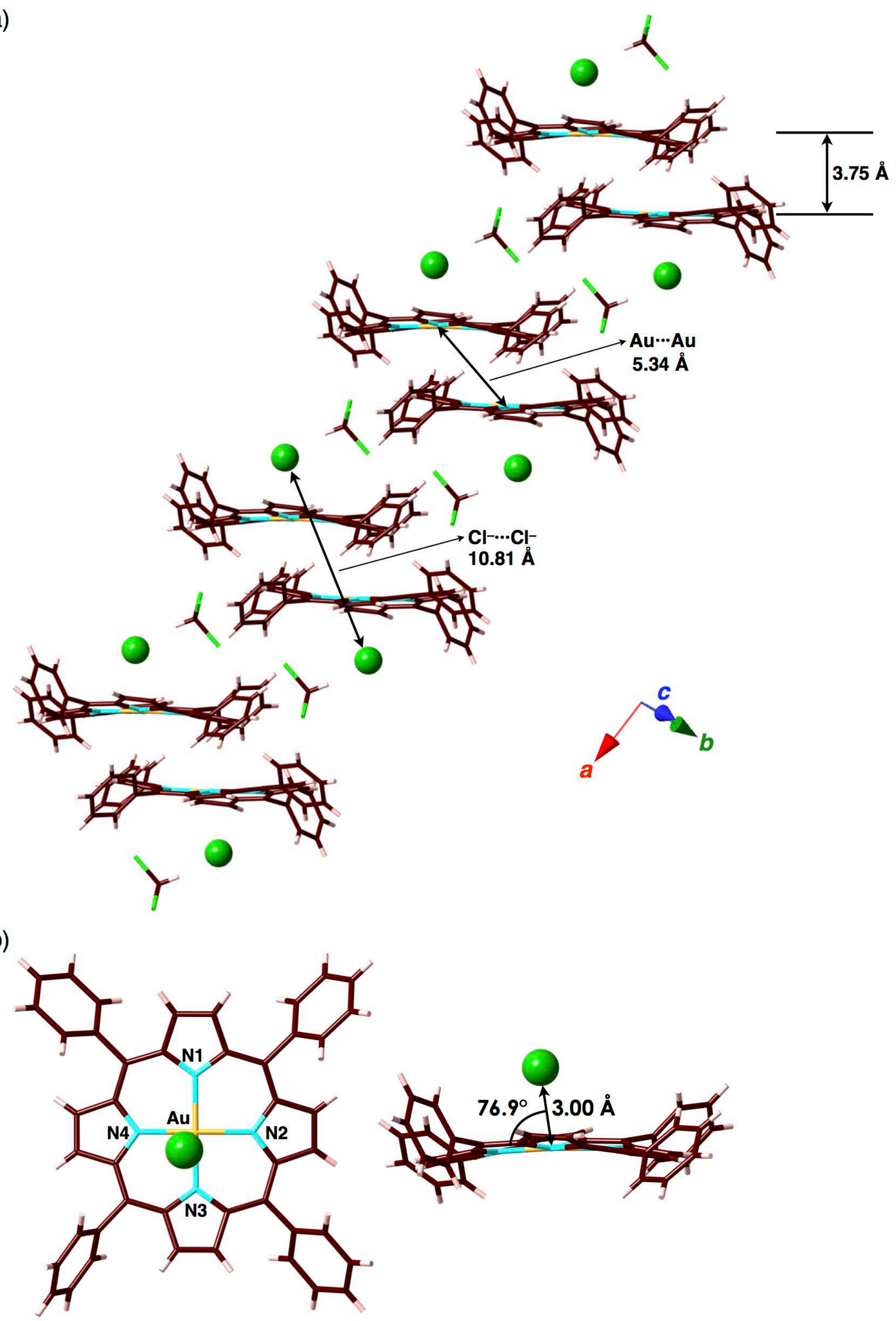

Figure S48 Packing diagram of Au0^{+}-Cl^{-}, Related to Figure 4.

(a) Packing diagram of **Au0**$^{+}$-Cl^{-} (type A), wherein **Au0**$^{+}$-Cl^{-} forms a stacking dimeric assembly with the columnar structure along the *a*-axis, and (b) top and side views of enlarged ion pair. The pyrrole-N1,2,3–Au and pyrrole-N4–Au distances are 2.02 and 2.03 Å, respectively. The N1–Au–N2, N2–Au–N3, N3–Au–N4, and N4–Au–N1 angles are 90.4°, 89.5°, 90.5°, and 89.6°, respectively. The distance between nearest Cl^{-}···Au is 3.00 Å and the angle of the line through Cl^{-} and Au to the core porphyrin plane (core 25 atoms including Au) is 76.9°, suggesting that Cl^{-} has no coordination to Au^{III}. The distances between two **Au0**$^{+}$, Au···Au, and Cl^{-}···Cl^{-} in the column are 3.75, 5.34, and 10.81 Å, respectively. Atom color code: brown, pink, light blue, green, and light orange refer to carbon, hydrogen, nitrogen, chorine, and gold, respectively.

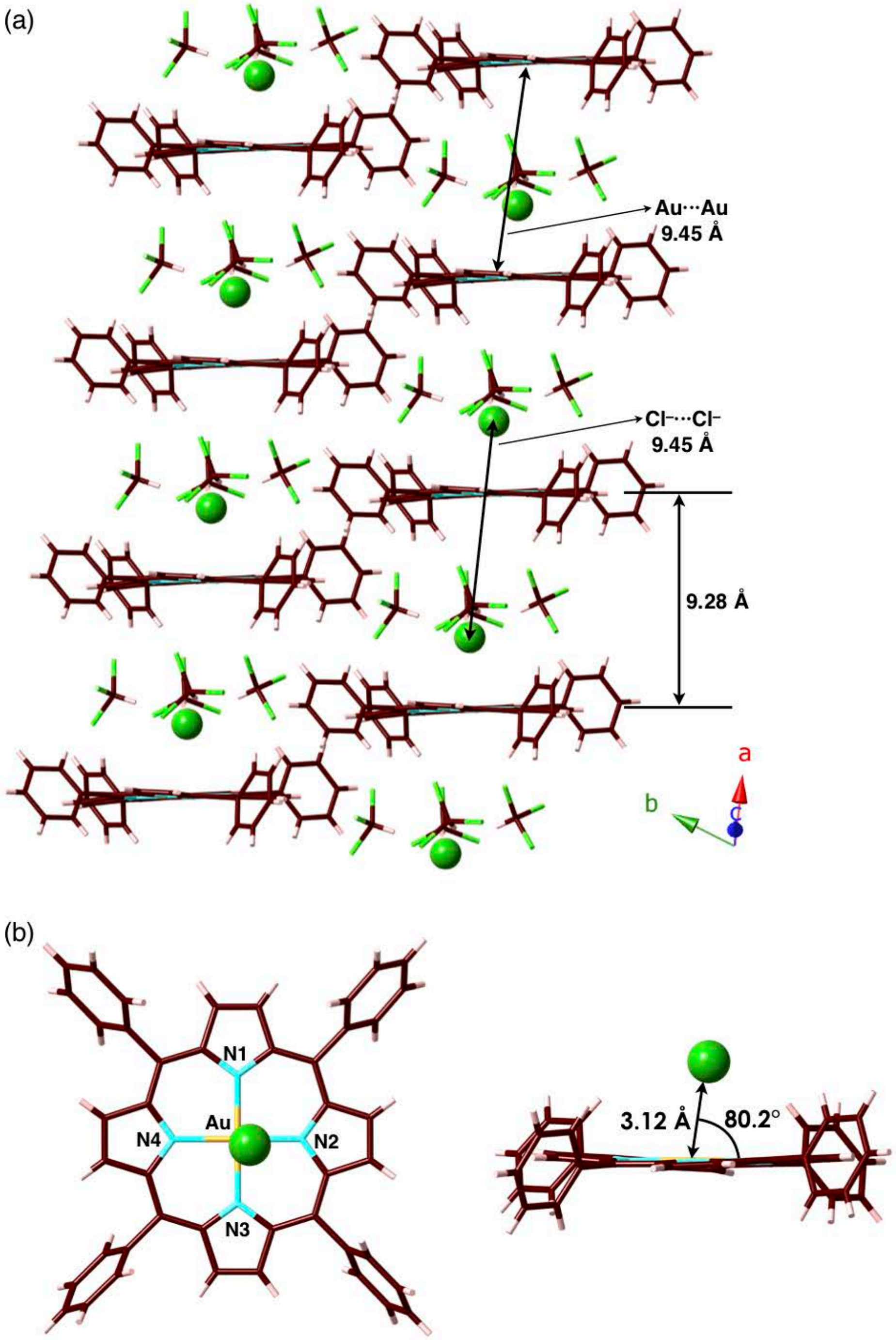

Figure S49 Packing diagram of Au0$^+$-Cl$^-$, Related to Figure 4.

(a) Packing diagram of **Au0**$^+$-Cl$^-$ (type B), wherein **Au0**$^+$-Cl$^-$ forms a charge-by-charge assembly with the columnar structure along the *a*-axis, and (b) top and side views of the enlarged ion pair. All the pyrrole-N–Au distances are 2.03 Å, whereas the N1–Au–N2, N2–Au–N3, N3–Au–N4, and N4–Au–N1 angles are 90.1°, 89.8°, 89.9°, and 90.1°, respectively. The distance between nearest Cl$^-$···Au is 3.12 Å and the angle of the line through Cl$^-$ and Au to the core porphyrin plane (core 25 atoms including Au) is 80.2°, suggesting that Cl$^-$ has no coordination to Au^{III}. The distances between two **Au0**$^+$, Au···Au, and Cl$^-$···Cl$^-$ in the column are 9.28, 9.45, and 9.45 Å, respectively. Atom color code: brown, pink, light blue, green, and light orange refer to carbon, hydrogen, nitrogen, chorine, and gold, respectively.

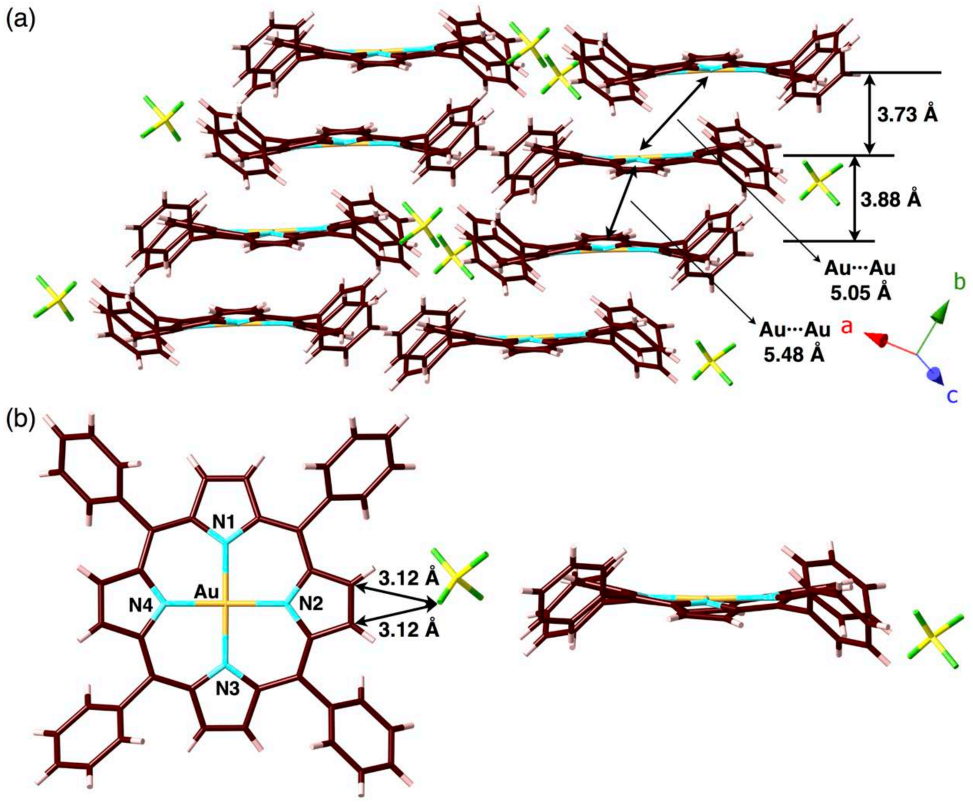

Figure S50 Packing diagram of Au0⁺-BF_4^-, Related to Figure 5.
(a) Packing diagram of **Au0**⁺-BF_4^-, wherein **Au0**⁺-BF_4^- forms a charge-segregated assembly with the columnar structure along the *b*-axis, and (b) top and side views of the enlarged ion pair. All the pyrrole-N–Au distances are 2.02 Å, whereas the N1–Au–N2, N2–Au–N3, N3–Au–N4, and N4–Au–N1 angles are 90.0°, 90.5°, 89.9°, and 89.7°, respectively. The stacking distances between two **Au0**⁺ (core 25 atoms) and the Au···Au distances in the column are 3.73/3.88 and 5.05/5.48 Å, respectively. The BF_4^- anion is proximally located around the porphyrin–Au^{III} complex with the pyrrole-β-C(–H)···F distance of 3.12 Å. Solvent molecules are omitted for clarity. Atom color code: brown, pink, yellow, light blue, light green, and light orange refer to carbon, hydrogen, boron, nitrogen, fluorine, and gold, respectively.

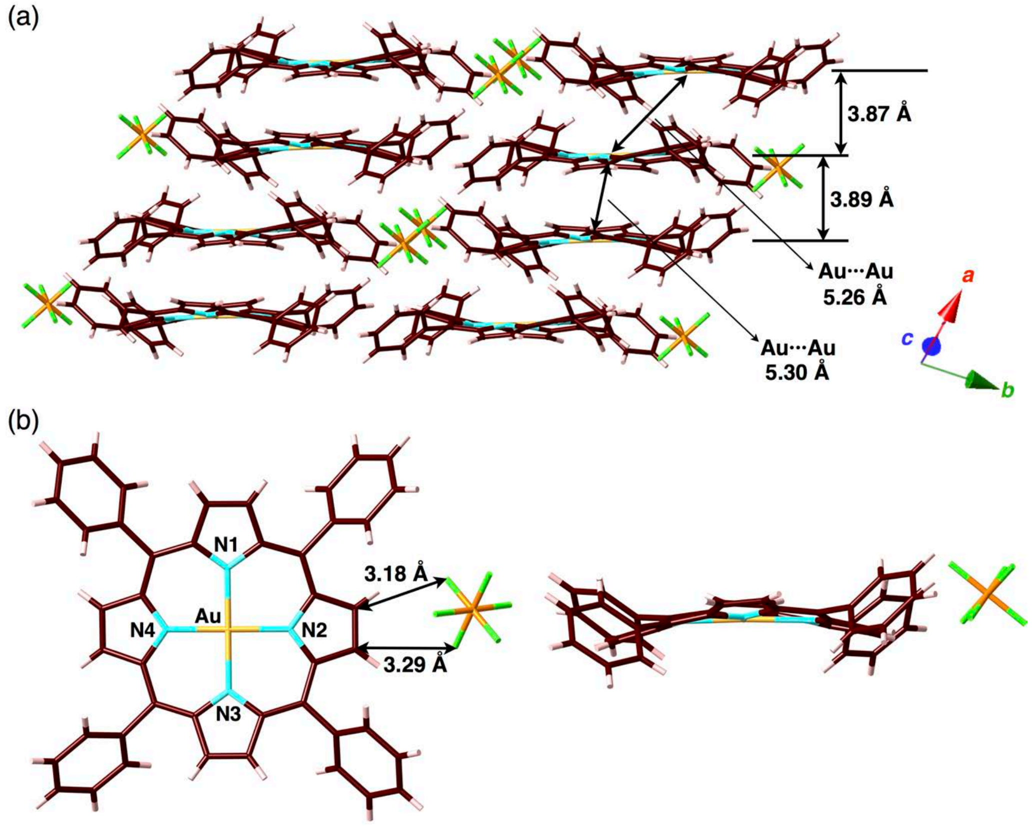

Figure S51 Packing diagram of Au0$^+$-PF$_6^-$, Related to Figure 5.
(a) Packing diagram of **Au0**$^+$-PF$_6^-$, wherein **Au0**$^+$-PF$_6^-$ forms a charge-segregated assembly with the columnar structure along the *a*-axis, and (b) top and side views of the enlarged ion pair. The pyrrole-N1–Au and pyrrole-N2,3,4–Au distances are 2.01 and 2.02 Å, respectively, whereas the N1–Au–N2, N2–Au–N3, N3–Au–N4, and N4–Au–N1 angles are 90.4°, 89.8°, 90.6°, and 89.3°, respectively. The stacking distances between two **Au0**$^+$ (core 25 atoms) and the Au···Au distances in the column are 3.87/3.89 and 5.26/5.30 Å, respectively. The PF$_6^-$ anion is proximally located around the porphyrin–Au^{III} complex with the pyrrole-β-C(–H)···F distances of 3.18 and 3.29 Å. Solvent molecules are omitted for clarity. Atom color code: brown, pink, light blue, light green, orange, and light orange refer to carbon, hydrogen, nitrogen, fluorine, phosphorus, and gold, respectively.

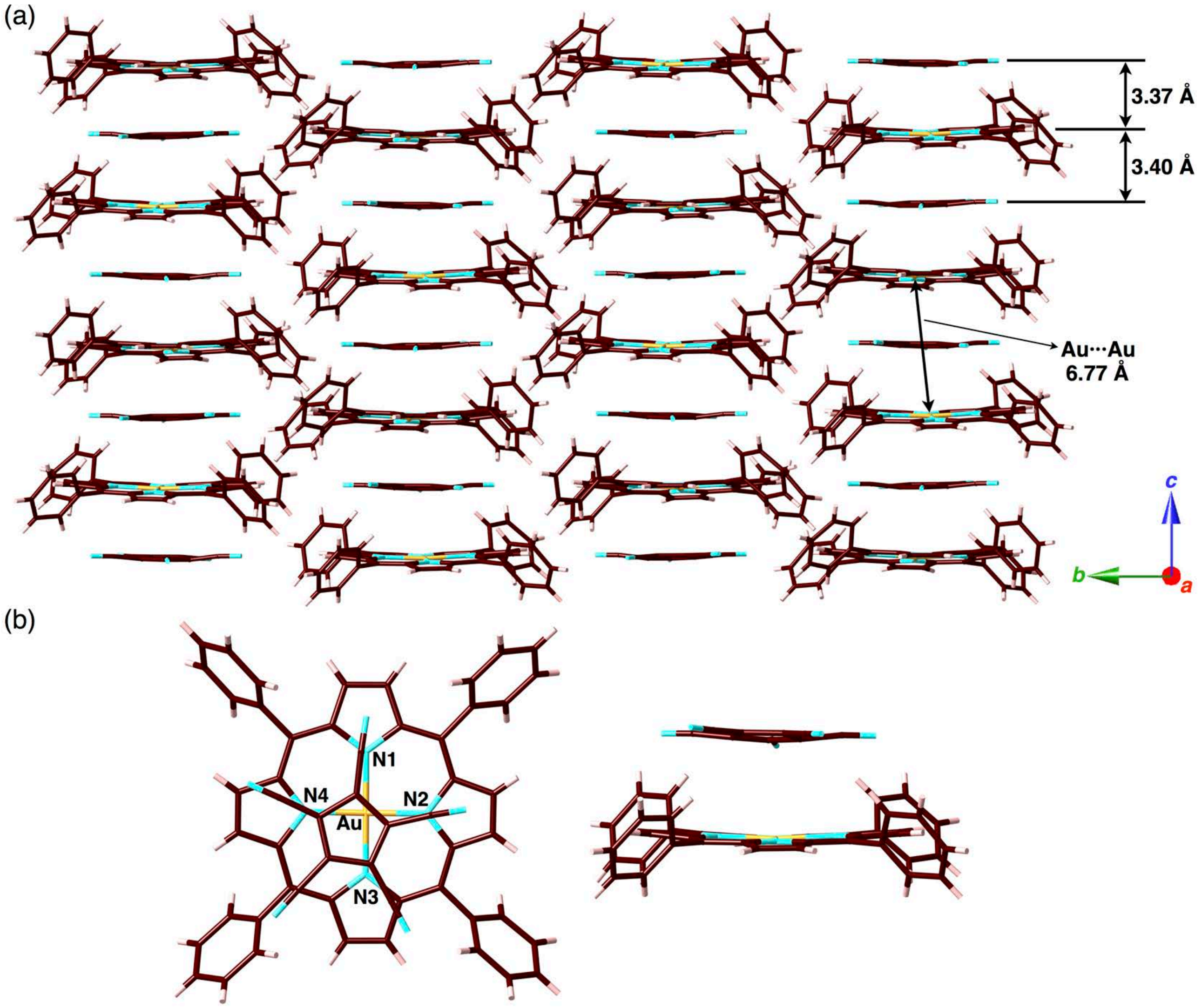

Figure S52 Packing diagram of Au0⁺-PCCp⁻, Related to Figure 5.

(a) Packing diagram of **Au0**⁺-PCCp⁻, wherein **Au0**⁺-PCCp⁻ forms a charge-by-charge assembly with the columnar structure along the *c*-axis, and (b) top and side views of the enlarged ion pair. The pyrrole-N1–Au, pyrrole-N2–Au, pyrrole-N3–Au, and pyrrole-N4–Au distances are 2.01, 1.99, 2.00, and 1.98 Å, respectively, whereas the N1–Au–N2, N2–Au–N3, N3–Au–N4, and N4–Au–N1 angles are 90.1°, 90.1°, 91.1°, and 88.9°, respectively. The stacking distances between **Au0**⁺ (core 25 atoms) and PCCp⁻ and the Au···Au distance in the column are 3.37/3.40 and 6.77 Å, respectively, suggesting that the charge-by-charge assembly is stabilized by π–π stacking and electrostatic interactions. In the charge-by-charge stacking columnar structure, *meso*-phenyl rings are aligned in the same direction in order to minimize the steric repulsion with cyano groups of PCCp⁻. Furthermore, neighboring **Au0**⁺ and PCCp⁻ are alternately arranged not only in the columnar direction (*c*-axis) but also in the intercolumnar (*a*- and *b*-axis) direction. Solvent molecules are omitted for clarity. Atom color code: brown, pink, light blue, and light orange refer to carbon, hydrogen, nitrogen, and gold, respectively.

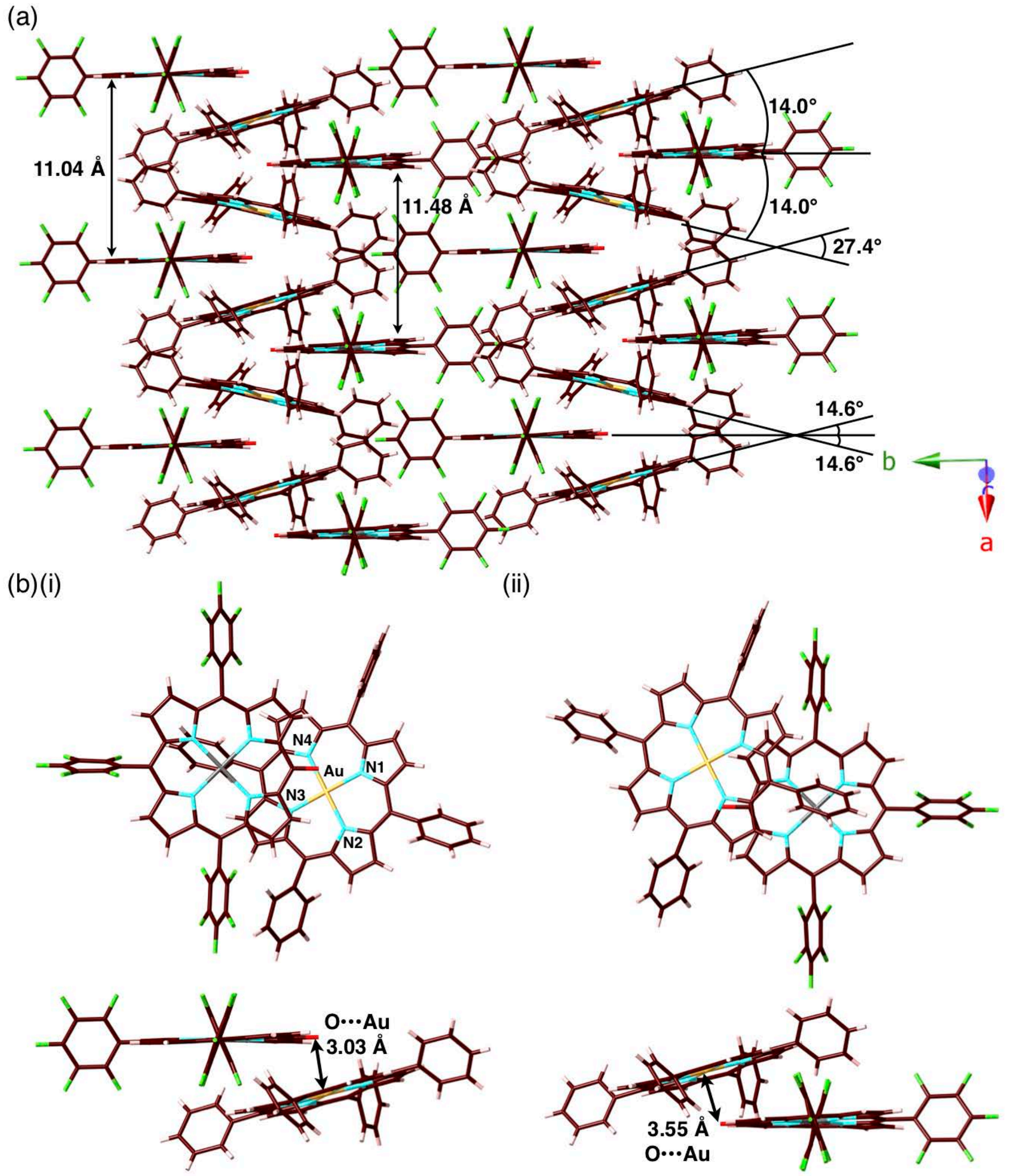

Figure S53 Packing diagram of Au0⁺-NiO⁻, Related to Figure 12.

(a) Packing diagram of **Au0⁺-NiO⁻**, wherein **Au0⁺-NiO⁻** forms a charge-segregated assembly with the columnar structure along the *c*-axis, and (b) top and side views of the enlarged ion pairs with independent **NiO⁻**. The pyrrole-N1–Au, pyrrole-N2–Au, and pyrrole-N3,4–Au distances are 2.03, 1.99, and 2.02 Å, respectively, whereas the N1–Au–N2, N2–Au–N3, N3–Au–N4, and N4–Au–N1 angles are 88.9°, 90.9°, 89.7°, and 90.6°, respectively. The (C–)O···Au distances were 3.03 and 3.55 Å with the O–Au–**Au0⁺**-plane (core 25 atoms) angles of 71.5° and 55.9°, respectively. The distance between mean planes of **NiO⁻** (core 25 atoms) were 11.04 and 11.48 Å. The dihedral angles between **Au0⁺** and **NiO⁻** were 14.0° and 14.6° and that between **Au0⁺** was 27.4°. Solvent molecules are omitted for clarity. Atom color code: brown, pink, light blue, light green, gray, and light orange refer to carbon, hydrogen, nitrogen, fluorine, nickel, and gold, respectively.

1-3. Theoretical studies

Semi-empirical calculations and DFT calculations. Semi-empirical calculations and DFT calculations for porphyrin derivatives and Au^{III} complexes were carried out by using the Gaussian 09 program (Frisch et al., 2013).

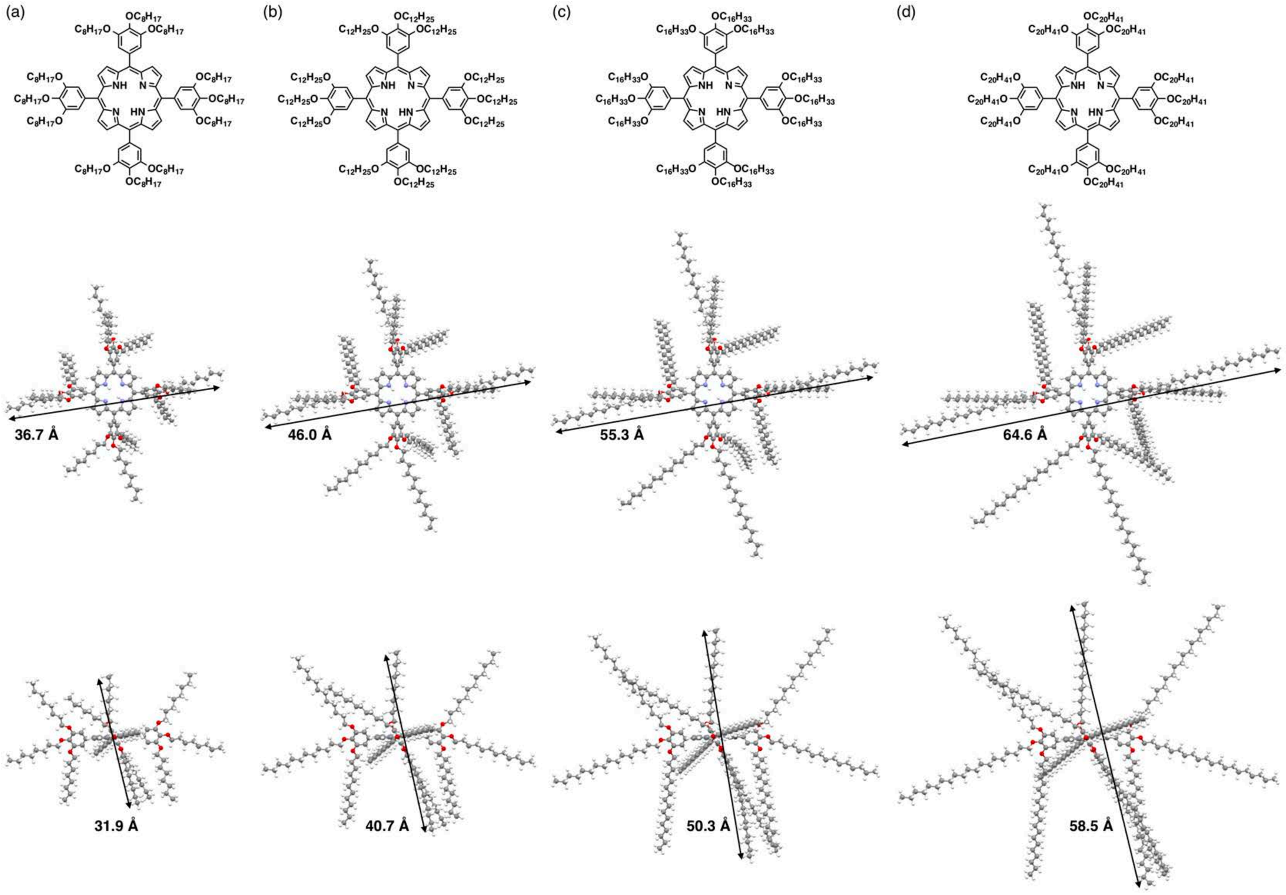

Figure S54 Optimized structures of metal-free porphyrins, Related to Figure 8.
Optimized structures (top and side views) of (a) **2H8**, (b) **2H12**, (c) **2H16**, and (d) **2H20** at AM1 level.

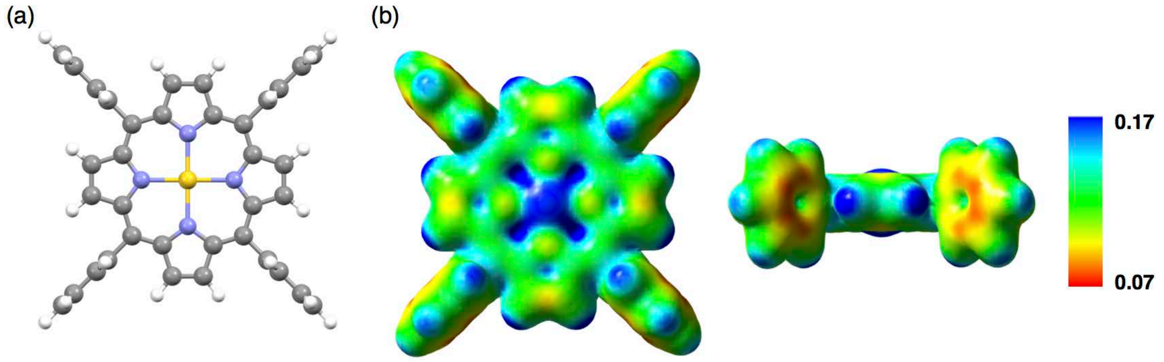

Figure S55 Optimized structures and electron density diagrams of Au^{III} complexes of $Au0^+$, Related to Figure 2.
(a) Optimized structures and (b) electron density diagrams of Au^{III} complexes of **$Au0^+$**. Geometry optimizations were performed using the DFT functional B3LYP and the basis set 6-31+G(d,p) for C, H, N, and O and def2TZVP for Au. Electrostatic potentials were mapped onto the electron density isosurface (δ = 0.01) calculated at B3LYP/6-31+G(d,p) for C, H, and N and def2TZVP for Au. Atom color code: gray, white, blue, and yellow refer to carbon, hydrogen, nitrogen, and gold, respectively.

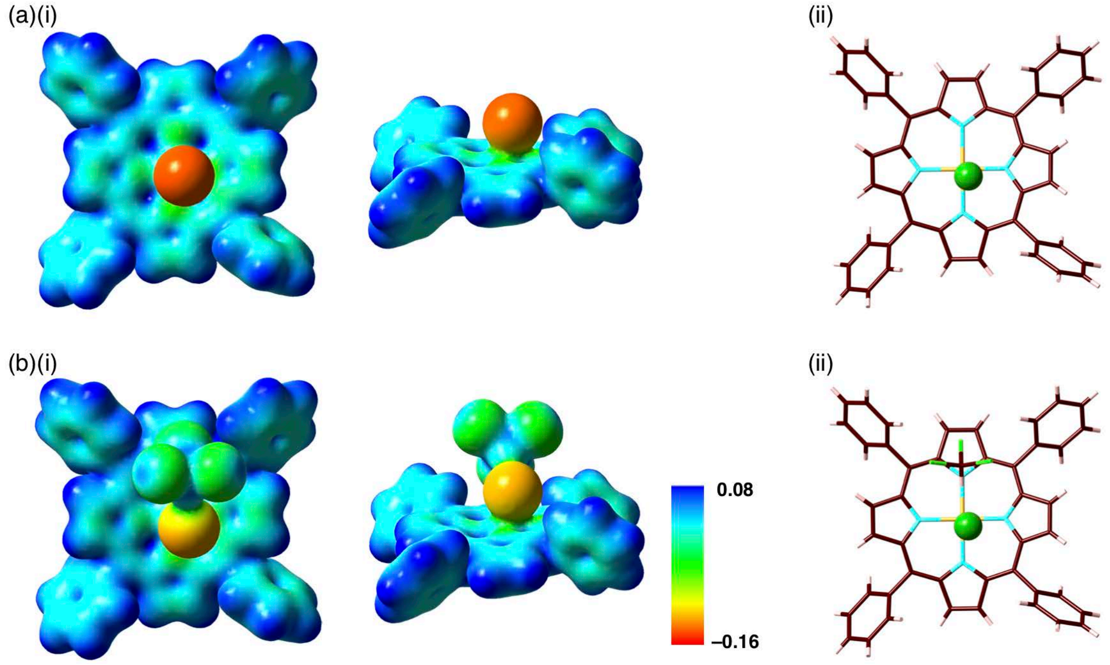

Figure S56 Electron density diagrams of Au0-Cl⁻, Related to Figure 4.
(i) Electron density diagrams (top and side perspective views) of (a) **Au0**-Cl^- and (b) $\mathbf{Au0}^+$-Cl^- with solvated $CHCl_3$ to Cl^- observed in (ii) the crystal structure of $\mathbf{Au0}^+$-Cl^- (type A) (Figure S41,48). Electrostatic potentials were mapped onto the electron density isosurface (δ = 0.01) calculated at B3LYP/6-31+G(d,p) for C, H, N, and Cl and def2TZVP for Au. Greater electron density was observed in $\mathbf{Au0}^+$ at the site proximal to Cl^-. Negative charge of Cl^- decreased by $\mathbf{Au0}^+$ and solvating $CHCl_3$. Atom color code: brown, pink, light blue, green, and light orange refer to carbon, hydrogen, nitrogen, chorine, and gold, respectively.

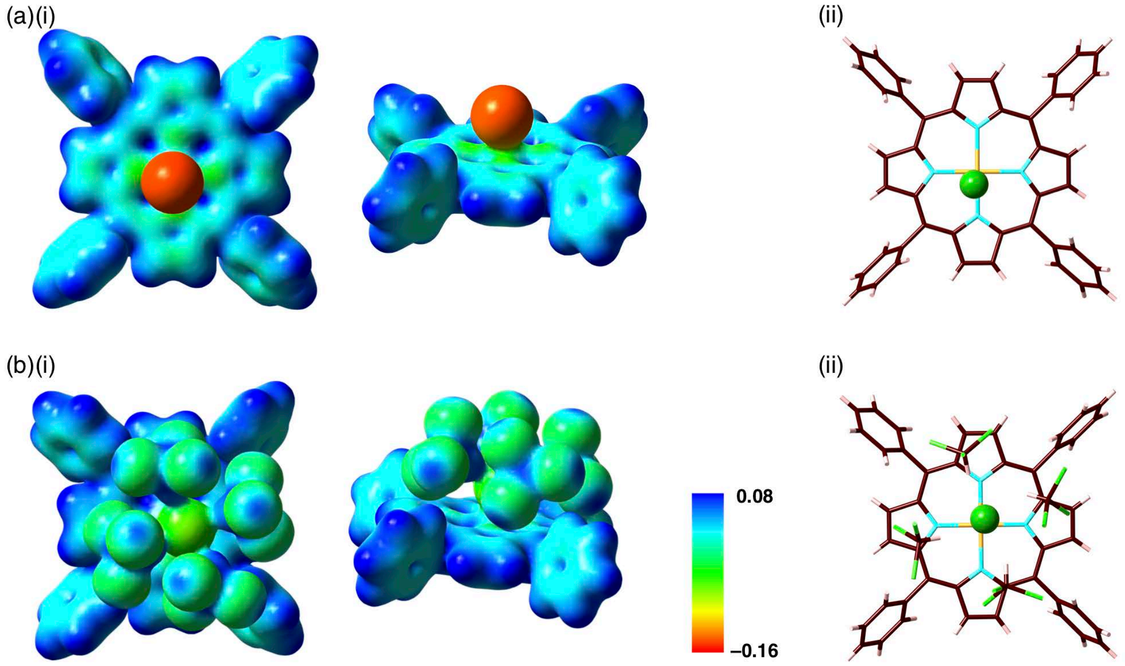

Figure S57 Electron density diagrams of Au0-Cl⁻, Related to Figure 4.
(i) Electron density diagrams (top and side perspective views) of (a) $\mathbf{Au0}^+$-Cl^- and (b) $\mathbf{Au0}^+$-Cl^- with solvated $CHCl_3$ to Cl^- observed in (ii) the crystal structure of $\mathbf{Au0}^+$-Cl^- (type B) (Figure S42,49). Electrostatic potentials were mapped onto the electron density isosurface (δ = 0.01) calculated at B3LYP/6-31+G(d,p) for C, H, N, and Cl and def2TZVP for Au. Greater electron density was observed in $\mathbf{Au0}^+$ at the site proximal to Cl^-. Negative charge of Cl^- decreased by $\mathbf{Au0}^+$ and solvating $CHCl_3$. Atom color code: brown, pink, light blue, green, and light orange refer to carbon, hydrogen, nitrogen, chorine, and gold, respectively.

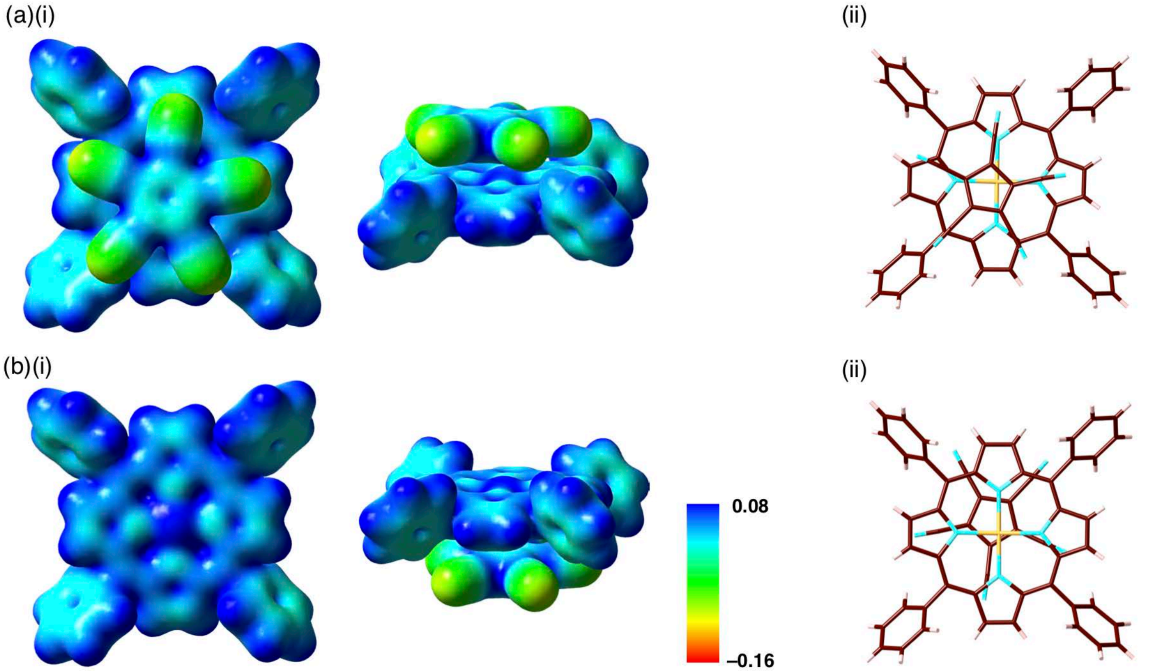

Figure S58 Electron density diagrams of Au0-PCCp⁻, Related to Figure 5.

(i) Electron density diagrams (top and side perspective views) of two types of stacking ion pair (a,b) **Au0**$^{+}$-PCCp^{-} observed in (ii) the crystal structure of **Au0**$^{+}$-PCCp^{-} (Figure S45,52). Electrostatic potentials were mapped onto the electron density isosurface (δ = 0.01) calculated at B3LYP/6-31+G(d,p) for C, H, and N and def2TZVP for Au. Small negative charge in **Au0**$^{+}$ was observed by stacking with PCCp^{-} compared to **Au0**$^{+}$-Cl^{-} (Figure S56,57). Atom color code: brown, pink, light blue, and light orange refer to carbon, hydrogen, nitrogen, and gold, respectively.

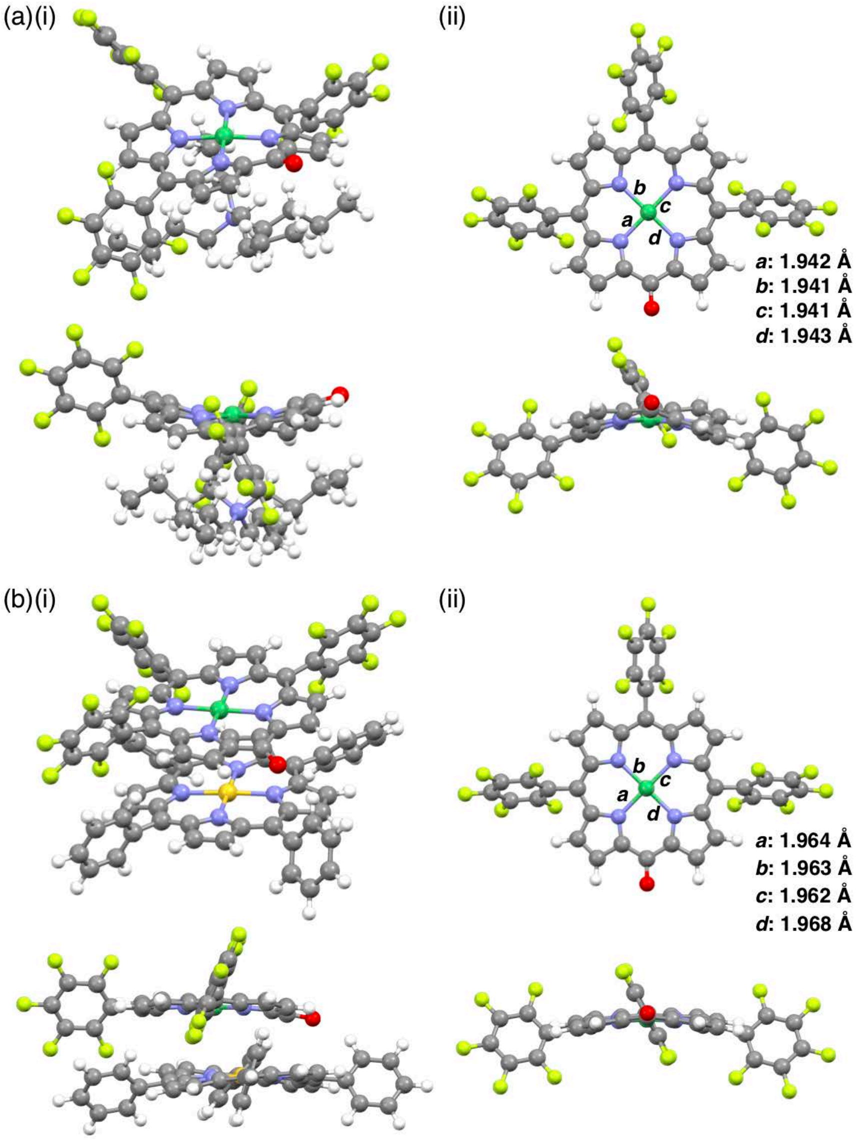

Figure S59 Optimized structures of TBA^{+}-NiO$^{–}$ and Au0^{+}-NiO$^{–}$, Related to Figure 11.
Optimized structures of (a) TBA^{+}-**NiO**$^{–}$ and (b) **Au0**$^{+}$-**NiO**$^{–}$ at B3LYP-GD3BJ with the 6-31G(d,p) basis set for C, H, N, O, F, and Ni and LanL2DZ for Au (optimized from the crystal structures of TBA^{+}-**NiO**$^{–}$ (Sasano et al., 2017) and **Au0**$^{+}$-**NiO**$^{–}$ (Figure S53b(i))), wherein (i) and (ii) show the side perspective and side views and the top and side views of **NiO**$^{–}$ with selected bond lengths, respectively. The mean-plane deviations of the 24-atom plane of **NiO**$^{–}$ in (a) and (b) were 0.41 and 0.17 Å, respectively. Longer Ni–N distance and planar structure of **NiO**$^{–}$ in **Au0**$^{+}$-**NiO**$^{–}$ than that of TBA^{+}-**NiO**$^{–}$ probably indicated the contribution of coordination of π-electron of **Au0**$^{+}$ to the Ni site. Atom color code: gray, white, blue, red, light green, green, and yellow refer to carbon, hydrogen, nitrogen, oxygen, fluorine, nickel, and gold, respectively. LanL2DZ basis set was used for Au atom due to the limited calculation resource.

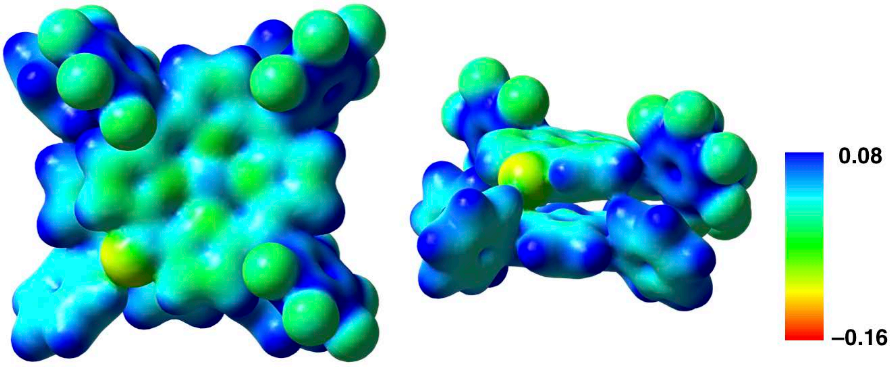

Figure S60 Electron density diagrams of $Au0^{+}$-NiO^{-}, Related to Figure 11.

Electron density diagrams (top and side perspective views) of **Au0**$^{+}$-**NiO**$^{-}$ as the optimized structure based on the single-crystal X-ray structure (Figure S53b(i)); see also Figure S59b). Electrostatic potentials were mapped onto the electron density isosurface (δ = 0.01) calculated at B3LYP-GD3BJ with the 6-31+G(d,p) basis set for C, H, N, O, F, and Ni and LanL2DZ for Au on the basis of optimized structure obtained by B3LYP-GD3BJ with the 6-31G(d,p) basis set for C, H, N, O, F, and Ni and LanL2DZ for Au. LanL2DZ basis set was used for Au atom due to the limited calculation resource. Effective delocalization of positive and negative charges for stacking was observed in **Au0**$^{+}$ and **NiO**$^{-}$, respectively.

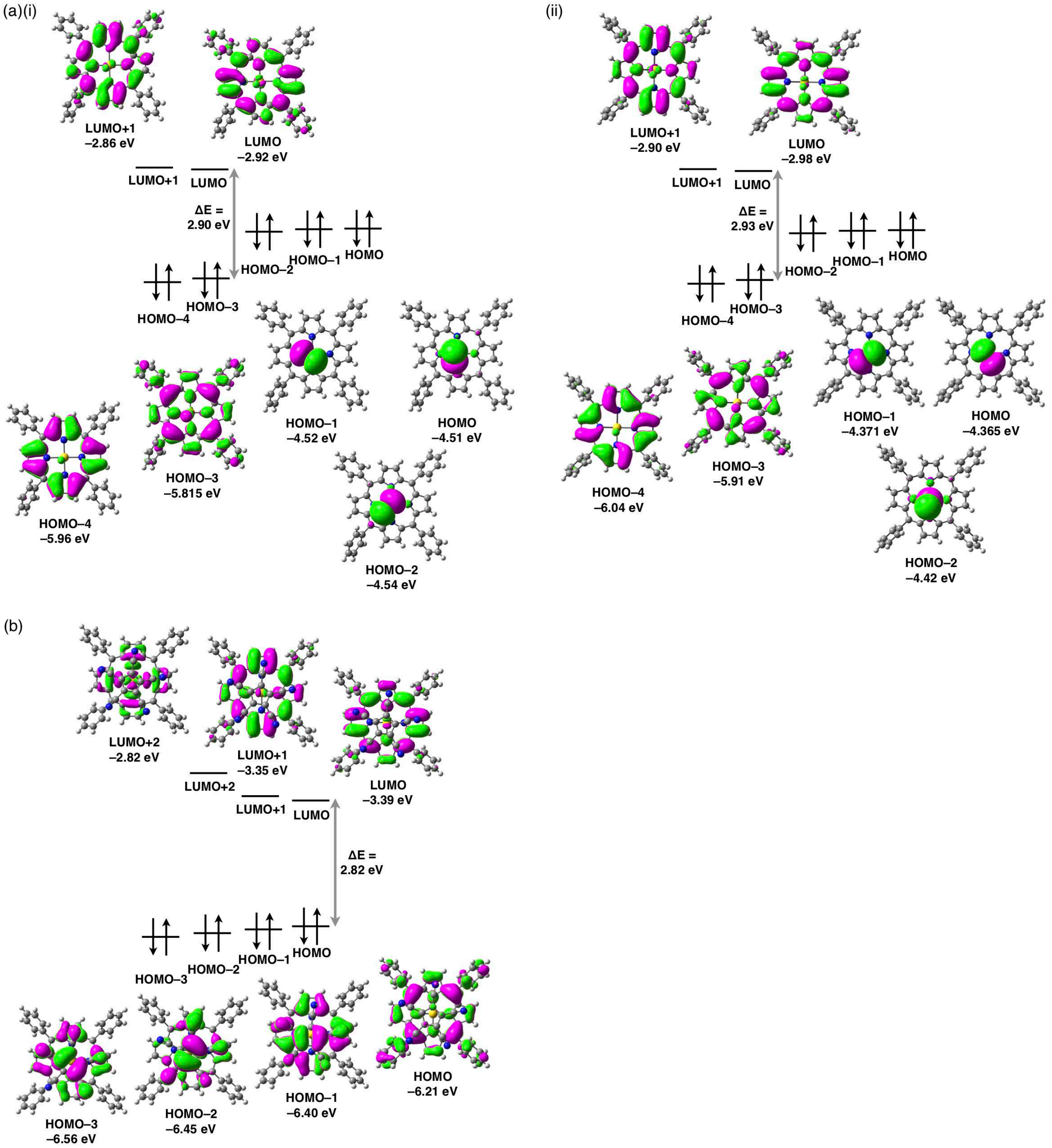

Figure S61 Molecular orbitals of **Au0**$^{+}$-Cl^{-} and **Au0**$^{+}$-PCCp^{-}, **Related to Figure 4 and 5.**
Molecular orbitals (MOs) of associating ion pairs in (a)(i) **Au0**$^{+}$-Cl^{-} (type A) and (ii) **Au0**$^{+}$-Cl^{-} (type B) and (b) **Au0**$^{+}$-PCCp^{-} found in the crystal structures (see also Figure S56–58) calculated at B3LYP/6-31+G(d,p) for C, H, N, and Cl and def2TZVP for Au. Separately localized MOs at **Au0**$^{+}$ and anions (Cl^{-}, PCCp^{-}) suggest that each ion exists as an independent species.

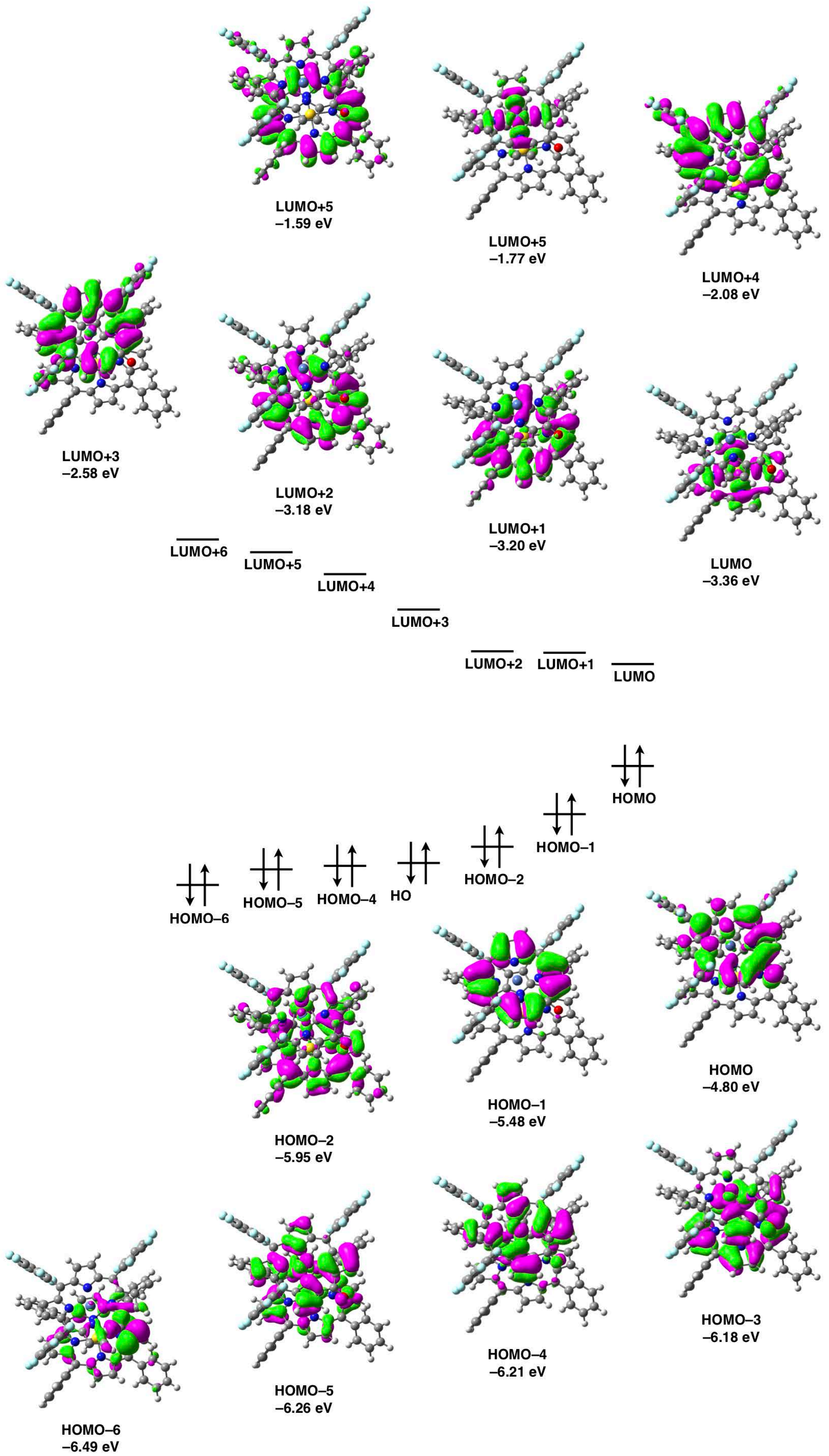

Figure S62 Molecular orbitals of **Au0⁺-NiO⁻**, **Related to Figure 12.**

Molecular orbitals (MOs) of the associating ion pair **Au0⁺-NiO⁻** calculated at B3LYP-GD3BJ level with the 6-31+G(d,p) basis set for C, H, N, O, F, and Ni and LanL2DZ for Au on the basis of optimized structure obtained by B3LYP-GD3BJ with the 6-31G(d,p) basis set for C, H, N, O, F, and Ni and LanL2DZ for Au (see also Figure S60). LanL2DZ basis set was used for Au atom due to the limited calculation resource.

Cartesian coordination of optimized structures

2H8 (AM1)
–1.0101567 hartree
C,1.313630469,2.3885532992,1.8251908904
C,0.92868797,3.5719776317,2.6550970288
C,-0.2756118482,4.0019289988,2.1842861222
C,-0.6515299428,3.0966882093,1.0591099052
C,-2.1776211111,2.31716543,-0.756818523
C,-3.3459713308,2.4500228494,-1.5713186928
C,-3.3298486766,1.4029814465,-2.5115228897
C,-2.1494090985,0.6301475858,-2.2765396921
C,-0.6035023185,-1.2599590692,-2.7984737461
C,-0.1844409566,-2.4542678534,-3.5882032763
C,1.012111577,-2.8659385898,-3.0822582971
C,1.3495192995,-1.9274086903,-1.9674813514
C,2.902203917,-1.2027230151,-0.1673031085
C,4.1567947988,-1.273594808,0.636725286
C,4.1526368665,-0.2469857893,1.5287435713
C,2.8940486872,0.531954949,1.3431751685
C,-1.7446483387,-0.5390738193,-3.0125682718
C,2.5580099757,-2.0511188569,-1.1870146756
C,2.5359481582,1.6519829937,2.046388587
C,-1.8009975486,3.1891619766,0.3259900963
C,3.4353210765,2.1496358262,3.0964383938
C,3.6783881927,1.363706485,4.2327456446
C,4.0423608877,3.3978980605,2.9631824578
C,4.5311463839,1.8339509335,5.2339362901
C,4.9159523674,3.8666082459,3.9621070191
C,5.1675703468,3.0924356802,5.1077709903
C,4.0150443988,-0.0063136881,6.6585218544
C,5.7963718288,5.9237590361,4.8084618737
C,7.210011845,2.9509875197,6.2802565408
C,4.4081184779,-0.4662899237,8.0516226272
C,5.8451440706,7.3390977165,4.2602424936
C,7.9318182334,3.68506605,7.3953863147
C,3.5949859783,-1.6778594172,8.45102145
C,6.2129721125,8.312425501,5.3583916277
C,9.2909277175,3.0663746693,7.6373980206
C,3.9724850059,-2.1516738856,9.8383494479
C,6.2622643117,9.7314009345,4.8334330222
C,10.0270797987,3.7865685266,8.7468589935
C,3.1609036652,-3.3635833426,10.2431320173
C,6.6308209784,10.7087748697,5.9289325586
C,11.3875763651,3.1702002707,8.99251098
C,3.5380457201,-3.8381952877,11.6299953462
C,6.6798504196,12.1278644153,5.404884956
C,12.1241758828,3.8897353078,10.1018050182
C,2.7269970146,-5.0500980256,12.0370016071
C,7.0484244623,13.1070036758,6.4992217261
C,13.4852642669,3.2745029002,10.348928509
C,3.4626889857,-3.1590537991,-1.5234357918
C,3.6844827114,-4.1804885962,-0.58750294
C,4.1050332705,-3.1881722457,-2.7605724047
C,4.5453350846,-5.2350512828,-0.8995624019
C,4.9702521972,-4.2512125907,-3.0793539125
C,5.199407271,-5.2845940513,-2.1550059084
C,4.1686485255,-6.259740052,1.2221828021
C,6.8200289928,-4.7331019997,-4.5169473466
C,5.5529096292,-7.5650143509,-2.6489410692

C,4.6364588982,-7.5079681427,1.9504128807
C,7.3943440288,-4.03792088,-5.7387822608
C,6.7064967502,-8.4798302104,-3.0175451723
C,4.0038517446,-7.5805944733,3.3226540079
C,8.7736557916,-4.5774803836,-6.046969803
C,6.2044276415,-9.8859974211,-3.260703026
C,4.4591357403,-8.8186509585,4.0653782936
C,9.3667022108,-3.8935099464,-7.2602215419
C,7.3419329363,-10.8137749764,-3.6305888632
C,3.829464689,-8.8956089567,5.4397251811
C,10.7468051102,-4.4309887748,-7.5727060249
C,6.8437909965,-12.2221878292,-3.8743698254
C,4.2842493084,-10.1331722787,6.1830253435
C,11.3405868061,-3.7472779679,-8.7854744237
C,7.9806822073,-13.1504027915,-4.2441515419
C,3.6558574818,-10.2118165256,7.5582005419
C,12.7210705232,-4.2836283663,-9.0996865238
C,7.4842748661,-14.5596957973,-4.4883131099
C,-2.6661601778,-0.9680595395,-4.0812072386
C,-3.2994597392,-2.2155232702,-3.983547497
C,-2.8938183755,-0.1437978227,-5.1816179116
C,-4.1724892263,-2.6305876729,-4.9918017059
C,-3.773215698,-0.5598066453,-6.198755579
C,-4.4224079488,-1.8033010245,-6.1143424096
C,-4.5567134176,-4.7149110716,-3.8958938659
C,-4.189576763,-0.1685079537,-8.5207700889
C,-6.6044877192,-2.3119674323,-6.8570737802
C,-5.3760505053,-5.9677446602,-4.1530192729
C,-3.7474906313,0.9186458927,-9.4843364058
C,-7.294911476,-2.7309029544,-8.1421037995
C,-5.1385095299,-6.9814828513,-3.0554921049
C,-4.0074341532,0.4892480125,-10.9114641263
C,-8.7882168419,-2.8374553784,-7.9247695004
C,-5.9463936659,-8.2392246809,-3.2944925085
C,-3.5689196792,1.5582422086,-11.8894543007
C,-9.4949147256,-3.254291675,-9.1969094713
C,-5.7120952518,-9.2571291309,-2.1989443233
C,-3.8279211684,1.1330458506,-13.3189104875
C,-10.9895812585,-3.3625033482,-8.9836720878
C,-6.5195050185,-10.5150190376,-2.4370357156
C,-3.3894103632,2.2012725668,-14.2974026963
C,-11.6968351438,-3.7793686855,-10.255230484
C,-6.2865555152,-11.5345154535,-1.3422624336
C,-3.6477354768,1.7778299588,-15.727822684
C,-13.1920298352,-3.8881853148,-10.0438184088
C,-2.7646725004,4.2596642515,0.6408654391
C,-4.0582094197,3.9212165833,1.0642636301
C,-2.3885969268,5.5960838908,0.5179280551
C,-4.9721171952,4.9302677545,1.3756857993
C,-3.3074549309,6.6148050132,0.832559502
C,-4.6048711511,6.2943769927,1.2663565191
C,-6.6847895873,3.3337185113,1.8337426259
C,-3.7284713532,8.9100226086,0.3120135954
C,-5.8984223561,7.471166147,2.8518959712
C,-8.1407563042,3.36414474,2.2652364597
C,-2.8823246819,10.0010248566,-0.3200576875
C,-6.8415052294,8.6598202515,2.88663437
C,-8.6979507889,1.9587954208,2.3219538118
C,-3.7558821825,11.1627038021,-0.7396436592
C,-7.2989739055,8.9253736995,4.3039381927

C,-10.1502432547,1.967772931,2.7491142727
C,-2.9314997827,12.2606249936,-1.3771886932
C,-8.2410978049,10.1089645151,4.3589470041
C,-10.7127215857,0.5636749695,2.8072460631
C,-3.8016518756,13.4255462359,-1.7979175488
C,-8.7016232598,10.3778899811,5.7755639371
C,-12.1647874232,0.5716099811,3.2342385339
C,-2.9781353603,14.5236287244,-2.4357895238
C,-9.6437499708,11.5611610255,5.8313377264
C,-12.7293203011,-0.831962144,3.2929943083
C,-3.8467863269,15.6898006967,-2.8572458031
C,-10.1054920862,11.8316029921,7.247599775
H,1.5402842542,3.9638109789,3.4636950204
H,-0.8934071154,4.8339140273,2.5149653801
H,-4.0932927647,3.2325826046,-1.467533058
H,-4.0640498906,1.2018199673,-3.2877896526
H,-0.7695469905,-2.8630540506,-4.4087480951
H,1.6475164235,-3.6943587621,-3.3849266629
H,4.9059698199,-2.0466673328,0.4702250537
H,4.8977672044,0.0293594093,2.2737385993
H,-0.6126900436,0.8797158494,-0.8278249227
H,1.3491143116,0.2490928203,-0.0920138508
H,3.1840711332,0.3866082125,4.3242159988
H,3.8576258032,4.024181738,2.0777753867
H,2.9377588904,0.3019203328,6.6220089658
H,4.2042176782,-0.8102778823,5.8993603356
H,6.7855590099,5.6152337391,5.2404390452
H,5.0014572934,5.8109762534,5.5894210448
H,7.0404182359,1.8735351983,6.5403225464
H,7.7730980742,3.0240057645,5.3138007148
H,4.2429770822,0.3703538776,8.7795517348
H,5.5023520757,-0.7097966789,8.068625906
H,6.5952589012,7.3908469324,3.4284829834
H,4.8476900361,7.6057819886,3.8236441297
H,8.0416384658,4.7664245451,7.1190950884
H,7.3134806324,3.6419774383,8.3296091313
H,2.5020219707,-1.4250525951,8.424475354
H,3.7619404859,-2.5060277804,7.7125433657
H,7.2113043792,8.035832743,5.7895643167
H,5.4627556714,8.244489972,6.1900692816
H,9.8981674495,3.1072297465,6.6946894152
H,9.1698963147,1.9839069054,7.9070172039
H,3.8061894005,-1.3233521815,10.5766708763
H,5.0653589217,-2.4041218705,9.865224045
H,7.0115283036,9.7985950646,4.0010162363
H,5.263890555,10.0077958773,4.4024892251
H,10.1474069982,4.8690635293,8.4775086339
H,9.4195506266,3.7460400024,9.689248
H,2.0680984287,-3.1107091962,10.2163684358
H,3.3266552585,-4.1915187032,9.5042941901
H,7.6292060632,10.4322074033,6.359721079
H,5.881637841,10.640885252,6.7613844163
H,11.9947483444,3.2107093289,8.0498728297
H,11.2671085032,2.0876276462,9.2615242654
H,4.6307059149,-4.0914438105,11.6570801392
H,3.3725084851,-3.0104071555,12.3690270603
H,7.4289016414,12.1960058934,4.5723855749
H,5.6815201849,12.404697333,4.9742032256
H,12.2449208729,4.9722910421,9.8329655487
H,11.5172803832,3.8493769584,11.044578071

H,1.6345365232,-4.7951571859,12.0083096515
H,2.8921980113,-5.8762319147,11.2957404248
H,8.0463198226,12.8275837768,6.9297997305
H,6.2990009564,13.0363301082,7.3314733273
H,14.0901664367,3.3144984225,9.4045905649
H,13.3626302556,2.1915099542,10.6160514266
H,3.1684934908,-4.1368233109,0.3815875333
H,3.9391948867,-2.3928005233,-3.5022996457
H,4.4755448545,-5.3320613868,1.7724255166
H,3.0559451657,-6.2655992773,1.0813522282
H,6.7417474429,-5.8420973903,-4.6727875073
H,7.4330341652,-4.5316475484,-3.6014884789
H,5.0473097731,-7.9013623529,-1.7063107594
H,4.803000126,-7.500657866,-3.4794583196
H,5.7541601886,-7.4909546434,2.0374532347
H,4.3643633712,-8.4138107273,1.3483964392
H,6.7111227685,-4.1968811777,-6.6134862587
H,7.4425465985,-2.9335250742,-5.5526684745
H,7.2154878768,-8.0856221135,-3.9358444807
H,7.4632646648,-8.4751661082,-2.1903421594
H,4.2763863598,-6.6663621066,3.9134396279
H,2.8861171823,-7.5909016487,3.2241318549
H,8.7147238601,-5.6830413745,-6.2295639537
H,9.4458258352,-4.4234172544,-5.1617695386
H,5.4423759283,-9.8781797682,-4.084305976
H,5.6919090425,-10.268181256,-2.3385716786
H,5.5768208778,-8.8088321476,4.163137125
H,4.1868898603,-9.7329024306,3.4747283295
H,8.6936910254,-4.0466836427,-8.1447674309
H,9.4256770053,-2.7880938254,-7.0773811816
H,7.8548003813,-10.4311688029,-4.5522204094
H,8.1040362064,-10.8210881361,-2.8071934184
H,4.1015871801,-7.9811310118,6.0300935674
H,2.711803327,-8.9051821623,5.3417128284
H,10.687630016,-5.5364021991,-7.7554610334
H,11.4193706227,-4.2780540732,-6.687760467
H,6.0815818041,-12.2144760887,-4.6976810789
H,6.3306613606,-12.6045477386,-2.9527730893
H,4.0119957311,-11.0478803144,5.5931338485
H,5.4018798828,-10.1239849967,6.2810509698
H,10.6681870868,-3.8999839127,-9.6705362218
H,11.4000377587,-2.6418785355,-8.6028665806
H,8.4938742042,-12.7683676141,-5.1658160027
H,8.7430482355,-13.1583437779,-3.4210424082
H,3.9282942307,-9.2957128182,8.146249119
H,2.5381628176,-10.2190166884,7.4582707032
H,12.6598600065,-5.3894475396,-9.2804677063
H,13.3916109951,-4.1313516713,-8.212858808
H,6.7209244502,-14.549483832,-5.3107917205
H,6.9699417405,-14.9394945369,-3.5660979058
H,-3.0981589757,-2.8503165834,-3.1091056481
H,-2.4016960186,0.8366629755,-5.2674377315
H,-3.4587168704,-4.9425326359,-3.8953893908
H,-4.8448587982,-4.2346897594,-2.9240618787
H,-5.283312881,-0.3965568158,-8.6303005916
H,-3.6002394489,-1.1102796465,-8.662642656
H,-6.7646625396,-3.0638626981,-6.0408210287
H,-6.9539871518,-1.3026631024,-6.5173849581
H,-5.0937665376,-6.3989034652,-5.1487838638
H,-6.4629883386,-5.6977470812,-4.2042814159

H,-4.302174649,1.8661892484,-9.2570238044
H,-2.6566208466,1.1295746331,-9.3353667188
H,-7.0725099689,-1.9810317991,-8.9458353652
H,-6.8806457124,-3.715547902,-8.4824883992
H,-4.0473101356,-7.2388211459,-3.0082494928
H,-5.4180033379,-6.53780807,-2.0634392464
H,-5.1009244147,0.2787425538,-11.0494525599
H,-3.4569407319,-0.4649812597,-11.1248656731
H,-9.1910771862,-1.8495909942,-7.5770127361
H,-8.9990752678,-3.5850270664,-7.114984998
H,-5.6671572737,-8.6826560751,-4.2865969714
H,-7.037439623,-7.9820409018,-3.3425191203
H,-4.1185346456,2.5125570305,-11.6749018249
H,-2.4754724717,1.7684294968,-11.7514252949
H,-9.2833996314,-2.5071561332,-10.0067705824
H,-9.0916416971,-4.2418266517,-9.544735496
H,-4.6209509834,-9.5140082973,-2.1510728826
H,-5.9909152814,-8.8133340547,-1.2068938793
H,-4.9213702053,0.9227629204,-13.456711666
H,-3.2784539338,0.1785026237,-13.5329164779
H,-11.3924874734,-2.3748711362,-8.6356591271
H,-11.2009260205,-4.1094307708,-8.1735626924
H,-7.6106932351,-10.2585279579,-2.4849955493
H,-6.240750428,-10.9591244246,-3.4289327111
H,-3.9386415889,3.1559414719,-14.0835662284
H,-2.2959467331,2.4115636125,-14.1599097725
H,-11.4857853876,-3.0325312676,-11.0654612081
H,-11.2941659056,-4.7669690941,-10.6034807313
H,-5.1945687132,-11.7886760633,-1.2948628508
H,-6.5643582605,-11.0880843671,-0.3509010099
H,-4.741491212,1.56642709,-15.8629952712
H,-3.0988301208,0.8221798014,-15.9392778925
H,-13.5926334994,-2.8999778,-9.6941874725
H,-13.4010985708,-4.6343017714,-9.2321113604
H,-4.3339943533,2.8599562624,1.1429781978
H,-1.3778345014,5.8722960778,0.1822683545
H,-6.5761358832,2.8994587613,0.8056411812
H,-6.059208405,2.7509027776,2.5597670019
H,-4.2783823368,9.286184943,1.2155483341
H,-4.4644027447,8.484196038,-0.4169976167
H,-6.4013650963,6.5370657617,3.2149126376
H,-4.9758244561,7.665543715,3.4579110736
H,-8.7297821891,3.9874346495,1.5429683201
H,-8.2212407849,3.8550753195,3.2699536455
H,-2.1071298829,10.3437646508,0.4139429611
H,-2.3375943722,9.5851980326,-1.2072109995
H,-6.3193379893,9.5616743753,2.4722129123
H,-7.7235324516,8.4536931761,2.2259185363
H,-8.6054431606,1.4745070265,1.3139418774
H,-8.0978042879,1.3424904777,3.042457406
H,-4.2979105904,11.5700454308,0.1544200935
H,-4.5344161008,10.8068510034,-1.4651757092
H,-6.4086964753,9.1224732375,4.9578975064
H,-7.8140492736,8.0152646824,4.7107328439
H,-10.7502372272,2.5843275786,2.0288775856
H,-10.2430139971,2.4526263352,3.7566563924
H,-2.1522777269,12.6152037958,-0.651994266
H,-2.3898196488,11.8531349724,-2.2712772133
H,-7.7262702975,11.018754229,3.9514462646
H,-9.1309879454,9.9118758826,3.7046608493

H,-10.6196101158,0.0790228236,1.7996164682
H,-10.1123042606,-0.0528106545,3.5271791284
H,-4.3433852958,13.8327901902,-0.9037612802
H,-4.5809572072,13.0704492064,-2.5228022257
H,-7.8114897569,10.5746761616,6.4296279299
H,-9.2161692857,9.4679035618,6.1830122966
H,-12.2582663371,1.0562015084,4.2418156625
H,-12.7654531586,1.1878935849,2.5143860826
H,-2.1986784273,14.8788306642,-1.7111871313
H,-2.4365113272,14.1166452082,-3.3301036425
H,-9.1294122445,12.4713144603,5.4240829533
H,-10.5339926182,11.3646472728,5.1774061873
H,-12.6335989092,-1.3154745084,2.2848612527
H,-12.1263968783,-1.4472220278,4.0121684332
H,-4.3888639877,16.0944553479,-1.9618856138
H,-4.6265510697,15.3322724741,-3.5807234627
H,-9.2137199947,12.0264304525,7.9003349383
H,-10.6182041093,10.9197767892,7.6537628701
N,0.381554065,2.1271041717,0.9013400115
N,0.3952061978,-1.0014466777,-1.8152495371
N,-1.4701042028,1.2064427742,-1.2065565039
N,2.1706383551,-0.1125303333,0.3276424508
O,4.8570993692,1.119131065,6.3683638386
O,5.5201388281,5.0734120711,3.6820263071
O,5.934798631,3.6068798564,6.1382284771
O,4.8143256085,-6.2945668068,-0.058436971
O,5.4882794137,-4.2141912717,-4.3562157046
O,6.1356462876,-6.2652150091,-2.4318223323
O,-4.8646555578,-3.8230382082,-4.9773265232
O,-3.9799815126,0.3637394374,-7.2007442853
O,-5.1989388441,-2.2487950101,-7.1691112519
O,-6.2544145719,4.7030529005,1.8286745514
O,-2.8058040197,7.8960060656,0.7481867797
O,-5.5321692027,7.3009762308,1.4684746849
C,-7.0885101689,-12.7887557992,-1.5751438701
H,-6.8078054026,-13.2616460195,-2.5473016391
H,-6.902195873,-13.5261571385,-0.7574888762
H,-8.18128449,-12.5592152747,-1.6008404602
C,-13.9004194355,-4.3030579936,-11.3073622792
H,-15.0003719957,-4.3771721182,-11.1294884541
H,-13.7240965445,-3.5585249031,-12.1210523432
H,-13.5319845066,-5.2975095382,-11.6578287104
C,-3.212590272,2.838464707,-16.7056711511
H,-3.4117243918,2.5063338651,-17.7530739947
H,-3.7667949993,3.7914773656,-16.526419405
H,-2.1197819862,3.0451678179,-16.6029141672
C,-14.1750324348,-0.8290146629,3.7177191768
H,-14.571074657,-1.8724868602,3.7544265612
H,-14.2875350622,-0.3731972174,4.7311683385
H,-14.7960528392,-0.2411148038,2.9992356423
C,-11.0438378442,13.0090315517,7.3079845398
H,-11.3717468459,13.1907738021,8.3598746247
H,-10.5423859141,13.9328172853,6.9302602271
H,-11.9506479682,12.8232354752,6.6829397841
C,-3.0310085527,16.7856260743,-3.4929787089
H,-3.6901139718,17.6346395878,-3.7959224415
H,-2.2643345123,17.1718120129,-2.778432121
H,-2.5027556545,16.4075753089,-4.4015723721
C,3.09949915,-5.5264649679,13.4171479584
H,2.4903970735,-6.4195488898,13.6971985806

H,4.1793434442,-5.808754765,13.4590001295
H,2.9183223372,-4.7247378674,14.1734038748
C,14.222375383,3.9877555397,11.4527462232
H,15.2221569005,3.5186765871,11.6183670978
H,14.3759780399,5.063291519,11.1936338624
H,13.6464466742,3.9373802995,12.4083988487
C,7.098486834,14.5213599883,5.9819457537
H,6.1056975911,14.8264141661,5.5710638957
H,7.3716139542,15.2261519613,6.8039477299
H,7.857694514,14.6171453931,5.1683496689
C,13.316247077,-3.6056356329,-10.3065732762
H,14.3327875565,-4.0154301041,-10.520424827
H,12.6743897634,-3.7670869926,-11.2062732352
H,13.4081145727,-2.5056807861,-10.1357678258
C,8.6129494704,-15.4874778643,-4.8568126971
H,8.2254169659,-16.5199790805,-5.0322766876
H,9.1220997856,-15.1389019808,-5.7878185201
H,9.3718959031,-15.5299104576,-4.0384581922
C,4.1054962293,-11.4428038122,8.3018270247
H,5.2146907434,-11.4413383915,8.4329444892
H,3.6310250332,-11.4811075499,9.3119983087
H,3.8208510909,-12.3671348932,7.7431726869

2H12 (AM1)
–1.5362474 hartree
C,3.0903548729,-0.5514109989,-1.1354572359
C,3.9680948071,-0.1654828033,-2.2834487116
C,3.1612988704,-0.0795924146,-3.3783006063
C,1.7779988963,-0.4097459964,-2.9267697148
C,-0.6706715474,-0.723520393,-3.3006673738
C,-1.817171438,-0.8246205319,-4.1500047312
C,-2.9233204364,-1.1316546628,-3.3359268152
C,-2.4546823041,-1.2229196001,-1.9876342887
C,-2.8020183311,-1.6342576324,0.450590699
C,-3.650114834,-1.9447219071,1.6379206437
C,-2.8188055954,-2.0039489152,2.7161971764
C,-1.4389657166,-1.7321637971,2.2066664064
C,0.9968591498,-1.4747718588,2.6278428101
C,2.22388041,-1.4937942993,3.4761031153
C,3.2965605643,-1.2254501623,2.6844617148
C,2.8110607366,-1.0171142473,1.2896144925
C,-3.2616243038,-1.5091856977,-0.8302788065
C,-0.2797920472,-1.7141962173,3.0671153802
C,3.5975228213,-0.7263981156,0.2054908794
C,0.6716486015,-0.4242851709,-3.728808242
C,5.0452986814,-0.572890873,0.4017441891
C,5.5262292289,0.4386386959,1.2473634697
C,5.935974244,-1.4274273923,-0.2466675638
C,6.9009072433,0.588275685,1.4418631296
C,7.3215330975,-1.2872897874,-0.042743899
C,7.8186963994,-0.2795786712,0.8011224893
C,6.5749057729,2.5238664787,2.7982002748
C,9.3941908598,-1.8070878773,-1.1186201623
C,9.712458923,-0.4304402586,2.2004327094
C,7.4551080838,3.5188324724,3.5346584768
C,9.7696552605,-2.7336703305,-2.2616814392
C,11.2119500172,-0.2151356614,2.107625301
C,6.6056647547,4.5774401951,4.2032323232
C,11.1637791172,-2.415773901,-2.7555989902
C,11.8732347843,-0.5515515166,3.4259729585

C,7.4724199028,5.6231476435,4.8717597174
C,11.5560686183,-3.3259154251,-3.8998279235
C,13.3708209564,-0.3433617144,3.3524719381
C,6.6330569951,6.6855848922,5.5489973571
C,12.9509158714,-3.0117530999,-4.3967401058
C,14.0363633361,-0.6785535145,4.6700103947
C,7.5025712085,7.7893054641,6.1121974034
C,13.3436024777,-3.921222704,-5.5412207205
C,15.5338579198,-0.4702749266,4.5972015431
C,6.6709452097,8.8685769262,6.7718425591
C,14.7384039058,-3.6075781777,-6.038504059
C,16.1998146717,-0.8053282909,5.9145392888
C,7.5367874192,10.0279382892,7.2168508512
C,15.131122912,-4.5169965853,-7.1829950901
C,17.6972888242,-0.5969566825,5.8418545683
C,6.7060900067,11.1410429866,7.8182942408
C,16.5259002391,-4.2035111249,-7.6804322507
C,18.3633857974,-0.9319883173,7.1591216857
C,7.5717176471,12.3198064945,8.2090897734
C,16.9186832458,-5.1128125362,-8.8248494563
C,19.8607595374,-0.723634683,7.0865914988
C,6.7435893442,13.4409290745,8.8001970082
C,18.3136429661,-4.8003668725,-9.3234384282
C,20.5283345071,-1.0584035739,8.4035592696
C,-0.4955261444,-1.9592500195,4.4992817464
C,-0.1621410413,-0.9570507679,5.4234505059
C,-1.0071484143,-3.1782479346,4.9416282781
C,-0.3395755346,-1.1813977047,6.7901628081
C,-1.2027417995,-3.4014639335,6.3174913338
C,-0.869080887,-2.4094942582,7.2556046229
C,0.6375710128,0.9243994025,7.3265636499
C,-1.3735735054,-5.2166356792,7.8659499417
C,-1.9979973001,-1.9422697647,9.2741378724
C,1.0242108504,1.6811288004,8.5851935801
C,-1.6088010174,-6.7074140517,7.6978549005
C,-1.9909678472,-2.4197697608,10.7147498112
C,1.7252174792,2.9708268242,8.2179495462
C,-1.278710064,-7.4365743188,8.9815880371
C,-3.0532264047,-1.696347624,11.512739566
C,2.2225156074,3.6926530439,9.4518138412
C,-1.5021560378,-8.9264472475,8.8337491955
C,-3.0617765735,-2.1601523483,12.9536826665
C,2.8830152095,5.0054816026,9.0882125499
C,-1.1737987951,-9.6599695758,10.1164597314
C,-4.1232155525,-1.438251164,13.7558140481
C,3.4534604792,5.6886701689,10.3122530211
C,-1.3966645645,-11.149870301,9.9691495882
C,-4.1320589578,-1.9014288654,15.1968409615
C,4.0553391964,7.0331279599,9.9638943907
C,-1.0686134578,-11.8838922057,11.2516200244
C,-5.1933172508,-1.1797488612,15.9993660244
C,4.6633512052,7.6908440475,11.1841271058
C,-1.2914220441,-13.3737904542,11.1043460714
C,-5.2021536979,-1.6428010664,17.4404195224
C,5.2294126132,9.0543104076,10.8509448746
C,-0.9634616649,-14.1079982877,12.3867276005
C,-6.2633646783,-0.921208363,18.2430773954
C,5.8622568576,9.6966775966,12.0666592112
C,-1.186215309,-15.5978038612,12.239558451
C,-6.2722785514,-1.3841210541,19.6840607522

C,6.3236143382,11.10730033,11.7687035607
C,-0.8585558915,-16.3335029667,13.521553958
C,-7.3333279791,-0.6631506855,20.4881175495
C,-4.7051119554,-1.6715129939,-1.0862334914
C,-5.6096145686,-0.7559272475,-0.5292571273
C,-5.1625324564,-2.7327682944,-1.8650355884
C,-6.9784810684,-0.9030497255,-0.7651497446
C,-6.5413726186,-2.8828216105,-2.1044681527
C,-7.4619862577,-1.970896908,-1.5607033651
C,-7.4864386512,0.9895817201,0.596092234
C,-8.1182039688,-4.5894066379,-2.6696750993
C,-9.4636056236,-1.2177646901,-2.5597703594
C,-8.7347184131,1.7324232241,1.0399315817
C,-7.9700808608,-5.9951416914,-3.224452833
C,-10.9096309862,-1.663944299,-2.6756497278
C,-8.3647913784,2.8642370996,1.9731054216
C,-9.2639781066,-6.7612912054,-3.0586517589
C,-11.6874819423,-0.7017612809,-3.5461079507
C,-9.5968838832,3.6170423712,2.428022563
C,-9.136135336,-8.1684936101,-3.6017448804
C,-13.1335492776,-1.1308107877,-3.6738764698
C,-9.2320692859,4.7507665657,3.362279867
C,-10.4290250206,-8.9385826265,-3.4385784848
C,-13.9156266906,-0.1703123131,-4.543939618
C,-10.463504273,5.5040594909,3.8176137994
C,-10.3017719445,-10.3459064175,-3.9811848367
C,-15.3617652051,-0.5986726368,-4.6718597831
C,-10.0993924329,6.638002009,4.7518268874
C,-11.5944119342,-11.1164385244,-3.8183988693
C,-16.1442559723,0.3615498694,-5.5418140663
C,-11.3307027413,7.3913902023,5.2072639544
C,-11.4672224979,-12.5237599806,-4.3609842724
C,-17.5904204409,-0.0666843898,-5.6696980489
C,-10.9668247477,8.5254254012,6.1414419209
C,-12.759771484,-13.2944586634,-4.1983110872
C,-18.3730511604,0.8934354917,-6.5396312603
C,-12.1979553136,9.2788490921,6.5969366029
C,-12.6326990672,-14.7016960054,-4.7408368082
C,-19.8191435948,0.4653554859,-6.6675575953
C,-11.8354847933,10.4135133318,7.5314358078
C,-13.9248820361,-15.4737879272,-4.5787765971
C,-20.6031855172,1.4248506804,-7.5374876567
C,0.8233643133,-0.1094185136,-5.1610505345
C,0.1506444172,0.997140003,-5.6996709444
C,1.6303235323,-0.9077823829,-5.9698431052
C,0.2996534031,1.3079988925,-7.0530055598
C,1.783121929,-0.595743303,-7.3337784334
C,1.1237565342,0.5140359201,-7.8885449718
C,-1.222631059,3.136136137,-6.8722858601
C,2.3359166403,-1.6720925759,-9.3958225238
C,1.9333048173,1.9381553367,-9.5881593661
C,-1.8048339159,4.1885813286,-7.7998388586
C,2.9808105668,-3.006159857,-9.7276999843
C,1.9789689522,1.9798017323,-11.1046333645
C,-2.7957391719,5.0530544626,-7.0518533949
C,2.7673838708,-3.3409239657,-11.1875052904
C,2.7317609445,3.2054074209,-11.5733990872
C,-3.3892277249,6.1084458177,-7.9604922028
C,3.3992460324,-4.6709900989,-11.5384756489
C,2.7881696512,3.2643023791,-13.0849761393

C,-4.3817348322,6.9761717737,-7.2166912379
C,3.1887917268,-5.0095065483,-12.9987291684
C,3.5404007765,4.4896090075,-13.5582472976
C,-4.9757903512,8.0316795556,-8.1245501792
C,3.8201182605,-6.3395476493,-13.350284698
C,3.5969663046,4.5491865363,-15.0696779583
C,-5.9683548515,8.8997955538,-7.3813497311
C,3.6101209972,-6.6784886964,-14.8104756655
C,4.3490832242,5.7743549825,-15.543409734
C,-6.5625608429,9.9553047232,-8.2890718907
C,4.2414121101,-8.0085119551,-15.1620989729
C,4.4056316711,5.8340562769,-17.0548224327
C,-7.5551353356,10.823563421,-7.5460690782
C,4.0315552366,-8.3476091258,-16.6222684994
C,5.1577273582,7.0591719424,-17.5287085829
C,-8.1494000532,11.8790299534,-8.4536173941
C,4.6627689668,-9.6775211661,-16.9739803917
C,5.2143256279,7.1190021628,-19.0400049712
C,-9.1424451499,12.748287519,-7.71173657
C,4.4536259871,-10.0179966623,-18.434275309
C,5.9663623801,8.3439723717,-19.5154191991
H,5.0385983955,0.0003676534,-2.1940705507
H,3.4111443467,0.1734143769,-4.4063068242
H,-1.8082342307,-0.6910437091,-5.2287743671
H,-3.9528206861,-1.2831171577,-3.6510979368
H,-4.7263049632,-2.0909796796,1.581380788
H,-3.0477854018,-2.2067434759,3.7593344336
H,2.1855759397,-1.706004852,4.5438857211
H,4.3526944513,-1.1648003791,2.9443088633
H,-0.496170337,-0.9752618093,-1.1967677349
H,0.8339401231,-1.2004276349,0.5244128335
H,4.808151704,1.1089507069,1.7398634014
H,5.571443904,-2.2238942158,-0.9123976489
H,6.0203479386,3.0178169164,1.9582111043
H,5.8462650395,2.0339296883,3.4962997239
H,10.1075087949,-1.9057186156,-0.2573889431
H,9.3600939504,-0.7373549232,-1.4488101301
H,9.2503246998,0.2304931922,2.9794704156
H,9.4678768579,-1.5023467607,2.4185570059
H,8.1663225403,3.9920489063,2.8083480328
H,8.0723673802,2.9773331466,4.2980711456
H,9.7149410726,-3.7976444233,-1.9121949613
H,9.0278487474,-2.6201309796,-3.0944390481
H,11.6302760858,-0.8574143637,1.2889566947
H,11.4176247359,0.8516851098,1.8308993457
H,5.9448794537,5.0690288864,3.4409396591
H,5.9349663124,4.0999473989,4.9652951594
H,11.8978198327,-2.5306718735,-1.9147286477
H,11.2104467454,-1.3463306186,-3.0921149952
H,11.6545798457,-1.6181792696,3.6972757979
H,11.4433883979,0.0919159593,4.2385331062
H,8.1376252899,6.1019976013,4.1050768307
H,8.140069458,5.1323496912,5.6279943336
H,11.5080359853,-4.3951573045,-3.563307683
H,10.8220256087,-3.2107771243,-4.7405320745
H,13.8004512242,-0.9862488396,2.5394899278
H,13.5892526386,0.7230706072,3.080728294
H,5.9077845138,7.1193348727,4.8107155365
H,6.0289221252,6.2226051762,6.3730948586
H,13.6848063927,-3.1269798665,-3.5559339779

H,12.9986881086,-1.9423185095,-4.7327091978
H,13.8177231454,-1.7450253738,4.9414636291
H,13.6063555616,-0.0359155865,5.4829921681
H,8.2216899794,7.3596330455,6.8582776766
H,8.1142634169,8.2401141418,5.2860901871
H,13.2956189239,-4.9906572324,-5.2053016789
H,12.6096996894,-3.8059339833,-6.3820178365
H,15.9638675529,-1.1129248918,3.7842240866
H,15.7525417251,0.5961548545,4.3256577759
H,5.8924887429,9.2362717715,6.0522133544
H,6.1281783774,8.4407350844,7.6567742185
H,15.4722943614,-3.7228238002,-5.1977042991
H,14.7863273673,-2.5381451075,-6.3744276134
H,15.9812354175,-1.8718126351,6.18596077
H,15.7697211983,-0.1628362943,6.7275853654
H,8.1132393941,10.4239876273,6.3391491542
H,8.2869444188,9.6690794349,7.9699510846
H,15.0831332978,-5.5864325113,-6.8470937998
H,14.3972132885,-4.4017437228,-8.023782241
H,18.1273811953,-1.2394824502,5.0288280841
H,17.9159133516,0.4694984577,5.5703790113
H,5.93001933,11.473231692,7.0793203409
H,6.1603875577,10.7587068504,8.7230379423
H,17.259780711,-4.3187152357,-6.8396093329
H,16.5738465911,-3.134057593,-8.0163024078
H,17.9332113695,-0.289559014,7.9721831222
H,18.1447714607,-1.9984735627,7.4305281674
H,8.3409783735,11.9890097629,8.9562854874
H,8.1248818695,12.6957801605,7.3083531669
H,16.1850416695,-4.9977004914,-9.665852939
H,16.8709264752,-6.1823161103,-8.4892193512
H,20.291202572,-1.3660831519,6.2737269898
H,20.0796883977,0.3427783963,6.8153053409
H,6.2248989274,13.0699058595,9.7251106106
H,5.9457607175,13.7420483319,8.0709968254
H,18.3598387934,-3.7299780171,-9.6572133419
H,19.0457003636,-4.914532686,-8.4806284339
H,20.09547798,-0.4160477882,9.2155220696
H,20.3070190454,-2.1248034289,8.6739066417
H,0.2309086677,-0.0004494543,5.0518048374
H,-1.2707502282,-3.9731313665,4.2281124141
H,1.5480616104,0.6333554031,6.7397875015
H,-0.0494484483,1.5330096389,6.6816502527
H,-2.005519624,-4.7902812855,8.6900585673
H,-0.2963420903,-4.989647649,8.0720523531
H,-1.7728604479,-0.8460777251,9.2035782869
H,-2.9788456956,-2.1607832244,8.7776436993
H,1.693628336,1.0384198926,9.2143449549
H,0.1056554955,1.896108795,9.1905862111
H,-2.6787191827,-6.8861731196,7.414150914
H,-0.9745501243,-7.0938558371,6.8581872579
H,-2.1723928352,-3.5261756765,10.7410986091
H,-0.9787524164,-2.2383433648,11.1613754657
H,2.5916131335,2.7504908559,7.5394199121
H,1.0206863845,3.6348847547,7.6507662266
H,-1.9170557847,-7.0412736616,9.815454273
H,-0.2097672702,-7.2421668847,9.262111866
H,-4.0606042444,-1.8780306742,11.0530809392
H,-2.8660189433,-0.5906502687,11.4744705364
H,2.9541720542,3.0410772329,9.9987203721

H,1.3643037906,3.8833240674,10.1487802
H,-2.5707224311,-9.1203226726,8.5519537998
H,-0.8636394424,-9.3215404994,8.0000509272
H,-3.2482501455,-3.2658651845,12.9917967705
H,-2.0542990141,-1.9788562686,13.4129819945
H,3.7029564114,4.8224848521,8.3444691511
H,2.133657169,5.679077218,8.5947449816
H,-1.81238281,-9.2647558877,10.9500329036
H,-0.1053042457,-9.4655206108,10.3981636951
H,-5.1305495163,-1.619706026,13.2962466489
H,-3.9368672667,-0.3325289094,13.7173001357
H,2.6442382864,5.8272394012,11.0771286392
H,4.2376742811,5.0339757499,10.77630519
H,-2.4650855725,-11.3443032094,9.6871820679
H,-0.7579794363,-11.5450662094,9.135634644
H,-4.3184341432,-3.0071506651,15.2353525628
H,-3.1247126163,-1.7201010251,15.6564119082
H,4.8423928045,6.9038494875,9.1740585251
H,3.2623459701,7.6994713905,9.5325760506
H,-1.7072555404,-11.4886866016,12.0851478552
H,-0.0001787702,-11.6894530438,11.5335254314
H,-6.200663205,-1.3612121572,15.5398459
H,-5.0070756806,-0.0740158369,15.960772261
H,5.4761222025,7.0373294208,11.5978531239
H,3.8828539604,7.7931715139,11.9837651005
H,-2.3598224467,-13.5682419448,10.8223287853
H,-0.6527374284,-13.7689814253,10.2708363944
H,-5.3884372994,-2.7485317574,17.4790101481
H,-4.1948074199,-1.461418845,17.8999595546
H,5.9945468719,8.9549096164,10.0350048566
H,4.4128258552,9.7154227427,10.4569933878
H,-1.6021030659,-13.7127505554,13.2202444844
H,0.1049512592,-13.9135126807,12.668701684
H,-6.07715669,0.1845337766,18.2044050153
H,-7.2707040295,-1.1026532937,17.783524903
H,5.1232916481,9.7160919206,12.9108242057
H,6.7347805416,9.0785582644,12.4061917197
H,-0.547571013,-15.9933181259,11.4062085223
H,-2.2545623824,-15.7925878481,11.9576745525
H,-6.4586392134,-2.4897980235,19.7230245495
H,-5.2651082928,-1.2027115757,20.1439113784
H,5.4397499676,11.7340033657,11.4745708117
H,7.0234241076,11.0974206565,10.8896815033
H,0.2097938317,-16.1362747226,13.8026288777
H,-1.4971087315,-15.9355371667,14.3541059062
H,-7.1462160113,0.4425358509,20.4466934644
H,-8.3396506106,-0.8445375106,20.0258518794
H,-5.2245509175,0.0686152129,0.087146702
H,-4.4608454959,-3.4566344977,-2.3060305122
H,-6.9565573853,0.529941416,1.4709396837
H,-6.7860477899,1.6687333534,0.042645206
H,-8.9358739717,-4.0243419314,-3.1915739802
H,-8.3216095882,-4.5997175324,-1.5683559705
H,-9.3829684849,-0.205703071,-2.0838507327
H,-8.9590326174,-1.2079268452,-3.5605426952
H,-9.4306161421,1.0176779112,1.5517875224
H,-9.2710382798,2.1304831123,0.1394501493
H,-7.6877126839,-5.9402473514,-4.3081684877
H,-7.1364289533,-6.5218151114,-2.6912366168
H,-10.9468255177,-2.697075493,-3.1104011631

H,-11.365082851,-1.7173720981,-1.6525586481
H,-7.8225532623,2.4557053115,2.8665570009
H,-7.6636609925,3.5695390855,1.4533958132
H,-10.0917508039,-6.2251713371,-3.5938559481
H,-9.5428674661,-6.8007917306,-1.9724667812
H,-11.2190689274,-0.6499432022,-4.5644453785
H,-11.6384163976,0.3291699715,-3.1058231375
H,-10.298046523,2.9117415197,2.9474443354
H,-10.1396533169,4.0249329252,1.5347933711
H,-8.8559282952,-8.1285961033,-4.6874341308
H,-8.3085258105,-8.7044337056,-3.0663108178
H,-13.1824959315,-2.1619531693,-4.1133941419
H,-13.6016070993,-1.183004495,-2.6555259982
H,-8.689220893,4.3426024685,4.2553539052
H,-8.530562593,5.4557015694,2.8428561726
H,-11.25653927,-8.4024613487,-3.9739480895
H,-10.7090604047,-8.9779687188,-2.3528156322
H,-13.4472949311,-0.1182952498,-5.5621782338
H,-13.8662834855,0.8608603585,-4.104531184
H,-11.0065589693,5.9120548242,2.9246030531
H,-11.1649542412,4.7991534897,4.337159166
H,-10.0214721675,-10.3065282123,-5.0668707273
H,-9.4743070688,-10.8820190277,-3.445711503
H,-15.4111149205,-1.6298507474,-5.1112635341
H,-15.8301012857,-0.6507928592,-3.6536365561
H,-9.5563873714,6.2300007537,5.644863996
H,-9.3978916613,7.3428949983,4.2323559917
H,-12.4219014003,-10.5802987017,-4.353781463
H,-11.8746165945,-11.1558420248,-2.7326899078
H,-15.6759686643,0.4135453118,-6.5600659556
H,-16.0948224318,1.392767073,-5.1025319318
H,-12.0321778447,6.6865204995,5.7268077101
H,-11.8738127426,7.7993155578,4.3142602673
H,-11.1869083714,-12.484375727,-5.4466623891
H,-10.6397452038,-13.0598874331,-3.8255583019
H,-17.639853327,-1.0978947617,-6.109006767
H,-18.0587261059,-0.1187479334,-4.6514627493
H,-10.423714727,8.1174842207,7.0344464477
H,-10.2653045814,9.2302746625,5.6219274468
H,-13.5872582738,-12.7582716074,-4.7336641636
H,-13.0400281792,-13.3338211775,-3.1126097306
H,-18.3235351638,1.9246718995,-6.1003881121
H,-17.9047303025,0.9454408626,-7.5578719489
H,-12.7412221136,9.6869952467,5.7041629274
H,-12.8995738471,8.5742811438,7.1166346925
H,-11.8053801473,-15.2381376896,-4.2055391855
H,-12.3525379739,-14.662634692,-5.8265386636
H,-19.8689487339,-0.5657795909,-7.1069318609
H,-20.2877576242,0.413361485,-5.649490006
H,-11.1325843698,11.1162454866,7.0105033997
H,-11.2909572513,10.003578155,8.4229046236
H,-14.2041972289,-15.5104749892,-3.49253979
H,-14.7513695975,-14.9349783463,-5.1134465399
H,-20.5509284902,2.4557846856,-7.0973581657
H,-20.1321624983,1.4766388913,-8.5547050029
H,-0.4888343607,1.604740198,-5.043584247
H,2.1585331223,-1.780876111,-5.5583152912
H,-2.0217695166,2.4522958176,-6.483547306
H,-0.6778166233,3.6095899959,-6.0137088251
H,2.7638969032,-0.841237348,-10.0177451545

H,1.2253293075,-1.7042084416,-9.5370403277
H,1.40038914,2.8310434126,-9.1686034886
H,2.9633616366,1.8710850051,-9.1514612043
H,-2.3054248657,3.684125303,-8.6670449931
H,-0.9756750404,4.8183797806,-8.2155273062
H,4.0769709937,-2.9578788728,-9.4969226682
H,2.5404174687,-3.8064679617,-9.0778961822
H,2.4749396968,1.0498265868,-11.4879137342
H,0.9328067058,1.9887066225,-11.5077123145
H,-3.6164434653,4.4120991734,-6.6337584948
H,-2.2861339928,5.5479848305,-6.1832447925
H,3.2095785573,-2.5333512226,-11.8288812374
H,1.6681066754,-3.3759201054,-11.410009223
H,3.7741590413,3.1905587992,-11.1583195463
H,2.2311919312,4.1290942041,-11.1792952614
H,-3.8984800019,5.6135472142,-8.8291765548
H,-2.5686969715,6.7489355691,-8.3792209901
H,4.4980810403,-4.6361229873,-11.3144924619
H,2.9567041634,-5.4783604552,-10.8972495935
H,3.2879696827,2.3403061251,-13.4789987784
H,1.7457982676,3.2787275132,-13.4998193805
H,-5.2019966164,6.3353866911,-6.7978511966
H,-3.8723766919,7.4707108091,-6.3478804487
H,3.6312392511,-4.2019930393,-13.6398122204
H,2.089859786,-5.0439562099,-13.222372593
H,4.5826687747,4.4749641033,-13.1431425986
H,3.0406909244,5.4135098257,-13.1638721121
H,-4.1555777415,8.672377854,-8.5435898112
H,-5.4852093344,7.5371371609,-8.9933254425
H,4.9189808147,-6.3052256057,-13.1263379049
H,3.3775056683,-7.1470459424,-12.7092789132
H,4.0966553381,3.625276633,-15.4640618638
H,2.5547132012,4.563760945,-15.4848049227
H,-6.7885611925,8.259097535,-6.9623027563
H,-5.4589776018,9.3943413533,-6.5125648865
H,4.0526220756,-5.8709594584,-15.4514965934
H,2.5112395165,-6.7128717053,-15.0343292259
H,5.3913567721,5.7597036031,-15.1283390397
H,3.8494920443,6.6982798571,-15.1489550054
H,-7.071975361,9.4607664401,-9.157842355
H,-5.7423818795,10.5959722921,-8.70821301
H,5.3402655737,-7.9742016738,-14.9381218375
H,3.7988349176,-8.8160281111,-14.5211027307
H,4.90523753,4.9101358557,-17.4492768076
H,3.3633730712,5.848665558,-17.4699222451
H,-8.3753003613,10.1828789182,-7.1269115357
H,-7.0457316583,11.3180787709,-6.6772763999
H,4.4740409903,-7.5400353084,-17.2632553194
H,2.9326784335,-8.3819249779,-16.846175002
H,4.6581754557,7.9830921634,-17.1341769116
H,6.1999890087,7.0445163097,-17.1135963402
H,-7.3294867971,12.5199392229,-8.8728367262
H,-8.6589991496,11.3847938561,-9.3224151321
H,4.2203267682,-10.4852774552,-16.3332424857
H,5.7616545371,-9.6434699973,-16.7502524379
H,5.7139740593,6.1952767242,-19.4347851703
H,4.1722122276,7.1337836731,-19.4553912912
H,-8.6313834137,13.2405070142,-6.8423644294
H,-9.9608316367,12.1054149489,-7.29193713
H,3.3541280219,-10.0501106587,-18.6564179228

H,4.895353665,-9.2083034742,-19.0734485997
H,5.4661317131,9.2666723843,-19.1183493223
H,7.0078015222,8.3281776444,-19.0977706501
N,1.8185582209,-0.6874916956,-1.5291910743
N,-1.4504106677,-1.514610297,0.8859643878
N,-1.0852704172,-0.9715633532,-1.9956114752
N,1.415506436,-1.1629110514,1.3257681198
O,7.468151928,1.5301822216,2.2750386015
O,8.0970650207,-2.2378047123,-0.6711414217
O,9.1831498288,-0.0751269337,0.9081848442
O,-0.0538484303,-0.2522392385,7.7697166515
O,-1.7891771667,-4.6090800808,6.6303445679
O,-0.9537024232,-2.6803968817,8.6099435072
O,-7.9453905421,-0.0436210037,-0.2880344985
O,-6.8743354455,-3.9217808643,-2.9466878229
O,-8.8198073555,-2.1889493026,-1.7116844059
O,-0.2926713451,2.3890789121,-7.6706765129
O,2.6622312166,-1.4058711977,-8.0201662536
O,1.1945651603,0.7477424604,-9.2502736046
C,6.0252282912,8.4076749819,-21.0197260934
H,4.9967410354,8.4490323212,-21.4533261738
H,6.5833576927,9.3173233598,-21.3484463583
H,6.5425717306,7.5080396754,-21.4326672487
C,5.0805185374,-11.3414939039,-18.7889939889
H,4.9147237774,-11.572891692,-19.8688034214
H,6.1807197802,-11.3207313984,-18.5975982862
H,4.6353369822,-12.1647698589,-18.1794527584
C,-9.7367281336,13.8007104622,-8.6115688334
H,-10.4652452262,14.429654532,-8.0452478294
H,-8.9386575672,14.4667852642,-9.0201441981
H,-10.2716679196,13.3286328615,-9.4709231369
C,18.7089673824,-5.7034108338,-10.4630417161
H,18.0050841416,-5.5854614192,-11.3221952414
H,19.739544717,-5.4563048803,-10.8149937522
H,18.69276928,-6.7732088416,-10.1424515651
C,22.0194599039,-0.8523949926,8.3359775486
H,22.4912527979,-1.1046658708,9.3161803132
H,22.4722160142,-1.5027544577,7.5488604017
H,22.2601405423,0.2106173307,8.0918837298
C,7.5943449853,14.6366960351,9.1417976858
H,8.099567863,15.0354004083,8.2289390235
H,6.9649942448,15.4487265982,9.5794809581
H,8.3815465505,14.361207406,9.8847407324
C,7.0100910358,11.727483079,12.9579837777
H,7.9115785255,11.1346836574,13.246761284
H,7.3345625849,12.768961071,12.7187572354
H,6.3207506661,11.7653178523,13.8359676545
C,-7.3458371162,-1.1209465309,21.9236416361
H,-8.1344651282,-0.5774258361,22.4978173361
H,-7.5554641388,-2.2162126404,21.9865493959
H,-6.3587939256,-0.925711801,22.4085475076
C,-1.0789107152,-17.8173362655,13.3796106781
H,-0.8316885728,-18.3399230976,14.3350192737
H,-2.1440810431,-18.0352230516,13.1237896975
H,-0.4325881337,-18.2364773257,12.5708474847
C,-13.0583507881,11.1665296202,7.9875381324
H,-12.7687865613,11.997706141,8.6747982868
H,-13.6009144375,11.6035355616,7.1145226309
H,-13.7597002176,10.4878719975,8.5307143013
C,-22.043720869,1.002509186,-7.6676751489

H,-22.6029373345,1.7227773796,-8.3122810051
H,-22.1170919858,-0.0127477794,-8.127434597
H,-22.5370064647,0.9689922063,-6.6661508402
C,-13.8029636341,-16.8756493007,-5.1176514965
H,-14.7656179191,-17.4266388112,-4.9883139496
H,-13.5491825808,-16.8594706904,-6.2052362256
H,-13.0005569817,-17.4364944594,-4.5799655074

2H16 (AM1)
–2.0623854 hartree
C,3.7457418643,-1.6279142731,1.6100149592
C,2.821421763,-0.5986498924,1.0413131851
C,3.5064754167,0.5793741578,1.0367607327
C,4.8592970109,0.2985342554,1.6001081623
C,7.169291981,0.9591821658,2.2795744726
C,8.1823264636,1.9377252874,2.5290865388
C,9.308284573,1.2636159665,3.0373809958
C,8.9846183851,-0.1280411049,3.1034171076
C,9.5312856917,-2.5011384589,3.6552290691
C,10.4469311968,-3.5764593132,4.1355155158
C,9.7422266192,-4.7425307942,4.10437055
C,8.3740780801,-4.4011301164,3.6053658282
C,6.0693698952,-5.1032951274,2.9953751094
C,4.9551168685,-6.0861535925,2.8612659378
C,3.8564750303,-5.4288256608,2.4028288375
C,4.212259185,-3.9925797881,2.2150258299
C,9.8594416002,-1.1783393906,3.5552697931
C,7.3346818801,-5.389266673,3.4401288808
C,3.3672845137,-3.0135339216,1.7610777128
C,5.8538927409,1.2227935927,1.7552212937
C,1.9918826224,-3.379072953,1.3970326621
C,1.7701753025,-4.3090749189,0.3695237861
C,0.9130874436,-2.8087311813,2.0715904769
C,0.4653966128,-4.668699804,0.0259936343
C,-0.4029167054,-3.1766211053,1.7343981199
C,-0.6411966566,-4.1082883374,0.7097797747
C,1.2300056767,-6.0389145585,-1.7728372991
C,-2.6366547388,-2.3317890887,1.8768454712
C,-2.3711111619,-5.7116630804,0.6525296643
C,0.5944436273,-6.916812621,-2.8369463047
C,-3.2753843572,-1.2076081115,2.6733118782
C,-3.8188083227,-5.8037545821,0.2063097165
C,1.6506918986,-7.4714837274,-3.767586806
C,-4.6485549259,-0.8918260343,2.1226050618
C,-4.3766495845,-7.1759289233,0.5136668143
C,1.0168405635,-8.3055641719,-4.8604536575
C,-5.3013484979,0.2309662332,2.900234456
C,-5.8215173008,-7.2866501072,0.0759369247
C,2.0528402185,-8.8412255153,-5.8258714281
C,-6.6761324642,0.5497934508,2.3529349184
C,-6.3834142056,-8.6585809853,0.3813635744
C,1.3922674637,-9.5805490829,-6.9698648725
C,-7.3292235724,1.6727781991,3.1298211858
C,-7.8280955817,-8.7699901524,-0.0564208357
C,2.4062008138,-10.0658094455,-7.9839678788
C,-8.7040769304,1.9920083022,2.5830068496
C,-8.3903859712,-10.1417835757,0.2487867099
C,1.7173841887,-10.689240014,-9.1793197211
C,-9.3571728892,3.1150187018,3.359827408
C,-9.835011383,-10.2533223547,-0.1891101323

C,2.7063699201,-11.0779797,-10.2573310005
C,-10.7320386644,3.4343729593,2.8131261209
C,-10.3973894709,-11.6250840019,0.1160626369
C,1.9914724245,-11.6323699325,-11.4712885814
C,-11.3851124481,4.5573878159,3.5899524612
C,-11.8419873886,-11.7366689548,-0.3219030066
C,2.961308824,-11.9848106582,-12.5785439039
C,-12.7599824771,4.8768020543,3.043299295
C,-12.4044057692,-13.1084148404,-0.016739696
C,2.2262564446,-12.4524046626,-13.8168036924
C,-13.4130401023,5.9998162446,3.8201349354
C,-13.8489892292,-13.2200241779,-0.4547374585
C,3.1888438219,-12.8635171228,-14.9100040373
C,-14.7879158832,6.3193028108,3.2735421421
C,-14.411475982,-14.5917449115,-0.1495951109
C,2.4507861353,-13.2534381798,-16.1728110741
C,-15.4410150535,7.4422285496,4.0502625244
C,-15.8559565059,-14.7034717189,-0.5875331244
C,3.4072258439,-13.7332373468,-17.2439690075
C,-16.8163508388,7.7628510004,3.5045684309
C,-16.4198641858,-16.0750864947,-0.2828806244
C,2.6848965745,-14.0605675605,-18.5250930868
C,-17.4704498142,8.880472878,4.2749872143
C,-17.8580437353,-16.1908974303,-0.7172709994
C,7.6673456641,-6.7846985272,3.7556741338
C,7.5847998946,-7.7508995944,2.7409860504
C,8.0404136433,-7.1550322344,5.0466494538
C,7.8727446623,-9.0874577238,3.0241175816
C,8.3486184383,-8.4986629516,5.3314061862
C,8.2654887548,-9.4785364593,4.3271284273
C,7.2774782422,-9.742932278,0.8136602648
C,8.4188897923,-9.9992947698,7.1928680438
C,9.6589225569,-11.3662530394,4.0778202982
C,7.1523652565,-11.0371030189,0.0291665516
C,8.4152064833,-9.7900593328,8.6969349258
C,9.7379935225,-12.7933074981,4.5883940797
C,6.6031001424,-10.7489173108,-1.3510698415
C,8.1219787842,-11.0942366805,9.4052731172
C,10.9718995539,-13.4781739607,4.0434613803
C,6.403636224,-12.0250108217,-2.1398435755
C,8.1090145017,-10.9064330385,10.9072942579
C,11.0682812365,-14.9040138392,4.5426573559
C,5.9010614365,-11.7284715164,-3.5368397324
C,7.8167998013,-12.2093684424,11.6201687602
C,12.3015829831,-15.5930813093,3.999185
C,5.6763363457,-13.0030324404,-4.3211446703
C,7.803346945,-12.0221558249,13.1221491011
C,12.398342961,-17.0189863114,4.4977861566
C,5.2518532478,-12.7069840011,-5.7436501848
C,7.5113749395,-13.3248254578,13.8355505885
C,13.6314538156,-17.7084693107,3.9544720067
C,5.0542863308,-13.9846612469,-6.5307753565
C,7.4978861174,-13.1376497852,15.337522909
C,13.72821184,-19.1344116419,4.4529331194
C,4.6843742971,-13.6942873671,-7.9693048438
C,7.2060102184,-14.4402565787,16.051071009
C,14.9612714889,-19.8239952129,3.9096394841
C,4.5577573893,-14.9724315302,-8.7705019469
C,7.1925308954,-14.2530605203,17.5530376347
C,15.0580041688,-21.2499560314,4.4080439372

C,4.2923400533,-14.6810715366,-10.2316032332
C,6.9007016294,-15.5556364083,18.2666589428
C,16.2910389374,-21.9395886058,3.8647593641
C,4.2262395706,-15.9561728506,-11.0447940351
C,6.8872331431,-15.3684216816,19.7686205496
C,16.3877578835,-23.3655569837,4.3631367012
C,4.0816405498,-15.6557978232,-12.5210883605
C,6.5954242089,-16.6709519343,20.4823303916
C,17.6207680605,-24.055259329,3.8198893921
C,4.0097205372,-16.9265639047,-13.3397755621
C,6.5819437604,-16.4838528631,21.9841985773
C,17.7175744457,-25.4811463916,4.3181579614
C,3.9451207023,-16.6216783643,-14.8213515821
C,6.290162527,-17.7859717834,22.6993766534
C,18.9504943662,-26.1722274672,3.7755273793
C,3.8429775675,-17.8805471564,-15.6426862631
C,6.2757601275,-17.604408388,24.1951362903
C,19.0509845754,-27.5923574264,4.2690865601
C,11.2213070073,-0.7538080024,3.9303161402
C,12.3158328188,-1.2184047022,3.1868155826
C,11.4150164367,0.0916743956,5.021024655
C,13.6091057043,-0.8227075101,3.5360415059
C,12.7172164455,0.4921274467,5.3745427277
C,13.8255016813,0.0425886855,4.636686213
C,14.5591812527,-2.1496048894,1.7954231032
C,13.9308806071,1.2670974579,7.2836788089
C,15.7967114373,1.2535242397,4.1678977706
C,15.9510046682,-2.4589356731,1.2718282988
C,13.5010800969,1.82613737,8.628504728
C,17.1318778531,1.5441566443,4.8284714488
C,15.8715490959,-3.4489163148,0.1306496258
C,14.6677376863,1.8254132337,9.5916010054
C,17.9499901855,2.4815966466,3.9679807281
C,17.2503096111,-3.77135449,-0.4048599385
C,14.2589071718,2.3752419537,10.9414377857
C,19.2865970899,2.7837052588,4.6112001529
C,17.1762850199,-4.7618725965,-1.5470735478
C,15.4236647393,2.377114711,11.9081643637
C,20.1086291575,3.7212705118,3.7530346874
C,18.5545244831,-5.0848339423,-2.0831372583
C,15.0154499472,2.9265554793,13.2582205683
C,21.4454282474,4.0234710389,4.3955431371
C,18.4812058805,-6.0752483157,-3.2254431511
C,16.1798596695,2.9288034594,14.2253209442
C,22.2678233387,4.960983691,3.5377103984
C,19.8593544024,-6.3983586154,-3.7615918872
C,15.7717043704,3.4782422881,15.5753815864
C,23.6046968219,5.2631017733,4.1800748781
C,19.7862128037,-7.388713248,-4.9039512718
C,16.9360291415,3.4806055994,16.542578115
C,24.4271715098,6.2006132945,3.3223234611
C,21.1643445622,-7.7118865749,-5.440090155
C,16.5278675655,4.0300689992,17.8926231052
C,25.7640829948,6.5026649157,3.96463274
C,21.091284674,-8.7022131548,-6.5824756092
C,17.6921519164,4.0324853262,18.8598664125
C,26.5865922757,7.4401822229,3.1069231104
C,22.4694139707,-9.0254183791,-7.1185919026
C,17.2839807685,4.5819670439,20.2098979298
C,27.9235234805,7.7421951728,3.7492029807

C,22.3964539974,-10.0157316501,-8.2609924579
C,18.4482085085,4.5844258644,21.1772069755
C,28.7460879334,8.6797191109,2.8915564304
C,23.7744410796,-10.3390051529,-8.7971308414
C,18.0401689198,5.1338651714,22.5271756775
C,30.0829636716,8.9817625587,3.5336855456
C,23.7029466463,-11.3294957651,-9.9399130866
C,19.2037759115,5.1368953701,23.4957513041
C,30.9067904476,9.9194630067,2.676854486
C,25.0728328479,-11.6543340917,-10.4769052013
C,18.8020601768,5.6832159382,24.8413470063
C,32.2388753913,10.2230017859,3.3123716753
C,5.5984915037,2.6202447446,1.3611552241
C,6.3796362023,3.2076536049,0.35520955
C,4.5874151869,3.3483281752,1.9859436282
C,6.1349534643,4.5271678004,-0.0312461246
C,4.3380366007,4.6779096609,1.5972850849
C,5.1047919786,5.2796849719,0.5853094458
C,7.9465251934,4.4820190163,-1.5831015514
C,3.3921821328,6.6862128069,2.4851347235
C,4.3566488677,6.8411272685,-1.019173828
C,8.5871032958,5.4463653736,-2.5662861797
C,2.5049626968,6.9716172978,3.6839476068
C,4.1512626546,8.3407830058,-1.1303677871
C,9.7827189661,4.798507661,-3.229214782
C,2.4982077733,8.4535622771,3.9879916463
C,3.5622745747,8.6910977364,-2.4790403617
C,10.4369983853,5.7453314509,-4.2126508551
C,1.6223054184,8.7593364042,5.1841537548
C,3.3497432161,10.1842543024,-2.6087705665
C,11.6342842219,5.1017448399,-4.878655068
C,1.6119505881,10.2414712509,5.4913891528
C,2.7607400656,10.539110601,-3.9572385285
C,12.2892599922,6.0478654486,-5.862052136
C,0.7365045928,10.5478836972,6.6875832777
C,2.5482304037,12.032093442,-4.0876785132
C,13.4866290074,5.4048748408,-6.5284066742
C,0.725619091,12.0299181879,6.9951437578
C,1.9592505531,12.3874136277,-5.436007358
C,14.1417948734,6.350889125,-7.5117486048
C,-0.1498012316,12.3363941095,8.1913246235
C,1.746831345,13.8803858203,-5.5665804066
C,15.339179426,5.7080441092,-8.1781991853
C,-0.160847515,13.8184106896,8.4989473292
C,1.1578378399,14.2358065041,-6.9148728058
C,15.9944072975,6.6540545521,-9.1614959916
C,-1.0362781023,14.1248815532,9.6951178688
C,0.9454806396,15.7287805611,-7.0454904292
C,17.1918044623,6.0112799136,-9.8279861146
C,-1.0473960424,15.6068901785,10.0027707769
C,0.3564735089,16.0842465027,-8.3937634926
C,17.8470523071,6.9572992561,-10.8112560131
C,-1.9228348732,15.9133523383,11.1989340562
C,0.1441539184,17.5772211068,-8.5244045553
C,19.0444665205,6.3146112365,-11.4777944065
C,-1.9340137351,17.395345552,11.5066518584
C,-0.4448686829,17.9327619857,-9.872649266
C,19.6997551329,7.2604913264,-12.4610063981
C,-2.8093708991,17.7018958202,12.7027170816
C,-0.6571981798,19.4256169867,-10.0034029944

C,20.8976663686,6.6189065704,-13.1284728138
C,-2.8214565434,19.1839701976,13.0116485564
C,-1.246363882,19.7826596127,-11.3515632345
C,21.5533328462,7.557196568,-14.1083616678
C,-3.6918629694,19.4938663606,14.2019535965
C,-1.4595002539,21.2682271,-11.4862116006
H,1.8082477267,-0.8147527286,0.712307737
H,3.1895484361,1.5650872172,0.7033530917
H,8.0746636936,3.0054309963,2.3556496164
H,10.2581047338,1.6990586276,3.3380148689
H,11.4736331735,-3.3930977074,4.4438014282
H,10.0519124048,-5.748334686,4.3766909424
H,5.0768083862,-7.1375777826,3.1189040203
H,2.8574055043,-5.8095964479,2.1942162368
H,7.1923196915,-1.1455622311,2.579138398
H,6.0395442671,-3.0010792587,2.669520176
H,2.6330462713,-4.7370139102,-0.1594703289
H,1.0738708792,-2.0779541582,2.8781958571
H,1.7430319008,-5.1529398656,-2.2297188699
H,1.9605676281,-6.620476288,-1.1514544573
H,-3.2575587238,-3.2663725658,1.9130346047
H,-2.472665414,-2.0389182446,0.8082695689
H,-1.7279543699,-6.4571665384,0.1160078011
H,-2.276529038,-5.8563347614,1.7600167338
H,-0.1536402209,-6.3133623673,-3.4147993032
H,0.0347790787,-7.7536371901,-2.3433846727
H,-3.3492856201,-1.5095159686,3.7506080213
H,-2.6209892295,-0.2985427759,2.6283511116
H,-4.420356807,-5.0146440441,0.7289764817
H,-3.88245466,-5.5959461249,-0.8935750644
H,2.2312358888,-6.6279110352,-4.2264207539
H,2.3778796832,-8.0982714931,-3.1874286024
H,-5.2946493406,-1.807984105,2.1705307825
H,-4.564480341,-0.6020601997,1.0418197162
H,-4.3007868879,-7.3768636852,1.6150208056
H,-3.7643031698,-7.9576725149,-0.0088098827
H,0.2713932939,-7.6804102893,-5.4203759681
H,0.4527557403,-9.1602905874,-4.4022868247
H,-5.3840668705,-0.0586600429,3.9810240877
H,-4.6551844629,1.1469436723,2.8520964676
H,-6.4336089107,-6.5045022191,0.5978703459
H,-5.897243788,-7.0852206424,-1.0252108211
H,2.6650469652,-7.9923083893,-6.2303320769
H,2.7559122724,-9.5287545317,-5.2862345896
H,-7.322154149,-0.3662599867,2.4012033389
H,-6.5930707868,0.8388837296,1.2720207327
H,-6.3075371406,-8.8596884899,1.4825681586
H,-5.7709817492,-9.4406759033,-0.140253065
H,0.8135478561,-10.4541582351,-6.5694112007
H,0.6554268523,-8.9007870787,-7.4751828422
H,-7.412095571,1.3837546781,4.2107617966
H,-6.6831712927,2.5888170426,3.0815047325
H,-8.4405326606,-7.9879068341,0.4652173204
H,-7.9040320172,-8.5687876507,-1.1575941584
H,3.0416610932,-9.2052597738,-8.322406126
H,3.091224117,-10.8167403219,-7.5072899183
H,-9.3501178607,1.0759733194,2.6313049005
H,-8.6211510065,2.2810254626,1.5020713838
H,-8.3145971458,-10.3428656228,1.3499943421
H,-7.7778716075,-10.9239209453,-0.2726618075

H,0.9728479446,-9.9632633701,-9.6020941331
H,1.1440565774,-11.5959078764,-8.8506654518
H,-9.440040084,2.8260253605,4.4407732217
H,-8.7111078522,4.0310417151,3.311522075
H,-10.4475276953,-9.4712096374,0.3323840112
H,-9.9108509214,-10.0521864809,-1.2902990328
H,3.3126417845,-10.1818404126,-10.55388649
H,3.4223741793,-11.8454966529,-9.8568108289
H,-11.3780969247,2.5183499391,2.8614205732
H,-10.6491489489,3.7233820143,1.7321877662
H,-9.7848330402,-12.4072336898,-0.4053174645
H,-10.3216535271,-11.8261476952,1.2172748146
H,1.4110049241,-12.5470093849,-11.1777784558
H,1.251610177,-10.8771890319,-11.8476518773
H,-10.7390369092,5.4734017371,3.541662329
H,-11.4679755854,4.2683849395,4.6708951022
H,-12.4545425399,-10.954537175,0.1995137587
H,-11.9177604874,-11.5355691087,-1.4231023178
H,3.6571971292,-12.7937327494,-12.2253480933
H,3.5908028571,-11.0910191354,-12.8295818693
H,-12.6771073697,5.1658120475,1.9623607776
H,-13.4060516911,3.9607861716,3.0915861909
H,-11.7918283196,-13.890570418,-0.5380864039
H,-12.3286929769,-13.3094694343,1.0844744758
H,1.566049778,-13.3215229747,-13.5546761189
H,1.5596212558,-11.631029074,-14.1904977507
H,-13.4959013648,5.7108091965,4.9010756265
H,-12.7669583034,6.9158250072,3.7718617482
H,-14.4615660257,-12.4378797618,0.0666319966
H,-13.924732994,-13.0189428446,-1.5559414536
H,3.8091068356,-13.7314368643,-14.5551442026
H,3.8934481285,-12.0192710051,-15.1310046046
H,-15.4339615447,5.4032600353,3.3217880351
H,-14.7050234051,6.6082716965,2.192587434
H,-13.7988430469,-15.3738924703,-0.6709029399
H,-14.3357155462,-14.7927895013,0.951623522
H,1.7127320223,-14.0664833363,-15.9405604642
H,1.8667810205,-12.374963108,-16.554360613
H,-14.7952387103,8.3584256149,4.0020757696
H,-15.5241559428,7.1534630383,5.131219646
H,-16.4688330179,-13.9215381339,-0.0662555896
H,-15.9320049208,-14.5026019242,-1.6887287647
H,3.950456826,-14.6442208503,-16.8729788518
H,4.1795721721,-12.9428534005,-17.4379527208
H,-17.4602279544,6.8450116072,3.552162794
H,-16.7313446379,8.049920714,2.4230708205
H,-15.8047339422,-16.8558285218,-0.8037719313
H,-16.3415601315,-16.274761211,0.8186063881
H,3.4070953815,-14.4202830825,-19.2971478448
H,1.9230024068,-14.8594176873,-18.3552215223
H,2.1618162826,-13.158284163,-18.9248650865
H,-18.482178844,9.100478581,3.8566298877
H,-17.5862932141,8.6029585732,5.3506598464
H,-16.8554376804,9.8111070146,4.2185476157
H,-18.2536345779,-17.2087096996,-0.4836259258
H,-18.4912525083,-15.4368095644,-0.190105058
H,-17.9530044615,-16.0194308209,-1.8168548555
H,7.2971396591,-7.4340147703,1.7287487831
H,8.1064181299,-6.4088582743,5.8524305166
H,6.2735047265,-9.2630779937,0.9549674083

H,7.9712696577,-9.0249853029,0.302021246
H,9.1907832892,-10.7535924005,6.8838383696
H,7.4145255455,-10.3249960944,6.8192404884
H,9.5862464682,-11.3335223714,2.9593623633
H,10.539753682,-10.7626205964,4.4185180126
H,6.4769339903,-11.7429749548,0.578948849
H,8.15681401,-11.5288576528,-0.0459006551
H,9.4110752721,-9.3885419521,9.0197307676
H,7.6451667147,-9.0210140835,8.9657471376
H,9.7613415641,-12.7867636199,5.7096235562
H,8.815727071,-13.3504232332,4.2785253282
H,5.6242015914,-10.2072021033,-1.2626756037
H,7.3077715839,-10.0718818828,-1.9028657305
H,8.8967172061,-11.8571626356,9.1281352873
H,7.1290092058,-11.4926644552,9.0670528319
H,11.887976347,-12.9086437295,4.3525617878
H,10.941484786,-13.4734693498,2.9217966039
H,5.6706974564,-12.6866289971,-1.6072473593
H,7.3732483973,-12.5863225226,-2.1997143517
H,9.1015429821,-10.5067529809,11.2448952356
H,7.334129973,-10.1437231472,11.1842096683
H,11.0979868284,-14.9086462351,5.664228372
H,10.1519963538,-15.4731830669,4.2339431208
H,4.9420578813,-11.1491059838,-3.4784463361
H,6.6451642456,-11.0823826421,-4.0735629096
H,8.5917438216,-12.9719540145,11.3431245419
H,6.8244064767,-12.6089461272,11.2820249606
H,13.2176757137,-15.0236661421,4.3080378565
H,12.2719637602,-15.5880265219,2.877610683
H,6.6207140701,-13.6092172535,-4.3306835102
H,4.8908756793,-13.6235477486,-3.8146335669
H,8.7956335792,-11.6222979261,13.460254506
H,7.0282918198,-11.259661494,13.3991647587
H,12.4280254899,-17.0240364423,5.6193622271
H,11.4822035573,-17.5883940374,4.1890851976
H,4.2993083936,-12.1134482948,-5.7408507957
H,6.0322604455,-12.0745486986,-6.2439890485
H,8.2863873144,-14.0873393241,13.5585134997
H,6.5190644311,-13.7246191893,13.4974454019
H,14.5475802593,-17.1391202205,4.263321289
H,13.6019016822,-17.7033122432,2.8329006319
H,4.2492666476,-14.60215455,-6.0521968736
H,5.9976948704,-14.5918402103,-6.501162982
H,8.4901413866,-12.737719928,15.6756243162
H,6.7228093061,-12.3751856493,15.614539724
H,13.7578389581,-19.1395583065,5.5745070789
H,12.8120617388,-19.7037690091,4.1441829039
H,3.7160010948,-13.1271898395,-8.0033680895
H,5.4663323159,-13.03640123,-8.433333594
H,7.9810560016,-15.2027375762,15.774037679
H,6.213728922,-14.8401407039,15.7129932875
H,14.9317268501,-19.8187934784,2.7880685219
H,15.8774200664,-19.2546851769,4.2184875006
H,5.5025096697,-15.5705236741,-8.6736146428
H,3.7262885163,-15.5988900633,-8.3530114513
H,6.4174489551,-13.4906046295,17.8300546419
H,8.1847806076,-13.853098794,17.8911144913
H,15.0876014739,-21.25514805,5.5296170515
H,14.1418416288,-21.8192746913,4.0992621499
H,5.1043984506,-14.0196451525,-10.6357458828

H,3.327537443,-14.1154734151,-10.3359909608
H,5.908434021,-15.9555665662,17.9285978903
H,7.6757624912,-16.3181030822,17.9896280437
H,16.2614937313,-21.9343654613,2.7431885364
H,17.2072003748,-21.370297921,4.1736010851
H,5.1565829319,-16.5601934121,-10.8749046514
H,3.3601843747,-16.5793358261,-10.6987198204
H,7.8794806911,-14.9684459655,20.1066841494
H,6.1121581066,-14.6059622246,20.0456409395
H,16.4173317167,-23.3707760917,5.4847089839
H,15.4715900064,-23.9348578951,4.0543408933
H,4.9524871795,-15.0359760681,-12.8643585257
H,3.1563230598,-15.04250336,-12.6935868934
H,7.3704772284,-17.4334172407,20.2052468401
H,5.6031725464,-17.0709045525,20.1442284072
H,17.5912239874,-24.0499718987,2.6983122589
H,18.5369215818,-23.4859300667,4.1287050844
H,4.9083793937,-17.5638882498,-13.1274533234
H,3.106153637,-17.5185542881,-13.0371510143
H,5.8068798857,-15.7215434037,22.2615575785
H,7.5741113696,-16.0840240811,22.3225853041
H,17.7472906322,-25.4867120757,5.4396988524
H,16.8016169777,-26.0507462997,4.0093822775
H,4.8607277576,-16.0467053906,-15.1242728325
H,3.0618045145,-15.9596097626,-15.0309053333
H,7.0654146597,-18.5475207525,22.4195583051
H,5.2982666991,-18.1850351073,22.3585478607
H,18.9197582986,-26.1643043504,2.6538038139
H,19.8653644165,-25.6003148322,4.0840776861
H,3.8018874254,-17.6306998283,-16.7304425051
H,4.7262920416,-18.5405858821,-15.4650388708
H,2.9207913404,-18.4516877519,-15.3765906828
H,6.0590629503,-18.5754369011,24.7022525275
H,7.2642372771,-17.230589308,24.5566287967
H,5.4923445591,-16.8671275034,24.4954415723
H,19.9659457798,-28.0827870415,3.8575664545
H,19.1076403303,-27.6192957988,5.3842412749
H,18.159490374,-28.1848175441,3.9501314442
H,12.1374867309,-1.889981591,2.3351889531
H,10.5627696739,0.4611455275,5.6109277926
H,14.064314477,-3.0716059678,2.1984415695
H,13.9177649256,-1.6934467271,0.9962977835
H,14.7641133075,1.8701411639,6.8338756846
H,14.2519468473,0.197203344,7.3650909171
H,15.9308026869,0.7535280635,3.173265863
H,15.1914788216,2.1887307659,4.0441221735
H,16.5777238444,-2.8736869547,2.1038962297
H,16.4395859241,-1.5096269489,0.929691791
H,13.1116242366,2.8686013139,8.4914707724
H,12.659469273,1.2093465788,9.0383098564
H,16.9564356572,1.9986503365,5.8386233894
H,17.6844958407,0.5818622734,4.9885030022
H,15.3746828456,-4.3917277781,0.4819450684
H,15.2373565577,-3.0267887143,-0.6932073402
H,15.5048559518,2.4433201177,9.1714815067
H,15.0564879711,0.7798159271,9.7129955599
H,17.3849427475,3.437706173,3.8077220778
H,18.1140313346,2.0202824572,2.958348166
H,17.884506295,-4.1931792975,0.4189948624
H,17.7476166267,-2.8286967648,-0.7554491859

H,13.8689632445,3.4201798624,10.819461323
H,13.4220170142,1.7571100984,11.36142183
H,19.1224959598,3.244249503,5.6210495425
H,19.851237168,1.8275471567,4.7717739915
H,16.678823353,-5.7043737196,-1.1962335274
H,16.5417060272,-4.3400731515,-2.3706270938
H,16.2605048299,2.9951533218,11.4879809595
H,15.8135165442,1.3320719565,12.0296272295
H,19.5437249497,4.6773076538,3.5926397023
H,20.2722908561,3.2608101966,2.743072437
H,19.0521640983,-4.1423731565,-2.4337919684
H,19.1890692628,-5.5067501056,-1.259612737
H,14.6253320063,3.9714859644,13.1367229455
H,14.1786826047,2.308409521,13.6784140655
H,21.2817763566,4.4839264187,5.4055126172
H,22.0103193926,3.0674505786,4.5560343679
H,17.9835933809,-7.0177202778,-2.874779588
H,17.8466403474,-5.6534004709,-4.0489715585
H,17.0166673232,3.5468589556,13.8051056627
H,16.5698786063,1.8838389082,14.3468369228
H,21.7029750624,5.9170568831,3.3773841361
H,22.4313530976,4.5006559345,2.5276712834
H,20.4939012619,-6.8202893355,-2.9380863332
H,20.3570599318,-5.4559061813,-4.1121643538
H,15.3815596702,4.5231567472,15.4538609157
H,14.9349347486,2.8601255364,15.9955982999
H,23.4411642822,5.7234591851,5.1901047965
H,24.16954903,4.3070454346,4.3404634488
H,19.2885205793,-8.3311766645,-4.5533892939
H,19.1516912654,-6.9668122729,-5.7274841097
H,17.7728244645,4.098667584,16.122347803
H,17.3261079912,2.4356701139,16.6641302618
H,24.5906352771,5.7403374185,2.3122505667
H,23.8623569166,7.1567118829,3.1620450816
H,21.6620890206,-6.7694366777,-5.7906107206
H,21.7988613018,-8.1338306432,-4.6165719288
H,15.6910898717,3.4119728627,18.3128483438
H,16.137720051,5.07497747,17.7710675363
H,25.6006131903,6.9629635858,4.9746979893
H,26.3289026146,5.5465789762,4.1249527885
H,20.4567934559,-8.2802840798,-7.4060166935
H,20.5935458793,-9.6446698675,-6.2319660904
H,18.0822578482,2.9875634234,18.9814401427
H,18.528944265,4.6505473437,18.439629766
H,26.7500199248,6.9799354736,2.0968311753
H,26.0217948084,8.3962942871,2.9466723247
H,22.967187548,-8.0829735616,-7.4690816868
H,23.1039097683,-9.4473660202,-6.2950618154
H,16.8938348006,5.6268730512,20.0883240109
H,16.4471915986,3.9638917655,20.6301269261
H,27.7600930562,8.2024532584,4.7592916217
H,28.4883271432,6.7860944234,3.9094848938
H,21.7619364749,-9.5937838812,-9.0845123588
H,21.8986410698,-10.9581605697,-7.9104945118
H,19.2850113991,5.2024547073,20.7569246705
H,18.8383358262,3.539500803,21.2987416468
H,28.9094460497,8.2194861233,1.8814396806
H,28.1812533144,9.635814849,2.7312975585
H,24.2724406413,-9.3967844203,-9.1478197269
H,24.4091233427,-10.7611105131,-7.9738609597

H,17.20347649,4.5159319029,22.9477366928
H,17.6501199056,6.1788202908,22.4059494413
H,29.9198931888,9.4421401189,4.5437490853
H,30.6480679835,8.0258657937,3.6939452293
H,23.0665879537,-10.9062623257,-10.7616146694
H,23.2032578008,-12.2705234669,-9.5877041351
H,20.0400912269,5.7540783674,23.0727465592
H,19.59344679,4.0912740216,23.6145182712
H,31.0675084519,9.4580399804,1.6666417298
H,30.3393844359,10.8742365373,2.5164415036
H,24.9971624378,-12.3853663727,-11.3176732084
H,25.5769407293,-10.7323444397,-10.855473798
H,25.713966678,-12.1002771469,-9.6784148075
H,19.6737307461,5.6764824623,25.5393195638
H,18.4340649166,6.7335184081,24.7467474407
H,17.9862191854,5.0662453842,25.2899637749
H,32.8294425532,10.9151461706,2.6649227209
H,32.0991713361,10.7060707439,4.309684479
H,32.8292652913,9.2860665404,3.4576149392
H,7.1785276658,2.6162603849,-0.1144645106
H,3.9721869756,2.898562888,2.7795887307
H,8.6609097975,4.2041403408,-0.764678235
H,7.584799949,3.555545847,-2.1016922541
H,3.0399378884,7.2353201026,1.5715262444
H,4.460047546,6.9556652024,2.6886968093
H,5.0716106828,6.4656754899,-1.7970808851
H,3.3841174846,6.2898573555,-1.0998326728
H,8.8993718731,6.3770345186,-2.0245680867
H,7.831497485,5.7487087153,-3.3373854025
H,1.4637663477,6.6145746924,3.4704349612
H,2.8774254935,6.3955263608,4.5705565466
H,3.4704302889,8.6853355456,-0.3084826175
H,5.1337665546,8.8618626745,-0.9883457393
H,10.5284460527,4.492996277,-2.4483573751
H,9.4601977925,3.8656536599,-3.7629725144
H,2.1260990317,9.0209091459,3.094277046
H,3.5455463176,8.8024483022,4.1892467084
H,2.5838053619,8.158684117,-2.614091696
H,4.2477710506,8.3364605986,-3.2935414525
H,10.7591383773,6.6782348418,-3.6789909962
H,9.6913103454,6.0514740616,-4.9930717068
H,0.5755466651,8.4090183355,4.9831654808
H,1.9947830762,8.1921879794,6.0777220864
H,2.6649694509,10.5389119168,-1.7938313784
H,4.328147042,10.7164363551,-2.4733092879
H,12.3796566997,4.7954395017,-4.0979757363
H,11.3119891556,4.1687285931,-5.4119965213
H,1.2396094482,10.8084856832,4.5976988167
H,2.6589088852,10.5914532806,5.6920026187
H,1.7824132763,10.0067131425,-4.0924376639
H,3.4454295572,10.1840641641,-4.7720865719
H,11.5439136334,6.3543459409,-6.6426706913
H,12.6115855823,6.9808695832,-5.328703475
H,-0.3103682899,10.1975926136,6.4871263762
H,1.1090152037,9.980981662,7.5812868908
H,1.8635665489,12.3871548966,-3.2728139104
H,3.5265323079,12.5645094849,-3.9524247859
H,14.2319581438,5.0983624343,-5.7477857554
H,13.1643403244,4.4718817423,-7.0617754979
H,0.3532186184,12.5968469287,6.1014272752

H,1.7725098444,12.3801218445,7.1956704756
H,0.9808905015,11.8550836922,-5.5711724821
H,2.6438093838,12.0322393257,-6.2509027262
H,14.4641119546,7.2838763405,-6.9783799414
H,13.3964826855,6.657481591,-8.2923508384
H,-1.1966559591,11.9860465094,7.990885849
H,0.2226970889,11.7695237716,9.085046041
H,1.0622601742,14.2355573002,-4.751689941
H,2.7251717878,14.4127382323,-5.4313861989
H,16.0844961029,5.4014365217,-7.397608386
H,15.0168878338,4.775077293,-8.7116085211
H,-0.5332780432,14.3852989081,7.6052175078
H,0.8860139793,14.1687083952,8.6994482601
H,1.8423439656,13.8805723216,-7.7297853723
H,0.1794530114,13.7035201191,-7.0500129
H,15.2491055626,6.9606992043,-9.9420860422
H,16.3167101163,7.5870238531,-8.6280926484
H,-0.6637914282,13.558019138,10.5888462996
H,-2.0831203561,13.7745061399,9.4946653333
H,0.2609624539,16.084008732,-6.2305819164
H,1.9238513111,16.2610839494,-6.9103302898
H,16.8695191372,5.0783236059,-10.3614163698
H,17.9371137223,5.7046308477,-9.0474060672
H,-0.0005485648,15.9572322518,10.2032613879
H,-1.4198389919,16.1737618304,9.1090352945
H,1.0409499632,15.7289781066,-9.2086858038
H,-0.6219255937,15.5519845436,-8.5288880748
H,17.1017564438,7.2639733675,-11.5918385702
H,18.1693418066,7.8902612652,-10.2778353936
H,-2.9696721355,15.5629687721,10.9984732239
H,-1.5503663066,15.3464854899,12.092665177
H,-0.5403252854,17.9324880583,-7.7094818739
H,1.1225411159,18.1094980435,-8.3892672228
H,18.722168578,5.381633067,-12.0111921181
H,19.7897390766,6.0079093742,-10.6971912454
H,-2.3064196045,17.9621996357,10.6128812971
H,-0.8871508756,17.745689401,11.7070927673
H,0.2395867897,17.5774226874,-10.6875672215
H,-1.4232561561,17.4004614526,-10.0077558613
H,18.9547549331,7.5672545303,-13.2417991183
H,20.0222818495,8.1934920741,-11.9278518384
H,-2.4370915046,17.1352764995,13.5966512541
H,-3.8563135619,17.3517684539,12.502501562
H,-1.341729268,19.7811918869,-9.1886938579
H,0.3210442887,19.9581589918,-9.8684749571
H,20.5734286709,5.6852150201,-13.6598505281
H,21.6409030625,6.3114247953,-12.3459593719
H,-3.1925651814,19.7490672813,12.11598426
H,-1.7734242858,19.5325655656,13.2101008389
H,-0.5611687501,19.4249018728,-12.1650671908
H,-2.2238594139,19.2479709311,-11.4852963762
H,22.4331327489,7.0647914703,-14.5884802448
H,20.8350517609,7.855359446,-14.9100319147
H,21.9053997854,8.4832379659,-13.592608748
H,-3.6865342025,20.5906078116,14.4120741308
H,-4.7458452485,19.176001054,14.0133075058
H,-3.322883601,18.9589127203,15.1103506873
H,-1.8940151907,21.5092458903,-12.4862765812
H,-2.1594323438,21.6392493052,-10.6988469709
H,-0.4922732,21.8166688684,-11.3804415941

N,4.9280069849,-1.0871068457,1.9275617491
N,8.2723462512,-3.0921032204,3.3446772385
N,7.6814310668,-0.2839409809,2.6387106751
N,5.562910898,-3.8645976742,2.5749720675
O,0.1463935474,-5.5991870289,-0.9410713881
O,-1.3813228805,-2.6085579961,2.5216800508
O,-1.9368458193,-4.3816383584,0.307794724
O,7.8288737871,-10.1016319804,2.0889156416
O,8.7837565795,-8.7322516632,6.6183658288
O,8.4549921053,-10.8122047177,4.6435895149
O,14.7470583514,-1.1997664009,2.8548257188
O,12.7852489791,1.3857664374,6.4216378693
O,15.1062074907,0.3539653504,5.0572352941
O,6.8243800707,5.1843249306,-1.0282095742
O,3.2678026298,5.2763244652,2.22718794
O,4.923194956,6.6183527212,0.2871124414

2H20 (AM1)
–2.5879587 hartree
C,1.9108894459,2.8439954499,0.5733683749
C,3.1592041903,3.5251348381,0.1094182412
C,4.058741479,2.5404608484,-0.1702296081
C,3.3842709979,1.2405570585,0.1156270431
C,3.3042873263,-1.253321613,0.2075629184
C,3.9208027793,-2.5415598062,0.127642769
C,2.944242315,-3.500944343,0.4539939378
C,1.7295385029,-2.8010030306,0.7375873739
C,-0.6755715885,-2.7265211731,1.4040006843
C,-1.965842213,-3.363710563,1.7972348737
C,-2.8530253076,-2.3555911199,2.0294432076
C,-2.1184568919,-1.075935223,1.7842924562
C,-2.0819054894,1.4089015449,1.7487261705
C,-2.6699339511,2.7639408927,1.9568327809
C,-1.7108701937,3.6919438801,1.6952747641
C,-0.4628266192,2.9791407906,1.296250901
C,0.4739626428,-3.402201193,1.1046010794
C,-2.7372437709,0.2193761923,1.9362710728
C,0.7222387576,3.5738615106,0.9492334447
C,3.9513225843,0.0080054197,-0.047764272
C,0.8090245929,5.0401913277,0.9537166338
C,-0.0303783506,5.7911338917,0.1163446694
C,1.7211089253,5.6819285212,1.7909015125
C,0.0412094717,7.1856351359,0.1296147796
C,1.7915165096,7.0872966359,1.8109151779
C,0.9532444589,7.8534868459,0.9835376554
C,-1.6515729496,7.3661192468,-1.5466205951
C,3.3231551695,8.8388112417,2.3670532239
C,-0.0031160465,9.9462732494,1.5067563498
C,-2.3924722461,8.4916637143,-2.2476648403
C,4.6215736231,8.87431536,3.1537977823
C,0.3703372666,11.4153048192,1.4294171629
C,-3.4062190781,7.9373320818,-3.2245203672
C,5.3498318226,10.1761186967,2.901728801
C,-0.7365403509,12.2695583243,2.0069776023
C,-4.1952396362,9.0586483344,-3.8663830647
C,6.6508860695,10.2297276863,3.6736692853
C,-0.3814039936,13.7395166534,1.9390546574
C,-5.1938442367,8.5351619229,-4.8767403727
C,7.3827083703,11.5311677818,3.4248566466
C,-1.4867632313,14.5979206802,2.5157088312

C,-6.0038036942,9.6695845124,-5.4671202949
C,8.6840156768,11.5851704247,4.196126999
C,-1.132329835,16.0679305091,2.4477342274
C,-6.9703827941,9.181728692,-6.5250564404
C,9.4162888988,12.8864013259,3.9477130667
C,-2.2374325577,16.9267147416,3.0242556976
C,-7.7703812746,10.3349667004,-7.0926750743
C,10.7176113325,12.9404172884,4.7189325857
C,-1.8831467023,18.396742317,2.9561490233
C,-8.7298617915,9.8778331378,-8.1702074311
C,11.4500357452,14.2415705633,4.4705774107
C,-2.9881939531,19.2556072331,3.5326485904
C,-9.4888182796,11.0521706407,-8.7510974408
C,12.7513491659,14.2955619666,5.2418079006
C,-2.633966656,20.7256420601,3.4644554256
C,-10.3994029177,10.6244396685,-9.881592593
C,13.4838456352,15.5966759752,4.9934634042
C,-3.7389951359,21.5845368644,4.0409437877
C,-11.1446941327,11.8089891544,-10.4590861233
C,14.7851504733,15.6506430414,5.7647083098
C,-3.3847956812,23.0545750739,3.9727002021
C,-12.086556775,11.3843806512,-11.5650111179
C,15.5176874845,16.9517348315,5.5163677612
C,-4.4898155675,23.9134856437,4.5491803045
C,-12.7350654646,12.582101386,-12.2256218984
C,16.8189853067,17.0056854192,6.2876247027
C,-4.1356313021,25.3835256242,4.4809093119
C,-13.6165414842,12.1603657546,-13.3814702209
C,17.551552429,18.3067611903,6.0392899706
C,-5.2406421349,26.2424505479,5.0573843487
C,-14.2870899532,13.3536220999,-14.0276983346
C,18.852844306,18.3607064964,6.8105532343
C,-4.8864710792,27.7124910415,4.9891037021
C,-4.1606622885,0.251492332,2.2984638903
C,-5.0794675975,0.8121045443,1.3978553348
C,-4.5969359885,-0.2518123843,3.522754379
C,-6.4360938018,0.861630982,1.7246834547
C,-5.96496679,-0.2112939561,3.8519179646
C,-6.8978324957,0.3448017625,2.959691707
C,-6.9439182994,1.9560569053,-0.3285055219
C,-7.3793276325,-0.2175072696,5.7809661693
C,-9.1172251324,-0.3748724073,2.6065318668
C,-8.1723121809,2.5030739118,-1.0343553631
C,-7.1452833525,-0.5703383472,7.2393033897
C,-10.4923967172,-0.1474561979,3.2070775168
C,-7.7659108139,3.1381718263,-2.3461916827
C,-8.2890102849,-0.0631483762,8.0897450507
C,-11.5218667888,-1.0007275501,2.4995252246
C,-8.9674686558,3.666394721,-3.0997626486
C,-8.0727195873,-0.4007165483,9.5494612688
C,-12.9011977839,-0.787572453,3.0856307598
C,-8.550417534,4.2777324439,-4.4204581011
C,-9.2154441554,0.1043065379,10.4040678707
C,-13.9344779815,-1.6393925898,2.3799799414
C,-9.747941926,4.7611008743,-5.209515432
C,-8.9995579652,-0.2325973529,11.8638841657
C,-15.3139896433,-1.4264444457,2.9654476913
C,-9.3271394602,5.3188245668,-6.5524412827
C,-10.142080094,0.2720361494,12.7189433276
C,-16.3476094147,-2.2780045498,2.2600291087

C,-10.5256284459,5.7490217053,-7.3704738253
C,-9.9262133914,-0.0648132088,14.1787625925
C,-17.7271589273,-2.065006866,2.8453608956
C,-10.1107231216,6.2043856107,-8.7531205836
C,-11.0686928496,0.4397009276,15.0339431558
C,-18.7608597778,-2.9164919692,2.1399773004
C,-11.3066456235,6.6528501185,-9.5650456839
C,-10.8528053979,0.1028420579,16.4937540547
C,-20.1404205394,-2.7034493834,2.7252592696
C,-10.9044118653,7.028300625,-10.9753092528
C,-11.9952689905,0.6072969423,17.3489892079
C,-21.1741575527,-3.5548923855,2.0198800369
C,-12.0907959117,7.5391932899,-11.7637980548
C,-11.7793592221,0.2704225456,18.8087922039
C,-22.5537213629,-3.3418226386,2.605142478
C,-11.7086789374,7.8640880644,-13.1920502785
C,-12.9218130518,0.774843633,19.6640598102
C,-23.5874805535,-4.1932370779,1.8997622967
C,-12.8863614348,8.4339173689,-13.9533310743
C,-12.7058865155,0.4379567868,21.1238568521
C,-24.9670445621,-3.9801541084,2.4850178767
C,-12.5144811032,8.7655194014,-15.3824473414
C,-13.8483292203,0.9423567756,21.9791513069
C,-26.0008227272,-4.8315478497,1.779641365
C,-13.7037346115,9.3098958612,-16.1444952229
C,-13.6323954803,0.6054643716,23.4389437896
C,-27.3803863051,-4.6184640868,2.3648920436
C,0.463416049,-4.8764588053,1.1469259886
C,-0.3507437399,-5.5845740465,0.2511519514
C,1.2477546715,-5.5552035896,2.0776202734
C,-0.3680887375,-6.9808489611,0.2854688981
C,1.2321704246,-6.9621396013,2.1138934912
C,0.4281566435,-7.688729404,1.2192089871
C,-1.9935566757,-7.0720789672,-1.4588218731
C,1.6611552269,-8.7510193206,3.6417038735
C,0.9384727312,-9.7711213119,0.2317765639
C,-2.7448526907,-8.1518738529,-2.2181715156
C,2.3797266407,-8.8236637238,4.9774387447
C,0.8207624493,-11.2455082556,0.5721008622
C,-3.7032664271,-7.5246105891,-3.2064237972
C,2.0503308613,-10.1234661771,5.6779070893
C,1.4187023151,-12.0883790422,-0.5326996527
C,-4.4633654149,-8.5856825621,-3.9732960346
C,2.7537519558,-10.213926165,7.015266152
C,1.3107221889,-13.5635890345,-0.2108066413
C,-5.4239495296,-7.9630562772,-4.9636859479
C,2.4275336573,-11.5135458724,7.7193926927
C,1.9076365071,-14.4106570822,-1.31415723
C,-6.1844215055,-9.0233625069,-5.7309182423
C,3.1303796075,-11.6044938422,9.0568950902
C,1.7996727965,-15.8859112558,-0.9929978986
C,-7.1450608631,-8.4013973077,-6.7216202612
C,2.8046266808,-12.9039781668,9.7614261801
C,2.3964234644,-16.7333737901,-2.0961012729
C,-7.9056506211,-9.4615561553,-7.4888986325
C,3.5074491685,-12.9949761562,11.0989231733
C,2.2883594553,-18.2086399511,-1.775086193
C,-8.8662429354,-8.8397563216,-8.4797391613
C,3.181817618,-14.2944278392,11.803562727
C,2.8850766428,-19.0561911194,-2.878134835

C,-9.6268800387,-9.8998842459,-9.2470033923
C,3.8846633933,-14.3854186198,13.141044434
C,2.7769404714,-20.5314602282,-2.5571711868
C,-10.5874370726,-9.2781534227,-10.2379177146
C,3.5590863141,-15.6848576698,13.8457304674
C,3.3736421255,-21.3790471228,-3.6601992346
C,-11.3480944398,-10.3382802279,-11.0051600877
C,4.2619535099,-15.7758325849,15.183200864
C,3.265461193,-22.8543166636,-3.3392573762
C,-12.3086324881,-9.7165888731,-11.9961160799
C,3.936407539,-17.0752646658,15.887913479
C,3.8621533388,-23.7019240218,-4.4422741379
C,-13.069299488,-10.7767204674,-12.763340513
C,4.6392896825,-17.1662261631,17.2253763052
C,3.7539467186,-25.1771932984,-4.1213426781
C,-14.0298302989,-10.1550608769,-13.754322542
C,4.313765783,-18.4656511679,17.9301116552
C,4.3506338076,-26.0248190281,-5.2243474963
C,-14.7905034701,-11.2151925187,-14.5215362511
C,5.0166542186,-18.5566071879,19.2675691582
C,4.242416864,-27.5000873889,-4.9034255652
C,5.3426971131,-0.0806887145,-0.526949478
C,5.6160947134,-0.7387491572,-1.7349924263
C,6.3753397993,0.4864939438,0.2178227854
C,6.9304346619,-0.8158679142,-2.2007167185
C,7.7006332745,0.4111826904,-0.2501031243
C,7.9919880519,-0.2363923368,-1.4624982025
C,6.2582566372,-2.1056228172,-4.0919551635
C,9.9356030943,0.4720048195,0.5969017231
C,9.6512624398,0.3693553253,-3.0283936599
C,6.9341255735,-2.7516993583,-5.2889287105
C,10.5300660003,0.9206024778,1.9202096782
C,11.1338343508,0.1365799147,-3.2549128103
C,5.917517024,-3.5034997451,-6.1195862754
C,11.9479113017,0.4107026646,2.0557260138
C,11.6039102126,0.8971943888,-4.4751752903
C,6.5724186606,-4.1569113501,-7.3178602545
C,12.557992274,0.8433644334,3.3718230159
C,13.0819154408,0.6773776184,-4.717450297
C,5.5592090687,-4.9107775973,-8.1523367562
C,13.9772807316,0.3359956411,3.5111840374
C,13.5563079016,1.4366074241,-5.937997578
C,6.213222832,-5.5646495477,-9.3506375617
C,14.5878144845,0.7680273974,4.8271521402
C,15.034119905,1.2170070889,-6.1809384115
C,5.2005277743,-6.3186550008,-10.1855523258
C,16.0071971498,0.2611255252,4.9669440415
C,15.5089476339,1.9760470002,-7.4014046504
C,5.8543800816,-6.9726952622,-11.3838242839
C,16.6177793935,0.6931201326,6.2828876462
C,16.9867200156,1.7564128014,-7.6444836161
C,4.8418058957,-7.7267192269,-12.218855962
C,18.037204349,0.1863721014,6.4227684983
C,17.4616448606,2.5154286387,-8.8649227322
C,5.4956343182,-8.3808284348,-13.4170974413
C,18.6477816681,0.6183832353,7.7387054845
C,18.9394000294,2.2957542783,-9.1080527668
C,4.4831101434,-9.134862502,-14.2521764108
C,20.0672310853,0.111714366,7.8786149748
C,19.4143630209,3.0547647532,-10.3284789957

C,5.1369455467,-9.7889987571,-15.4503974282
C,20.6777954992,0.5437416791,9.194551555
C,20.8921093097,2.8350620762,-10.5716315859
C,4.1244509484,-10.543043069,-16.2855013857
C,22.0972598302,0.0371192704,9.3344749173
C,21.3670935936,3.5940701092,-11.7920504442
C,4.7782967289,-11.1971887876,-17.4837105627
C,22.707813265,0.4691561534,10.6504128367
C,22.844834841,3.3743502081,-12.0352144833
C,3.7658268391,-11.9512434691,-18.3188341285
C,24.1272867166,-0.0374359539,10.7903508655
C,23.3198387844,4.1333554411,-13.2556269484
C,4.4196756601,-12.6053927233,-19.5170368654
C,24.7378357576,0.3946017277,12.1062881394
C,24.7975754899,3.9136302783,-13.4988004581
H,3.2634014948,4.6042920883,0.0316467505
H,5.0815561299,2.6174014805,-0.5323939461
H,4.9607658745,-2.7204140137,-0.133409081
H,3.0677544291,-4.5803370441,0.4962679152
H,-2.1021819892,-4.4401152568,1.8716301605
H,-3.8944230479,-2.4036857374,2.337456099
H,-3.7019464403,2.9029618938,2.2766121354
H,-1.7638642113,4.7784593685,1.7522825514
H,1.3122626132,-0.715756277,0.7202917949
H,-0.0848661695,0.8858105751,1.2351136414
H,-0.7282742738,5.2660589813,-0.5504729385
H,2.3854745278,5.1039248997,2.4504889297
H,-1.0625676793,6.7486271822,-2.2735315999
H,-2.3605011881,6.7085201851,-0.978269171
H,2.645018299,9.6864471045,2.6532423024
H,3.5087019046,8.8714010492,1.2629569732
H,-0.9309744418,9.7272165239,0.9164847243
H,-0.1426247958,9.6170949816,2.5690709932
H,-1.6544613697,9.1439747563,-2.7835765126
H,-2.9033637336,9.1308194309,-1.4806169056
H,4.3983336101,8.7597837266,4.2466017217
H,5.2643123938,8.0060460003,2.8543781338
H,1.3256582902,11.586621993,1.991335342
H,0.5591151572,11.6947791844,0.3601880811
H,-2.882346325,7.3406791856,-4.0169592428
H,-4.1065781674,7.2408300019,-2.6924540912
H,4.6977915491,11.0382132959,3.2029179459
H,5.5581502359,10.285951315,1.8046091471
H,-0.9226927754,11.9764390437,3.0740013769
H,-1.6884504829,12.0860396206,1.4419528357
H,-3.490117547,9.7706166811,-4.3717104066
H,-4.7354623871,9.6378630267,-3.0712776282
H,6.4422513007,10.1186512192,4.7704666959
H,7.3027004313,9.3676487613,3.372307542
H,0.5707965613,13.922834438,2.5034436793
H,-0.1948354696,14.0323063516,0.8721421474
H,-4.6530639853,7.9901180751,-5.6945157814
H,-5.8804254182,7.7971812166,-4.3845610645
H,6.7307810543,12.3931085594,3.7263123884
H,7.5907224253,11.6421180853,2.3279207201
H,-1.6730511523,14.3048936193,3.5826132476
H,-2.4390261261,14.4141785907,1.9515607804
H,-6.5729691382,10.1870914871,-4.6500407545
H,-5.3104330206,10.4286325798,-5.9175069024
H,8.475999442,11.4740508966,5.2930391049

H,9.3358987122,10.7231958203,3.8946408381
H,-0.1800757617,16.2516776845,3.0119053699
H,-0.9459302696,16.3609742916,1.3808637895
H,-6.4031795433,8.673931756,-7.3490401108
H,-7.6649176773,8.4187750165,-6.0838062256
H,8.7644009589,13.7483711085,4.249161747
H,9.6242937042,12.9974603224,2.8507950309
H,-2.4236528322,16.6337726455,4.0911872661
H,-3.1897634975,16.742850487,2.4602691963
H,-7.069002076,11.1015118612,-7.5172563181
H,-8.3446150019,10.8345898662,-6.2681711221
H,10.5096055995,12.8293274562,5.8158466552
H,11.3694631003,12.0784099133,4.4174954286
H,-0.9308398764,18.5806048945,3.5201870813
H,-1.6968527565,18.6897079331,1.8892421072
H,-8.1630936153,9.3536062679,-8.9844465215
H,-9.4537867197,9.1343307091,-7.7426335995
H,10.7981783454,15.1035757404,4.7719918352
H,11.6580575629,14.3526252531,3.3736641785
H,-3.9405492914,19.0716789922,2.9687242604
H,-3.1743739004,18.962717718,4.5995991527
H,-10.0953359675,11.5417660879,-7.9440198349
H,-8.7614307862,11.8191869913,-9.1281713914
H,13.4031812274,13.4335288659,4.9404090094
H,12.5433208739,14.184502851,6.3387200389
H,-1.6816329307,20.9095658289,4.0284253352
H,-2.4477315888,21.0185483962,2.397522637
H,-11.13131143,9.8572235447,-9.5124183524
H,-9.792839117,10.1332275735,-10.6885624821
H,13.6918835235,15.7077161901,3.8965522887
H,12.8320094307,16.4587061138,5.2948537489
H,-4.6913595218,21.4005745193,3.4770453
H,-3.9251576958,21.2916782598,5.1079047404
H,-11.7258871831,12.3217792774,-9.647984041
H,-10.4070288134,12.5546336452,-10.8581764848
H,14.5771052207,15.5396027057,6.8616188466
H,15.4369694401,14.7885935106,5.4633308449
H,-2.432448148,23.2385327879,4.5366342318
H,-3.1985916881,23.3474457597,2.9057523107
H,-12.8805461924,10.7104344842,-11.1461858702
H,-11.5251709079,10.7868208243,-12.3326626088
H,14.8658649371,17.8137813419,5.8177416805
H,15.7257383201,17.0627643658,4.4194581777
H,-5.4421832762,23.7295050891,3.9852929567
H,-4.6759724415,23.6206444145,5.6161463678
H,-13.3451493581,13.143682016,-11.4700651065
H,-11.9416783437,13.2834938842,-12.596698058
H,17.4707956019,16.1436245899,5.9862623504
H,16.6109283466,16.8946564453,7.384533914
H,-3.1832751448,25.5675021825,5.0448215417
H,-3.9494441543,25.6763766081,3.4139531414
H,-14.3976503891,11.4412627646,-13.0177587045
H,-12.9992821239,11.6147136614,-14.1451265693
H,16.899737559,19.1688186421,6.3406492142
H,17.7596106134,18.4177836373,4.9423812289
H,-6.1930116478,26.058459439,4.4935034569
H,-5.4267992902,25.9496149333,6.1243513675
H,-14.9407750287,13.8700610083,-13.2762556294
H,-13.5086851176,14.0935590639,-14.3522779774
H,18.6447852013,18.2496786145,7.9074615472

H,19.504652051,17.4986390904,6.5092066377
H,-3.934107797,27.8964813277,5.5529978648
H,-4.7002881718,28.0053370399,3.9221458936
H,-4.7107330793,1.2015246882,0.4386993366
H,-3.887208889,-0.691379205,4.2392334202
H,-6.2132210637,2.7762506249,-0.1015477082
H,-6.4440963949,1.1641613783,-0.9464258089
H,-8.3344030794,-0.6654588844,5.3969746002
H,-7.4023415081,0.8911177561,5.6233431036
H,-9.0905345296,-0.0906421833,1.522194153
H,-8.791322195,-1.4404390841,2.727499143
H,-8.6793277527,3.2554660455,-0.3761555882
H,-8.9024870279,1.6715271788,-1.2127709221
H,-7.0484074522,-1.6825887266,7.3426660756
H,-6.1775866461,-0.1203454199,7.5824050499
H,-10.4671250669,-0.3968716539,4.3001916429
H,-10.7610356068,0.937544677,3.1199751817
H,-7.0467885072,3.9774711428,-2.1518094488
H,-7.2296130128,2.3825865609,-2.9797762791
H,-9.2524250351,-0.5176905625,7.7371771369
H,-8.3841606108,1.0485367976,7.9704600425
H,-11.2395540265,-2.0832814261,2.5860212591
H,-11.5343726071,-0.7477871577,1.4063931671
H,-9.4969191031,4.434771674,-2.4773400021
H,-9.6937452091,2.8310958659,-3.2831474725
H,-7.9762904378,-1.5122256515,9.6681215268
H,-7.1094590623,0.0540411173,9.9018517138
H,-12.888429526,-1.0398310597,4.1788043526
H,-13.1831031103,0.2949935114,2.9995081095
H,-7.8514098849,5.1354077491,-4.2344595214
H,-7.9868359276,3.5173331959,-5.0236189789
H,-10.1786017316,-0.3505407545,10.0515488235
H,-9.3118470023,1.2157628347,10.2848222716
H,-13.6522808199,-2.7218760381,2.4662596964
H,-13.9468701367,-1.387338926,1.2867525927
H,-10.4641339089,3.9110142459,-5.3633652227
H,-10.2918594769,5.5499414941,-4.6264112935
H,-8.9028750205,-1.3440273012,11.9830905116
H,-8.036447212,0.2223708995,12.2163999488
H,-15.3016136535,-1.6785509136,4.0586672202
H,-15.5961675631,-0.3439516682,2.8793194987
H,-8.6421388996,6.1949409904,-6.3994940039
H,-8.7477781017,4.5406488498,-7.1164688675
H,-11.1051956488,-0.1828903882,12.3664219959
H,-10.238701064,1.3834689854,12.5997300148
H,-16.0654856735,-3.3604992151,2.3463167508
H,-16.3599174732,-2.0260569188,1.1667803769
H,-11.061225201,6.5822454606,-6.8437327991
H,-11.2481621041,4.89500904,-7.4584351103
H,-9.8294625526,-1.1762335556,14.2979727676
H,-8.9631192832,0.3901786237,14.5312767399
H,-17.7148643522,-2.3170302976,3.9385972312
H,-18.0092823133,-0.9825076462,2.7591778577
H,-9.3774423273,7.0508213608,-8.6671793465
H,-9.5879673592,5.3655820348,-9.2840090172
H,-12.0317929639,-0.0152591389,14.6814231477
H,-11.1653955022,1.5511273654,14.9147538476
H,-18.7731237787,-2.6645636255,1.0467238433
H,-18.4787821403,-3.998996638,2.2262632815
H,-12.0708594452,5.8322494366,-9.5977236295

H,-11.7881804487,7.535236212,-9.0652716483
H,-9.8897168733,0.5578428265,16.8462657633
H,-10.7560257632,-1.0085773359,16.6129419667
H,-20.1281648178,-2.9554398715,3.8185022236
H,-20.4224984543,-1.6209412342,2.639048732
H,-10.4609038064,6.1369989911,-11.4917707484
H,-10.1109187829,7.8222548804,-10.9389671041
H,-12.0920145636,1.7187210019,17.2298178772
H,-12.9583612823,0.1523172248,16.9964722406
H,-21.1863994095,-3.3029597378,0.9266270966
H,-20.8921087061,-4.6374045429,2.1061544325
H,-12.9084209223,6.7714232503,-11.7579390659
H,-12.4958041496,8.4624484696,-11.2676540564
H,-11.6825648747,-0.8409972307,18.9279621308
H,-10.8162726971,0.7254273601,19.1613012158
H,-22.5414848356,-3.5938016972,3.6983875092
H,-22.8357706325,-2.2593079259,2.518922749
H,-11.3378122245,6.9380182957,-13.7046527255
H,-10.8660721841,8.6057496686,-13.1953689035
H,-13.8849019724,0.3198533773,19.3115464767
H,-13.0185833625,1.8862666683,19.5449012876
H,-23.5997122583,-3.9412942956,0.8065113976
H,-23.305449091,-5.2757544363,1.9860215805
H,-13.7323142378,7.6973895333,-13.9447712823
H,-13.2486605219,9.3645022342,-13.4365055106
H,-11.7427993145,0.8929608337,21.4763631158
H,-12.6090845141,-0.6734634252,21.2430146725
H,-24.954816384,-4.2321286412,3.5782634789
H,-25.2490776019,-2.897635313,2.398797927
H,-12.1260882244,7.8467841458,-15.8955694333
H,-11.6867796435,9.5233733206,-15.3872115119
H,-14.8114176925,0.4873638566,21.6266400518
H,-13.9451145699,2.0537788997,21.8599988022
H,-26.0130489683,-4.5795973227,0.6863921967
H,-25.7187986609,-5.9140679243,1.8658850188
H,-14.5259965126,8.5468448918,-16.1545186512
H,-14.1023436104,10.2192822636,-15.6175642898
H,-13.535585648,-0.5059548933,23.5580989773
H,-12.6693054151,1.0604598261,23.7914507931
H,-27.3681646168,-4.8704315821,3.4581385371
H,-27.6624143047,-3.5359437776,2.2786810028
H,-0.9653814226,-5.0260858516,-0.468862754
H,1.8896572555,-5.0086386655,2.7847735106
H,-2.6968318027,-6.4298030258,-0.8669675709
H,-1.3904509259,-6.4321139676,-2.1551031364
H,1.9533058167,-9.6014999887,2.969697898
H,0.5486766834,-8.7483675986,3.7711949652
H,0.3825886142,-9.521227323,-0.7094015852
H,2.0108100427,-9.4602969301,0.133201792
H,-3.3028878806,-8.7979346397,-1.4913065564
H,-2.011314098,-8.8103240357,-2.7523288681
H,3.4854915987,-8.7420814557,4.8106625218
H,2.0755563651,-7.9522788069,5.6137163322
H,1.3470638703,-11.4470092997,1.5417175146
H,-0.2592954368,-11.5077013639,0.7195311202
H,-4.4276639023,-6.8624717284,-2.6623693191
H,-3.1355516481,-6.8757001303,-3.9245804907
H,2.3565851116,-10.9886815217,5.0323946077
H,0.941195383,-10.1999262602,5.8299253118
H,2.4967965437,-11.8127785943,-0.6771454759

H,0.889548747,-11.8743792133,-1.498762967
H,-5.0308566283,-9.2346401207,-3.2551839949
H,-3.7392157167,-9.2483935601,-4.5166685746
H,3.8626155152,-10.1360579882,6.8629911627
H,2.4472289208,-9.3488088039,7.6606147249
H,1.8391804178,-13.7773515782,0.7555595407
H,0.2327243625,-13.838870966,-0.0659593124
H,-6.1478779672,-7.3002855148,-4.4200635872
H,-4.8563396353,-7.3138297118,-5.6814400355
H,2.7339980969,-12.3785431723,7.0738805962
H,1.3185772301,-11.5911924882,7.8711779909
H,2.9856070832,-14.1351271091,-1.4587715076
H,1.3793843031,-14.1964937075,-2.280550545
H,-5.4605750478,-9.6863691456,-6.2743388674
H,-6.7521845228,-9.6724784402,-5.0131818014
H,4.2393125147,-11.5265740311,8.9051404586
H,2.8238021267,-10.739525204,9.7024076859
H,2.3279293481,-16.1000840387,-0.0266053569
H,0.7217278818,-16.1614558597,-0.848276962
H,-7.8689228795,-7.7384082083,-6.178201343
H,-6.5773501326,-7.7522504128,-7.4393541977
H,3.1111087348,-13.7689522388,9.115896336
H,1.6956928041,-12.9818018005,9.9132191542
H,3.4744108463,-16.4579196593,-2.2406782555
H,1.8683213562,-16.5191114566,-3.0625496236
H,-8.4734697115,-10.1106318366,-6.7711813394
H,-7.1818297312,-10.1246722671,-8.0322123618
H,4.6163756841,-12.9170370974,10.9471541902
H,3.2009099004,-12.1300094677,11.7444465536
H,2.8165000591,-18.4229067223,-0.808654835
H,1.2103907008,-18.4841165151,-1.6304414222
H,-9.5900812136,-8.1766480724,-7.936440704
H,-8.2984575243,-8.1906900068,-9.1974842372
H,3.4882994144,-15.1593986421,11.1580281481
H,2.0728927626,-14.3723077373,11.955375794
H,2.3570307681,-18.8418764387,-3.8446018519
H,3.9630774567,-18.7807850098,-3.0226865469
H,-8.9030669303,-10.5630629338,-9.7902488447
H,-10.194730212,-10.5489128493,-8.5292720352
H,3.5781467372,-13.5204492733,13.7865713536
H,4.9935843438,-14.307472975,12.9892432776
H,3.3050213611,-20.7457750521,-1.5907194167
H,1.6989528147,-20.8068827043,-2.412570791
H,-10.0196082322,-8.6291394408,-10.9556750402
H,-11.3112633408,-8.6149786053,-9.6946865752
H,2.450166383,-15.7627659702,13.9975593579
H,3.8655699946,-16.5498281819,13.20019568
H,2.8456206817,-21.1647047519,-4.6266736181
H,4.4516509194,-21.1036701158,-3.804739728
H,-10.624285217,-11.0014955181,-11.5483650733
H,-11.9159611514,-10.987275655,-10.2874135231
H,5.3708709491,-15.6978828491,15.0313774874
H,3.9554482302,-14.9108608764,15.8287283678
H,3.793509391,-23.0686576627,-2.372794381
H,2.1874618543,-23.1297054586,-3.1946816169
H,-11.7407807708,-9.0676057004,-12.7138828936
H,-13.0324519111,-9.0533761807,-11.4529228199
H,4.2428927677,-17.940236252,15.2423803533
H,2.8274906576,-17.1531899855,16.0397535197
H,3.3341437756,-23.4875673444,-5.4087519942

H,4.9401664465,-23.4265640299,-4.5868108819
H,-12.3454927246,-11.4399581798,-13.3065206893
H,-13.6371735604,-11.425695515,-12.0455823577
H,4.3327924608,-16.3012515882,17.8709027661
H,5.7482048941,-17.0882737106,17.0735391397
H,4.2819736701,-25.3915482266,-3.1548717744
H,2.6759406733,-25.4525628217,-3.9767812968
H,-13.4619670496,-9.5060931755,-14.4720939027
H,-14.7536440039,-9.4918246828,-13.2111509403
H,4.6202492814,-19.3306244554,17.2845798653
H,3.2048505743,-18.5435872934,18.0819562999
H,3.8226329344,-25.8104540323,-6.1908282273
H,5.4286481115,-25.7494653483,-5.368883923
H,-14.0667021468,-11.8784478458,-15.0647008184
H,-15.3583774784,-11.8641618119,-13.8037741012
H,6.1255682274,-18.4786496317,19.1157296726
H,4.7101690863,-17.6916296677,19.9130960677
H,4.7704250247,-27.7144531105,-3.9369476886
H,3.1644094078,-27.7754512902,-4.7588677675
H,4.7857505352,-1.1878287365,-2.2983912987
H,6.1722594479,1.0005992939,1.1692004973
H,5.7977472933,-2.8775332104,-3.4215339778
H,5.4762542185,-1.3699378294,-4.4165690873
H,10.5347741082,0.8506362721,-0.2736775824
H,9.860299947,-0.643765213,0.5355414003
H,9.0411895839,0.0064967517,-3.8963914986
H,9.4335613261,1.4521609043,-2.8377139858
H,7.7365964237,-3.4484912538,-4.9316683702
H,7.4325833115,-1.960349582,-5.9071941492
H,10.5134373853,2.0403339184,1.9757339131
H,9.8980120862,0.5377114115,2.7631724716
H,11.7053861063,0.4666074654,-2.3481850648
H,11.3219565398,-0.961006854,-3.3849892798
H,5.4195374819,-4.2871038925,-5.4893764952
H,5.116507226,-2.79848015,-6.4666676915
H,12.571312689,0.7975932777,1.2067504744
H,11.9544250795,-0.709339046,1.9878517446
H,11.4040264702,1.9927884719,-4.3379679261
H,11.0215793758,0.5641259107,-5.3746422017
H,7.3735802725,-4.8615831737,-6.9707974899
H,7.0710141814,-3.3736373355,-7.9477138498
H,12.5499305915,1.9632354709,3.4399906802
H,11.934665133,0.456096506,4.2205562167
H,13.6640490665,1.0096618443,-3.817711042
H,13.2816163571,-0.4181824405,-4.8542300328
H,5.0605565126,-5.6937607871,-7.5221418321
H,4.7580104845,-4.2059985565,-8.4990563801
H,14.6004350866,0.7232187335,2.6623244876
H,13.984950463,-0.7838688853,3.4426330479
H,13.3563775928,2.5320976353,-5.8009625947
H,12.9738412575,1.1044870018,-6.837588247
H,6.712058062,-4.7817605049,-9.9807845896
H,7.0143818367,-6.2694748913,-9.0039155544
H,14.5798123247,1.8878678275,4.8958764225
H,13.9647230374,0.3805960257,5.6759761331
H,15.6165905956,1.5490937225,-5.281336299
H,15.2340790407,0.1215223583,-6.3179208466
H,4.7016616047,-7.1015117513,-9.5553921281
H,4.3993720638,-5.6138585374,-10.5323092521
H,16.6302780634,0.6484521652,4.1180814354

H,16.0151143408,-0.8587217361,4.8982920032
H,15.3090809072,3.0715376838,-7.2643391852
H,14.9264082894,1.64408896,-8.3010021631
H,6.6555121722,-7.6775258541,-11.0370731288
H,6.3533279089,-6.1898837733,-12.0139723355
H,16.6097060921,1.8129560883,6.3516359193
H,15.9947291624,0.3056750709,7.1317278378
H,17.5692581091,2.0883842048,-6.7448862778
H,17.1866164258,0.6609272874,-7.7815210933
H,4.3428418742,-8.5095260046,-11.588716684
H,4.0406844671,-7.0219070098,-12.5656543703
H,18.6602489192,0.5737529498,5.5739036711
H,18.0452313467,-0.9334699085,6.3540840758
H,16.8790696058,2.183535821,-9.7645202896
H,17.2618218052,3.6109222265,-8.7278336168
H,5.9946353064,-7.5980472968,-14.0472391668
H,6.2967473638,-9.0856576865,-13.070307138
H,18.0247530033,0.2309281807,8.5875540539
H,18.6396611502,1.7382189245,7.8074462731
H,19.5219714191,2.6276648562,-8.2084560157
H,19.1392451089,1.2002640269,-9.2451201654
H,3.6820037332,-8.4300461841,-14.598999683
H,3.984103512,-9.9176478889,-13.6220458121
H,20.0753214352,-1.0081253013,7.8099141709
H,20.6902551743,0.4991316433,7.0297511254
H,18.8317695207,2.7229033076,-11.2280763373
H,19.2145661794,4.1502607193,-10.1913777401
H,5.6359705155,-9.0062297289,-16.0805343695
H,5.9380500017,-10.4938236255,-15.1035816034
H,20.6696453012,1.6635774989,9.263286836
H,20.0547789618,0.1562774492,10.0434034806
H,21.4746988803,3.1669383861,-9.6720347316
H,21.0919222885,1.7395683036,-10.7087157709
H,3.3233485616,-9.8382289044,-16.6323378116
H,3.6254262866,-11.3258197548,-15.655374758
H,22.7202728482,0.4245604688,8.4856134182
H,22.1053891684,-1.0827190486,9.2657635013
H,20.7844906521,3.2622257615,-12.6916480691
H,21.1673118495,4.6895676458,-11.6549436504
H,5.2773330971,-10.4144240976,-18.1138432466
H,5.5793992586,-11.9020079048,-17.1368805347
H,22.0848027464,0.0816861088,11.4992656836
H,22.6996444943,1.5889919021,10.7191466841
H,23.4274347154,3.7062044524,-11.1356167291
H,23.0446291003,2.2788543674,-12.172308239
H,2.9647231313,-11.2464332241,-18.6656748798
H,3.266792743,-12.7340149884,-17.6887089835
H,24.7502934759,0.3500188099,9.9414908336
H,24.1354397042,-1.1572730939,10.7216287209
H,22.7372296806,3.8015227689,-14.1552248129
H,23.1200620954,5.2288532025,-13.1185179561
H,4.9187218366,-11.8226334226,-20.1471672973
H,5.2207829949,-13.3102040442,-19.1702045959
H,24.7296547532,1.5144364823,12.1750297676
H,24.114828952,0.007133823,12.955143613
H,25.3801846063,4.2454652523,-12.5992024812
H,24.9973657965,2.8181345138,-13.6358965199
N,2.060531358,1.514183479,0.5684735098
N,-0.8533219891,-1.3126702235,1.4167646711
N,1.976808056,-1.439774832,0.5810297144

N,-0.7573968543,1.6070203595,1.3317427553
O,-0.7579781014,8.0139388292,-0.6298244828
O,2.6734719784,7.6089730406,2.7327580935
O,1.1033549618,9.2279825341,0.9271685813
O,-7.41305923,1.377700782,0.8977353513
O,-6.2893991131,-0.8078788358,5.0515037476
O,-8.2211743039,0.4830491019,3.3396125545
O,-1.1103860354,-7.7693847397,-0.5680015816
O,2.0998590588,-7.5287815215,3.0225555424
O,0.3375442596,-9.0644003528,1.3346632373
O,7.299331597,-1.4122402727,-3.3878805739
O,8.6292851112,1.0706256496,0.5264433692
O,9.3076787379,-0.3985373174,-1.8582305092
C,19.5854687395,19.6617562118,6.5622579582
H,19.7934770617,19.7727764132,5.4653327911
H,18.9336171218,20.5238091266,6.8635754476
C,20.8866901061,19.7158037872,7.3334243424
H,20.6789582725,19.6049415838,8.4303853181
H,21.5387844403,18.853943966,7.0321991479
C,21.6205537525,21.0167049504,7.0859768326
H,20.9662685514,21.8774828561,7.3863849084
H,21.8260515913,21.1265224817,5.9882595619
C,22.9168773948,21.0749160669,7.8518912257
H,23.5923927617,20.2389183528,7.5480952806
H,23.4411529832,22.0411076242,7.6553429937
H,22.7302960692,20.9918901715,8.9499802725
C,-5.99144575,28.5714682755,5.5655667363
H,-6.9438192852,28.3874221783,5.0016972655
H,-6.1776215016,28.2785769618,6.6325218704
C,-5.6374100456,30.0414346868,5.497347055
H,-4.6851620436,30.2257809918,6.0612685428
H,-5.4513041782,30.3346308563,4.4304987004
C,-6.74184412,30.9018033173,6.0736479334
H,-6.9275623606,30.6060909747,7.1401080873
H,-7.6936849389,30.7149333551,5.5094222423
C,-6.3936277179,32.3663434793,6.0078083243
H,-7.2204392988,32.9818990654,6.4373405271
H,-5.4609200328,32.5763601585,6.5852639109
H,-6.2290857037,32.6855052527,4.9501857466
C,-15.117000211,12.9348480321,-15.2222852574
H,-15.8740510235,12.1697806964,-14.9052504125
H,-14.4552920593,12.4454655701,-15.9863386596
C,-15.82140621,14.1195165777,-15.8473564103
H,-15.0628479735,14.8805171035,-16.1701628245
H,-16.4795208854,14.6125178797,-15.0840663969
C,-16.6558634521,13.698595215,-17.0385392151
H,-17.4186117903,12.9432674189,-16.7114039639
H,-15.9988877696,13.1945317525,-17.7974722763
C,-17.3493741195,14.8768550775,-17.671854894
H,-16.604062101,15.6290769429,-18.0270852929
H,-17.9593359282,14.5446279814,-18.5462770323
H,-18.0274478267,15.3763948943,-16.9382387091
C,-13.3294272531,9.6716606583,-17.5657565412
H,-12.4964728186,10.4234596874,-17.5548890583
H,-12.9462722076,8.7616606958,-18.0982356806
C,-14.5144389558,10.2393809861,-18.3167943489
H,-15.3422910744,9.4827169698,-18.3418209121
H,-14.9073236092,11.1407030688,-17.7733389848
C,-14.1390993675,10.62688748,-19.7312708347
H,-13.7746070155,9.7205504464,-20.2832217282

H,-13.2900815645,11.3602959405,-19.7050493765
C,-15.3077449496,11.2326493176,-20.4648136203
H,-15.0084868198,11.5236154638,-21.5004755135
H,-16.151457074,10.503994235,-20.5327569602
H,-15.6754840425,12.1445548818,-19.9336308719
C,-28.4142145598,-5.4698249914,1.6595520741
H,-28.1321431999,-6.5523424896,1.7457563444
H,-28.4263881577,-5.2178809324,0.5662939802
C,-29.7937269405,-5.2568401949,2.2446843858
H,-29.7818729776,-5.5088599769,3.3378965213
H,-30.0761084898,-4.174436161,2.1585020176
C,-30.8288287287,-6.1078424493,1.5400629371
H,-30.5439169937,-7.1898359957,1.6260235886
H,-30.8381400216,-5.8554833195,0.4466735929
C,-32.2040300365,-5.89900466,2.1194383011
H,-32.9509602318,-6.5351125182,1.5860657642
H,-32.2206586987,-6.1689608518,3.2031137847
H,-32.5156783094,-4.8310105489,2.0206056042
C,-14.7748038829,1.1098332478,24.2943003047
H,-15.7378918919,0.6548598431,23.9417412595
H,-14.871607858,2.2212547007,24.1750952844
C,-14.5589835117,0.773016823,25.7540167997
H,-13.5960369388,1.2279883443,26.1068648699
H,-14.4622749697,-0.3383475764,25.8735255049
C,-15.7009833151,1.2769538247,26.6107630359
H,-16.6633116881,0.8220653579,26.2554144636
H,-15.7971053302,2.388324739,26.4887453952
C,-15.4903174292,0.9437926335,28.0650986536
H,-14.5483180122,1.4092241237,28.4439342498
H,-16.3427905207,1.323918283,28.6781372482
H,-15.4168462245,-0.1612484788,28.2099815153
C,4.6911752338,-19.8560111489,19.9723615143
H,4.9976256586,-20.7209745474,19.3267891129
H,3.5822466455,-19.9339523568,20.1241538956
C,5.3939668102,-19.9470645671,21.3097429256
H,5.0875841908,-19.0822995222,21.955558553
H,6.5029119078,-19.8692798809,21.1582304116
C,5.0692746529,-21.2463569543,22.0158194549
H,5.3749771948,-22.1099951747,21.3677861936
H,3.9597192236,-21.3230450724,22.1650797538
C,5.7669397233,-21.3413572243,23.3480007304
H,5.4560095464,-20.5025772915,24.0167879678
H,5.5140821019,-22.3057449396,23.8510328343
H,6.8750754819,-21.2916379908,23.2173552789
C,4.8390901863,-28.347769335,-6.0063928634
H,4.3111045724,-28.1333501523,-6.9728776498
H,5.9170950834,-28.0723613461,-6.150945413
C,4.7309369026,-29.8229613299,-5.6856000916
H,5.2589673918,-30.0376800686,-4.7192425774
H,3.6530341121,-30.0986741174,-5.5411244115
C,5.3274045346,-30.6720448565,-6.7880587614
H,6.4049541734,-30.3938180792,-6.9321093352
H,4.7991019693,-30.4548072144,-7.7539657277
C,5.2219253031,-32.1417414379,-6.4728860945
H,4.1534677222,-32.4431532895,-6.3501938358
H,5.6671012516,-32.7488243839,-7.297684552
H,5.7636438843,-32.3819920894,-5.5261410878
C,-15.7510482872,-10.5936042788,-15.5125473618
H,-16.474822935,-9.9303229391,-14.9693654036
H,-15.1831603007,-9.9446064893,-16.2302821819

C,-16.5117272409,-11.6535771106,-16.2797648761
H,-17.0798176424,-12.3026296164,-15.5622894328
H,-15.78821037,-12.3169143716,-16.8231554758
C,-17.4729166796,-11.0331957568,-17.2714174501
H,-18.194713387,-10.3689951289,-16.7263301503
H,-16.9031647767,-10.3832728984,-17.9871379858
C,-18.2325574599,-12.0849922286,-18.0376667608
H,-18.9346627971,-11.6064800645,-18.7623039887
H,-17.5312233573,-12.7416354934,-18.6072757951
H,-18.826240245,-12.7273214096,-17.3430742915
C,3.4072628788,-13.3594699423,-20.3522068825
H,2.6061387851,-12.6546485603,-20.6989991476
H,2.908202995,-14.1422047383,-19.7220449214
C,4.0609619498,-14.0136184644,-21.5503573531
H,4.5599774527,-13.2311031168,-22.1807694645
H,4.8620296338,-14.7185945168,-21.2038515142
C,3.0493949275,-14.7682653095,-22.3866424946
H,2.2482755332,-14.0619243812,-22.7309719274
H,2.5503209833,-15.549347308,-21.7541005112
C,3.6958908145,-15.4212754089,-23.5808321507
H,2.933218905,-15.9715805292,-24.182928734
H,4.1775303413,-14.6558702797,-24.2363340417
H,4.4803859751,-16.1472896641,-23.2568327398
C,25.2726422452,4.6726153722,-14.719199422
H,24.6899812227,4.3408037473,-15.6187797022
H,25.0728106315,5.7681088915,-14.5820842766
C,26.7502691639,4.4529777857,-14.9624571927
H,27.3331710288,4.784842571,-14.0630918479
H,26.9503666348,3.3575871606,-15.0997369707
C,27.2267981333,5.2116162316,-16.1829157723
H,26.6416671606,4.8794916833,-17.081017832
H,27.02445979,6.3066732428,-16.0444129604
C,28.6976349725,4.9958447478,-16.4289835302
H,28.9152539105,3.9128183561,-16.5939082588
H,29.0277258231,5.5633987552,-17.3322808507
H,29.2990668837,5.3438439344,-15.5545227743
C,26.1573169878,-0.111948849,12.2462848562
H,26.7802920762,0.2754921713,11.3973863966
H,26.1654629256,-1.231788784,12.1775022219
C,26.76792295,0.3199988741,13.5621029232
H,26.1452231479,-0.0674333089,14.4111660246
H,26.7600190438,1.4397948514,13.6310935422
C,28.1878153607,-0.1860215536,13.7034488383
H,28.808640785,0.2012076653,12.8526249318
H,28.1938666083,-1.3059442473,13.6326682862
C,28.7998216783,0.2417182461,15.0122479819
H,28.2087125989,-0.1556028073,15.8726152876
H,29.8453702084,-0.1415131618,15.0958187156
H,28.8251383438,1.3556046931,15.0904837124

Au0⁺ (DFT)
–2048.2451046 hartree
C,-2.8606637445,-1.109534015,-0.0000295681
C,-4.2291330195,-0.6709902412,-0.0002256063
H,-5.0797876179,-1.33595995,-0.0003787823
C,-4.2258784863,0.6912223249,-0.0000224615
H,-5.0733587113,1.3602303188,-0.0000117733
C,-2.8553285538,1.1232394359,-0.0000160246
C,-1.1095696562,2.8606543434,0.0000961237
C,-0.6710197859,4.2291214106,0.0001888305

H,-1.3359879135,5.0797767579,0.000230587
C,0.6911934207,4.2258644743,0.0002213824
H,1.3602049124,5.0733418386,0.0002961735
C,1.1232058775,2.8553128449,0.000143492
N,-2.0380349172,0.0048885338,0.0000341801
C,2.8606249815,1.1095591014,0.0000957162
C,4.2290903172,0.6710060735,0.0001350807
H,5.0797485177,1.3359707605,0.0001777783
C,4.2258316852,-0.6912064199,-0.0000627263
H,5.073308983,-1.3602185117,-0.0001756989
C,2.8552781867,-1.1232136886,0.0000696687
C,1.109527061,-2.8606316997,0.0000668999
C,0.6709768606,-4.2290996092,0.0000953847
H,1.3359445189,-5.0797551677,0.0001385574
C,-0.6912354244,-4.2258433093,0.0000780102
H,-1.3602453843,-5.0733215586,0.0001060877
C,-1.1232506236,-2.8552911743,0.0000269267
N,2.0379898252,-0.0048663699,0.0001048638
N,0.0048550926,2.0380236729,0.0000765402
N,-0.0048995791,-2.038001264,0.0000211484
C,2.459312679,2.4475978705,0.000140484
C,2.4475671753,-2.4593211082,0.0000699384
C,-2.4593575103,-2.447575597,-0.0000177378
C,-2.4476114226,2.459344323,0.0000379327
Au,-0.0000226496,0.0000114417,0.0000680946
C,3.5233612813,3.5064951908,0.0002197774
C,4.0258591943,4.0058592439,1.2112552577
C,4.0253488506,4.0065799642,-1.2107168019
C,5.0166338447,4.9917685529,1.2099501585
H,3.6418683101,3.6232307718,2.1530175525
C,5.0161446679,4.9924817065,-1.209238415
H,3.6409804899,3.6245157931,-2.1525545451
C,5.5130665801,5.4863605506,0.0003956213
H,5.3987758501,5.3715035815,2.1528048908
H,5.3978922546,5.3727609367,-2.1520334629
H,6.282761758,6.2522026343,0.0004709064
C,3.5064701888,-3.5233627413,0.0000380697
C,4.0063790913,-4.0254468899,1.2110206447
C,4.0060228764,-4.0257524512,-1.2109513941
C,4.9922872023,-5.0162225003,1.2096108806
H,3.6241616859,-3.6411466682,2.1528237812
C,4.9919359147,-5.0165370988,-1.2095777462
H,3.6235438916,-3.6416970335,-2.1527483645
C,5.486349482,-5.513054716,0.0000038056
H,5.3724367513,-5.3980514936,2.1524253912
H,5.3718053326,-5.3985928055,-2.1524131857
H,6.2521964736,-6.2827450181,-0.0000021245
C,-3.5234098454,-3.506467918,-0.0000995338
C,-4.0252777702,-4.0064636191,-1.2111358133
C,-4.0260458191,-4.0059036623,1.2108360619
C,-5.0160762867,-4.992348854,-1.2098324756
H,-3.6408021167,-3.6243206606,-2.1528974766
C,-5.0168334684,-4.9918135434,1.2093560102
H,-3.6421620709,-3.6233540269,2.1526744122
C,-5.513133519,-5.4863153854,-0.0002788013
H,-5.3977398696,-5.3725630087,-2.1526879068
H,-5.3990711391,-5.3716017237,2.1521504245
H,-6.2828359551,-6.2521501776,-0.0003554383
C,-3.5065102179,3.5233914387,0.0000668787
C,-4.006491238,4.0253334175,1.2110783551

C,-4.0059769759,4.025936065,-1.2108937212
C,-4.9923945684,5.0161139509,1.2097258316
H,-3.624338658,3.6409140811,2.152859087
C,-4.9918843974,5.0167263003,-1.2094627576
H,-3.6234338098,3.6419992486,-2.1527130593
C,-5.486376098,5.5130962703,0.0001475017
H,-5.3726046594,5.3978283843,2.1525623106
H,-5.3716872048,5.398902815,-2.1522760775
H,-6.2522183786,6.2827912558,0.0001859876

TBA⁺-NiO⁻ (DFT)
–5439.3230725 hartree
C,-110.1582835282,67.5072624404,11.3572138526
C,-111.2988594729,67.8345946756,12.1299706092
C,-112.255399262,68.2981558759,11.2526075354
C,-111.6936347129,68.1953522296,9.9451047253
C,-112.4139018144,68.2652907421,8.7413475694
C,-111.8734953717,67.9249154529,7.5157316465
C,-112.6280087974,67.6679607812,6.3173939718
C,-111.7362011044,67.3129063294,5.3520056189
C,-110.4196760183,67.4240145886,5.9325870225
C,-109.2262689482,67.4350940632,5.2081819079
C,-108.0195528927,67.8257070162,5.7938770421
C,-106.8547847696,68.2459874201,5.0530703453
C,-105.9290028889,68.6594000432,5.9602965786
C,-106.4856526467,68.4239718714,7.2686275076
C,-105.7871640005,68.4629579881,8.4618562362
C,-106.3114503251,67.9745784667,9.6733332404
C,-105.5504025452,67.6825064036,10.8418275218
C,-106.4310651533,67.1694070556,11.7693312614
C,-107.714140957,67.1928779433,11.1714299763
C,-108.9244878576,67.024242827,11.949905967
C,-113.8502965829,68.645039439,8.8067955728
C,-114.2814525494,69.9033213591,8.3883500662
C,-115.6049278987,70.3131991556,8.4967009703
C,-116.5518257026,69.4331312997,9.0126138469
C,-116.1610995906,68.1594282725,9.4198846681
C,-114.824561469,67.7809918357,9.3151874184
C,-109.2306464766,67.1236767592,3.7567655762
C,-109.8599172635,67.9313618618,2.8054071241
C,-109.8502404997,67.6299104589,1.4471060524
C,-109.1761820772,66.4962649966,1.0018743736
C,-108.5241839892,65.6753938718,1.9183693062
C,-108.5594880276,65.9938559419,3.2731063968
C,-104.4132826299,69.0286601043,8.4882022564
C,-103.3285388365,68.4525033895,7.8203740919
C,-102.0633259081,69.037428421,7.8257664474
C,-101.8496981713,70.2201344235,8.528721156
C,-102.9050148058,70.8102088883,9.2197823548
C,-104.1527676871,70.2016886989,9.2028090381
C,-108.0422193734,72.7338297946,8.4883219764
C,-107.0252351855,72.0237596127,7.5993991946
C,-105.7772892832,72.8952939056,7.4013510149
C,-104.8225563125,72.3126394139,6.3572945429
C,-108.5389551863,70.8958144576,10.1128740077
C,-107.8225167754,71.5752790859,11.2774190255
C,-107.5127814633,70.5619701326,12.3932070375
C,-108.7327405229,70.204266447,13.2442354507
C,-110.1824423982,72.7427042387,9.6974920547
C,-111.3926329991,72.019785294,10.2745352567

C,-112.3719429459,73.0195504151,10.9060887646
C,-113.7225489259,72.368277108,11.2140003603
C,-109.7604691123,70.989722812,7.9698367312
C,-110.4527307249,71.7825818878,6.8698142571
C,-110.9040533418,70.8570235976,5.7359015539
C,-111.6810810166,71.6079142521,4.6560387086
F,-113.3929200212,70.7985393046,7.9038843231
F,-115.9591709715,71.5560113081,8.1430728439
F,-117.8275490882,69.8105792298,9.1228625256
F,-117.0712330462,67.3107565513,9.9068891283
F,-114.4835788822,66.5548921822,9.7205563652
F,-110.4959414813,69.0527328998,3.1873390541
F,-110.4654274077,68.4307568781,0.5677477799
F,-109.1532452044,66.19886041,-0.3001805473
F,-107.8779026707,64.5848645163,1.491663589
F,-107.9266324596,65.1793400567,4.1252056994
F,-103.4852462852,67.3126798044,7.1382507244
F,-101.0499313946,68.4634634126,7.169995265
F,-100.6433272522,70.7915203542,8.5318637108
F,-102.7235923394,71.9654482367,9.8726989641
F,-105.1532624936,70.8242870118,9.8599333317
H,-111.3499645195,67.7155174562,13.2014906181
H,-113.2618867939,68.6210283882,11.4757924417
H,-113.7042546274,67.7249293259,6.2417564093
H,-111.9422485023,67.0354528692,4.3295484665
H,-106.7766186059,68.2461369747,3.9759825793
H,-104.9398553928,69.0422623684,5.7621902266
H,-104.4832169838,67.8207667349,10.9391027621
H,-106.2489465909,66.8452439967,12.7825598537
H,-107.551918226,73.2016570524,9.3418834586
H,-108.5489547857,73.5231457485,7.9312831909
H,-106.7228668217,71.069442826,8.0321025359
H,-107.4706346397,71.7960706846,6.6257095953
H,-105.2586055902,72.995200697,8.3623259886
H,-106.0748313696,73.9079963798,7.0986749289
H,-109.3627956607,70.272319881,10.4538901962
H,-107.8558883781,70.2371036304,9.5812885341
H,-106.8864294914,72.0170218752,10.9297944886
H,-108.4280896267,72.3826177074,11.7039200666
H,-106.7354171249,70.9925569961,13.0339344435
H,-107.0766477242,69.6612479154,11.9555112345
H,-109.6705280794,73.3301905833,10.460055979
H,-110.4987087325,73.4322506606,8.9139202195
H,-111.0985157147,71.2830326492,11.0271487801
H,-111.9113599317,71.4736200263,9.4854237062
H,-111.9323404091,73.4332118398,11.8221037372
H,-112.5266045385,73.868061889,10.2264500152
H,-110.4579291209,70.3160144585,8.4631590422
H,-108.9625036276,70.3660739934,7.5698827359
H,-111.3307755496,72.3018146564,7.2673603678
H,-109.7852260824,72.5486984821,6.4582942922
H,-111.5351329946,70.0687820775,6.1489766868
H,-110.033741452,70.3596454453,5.297113132
H,-103.9339739454,72.9401436161,6.244002827
H,-105.3080851026,72.233877844,5.3791539951
H,-104.492154592,71.3101869816,6.6418738758
H,-108.5054681057,69.3834302008,13.9286760159
H,-109.574837879,69.8677626807,12.6335313633
H,-109.0666534168,71.0664680517,13.8332930662
H,-114.372035892,73.0458387471,11.7751492311

H,-113.5934980537,71.4553876275,11.8048934286
H,-114.2402527116,72.1017665307,10.2879229934
H,-111.9752959203,70.9287774287,3.8530620022
H,-111.0824950392,72.4118998808,4.2123447452
H,-112.5909456419,72.0563488731,5.0705004763
N,-110.3764378536,67.7577817796,10.0274178904
N,-110.5179967642,67.7425874539,7.2694871384
N,-107.7958382051,67.9628528778,7.14312011
N,-107.6442450924,67.6610737369,9.8800111038
N,-109.1319092565,71.8426940986,9.066696733
Ni,-109.0827353636,67.7343644516,8.5776958388
O,-108.8916636782,66.6271128661,13.1264269055

Au0^{+}-NiO^{-} (DFT)
–6801.2902921 hartree
Au,-64.5077184835,42.867938224,-6a4.3439049396
C,-63.5145823788,40.0523605752,-63.6388563662
C,-62.3631842919,39.3792709993,-63.1037460992
C,-61.3801288622,40.3088057996,-62.9399047224
C,-61.8971576425,41.5724618647,-63.3834635834
C,-61.1661295641,42.7580708679,-63.4293913556
C,-59.714965815,42.7047532239,-63.0910345347
C,-59.2237401376,43.3837918953,-61.9663755054
C,-57.8621029139,43.3376227425,-61.6692757202
C,-56.9829985514,42.6209498266,-62.4837419409
C,-57.4694579487,41.9417748829,-63.6020276111
C,-58.8301889008,41.9819790259,-63.9039113203
C,-61.7078389233,43.9891543841,-63.803426208
C,-60.949640269,45.1953310114,-63.9615567582
C,-61.8169477011,46.185165274,-64.3174913738
C,-63.1337887928,45.6146183105,-64.3772334085
C,-64.3000104777,46.3157241531,-64.7012927276
C,-64.1979685694,47.7830054228,-64.9390534536
C,-63.6309501562,48.633917763,-63.9791821528
C,-63.5530767268,50.0064383629,-64.2041243272
C,-64.0416197921,50.5537725584,-65.3910303344
C,-64.5838844858,49.713420434,-66.3643268572
C,-64.6563523707,48.3397653979,-66.1437857667
C,-65.5422088855,45.7010168047,-64.8899477583
C,-66.7732181871,46.3867294234,-65.1589805865
C,-67.7225262726,45.447461831,-65.4324242063
C,-67.1059308849,44.156134969,-65.3266595112
C,-67.7313743761,42.9367737495,-65.6185801007
C,-69.0624157588,42.9716402162,-66.2876974196
C,-70.1591758376,42.2710918004,-65.7649622002
C,-71.3998332437,42.3197179991,-66.3969182687
C,-71.568529017,43.0724240885,-67.5598044194
C,-70.4790110883,43.7577571226,-68.0993856681
C,-69.2359849358,43.7041780763,-67.4726584478
C,-67.1512816807,41.6932323581,-65.356341615
C,-67.7409837938,40.4211932547,-65.6790029675
C,-66.9235499607,39.4480234913,-65.1941692786
C,-65.7802255812,40.0888209115,-64.6045684621
C,-64.6946549929,39.4205885967,-64.0398291213
C,-63.2483118411,43.7126321147,-60.9513212212
C,-63.8625088698,44.9987010827,-61.0988448435
C,-64.1024500262,42.5658424456,-60.8878050227
C,-63.1218069269,46.1804290316,-60.8565701098
C,-63.6097453089,41.3206084915,-60.4360634286
C,-64.0057817549,47.2225235513,-60.9674900685

C,-64.6825759013,40.4661040626,-60.4027790936
C,-65.2700893901,46.6547415396,-61.3120676461
C,-65.8137285999,41.2078569825,-60.8575474186
C,-66.4080198194,47.4067520935,-61.6210693875
C,-67.0894616524,40.660953378,-61.0389883526
C,-66.3192751355,48.8874835878,-61.4867806748
C,-67.3184463139,39.2707670111,-60.556520072
C,-66.4139349152,49.7539566731,-62.5788325928
C,-67.2138222624,38.9701103482,-59.1920538925
C,-66.2589465107,51.130749829,-62.4536213809
C,-67.3621325043,37.6738394746,-58.7069755306
C,-66.0134211183,51.6836057545,-61.2011703797
C,-67.6380635787,36.6336081981,-59.5915311209
C,-65.9363574461,50.8535189684,-60.0853000909
C,-67.7715923703,36.9019140261,-60.9495377061
C,-66.0953639256,49.4789742337,-60.2369976355
C,-67.6095305547,38.2047172012,-61.409506602
C,-67.5919159513,46.8364023525,-62.0407420278
C,-68.1330643404,41.366982116,-61.5974595398
C,-68.7536675256,47.6001068097,-62.4115825205
C,-69.4186693978,40.7907293354,-61.8855473557
C,-69.7160637645,46.7122134125,-62.760037548
C,-70.1870101448,41.7775592367,-62.4071980815
C,-69.1561915989,45.3956085987,-62.5796036572
C,-69.3896006798,42.980516337,-62.4040467955
C,-69.9022426784,44.2321162028,-62.7337563768
C,-71.3357518332,44.3423225258,-63.1195401882
C,-72.3567221921,43.9740914839,-62.2316464582
C,-71.7506727734,44.816524844,-64.3672130454
C,-73.7024307228,44.0090276397,-62.5863256843
C,-73.0878104407,44.8651361577,-64.7468084214
C,-74.0709268037,44.452283141,-63.854025582
C,-64.7719758917,37.9372557311,-63.8872895692
C,-64.7988223171,37.3659232546,-62.6083175258
C,-64.8906981441,35.9842047464,-62.4515228264
C,-64.9487930016,35.1543037504,-63.5718527239
C,-64.9058149691,35.713595047,-64.8497108039
C,-64.8146263575,37.0964935533,-65.0072785242
F,-66.6560647198,49.2789388334,-63.8100324354
F,-66.956665692,39.9407872808,-58.3091611821
F,-67.2476758486,37.4204565773,-57.3985722176
F,-66.3235884587,51.9202718229,-63.5333881205
F,-67.7726426218,35.3835267011,-59.1378840506
F,-65.856537992,53.0039048798,-61.0688511083
F,-65.717258062,51.3834593344,-58.8767166375
F,-68.0190288131,35.9053016607,-61.8080344129
F,-66.0192541463,48.7175727343,-59.1396978682
F,-67.7262029881,38.4126767265,-62.729476588
F,-72.0550478417,43.5599620915,-60.9944275282
F,-70.8527878946,45.2475399728,-65.2660348483
F,-74.6450467608,43.6339090428,-61.713520004
F,-73.4272195674,45.2864068873,-65.9720037942
F,-75.3592476554,44.4855648998,-64.2074796245
H,-62.3229065177,38.3240101877,-62.885920403
H,-60.3838399796,40.166522127,-62.5520289747
H,-59.9302505899,43.9073670386,-61.332362729
H,-57.4882007156,43.857237428,-60.7920933318
H,-55.9236258509,42.5891326967,-62.247067228
H,-56.7910889602,41.384729858,-64.2412336224
H,-59.2127850977,41.4610873189,-64.776542075

H,-59.8825420732,45.2622407565,-63.8212797917
H,-61.5890501556,47.2153830847,-64.5371365143
H,-63.2828143785,48.2096282204,-63.0467359618
H,-63.1266802333,50.6512228591,-63.4414969231
H,-63.9983455734,51.625359377,-65.5565441199
H,-64.9498601326,50.1274580565,-67.2988152346
H,-65.068842558,47.6884503703,-66.9070416987
H,-66.9003194981,47.4533341766,-65.1092010171
H,-68.7638547498,45.6074151962,-65.6444994029
H,-70.0392573566,41.714837778,-64.844053064
H,-72.2413786024,41.7849323096,-65.9666119942
H,-72.5401929872,43.1259152165,-68.040110103
H,-70.5968659493,44.3356967904,-69.0107994785
H,-68.3895187352,44.2330673442,-67.8981659075
H,-68.6741652284,40.2956368216,-66.202633075
H,-67.0755604931,38.3817537044,-65.2232153533
H,-62.0719131734,46.1869193973,-60.609292357
H,-62.5794021239,41.1484928252,-60.1687990587
H,-63.8132023947,48.2756325339,-60.831239156
H,-64.6988292994,39.435426899,-60.0823227523
H,-68.8215384285,48.6766003028,-62.3900769311
H,-69.6937706443,39.7629445657,-61.70929318
H,-70.7277764023,46.9180743295,-63.0729498883
H,-71.2103218027,41.7118602733,-62.7420355686
H,-64.7732385693,37.5315356765,-66.0009758609
H,-64.7680910516,38.0187041365,-61.744493022
H,-64.9279737352,35.5568737163,-61.4541911514
H,-65.0258416695,34.0785509532,-63.4495045214
H,-64.9396225037,35.0735440818,-65.7260330372
N,-63.2030810994,41.3871783833,-63.7932179827
N,-63.0343183928,44.2701696173,-64.066647641
N,-65.7870729916,44.3393069527,-64.9531352668
N,-65.9480723169,41.4578107604,-64.7164466503
N,-65.183695567,45.2690883907,-61.3775699083
N,-65.4565159085,42.5166036385,-61.1535147881
N,-67.8480457202,45.4711545694,-62.1478084739
N,-68.1151183973,42.7201738798,-61.9403658301
Ni,-66.6453592896,43.9926656031,-61.6790704279
O,-62.0078065329,43.5983263028,-60.8041674385

1-4. Examination of organized structures

Absorption spectroscopy. UV-visible absorption spectra were recorded on a Hitachi U-3500 spectrometer.

Atomic force microscopy (AFM). AFM measurements were carried out with an Olympus LEXT OLS3500 in a dynamic force mode (tapping mode) using a silicon (100) substrate.

Differential scanning calorimetry (DSC). The phase transitions were measured on a differential scanning calorimetry (Shimadzu DSC-60).

Polarizing optical microscopy (POM). POM measurements were carried out with a Nikon OPTIPHOT-POL polarizing optical microscope equipped with a Mettler FP82 HT hot stage.

Synchrotron X-ray diffraction analysis (XRD). High-resolution XRD analysis was carried out using a synchrotron radiation X-ray beam with the wavelengths of 1.00 Å, 0.71 Å (wide-angle XRD), and 0.83 Å (XRD for the sheared samples) on BL40B2 at SPring-8 (Hyogo, Japan). A large Debye-Scherrer camera with camera lengths of 541.6 mm for VT-XRD of **2H8** and **Au8**$^+$-Cl$^-$, 543.0 mm for VT-XRD of **Au8**$^+$-BF_4^- and **Au8**$^+$-PF_6^-, 543.5 mm for VT-XRD of **2H12**, **Au12**$^+$-Cl$^-$, **Au12**$^+$-BF_4^-, **Au16**$^+$-BF_4^-, **Au12**$^+$-PF_6^-, **Au16**$^+$-PF_6^-, **Au12**$^+$-PCCp$^-$, and **Au16**$^+$-PCCp$^-$, 546.0 mm for VT-XRD of **2H16** and **Au16**$^+$-Cl$^-$, 541.8 mm for VT-XRD of **2H20**, **Au20**$^+$-Cl$^-$, **Au20**$^+$-BF_4^-, **Au20**$^+$-PF_6^-, and **Au20**$^+$-PCCp$^-$, 292.6 mm for the wide-angle XRD of **Au12**$^+$-Cl$^-$, **Au16**$^+$-Cl$^-$, **Au20**$^+$-Cl$^-$, **Au12**$^+$-PCCp$^-$, **Au16**$^+$-PCCp$^-$, and **Au20**$^+$-PCCp$^-$, and 541.8 mm for the XRD for the sheared samples of **Au16**$^+$-PCCp$^-$ and **Au20**$^+$-PCCp using an imaging plate as a detector. The diffraction patterns were obtained with a 0.01° step in 2θ under 10 sec exposure time of X-ray beam. The samples were sealed in a quartz capillary for VT-XRD and inserted between two Kapton (polyimide) films for shearing. The initial precipitate samples were prepared by reprecipitation from the CH_2Cl_2/MeOH solution, otherwise indicated.

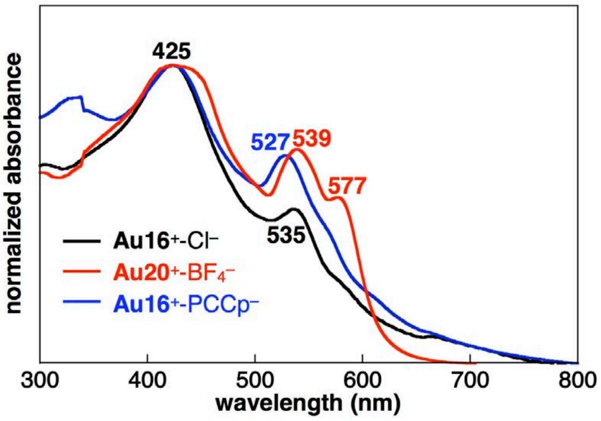

Figure S63 UV/vis absorption spectra of Au16$^+$-Cl$^-$, Au20$^+$-BF_4^-, and Au16$^+$-PCCp$^-$, Related to Figure 7. UV/vis absorption spectra of **Au16**$^+$-Cl$^-$ (black), **Au20**$^+$-BF_4^- (red), and **Au16**$^+$-PCCp$^-$ (blue) in the solid state at r.t. cooling from isotropic liquids (Iso). Ion pairs **Au16**$^+$-X$^-$ (X$^-$ = Cl$^-$ and PCCp$^-$) and **Au20**$^+$-BF_4^- showed the λ_{max} at 425 nm with characteristic small bands: the Cl$^-$ and BF_4^- ion pairs showed similar peaks at 535/576 nm (shoulder) and 539/577 nm, respectively, whereas **Au16**$^+$-PCCp$^-$ showed a blue-shifted shoulder peak of 527 nm. The shifted shoulder peaks were derived from the different stacking modes as ion-pairing assemblies. The large peak of **Au20**$^+$-BF_4^- at 577 nm can be the result of the possible slipped stacking of porphyrin core unit due to the less ordered arrangement. In contrast, the blue-shifted shoulder of **Au16**$^+$-PCCp$^-$ is more similar to the Q band of the solution-state monomeric ion pairs as observed at 521 nm. The blue-shifted absorption compared to the Cl$^-$ and BF_4^- ion pairs can be explained by the distinct alternate stacking of **Au16**$^+$ and PCCp$^-$, resulting in the monomer-state UV/vis absorption. These results showed that the ion pairs with Cl$^-$ and BF_4^- probably form preferentially charge-segregated assemblies, whereas planar PCCp$^-$ forms a charge-by-charge assembly. **Au16**$^+$-PF_6^- is not shown due to the isotropic state at r.t. cooling from Iso.

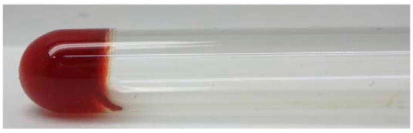

Figure S64 Photograph of gel, Related to Figure 6.
Photograph of gel prepared from an octane solution of **Au16**$^{+}$-PCCp^{-} (10 mg/mL). The gel of **Au16**$^{+}$-PCCp^{-} was transformed to the solution state at 31 °C. Other ion pairs such as **Au16**$^{+}$-Cl^{-}, **Au16**$^{+}$-BF_4^{-}, and **Au16**$^{+}$-PF_6^{-} did not show gelation behaviors.

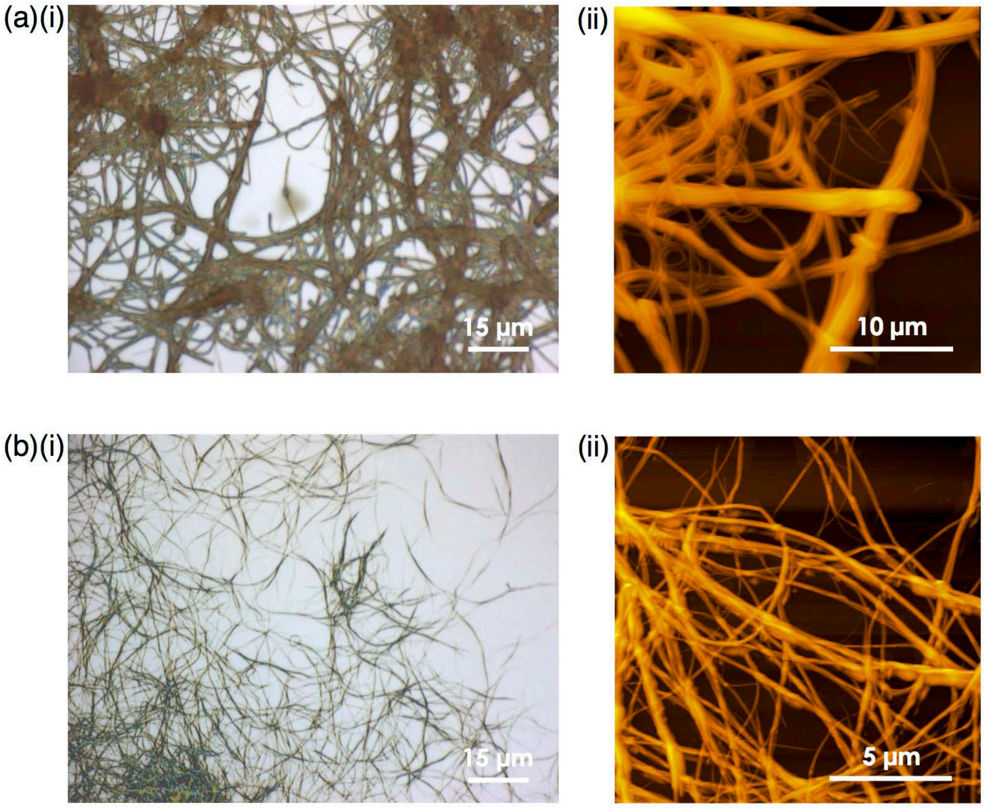

Figure S65 Photographs of optical microscopy and AFM images, Related to Figure 6.
Photographs of (i) optical microscopy (OM) and (ii) AFM images of (a) precipitate of **Au16**$^{+}$-BF_4^{-} and (b) gel (xerogel) of **Au16**$^{+}$-PCCp^{-} prepared from octane solutions (10 mg/mL).

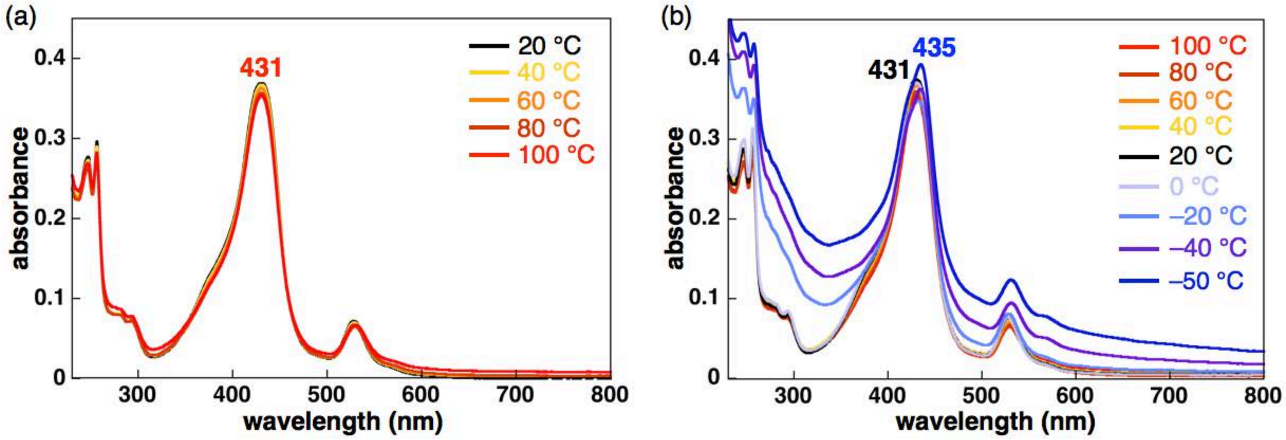

Figure S66 Variable-temperature (VT) UV/vis absorption spectra of Au16^{+}-PCCp^{-}, Related to Figure 6.
Variable-temperature (VT) UV/vis absorption spectra of **Au16**$^{+}$-PCCp^{-} in octane (4×10^{-6} M) from (a) 20 to 100 °C and (b) 100 to –50 °C. Broader peak maxima were observed than those in CH_2Cl_2 solutions due to the formation of tightly bound ion pairs and resulting aggregations.

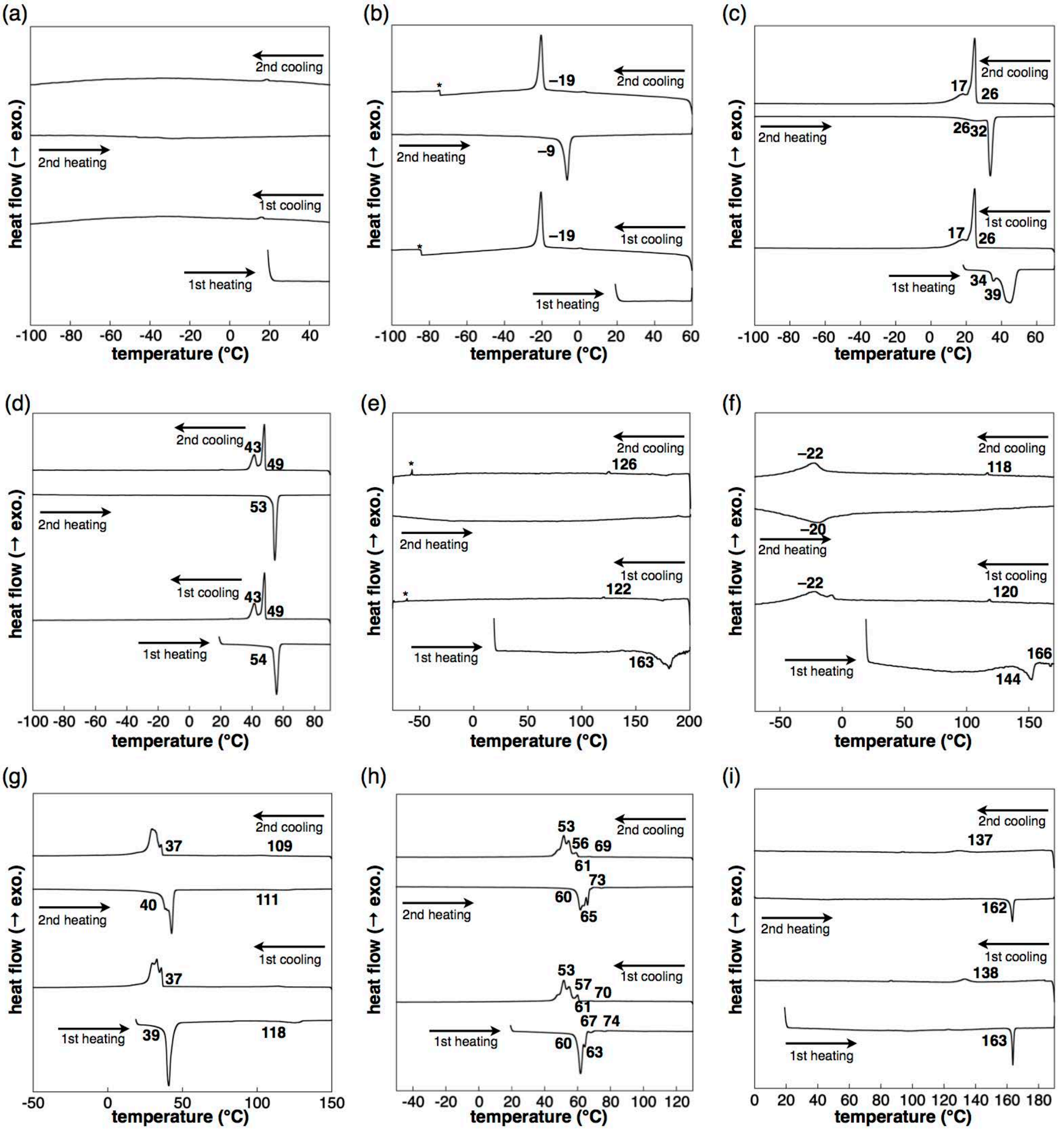

Figure S67 DSC thermographs of ion pairs, Related to Table 1.

DSC thermographs of (a) **2H8**, (b) **2H12**, (c) **2H16**, (d) **2H20**, (e) **Au8**$^{+}$-Cl^{-}, (f) **Au12**$^{+}$-Cl^{-}, (g) **Au16**$^{+}$-Cl^{-}, (h) **Au20**$^{+}$-Cl^{-}, and (i) **Au8**$^{+}$-BF_4^{-} at a scanning rate of 5 °C/min. Onset temperatures for transitions are described except for several transitions in (c) due to the board peaks. **2H8** as a liquid-like state was obtained by the evaporation from a CH_2Cl_2 solution. In the cooling processes of (b), unidentified peaks derived from machine operation (marked by asterisks) were observed.

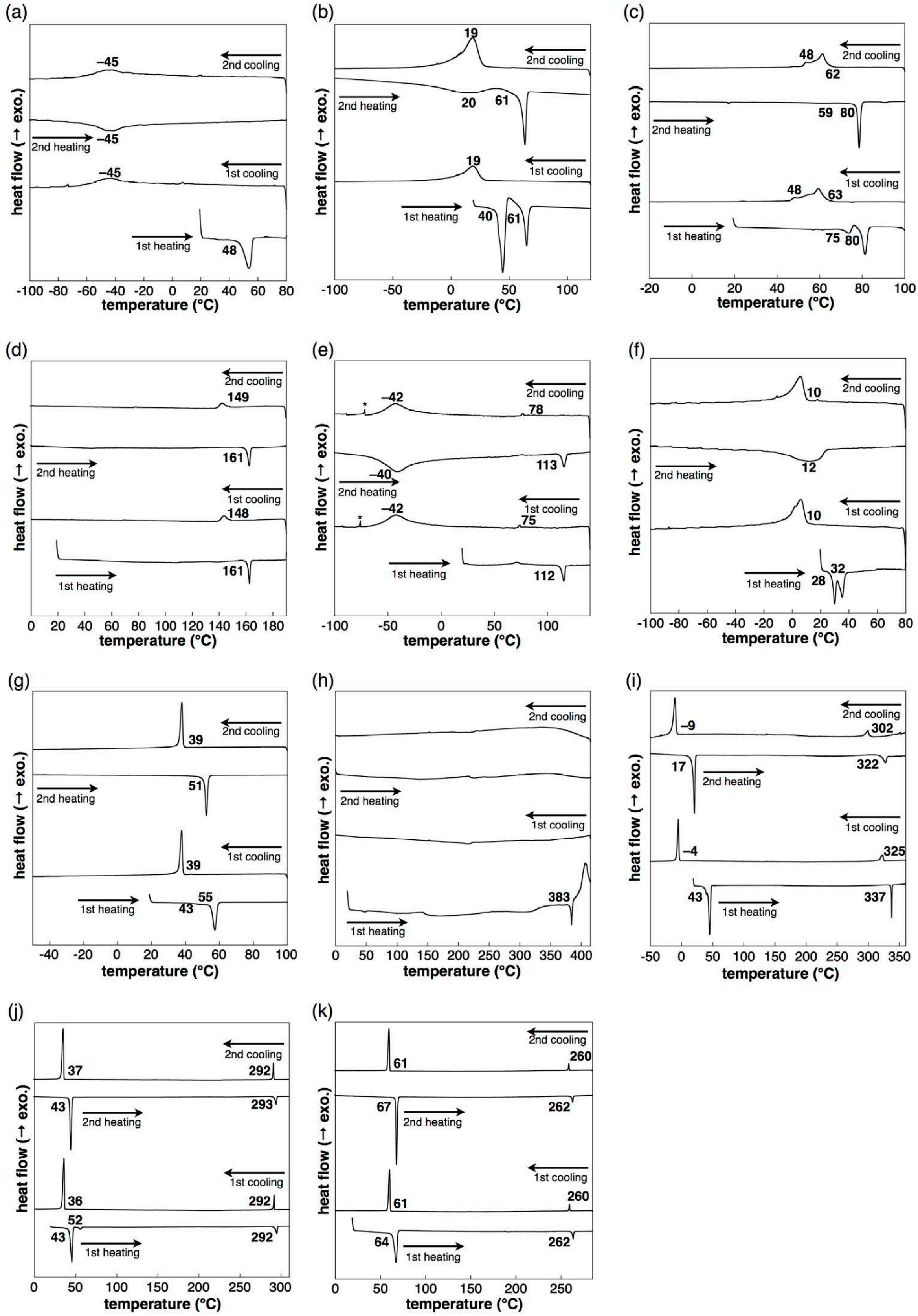

Figure S68 DSC thermographs of ion pairs, Related to Table 1.

DSC thermographs of (a) **Au12**$^+$-BF_4^-, (b) **Au16**$^+$-BF_4^-, (c) **Au20**$^+$-BF_4^-, (d) **Au8**$^+$-PF_6^-, (e) **Au12**$^+$-PF_6^-, (f) **Au16**$^+$-PF_6^-, (g) **Au20**$^+$-PF_6^-, (h) **Au8**$^+$-PCCp$^-$, (i) **Au12**$^+$-PCCp$^-$, (j) **Au16**$^+$-PCCp$^-$, and (k) **Au20**$^+$-PCCp$^-$ at a scanning rate of 5 °C/min. Onset temperatures for transitions are described except for several transitions in (a–c,e,f) due to the board peaks. In the cooling processes of (e), unidentified peaks derived from machine operation (marked by asterisks) were observed.

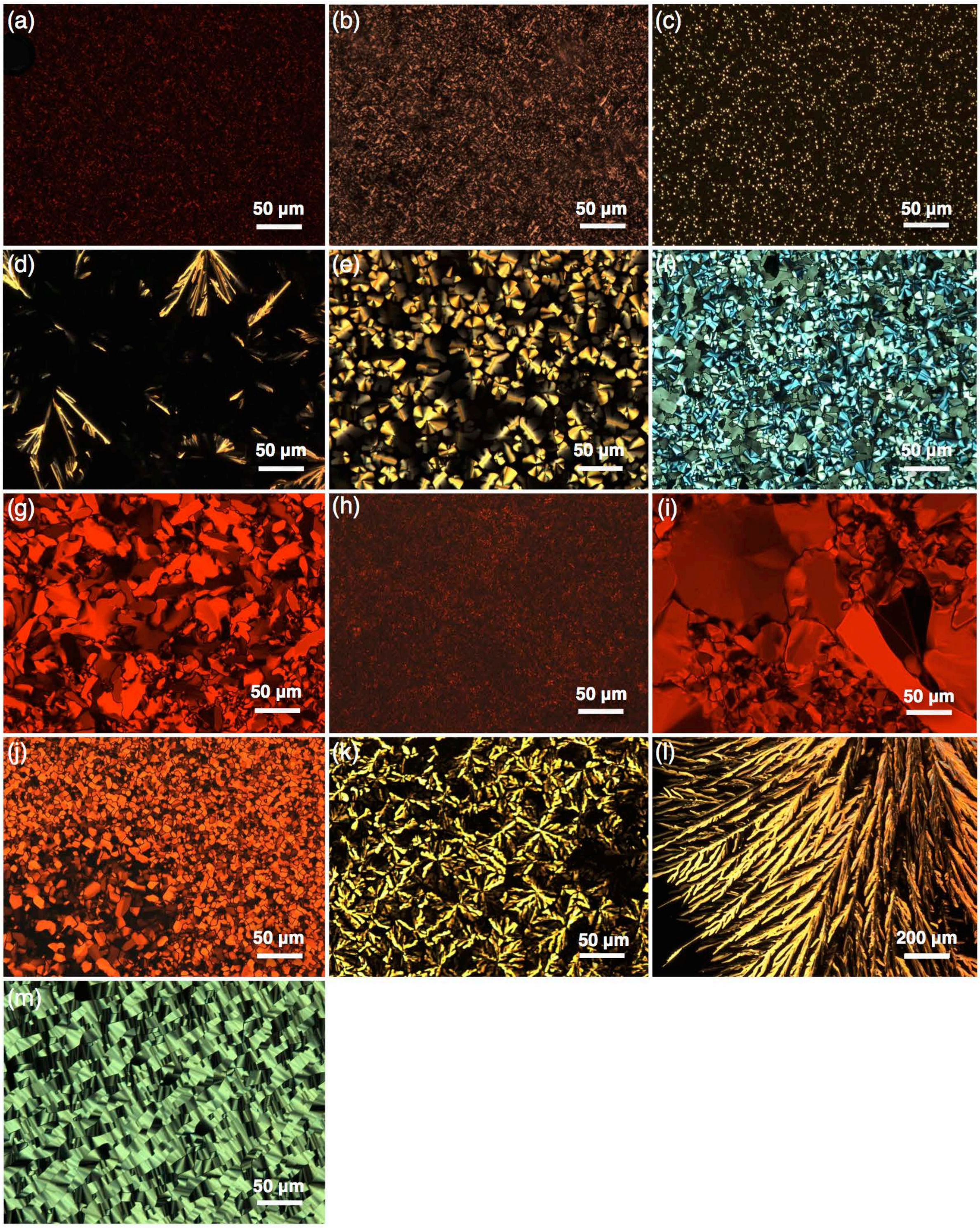

Figure S69 POM images of ion pairs, Related to Figure 7.
POM images of (a) **2H16** at 25 °C, (b) **2H20** at 35 °C, (c) **Au8**$^+$-Cl$^-$ at 80 °C, (d) **Au12**$^+$-Cl$^-$ at 50 °C, (e) **Au16**$^+$-Cl$^-$ at 100 °C, (f) **Au20**$^+$-Cl$^-$ at 50 °C, (g) **Au8**$^+$-BF$_4^-$ at 110 °C, (h) **Au20**$^+$-BF$_4^-$ at 35 °C, (i) **Au8**$^+$-PF$_6^-$ at 120 °C, (j) **Au12**$^+$-PF$_6^-$ at 105 °C, (k) **Au12**$^+$-PCCp$^-$ at 100 °C, (l) **Au16**$^+$-PCCp$^-$ at 280 °C, and (m) **Au20**$^+$-PCCp$^-$ at 250 °C obtained by cooling from isotropic liquids. The textures of (a)–(f), (h), and (j)–(l) were observed as mesophases, whereas those of (g) and (i) were observed as crystal states. **Au16**$^+$-BF$_4^-$ and **Au20**$^+$-PF$_6^-$ did not show POM textures between r.t. and isotropic state due to less ordered assembling states as seen in the XRD data of the crystalline phases (Figure S95,106). POM observations of **2H8**, **2H12**, **Au12**$^+$-BF$_4^-$, and **Au16**$^+$-PF$_6^-$ were not examined due to their isotropic state at r.t. and that of **Au8**$^+$-PCCp$^-$ was not examined due to the decomposition in the thermal process at high temperature.

Table S2 Summarized phase transition behaviors of ion-pairing assemblies, Related to Table 1. Crystalline states are shown in italic. The details of the packing structures such as XRD patterns of ion pairs are shown in Figure S71–120 and Table S3–19.

compounds	cooling[a]	heating[a]
**2H8**	Iso[b]	Iso[b]
**2H12**	*lamellar* –19 Iso	*lamellar* –9 Iso
**2H16**	*lamellar* 17[c] lamellar 26 Iso	*lamellar* 26[c] lamellar 32 Iso
**2H20**	*lamellar* 43 lamellar 49 Iso	*lamellar* 53 Iso
**Au8**$^+$-Cl^-	*Col_h* 122 Iso	*Col_h* 144[d] Iso
**Au12**$^+$-Cl^-	*Col_h* –22[c] Col_h 120 Iso	*Col_h* –20[c] Col_h 138[d] Iso
**Au16**$^+$-Cl^-	*Col_h* 37 Col_h 109[c] Iso	*Col_h* 40 Col_h 111 Iso
**Au20**$^+$-Cl^-	*Col_h* 53 Col_h 57 Col_h 61 Col_h 70 Iso	*Col_h* 60 Col_h 65 Col_h 73 Iso
**Au8**$^+$-BF_4^-	*Cr* 138 Iso	*Cr* 162 Iso
**Au12**$^+$-BF_4^-	amorphous –45[c] Iso	amorphous –45[c] Iso
**Au16**$^+$-BF_4^-	*Col_h* 19[c,f] Iso	*Col_h* 20[c] Col_h 61 Iso
**Au20**$^+$-BF_4^-	*Col_r* 48[c] Col_h 63 Iso	*Col_r* 59[c] Col_h 80 Iso
**Au8**$^+$-PF_6^-	*Cr* 148 Iso	*Cr* 161 Iso
**Au12**$^+$-PF_6^-	*Col_{ob}* –42[c] Col_{ob} 75 Col_{ob} 105[d] Iso	*Col_{ob}* –40[c] Col_{ob} 113 Iso
**Au16**$^+$-PF_6^-	*lamellar* 10 Iso	*lamellar* 12[c] Iso
**Au20**$^+$-PF_6^-	*lamellar* 39 Iso	*lamellar* 51 Iso
**Au8**$^+$-PCCp$^-$	–[g]	–[g]
**Au12**$^+$-PCCp$^-$	–[h]	–[h]
**Au16**$^+$-PCCp$^-$	*Col_h* 36 Col_h 292 Iso	*Col_h* 43 Col_h 293 Iso
**Au20**$^+$-PCCp$^-$	*Col_r* 61 Col_h 260 Iso	*Col_r* 67 Col_h 262 Iso

[a] Transition temperatures (°C, the onset of the peak) from DSC 1st cooling and 2nd heating scans (5 °C min^{-1}). [b] Evaluated from –100 °C to 50 °C. [c] Peak top temperatures due to the broad DSC peaks. [d] Transition temperatures from POM. [e] Transition temperatures from 2nd cooling. [f] Although there may be a transition at ~0 °C, the detailed examination on the possible mesophase was difficult. [g] Decomposed at 383 °C. [h] Slightly transformed to other species after the transition to Iso at >300 °C.

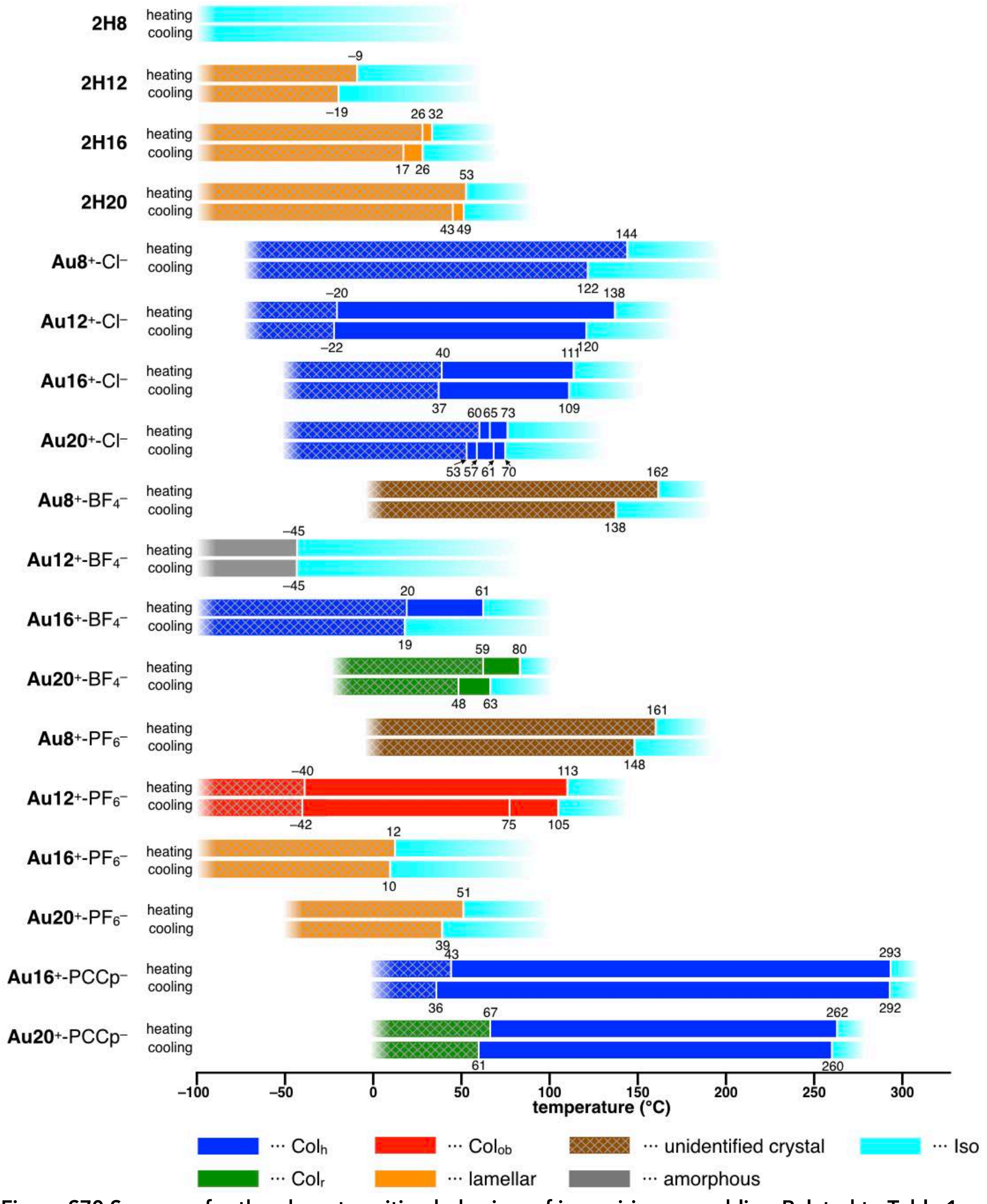

Figure S70 Summary for the phase transition behaviors of ion-pairing assemblies, Related to Table 1. Summary for the phase transition behaviors of ion-pairing assemblies for heating and cooling processes based on DSC and POM observations (Figure S67–69, Table S2) along with XRD (Figure S71–120). The lower and upper limit temperatures for each ion pair are derived from the ranges of DSC measurements. Shaded bars show the crystalline phases. **Au8**$^{+}$-PCCp^{-} and **Au12**$^{+}$-PCCp^{-} are not shown due to the decomposition and the slightly transformed behavior, respectively, in the thermal processes at high temperature (>300 °C).

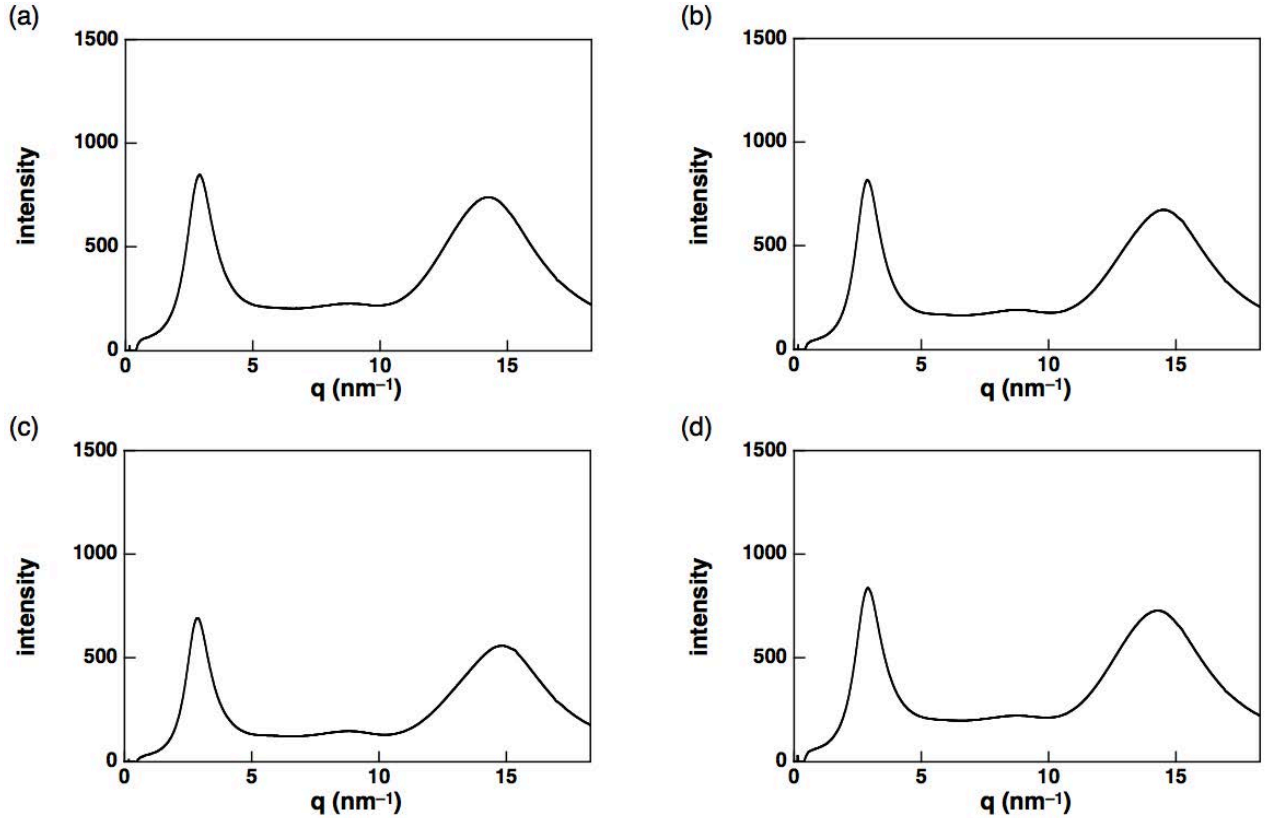

Figure S71 XRD patterns of 2H8, Related to Table 1.
XRD patterns of **2H8** at (a) 25 °C (1st heating), (b) –10 °C (1st cooling), (c) –50 °C (1st cooling), and (d) 25 °C (2nd heating). The initial liquid-like sample was obtained by evaporation from the CH_2Cl_2 solution.

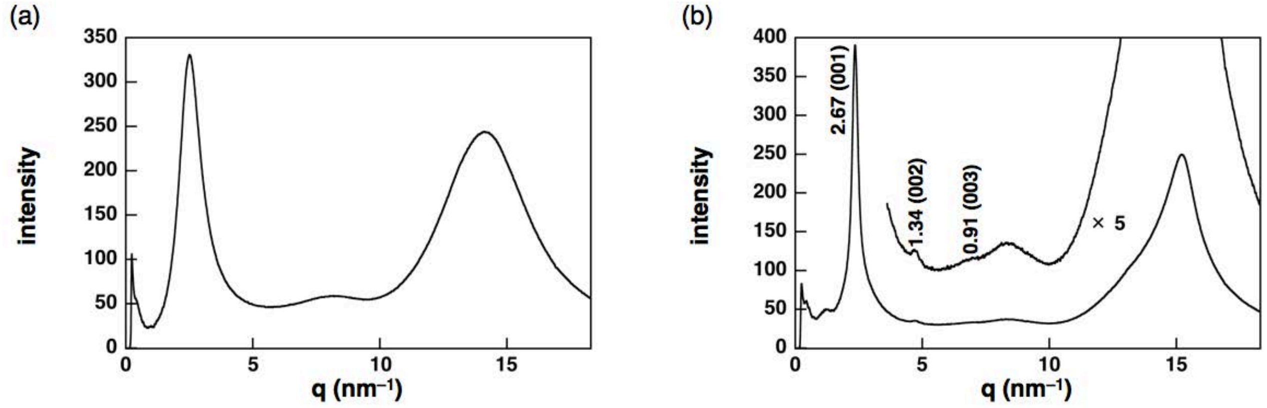

Figure S72 XRD patterns of 2H12, Related to Table 1.
XRD patterns of **2H12** at (a) 25 °C (1st heating) and (b) –35 °C (1st cooling). The XRD pattern of (b) exhibits a lamellar structure (Figure S73).

Table S3 XRD peaks of 2H12, Related to Table 1.
XRD peaks of **2H12** at (b) –35 °C (1st cooling) (Figure S72). The peaks which can be indexed are represented.

	q (nm^{-1})	*d*-spacing (nm)	ratio	ratio (calc.)	*hkl*
(b) **2H12**	2.36	2.67	1.00	1.000	001
–35 °C (1st cooling)	4.69	1.34	0.50	0.500	002
lamellar	6.89	0.91	0.34	0.333	003

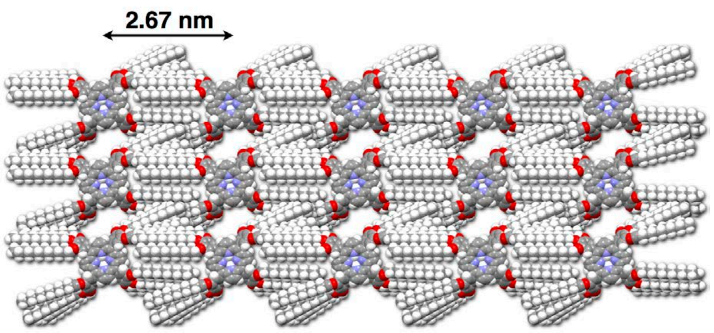

Figure S73 Possible packing model of 2H12, Related to Table 1.
Possible packing model of **2H12** in a lamellar structure.

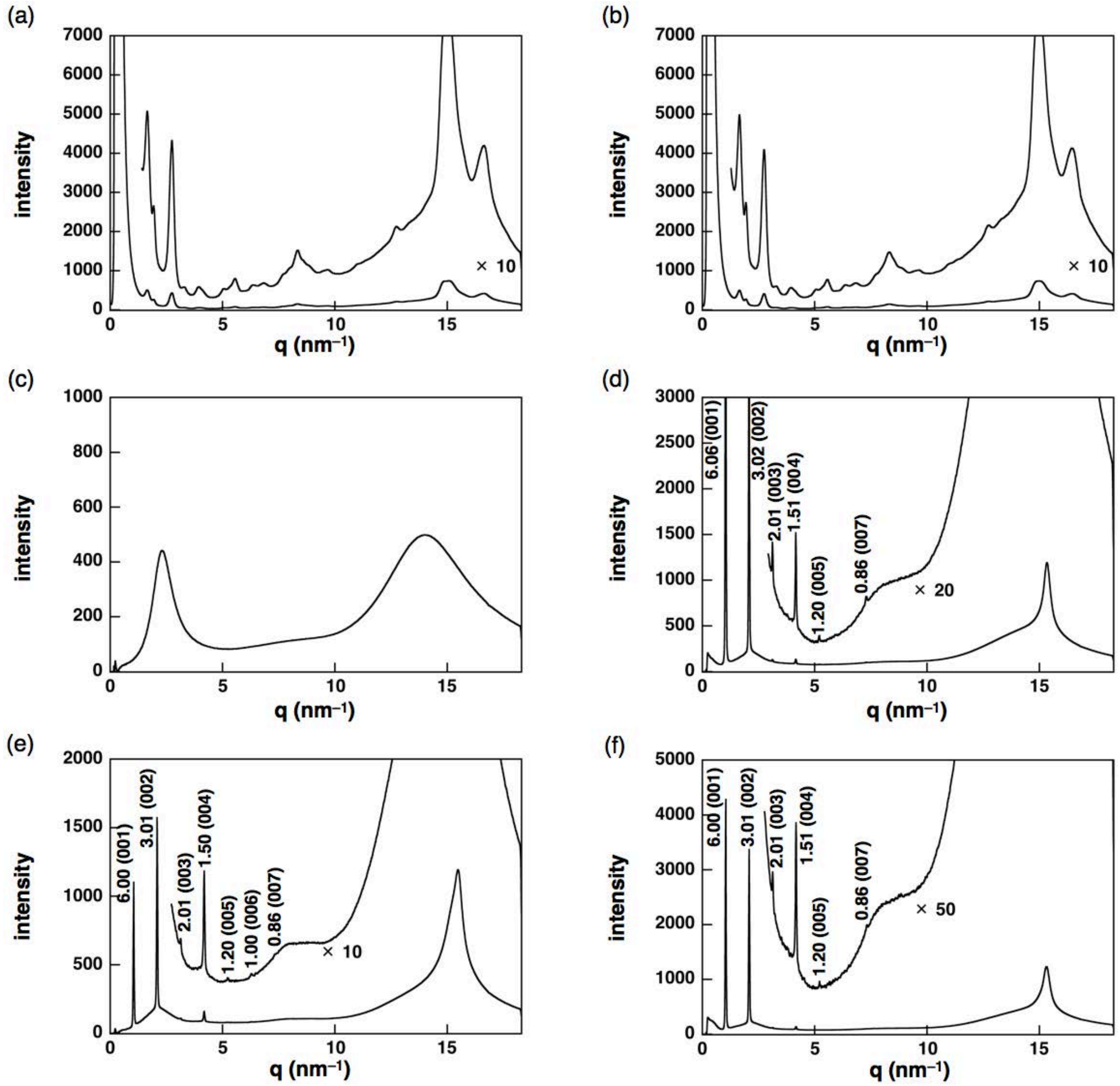

Figure S74 XRD patterns of 2H16, Related to Table 1 and Figure 8.
XRD patterns of **2H16** at (a) 25 °C (1st heating), (b) 36 °C (1st heating), (c) 65 °C (1st heating), (d) 22 °C (1st cooling), (e) 0 °C (1st cooling), and (f) 27 °C (2nd heating). The XRD patterns of (d–f) exhibit lamellar structures (Figure S75).

Table S4 XRD peaks of 2H16, Related to Table 1 and Figure 8.

XRD peaks of **2H16** at (d) 22 °C (1st cooling), (e) 0 °C (1st cooling), and (f) 27 °C (2nd heating) (Figure S74). The peaks which can be indexed are represented.

	q (nm^{-1})	*d*-spacing (nm)	ratio	ratio (calc.)	*hkl*
(d) **2H16** 22 °C (1st cooling) lamellar	1.04	6.06	1.00	1.000	001
	2.08	3.02	0.50	0.500	002
	3.12	2.01	0.33	0.333	003
	4.16	1.51	0.25	0.250	004
	5.22	1.20	0.20	0.200	005
	7.30	0.86	0.14	0.143	007
(e) **2H16** 0 °C (1st cooling) lamellar	1.05	6.00	1.00	1.000	001
	2.09	3.01	0.50	0.500	002
	3.13	2.01	0.33	0.333	003
	4.18	1.50	0.25	0.250	004
	5.23	1.20	0.20	0.200	005
	6.28	1.00	0.17	0.167	006
	7.33	0.86	0.14	0.143	007
(f) **2H16** 27 °C (2nd heating) lamellar	1.05	6.00	1.00	1.000	001
	2.09	3.01	0.50	0.500	002
	3.13	2.01	0.33	0.333	003
	4.17	1.51	0.25	0.250	004
	5.22	1.20	0.20	0.200	005
	7.33	0.86	0.14	0.143	007

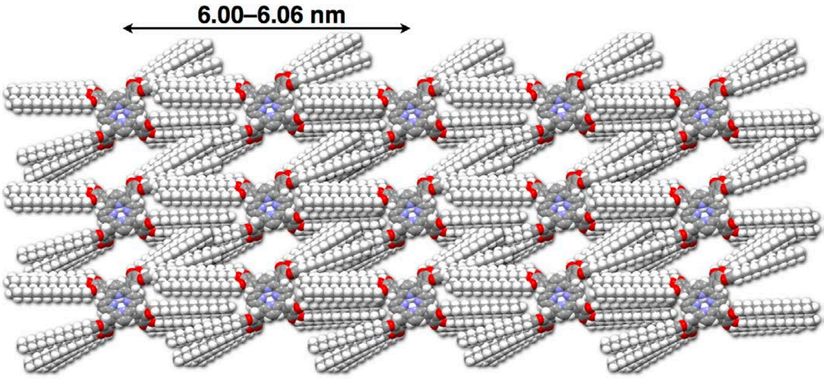

Figure S75 Possible packing model of 2H16, Related to Table 1 and Figure 8.

Possible packing model of **2H16** in a lamellar structure.

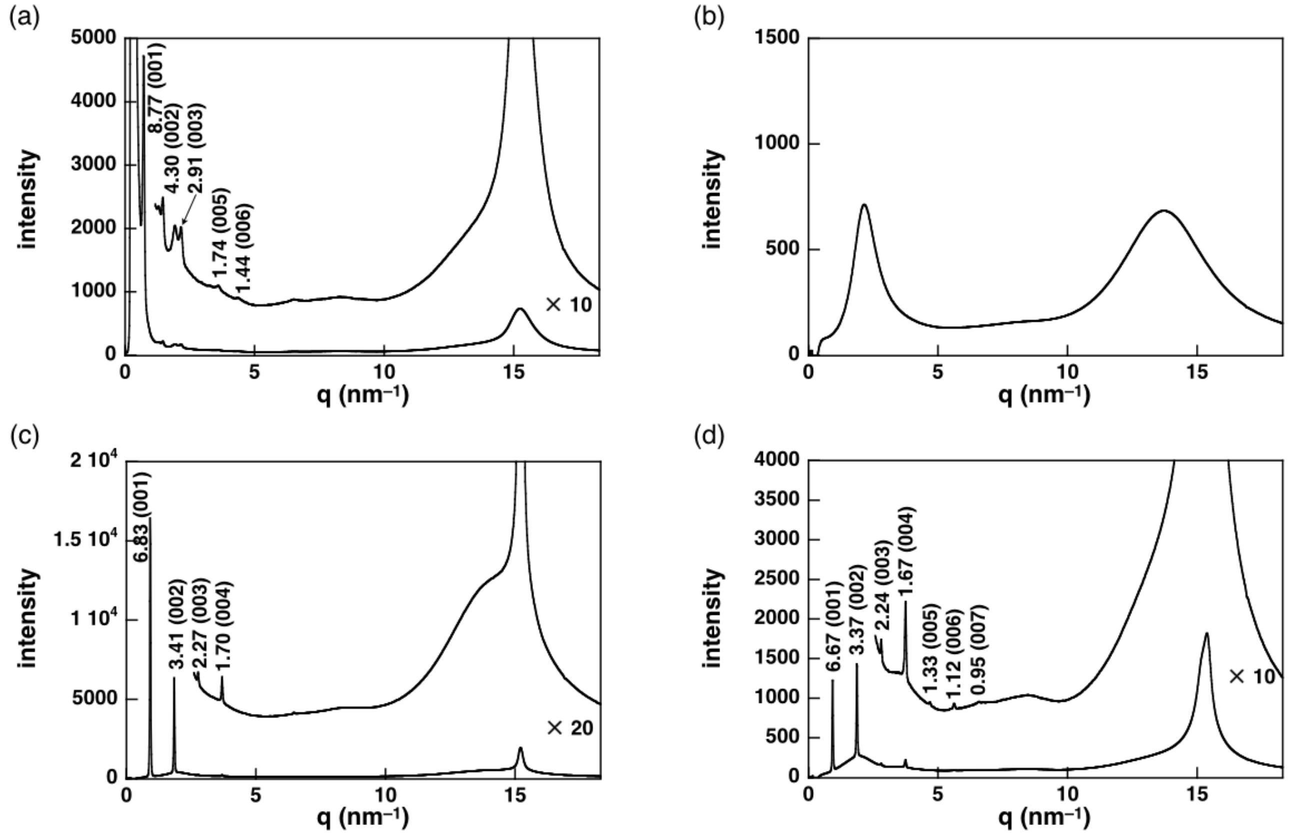

Figure S76 XRD patterns of 2H20, Related to Table 1.
XRD patterns of **2H20** at (a) 25 °C (1st heating), (b) 80 °C (1st heating), (c) 45 °C (1st cooling), and (d) 25 °C (1st cooling). The XRD patterns of (a,c,d) exhibit lamellar structures (Figure S77).

Table S5 XRD peaks of 2H20, Related to Table 1.
XRD peaks of **2H20** at (a) 25 °C (1st heating), (c) 45 °C (1st cooling), and (d) 25 °C (1st cooling) (Figure S76). The peaks which can be indexed are represented.

	q (nm^{-1})	*d*-spacing (nm)	ratio	ratio (calc.)	*hkl*
	0.72	8.77	1.00	1.000	001
(a) **2H20**	1.46	4.30	0.49	0.500	002
25 °C (1st heating)	2.16	2.91	0.33	0.333	003
lamellar	3.60	1.74	0.20	0.250	005
	4.36	1.44	0.16	0.167	006
	0.92	6.83	1.00	1.000	001
(c) **2H20**	1.84	3.41	0.50	0.500	002
45 °C (1st cooling)	2.77	2.27	0.33	0.333	003
lamellar	3.69	1.70	0.25	0.250	004
	0.94	6.67	1.00	1.000	001
	1.87	3.37	0.50	0.500	002
(d) **2H20**	2.80	2.24	0.34	0.333	003
25 °C (1st cooling)	3.75	1.67	0.25	0.250	004
lamellar	4.71	1.33	0.20	0.200	005
	5.62	1.12	0.17	0.167	006
	6.58	0.95	0.14	0.143	007

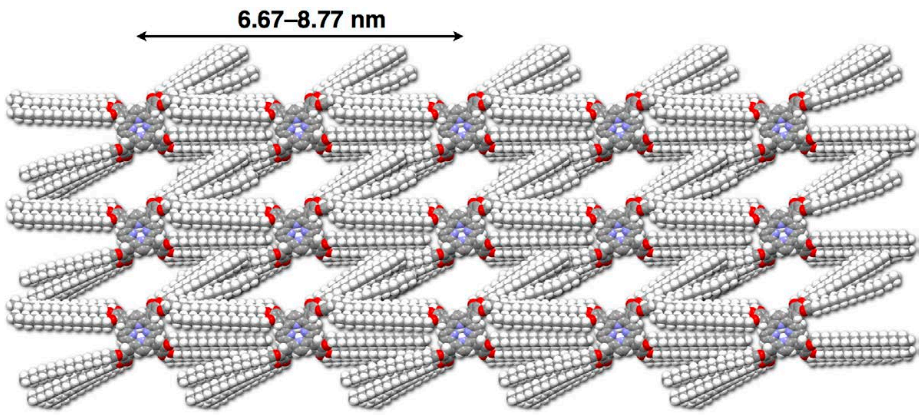

Figure S77 Possible packing model of 2H20, Related to Table 1.
Possible packing model of **2H20** in a lamellar structure.

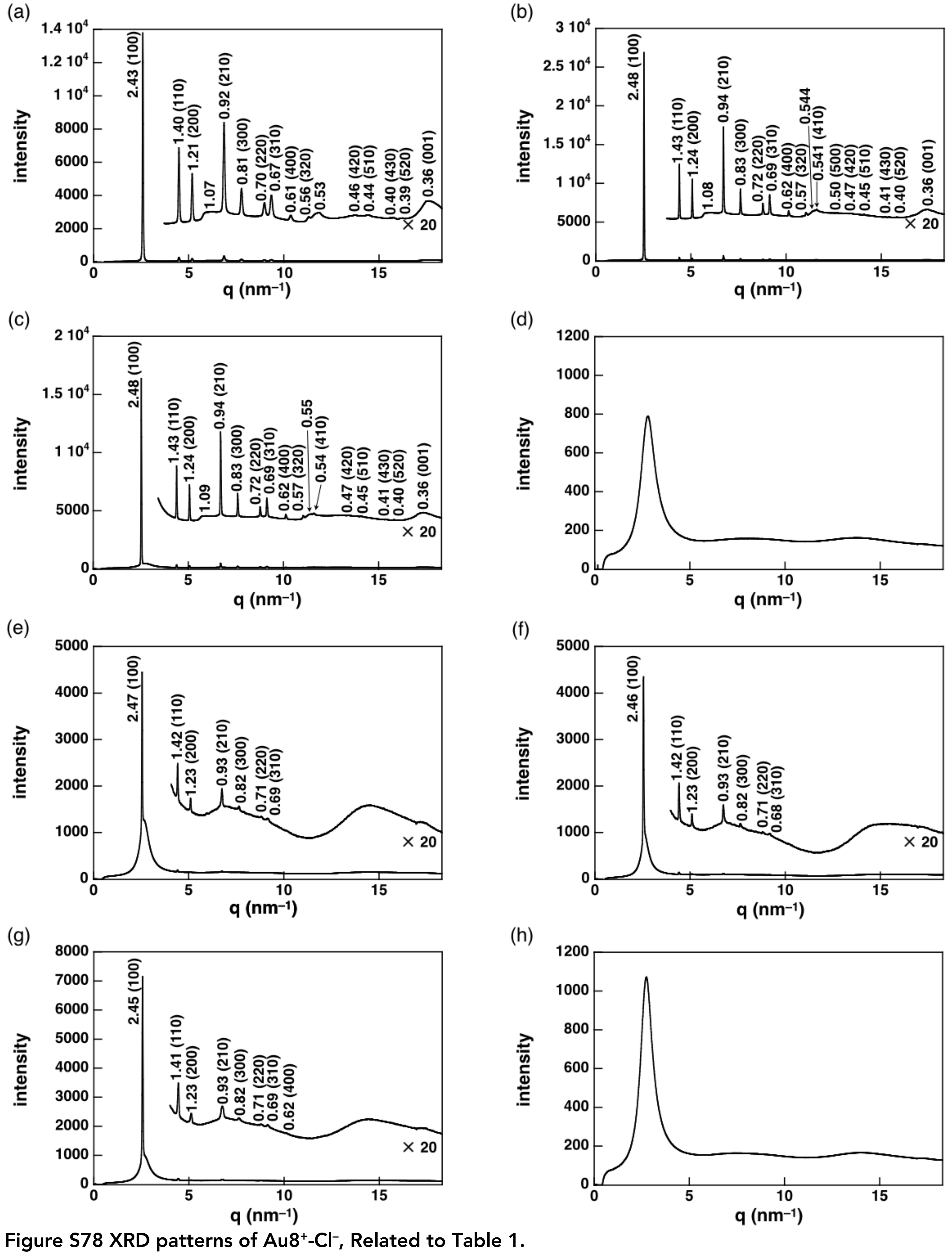

Figure S78 XRD patterns of Au8⁺-Cl⁻, Related to Table 1.

XRD patterns of **Au8**⁺-Cl⁻ at (a) 25 °C (1st heating), (b) 150 °C (1st heating), (c) 166 °C (1st heating), (d) 190 °C (1st heating), (e) 80 °C (1st cooling), (f) 25 °C (1st cooling), (g) 80 °C (2nd heating), and (h) 150 °C (2nd heating). The XRD patterns of (a–c,e–g) exhibit Col_h structures (Figure S79).

Table S6 XRD peaks of Au8$^+$-Cl$^-$, Related to Table 1.

XRD peaks of **Au8**$^+$-Cl$^-$ at (a) 25 °C (1st heating), (b) 150 °C (1st heating), (c) 166 °C (1st heating), (e) 80 °C (1st cooling), (f) 25 °C (1st cooling), and (g) 80 °C (2nd heating) (Figure S78). The peaks which can be indexed are represented.

	q (nm^{-1})	*d*-spacing (nm)	ratio	ratio (calc.)	*hkl*
(a) **Au8**$^+$-Cl$^-$<br>25 °C (1st heating)<br>Col$_h$<br>a = 2.80 nm, c = 0.36 nm<br>M = 2383.73, Z = 1 for ρ = 1.64	2.59	2.43	1.00	1.000	100
	4.49	1.40	0.58	0.577	110
	5.19	1.21	0.50	0.500	200
	6.86	0.92	0.38	0.378	210
	7.77	0.81	0.33	0.333	300
	8.98	0.70	0.29	0.289	220
	9.34	0.67	0.28	0.277	310
	10.36	0.61	0.25	0.250	400
	11.32	0.56	0.23	0.229	320
	13.76	0.46	0.19	0.189	420
	14.42	0.44	0.18	0.180	510
	15.76	0.40	0.16	0.164	430
	16.24	0.39	0.16	0.160	520
	17.64	0.36	–	–	001
(b) **Au8**$^+$-Cl$^-$<br>150 °C (1st heating)<br>Col$_h$<br>a = 2.86 nm, c = 0.36 nm<br>M = 2383.73, Z = 1 for ρ = 1.54	2.53	2.48	1.00	1.000	100
	4.40	1.43	0.58	0.577	110
	5.07	1.24	0.50	0.500	200
	6.71	0.94	0.38	0.378	210
	7.60	0.83	0.33	0.333	300
	8.78	0.72	0.29	0.289	220
	9.14	0.69	0.28	0.277	310
	10.13	0.62	0.25	0.250	400
	11.04	0.57	0.23	0.229	320
	11.61	0.54	0.22	0.218	410
	12.67	0.50	0.20	0.200	500
	13.39	0.47	0.19	0.189	420
	14.09	0.45	0.18	0.180	510
	15.40	0.41	0.17	0.164	430
	15.81	0.40	0.16	0.160	520
	17.45	0.36	–	–	001
(c) **Au8**$^+$-Cl$^-$<br>166 °C (1st heating)<br>Col$_h$<br>a = 2.86 nm, c = 0.36 nm<br>M = 2383.73, Z = 1 for ρ = 1.54	2.53	2.48	1.00	1.000	100
	4.39	1.43	0.58	0.577	110
	5.06	1.24	0.50	0.500	200
	6.70	0.94	0.38	0.378	210
	7.59	0.83	0.33	0.333	300
	8.78	0.72	0.29	0.289	220
	9.13	0.69	0.28	0.277	310
	10.12	0.62	0.25	0.250	400
	11.03	0.57	0.23	0.229	320
	11.60	0.54	0.22	0.218	410
	13.38	0.47	0.19	0.189	420
	14.08	0.45	0.18	0.180	510
	15.38	0.41	0.17	0.164	430
	15.80	0.40	0.16	0.160	520
	17.35	0.36	–	–	001

Table S6 (Continued)

	q (nm⁻¹)	*d*-spacing (nm)	ratio	ratio (calc.)	*hkl*
	2.55	2.47	1.00	1.000	100
(e) **Au8**$^+$-Cl$^-$	4.42	1.42	0.58	0.577	110
80 °C (1st cooling)	5.10	1.23	0.50	0.500	200
Col$_h$[a]	6.74	0.93	0.38	0.378	210
a = 2.85 nm	7.64	0.82	0.33	0.333	300
	8.82	0.71	0.29	0.289	220
	9.17	0.69	0.28	0.277	310
	2.56	2.46	1.00	1.000	100
(f) **Au8**$^+$-Cl$^-$	4.42	1.42	0.58	0.577	110
25 °C (1st cooling)	5.10	1.23	0.50	0.500	200
Col$_h$[a]	6.74	0.93	0.38	0.378	210
a = 2.85 nm	7.64	0.82	0.33	0.333	300
	8.81	0.71	0.29	0.289	220
	9.18	0.68	0.28	0.277	310
	2.57	2.45	1.00	1.000	100
	4.44	1.41	0.58	0.577	110
(g) **Au8**$^+$-Cl$^-$	5.12	1.23	0.50	0.500	200
80 °C (2nd heating)	6.74	0.93	0.38	0.378	210
Col$_h$[a]	7.62	0.82	0.34	0.333	300
a = 2.83 nm	8.80	0.71	0.29	0.289	220
	9.14	0.69	0.28	0.277	310
	10.11	0.62	0.25	0.250	400

[a] *Z* and *ρ* values are not given due to the unclear height value (*c*) in the XRD chart.

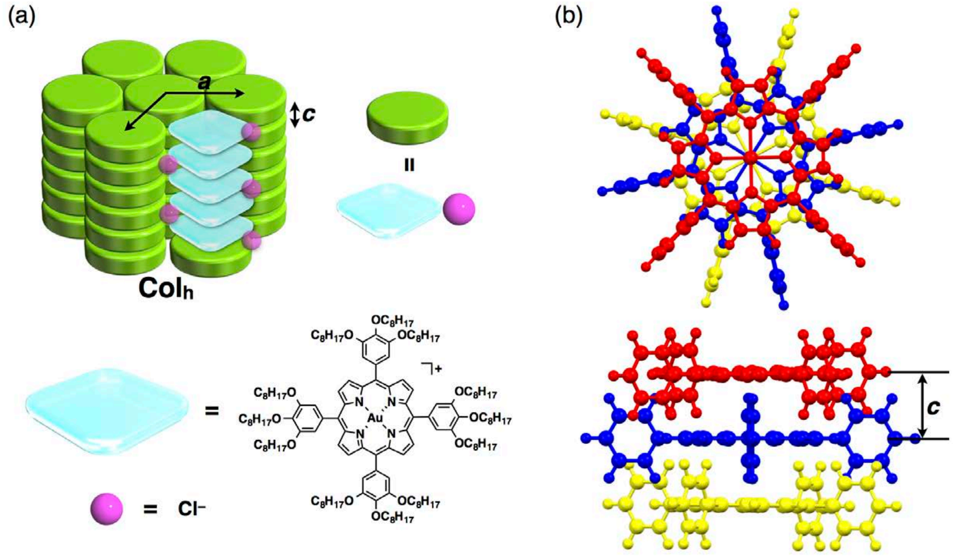

Figure S79 Possible packing model of Au8$^+$-Cl$^-$, Related to Table 1.
(a) Possible packing model of **Au8**$^+$-Cl$^-$ in a Col$_h$ structure and (b) columnar stacking model of the cationic Au^{III} complex (shown by geometry-optimized **Au0**$^+$ instead of **Au8**$^+$). Porphyrin–Au^{III} complexes are stacked with the distance of 0.36 nm (001 peak). Diffraction peak at 0.53–0.54 nm can be ascribable to the arrangement of the peripheral aryl rings or coexisting Cl$^-$. Arrangement of the anions in the model structure of (a) is not exactly determined.

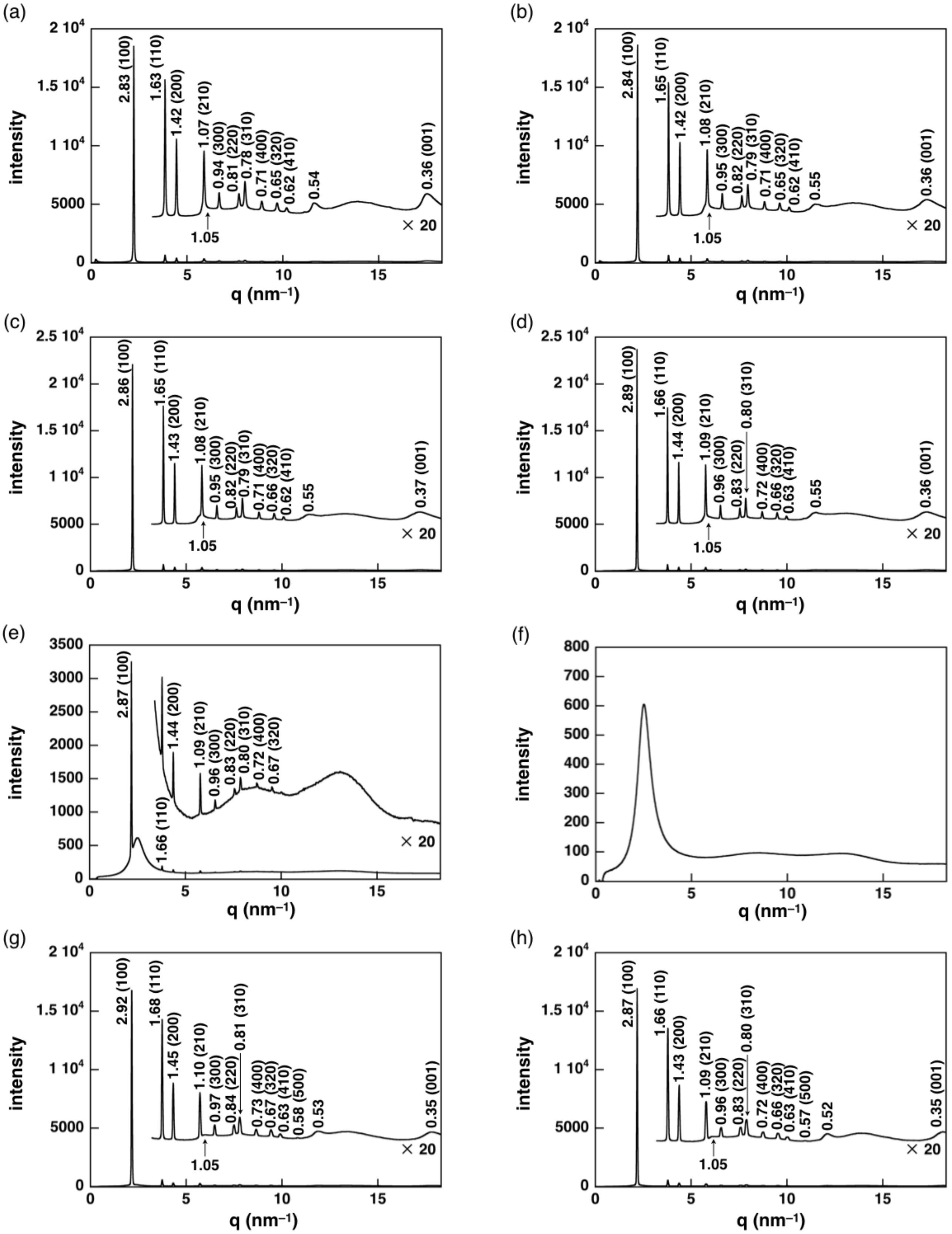

Figure S80 XRD patterns of Au12$^+$-Cl$^-$, Related to Table 1.

XRD patterns of **Au12**$^+$-Cl$^-$ at (a) 25 °C (1st heating), (b) 90 °C (1st heating), (c) 120 °C (1st heating), (d) 150 °C (1st heating), (e) 170 °C (1st heating), (f) 180 °C (1st heating), (g) 100 °C (1st cooling), and (h) 20 °C (1st cooling). The XRD patterns of (a–e,g,h) exhibit Col_h structures (Figure S82). The broad peak around *d* = 1 nm is partially overlapped with other peaks.

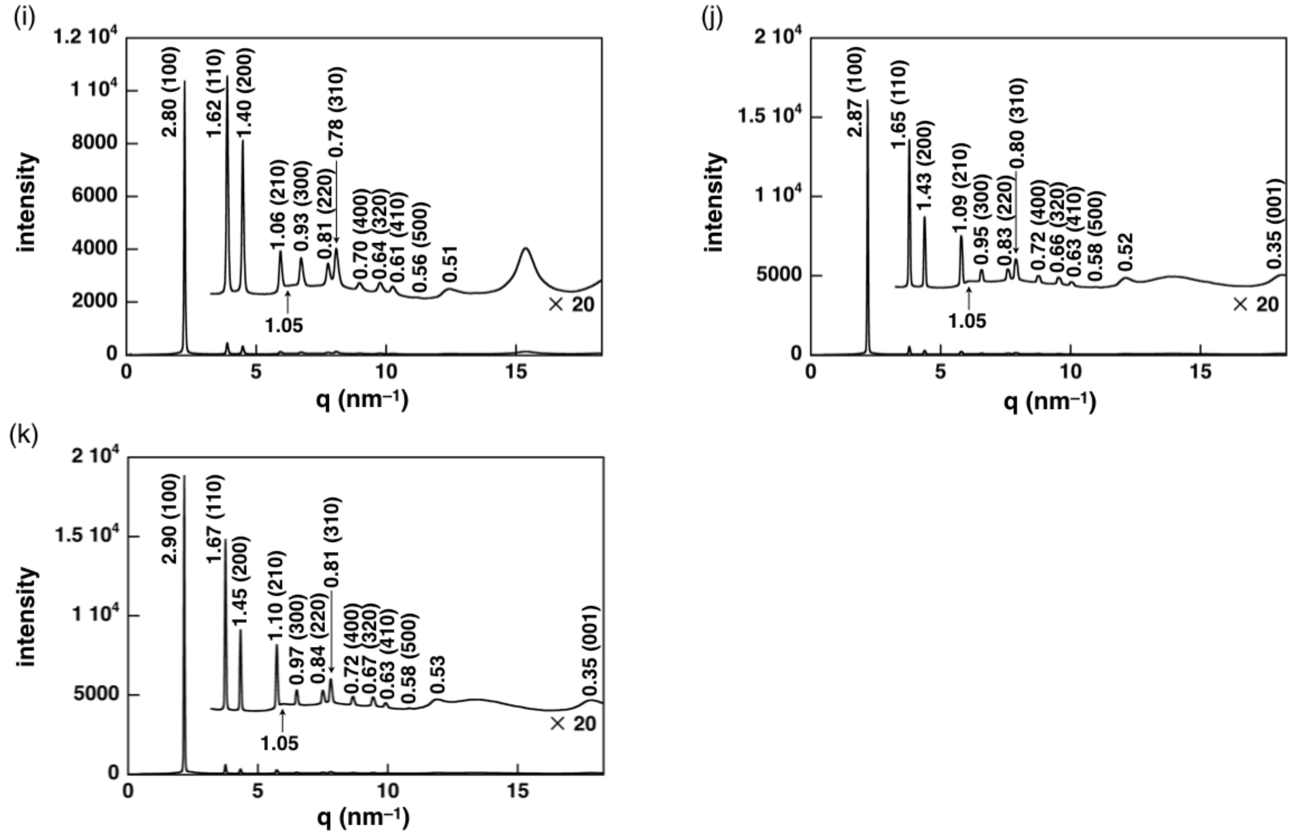

Figure S81 XRD patterns of Au12$^+$-Cl$^-$, Related to Table 1.
XRD patterns of **Au12**$^+$-Cl$^-$ at (i) –60 °C (1st cooling), (j) 20 °C (2nd heating), and (k) 100 °C (2nd heating) (Figure labels are continued from Figure S80). The XRD patterns of (i–k) exhibit Col$_h$ structures (Figure S82). The broad peak around d = 1 nm is partially overlapped with other peaks.

Table S7 XRD peaks of Au12$^+$-Cl$^-$, Related to Table 1.
XRD peaks of **Au12**$^+$-Cl$^-$ at (a) 25 °C (1st heating), (b) 90 °C (1st heating), (c) 120 °C (1st heating), (d) 150 °C (1st heating), (e) 170 °C (1st heating), (g) 100 °C (1st cooling), (h) 20 °C (1st cooling), (i) –60 °C (1st cooling), (j) 20 °C (2nd heating), and (k) 100 °C (2nd heating) (Figure S80,81). The peaks which can be indexed are represented.

	q (nm^{-1})	*d*-spacing (nm)	ratio	ratio (calc.)	*hkl*
	2.22	2.83	1.00	1.000	100
	3.85	1.63	0.58	0.577	110
	4.44	1.42	0.50	0.500	200
(a) **Au12**$^+$-Cl$^-$	5.89	1.07	0.38	0.378	210
25 °C (1st heating)	6.68	0.94	0.33	0.333	300
Col$_h$	7.71	0.81	0.29	0.289	220
a = 3.27 nm, c = 0.36 nm	8.03	0.78	0.28	0.277	310
M = 3057.03, Z = 1 for ρ = 1.54	8.90	0.71	0.25	0.250	400
	9.70	0.65	0.23	0.229	320
	10.20	0.62	0.22	0.218	410
	17.52	0.36	–	–	001

Table S7 (Continued)

	q (nm^{-1})	*d*-spacing (nm)	ratio	ratio (calc.)	*hkl*
(b) **Au12**$^+$-Cl$^-$ 90 °C (1st heating) Col$_h$ a = 3.28 nm, c = 0.36 nm M = 3057.03, Z = 1 for ρ = 1.50	2.21	2.84	1.00	1.000	100
	3.82	1.65	0.58	0.577	110
	4.42	1.42	0.50	0.500	200
	5.84	1.08	0.38	0.378	210
	6.62	0.95	0.33	0.333	300
	7.64	0.82	0.29	0.289	220
	7.96	0.79	0.28	0.277	310
	8.83	0.71	0.25	0.250	400
	9.62	0.65	0.23	0.229	320
	10.11	0.62	0.22	0.218	410
	17.30	0.36	–	–	001
(c) **Au12**$^+$-Cl$^-$ 120 °C (1st heating) Col$_h$ a = 3.30 nm, c = 0.37 nm M = 3057.03, Z = 1 for ρ = 1.48	2.20	2.86	1.00	1.000	100
	3.81	1.65	0.58	0.577	110
	4.40	1.43	0.50	0.500	200
	5.82	1.08	0.38	0.378	210
	6.60	0.95	0.33	0.333	300
	7.62	0.82	0.29	0.289	220
	7.93	0.79	0.28	0.277	310
	8.79	0.71	0.25	0.250	400
	9.58	0.66	0.23	0.229	320
	10.07	0.62	0.22	0.218	410
	17.18	0.37	–	–	001
(d) **Au12**$^+$-Cl$^-$ 150 °C (1st heating) Col$_h$ a = 3.30 nm, c = 0.36 nm M = 3057.03, Z = 1 for ρ = 1.45	2.18	2.89	1.00	1.000	100
	3.77	1.66	0.58	0.577	110
	4.36	1.44	0.50	0.500	200
	5.75	1.09	0.38	0.378	210
	6.53	0.96	0.33	0.333	300
	7.54	0.83	0.29	0.289	220
	7.85	0.80	0.28	0.277	310
	8.70	0.72	0.25	0.250	400
	9.49	0.66	0.23	0.229	320
	9.97	0.63	0.22	0.218	410
	17.29	0.36	–	–	001
(e) **Au12**$^+$-Cl$^-$ 170 °C (1st heating) Col$_h$^{a} a = 3.37 nm	2.19	2.87	1.00	1.000	100
	3.79	1.66	0.58	0.577	110
	4.37	1.44	0.50	0.500	200
	5.78	1.09	0.38	0.378	210
	6.55	0.96	0.33	0.333	300
	7.55	0.83	0.29	0.289	220
	7.87	0.80	0.28	0.277	310
	8.72	0.72	0.25	0.250	400
	9.51	0.67	0.23	0.229	320
(g) **Au12**$^+$-Cl$^-$ 100 °C (1st cooling) Col$_h$ a = 3.37 nm, c = 0.35 nm M = 3057.03, Z = 1 for ρ = 1.46	2.15	2.92	1.00	1.000	100
	3.74	1.68	0.58	0.577	110
	4.33	1.45	0.50	0.500	200
	5.72	1.10	0.38	0.378	210
	6.49	0.97	0.33	0.333	300
	7.50	0.84	0.29	0.289	220
	7.79	0.81	0.28	0.277	310
	8.66	0.73	0.25	0.250	400
	9.42	0.67	0.23	0.229	320
	9.91	0.63	0.22	0.218	410
	10.82	0.58	0.20	0.200	500
	17.80	0.35	–	–	001

Table S7 (Continued)

	q (nm^{-1})	*d*-spacing (nm)	ratio	ratio (calc.)	*hkl*
(h) **Au12**$^{+}$-Cl^{-}<br>20 °C (1st cooling)<br>Col$_h$<br>*a* = 3.32 nm, *c* = 0.35 nm<br>*M* = 3057.03, *Z* = 1 for ρ = 1.54	2.19	2.87	1.00	1.000	100
	3.79	1.66	0.58	0.577	110
	4.38	1.43	0.50	0.500	200
	5.79	1.09	0.38	0.378	210
	6.58	0.96	0.33	0.333	300
	7.60	0.83	0.29	0.289	220
	7.90	0.80	0.28	0.277	310
	8.77	0.72	0.25	0.250	400
	9.51	0.66	0.23	0.229	320
	10.00	0.63	0.22	0.218	410
	10.96	0.57	0.20	0.200	500
	18.13	0.35	–	–	001
(i) **Au12**$^{+}$-Cl^{-}<br>–60 °C (1st cooling)<br>Col$_h$[b]<br>*a* = 3.32 nm	2.24	2.80	1.00	1.000	100
	3.89	1.62	0.58	0.577	110
	4.48	1.40	0.50	0.500	200
	5.93	1.06	0.38	0.378	210
	6.72	0.93	0.33	0.333	300
	7.77	0.81	0.29	0.289	220
	8.08	0.78	0.28	0.277	310
	8.96	0.70	0.25	0.250	400
	9.77	0.64	0.23	0.229	320
	10.25	0.61	0.22	0.218	410
	11.13	0.56	0.20	0.200	500
(j) **Au12**$^{+}$-Cl^{-}<br>20 °C (2nd heating)<br>Col$_h$<br>*a* = 3.32 nm, *c* = 0.35 nm<br>*M* = 3057.03, *Z* = 1 for ρ = 1.54	2.19	2.87	1.00	1.000	100
	3.80	1.65	0.58	0.577	110
	4.40	1.43	0.50	0.500	200
	5.79	1.09	0.38	0.378	210
	6.59	0.95	0.33	0.333	300
	7.58	0.83	0.29	0.289	220
	7.89	0.80	0.28	0.277	310
	8.75	0.72	0.25	0.250	400
	9.53	0.66	0.23	0.229	320
	10.02	0.63	0.22	0.218	410
	10.91	0.58	0.20	0.200	500
	18.14	0.35	–	–	001
(k) **Au12**$^{+}$-Cl^{-}<br>100 °C (2nd heating)<br>Col$_h$<br>*a* = 3.35 nm, *c* = 0.35 nm<br>*M* = 3057.03, Z = 1 for ρ = 1.48	2.17	2.90	1.00	1.000	100
	3.75	1.67	0.58	0.577	110
	4.34	1.45	0.50	0.500	200
	5.73	1.10	0.38	0.378	210
	6.50	0.97	0.33	0.333	300
	7.50	0.84	0.29	0.289	220
	7.80	0.81	0.28	0.277	310
	8.67	0.72	0.25	0.250	400
	9.43	0.67	0.23	0.229	320
	9.92	0.63	0.22	0.218	410
	10.83	0.58	0.20	0.200	500
	17.80	0.35	–	–	001

[a] *Z* and ρ values are not given due to the unclear height value (*c*) in the XRD chart. [b] The diffraction peak which corresponds to 001 was observed at the wide-angle region (Figure S83).

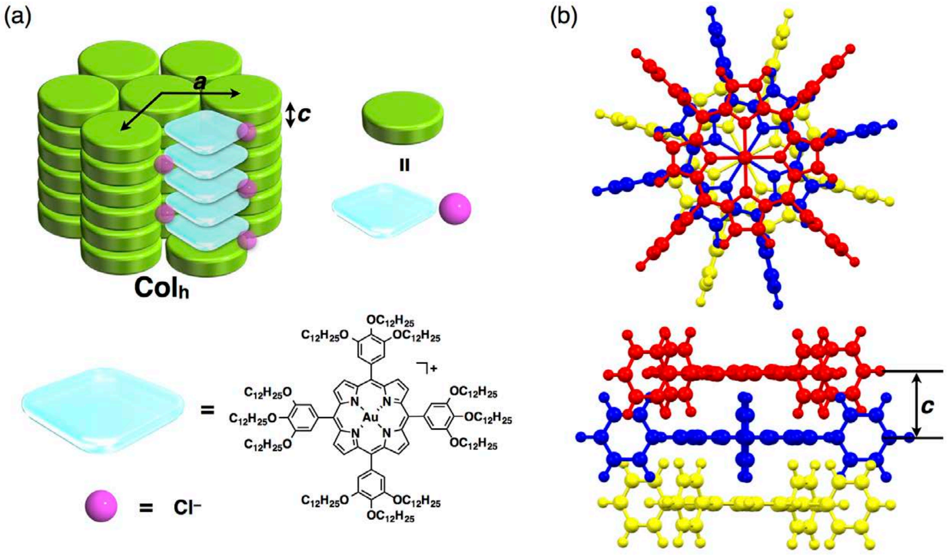

Figure S82 Possible packing model of Au12$^+$-Cl$^-$, Related to Table 1.

(a) Possible packing model of **Au12**$^+$-Cl$^-$ in a Col$_h$ structure and (b) columnar stacking model of the cationic Au^{III} complex (shown by geometry-optimized **Au0**$^+$ instead of **Au12**$^+$). Porphyrin–Au^{III} complexes are stacked with the distance of 0.35–0.37 nm (001 peak). Diffraction peak at 0.51–0.55 nm can be ascribable to the arrangement of peripheral aryl rings or coexisting Cl$^-$. Arrangement of the anions in the model structure of (a) is not exactly determined.

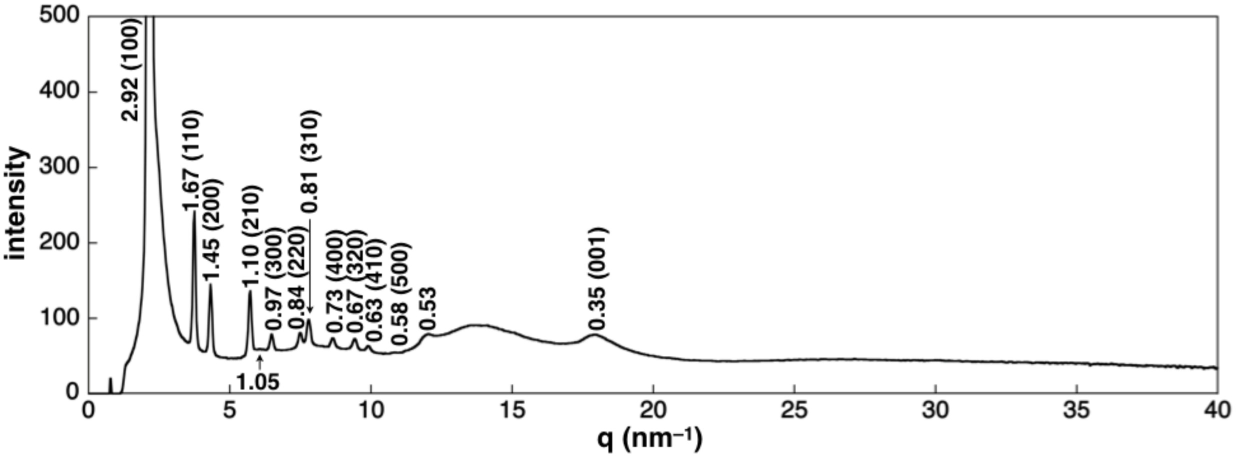

Figure S83 Wide-angle XRD of Au12$^+$-Cl$^-$, Related to Table 1.

Wide-angle XRD of **Au12**$^+$-Cl$^-$ at 100 °C (1st cooling). Wide-angle XRD clearly suggests the existence of the diffraction peak at 0.35 nm (Figure S80,81). The diffraction peak at 0.35 nm is derived from the stacking of porphyrin–Au^{III} complexes in the Col$_h$ packing structure (Table S7 and Figure S82).

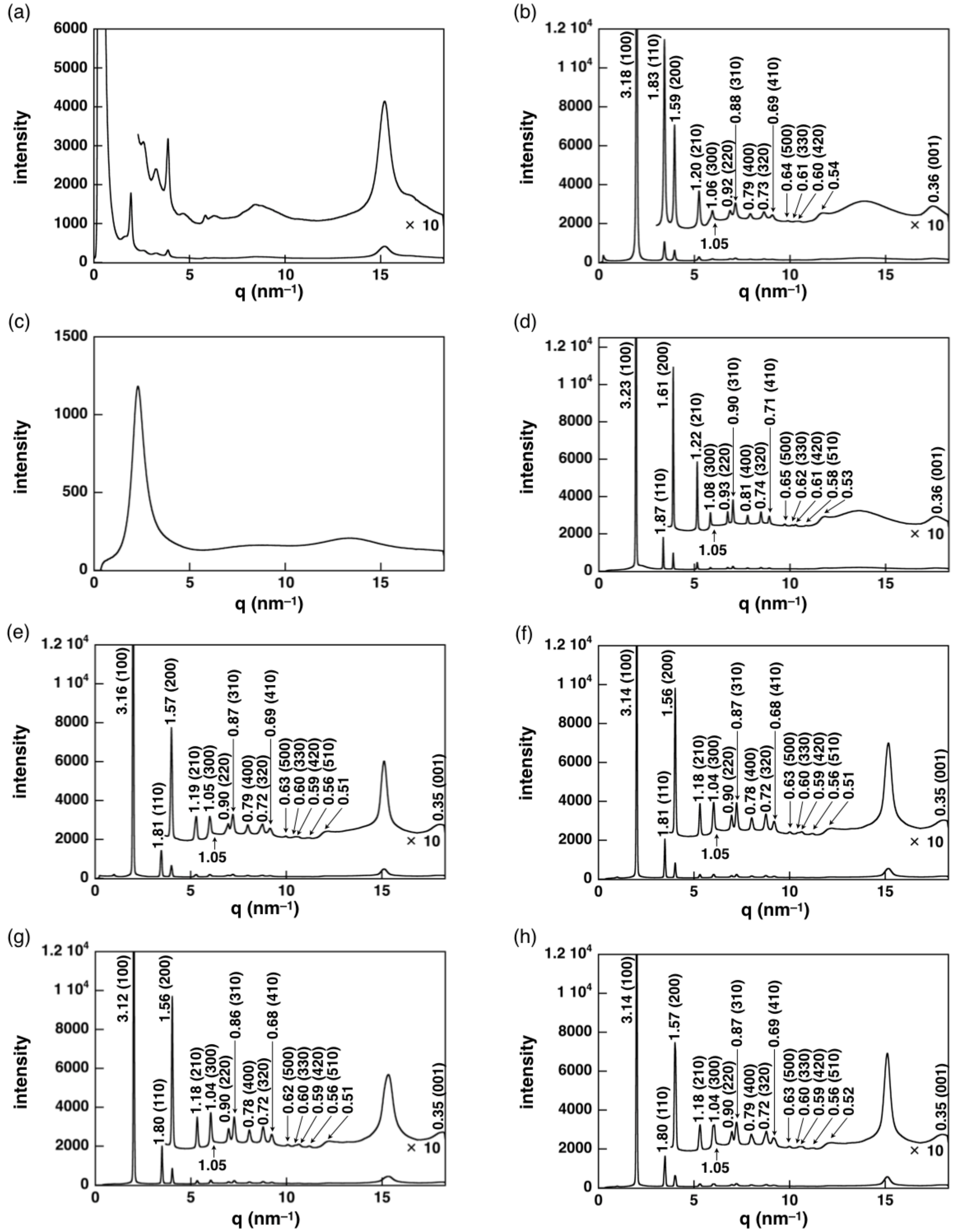

Figure S84 XRD patterns of Au16$^+$-Cl$^-$, Related to Table 1 and Figure 8.

XRD patterns of **Au16**$^+$-Cl$^-$ at (a) 25 °C (1st heating), (b) 60 °C (1st heating), (c) 140 °C (1st heating), (d) 100 °C (1st cooling), (e) 35 °C (1st cooling), (f) 32 °C (1st cooling), (g) 10 °C (1st cooling), and (h) 39 °C (2nd heating). The XRD patterns of (b,d–h) exhibit Col$_h$ structures (Figure S86). The broad peak around d = 1 nm is partially overlapped with other peaks.

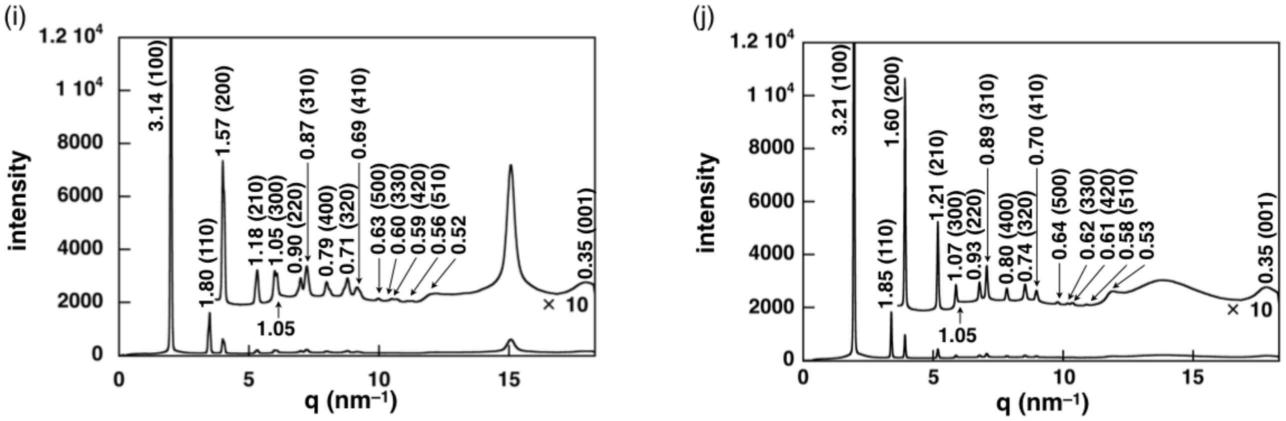

Figure S85 XRD patterns of Au16⁺-Cl⁻, Related to Table 1 and Figure 8.
XRD patterns of **Au16**⁺-Cl⁻ at (i) 41 °C (2nd heating) and (j) 60 °C (2nd heating) (Figure labels are continued from Figure S84). The XRD patterns of (i,j) exhibit Col_h structures (Figure S86). The broad peak around *d* = 1 nm is partially overlapped with other peaks.

Table S8 XRD peaks of Au16⁺-Cl⁻, Related to Table 1 and Figure 8.
XRD peaks of **Au16**⁺-Cl⁻ at (b) 60 °C (1st heating), (d) 100 °C (1st cooling), (e) 35 °C (1st cooling), (f) 32 °C (1st cooling), (g) 10 °C (1st cooling), (h) 39 °C (2nd heating), (i) 41 °C (2nd heating), and (j) 60 °C (2nd heating) (Figure S84,85). The peaks which can be indexed are represented.[a]

	q (nm⁻¹)	*d*-spacing (nm)	ratio	ratio (calc.)	*hkl*
(b) **Au16**⁺-Cl⁻<br>60 °C (1st heating)<br>Col_h<br>*a* = 3.67 nm, *c* = 0.35 nm<br>*M* = 3730.33, *Z* = 1 for *ρ* = 1.48	1.98	3.18	1.00	1.000	100
	3.43	1.83	0.58	0.577	110
	3.96	1.59	0.50	0.500	200
	5.25	1.20	0.38	0.378	210
	5.94	1.06	0.33	0.333	300
	6.86	0.92	0.29	0.289	220
	7.14	0.88	0.28	0.277	310
	7.93	0.79	0.25	0.250	400
	8.63	0.73	0.23	0.229	320
	9.08	0.69	0.22	0.218	410
	9.89	0.64	0.20	0.200	500
	10.28	0.61	0.19	0.192	330
	10.42	0.60	0.19	0.189	420
	17.46	0.36	–	–	001
(d) **Au16**⁺-Cl⁻<br>100 °C (1st cooling)<br>Col_h<br>*a* = 3.73 nm, *c* = 0.36 nm<br>*M* = 3730.33, *Z* = 1 for *ρ* = 1.44	1.94	3.23	1.00	1.000	100
	3.37	1.87	0.58	0.577	110
	3.89	1.61	0.50	0.500	200
	5.15	1.22	0.38	0.378	210
	5.84	1.08	0.33	0.333	300
	6.74	0.93	0.29	0.289	220
	7.02	0.90	0.28	0.277	310
	7.78	0.81	0.25	0.250	400
	8.49	0.74	0.23	0.229	320
	8.91	0.71	0.22	0.218	410
	9.73	0.65	0.20	0.200	500
	10.11	0.62	0.19	0.192	330
	10.29	0.61	0.19	0.189	420
	10.83	0.58	0.18	0.180	510
	17.65	0.36	–	–	001

Table S8 (Continued)

	q (nm^{-1})	*d*-spacing (nm)	ratio	ratio (calc.)	*hkl*
	1.99	3.16	1.00	1.000	100
	3.47	1.81	0.57	0.577	110
	3.99	1.57	0.50	0.500	200
	5.29	1.19	0.38	0.378	210
	6.00	1.05	0.33	0.333	300
(e) **Au16**$^{+}$-Cl^{-}	6.96	0.90	0.29	0.289	220
35 °C (1st cooling)	7.21	0.87	0.28	0.277	310
Col_h	7.98	0.79	0.25	0.250	400
a = 3.65 nm, c = 0.35 nm	8.77	0.72	0.23	0.229	320
M = 3730.33, Z = 1 for ρ = 1.55	9.16	0.69	0.22	0.218	410
	9.97	0.63	0.20	0.200	500
	10.48	0.60	0.19	0.192	330
	10.64	0.59	0.19	0.189	420
	11.22	0.56	0.18	0.180	510
	18.04	0.35	–	–	001
	2.00	3.14	1.00	1.000	100
	3.48	1.81	0.57	0.577	110
	4.02	1.56	0.50	0.500	200
	5.32	1.18	0.38	0.378	210
	6.03	1.04	0.33	0.333	300
(f) **Au16**$^{+}$-Cl^{-}	6.96	0.90	0.29	0.289	220
32 °C (1st cooling)	7.23	0.87	0.28	0.277	310
Col_h	8.02	0.78	0.25	0.250	400
a = 3.63 nm, c = 0.35 nm	8.76	0.72	0.23	0.229	320
M = 3730.33, Z = 1 for ρ = 1.56	9.19	0.68	0.22	0.218	410
	10.02	0.63	0.20	0.200	500
	10.42	0.60	0.19	0.192	330
	10.63	0.59	0.19	0.189	420
	11.21	0.56	0.18	0.180	510
	18.07	0.35	–	–	001
	2.01	3.12	1.00	1.000	100
	3.49	1.80	0.58	0.577	110
	4.03	1.56	0.50	0.500	200
	5.33	1.18	0.38	0.378	210
	6.03	1.04	0.33	0.333	300
(g) **Au16**$^{+}$-Cl^{-}	6.97	0.90	0.29	0.289	220
10 °C (1st cooling)	7.27	0.86	0.28	0.277	310
Col_h	8.06	0.78	0.25	0.250	400
a = 3.61 nm, c = 0.35 nm	8.78	0.72	0.23	0.229	320
M = 3730.33, Z = 1 for ρ = 1.57	9.23	0.68	0.22	0.218	410
	10.06	0.62	0.20	0.200	500
	10.48	0.60	0.19	0.192	330
	10.69	0.59	0.19	0.189	420
	11.19	0.56	0.18	0.180	510
	18.03	0.35	–	–	001

Table S8 (Continued)

	q (nm^{-1})	*d*-spacing (nm)	ratio	ratio (calc.)	*hkl*
	2.00	3.14	1.00	1.000	100
	3.49	1.80	0.57	0.577	110
	4.01	1.57	0.50	0.500	200
	5.32	1.18	0.38	0.378	210
	6.06	1.04	0.33	0.333	300
(h) **Au16**$^+$-Cl$^-$	7.00	0.90	0.29	0.289	220
39 °C (2nd heating)	7.22	0.87	0.28	0.277	310
Col$_h$	7.99	0.79	0.25	0.250	400
a = 3.63 nm, c = 0.35 nm	8.78	0.72	0.23	0.229	320
M = 3730.33, Z = 1 for ρ = 1.55	9.16	0.69	0.22	0.218	410
	9.99	0.63	0.20	0.200	500
	10.53	0.60	0.19	0.192	330
	10.66	0.59	0.19	0.189	420
	11.25	0.56	0.18	0.180	510
	17.94	0.35	–	–	001
	2.00	3.14	1.00	1.000	100
	3.49	1.80	0.57	0.577	110
	3.99	1.57	0.50	0.500	200
	5.32	1.18	0.38	0.378	210
	5.99	1.05	0.33	0.333	300
(i) **Au16**$^+$-Cl$^-$	6.99	0.90	0.29	0.289	220
41 °C (2nd heating)	7.22	0.87	0.28	0.277	310
Col$_h$	7.98	0.79	0.25	0.250	400
a = 3.63 nm, c = 0.35 nm	8.79	0.71	0.23	0.229	320
M = 3730.33, Z = 1 for ρ = 1.55	9.16	0.69	0.22	0.218	410
	9.97	0.63	0.20	0.200	500
	10.48	0.60	0.19	0.192	330
	10.68	0.59	0.19	0.189	420
	11.29	0.56	0.18	0.180	510
	17.98	0.35	–	–	001
	1.95	3.21	1.00	1.000	100
	3.39	1.85	0.58	0.577	110
	3.92	1.60	0.50	0.500	200
	5.18	1.21	0.38	0.378	210
	5.88	1.07	0.33	0.333	300
(j) **Au16**$^+$-Cl$^-$	6.78	0.93	0.29	0.289	220
60 °C (2nd heating)	7.06	0.89	0.28	0.277	310
Col$_h$	7.84	0.80	0.25	0.250	400
a = 3.71 nm, c = 0.35 nm	8.54	0.74	0.23	0.229	320
M = 3730.33, Z = 1 for ρ = 1.47	8.97	0.70	0.22	0.218	410
	9.80	0.64	0.20	0.200	500
	10.18	0.62	0.19	0.192	330
	10.35	0.61	0.19	0.189	420
	10.92	0.58	0.18	0.180	510
	17.81	0.35	–	–	001

[a] The diffraction peak which corresponds to 001 was clearly observed at the wide-angle region (Figure S87).

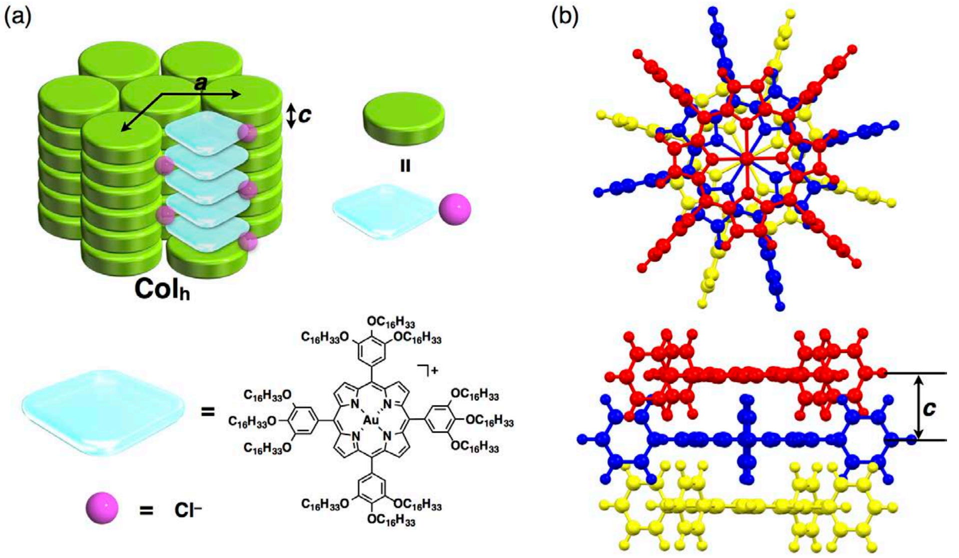

Figure S86 Possible packing model of Au16⁺-Cl⁻, Related to Table 1 and Figure 8.
(a) Possible packing model of **Au16**⁺-Cl⁻ in a Col_h structure and (b) columnar stacking model of the cationic Au^{III} complex (shown by geometry-optimized **Au0**⁺ instead of **Au16**⁺). Porphyrin–Au^{III} complexes are stacked with the distance of 0.35–0.36 nm (001 peak). Diffraction peak at 0.51–0.55 nm can be ascribable to the arrangement of peripheral aryl rings or coexisting Cl⁻. Arrangement of the anions in the model structure of (a) is not exactly determined.

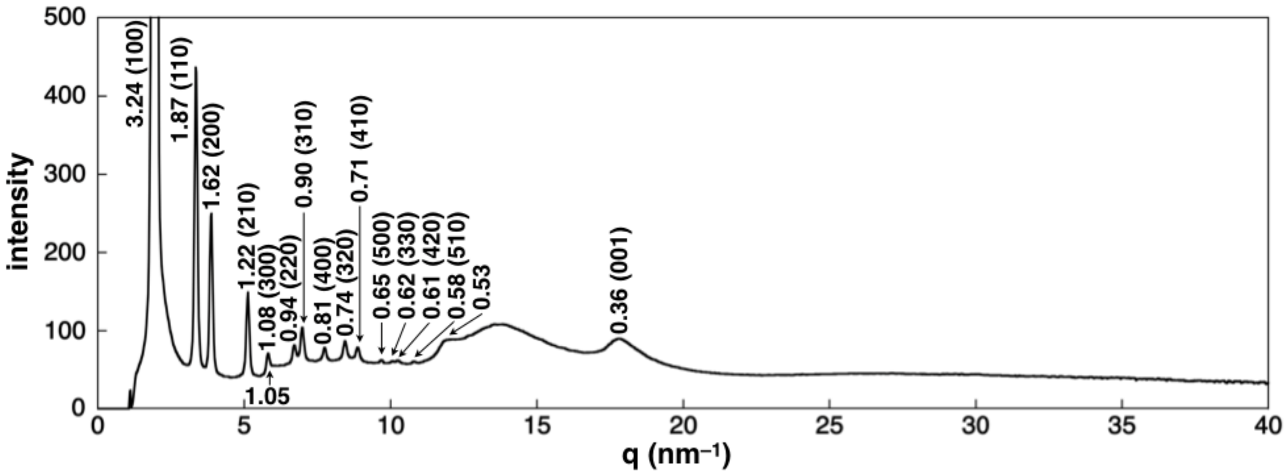

Figure S87 Wide-angle XRD of Au16⁺-Cl⁻, Related to Table 1 and Figure 8.
Wide-angle XRD of **Au16**⁺-Cl⁻ at 100 °C (1st cooling). Wide-angle XRD clearly suggests the existence of the diffraction peak at 0.36 nm (Figure S84,85). The diffraction peak at 0.36 nm is derived from the stacking of porphyrin–Au^{III} complexes in the Col_h packing structure (Table S8 and Figure S86).

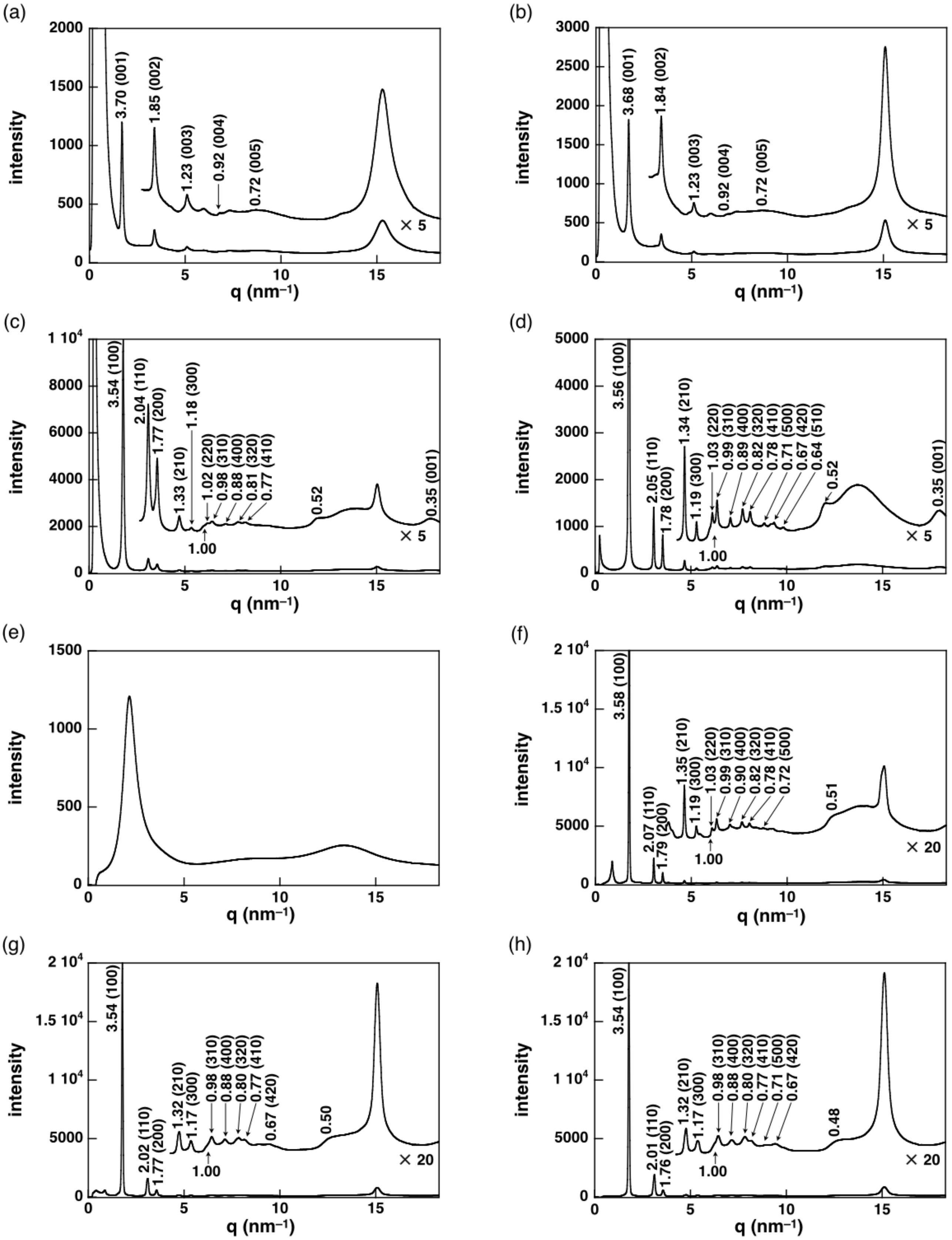

Figure S88 XRD patterns of Au20⁺-Cl⁻, Related to Table 1.

XRD patterns of **Au20**⁺-Cl⁻ at (a) 25 °C (1st heating), (b) 62 °C (1st heating), (c) 65 °C (1st heating), (d) 70 °C (1st heating), (e) 130 °C (1st heating), (f) 62 °C (1st cooling), (g) 58 °C (1st cooling), and (h) 54 °C (1st cooling). The XRD patterns of (a,b) and (c,d,f–h) exhibit lamellar and Col_h structures, respectively (Figure S90). The broad peak around *d* = 1 nm is partially overlapped with other peaks.

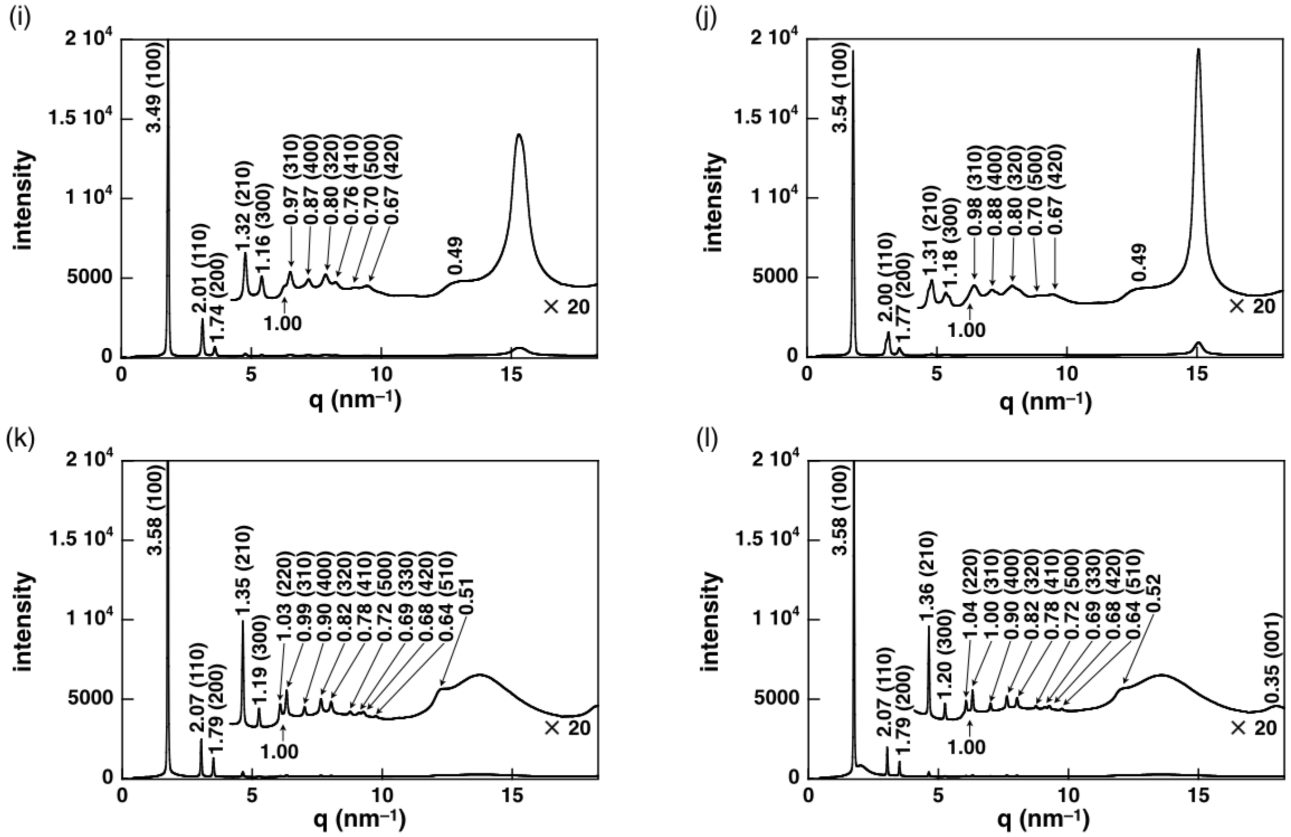

Figure S89 XRD patterns of Au20^{+}-Cl^{-}, Related to Table 1.

XRD patterns of **Au20**$^{+}$-Cl^{-} at (i) 25 °C (1st cooling), (j) 62 °C (2nd heating), (k) 70 °C (2nd heating), and (l) 100 °C (2nd heating) (Figure labels are continued from Figure S88). The XRD patterns of (i–l) exhibit lamellar and Col$_{h}$ structures, respectively (Figure S90). The broad peak around *d* = 1 nm is partially overlapped with other peaks.

Table S9 XRD peaks of Au20^{+}-Cl^{-}, Related to Table 1.

XRD peaks of **Au20**$^{+}$-Cl^{-} at (a) 25 °C (1st heating), (b) 62 °C (1st heating), (c) 65 °C (1st heating), (d) 70 °C (1st heating), (f) 62 °C (1st cooling), (g) 58 °C (1st cooling), (h) 54 °C (1st cooling), (i) 25 °C (1st cooling), (j) 62 °C (2nd heating), (k) 70 °C (2nd heating), and (l) 100 °C (2nd heating) (Figure S88,89). The peaks which can be indexed are represented.

	q (nm^{-1})	*d*-spacing (nm)	ratio	ratio (calc.)	*hkl*
	1.70	3.70	1.00	1.000	001
(a) **Au20**$^{+}$-Cl^{-}	3.39	1.85	0.50	0.500	002
25 °C (1st heating)	5.09	1.23	0.33	0.333	003
lamellar	6.81	0.92	0.25	0.250	004
	8.67	0.72	0.20	0.200	005
	1.71	3.68	1.00	1.000	001
(b) **Au20**$^{+}$-Cl^{-}	3.41	1.84	0.50	0.500	002
62 °C (1st heating)	5.12	1.23	0.33	0.333	003
lamellar	6.84	0.92	0.25	0.250	004
	8.72	0.72	0.20	0.200	005

Table S9 (Continued)

	q (nm^{-1})	*d*-spacing (nm)	ratio	ratio (calc.)	*hkl*
	1.78	3.54	1.00	1.000	100
	3.09	2.04	0.58	0.577	110
	3.56	1.77	0.50	0.500	200
(c) Au20^{+}-Cl^{-}	4.71	1.33	0.38	0.378	210
65 °C (1st heating)	5.34	1.18	0.33	0.333	300
Col$_h$	6.19	1.02	0.29	0.289	220
a = 4.08 nm, c = 0.35 nm	6.41	0.98	0.28	0.277	310
M = 4403.62, Z = 1 for ρ = 1.44	7.12	0.88	0.25	0.250	400
	7.76	0.81	0.23	0.229	320
	8.14	0.77	0.22	0.218	410
	17.85	0.35	–	–	001
	1.77	3.56	1.00	1.000	100
	3.06	2.05	0.58	0.577	110
	3.53	1.78	0.50	0.500	200
	4.68	1.34	0.38	0.378	210
(d) **Au20**$^{+}$-Cl^{-}	5.30	1.19	0.33	0.333	300
70 °C (1st heating)	6.12	1.03	0.29	0.289	220
Col$_h$	6.37	0.99	0.28	0.277	310
a = 4.11 nm, c = 0.35 nm	7.06	0.89	0.25	0.250	400
M = 4403.62, Z = 1 for ρ = 1.43	7.70	0.82	0.23	0.229	320
	8.09	0.78	0.22	0.218	410
	8.83	0.71	0.20	0.200	500
	9.34	0.67	0.19	0.189	420
	9.82	0.64	0.18	0.180	510
	17.99	0.35	–	–	001
	1.75	3.58	1.00	1.000	100
	3.04	2.07	0.58	0.577	110
	3.50	1.79	0.50	0.500	200
(f) **Au20**$^{+}$-Cl^{-}	4.64	1.35	0.38	0.378	210
62 °C (1st cooling)	5.26	1.19	0.33	0.333	300
Col$_h$[a]	6.09	1.03	0.29	0.289	220
a = 4.14 nm	6.32	0.99	0.28	0.277	310
	7.01	0.90	0.25	0.250	400
	7.64	0.82	0.23	0.229	320
	8.03	0.78	0.22	0.218	410
	8.77	0.72	0.20	0.200	500
	1.78	3.54	1.00	1.000	100
	3.11	2.02	0.57	0.577	110
	3.56	1.77	0.50	0.500	200
(g) **Au20**$^{+}$-Cl^{-}	4.76	1.32	0.37	0.378	210
58 °C (1st cooling)	5.35	1.17	0.33	0.333	300
Col$_h$[a]	6.44	0.98	0.28	0.277	310
a = 4.08 nm	7.14	0.88	0.25	0.250	400
	7.85	0.80	0.23	0.229	320
	8.14	0.77	0.22	0.218	410
	9.38	0.67	0.19	0.189	420

Table S9 (Continued)

	q (nm⁻¹)	*d*-spacing (nm)	ratio	ratio (calc.)	*hkl*
(h) **Au20**⁺-Cl⁻ 54 °C (1st cooling) Col_h[a] *a* = 4.08 nm	1.78	3.54	1.00	1.000	100
	3.12	2.01	0.57	0.577	110
	3.57	1.76	0.50	0.500	200
	4.78	1.32	0.37	0.378	210
	5.36	1.17	0.33	0.333	300
	6.44	0.98	0.28	0.277	310
	7.15	0.88	0.25	0.250	400
	7.84	0.80	0.23	0.229	320
	8.17	0.77	0.22	0.218	410
	8.90	0.71	0.20	0.200	500
	9.43	0.67	0.19	0.189	420
(i) **Au20**⁺-Cl⁻ 25 °C (1st cooling) Col_h[a] *a* = 4.03 nm	1.80	3.49	1.00	1.000	100
	3.12	2.01	0.58	0.577	110
	3.60	1.74	0.50	0.500	200
	4.77	1.32	0.38	0.378	210
	5.40	1.16	0.33	0.333	300
	6.50	0.97	0.28	0.277	310
	7.20	0.87	0.25	0.250	400
	7.86	0.80	0.23	0.229	320
	8.23	0.76	0.22	0.218	410
	9.02	0.70	0.20	0.200	500
	9.43	0.67	0.19	0.189	420
(j) **Au20**⁺-Cl⁻ 62 °C (2nd heating) Col_h[a] *a* = 4.03 nm	1.78	3.54	1.00	1.000	100
	3.14	2.00	0.58	0.577	110
	3.56	1.77	0.50	0.500	200
	4.80	1.31	0.38	0.378	210
	5.33	1.18	0.33	0.333	300
	6.44	0.98	0.28	0.277	310
	7.12	0.88	0.25	0.250	400
	7.90	0.80	0.23	0.229	320
	8.95	0.70	0.20	0.218	500
	9.41	0.67	0.19	0.200	420
(k) **Au20**⁺-Cl⁻ 70 °C (2nd heating) Col_h[a] *a* = 4.14 nm	1.75	3.58	1.00	1.000	100
	3.04	2.07	0.58	0.577	110
	3.50	1.79	0.50	0.500	200
	4.64	1.35	0.38	0.378	210
	5.26	1.19	0.33	0.333	300
	6.08	1.03	0.29	0.289	220
	6.32	0.99	0.28	0.277	310
	7.01	0.90	0.25	0.250	400
	7.64	0.82	0.23	0.229	320
	8.04	0.78	0.22	0.218	410
	8.77	0.72	0.20	0.200	500
	9.12	0.69	0.19	0.192	330
	9.28	0.68	0.19	0.189	420
	9.75	0.64	0.18	0.180	510

	q (nm^{-1})	*d*-spacing (nm)	ratio	ratio (calc.)	*hkl*
	1.75	3.58	1.00	1.000	100
	3.03	2.07	0.58	0.577	110
	3.50	1.79	0.50	0.500	200
	4.63	1.36	0.38	0.378	210
	5.25	1.20	0.33	0.333	300
(l) Au20^{+}-Cl^{-}	6.06	1.04	0.29	0.289	220
100 °C (2nd heating)	6.31	1.00	0.28	0.277	310
Col$_h$	7.00	0.90	0.25	0.250	400
a = 4.14 nm, c = 0.35 nm	7.63	0.82	0.23	0.229	320
M = 4403.62, Z = 1 for ρ = 1.41	8.03	0.78	0.22	0.218	410
	8.76	0.72	0.20	0.200	500
	9.10	0.69	0.19	0.192	330
	9.26	0.68	0.19	0.189	420
	9.75	0.64	0.18	0.180	510
	18.03	0.35	–	–	001

[a] The diffraction peak which corresponds to 001 was observed at the wide-angle region (Figure S91).

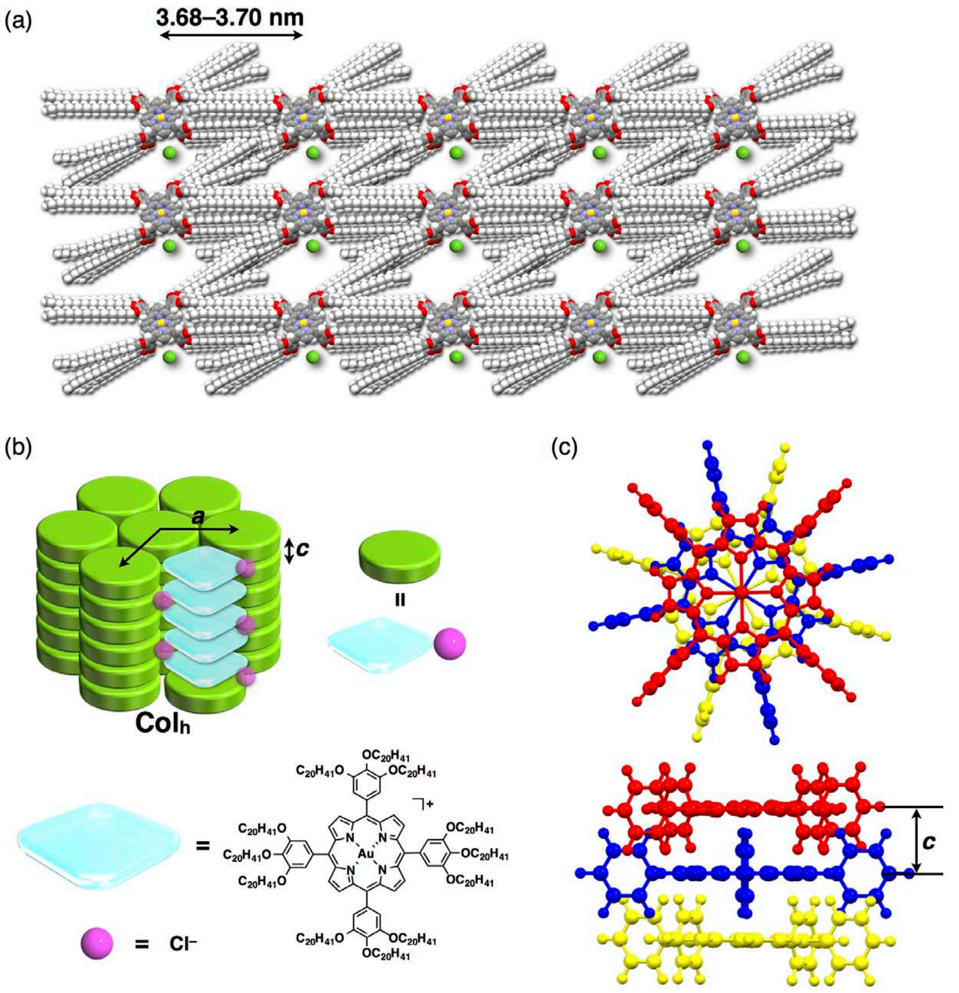

Figure S90 Possible packing models of Au20^{+}-Cl^{-}, Related to Table 1.
Possible packing models of **Au20**$^{+}$-Cl^{-} in (a) lamellar, (b) Col$_h$ structures, and (c) columnar stacking model of the cationic Au^{III} complex (shown by geometry-optimized **Au0**$^{+}$ instead of **Au20**$^{+}$). Porphyrin–Au^{III} complexes are stacked with the distance of 0.35 nm (001 peak). Diffraction peak at 0.48–0.52 nm can be ascribable to the arrangement of peripheral aryl rings or coexisting Cl^{-}. Arrangement of the anions in the model structure of (b) is not exactly determined.

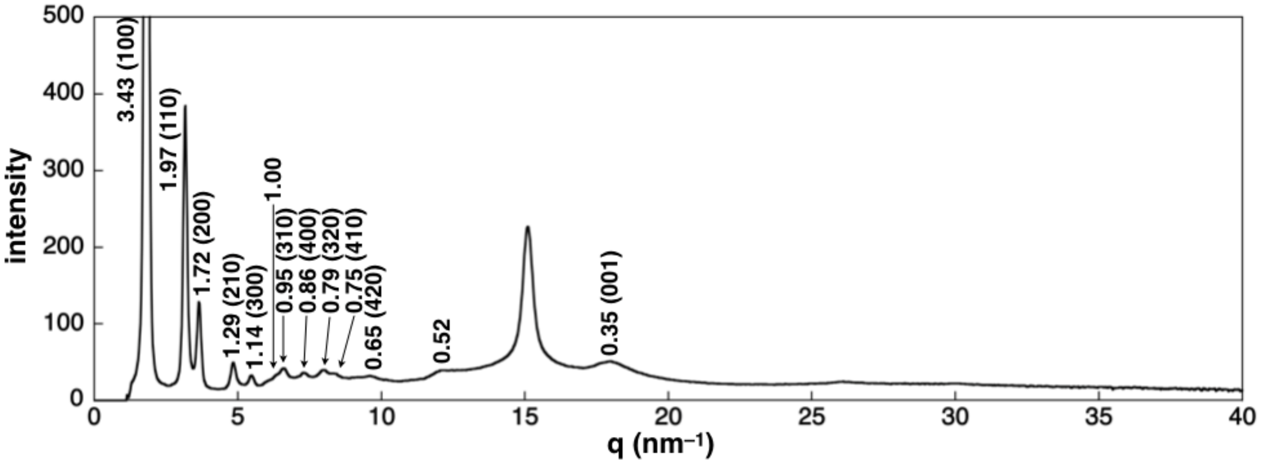

Figure S91 Wide-angle XRD of Au20^{+}-Cl^{-}, Related to Table 1.

Wide-angle XRD of **Au20**$^{+}$-Cl^{-} at 58 °C (1st cooling). Wide-angle XRD clearly suggests the existence of the diffraction peak at 0.35 nm (Figure S88,89). The diffraction peak at 0.35 nm is derived from the stacking of porphyrin–Au^{III} complexes in the Col$_h$ packing structure (Table S9 and Figure S90).

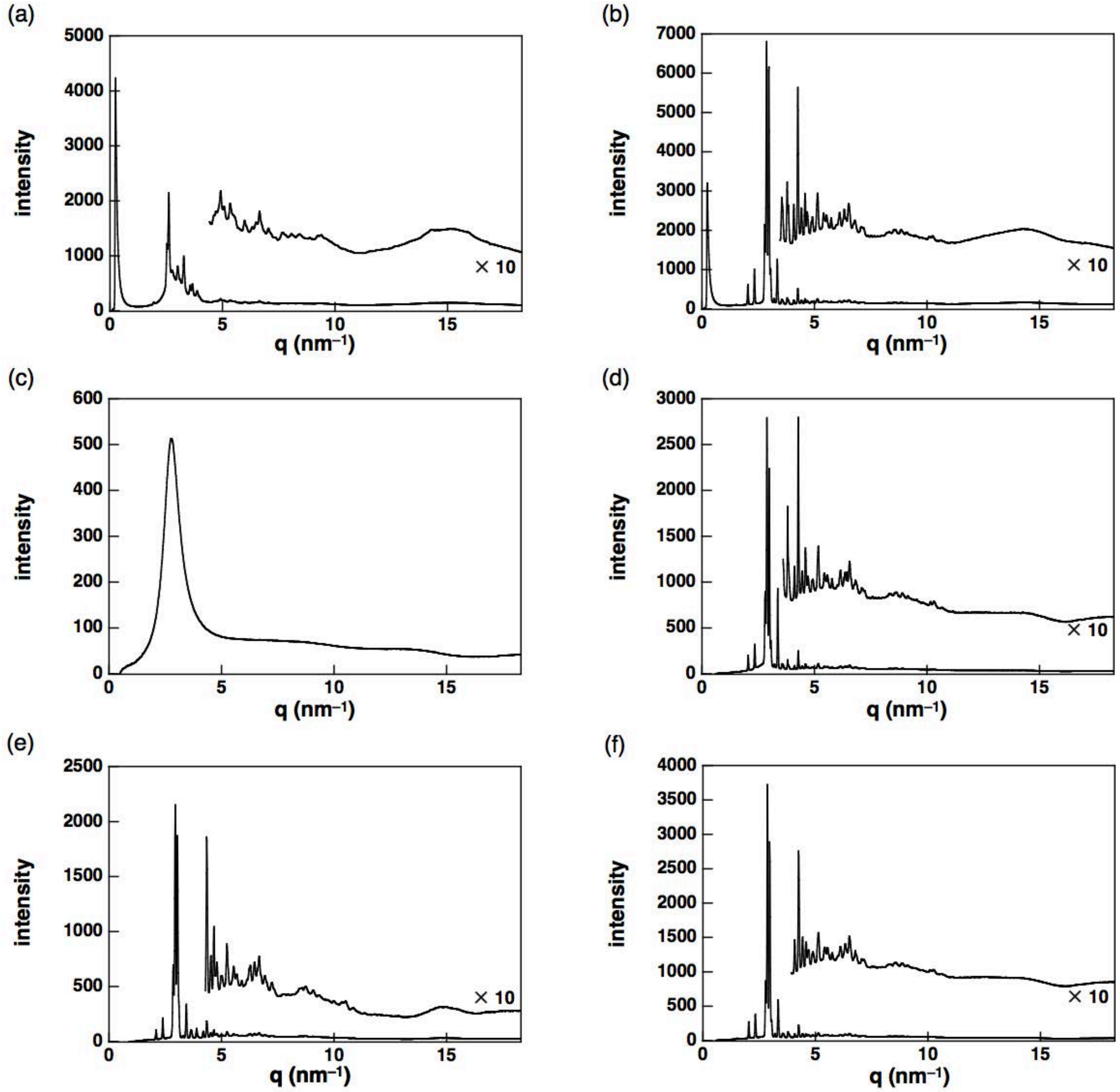

Figure S92 XRD patterns of Au8^{+}-BF$_4^{-}$, Related to Table 1.

XRD patterns of **Au8**$^{+}$-BF$_4^{-}$ at (a) 25 °C (1st heating), (b) 150 °C (1st heating), (c) 190 °C (1st heating), (d) 120 °C (1st cooling), (e) 25 °C (1st cooling), and (f) 150 °C (2nd heating). Complicated peak patters were obtained for (a,b,d–f), suggesting the highly crystalline states.

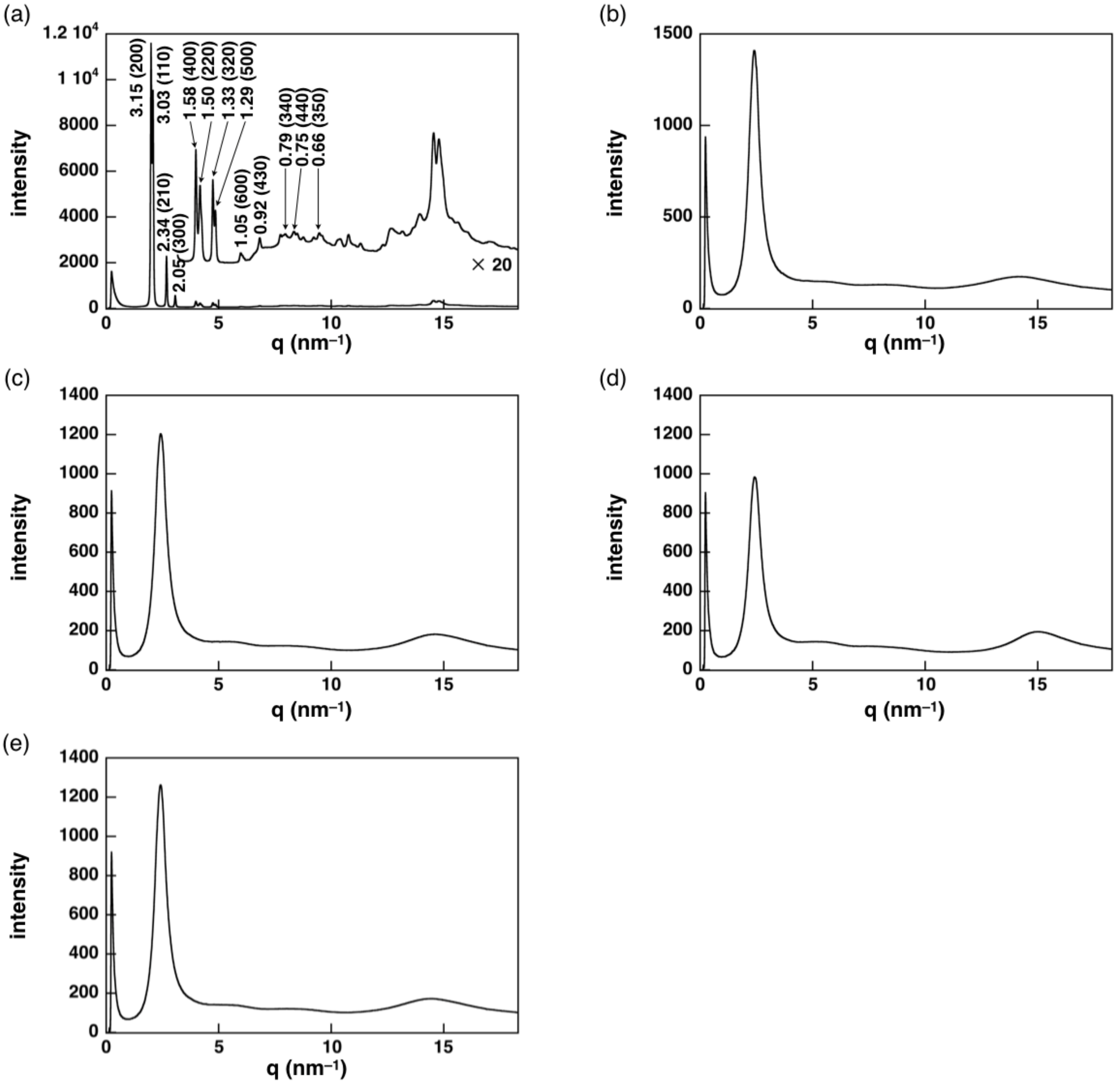

Figure S93 XRD patterns of Au12^{+}-BF_4^{-}, Related to Table 1.

XRD patterns of **Au12**$^{+}$-BF_4^{-} at (a) 25 °C (1st heating), (b) 70 °C (1st heating), (c) –10 °C (1st cooling), (d) –65 °C (1st cooling), and (e) 25 °C (2nd heating). The XRD pattern of (a) exhibits a Col_r structure (Figure S94).

Table S10 XRD peaks of Au12$^+$-BF$_4^-$, Related to Table 1.

XRD peaks of **Au12**$^+$-BF$_4^-$ at (a) 25 °C (1st heating) (Figure S93). The peaks which can be indexed are represented.

	q (nm^{-1})	*d*-spacing (nm)	ratio	ratio (calc.)	*hkl*
(a) **Au12**$^+$-BF$_4^-$ 25 °C (1st heating) Col$_r$ *a* = 6.29 nm, *b* = 3.45 nm	2.00	3.15	1.00	1.000	200
	2.08	3.03	0.96	0.962	110
	2.68	2.34	0.74	0.739	210
	3.07	2.05	0.65	0.667	300
	3.99	1.58	0.50	0.500	400
	4.18	1.50	0.48	0.481	220
	4.74	1.33	0.42	0.424	320
	4.85	1.29	0.41	0.400	500
	5.98	1.05	0.33	0.333	600
	6.82	0.92	0.29	0.295	430
	7.96	0.79	0.25	0.254	340
	8.34	0.75	0.24	0.241	440
	9.48	0.66	0.21	0.208	350

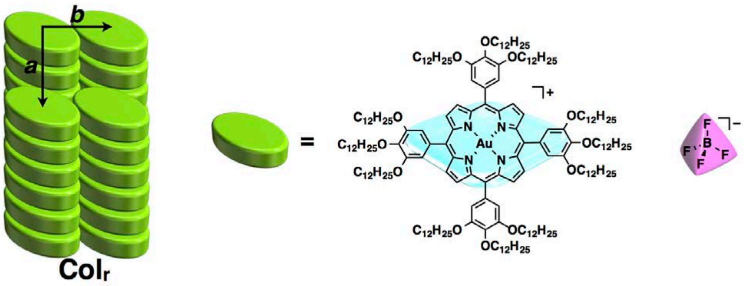

Figure S94 Possible packing model of Au12$^+$-BF$_4^-$, Related to Table 1.

Possible packing model of **Au12**$^+$-BF$_4^-$ in a Col$_r$ structure.

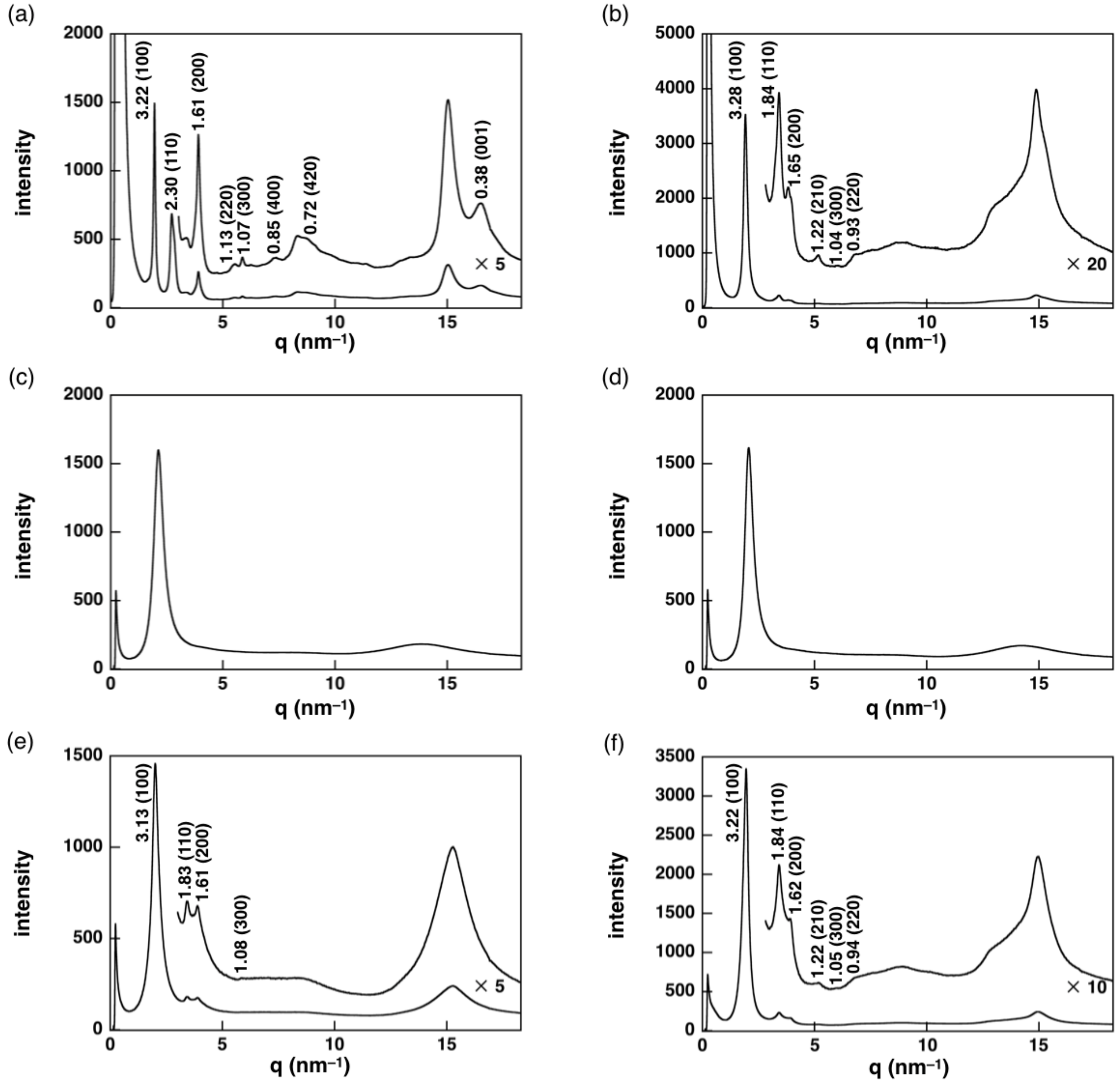

Figure S95 XRD patterns of Au16$^+$-BF_4^-, Related to Table 1.

XRD patterns of **Au16**$^+$-BF_4^- at (a) 25 °C (1st heating), (b) 50 °C (1st heating), (c) 100 °C (1st heating), (d) 35 °C (1st cooling), (e) –30 °C (1st cooling), and (f) 45 °C (2nd heating). The XRD patterns of (a) and (b,e,f) exhibit Col$_t$ and Col$_h$ structures, respectively (Figure S96).

Table S11 XRD peaks of Au16$^+$-BF_4^-, Related to Table 1.

XRD peaks of **Au16**$^+$-BF_4^- at (a) 25 °C (1st heating), (b) 50 °C (1st heating), (e) –30 °C (1st cooling), and (f) 45 °C (2nd heating) (Figure S95). The peaks which can be indexed are represented.

	q (nm^{-1})	*d*-spacing (nm)	ratio	ratio (calc.)	*hkl*
(a) **Au16**$^+$-BF_4^-<br>25 °C (1st heating)<br>$\mathrm{Col_t}$<br>a = 3.22 nm, c = 0.38 nm<br>M = 3781.68, Z = 1 for ρ = 1.60	1.95	3.22	1.00	1.000	100
	2.73	2.30	0.72	0.707	110
	3.91	1.61	0.50	0.500	200
	5.54	1.13	0.35	0.354	220
	5.88	1.07	0.33	0.333	300
	7.39	0.85	0.26	0.250	400
	8.68	0.72	0.22	0.224	420
	16.49	0.38	–	–	001
(b) **Au16**$^+$-BF_4^-<br>50 °C (1st heating)<br>$\mathrm{Col_h}^a$<br>a = 3.78 nm	1.92	3.28	1.00	1.00	100
	3.41	1.84	0.56	0.577	110
	3.82	1.65	0.50	0.500	200
	5.17	1.22	0.37	0.378	210
	6.02	1.04	0.32	0.333	300
	6.78	0.93	0.28	0.289	220
(e) **Au16**$^+$-BF_4^-<br>–30 °C (1st cooling)<br>$\mathrm{Col_h}^a$<br>a = 3.61 nm	2.01	3.13	1.00	1.00	100
	3.43	1.83	0.59	0.577	110
	3.90	1.61	0.52	0.500	200
	5.80	1.08	0.35	0.333	300
(f) **Au16**$^+$-BF_4^-<br>45 °C (2nd heating)<br>$\mathrm{Col_h}^a$<br>a = 3.72 nm	1.95	3.22	1.00	1.00	100
	3.41	1.84	0.57	0.577	110
	3.89	1.62	0.50	0.500	200
	5.16	1.22	0.38	0.378	210
	5.96	1.05	0.33	0.333	300
	6.71	0.94	0.29	0.289	220

^{a} Z and ρ values are not given due to the unclear height value (c) in XRD chart.

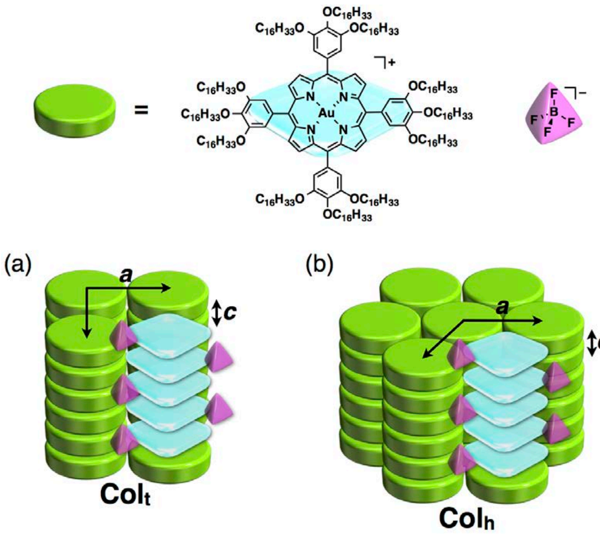

Figure S96 Possible packing models of Au16$^+$-BF_4^-, Related to Table 1.

Possible packing models of **Au16**$^+$-BF_4^- in (a) $\mathrm{Col_t}$ and (b) $\mathrm{Col_h}$ structures. Arrangement of the anions in the model structures of (a) and (b) is not exactly determined.

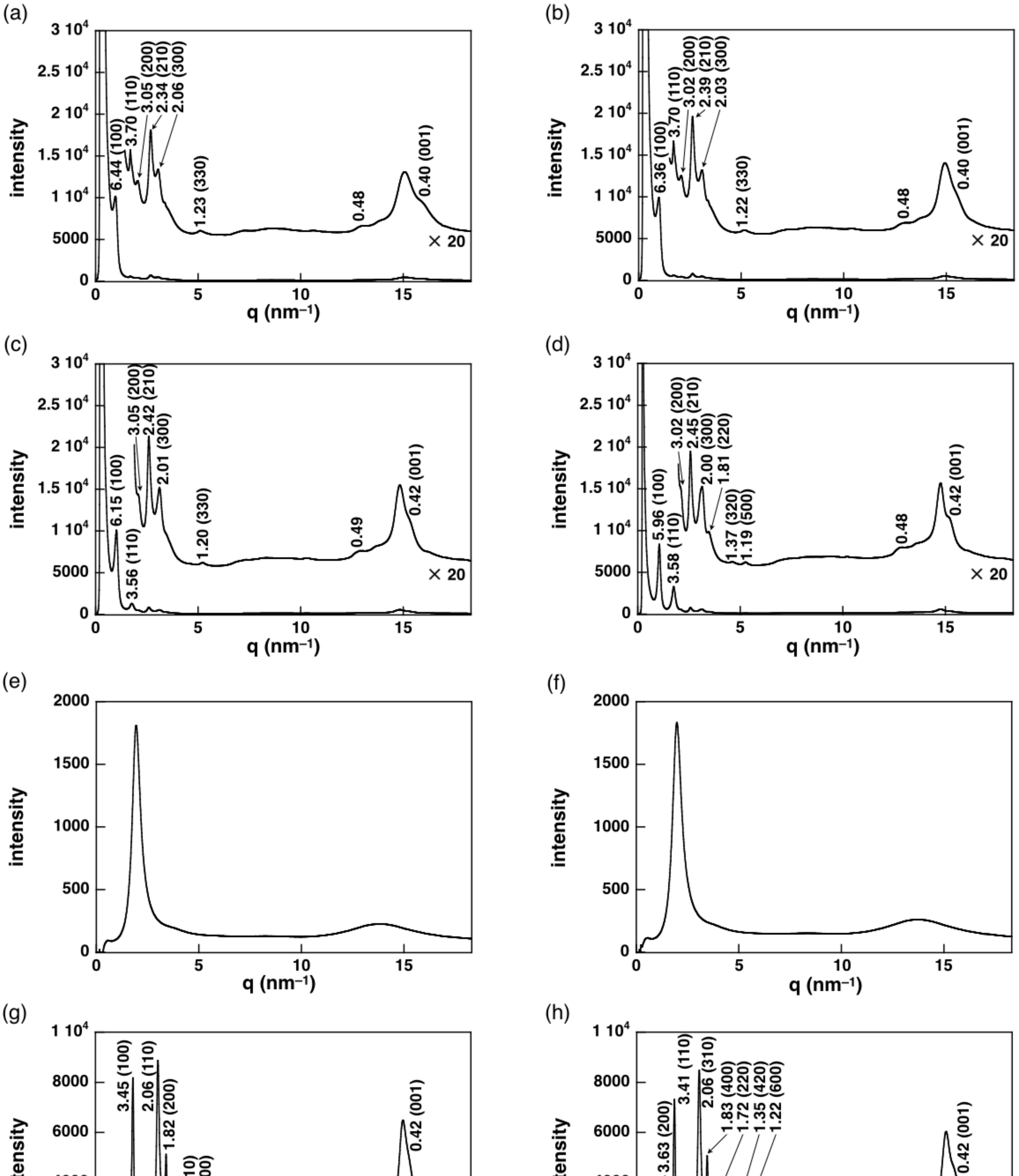

Figure S97 XRD patterns of Au20$^+$-BF_4^-, Related to Table 1 and Figure 8.

XRD patterns of **Au20**$^+$-BF_4^- at (a) 25 °C (1st heating), (b) 58 °C (1st heating), (c) 66 °C (1st heating), (d) 75 °C (1st heating), (e) 90 °C (1st heating), (f) 110 °C (1st heating), (g) 52 °C (1st cooling), and (h) 35 °C (1st cooling). The XRD patterns of (a–d,g) and (h) exhibit Col_h and Col_r structures, respectively (Figure S99).

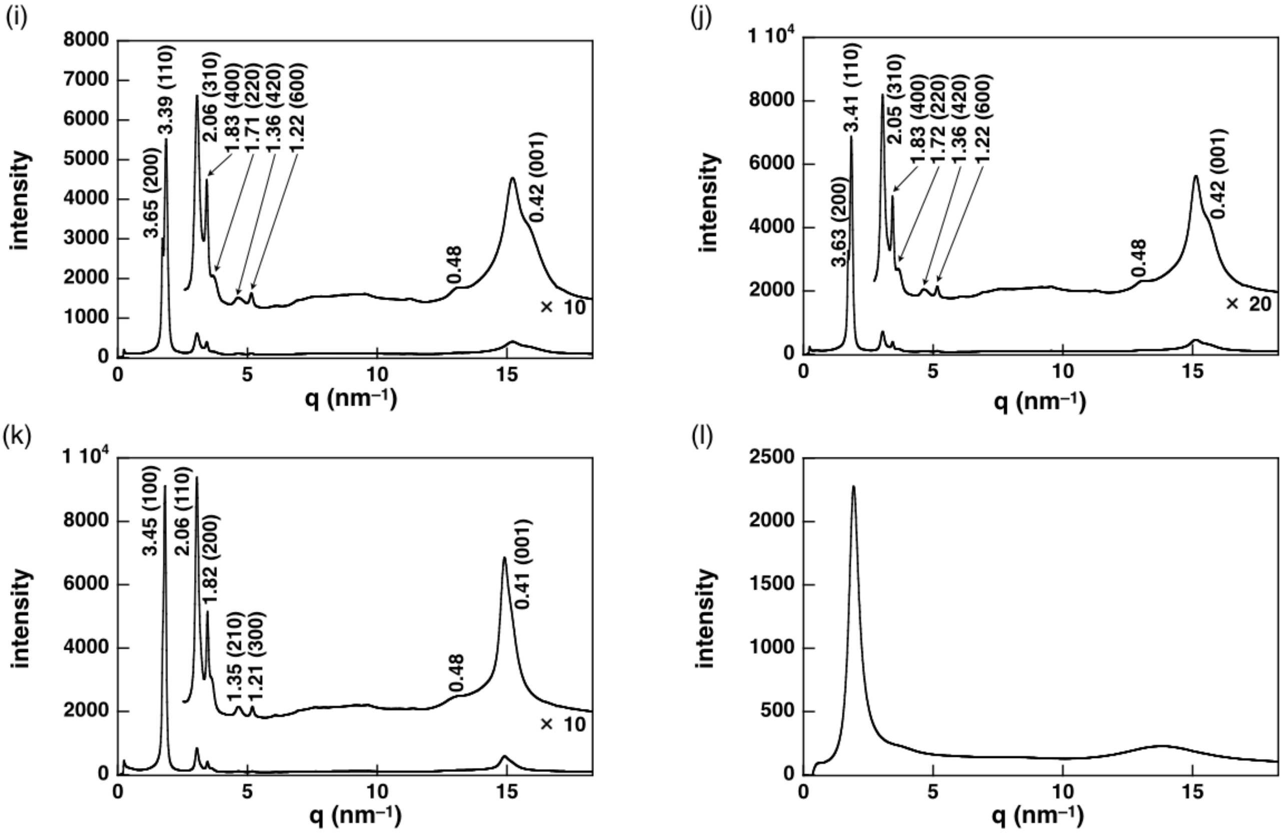

Figure S98 XRD patterns of Au20$^+$-BF$_4^-$, Related to Table 1 and Figure 8.
XRD patterns of **Au20**$^+$-BF$_4^-$ at (i) –10 °C (1st cooling), (j) 25 °C (2nd heating), (k) 65 °C (2nd heating), and (l) 90 °C (2nd heating) (Figure labels are continued from Figure S97). The XRD patterns of (k) and (i,j) exhibit Col$_h$ and Col$_r$ structures, respectively (Figure S99).

Table S12 XRD peaks of Au20$^+$-BF$_4^-$, Related to Table 1 and Figure 8.
XRD peaks of **Au20**$^+$-BF$_4^-$ at (a) 25 °C (1st heating), (b) 58 °C (1st heating), (c) 66 °C (1st heating), (d) 75 °C (1st heating), (g) 52 °C (1st cooling), (h) 35 °C (1st cooling), (i) –10 °C (1st cooling), (j) 25 °C (2nd heating), and (k) 65 °C (2nd heating) (Figure S97,98). The peaks which can be indexed are represented.

	q (nm^{-1})	*d*-spacing (nm)	ratio	ratio (calc.)	*hkl*
	0.98	6.44	1.00	1.000	100
(a) **Au20**$^+$-BF$_4^-$	1.70	3.70	0.57	0.577	110
25 °C (1st heating)	2.06	3.05	0.47	0.500	200
Col$_h$	2.69	2.34	0.36	0.378	210
a = 7.43 nm, *c* = 0.40 nm	3.05	2.06	0.32	0.333	300
M = 4454.97, *Z* = 2 for ρ = 0.80	5.09	1.23	0.19	0.192	330
	15.90	0.40	–	–	001
	0.99	6.36	1.00	1.000	100
(b) **Au20**$^+$-BF$_4^-$	1.70	3.70	0.58	0.577	110
58 °C (1st heating)	2.08	3.02	0.47	0.500	200
Col$_h$	2.63	2.39	0.37	0.378	210
a = 7.35 nm, *c* = 0.40 nm	3.10	2.03	0.32	0.333	300
M = 4454.97, *Z* = 2 for ρ = 0.76	5.14	1.22	0.19	0.192	330
	15.90	0.40	–	–	001
	1.02	6.15	1.00	1.000	100
(c) **Au20**$^+$-BF$_4^-$	1.77	3.56	0.58	0.577	110
66 °C (1st heating)	2.06	3.05	0.50	0.500	200
Col$_h$	2.60	2.42	0.39	0.378	210
a = 7.11 nm, *c* = 0.42 nm	3.12	2.01	0.33	0.333	300
M = 4454.97, *Z* = 2 for ρ = 0.80	5.22	1.20	0.20	0.192	330
	14.83	0.42	–	–	001

Table S12 (Continued)

	q (nm^{-1})	*d*-spacing (nm)	ratio	ratio (calc.)	*hkl*
(d) **Au20**$^{+}$-BF_4^{-} 75 °C (1st heating) Col_h *a* = 6.88 nm, *c* = 0.42 nm *M* = 4454.97, *Z* = 2 for *ρ* = 0.87	1.05	5.96	1.00	1.000	100
	1.75	3.58	0.60	0.577	110
	2.08	3.02	0.51	0.500	200
	2.57	2.45	0.41	0.378	210
	3.14	2.00	0.34	0.333	300
	3.47	1.81	0.30	0.289	220
	4.60	1.37	0.23	0.229	320
	5.26	1.19	0.20	0.200	500
	15.11	0.42	–	–	001
(g) **Au20**$^{+}$-BF_4^{-} 52 °C (1st cooling) Col_h *a* = 3.98 nm, *c* = 0.42 nm *M* = 4454.97, *Z* = 1 for *ρ* = 1.30	1.82	3.45	1.00	1.000	100
	3.05	2.06	0.60	0.577	110
	3.45	1.82	0.53	0.500	200
	4.65	1.35	0.39	0.378	210
	5.17	1.21	0.35	0.333	300
	15.00	0.42	–	–	001
(h) **Au20**$^{+}$-BF_4^{-} 35 °C (1st cooling) Col_r (*c*2*mm*) *a* = 7.26 nm, *b* = 3.88 nm, *c* = 0.42 nm *M* = 4454.97, *Z* = 2 for *ρ* = 1.27	1.73	3.63	1.00	1.000	200
	1.84	3.41	0.94	0.941	110
	3.05	2.06	0.57	0.565	310
	3.44	1.83	0.50	0.500	400
	3.65	1.72	0.47	0.471	220
	4.65	1.35	0.37	0.365	420
	5.16	1.22	0.34	0.333	600
	15.09	0.42	–	–	001
(i) **Au20**$^{+}$-BF_4^{-} –10 °C (1st cooling) Col_r (*c*2*mm*) *a* = 7.30 nm, *b* = 3.82 nm, *c* = 0.42 nm *M* = 4454.97, *Z* = 2 for *ρ* = 1.29	1.72	3.65	1.00	1.000	200
	1.86	3.39	0.93	0.927	110
	3.05	2.06	0.56	0.562	310
	3.44	1.83	0.50	0.500	400
	3.67	1.71	0.47	0.464	220
	4.63	1.36	0.37	0.361	420
	5.15	1.22	0.33	0.333	600
	15.11	0.42	–	–	001
(j) **Au20**$^{+}$-BF_4^{-} 25 °C (2nd heating) Col_r (*c*2*mm*) *a* = 7.26 nm, *b* = 3.86 nm, *c* = 0.42 nm *M* = 4454.97, *Z* = 2 for *ρ* = 1.28	1.73	3.63	1.00	1.000	200
	1.84	3.41	0.94	0.939	110
	3.06	2.05	0.57	0.565	310
	3.44	1.83	0.50	0.500	400
	3.65	1.72	0.47	0.469	220
	4.63	1.36	0.37	0.364	420
	5.16	1.22	0.34	0.333	600
	15.12	0.42	–	–	001
(k) **Au20**$^{+}$-BF_4^{-} 65 °C (2nd heating) Col_h *a* = 3.98 nm, *c* = 0.41 nm *M* = 4454.97, *Z* = 1 for *ρ* = 1.31	1.82	3.45	1.00	1.000	100
	3.05	2.06	0.60	0.577	110
	3.46	1.82	0.53	0.500	200
	4.64	1.35	0.39	0.378	210
	5.18	1.21	0.35	0.333	300
	15.20	0.41	–	–	001

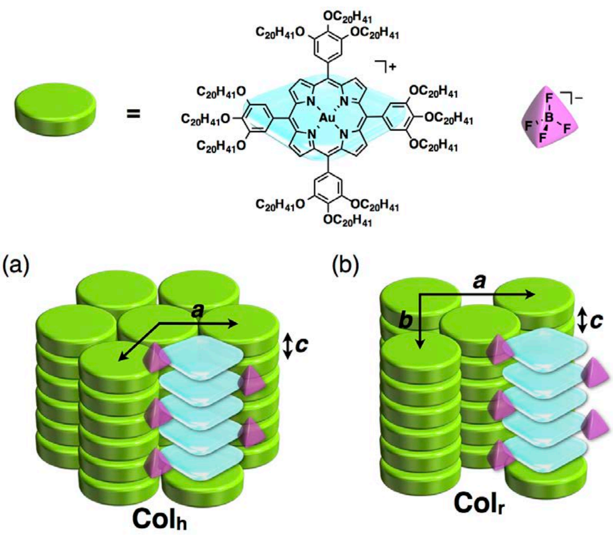

Figure S99 Possible packing models of Au20$^+$-BF_4^-, Related to Table 1 and Figure 8.

Possible packing models of **Au20**$^+$-BF_4^- in (a) Col$_h$ (Z = 1) and (b) Col$_r$ ($c2mm$) structures. The assemblies of **Au20**$^+$-BF_4^- were discussed in detail in the manuscript due to the ambiguous packing modes of **Au8**$^+$-BF_4^-, **Au12**$^+$-BF_4^-, and **Au16**$^+$-BF_4^-. Arrangement of the anions in the model structures of (a) and (b) is not exactly determined.

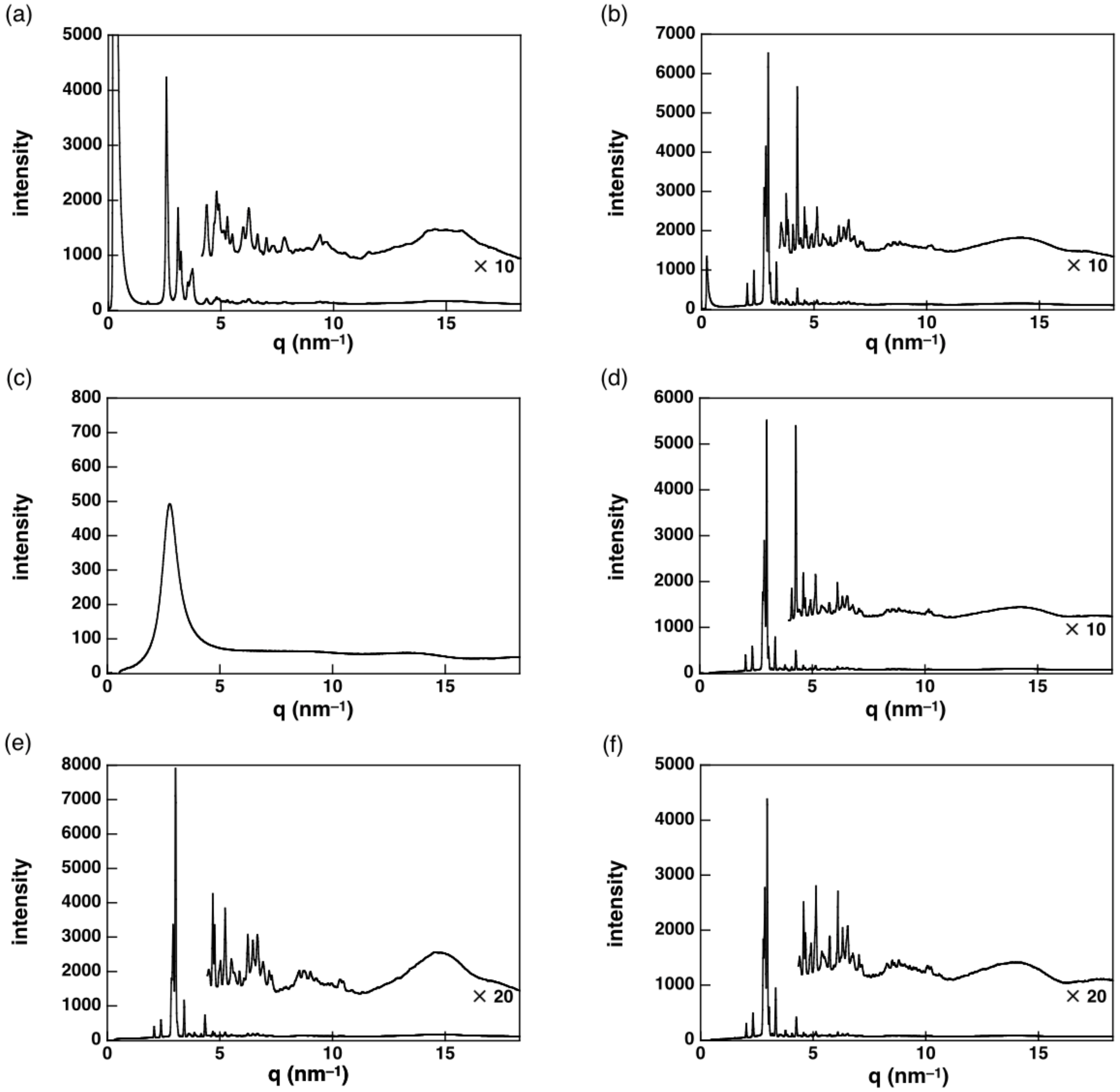

Figure S100 XRD patterns of Au8^{+}-PF$_6^{-}$, Related to Table 1.

XRD patterns of **Au8**$^{+}$-PF$_6^{-}$ at (a) 25 °C (1st heating), (b) 150 °C (1st heating), (c) 190 °C (1st heating), (d) 130 °C (1st cooling), (e) 25 °C (1st cooling), and (f) 150 °C (2nd heating). Complicated peak patters were obtained for (a,b,d–f), suggesting the highly crystalline states.

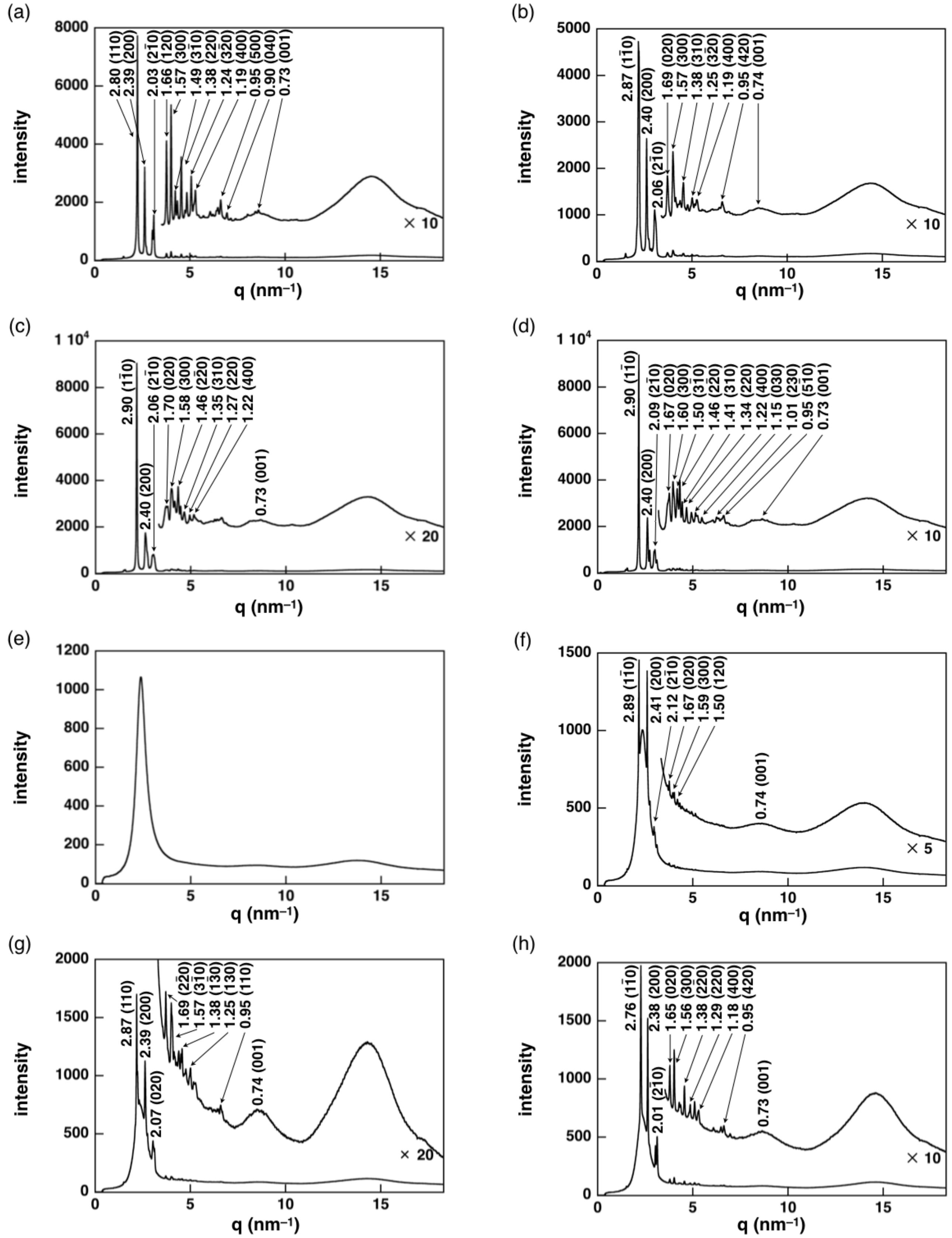

Figure 101 XRD patterns of Au12$^+$-PF_6^-, Related to Table 1.

XRD patterns of **Au12**$^+$-PF_6^- at (a) 25 °C (1st heating), (b) 60 °C (1st heating), (c) 80 °C (1st heating), (d) 100 °C (1st heating), (e) 130 °C (1st heating), (f) 100 °C (1st cooling), (g) 50 °C (1st cooling), and (h) 0 °C (1st cooling). The XRD patterns of (a–d,f–h) exhibit Col_{ob} structures (Figure S103).

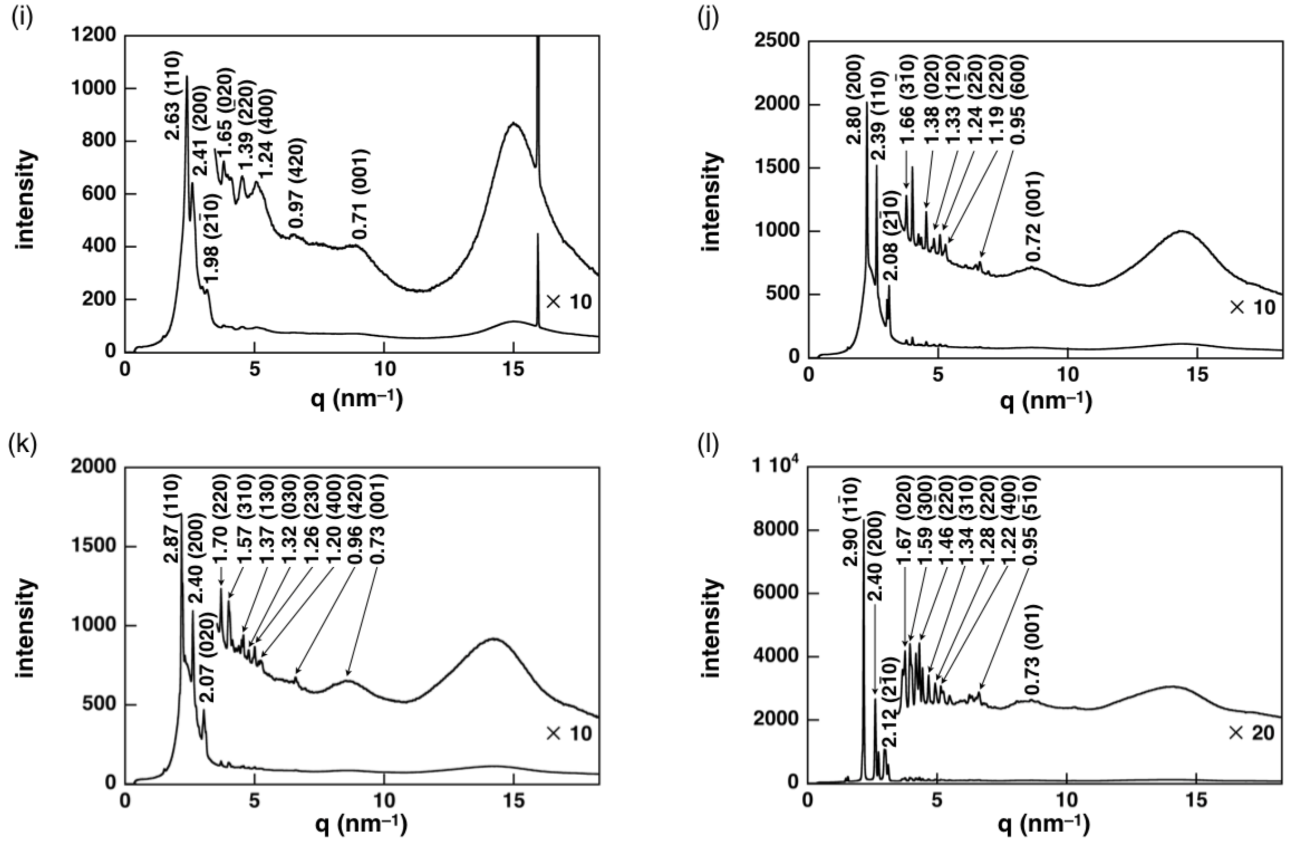

Figure 102 XRD patterns of Au12$^+$-PF$_6^-$, Related to Table 1.

XRD patterns of **Au12**$^+$-PF$_6^-$ at (i) –70 °C (1st cooling), (j) 25 °C (2nd heating), (k) 60 °C (2nd heating), and (l) 100 °C (2nd heating) (Figure labels are continued from Figure S101). The XRD patterns of (i–l) exhibit Col$_{ob}$ structures (Figure S103). XRD pattern at (i) –70 °C shows the sharp diffraction around 0.4 nm derived from frost.

Table S13 XRD peaks of Au12$^+$-PF$_6^-$, Related to Table 1.

XRD peaks of **Au12**$^+$-PF$_6^-$ at (a) 25 °C (1st heating), (b) 60 °C (1st heating), (c) 80 °C (1st heating), (d) 100 °C (1st heating), (f) 100 °C (1st cooling), (g) 50 °C (1st cooling), (h) 0 °C (1st cooling), (i) –70 °C (1st cooling), (j) 25 °C (2nd heating), (k) 60 °C (2nd heating), and (l) 100 °C (2nd heating) (Figure S101,102). The peaks which can be indexed are represented.

	q (nm^{-1})	*d*-spacing (nm)	ratio	ratio (calc.)	*hkl*
	2.24	2.80	1.00	1.000	110
	2.63	2.39	0.85	0.854	200
	3.10	2.03	0.72	0.741	$2\bar{1}0$
(a) **Au12**$^+$-PF$_6^-$	3.77	1.66	0.59	0.594	120
25 °C (1st heating)	4.01	1.57	0.56	0.570	300
Col$_{ob}$	4.22	1.49	0.53	0.537	$3\bar{1}0$
a = 4.80 nm, b = 3.66 nm,	4.55	1.38	0.49	0.500	220
c = 0.73 nm, γ = 94.4°	5.07	1.24	0.44	0.446	$3\bar{2}0$
M = 3166.54, Z = 2 for ρ = 0.82	5.28	1.19	0.42	0.427	400
	6.62	0.95	0.34	0.342	500
	6.95	0.90	0.32	0.326	040
	8.60	0.73	–	–	001

Table S13 (Continued)

	q (nm^{-1})	*d*-spacing (nm)	ratio	ratio (calc.)	*hkl*
(b) **Au12**$^+$-PF_6^-<br>60 °C (1st heating)<br>Col$_{ob}$<br>a = 4.83 nm, b = 3.36 nm,<br>c = 0.74 nm, γ = 95.3°<br>M = 3166.54, Z = 2 for ρ = 0.88	2.19	2.87	1.00	1.000	$1\bar{1}0$
	2.62	2.40	0.84	0.837	200
	3.04	2.06	0.72	0.711	$2\bar{1}0$
	3.72	1.69	0.59	0.582	020
	4.00	1.57	0.55	0.558	300
	4.55	1.38	0.48	0.486	310
	5.01	1.25	0.44	0.423	$3\bar{2}0$
	5.26	1.19	0.42	0.418	400
	6.59	0.95	0.33	0.326	420
	8.50	0.74	–	–	001
(c) **Au12**$^+$-PF_6^-<br>80 °C (1st heating)<br>Col$_{ob}$<br>a = 4.83 nm, b = 3.37 nm,<br>c = 0.73 nm , γ = 96.2°<br>M = 3166.54, Z = 2 for ρ = 0.89	2.17	2.90	1.00	1.000	$1\bar{1}0$
	2.62	2.40	0.83	0.828	200
	3.04	2.06	0.71	0.710	$2\bar{1}0$
	3.71	1.70	0.59	0.586	020
	3.99	1.58	0.54	0.545	300
	4.30	1.46	0.50	0.503	$2\bar{2}0$
	4.66	1.35	0.47	0.466	310
	4.94	1.27	0.44	0.438	220
	5.17	1.22	0.42	0.421	400
	8.66	0.73	–	–	001
(d) **Au12**$^+$-PF_6^-<br>100 °C (1st heating)<br>Col$_{ob}$<br>a = 4.83 nm, b = 3.38 nm,<br>c = 0.73 nm, γ = 95.3°<br>M = 3166.54, Z = 2 for ρ = 0.89	2.17	2.90	1.00	1.000	$1\bar{1}0$
	2.62	2.40	0.83	0.828	200
	3.01	2.09	0.72	0.710	$2\bar{1}0$
	3.76	1.67	0.58	0.580	020
	3.63	1.60	0.55	0.552	300
	4.18	1.50	0.52	0.520	$3\bar{1}0$
	4.31	1.46	0.50	0.500	$2\bar{2}0$
	4.45	1.41	0.49	0.479	310
	4.67	1.34	0.46	0.453	220
	5.15	1.22	0.42	0.414	400
	6.47	1.15	0.40	0.386	030
	6.25	1.01	0.35	0.337	230
	6.62	0.95	0.33	0.328	$5\bar{1}0$
	8.66	0.73	–	–	001
(f) **Au12**$^+$-PF_6^-<br>100 °C (1st cooling)<br>Col$_{ob}$<br>a = 4.86 nm, b = 3.31 nm,<br>c = 0.74 nm, γ = 97.1°<br>M = 3166.54, Z = 2 for ρ = 0.89	2.18	2.89	1.00	1.000	$1\bar{1}0$
	2.60	2.41	0.84	0.836	200
	2.96	2.12	0.73	0.717	$2\bar{1}0$
	3.76	1.67	0.58	0.569	020
	3.95	1.59	0.55	0.557	300
	4.19	1.50	0.52	0.519	120
	8.52	0.74	–	–	001
(g) **Au12**$^+$-PF_6^-<br>50 °C (1st cooling)<br>Col$_{ob}$<br>a = 4.85 nm, b = 4.11 nm,<br>c = 0.74 nm, γ = 99.3°<br>M = 3166.54, Z = 2 for ρ = 0.73	2.19	2.87	1.00	1.000	110
	2.63	2.39	0.83	0.833	200
	3.04	2.07	0.72	0.706	020
	3.72	1.69	0.59	0.587	$2\bar{2}0$
	4.00	1.57	0.55	0.548	$3\bar{1}0$
	4.56	1.38	0.48	0.473	$1\bar{3}0$
	5.01	1.25	0.44	0.435	130
	6.59	0.95	0.33	0.336	110
	8.51	0.74	–	–	001

Table S13 (Continued)

	q (nm^{-1})	*d*-spacing (nm)	ratio	ratio (calc.)	*hkl*
(h) **Au12**$^{+}$-PF_6^{-}<br>0 °C (1st cooling)<br>Col_{ob}<br>a = 4.76 nm, b = 3.30 nm,<br>c = 0.73 nm, γ = 92.2°<br>M = 3166.54, Z = 1 for ρ = 0.92	2.28	2.76	1.00	1.000	$1\bar{1}0$
	2.64	2.38	0.86	0.862	200
	3.13	2.01	0.73	0.712	$2\bar{1}0$
	3.80	1.65	0.60	0.597	020
	4.02	1.56	0.57	0.575	300
	4.56	1.38	0.50	0.500	$2\bar{2}0$
	4.87	1.29	0.47	0.482	220
	5.32	1.18	0.43	0.431	400
	6.63	0.95	0.34	0.343	420
	8.65	0.73	–	–	010
(i) **Au12**$^{+}$-PF_6^{-}<br>–70 °C (1st cooling)<br>Col_{ob}<br>a = 4.83 nm, b = 3.22 nm,<br>c = 0.71 nm, γ = 92.4°<br>M = 3166.54, Z = 2 for ρ = 0.96	2.39	2.63	1.00	1.000	110
	2.60	2.41	0.92	0.918	200
	3.17	1.98	0.75	0.750	$2\bar{1}0$
	3.82	1.65	0.63	0.613	020
	4.53	1.39	0.53	0.520	$2\bar{2}0$
	5.07	1.24	0.47	0.459	400
	6.49	0.97	0.37	0.360	420
	8.90	0.71	–	–	001
(j) **Au12**$^{+}$-PF_6^{-}<br>25 °C (2nd heating)<br>Col_{ob}<br>a = 5.62 nm, b = 2.77 nm,<br>c = 0.72 nm, γ = 95.2°<br>M = 3166.54, Z = 2 for ρ = 0.95	2.24	2.80	1.00	1.000	200
	2.63	2.39	0.85	0.854	110
	3.02	2.08	0.74	0.736	$2\bar{1}0$
	3.77	1.66	0.59	0.577	$3\bar{1}0$
	4.54	1.38	0.49	0.493	020
	4.74	1.33	0.47	0.469	120
	5.07	1.24	0.44	0.459	$2\bar{2}0$
	5.28	1.19	0.42	0.427	220
	6.60	0.95	0.34	0.333	600
	8.78	0.72	–	–	001
(k) **Au12**$^{+}$–PF_6^{-}<br>60 °C (2nd heating)<br>Col_{ob}<br>a = 4.87 nm, b = 4.11 nm,<br>c = 0.73 nm, γ = 99.6°<br>M = 3166.54, Z = 2 for ρ = 0.73	2.19	2.87	1.00	1.000	110
	2.62	2.40	0.84	0.837	200
	3.04	2.07	0.72	0.706	020
	3.71	1.70	0.59	0.590	220
	4.00	1.57	0.55	0.551	310
	4.57	1.37	0.48	0.474	130
	4.78	1.32	0.46	0.471	030
	5.00	1.26	0.44	0.443	230
	5.24	1.20	0.42	0.418	400
	6.58	0.96	0.33	0.336	420
	8.59	0.73	–	–	001
(l) **Au12**$^{+}$-PF_6^{-}<br>100 °C (2nd heating)<br>Col_{ob}<br>a = 4.85 nm, b = 3.31 nm,<br>c = 0.73 nm, γ = 98.0°<br>M = 3166.54, Z = 2 for ρ = 0.73	2.17	2.90	1.00	1.000	$1\bar{1}0$
	2.62	2.40	0.83	0.828	200
	2.96	2.12	0.73	0.717	$2\bar{1}0$
	3.76	1.67	0.58	0.565	020
	3.95	1.59	0.55	0.552	300
	4.31	1.46	0.50	0.500	$2\bar{2}0$
	4.67	1.34	0.46	0.471	310
	4.92	1.28	0.44	0.439	220
	5.14	1.22	0.42	0.414	400
	6.61	0.95	0.33	0.330	$5\bar{1}0$
	8.65	0.73	–	–	001

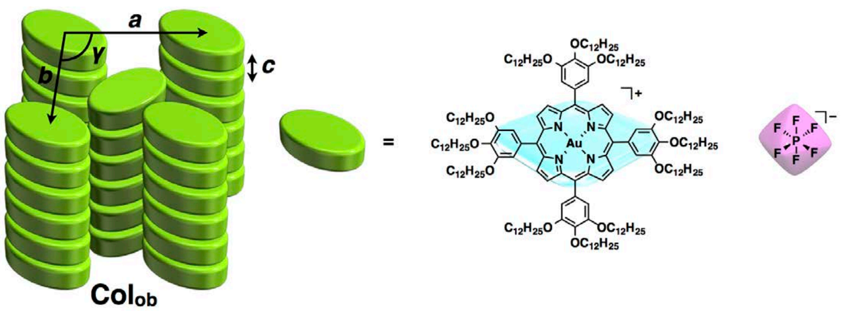

Figure S103 Possible packing models of Au12$^+$-PF_6^-, Related to Table 1.
Possible packing models of **Au12**$^+$-PF_6^- in an Col$_{ob}$ structure.

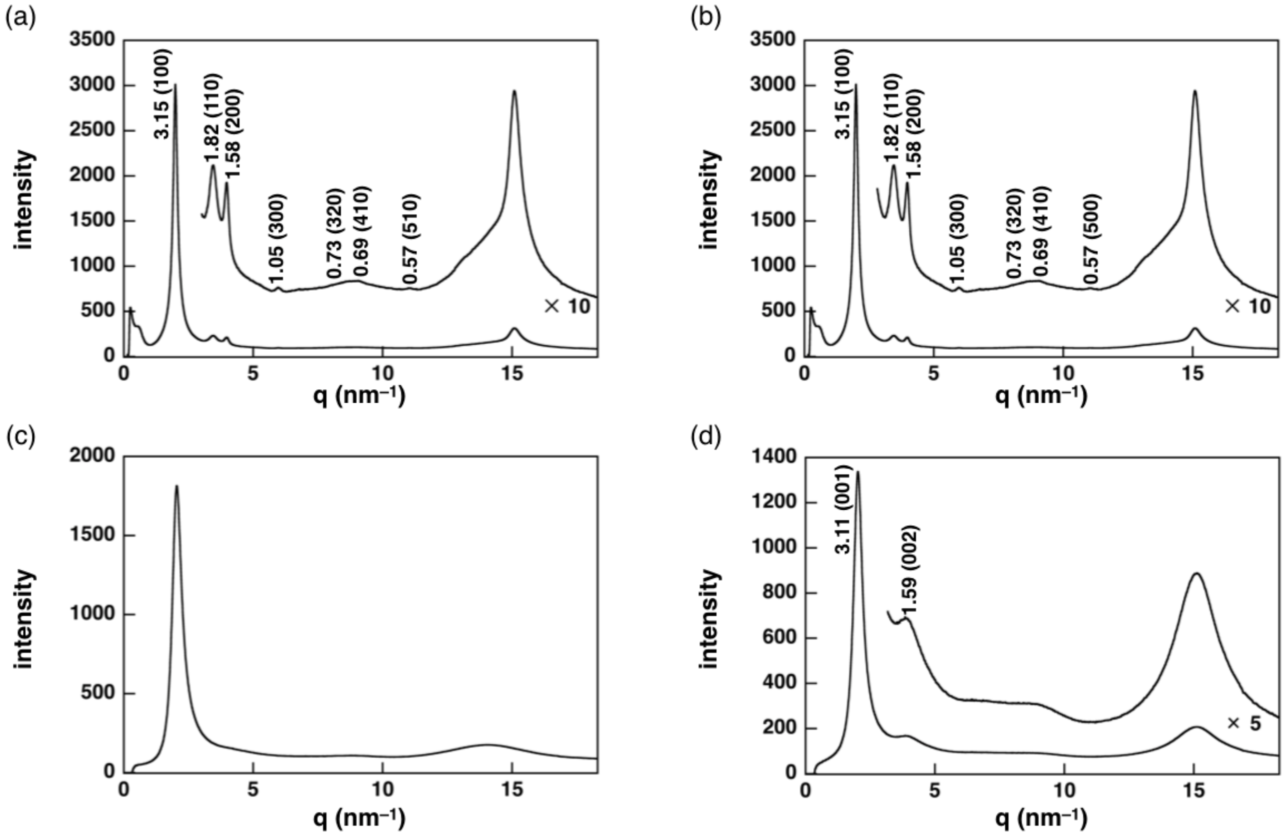

Figure S104 XRD patterns of Au16$^+$-PF_6^-, Related to Table 1.
XRD patterns of **Au16**$^+$-PF_6^- at (a) 25 °C (1st heating), (b) 31 °C (1st heating), (c) 60 °C (1st heating), and (d) –30 °C (1st cooling). The XRD patterns of (a,b) and (d) exhibit Col$_h$ and lamellar structures, respectively (Figure S105).

Table S14 XRD peaks of Au16$^+$-PF_6^-, Related to Table 1.
XRD peaks of **Au16**$^+$-PF_6^- at (a) 25 °C (1st heating), (b) 31 °C (1st heating), and (d) –30 °C (1st cooling) (Figure S104). The peaks which can be indexed are represented.

	q (nm^{-1})	*d*-spacing (nm)	ratio	ratio (calc.)	*hkl*
(a) **Au16**$^+$-PF_6^- 25 °C (1st heating) Col$_h$[a] a = 3.63 nm	2.00	3.15	1.00	1.000	100
	3.46	1.82	0.58	0.577	110
	3.98	1.58	0.50	0.500	200
	5.97	1.05	0.33	0.333	300
	8.61	0.73	0.23	0.229	320
	9.05	0.69	0.22	0.218	410
	11.06	0.57	0.18	0.180	510

Table S14 (Continued)

	q (nm⁻¹)	*d*-spacing (nm)	ratio	ratio (calc.)	*hkl*
(b) **Au16**$^+$-PF_6^- 31 °C (1st heating) Col_h[a] *a* = 3.63 nm	2.00	3.15	1.00	1.000	100
	3.46	1.82	0.58	0.577	110
	3.98	1.58	0.50	0.500	200
	5.97	1.05	0.33	0.333	300
	8.60	0.73	0.23	0.229	320
	9.08	0.69	0.22	0.218	410
	10.99	0.57	0.18	0.180	510
(d) **Au16**$^+$-PF_6^- –30 °C (1st cooling) lamellar	2.02	3.11	1.00	1.000	001
	3.96	1.59	0.51	0.500	002

[a] *Z* and ρ values are not given due to the unclear height value (*c*) in XRD chart.

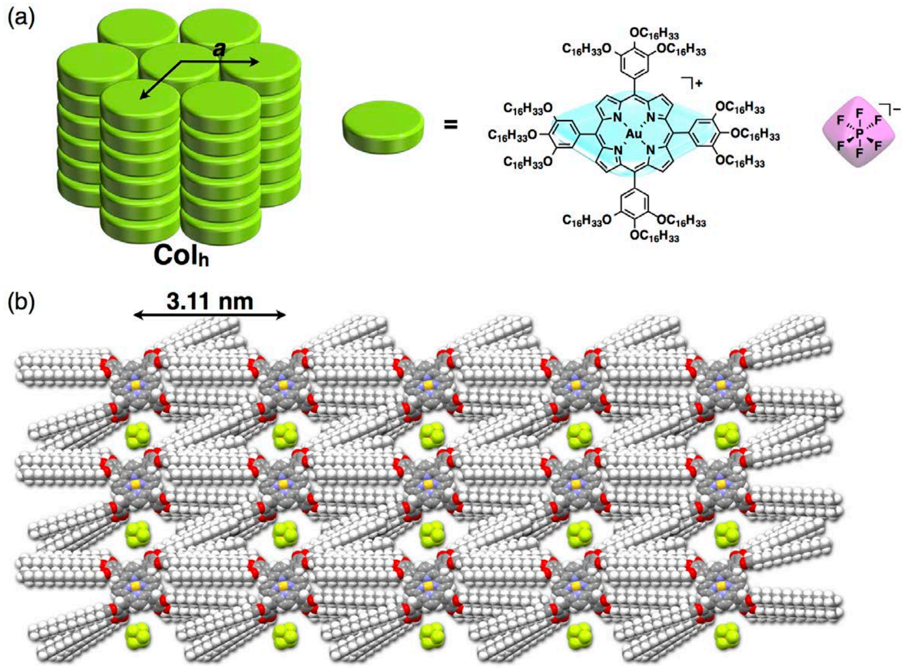

Figure S105 Possible packing models of Au16$^+$-PF_6^-, Related to Table 1.
Possible packing models of **Au16**$^+$-PF_6^- in (a) Col_h and (b) lamellar structures.

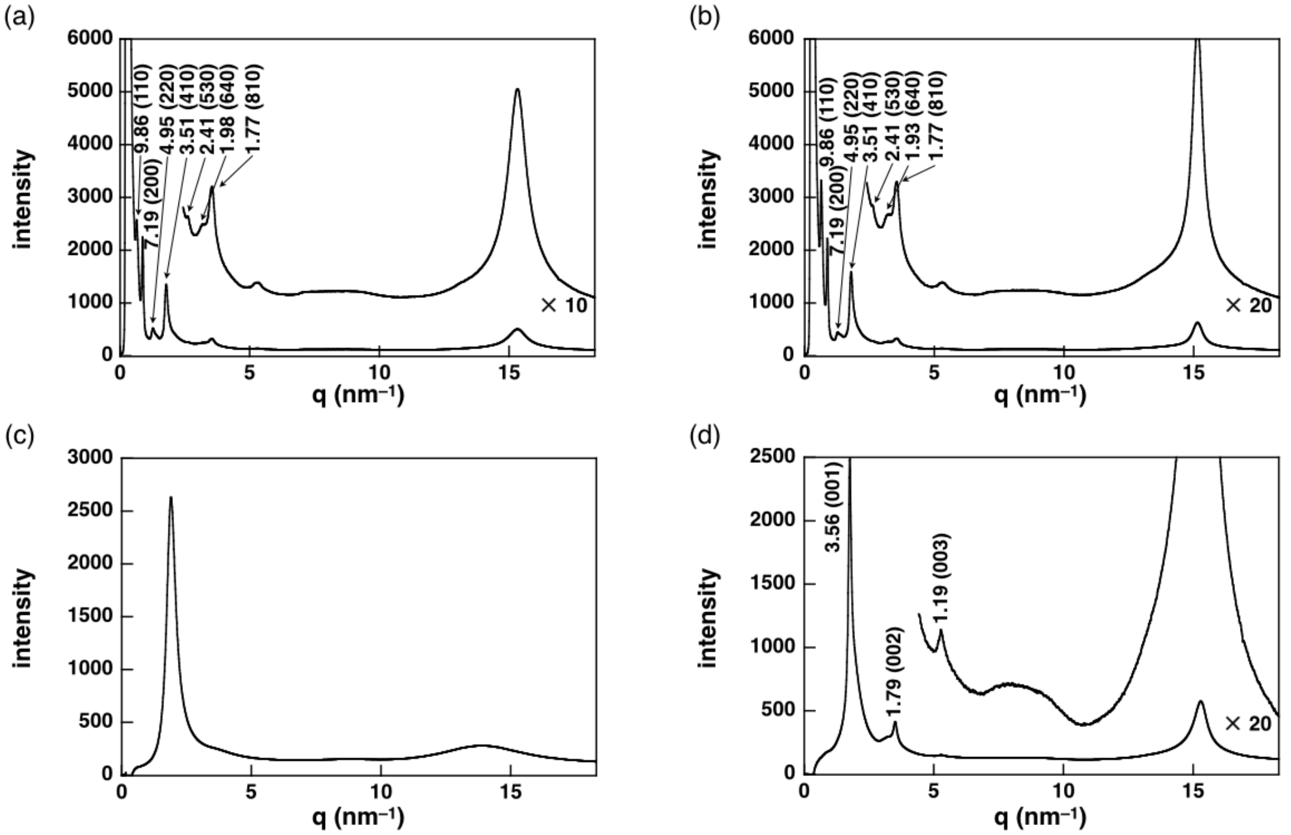

Figure S106 XRD patterns of Au20$^+$-PF$_6^-$, Related to Table 1.

XRD patterns of **Au20**$^+$-PF$_6^-$ at (a) 25 °C (1st heating), (b) 52 °C (1st heating), (c) 80 °C (1st heating), and (d) 25 °C (1st cooling). The XRD patterns of (a,b) and (d) exhibit Col$_r$ and lamellar structures, respectively (Figure S107).

Table S15 XRD peaks of Au20⁺-PF₆⁻, Related to Table 1.

XRD peaks of **Au20**$^{+}$-PF_6^{-} at (a) 25 °C (1st heating), (b) 52 °C (1st heating), and (d) 25 °C (1st cooling) (Figure S106). The peaks which can be indexed are represented.

	q (nm^{-1})	*d*-spacing (nm)	ratio	ratio (calc.)	*hkl*
(a) **Au20**$^{+}$-PF_6^{-} 25 °C (1st heating) Col_r[a] *a* = 14.37 nm, *b* = 13.54 nm	0.64	9.86	1.00	1.000	110
	0.87	7.19	0.73	0.729	200
	1.27	4.95	0.50	0.500	220
	1.79	3.51	0.36	0.352	410
	2.61	2.41	0.24	0.246	530
	3.18	1.98	0.20	0.198	640
	3.55	1.77	0.18	0.181	810
(b) **Au20**$^{+}$-PF_6^{-} 52 °C (1st heating) Col_r[a] *a* = 14.37 nm, *b* = 13.54 nm	0.64	9.86	1.00	1.000	110
	0.87	7.19	0.73	0.729	200
	1.27	4.95	0.50	0.500	220
	1.79	3.51	0.36	0.352	410
	2.61	2.41	0.24	0.246	530
	3.25	1.93	0.20	0.198	640
	3.55	1.77	0.18	0.181	810
(d) **Au20**$^{+}$-PF_6^{-} 25 °C (1st cooling) lamellar	1.77	3.56	1.00	1.000	001
	3.51	1.79	0.50	0.500	002
	5.27	1.19	0.33	0.333	003

[a] *Z* and ρ values are not given due to the unclear height value (*c*) in XRD chart.

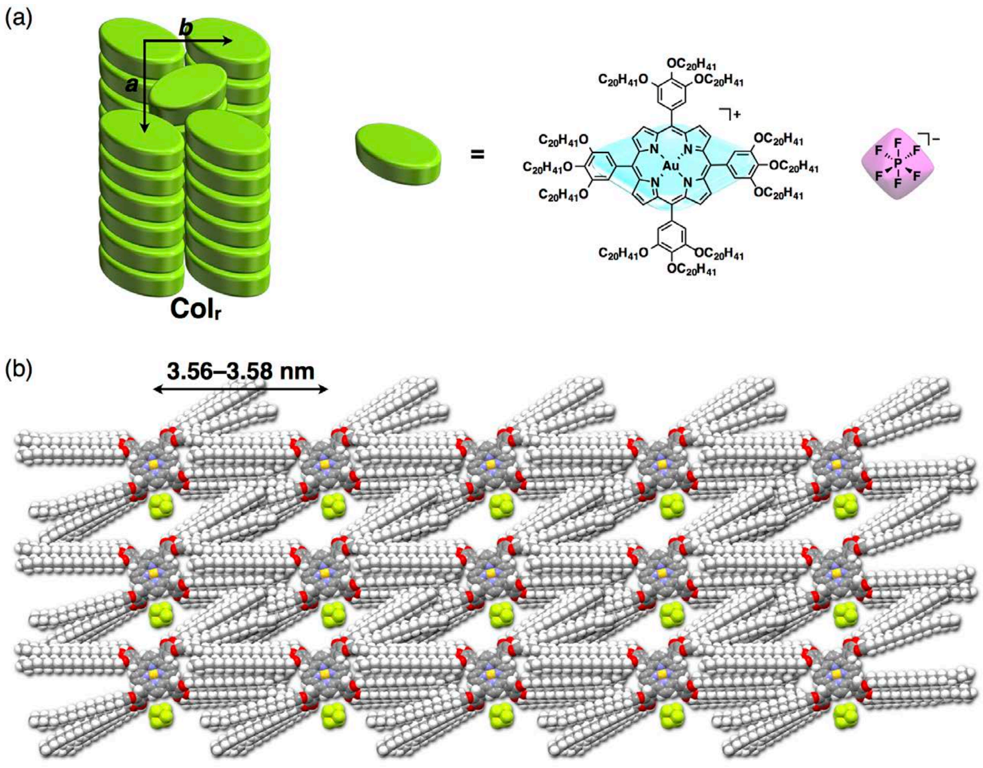

Figure S107 Possible packing models of Au20⁺-PF₆⁻, Related to Table 1.

Possible packing models of **Au20**$^{+}$-PF_6^{-} in (a) Col_r and (b) lamellar structures.

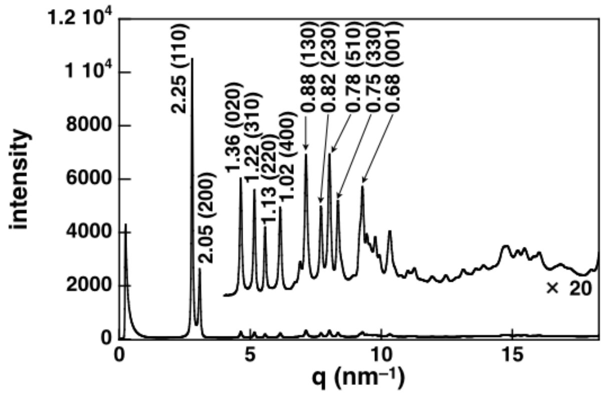

Figure S108 XRD patterns of Au8$^+$-PCCp$^-$, Related to Table 1.
XRD patterns of **Au8**$^+$-PCCp$^-$ at 25 °C (1st heating). The XRD pattern exhibits a Col$_r$ structure (Figure S109).

Table S16 XRD peaks of Au8$^+$-PCCp$^-$, Related to Table 1.
XRD peaks of **Au8**$^+$-PCCp$^-$ at 25 °C (1st heating) (Figure S108). The peaks which can be indexed are represented.

	q (nm^{-1})	*d*-spacing (nm)	ratio	ratio (calc.)	*hkl*
**Au8**$^+$-PCCp$^-$<br>25 °C (1st heating)<br>Col$_r$ (*p*2*gg*)<br>*a* = 4.10 nm, *b* = 2.70 nm,<br>*c* = 0.68 nm<br>*M* = 2538.43, *Z* = 2 for *ρ* = 1.17	2.79	2.25	1.00	1.000	110
	3.07	2.05	0.91	0.908	200
	4.63	1.36	0.60	0.599	020
	5.16	1.22	0.54	0.540	310
	5.57	1.13	0.50	0.500	220
	6.14	1.02	0.45	0.454	400
	7.12	0.88	0.39	0.390	130
	7.70	0.82	0.36	0.366	230
	8.02	0.78	0.35	0.348	510
	8.35	0.75	0.33	0.333	330
	9.19	0.68	–	–	001

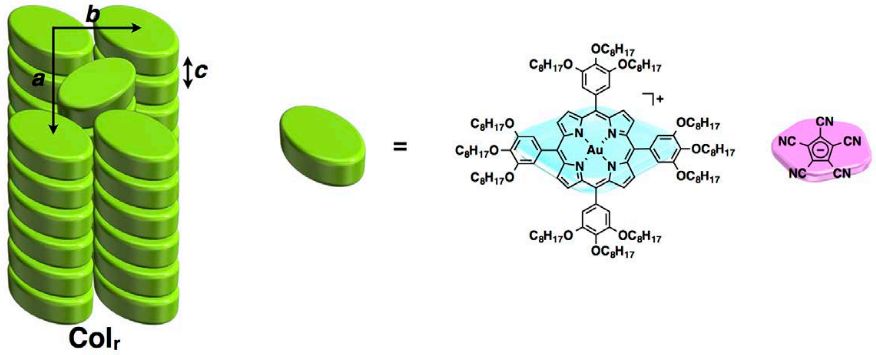

Figure S109 Possible packing model of Au8$^+$-PCCp$^-$, Related to Table 1.
Possible packing model of **Au8**$^+$-PCCp$^-$ in a Col$_r$ (*p*2*gg*) structure.

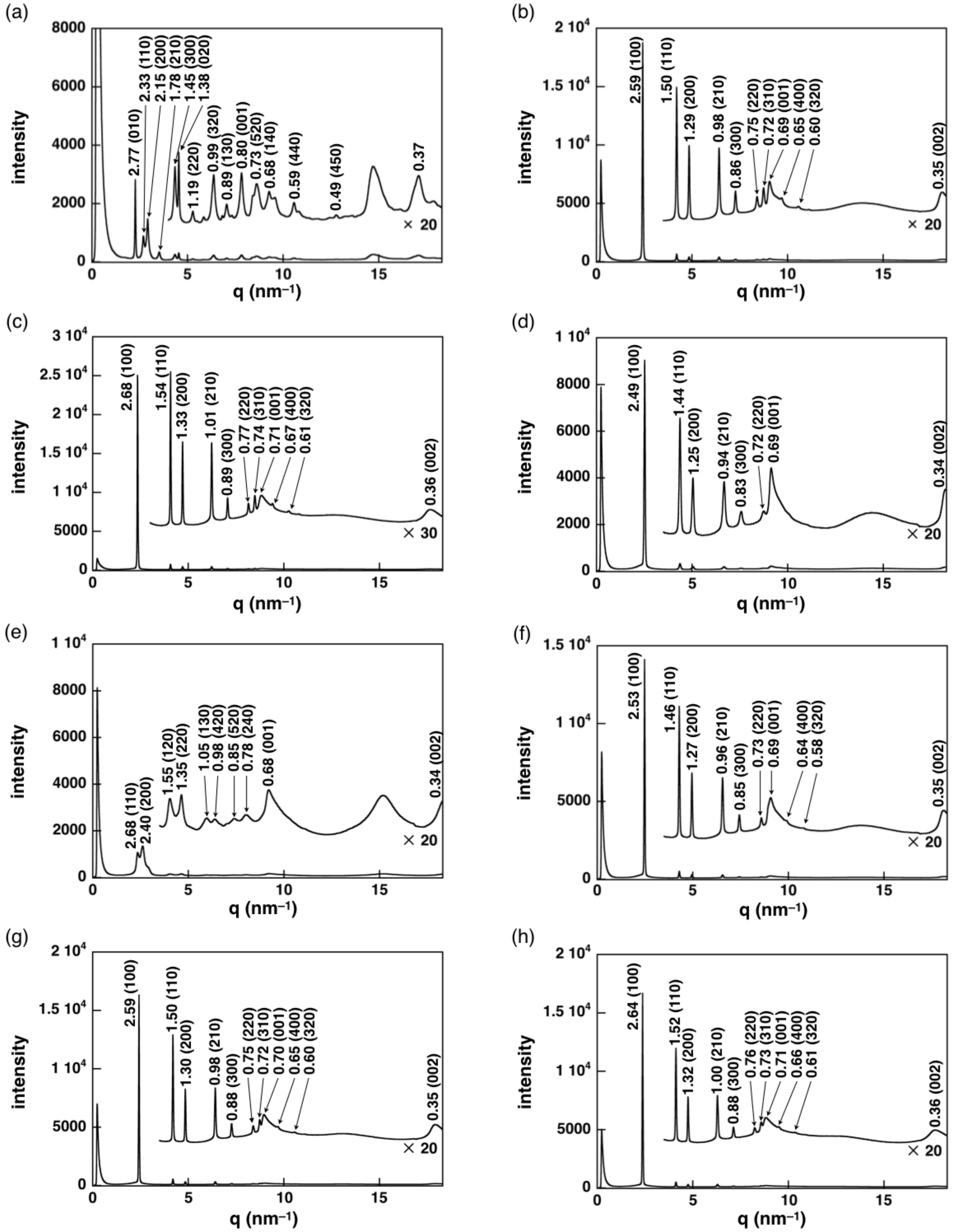

Figure S110 XRD patterns of Au12⁺-PCCp⁻, Related to Table 1.

XRD patterns of **Au12**⁺-PCCp⁻ at (a) 25 °C (1st heating), (b) 100 °C (1st heating), (c) 300 °C (1st heating), (d) 25 °C (1st cooling), (e) –20 °C (1st cooling), (f) 100 °C (2nd heating), (g) 200 °C (2nd heating), and (h) 300 °C (2nd heating). The XRD patterns of (a,e) and (b–d,f–h) exhibit Col_r and Col_h structures, respectively (Figure S111).

Table S17 XRD peaks of Au12$^+$-PCCp$^-$, Related to Table 1.

XRD peaks of **Au12**$^+$-PCCp$^-$ at (a) 25 °C (1st heating), (b) 100 °C (1st heating), (c) 300 °C (1st heating), (d) 25 °C (1st cooling), (e) –20 °C (1st cooling), (f) 100 °C (2nd heating), (g) 200 °C (2nd heating), and (h) 300 °C (2nd heating) (Figure S110). The peaks which can be indexed are represented.[a]

	q (nm^{-1})	*d*-spacing (nm)	ratio	ratio (calc.)	*hkl*
	2.27	2.77	1.00	1.000	010
	2.69	2.33	0.84	0.841	110
	2.92	2.15	0.78	0.778	200
	3.53	1.78	0.64	0.614	210
(a) **Au12**$^+$-PCCp$^-$	4.35	1.45	0.52	0.519	300
25 °C (1st heating)	4.54	1.38	0.50	0.500	020
Col$_r$	5.28	1.19	0.43	0.421	220
a = 4.31 nm, b = 2.77 nm,	6.37	0.99	0.36	0.360	320
c = 0.80 nm	7.05	0.89	0.32	0.326	130
M = 3211.72, Z = 2 for ρ = 1.10	7.82	0.80	–	–	001
	8.61	0.73	0.26	0.264	520
	9.26	0.68	0.24	0.247	140
	10.57	0.59	0.21	0.210	440
	12.80	0.49	0.18	0.178	450
	2.42	2.59	1.00	1.000	100
	4.20	1.50	0.58	0.577	110
	4.85	1.29	0.50	0.500	200
(b) **Au12**$^+$-PCCp$^-$	6.42	0.98	0.38	0.378	210
100 °C (1st heating)	7.27	0.86	0.33	0.333	300
Col$_h$	8.41	0.75	0.29	0.289	220
a = 2.99 nm, c = 0.69 nm	8.75	0.72	0.28	0.277	310
M = 3211.72, Z = 1 for ρ = 0.99	9.05	0.69	–	–	001
	9.68	0.65	0.25	0.250	400
	10.56	0.60	0.23	0.229	320
	18.10	0.35	–	–	002
	2.35	2.68	1.00	1.000	100
	4.08	1.54	0.58	0.577	110
	4.71	1.33	0.50	0.500	200
(c) **Au12**$^+$-PCCp$^-$	6.23	1.01	0.38	0.378	210
300 °C (1st heating)	7.06	0.89	0.33	0.333	300
Col$_h$	8.15	0.77	0.29	0.289	220
a = 3.09 nm, c = 0.71 nm	8.48	0.74	0.28	0.277	310
M = 3211.72, Z = 1 for ρ = 0.91	8.84	0.71	–	–	001
	9.40	0.67	0.25	0.250	400
	10.24	0.61	0.23	0.229	320
	17.68	0.36	–	–	002
	2.53	2.49	1.00	1.000	100
(d) **Au12**$^+$-PCCp$^-$	4.37	1.44	0.58	0.577	110
25 °C (1st cooling)	5.05	1.25	0.50	0.500	200
Col$_h$	6.68	0.94	0.38	0.378	210
a = 2.87 nm, c = 0.69 nm	7.58	0.83	0.33	0.333	300
M = 3211.72, Z = 1 for ρ = 1.09	8.76	0.72	0.29	0.289	220
	9.14	0.69	–	–	001
	18.25	0.34	–	–	002

Table S17 (Continued)

	q (nm^{-1})	*d*-spacing (nm)	ratio	ratio (calc.)	*hkl*
(e) **Au12**$^{+}$-PCCp^{-} –20 °C (1st cooling) Col$_r$ a = 4.80 nm, b = 3.23 nm, c = 0.68 nm M = 3211.72, Z = 2 for ρ = 1.01	2.35	2.68	1.00	1.000	110
	2.62	2.40	0.90	0.897	200
	4.04	1.55	0.58	0.571	120
	4.64	1.35	0.51	0.500	220
	5.97	1.05	0.39	0.392	130
	6.40	0.98	0.37	0.360	420
	7.40	0.85	0.32	0.308	520
	8.04	0.78	0.29	0.285	240
	9.21	0.68	–	–	001
(f) **Au12**$^{+}$-PCCp^{-} 100 °C (1st cooling) Col$_h$ a = 2.92 nm, c = 0.69 nm M = 3211.72, Z = 1 for ρ = 1.04	2.48	2.53	1.00	1.000	100
	4.29	1.46	0.58	0.577	110
	4.96	1.27	0.50	0.500	200
	6.55	0.96	0.38	0.378	210
	7.43	0.85	0.33	0.333	300
	8.59	0.73	0.29	0.289	220
	9.08	0.69	–	–	001
	9.89	0.64	0.25	0.250	400
	10.77	0.58	0.23	0.229	320
	18.10	0.35	–	–	002
(g) **Au12**$^{+}$-PCCp^{-} 200 °C (1st cooling) Col$_h$ a = 2.99 nm, c = 0.70 nm M = 3211.72, Z = 1 for ρ = 0.98	2.42	2.59	1.00	1.000	100
	4.20	1.50	0.58	0.577	110
	4.84	1.30	0.50	0.500	200
	6.41	0.98	0.38	0.378	210
	7.27	0.88	0.33	0.333	300
	8.40	0.75	0.29	0.289	220
	8.74	0.72	0.28	0.277	310
	8.96	0.70	–	–	001
	9.67	0.65	0.25	0.250	400
	10.56	0.60	0.23	0.229	320
	17.92	0.35	–	–	002
(h) **Au12**$^{+}$-PCCp^{-} 300 °C (1st cooling) Col$_h$ a = 3.05 nm, c = 0.71 nm M = 3211.72, Z = 1 for ρ = 0.93	2.38	2.64	1.00	1.000	100
	4.12	1.52	0.58	0.577	110
	4.75	1.32	0.50	0.500	200
	6.29	1.00	0.38	0.378	210
	7.13	0.88	0.33	0.333	300
	8.24	0.76	0.29	0.289	220
	8.58	0.73	0.28	0.277	310
	8.84	0.71	–	–	001
	9.48	0.66	0.25	0.250	400
	10.33	0.61	0.23	0.229	320
	17.68	0.36	–	–	002

[a] The diffraction peaks which corresponded to 002, 003, and 004 were observed at the wide-angle region (Figure S112).

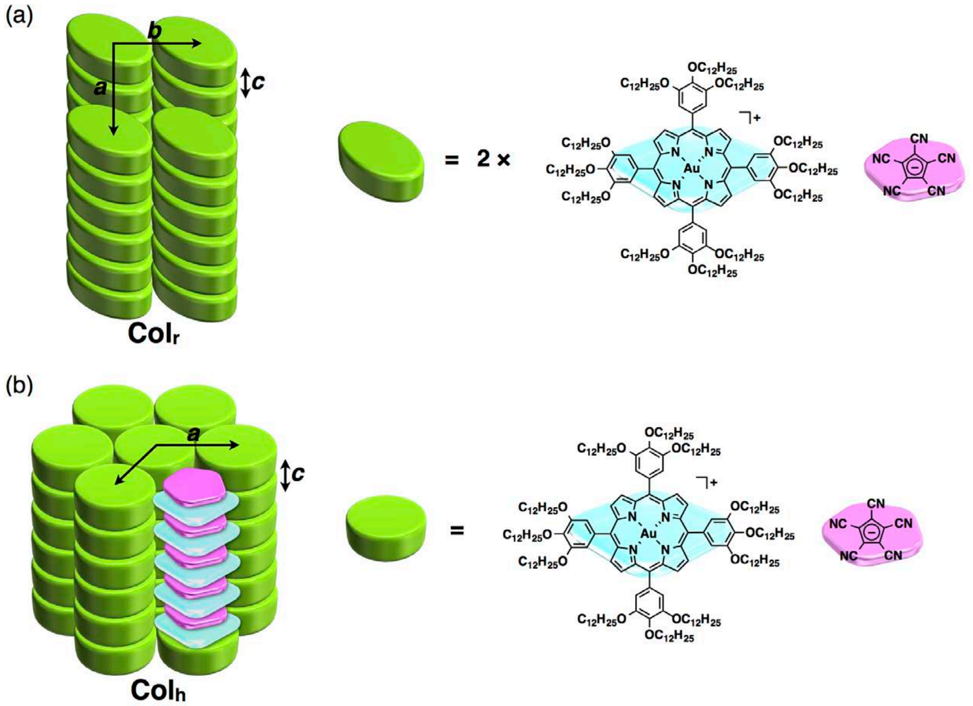

Figure S111 Possible packing models of Au12⁺-PCCp⁻, Related to Table 1.
Possible packing models of **Au12**$^{+}$-PCCp^{-} in (a) Col$_{r}$ and (b) Col$_{h}$ structures.

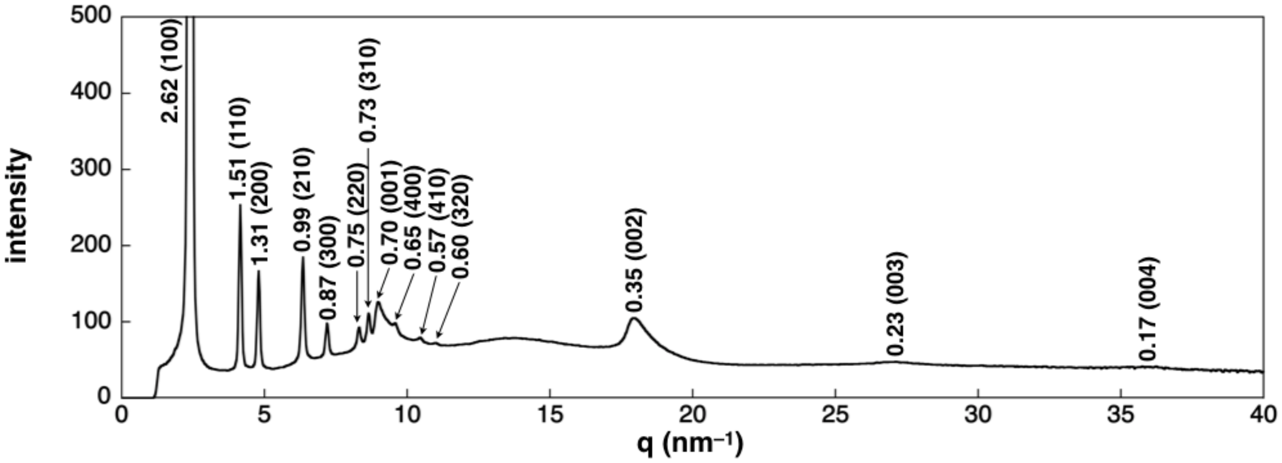

Figure S112 Wide-angle XRD of Au12⁺-PCCp⁻, Related to Table 1.
Wide-angle XRD of **Au12**$^{+}$-PCCp^{-} at 200 °C (1st cooling). The diffraction peak at 0.70 nm is derived from the alternate stacking of porphyrin–Au^{III} complex and PCCp^{-} in the Col$_{h}$ packing structure (Table S17 and Figure S110,111). Highly ordered charge-by-charge assembly affords higher-order diffractions derived from (001) peak.

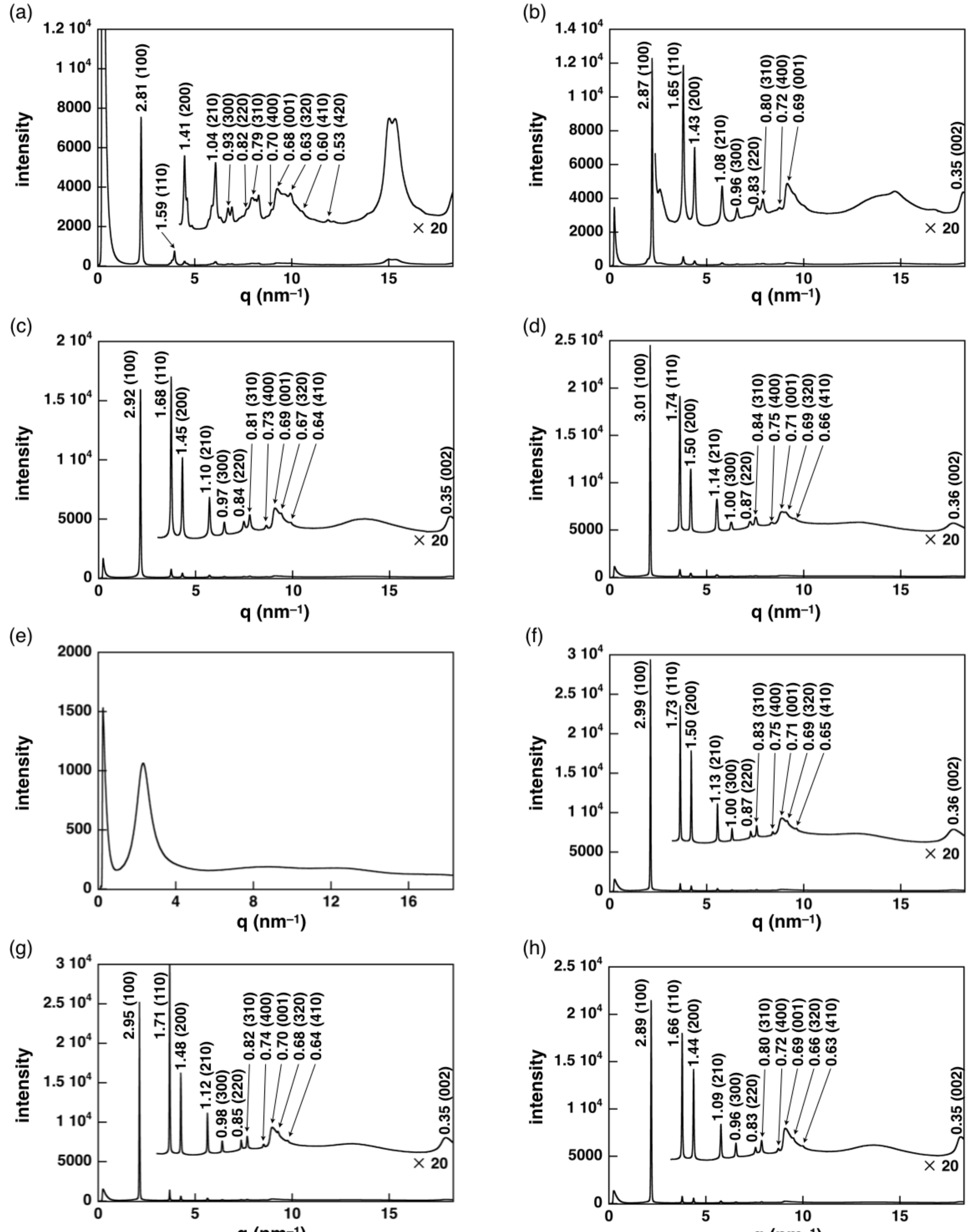

Figure S113 XRD patterns of Au16^{+}-PCCp^{-}, Related to Table 1 and Figure 8.

XRD patterns of **Au16**$^{+}$-PCCp^{-} at (a) 25 °C (1st heating), (b) 48 °C (1st heating), (c) 100 °C (1st heating), (d) 280 °C (1st heating), (e) 330 °C (1st heating), (f) 280 °C (1st cooling), (g) 200 °C (1st cooling), and (h) 100 °C (1st cooling). The XRD patterns of (a–d,f–h) exhibit Col$_{h}$ structures (Figure S115).

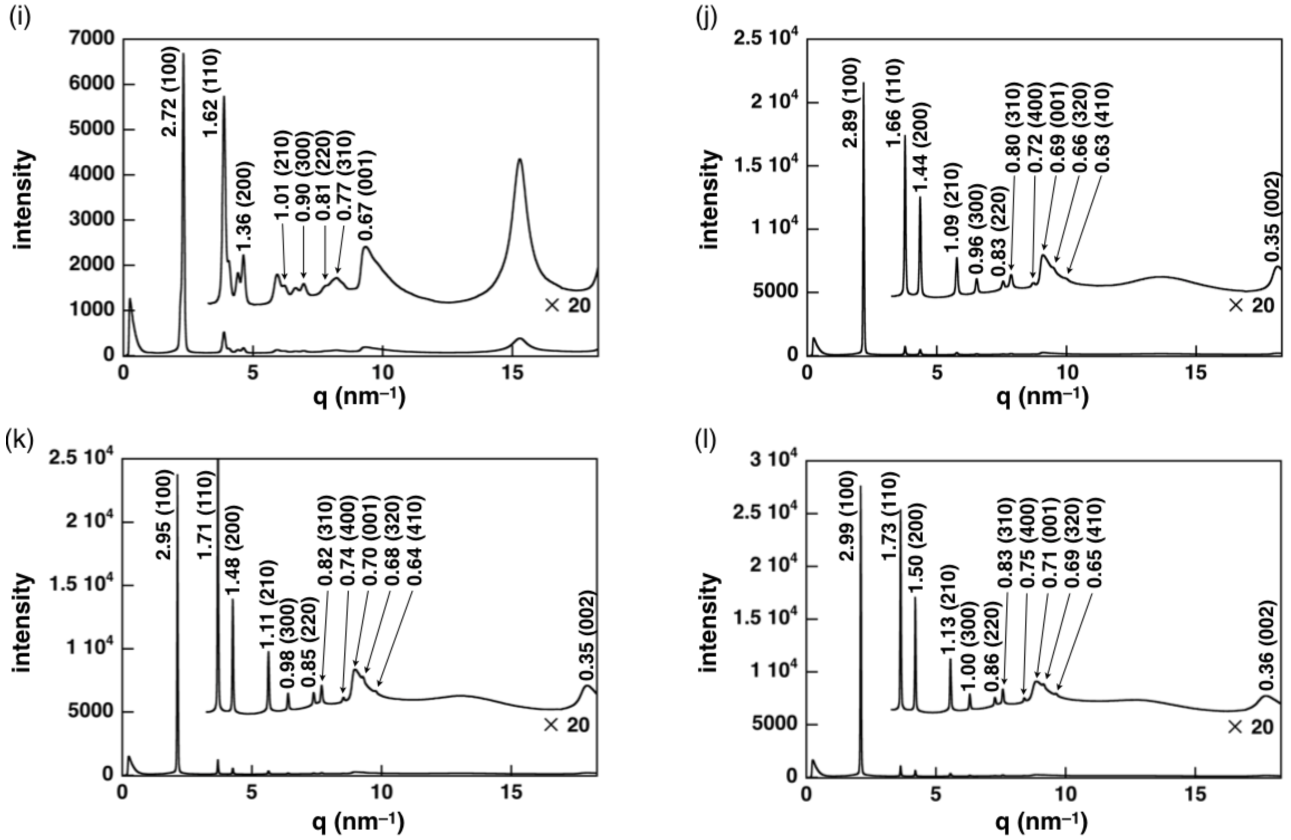

Figure S114 XRD patterns of Au16⁺-PCCp⁻, Related to Table 1 and Figure 8.
XRD patterns of **Au16**⁺-PCCp⁻ at (i) 0 °C (1st cooling), (j) 100 °C (2nd heating), (k) 200 °C (2nd heating), and (l) 280 °C (2nd heating) (Figure labels are continued from Figure S113). The XRD patterns of (i–l) exhibit Col_h structures (Figure S115).

Table S18 XRD peaks of Au16⁺-PCCp⁻, Related to Table 1 and Figure 8.
XRD peaks of **Au16**⁺-PCCp⁻ at (a) 25 °C (1st heating), (b) 48 °C (1st heating), (c) 100 °C (1st heating), (d) 280 °C (1st heating), (f) 280 °C (1st cooling), (g) 200 °C (1st cooling), (h) 100 °C (1st cooling), (i) 0 °C (1st cooling), (j) 100 °C (2nd heating), (k) 200 °C (2nd heating), and (l) 280 °C (2nd heating) (Figure S113,114). The peaks which can be indexed are represented.[a]

	q (nm⁻¹)	*d*-spacing (nm)	ratio	ratio (calc.)	*hkl*
(a) **Au16**⁺-PCCp⁻<br>25 °C (1st heating)<br>Col_h<br>a = 3.25 nm, c = 0.68 nm<br>M = 3885.02, Z = 1 for ρ = 1.04	2.23	2.81	1.00	1.000	100
	3.95	1.59	0.56	0.577	110
	4.47	1.41	0.50	0.500	200
	6.07	1.04	0.37	0.378	210
	6.72	0.93	0.33	0.333	300
	7.68	0.82	0.29	0.289	220
	7.97	0.79	0.28	0.277	310
	8.97	0.70	0.25	0.250	400
	9.26	0.68	–	–	001
	9.93	0.63	0.22	0.229	320
	10.48	0.60	0.21	0.218	410
	11.86	0.53	0.19	0.189	420

Table S18 (Continued)

	q (nm⁻¹)	*d*-spacing (nm)	ratio	ratio (calc.)	*hkl*
(b) **Au16**$^{+}$-PCCp^{-}<br>48 °C (1st heating)<br>Col$_h$<br>a = 3.32 nm, c = 0.69 nm<br>M = 3885.02, Z = 1 for ρ = 0.99	2.19	2.87	1.00	1.000	100
	3.80	1.65	0.58	0.577	110
	4.38	1.43	0.50	0.500	200
	5.80	1.08	0.38	0.378	210
	6.56	0.96	0.33	0.333	300
	7.59	0.83	0.29	0.289	220
	7.89	0.80	0.28	0.277	310
	8.75	0.72	0.25	0.250	400
	9.15	0.69	–	–	001
	18.22	0.35	–	–	002
(c) **Au16**$^{+}$-PCCp^{-}<br>100 °C (1st heating)<br>Col$_h$<br>a = 3.37 nm, c = 0.69 nm<br>M = 3885.02, Z = 1 for ρ = 0.95	2.15	2.92	1.00	1.000	100
	3.74	1.68	0.58	0.577	110
	4.33	1.45	0.50	0.500	200
	5.72	1.10	0.38	0.378	210
	6.49	0.97	0.33	0.333	300
	7.49	0.84	0.29	0.289	220
	7.79	0.81	0.28	0.277	310
	8.65	0.73	0.25	0.250	400
	9.10	0.69	–	–	001
	9.39	0.67	0.23	0.229	320
	9.87	0.64	0.22	0.218	410
	18.14	0.35	–	–	002
(d) **Au16**$^{+}$-PCCp^{-}<br>280 °C (1st heating)<br>Col$_h$<br>a = 3.48 nm, c = 0.71 nm<br>M = 3885.02, Z = 1 for ρ = 0.87	2.09	3.01	1.00	1.000	100
	3.62	1.74	0.58	0.577	110
	4.18	1.50	0.50	0.500	200
	5.53	1.14	0.38	0.378	210
	6.26	1.00	0.33	0.333	300
	7.23	0.87	0.29	0.289	220
	7.52	0.84	0.28	0.277	310
	8.34	0.75	0.25	0.250	400
	8.89	0.71	–	–	001
	9.06	0.69	0.23	0.229	320
	9.51	0.66	0.22	0.218	410
	17.71	0.36	–	–	002
(f) **Au16**$^{+}$-PCCp^{-}<br>280 °C (1st cooling)<br>Col$_h$<br>a = 3.46 nm, c = 0.71 nm<br>M = 3885.02, Z = 1 for ρ = 0.88	2.10	2.99	1.00	1.000	100
	3.64	1.73	0.58	0.577	110
	4.20	1.50	0.50	0.500	200
	5.56	1.13	0.38	0.378	210
	6.31	1.00	0.33	0.333	300
	7.26	0.87	0.29	0.289	220
	7.58	0.83	0.28	0.277	310
	8.41	0.75	0.25	0.250	400
	8.88	0.71	–	–	001
	9.13	0.69	0.23	0.229	320
	9.62	0.65	0.22	0.218	410
	17.71	0.36	–	–	002

Table S18 (Continued)

	q (nm⁻¹)	*d*-spacing (nm)	ratio	ratio (calc.)	*hkl*
(g) **Au16**$^+$-PCCp$^-$<br>200 °C (1st cooling)<br>Col_h<br>a = 3.40 nm, c = 0.70 nm<br>M = 3885.02, Z = 1 for ρ = 0.92	2.13	2.95	1.00	1.000	100
	3.68	1.71	0.58	0.577	110
	4.26	1.48	0.50	0.500	200
	5.63	1.12	0.38	0.378	210
	6.40	0.98	0.33	0.333	300
	7.37	0.85	0.29	0.289	220
	7.68	0.82	0.28	0.277	310
	8.53	0.74	0.25	0.250	400
	8.97	0.70	–	–	001
	9.26	0.68	0.23	0.229	320
	9.76	0.64	0.22	0.218	410
	17.93	0.35	–	–	002
(h) **Au16**$^+$-PCCp$^-$<br>100 °C (1st cooling)<br>Col_h<br>a = 3.33 nm, c = 0.69 nm<br>M = 3885.02, Z = 1 for ρ = 0.97	2.18	2.89	1.00	1.000	100
	3.77	1.66	0.58	0.577	110
	4.37	1.44	0.50	0.500	200
	5.78	1.09	0.38	0.378	210
	6.55	0.96	0.33	0.333	300
	7.57	0.83	0.29	0.289	220
	7.87	0.80	0.28	0.277	310
	8.74	0.72	0.25	0.250	400
	9.10	0.69	–	–	001
	9.48	0.66	0.23	0.229	320
	10.00	0.63	0.22	0.218	410
	18.13	0.35	–	–	002
(i) **Au16**$^+$-PCCp$^-$<br>0 °C (1st cooling)<br>Col_h<br>a = 3.14 nm, c = 0.67 nm<br>M = 3885.02, Z = 1 for ρ = 1.12	2.31	2.72	1.00	1.000	100
	3.88	1.62	0.60	0.577	110
	4.63	1.36	0.50	0.500	200
	6.22	1.01	0.37	0.378	210
	6.96	0.90	0.33	0.333	300
	7.80	0.81	0.30	0.289	220
	8.22	0.77	0.28	0.277	310
(j) **Au16**$^+$-PCCp$^-$<br>100 °C (2nd heating)<br>Col_h<br>a = 3.33 nm, c = 0.69 nm<br>M = 3885.02, Z = 1 for ρ = 0.97	2.18	2.89	1.00	1.000	100
	3.77	1.66	0.58	0.577	110
	4.36	1.44	0.50	0.500	200
	5.78	1.09	0.38	0.378	210
	6.54	0.96	0.33	0.333	300
	7.55	0.83	0.29	0.289	220
	7.87	0.80	0.28	0.277	310
	8.74	0.72	0.25	0.250	400
	9.08	0.69	–	–	001
	9.47	0.66	0.23	0.229	320
	9.94	0.63	0.22	0.218	410
	18.14	0.35	–	–	002
(k) **Au16**$^+$-PCCp$^-$<br>200 °C (2nd heating)<br>Col_h<br>a = 3.40 nm, c = 0.70 nm<br>M = 3885.02, Z = 1 for ρ = 0.92	2.13	2.95	1.00	1.000	100
	3.68	1.71	0.58	0.577	110
	4.26	1.48	0.50	0.500	200
	5.64	1.11	0.38	0.378	210
	6.40	0.98	0.33	0.333	300
	7.37	0.85	0.29	0.289	220
	7.69	0.82	0.28	0.277	310
	8.53	0.74	0.25	0.250	400
	8.97	0.70	–	–	001
	9.26	0.68	0.23	0.229	320
	9.76	0.64	0.22	0.218	410
	17.90	0.35	–	–	002

Table S18 (Continued)

	q (nm^{-1})	*d*-spacing (nm)	ratio	ratio (calc.)	*hkl*
(l) **Au16**$^{+}$-PCCp^{-}<br>280 °C (2nd heating)<br>Col_h<br>a = 3.46 nm, c = 0.71 nm<br>M = 3885.02, Z = 1 for ρ = 0.88	2.10	2.99	1.00	1.000	100
	3.64	1.73	0.58	0.577	110
	4.20	1.50	0.50	0.500	200
	5.55	1.13	0.38	0.378	210
	6.31	1.00	0.33	0.333	300
	7.27	0.86	0.29	0.289	220
	7.58	0.83	0.28	0.277	310
	8.41	0.75	0.25	0.250	400
	8.87	0.71	–	–	001
	9.14	0.69	0.23	0.229	320
	9.61	0.65	0.22	0.218	410
	18.71	0.36	–	–	002

[a] The diffraction peaks which corresponded to 002, 003, and 004 were observed at the wide-angle region (Figure S116).

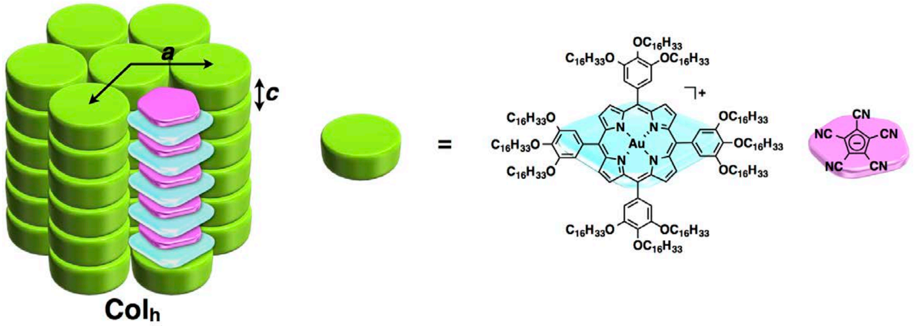

Figure S115 Possible packing model of Au16^{+}-PCCp^{-}, Related to Table 1 and Figure 8.
Possible packing model of **Au16**$^{+}$-PCCp^{-} in a Col_h structure.

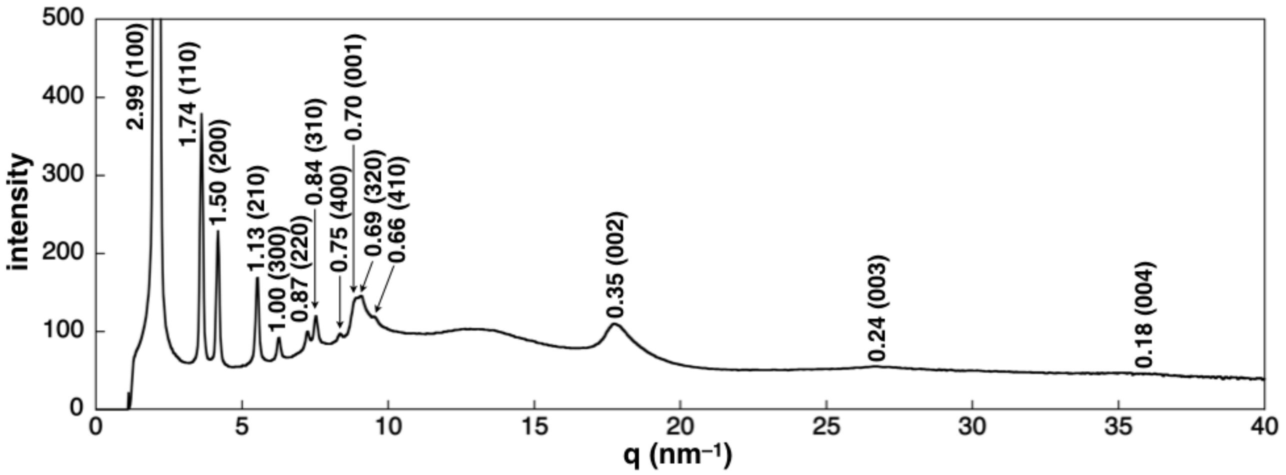

Figure S116 Wide-angle XRD of Au16^{+}-PCCp^{-}, Related to Table 1 and Figure 8.
Wide-angle XRD of **Au16**$^{+}$-PCCp^{-} at 280 °C (1st cooling). The diffraction peak at 0.70 nm is derived from the alternate stacking of porphyrin–Au^{III} complex and PCCp^{-} in the Col_h packing structure (Table S18 and Figure S113–115). Highly ordered charge-by-charge assembly affords higher-order diffractions derived from (001) peak.

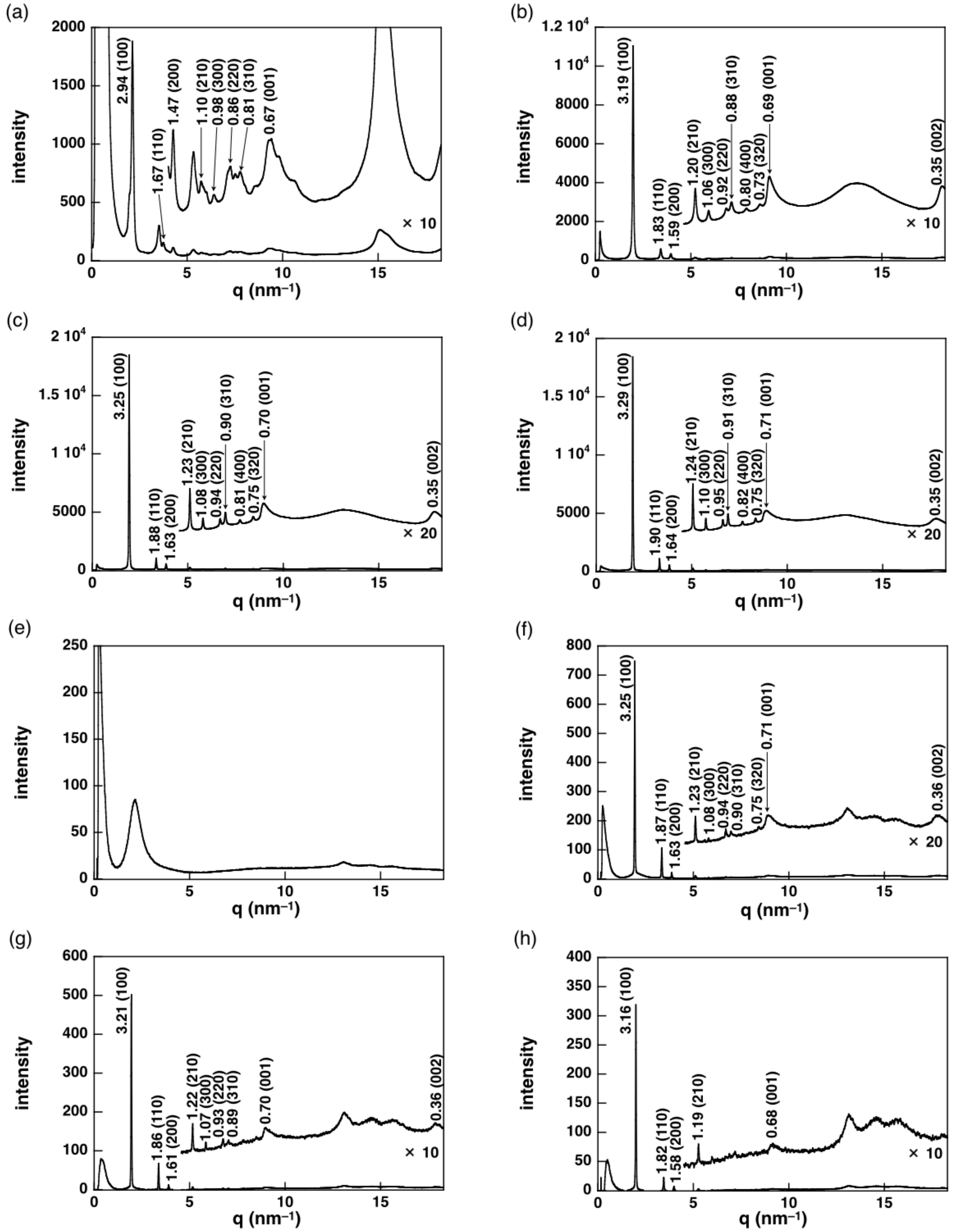

Figure S117 XRD patterns of Au20$^+$-PCCp$^-$, Related to Table 1.

XRD patterns of **Au20**$^+$-PCCp$^-$ at (a) 25 °C (1st heating), (b) 100 °C (1st heating), (c) 200 °C (1st heating), (d) 250 °C (1st heating), (e) 280 °C (1st heating), (f) 250 °C (1st cooling), (g) 200 °C (1st cooling), and (h) 100 °C (1st cooling). The XRD patterns of (a–d,f–h) exhibit Col$_h$ structure (Figure S119).

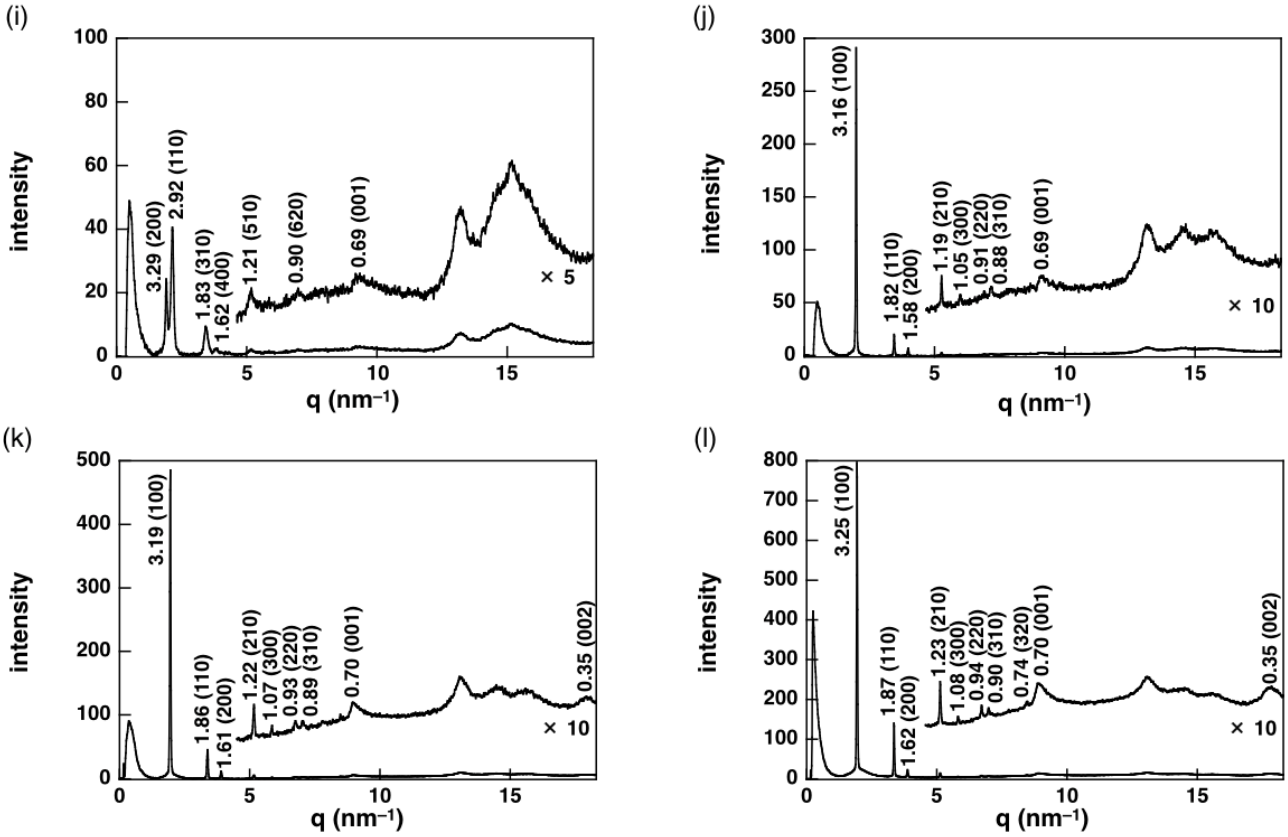

Figure S118 XRD patterns of Au20$^+$-PCCp$^-$, Related to Table 1.
XRD patterns of **Au20**$^+$-PCCp$^-$ at (i) 25 °C (1st cooling), (j) 100 °C (2nd heating), (k) 200 °C (2nd heating), and (l) 250 °C (2nd heating) (Figure labels are continued from Figure S117). The XRD patterns of (i) and (j–l) exhibit Col$_r$ and Col$_h$ structures, respectively (Figure S119).

Table S19 XRD peaks of Au20$^+$-PCCp$^-$, Related to Table 1.
XRD peaks of **Au20**$^+$-PCCp$^-$ at (a) 25 °C (1st heating), (b) 100 °C (1st heating), (c) 200 °C (1st heating), (d) 250 °C (1st heating), (f) 250 °C (1st cooling), (g) 200 °C (1st cooling), (h) 100 °C (1st cooling), (i) 25 °C (1st cooling), (j) 100 °C (2nd heating), (k) 200 °C (2nd heating), and (l) 250 °C (2nd heating) (Figure S117,118). The peaks which can be indexed are represented.[a]

	q (nm^{-1})	*d*-spacing (nm)	ratio	ratio (calc.)	*hkl*
	2.14	2.94	1.00	1.000	100
	3.75	1.67	0.57	0.577	110
(a) **Au20**$^+$-PCCp$^-$	4.27	1.47	0.50	0.500	200
25 °C (1st heating)	5.74	1.10	0.37	0.378	210
Col$_h$	6.40	0.98	0.33	0.333	300
a = 3.39 nm, c = 0.67 nm	7.27	0.86	0.29	0.289	220
M = 4558.32, Z = 1 for ρ = 1.14	7.77	0.81	0.28	0.277	310
	9.41	0.67	–	–	001
	1.97	3.19	1.00	1.000	110
	3.42	1.83	0.57	0.577	110
	3.95	1.59	0.50	0.500	200
(b) **Au20**$^+$-PCCp$^-$	5.23	1.20	0.38	0.378	210
100 °C (1st heating)	5.93	1.06	0.33	0.333	300
Col$_h$	6.85	0.92	0.29	0.289	220
a = 3.69 nm, c = 0.69 nm	7.12	0.88	0.28	0.277	310
M = 4558.32, Z = 1 for ρ = 0.93	7.90	0.80	0.25	0.250	400
	8.61	0.73	0.23	0.229	320
	9.11	0.69	–	–	001
	18.14	0.35	–	–	002

Table S19 (Continued)

	q (nm^{-1})	*d*-spacing (nm)	ratio	ratio (calc.)	*hkl*
	1.93	3.25	1.00	1.000	100
	3.35	1.88	0.58	0.577	110
	3.86	1.63	0.50	0.500	200
(c) **Au20**$^+$-PCCp$^-$	5.11	1.23	0.38	0.378	210
200 °C (1st heating)	5.79	1.08	0.33	0.333	300
Col$_h$	6.70	0.94	0.29	0.289	220
a = 3.75 nm, c = 0.70 nm	6.97	0.90	0.28	0.277	310
M = 4558.32, Z = 1 for ρ = 0.89	7.72	0.81	0.25	0.250	400
	8.42	0.75	0.23	0.229	320
	8.96	0.70	–	–	001
	17.90	0.35	–	–	002
	1.91	3.29	1.00	1.000	100
	3.31	1.90	0.58	0.577	110
	3.83	1.64	0.50	0.500	200
(d) **Au20**$^+$-PCCp$^-$	5.06	1.24	0.38	0.378	210
250 °C (1st heating)	5.74	1.10	0.33	0.333	300
Col$_h$	6.63	0.95	0.29	0.289	220
a = 3.79 nm, c = 0.71 nm	6.90	0.91	0.28	0.277	310
M = 4558.32, Z = 1 for ρ = 0.86	7.65	0.82	0.25	0.250	400
	8.34	0.75	0.23	0.229	320
	8.91	0.71	–	–	001
	17.80	0.35	–	–	002
	1.93	3.25	1.00	1.000	100
	3.36	1.87	0.58	0.577	110
	3.86	1.63	0.50	0.500	200
(f) **Au20**$^+$-PCCp$^-$	5.11	1.23	0.38	0.378	210
250 °C (1st cooling)	5.79	1.08	0.33	0.333	300
Col$_h$	6.71	0.94	0.29	0.289	220
a = 3.75 nm, c = 0.71 nm	6.97	0.90	0.28	0.277	310
M = 4558.32, Z = 1 for ρ = 0.88	8.42	0.75	0.23	0.229	320
	8.87	0.71	–	–	001
	17.69	0.36	–	–	002
	1.96	3.21	1.00	1.000	100
	3.38	1.86	0.58	0.577	110
(g) **Au20**$^+$–PCCp$^-$	3.90	1.61	0.50	0.500	200
200 °C (1st cooling)	5.16	1.22	0.38	0.378	210
Col$_h$	5.85	1.07	0.33	0.333	300
a = 3.71 nm, c = 0.70 nm	6.75	0.93	0.29	0.289	220
M = 4558.32, Z = 1 for ρ = 0.91	7.05	0.89	0.28	0.277	310
	8.96	0.70	–	–	001
	17.89	0.35	–	–	002
(h) **Au20**$^+$-PCCp$^-$	1.99	3.16	1.00	1.000	100
100 °C (1st cooling)	3.45	1.82	0.58	0.577	110
Col$_h$	3.99	1.58	0.50	0.500	200
a = 3.64 nm, c = 0.68 nm	5.26	1.19	0.38	0.378	210
M = 4558.32, Z = 1 for ρ = 0.96	9.18	0.68	–	–	001

Table S19 (Continued)

	q (nm^{-1})	*d*-spacing (nm)	ratio	ratio (calc.)	*hkl*
(i) **Au20**$^{+}$-PCCp^{-}<br>25 °C (1st cooling)<br>Col$_r$ (*c2mm*)<br>a = 6.57 nm, b = 3.26 nm,<br>c = 0.69 nm<br>M = 4558.32, Z = 2 for ρ = 1.03	1.91	3.29	1.00	1.000	200
	2.15	2.92	0.89	0.890	110
	3.42	1.83	0.56	0.554	310
	3.88	1.62	0.49	0.500	400
	5.18	1.21	0.37	0.371	510
	6.97	0.90	0.27	0.277	620
	9.09	0.69	–	–	001
(j) **Au20**$^{+}$-PCCp^{-}<br>100 °C (2nd heating)<br>Col$_h$<br>a = 3.64 nm, c = 0.69 nm<br>M = 4558.32, Z = 1 for ρ = 0.95	1.99	3.16	1.00	1.000	100
	3.45	1.82	0.58	0.577	110
	3.99	1.58	0.50	0.500	200
	5.26	1.19	0.38	0.378	210
	5.99	1.05	0.33	0.333	300
	6.90	0.91	0.29	0.289	220
	7.17	0.88	0.28	0.277	310
	9.09	0.69	–	–	001
(k) **Au20**$^{+}$-PCCp^{-}<br>200 °C (2nd heating)<br>Col$_h$<br>a = 3.69 nm, c = 0.70 nm<br>M = 4558.32, Z = 1 for ρ = 0.92	1.97	3.19	1.00	1.000	100
	3.38	1.86	0.58	0.577	110
	3.91	1.61	0.50	0.500	200
	5.16	1.22	0.38	0.378	210
	5.86	1.07	0.34	0.333	300
	6.75	0.93	0.29	0.289	220
	7.03	0.89	0.28	0.277	310
	9.02	0.70	–	–	001
	17.92	0.35	–	–	002
(l) **Au20**$^{+}$-PCCp^{-}<br>250 °C (2nd heating)<br>Col$_h$<br>a = 3.75 nm, c = 0.70 nm<br>M = 4558.32, Z = 1 for ρ = 0.89	1.93	3.25	1.00	1.000	100
	3.36	1.87	0.58	0.577	110
	3.88	1.62	0.50	0.500	200
	5.13	1.23	0.38	0.378	210
	5.82	1.08	0.33	0.333	300
	6.72	0.94	0.29	0.289	220
	6.99	0.90	0.28	0.277	310
	8.48	0.74	0.23	0.229	320
	13.04	0.48	–	–	001
	17.73	0.35	–	–	002

[a] The diffraction peaks which corresponded to 002, 003, and 004 were observed at the wide-angle region (Figure S120).

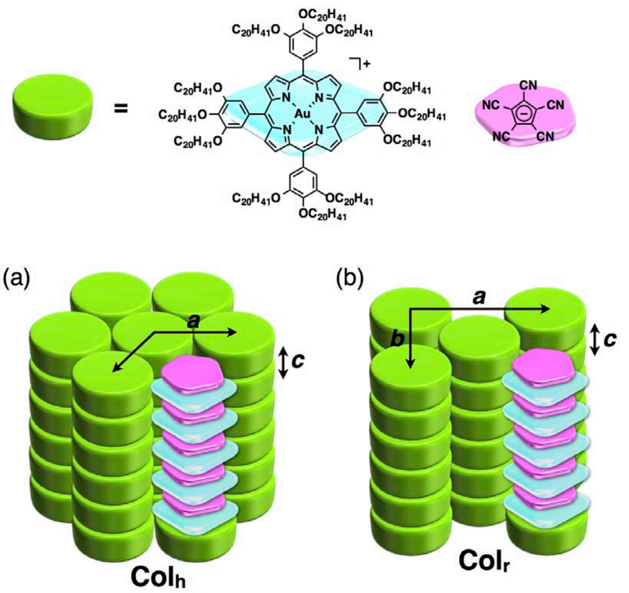

Figure S119 Possible packing models of Au20$^+$-PCCp$^-$, Related to Table 1.
Possible packing models of **Au20**$^+$-PCCp$^-$ in (a) Col$_h$ and (b) Col$_r$ (*c*2*mm*) structures.

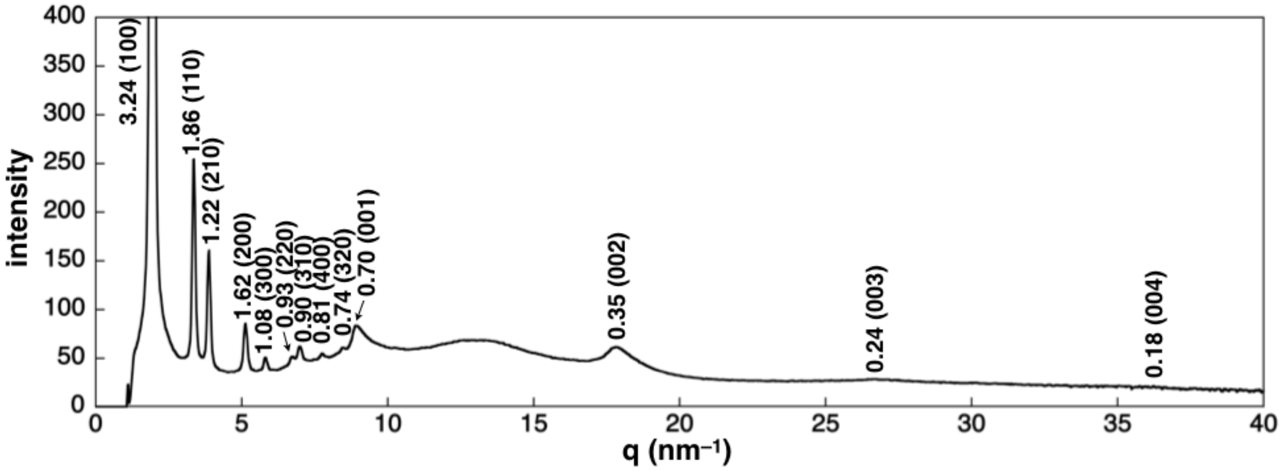

Figure S120 Wide-angle XRD of Au20$^+$-PCCp$^-$, Related to Table 1.
Wide-angle XRD of **Au20**$^+$-PCCp$^-$ at 250 °C (1st cooling). The diffraction peak at 0.70 nm is derived from the alternate stacking of porphyrin–Au^{III} complex and PCCp$^-$ in the Col$_h$ packing structure (Table S19 and Figure S117–119). Highly ordered charge-by-charge assembly affords higher-order diffractions derived from (001) peak.

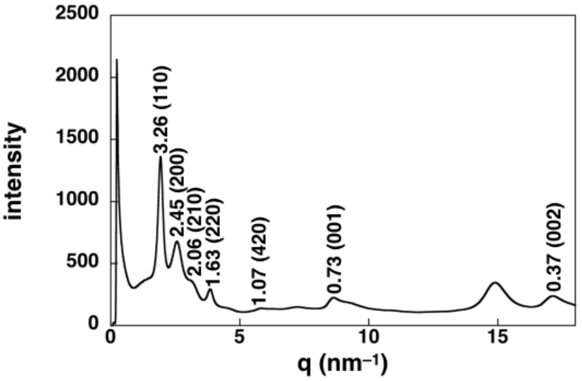

Figure S121 XRD peaks of xerogel, Related to Figure 6.
XRD peaks of xerogel obtained from an octane gel (10 mg/mL) of **Au16**$^+$-PCCp$^-$. The XRD pattern exhibits a Col$_r$ structure (Figure S122).

Table S20 XRD peaks of xerogel, Related to Figure 6.

XRD peaks of xerogel obtained from an octane gel (10 mg/mL) of **Au16**$^{+}$-PCCp^{-} (Figure S121). The peaks which can be indexed are represented.

	q (nm^{-1})	*d*-spacing (nm)	ratio	ratio (calc.)	*hkl*
**Au16**$^{+}$-PCCp^{-}<br>25 °C<br>Col$_r$ (*p2gg*)<br>*a* = 4.90 nm, *b* = 4.36 nm,<br>*c* = 0.73 nm<br>*M* = 3885.02, *Z* = 2 for *ρ* = 0.83	1.98	3.26	1.00	1.000	200
	2.56	2.45	0.75	0.750	110
	3.04	2.06	0.60	0.655	210
	3.86	1.63	0.47	0.499	220
	5.84	1.07	0.31	0.327	420
	8.64	0.73	–	–	001
	17.11	0.37	–	–	002

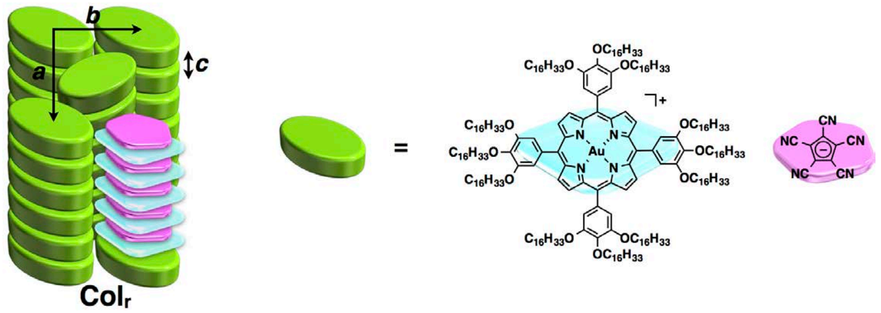

Figure S122 Possible packing model of xerogel, Related to Figure 6.

Possible packing model of xerogel obtained from an octane gel (10 mg/mL) of **Au16**$^{+}$-PCCp^{-} in a Col$_r$ (*p*2*gg*) structure.

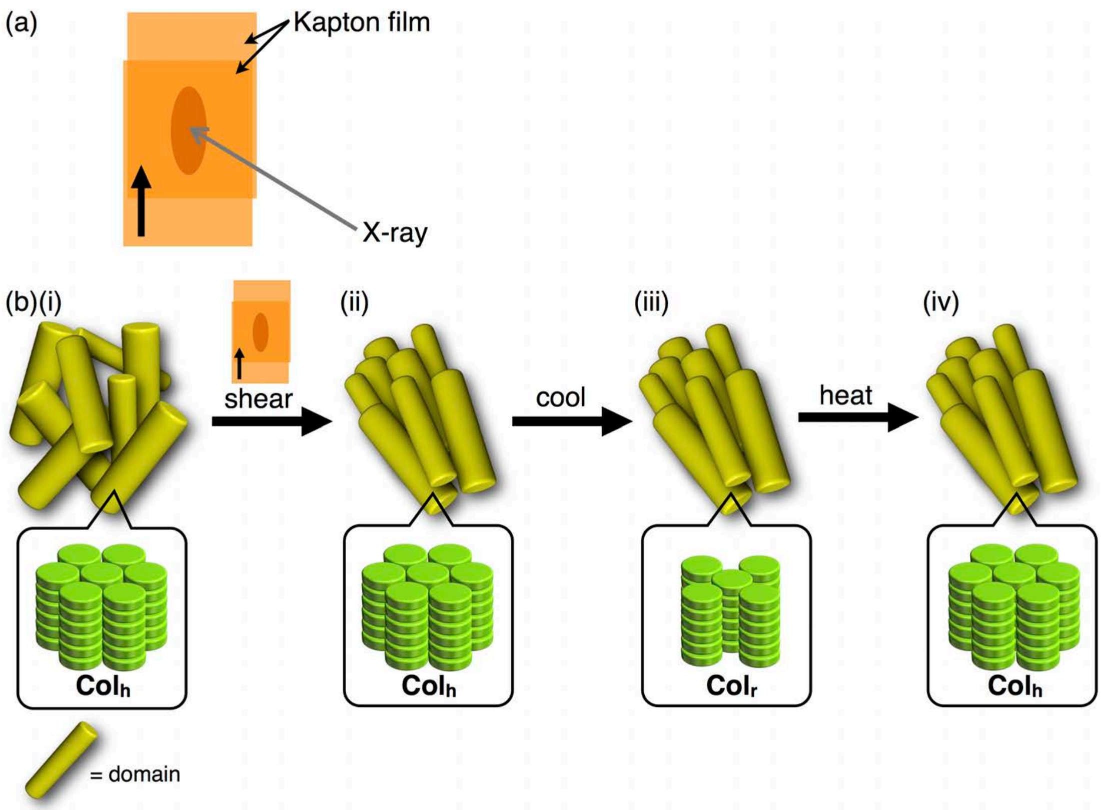

Figure S123 Summary for the XRD measurements of sheared ion-pairing assemblies of Au20^{+}-PCCp^{-}, Related to Figure 9.

Summary for the XRD measurements of sheared ion-pairing assemblies of **Au20**$^{+}$-PCCp^{-} (Figure S125,126): cartoons for (a) the shearing experiment, for which the sample was sheared between two Kapton (polyimide) films, and (b) the orientations of domains (yellow cylinders) (top) and the packing mode inside of the domains (bottom) for (i) the mesophase as the original state, (ii) the sheared sample at 230 °C, (iii) that upon cooling to r.t., and (iv) that upon heating to 100 °C. Here, the detailed investigation for **Au20**$^{+}$-PCCp^{-} was conducted due to the appearance of two different assembling structures as the mesophase and crystalline states. In the mesophase as the original state, domains consisting of a Col$_{h}$ structure were non-oriented with multiple domains ((i)). By shearing in one direction, domains were aligned along the sheared direction ((ii)). An anisotropic orientation caused by shearing was maintained even at 100 °C as the Col$_{h}$ mesophase ((iv)) (Figure S126) after the phase transition from Col$_{r}$ at r.t. ((iii)) (Figure S125). The retention of the domain orientations during thermal processes suggested the contributions of robust packing states based on charge-by-charge assemblies. Such retention of the domain orientations during thermal processes was also observed in the assemblies of pentaalkyl-substituted hexabenzocoronenes (HBCs) with appropriate substituents exhibiting dipoles (Grigoriadis et al., 2010; Haase et al., 2011).

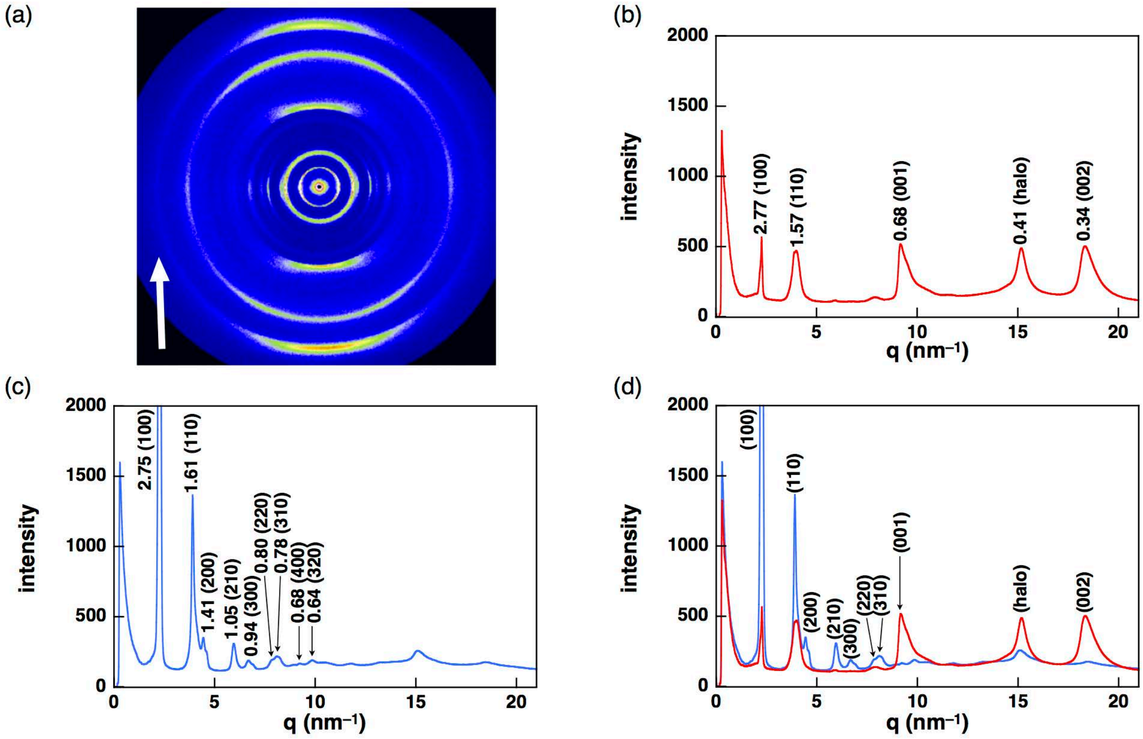

Figure S124 XRD patterns of Au16$^+$-PCCp$^-$ sheared sample, Related to Figure 9.

XRD patterns of **Au16**$^+$-PCCp$^-$ sheared between Kapton (polyimide) films at ca. 250 °C and cooled to r.t. (1st cooling): (a) 2D XRD diffraction pattern with an arrow indicating sheared direction, (b) a diagram of meridional (sheared) direction (90° ± 20°) of 2D XRD, (c) a diagram of equatorial direction (0° ± 20°) of 2D XRD, and (d) a combined diagram including meridional (sheared) (red) and equatorial (blue) directions. 2D XRD diffraction pattern image is slightly tilted due to the sample setting conditions. The diffractions at the smaller angle region assignable to the hexagonal packing increased in the equatorial direction (blue line in (c,d)), whereas the diffractions at the wider angle region including the 001 peak (0.68 nm) enhanced in the meridional direction (red in (b,d)). The 001 (0.68 nm) peak is assignable to the repeating distance of identical π-electronic charged species in the assembly comprising alternately arranged π-electronic ions (charge-by-charge assembly). These results clearly suggest that the hexagonally assembled charge-by-charge columnar assembly is highly oriented by shearing. The enhanced diffraction peak for halo at the angle of ~±20° based on meridional (sheared) direction can be ascribable to the charge-by-charge columnar assembly with the arrangement of laterally rotating porphyrin–Au^{III} complexes with a rotating angle of ~20° (Pisula et al., 2007).

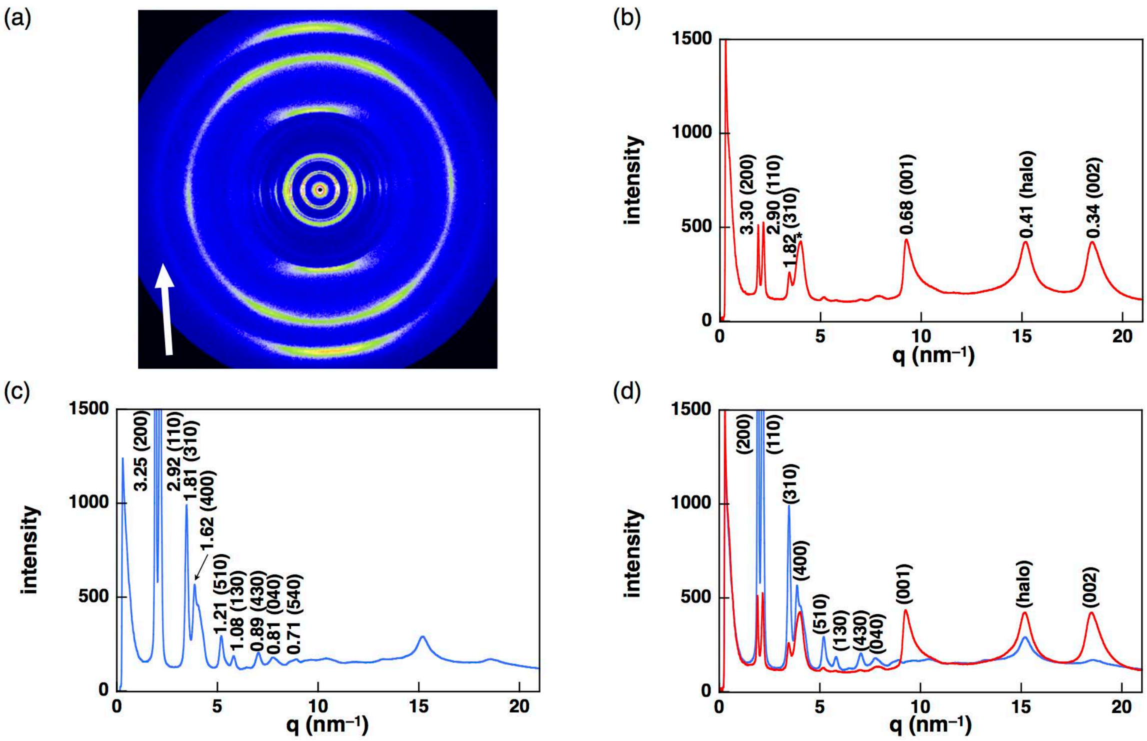

Figure S125 XRD patterns of Au20^{+}-PCCp^{-} sheared sample, Related to Figure 9.

XRD patterns of **Au20**$^{+}$-PCCp^{-} sheared between Kapton (polyimide) films at ca. 230 °C and cooled to r.t. (1st cooling): (a) 2D XRD diffraction pattern with an arrow indicating sheared direction, (b) a diagram of meridional (sheared) direction (90° ± 20°), (c) a diagram of equatorial direction (0° ± 20°), and (d) a combined diagram including meridional (sheared) (red) and equatorial (blue) directions. 2D XRD diffraction pattern image is slightly tilted due to the sample setting conditions. Diffractions of asterisk indicate the diffractions from Kapton film. The diffractions at the smaller angle region assignable to the rectangular packing increased in the equatorial direction (blue line in (c,d)), whereas the diffractions at the wider angle region including the 001 peak (0.68 nm) enhanced in the meridional direction (red in (b,d)). The 001 (0.68 nm) peak is assignable to the repeating distance of identical π-electronic charged species in the assembly comprising alternately arranged π-electronic ions (charge-by-charge assembly). These results clearly suggest that the rectangularly assembled charge-by-charge columnar assembly is highly oriented by shearing (Figure S123b). The enhanced diffraction peak for halo at the angle of ~±20° based on meridional (sheared) direction can be ascribable to the charge-by-charge columnar assembly with the arrangement of laterally rotating porphyrin–Au^{III} complexes with a rotating angle of ~20° (Pisula et al., 2007).

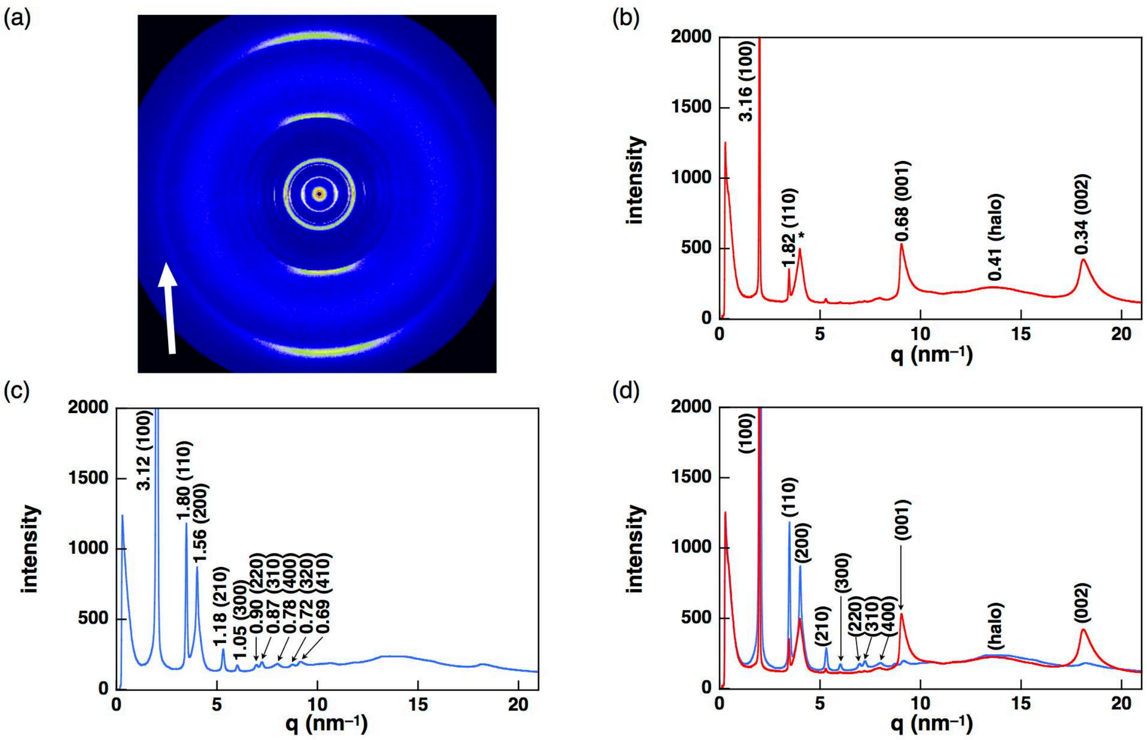

Figure S126 XRD patterns of $Au20^{+}$-$PCCp^{-}$ sheared sample, Related to Figure 9.

XRD patterns of **Au20**$^{+}$-$PCCp^{-}$ sheared between Kapton (polyimide) films at ca. 230 °C, cooled to r.t., and heated to 100 °C (2nd heating): (a) 2D XRD diffraction pattern with an arrow indicating sheared direction, (b) a diagram of meridional (sheared) direction (90° ± 20°), (c) a diagram of equatorial direction (0° ± 20°), and (d) a combined diagram including meridional (sheared) (red) and equatorial (blue) directions. 2D XRD diffraction pattern image is slightly tilted due to the sample setting conditions. Diffractions of asterisk indicate the diffractions from Kapton film. The diffractions at the smaller angle region assignable to the hexagonal packing increased in the equatorial direction (blue line in (c,d)), whereas the diffractions at the wider angle region including the 001 peak (0.68 nm) enhanced in the meridional direction (red in (b,d)). The 001 (0.68 nm) peak is assignable to the repeating distance of identical π-electronic charged species in the assembly comprising alternately arranged π-electronic ions (charge-by-charge assembly). These results clearly suggest that the hexagonally assembled columnar charge-by-charge assembly is highly oriented by shearing. In addition, anisotropic orientation caused by shearing was maintained at 100 °C as a Col_h mesophase after the phase transition from Col_r at r.t. (Figure S123b).

2. Supplemental references

Che, C.M., Sun, R.W.Y., Yu, W.Y., Ko, C.B., Zhu, N., and Sun, H. (2003). Gold(III) porphyrins as a new class of anticancer drugs: cytotoxicity, DNA binding and induction of apoptosis in human cervix epitheloid cancer cells. Chem. Commun. 1718–1719.

Frisch, M.J., Trucks, G.W., Schlegel, H.B., Scuseria, G.E., Robb, M.A., Cheeseman, J.R., Scalmani, G., Barone, V., Mennucci, B., Petersson, G.A., Nakatsuji, H. Caricato, M., Li, X., Hratchian, H.P., Izmaylov, A.F., Bloino, J., Zheng,G., Sonnenberg, J.L., Hada, M., Ehara, M., Toyota, K., Fukuda, R., Hasegawa, J., Ishida, M., Nakajima, T., Honda, Y., Kitao, O., Nakai, H., Vreven, T., Montgomery, Jr., J.A., Peralta, J.E., Ogliaro, F., Bearpark, M., Heyd, J.J., Brothers, E., Kudin, K.N., Staroverov, V.N., Keith, T., Kobayashi, R., Normand, J., Raghavachari, K., Rendell, A., Burant, J.C., Iyengar, S.S., Tomasi, J., Cossi, M., Rega, N., Millam, J.M., Klene, M., Knox, J.E., Cross, J.B., Bakken, V., Adamo, C., Jaramillo, J., Gomperts, R., Stratmann, R.E., Yazyev, O., Austin, A.J., Cammi, R., Pomelli, C., Ochterski, J.W., Martin, R.L., Morokuma, K., Zakrzewski, V.G., Voth, G.A., Salvador, P., Dannenberg, J.J., Dapprich, S., Daniels, A.D., Farkas, Ö., Foresman, J.B., Ortiz, J.V., Cioslowski, J., and Fox, D.J. *Gaussian 09*, Revision D.01, Gaussian, Inc., Wallingford CT, 2013.

Grigoriadis, C., Haase, N., Butt, H.J., Müllen, K., and Floudas, G. (2010). Negative Thermal Expansion in Discotic Liquid Crystals of Nanographenes. Adv. Mater. *22*, 1403–1406.

Haase, N., Grigoriadis, C., Butt, H.J., Müllen, K., and Floudas, G. (2011). Effect of Dipole Functionalization on the Thermodynamics and Dynamics of Discotic Liquid Crystals. J. Phys. Chem. B *115,* 5807–5814.

Kabuto, C., Akine, S., Nemoto, T., Kwon, E. (2009). Release of Software (Yadokari-XG 2009) for Crystal Structure Analyses. J. Cryst. Soc. Jpn. *51*, 218–224.

Maruyama, S., Sato, K., and Iwahashi, H. (2010). Room Temperature Liquid Porphyrins. Chem. Lett. *39*, 714–716.

Nowak-Król, A., Gryko, D., and Gryko, D.T. (2010). *Meso*-Substituted Liquid Porphyrins. Chem. Asian J. *5*, 904–909.

Pisula, W., Tomović, Ž., Watson, M.D., Müllen, K., Kussmann, J., Ochsenfeld, C., Metzroth, T., and Gauss, J. (2007). Helical Packing of Discotic Hexaphenyl Hexa-*peri*-hexabenzocoronenes: Theory and Experiment. J. Phys. Chem. B *111*, 7481–7487.

Sakai, T., Seo, S., Matsuoka, J., and Mori, Y. (2013). Synthesis of Functionalized Tetracyanocyclopentadienides from Tetracyanothiophene and Sulfones. J. Org. Chem. *78*, 10978–10985.

Sasano, Y., Yasuda, N., and Maeda, H. (2017). Deprotonated *meso*-hydroxyporphyrin as a stable π-electronic anion: the building unit of an ion-pairing assembly. Dalton Trans. *46*, 8924–8928.

Stähler, C., Shimizu, D., Yoshida, K., Furukawa, K., Herges, R., and Osuka, A. (2017). Stable Ni^{II} Porphyrin *meso*-Oxy Radical with a Quartet Ground State. Chem. Eur. J. *23*, 7217–7220.

Timkovich, R., and Tulinsky, A. (1977). Coordination and Geometry of Gold in Chloro(α,β,γ,δ-tetraphenylporphinate)gold(III). Inorg. Chem. *16*, 962–963.

Yasuda, N., Murayama, H., Fukuyama, Y., Kim, J. E., Kimura, S., Toriumi, K., Tanaka, Y., Moritomo, Y., Kuroiwa, Y., Kato, K., Tanaka, H., and Takata, M. (2009). X-ray diffractometry for the structure determination of a submicrometre single powder grain. J. Synchrotron Rad. *16*, 352–357.

Yasuda, N., Fukuyama, Y., Toriumi, K., Kimura, S., and Takata, M. (2010). Submicrometer Single Crystal Diffractometry for Highly Accurate Structure Determination. AIP Conf. Proc. *1234*, 147–150.

Wakita, K. *Yadokari-XG, Software for Crystal Structure Analyses*, 2001.
